# Kinetic resolution of amino acids by phosphine oxide catalyzed enantioselective esterification

**DOI:** 10.1038/s41467-026-71469-x

**Published:** 2026-04-13

**Authors:** Ji-Wei Ren, Jing-Hui Sun, Ke-Hang Li, Mu-Ran Lin, Jin-Lan Zeng, Xiao-Mei Ai

**Affiliations:** College of Chemistry and Chemical Engineering, Taishan University, Tai’an, Shandong P. R. China

**Keywords:** Organocatalysis, Synthetic chemistry methodology

## Abstract

Chiral amino acids are essential building blocks in asymmetric synthesis and drug discovery, yet their efficient preparation from racemic mixtures remains challenging. Here we show that a rationally designed phosphine oxide catalyst derived from *L*-pyroglutaminol enables the highly efficient kinetic resolution of racemic amino acids under mild conditions. Using *L*-pyroglutaminol as the esterification reagent, this catalytic system delivers a broad range of chiral esters and recovered amino acids with excellent stereoselectivities (s > 1057). Mechanistic studies suggest that the superior stereocontrol arises from a cooperative double hydrogen-bonding interaction between the catalyst and the pyroglutaminol core. This work provides a practical and scalable approach to enantioenriched amino acids, highlighting the potential of dual chiral cooperative catalysis in asymmetric synthesis.

## Abstract (web summary)

The authors report a kinetic resolution of racemic amino acids with L-pyroglutaminol as an esterification reagent, via a phosphine oxide organocatalytic system, which provides a wide range of chiral esters and recovered amino acids with excellent stereoselectivities.

## Introduction

**Fig. 1:**
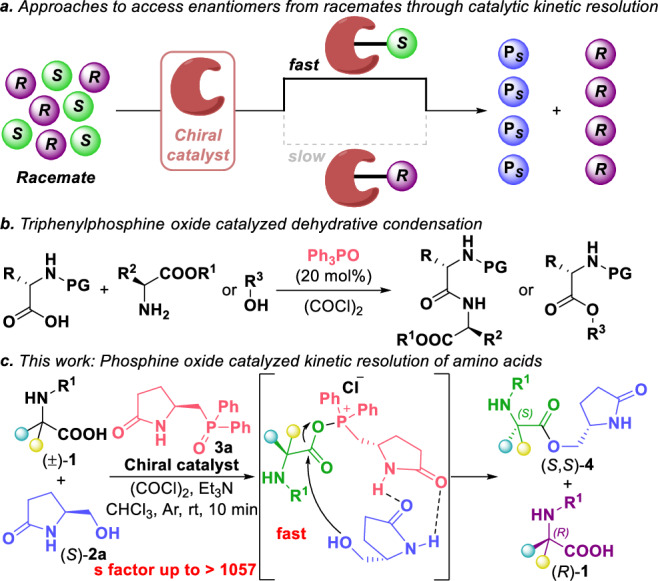
Strategy for the catalytic kinetic resolution of amino acids. **a** Approaches to access enantiomers from racemates through catalytic kinetic resolution. **b** Triphenylphosphine oxide catalyzed dehydrative condensation. **c** Phosphine oxide catalyzed kinetic resolution of amino acids.

Chiral amino acids find applications as versatile building blocks in the synthesis of functional molecules, as a source of chiral information in asymmetric synthesis and as tools to expand and explore the function of native biological machinery^1–8^. Therefore, chiral amino acids represent a class of valuable and indispensable compounds whose stereoselective synthesis is a major objective within synthetic chemists and synthetic biologists^9,10^. An array of effective synthetic strategies for the construction of chiral amino acids have been developed, such as asymmetric hydrogenation and nucleophilic addition of imines^11–18^, enantioselective carbene insertion into N-H bonds of amines or amides^19–23^, stereoselective photobiocatalytic cross-coupling^24–27^, stereocontrolled 1,3-nitrogen migration of carboxylic acids^28^, and asymmetric hydrolysis of amino acid esters^29^. However, identifying a highly enantioselective chiral catalyst for a specific reaction is not always an easy task. An alternative well-established strategy relies on the catalytic kinetic resolution (KR) of a racemic mixture (Fig. 1a)^30–32^. The KR stands out as one of the most practical and straightforward strategies for obtaining enantioenriched molecules and recovering the starting materials, effectively allowing for access to both enantiomers from a single enantiomer of catalyst. Numerous highly efficient catalytic KR processes have been developed that reliably deliver enantiopure compounds, including chiral alcohols^33–37^, monohydrosilanes^38^, organoperoxides^39^, alkynes^40,41^, sulfonyl ketones^42^, amines^43^, imines^44^, sulfoximines^45,46^, aldehydes^47^, phosphindane oxides^48,49^, and heterocyclic compounds^50–53^. Despite this significant progress in the field, the catalytic KR of amino acids remains a challenging task and has been rarely explored^54–58^.

On the other hand, we noticed that the intermediate chlorophosphonium salt (Ph_3_PCl^+^/Cl^-^) generated from triphenylphosphine oxide (Ph_3_PO) and oxalyl chloride ((COCl)_2_) can efficiently activate carboxylate group. In 1977, Masaki and Fukui^59^ reported that the industrial byproduct Ph_3_PO could be easily converted into Ph_3_PCl^+^/Cl^-^, which was subsequently applied in amide and ester synthesis via stoichiometric activation of carboxylic acids, dehydration of amides to nitriles, Appel reaction and others^60–62^. In 2021, Ni and co-workers reported an efficient triphenylphosphine oxide catalyzed amidation and esterification for rapid synthesis of a series of dipeptides, amides and esters (Fig. 1b)^63^. Through analysis of an improved amidation and esterification reaction where catalytic Ph_3_PO was deoxygenated to the Ph_3_PCl^+^/Cl^-^ by (COCl)_2_, we anticipated that chiral phosphine oxides generating chiral chlorophosphonium salts would be a promising strategy for the kinetic resolution of a broad variety of racemic amino acids. Noticeably, pyroglutaminol, a cost-effective chiral feedstock with both enantiomeric forms readily available, was commonly employed as a starting material in asymmetric synthesis owing to its distinctive features, including a predefined chiral center, five-membered lactam ring, and hydroxyl group^64–68^. Conceivably, the lactam moiety in pyroglutaminol offers H-bonding donor and acceptor simultaneously, which could potentially bind to the substrate through double H-bonding. Subsequently, we designed a *L*-pyroglutaminol-derived organocatalyst featuring both H-bonding site and activator site, which can be prepared from *L*-pyroglutaminol and potassium diphenylphosphide. Herein, we disclose the development and application of the *L*-pyroglutaminol-derived phosphine oxide organocatalyst. We report the KR of racemic amino acids with *L*-pyroglutaminol as esterification reagent via a phosphine oxide catalysis under mild conditions, which provides a wide range of chiral amino acids and esters in good yields with excellent diastereoselectivities and enantioselectivities (s > 1057) (Fig. 1c).

## Results

### Optimization studies of kinetic resolution of amino acid

To test the feasibility of our design, the kinetic resolution of racemic amino acid **1a** was explored as a model substrate using 20 mol% of the chiral *L*-pyroglutaminol-derived phosphine oxide catalyst **3a** and 1.5 equiv of oxalyl chloride and *L*-pyroglutaminol as the esterification reagent in chloroform provided the ester (*S*,*S*)-**4a** with 98:2 dr and the recovered (*R*)-**1a** with >99% ee, with excellent selectivity factor (s > 259) (Table 1, Entry 1). The absolute configuration of the recovered starting material **1a** was unambiguously determined by the optical rotation analysis (Dextrorotation) and that of other recovered starting materials by analogy. Noticeably, the reaction was completed within 10 min. The study of variation of the chiral phosphine oxide catalysts indicated that only the catalyst **3e** gave decent kinetic resolution performances (s = 20), while other catalysts all provided poor results (Table 1, Entries 2–5). When **3a** was replaced with the other enantiomer **3 f**, the stereoselectivities decreased considerably (Table 1, Entry 6). Thereafter, a series of solvents were evaluated (Table 1, Entries 7–10). The reaction could also be conducted in toluene (PhMe), tetrahydrofuran (THF) or acetonitrile (MeCN), but the stereoselectivities would drop dramatically. The reaction in 1,2-dichloroethane (DCE) could provide good kinetic resolution performance (s = 68). Further reduction of catalyst loading to 10 mol%, a slightly lower selectivity factor (107) was observed (Table 1, Entry 11). In the absence of catalyst **3a**, none of the desired product formed in 10 min, indicating that the catalyst was essential (Table 1, Entry 12). The racemic ester was obtained in the presence of triphenylphosphine oxide. The reactions of various alcohols and catalysts were also evaluated under the optimized conditions, while all provided poor results (please see Supplementary Information for details).

**Table 1:** Optimization of kinetic resolution of amino acid 1a^a^

Entry	Variation from the standard conditions	4a Yield (%)^b^	4a dr^c^	1a Yield (%)^b^	1a ee (%)^c^	S^d^
1	None	44	98:2	40	>99	>259
2	**3b** instead of **3a**	51	51:49	38	0	–
3	**3c** instead of **3a**	31	52:48	46	2	1
4	**3 d** instead of **3a**	48	56:44	32	10	1
5	**3e** instead of **3a**	46	89:11	41	80	20
6	**3 f** instead of **3a**	49	53:47	30	3	1
7	PhMe instead of CHCl_3_	32	95:5	54	43	29
8	THF instead of CHCl_3_	35	93:7	49	51	22
9	MeCN instead of CHCl_3_	20	64:36	67	18	2
10	DCE instead of CHCl_3_	39	96:4	49	87	68
11	10 mol% catalyst **3a**	39	97:3	42	92	107
12	Without catalyst	NR	–	–	–	–

^a^Unless otherwise noted, all reactions were carried out using amino acid **1a** (0.5 mmol, 1.0 equiv.), *L*-pyroglutaminol **2a** (0.3 mmol, 0.6 equiv.) and catalyst **3a** in chloroform (1.0 mL) and oxalyl chloride (0.75 mmol, 1.5 equiv.) and triethylamine (0.75 mmol, 1.5 equiv.) were added in sequence at ambient temperature in argon.

^b^Isolated yield.

^c^Determined by HPLC on a chiral stationary phase.

^d^Selectivity factor (s) = ln[(1-C)(1-ee_s_)]/ln[(1-C)(1+ee_s_)], C = ee_s_/(ee_s_+de_p_).

### Kinetic resolution of amino acids

**Fig. 2:**
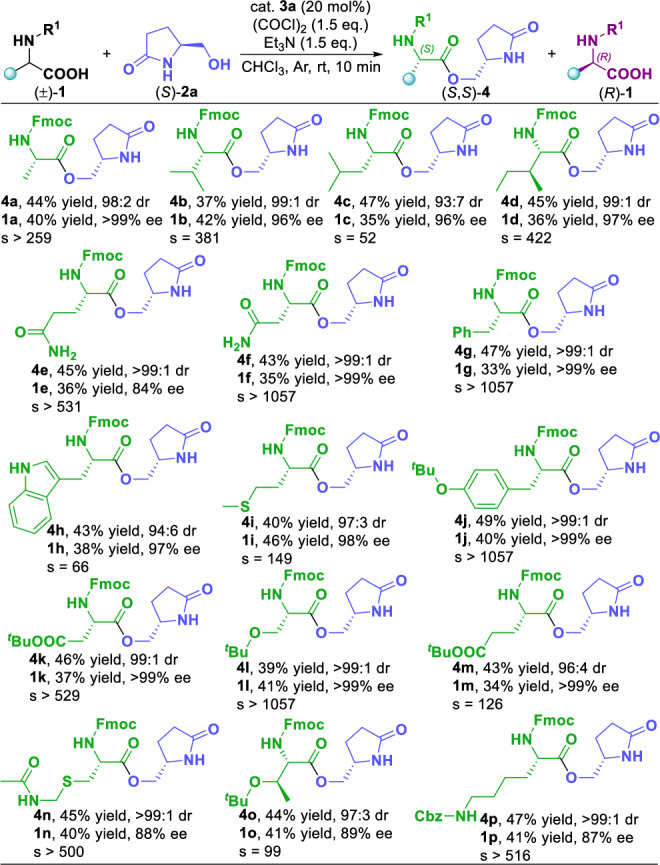
Scope of natural amino acids. Unless otherwise noted, all reactions were carried out using amino acids **1** (0.5 mmol, 1.0 equiv.), *L*-pyroglutaminol **2a** (0.3 mmol, 0.6 equiv.) and catalyst **3a** in chloroform (1.0 mL) and oxalyl chloride (0.75 mmol, 1.5 equiv.) and triethylamine (0.75 mmol, 1.5 equiv.) were added in sequence at ambient temperature in argon.

We next investigated the scope of this method for catalytic kinetic resolution of natural amino acids (Fig. 2). An array of racemic amino acids **1a**-**1d** bearing *α*-alkyl groups, including methyl, isopropyl, isobutyl, and sec-butyl, serves as effective substrates, yielding both ester products and remaining amino acids in good yields and excellent enantioselectivities (s = 52–422). The amino acids **1e** and **1 f**, whose side-chain contains an amide, were well tolerated with a higher level of enantiocontrol (s = 531 and 1057). Substrates **1 g** and **1 h** bearing either benzyl or 3-indolyl group also proved to be effective components, exhibiting enantioselectivities of 1057 and 66, respectively. Methionine (Met) **1i** was also a suitable component (s = 149). Notably, good yields and excellent enantio-discrimination (s = 99–1057) were observed for diverse side-chain protected amino acids (Tyr, Asp, Ser, Glu, Cys, Thr, Lys) **1j**-**1p**. The electronic effects of side chain substituents demonstrated no significant correlation with enantioselectivity.

**Fig. 3:**
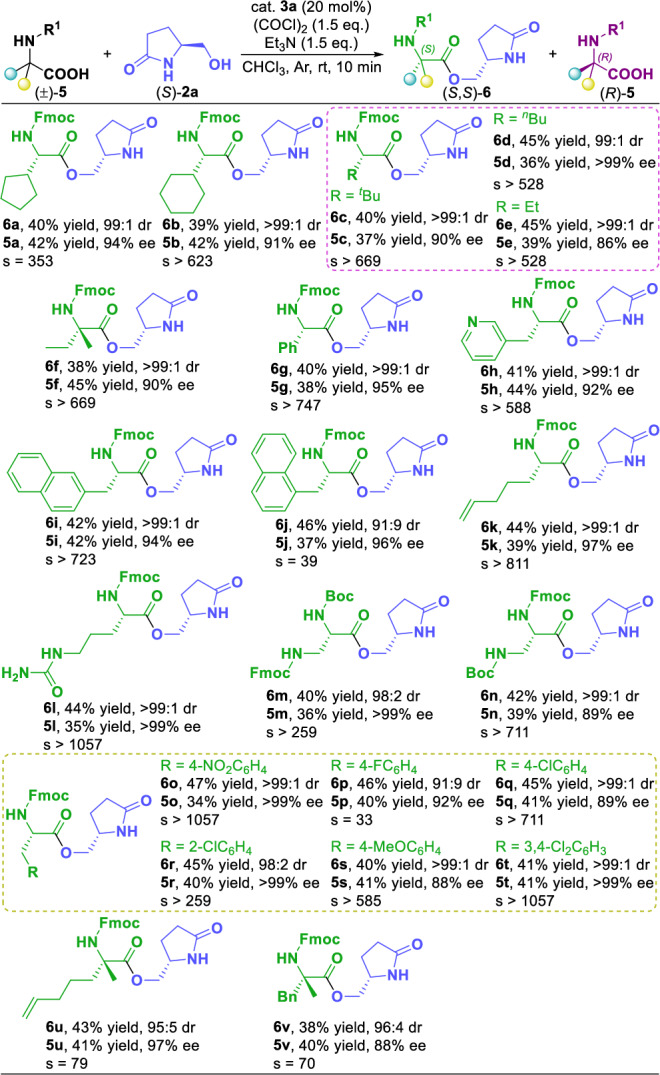
Scope of non-natural amino acids. Unless otherwise noted, all reactions were carried out using amino acids **5** (0.5 mmol, 1.0 equiv.), *L*-pyroglutaminol **2a** (0.3 mmol, 0.6 equiv.) and catalyst **3a** in chloroform (1.0 mL) and oxalyl chloride (0.75 mmol, 1.5 equiv.) and triethylamine (0.75 mmol, 1.5 equiv.) were added in sequence at ambient temperature in argon.

Second, the non-natural amino acids were also fairly broad in scope (Fig. 3). Good yields and excellent enantioselectivities (s = 353–669) were observed for diverse *α*-alkyl moieties that vary in steric demand from ethyl to cyclohexyl (**5a**-**5e**). Variations in the steric hindrance of the side chain resulted in only minor changes to enantioselectivity. The same catalyst can facilitate the enantioselective KR of *α*,*α*-dialkyl substituted amino acid **5 f**, yielding the corresponding enantioenriched amino acid and ester. The amino acids **5g**-**5j**, whose side-chain contains aryl group (phenyl, pyridyl, naphthyl), also proved to be effective components (s = 39–747). The amino acids **5k** and **5 l**, which contain alkene and urea moieties in their side-chains, exhibited good tolerance and demonstrated excellent enantiocontrol (s = 811 and 1057). The side-chain protected amino acids **5 m** and **5n** also reacted smoothly to give both esters and remaining amino acids in good yields and excellent enantioselectivities (s = 259 and 711). An array of racemic amino acids **5o**-**5t** bearing electronically varied *α*-benzyl groups, including *ortho*-, *meta*-, and *para*- substituted compounds, serves as effective substrates, providing the corresponding products with good yield and excellent enantiocontrol (s = 33–1057). The electronic effects of side chain substituents demonstrated no significant correlation with enantioselectivity. The *α*,*α*-dialkyl substituted amino acids **5 u** and **5 v** also reacted smoothly to give both esters and remaining amino acids in good yields and excellent enantioselectivities (s = 79 and 70). The steric effect of *α*,*α*-dialkyl substituted amino acids has a significant impact on the enantioselectivity. Specifically, the steric hindrance substrates exhibited a moderate reduction in enantioselectivity control (**5 u** *vs* **5k** and **5 v** *vs* **1 g**).

Compared to existing methods, this organocatalytic approach offers distinct advantages in efficiency, scope, and practicality. Enzymatic KR (e.g., using hydrolases/deaminases) typically achieves moderate selectivity (s ≈ 20–200) but suffers from narrow substrate scope (limited to natural amino acids), sensitivity to pH/temperature, and high enzyme costs^54–58^. In contrast, our method achieves excellent selectivity (s up to 1057) within 10 min under mild conditions, accommodates broad substrate diversity-including natural/non-natural amino acids, sterically demanding *α*,*α*-dialkyl substituted amino acids. While enzymatic KR remains viable for aqueous biotransformations, this organocatalytic system bridges critical gaps in efficiency, versatility, and scalability, providing a practical, sustainable alternative for synthesizing enantiopure amino acid building blocks.

### Gram-scale reaction, control experiments and nonlinear effect experiment

**Fig. 4:**
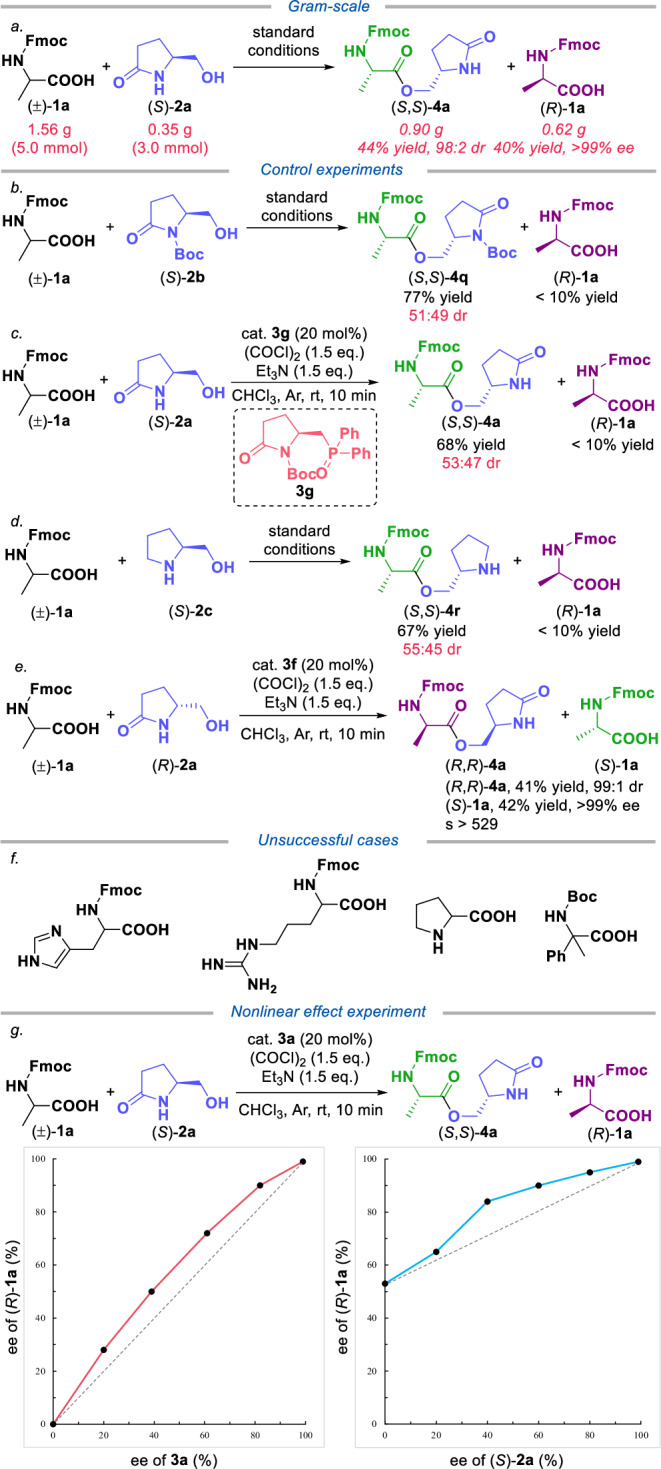
Gram-scale reaction, control experiments, unsuccessful cases and nonlinear effect experiment. **a** The 5 mmol scale reaction. **b**–**e** Control experiments. **f** Unsuccessful cases. **g** Nonlinear effect experiments of 3a and 2a.

The practicality and scalability of this protocol were successfully demonstrated by performing the reaction for **1a** at 5 mmol scale under the optimal conditions to give comparable result (s > 259) without any loss of efficiency (Fig. 4a). Aiming at better understanding the reaction mechanism, control experiments were carried out under the optimal conditions (Fig. 4b–e). *N*-Protected pyroglutaminol **2b,** *N*-protected catalyst **3 g** and *L*-prolinol **2c** were employed to intentionally mask the hydrogen-bonding sites. Not surprisingly, the corresponding esters (**4q,** **4a** and **4r**) were obtained in markedly reduced diastereoselectivities. These results suggested that lactams in both pyroglutaminol and catalyst had sizeable influence on the observed diastereoselectivity. The KR of **1a** was carried out using catalyst **3 f** and *D*-pyroglutaminol (*R*)-**2a** provided the enantiomer (*R*,*R*)-**4a** with 99:1 dr and the recovered (*S*)-**1a** with >99% ee, with excellent selectivity factor (s > 529) (Fig. 4e). These results demonstrated that the chiral environments of both the catalyst and pyroglutaminol synergistically govern the stereoselectivity. Moreover, this statement was also confirmed through ^1^H NMR spectroscopic studies on the mixture of (*S*)-**2a** with **3a** with a molar ratio of 1:1, in which upfield shifts of 0.27 ppm and downfield shifts of 0.11 ppm were induced for the N-H signals in **2a** and **3a**, respectively (see SI for spectra). Additional diversely substituted amino acids were also evaluated under the optimal conditions (Fig. 4f). Unfortunately, the corresponding products were not obtained. This outcome may be attributed to interference from alternative reactive sites, leading to the formation of undefined byproducts.

In addition, we carried out a nonlinear effect experiment for elucidating the nature of the interaction. We observed a small but positive nonlinear effect (Fig. 4g, left, please see Supplementary Information and Supplementary Data for details), which indicates that more than one molecule of **3a** was likely to be involved in the transition state of the enantiodifferentiating step. To gain deeper mechanistic insights and reconcile the observed positive nonlinear effect (Fig. 4g, left), we conducted a complementary nonlinear effect experiment by varying the enantiomeric purity of the chiral esterification reagent, *L*-pyroglutaminol **2a**, while employing enantiopure catalyst **3a**. Intriguingly, a distinct trend emerged: the enantiopurity of the recovered amino acid (*R*)-**1a** increased nonlinearly from 53% ee (when **2a** was racemic) to 99% ee (when **2a** was enantiopure) (Fig. 4g, right, please see Supplementary Information and Supplementary Data for details). This nonlinear correlation underscores two critical points. First, the catalyst **3a** itself possesses an intrinsic enantioselectivity (baseline of 53% ee), which forms the primary chiral framework for substrate discrimination. Second, the matched chiral environment of (*S*)-**2a** acts in a cooperative manner to dramatically amplify and perfect this stereocontrol. This interpretation is strongly corroborated by our earlier observations: the drastic drop in selectivity when the mismatched catalyst enantiomer **3 f** was used with (*S*)-**2a** (Table 1, entry 6), and the full restoration of high selectivity when both components were switched to the matched **3 f**/(*R*)-**2a** pair (Fig. 4e), which unambiguously established the necessity of chiral matching between the catalyst and the esterification reagent. When the catalyst’s ee decreases, the mismatched catalyst enantiomer **3 f** present in the mixture can engage in detrimental interactions with the ever-present, enantiopure (*S*)-**2a**. These unfavorable interactions presumably disrupt the finely tuned double H-bonding network that is optimal for the matched **3a**/(*S*)-**2a** pair, thereby depressing the overall selectivity in a disproportionate, nonlinear manner. This explanation elegantly unifies all experimental observations: it preserves the 1:1 catalyst-substrate interaction mode depicted in the DFT calculated transition state, which represents the optimal productive pathway for the matched pair, while attributing the nonlinear phenomenon to inhibitory interactions from mismatched catalyst components. It conclusively highlights that the supreme stereocontrol (s > 1057) originates from a synergistic, dual-chiral induction where the phosphine oxide catalyst provides the principal stereodifferentiating element, and the chiral pyroglutaminol reagent serves as an essential co-operative partner that refines and maximizes fidelity through specific, match-dependent non-covalent interactions.

### Density functional theory calculations and proposed reaction mechanism

**Fig. 5:**
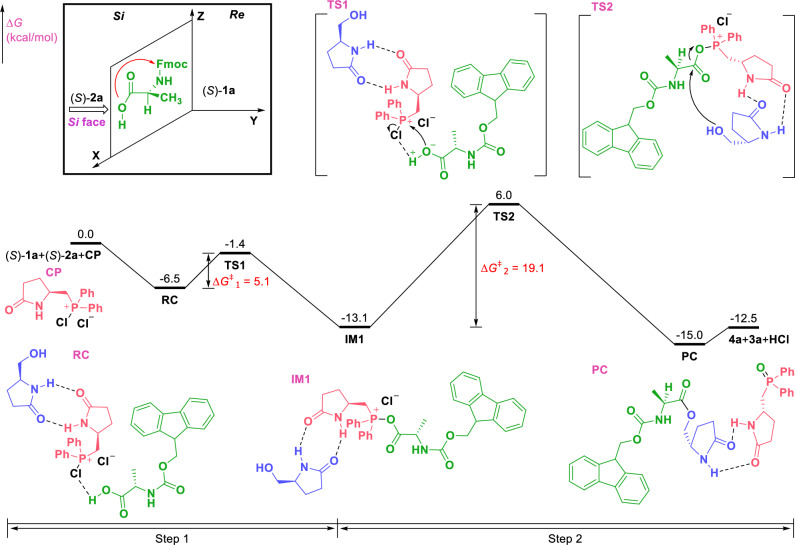
Density functional theory calculations. Relative energy profiles (in kcal/mol) of nucleophilic attack along the *Si* face of the substrate (*S*)-**1a** plane obtained via the B3LYP-D3/6-311 G(d,p)/SMD(CHCl_3_) method. Step 1, nucleophilic attack of chlorophosphonium **CP** with amino acid **1a** formed acyl phosphonium salt; Step 2, nucleophilic addition of *L*-pyroglutaminol **2a** with acyl phosphonium salt.

On the basis of the above experimental results, in order to gain greater insights into the origin of stereoselectivity of the KR, density functional theory (DFT) was employed to study **3a**-catalyzed esterification of amino acid **1a** and *L*-pyroglutaminol **2a**. Both steps of the KR reaction mechanism were as follows (Fig. 5): nucleophilic attack of chlorophosphonium **CP** with amino acid **1a** formed acyl phosphonium salt (step 1), and nucleophilic addition of *L*-pyroglutaminol **2a** with acyl phosphonium salt (step 2). According to our previous study^62^, triphenylphosphine oxide and oxalyl chloride rapidly formed chlorophosphonium. Step 1 commences via H-bonding between chlorophosphonium **CP**, amino acid **1a**, and *L*-pyroglutaminol **2a**, producing complex **RC**. The carboxyl’s oxygen atom acts as the nucleophilic site, allowing nucleophilic attack of **1a** to chlorophosphonium **CP** via transition state **TS1** with the energy barrier of 5.1 kcal/mol. In intermediate **IM1**, double H-bondings were formed spontaneously between *L*-pyroglutaminol **2a** and acyl phosphonium salt through the perfectly matched lactam moieties. In step 2, using the oxygen atom in the hydroxyl group as the nucleophilic site, nucleophilic addition of *L*-pyroglutaminol **2a** to acyl phosphonium salt occurred along the *Si* face of amino acid **1a** via transition state **TS2** with an energy barrier of 19.1 kcal/mol. Finally, product complex **PC** was formed, releasing catalyst **3a**, (*S*,*S*)-**4a** and hydrogen chloride. Comparison of the relative free energy of **TS1** (-1.4 kcal/mol) and **TS2** (6.0 kcal/mol) revealed that the latter was the rate-determining step.

**Fig. 6:**
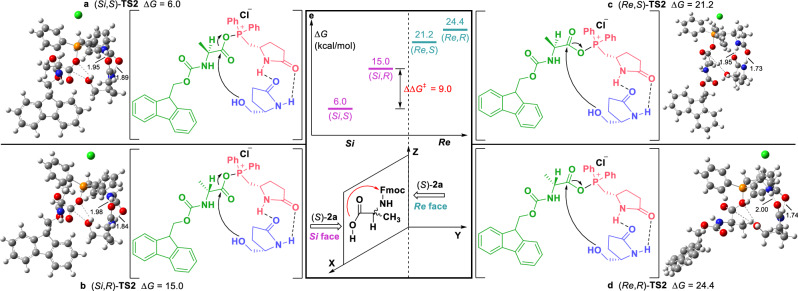
DFT-optimized structures (bond lengths, Å) and relative free energies (Δ*G*, kcal/mol) of transition state. **a** (*Si*,*S*)-**TS2** and **b** (*Si*,*R*)-**TS2** along the *Si* face of the substrate **1a** plane, as well as (**c**) (*Re*,*S*)-**TS2** and **d** (*Re*,*R*)-**TS2** along the *Re* face of the substrate **1a** plane, obtained by the B3LYP-D3/6-311 G(d,p)/SMD(CHCl_3_) method.

The free energy and geometric property of the transition states were explored in detail in order to comprehensively understand the stereoselectivity of the reaction (Fig. 6). As shown in Fig. 6a, b, the relative free energy of (*Si*,*S*)-**TS2** (Δ*G* = 6.0 kcal/mol, leading to product (*S*,*S*)-**4a**) was 9.0 kcal/mol (ΔΔG^⧧^) lower than that of (*Si*,*R*)-**TS2** (Δ*G* = 15.0 kcal/mol, leading to product (*R*,*S*)-**4a**), which indicated that (*S*,*S*)-**4a** was the dominant product and showed good agreement with the experimental results (Table 1, Entry 1). When *L*-pyroglutaminol **2a** attacked amino acid **1a** from the *Re* face (Fig. 6c, d), the relative free energy of (*Re*,*S*)-**TS2** (Δ*G* = 21.2 kcal/mol, leading to product (*S*,*S*)-**4a**) was 3.2 kcal/mol lower than that of (*Re*,*R*)-**TS2** (Δ*G* = 24.4 kcal/mol, leading to product (*R*,*S*)-**4a**), which was consistent with the experimental results ((*S*,*S*)-**4a** as the dominant product). Notably, the relative free energies of transition states (*Re*,*S*)-**TS2** and (*Re*,*R*)-**TS2** were much higher than those of (*Si*,*S*)-**TS2** and (*Si*,*R*)-**TS2**, leading to the *Si* face attack being the dominant attack pathway. As shown in Fig. 6e, the theoretical chemical study indicates that, to identify the correct transition state, a comprehensive comparison of the *Si* and *Re* face attacks was required. Importantly, the relative free energy of transition state (*Si*,*S*)-**TS2** was the lowest, corresponding to the key selective step and the major isomer (*S*,*S*)-**4a**.

**Fig. 7:**
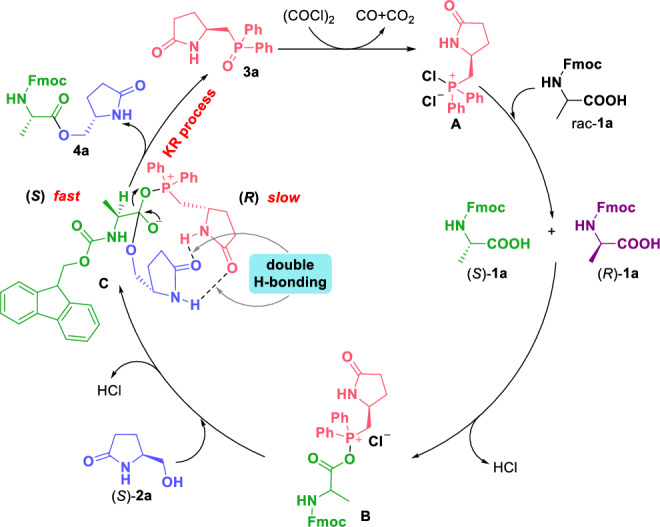
Proposed reaction mechanism. Based on the control experiments and DFT calculations, a possible KR mechanism was proposed.

Based on the related literature^23^, the control experiments and DFT calculations, a possible KR mechanism was proposed (Fig. 7). Initially, phosphine oxide catalyst **3a** reacted with oxalyl chloride to generate chlorophosphonium **A**. The in situ generated intermediate **A** subsequently acts as an activator for the amino acid, leading to the formation of an acyl phosphonium salt **B**. The key intermediates **A** and **B** were successfully characterized by high-resolution mass spectrometry (see Supplementary Information for details). Finally, *L*-pyroglutaminol **2a** underwent a nucleophilic addition to generate the ester **4a** and phosphine oxide catalyst **3a** to complete the catalytic cycle. Among the amino acids, the reaction rate of the *S*-enantiomer was faster, and (*R*)-**1a** remained. Presumably, double H-bondings were formed spontaneously between *L*-pyroglutaminol **2a** and phosphine oxide catalyst **3a** through the perfectly matched lactam moieties. The developed catalyst demonstrated excellent stereocontrol and catalytic activity in the KR of racemic amino acids, which presumably benefited from an intimate double H-bonding interaction.

## Discussion

In conclusion, we successfully developed a *L*-pyroglutaminol-derived phosphine oxide organocatalyst featuring both H-bonding site and activator site. We reported the highly efficient KR of racemic amino acids with *L*-pyroglutaminol as an esterification reagent catalyzed by phosphine oxide under mild conditions, which provides a wide range of chiral esters and recovered amino acids in good yields with excellent selectivities (s > 1057). The developed catalyst demonstrated excellent stereocontrol and catalytic activity in the KR of racemic amino acids, which presumably benefited from an intimate double H-bonding interaction between the pyroglutaminol core and the catalyst. Density functional theory calculations were performed to illustrate the origin of enantiodiscrimination during the KR process. Our system has the advantages of a short reaction time (less than 10 min), high kinetic resolution efficiency, good functional tolerance, broad substrate scope (36 examples) and atom-economy (only CO, CO_2_, and ammonium salt as wastes at the end of reaction). Development of this phosphine oxide catalyst could inspire the emergence of versatile pyroglutaminol-based organocatalysts as well as their broader application in asymmetric synthesis.

## Methods

### General procedure

In a 15 mL oven-dried Schlenk tube, racemic amino acids (0.5 mmol, 1.0 equiv.), *L*-pyroglutaminol (0.3 mmol, 0.6 equiv.) and catalyst (0.1 mmol, 20 mol %) were well mixed in chloroform (1.0 mL). Then oxalyl chloride (0.75 mmol, 1.5 equiv.) and triethylamine (0.75 mmol, 1.5 equiv.) were added in sequence at ambient temperature under argon atmosphere. The resulting mixture was stirred at room temperature for 10 min. Subsequently, the mixture was partitioned between EtOAc (70 mL) and H_2_O (30 mL) at room temperature. The organic layer was washed with saturated brine (30 mL × 2), dried over Na_2_SO_4_, and concentrated *in vacuo*. The resulting residue was dissolved in dichloromethane (10 mL) and 1 M aqueous solution NaOH (3 mL). The organic layer and the aqueous layer were separated. The organic layer was washed with saturated brine (10 mL × 2), dried over Na_2_SO_4_, and concentrated in vacuo. The resulting residue was purified via silica gel column chromatography to yield esters. The aqueous layer was washed with dichloromethane (10 mL × 2). The organic layer was discarded. The aqueous layer was made acidic with excess 1 M aqueous solution HCl (to pH ~5) and was extracted with dichloromethane (10 mL × 2). The combined organic layer was washed with brine (20 mL × 2), dried over Na_2_SO_4_, then filtered and evaporated to afford the recovered amino acids.

## Supplementary information

Supplementary Information

Description of Additional Supplementary Files

Supplementary Data 1

Supplementary Data 2

Transparent Peer Review file

## Data Availability

The authors declare that the data supporting the findings of this study are available within the paper and its Supplementary Information and Supplementary Data files. Should any raw data files be needed in another format they are available from the corresponding author upon request.
