# Supplementary material for: Kinetic resolution of amino acids by phosphine oxide catalyzed enantioselective esterification
Source: Nat Commun. 2026 Apr 13;17:5157. doi: 10.1038/s41467-026-71469-x (PMC13250068; doi:10.1038/s41467-026-71469-x)
Supplement: Supplementary file 1 — Supplementary Information [file 41467_2026_71469_MOESM1_ESM.pdf]

Supplementary Information

Kinetic Resolution of Amino Acids by Phosphine Oxide Catalyzed Enantioselective Esterification

Ji-Wei Ren,*[1] Jing-Hui Sun,[1] Ke-Hang Li,[1] Mu-Ran Lin,[1] Jin-Lan Zeng,[1] and Xiao-Mei Ai[1]

[1]College of Chemistry and Chemical Engineering, Taishan University, Tai'an, Shandong, 271000, P. R. China. e-mail: renjiwei@tsu.edu.cn

Table of Contents

1. **General experimental information**······1

2. **Experimental procedures and characterization data**······2

   2.1. Synthesis of catalysts **3a**, **3f** and **3g**······2

   2.2. Optimization studies······4

   2.3. General procedure for the kinetic resolution of amino acids······6

   2.4. General procedure for the reaction of amino acid and alcohols······30

   2.5. Synthetic procedure of 5 mmol scale model reaction······32

   2.6. Control experiments······32

   2.7. High-resolution mass spectrometry of the intermediates **A** and **B** in Figure 7······37

3. **NMR Spectra** ······38

4. **Crude ^{1}H NMR Spectra** ······110

5. **Chiral HPLC Spectra**······128

6. **Quantum chemical calculations**······209

7. **References**······241

1. General experimental information

Unless otherwise noted, all the substrates and reagents were purchased from commercial suppliers and used without further purification, which were known compounds. ^{1}H NMR spectra were recorded at 500 MHz. The chemical shifts were recorded in ppm relative to tetramethylsilane and with the solvent resonance as the internal standard. Data were reported as follows: chemical shift, multiplicity (s = singlet, d = doublet, t = triplet, q = quartet, m = multiplet), coupling constants (Hz), integration. ^{13}C NMR data were collected at 125 MHz with complete proton decoupling. ^{31}P NMR data were collected at 203 MHz with complete proton decoupling. Chemical shifts were reported in ppm from the tetramethylsilane with the solvent resonance as internal standard. Diastereomeric ratios (dr) and enantiomeric excesses (ee) were determined by HPLC analysis on Agilent 1200 Infinity instruments with Daicel chiral columns. Optical rotations were measured on an Anton Paar MCP 100 automatic polarimeter. High resolution mass spectroscopy (HRMS) was performed on Thermo Q Exactive Plus (FTMS ESI) mass spectrometer and acetonitrile was used to dissolve the sample. Column chromatography was carried out on silica gel (200-300 mesh).

2. Experimental procedures and characterization data

2.1. Synthesis of catalysts 3a, 3f and 3g

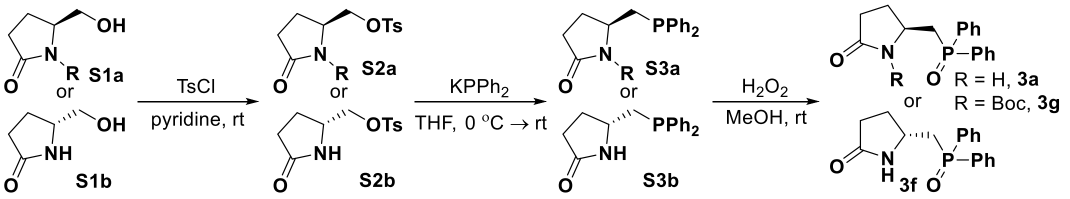

To a solution of *p*-toluenesulfonyl chloride (4.55 g, 22 mmol) in pyridine (15 mL) was added, the solution of pyroglutaminol (2.30 g, 20 mmol) in pyridine (20 mL). The mixture was stirred at room temperature for 24 h and quenched with acetic acid (30 mL) and water (90 mL). The organic layer was separated, and the aqueous layer was extracted with ethyl acetate (30 mL×3). The combined organic layer was washed with brine (50 mL), dried over Na_2SO_4, then filtered and evaporated. The residue was recrystallization from ethyl acetate and hexane to afford the white crystal **S2a** (3.61 g, yield 67%), colorless oil *N*-Boc-**S2a** (4.14 g, yield 56%) or white crystal **S2b** (3.50 g, yield 65%).

To a suspension of **S2a** or **S2b** (2.69 g, 10 mmol) in THF (20 mL) was added potassium diphenylphosphide solution (0.5 M in THF, 30 mL, 15 mmol) at 0 °C. The mixture was stirred at room temperature for 2 h and quenched with sat. $NaHCO_3$ (40 mL). The organic layer was separated, and the aqueous layer was extracted with ethyl acetate (40 mL×3). The combined organic layer was washed with brine (50 mL), dried over Na_2SO_4, then filtered and evaporated. The residue was subjected to column chromatography on silica gel using hexane/ethyl acetate 2:1 as an eluent to afford the white solid **S3a** (2.26 g, yield 80%), colorless oil *N*-Boc-**S3a** (2.88 g, yield 75%) or white solid **S3b** (2.30 g, yield 81%).

To a solution of **S3a** or **S3b** (2.69 g, 5 mmol) in CH_3OH (10 mL), H_2O_2 (1.7 mL, 30% in water, 15 mmol) was dropped slowly and stirred at room temperature. The resulting mixture was continued to stir for 30 minutes. After checking the complete conversion of the phosphine by TLC, the reaction mixture was quenched by sat. $NaHCO_3$ solution (20 mL) and sat. $Na_2S_2O_3$ solution (20 mL) and stirred for a few minutes additionally. The reaction mixture was separated, and the water layer was extracted with DCM (30 mL×3). The combined organic layer was dried over Na_2SO_4 and volatiles were removed in vacuo. The residue was subjected to column chromatography on silica gel using DCM/CH_3OH 9:1 as an eluent to afford the white solid **3a**, colorless oil **3g** or white

solid **3f**.

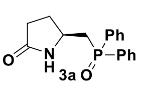

Catalyst 3a: Purified by flash chromatography on silica gel, eluting with methanol/dichloromethane 10% (v/v); White solid (1.30 g, 87% yield); m.p. 184-185 °C; $[\alpha]_D^{20}$ = 71.4 (c = 0.5 in DCM); ^{1}H NMR (500 MHz, $CDCl_3$) δ 7.70-7.78 (m, 4H), 7.47-7.59 (m, 6H), 6.81 (s, 1H), 3.89-3.94 (m, 1H), 2.44-2.55 (m, 2H), 2.22-2.34 (m, 3H), 1.80-1.86 (m, 1H); ^{13}C NMR (125 MHz, $CDCl_3$) δ 176.9, 132.7 (d, J = 100.0 Hz), 132.3(3) (d, J = 3.8 Hz), 132.2(5) (d, J = 2.5 Hz), 131.6 (d, J = 98.8 Hz), 130.9 (d, J = 10.0 Hz), 130.5 (d, J = 10.0 Hz), 129.0 (d, J = 1.3 Hz), 128.9 (d, J = 2.5 Hz), 49.5 (d, J = 5.0 Hz), 36.9 (d, J = 70.0 Hz), 29.8, 29.7 (d, J = 12.5 Hz); ^{31}P NMR (203 MHz, $CDCl_3$) δ 30.5; HRMS (FTMS-ESI) m/z: $[M+H]^+$ calcd for $C_{17}H_{19}NO_2P^+$ 300.1148, found 300.1126.

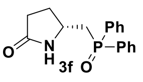

Catalyst 3f: Purified by flash chromatography on silica gel, eluting with methanol/dichloromethane 10% (v/v); White solid (1.29 g, 86% yield); m.p. 184-185 °C; $[\alpha]_D^{20}$ = -72.2 (c = 0.5 in DCM); ^{1}H NMR (500 MHz, $CDCl_3$) δ 7.70-7.78 (m, 4H), 7.47-7.59 (m, 6H), 6.83 (s, 1H), 3.88-3.94 (m, 1H), 2.46-2.55 (m, 2H), 2.21-2.34 (m, 3H), 1.79-1.86 (m, 1H); ^{13}C NMR (125 MHz, $CDCl_3$) δ 177.0, 132.7 (d, J = 98.8 Hz), 132.3(2) (d, J = 2.5 Hz), 132.2(5) (d, J = 2.5 Hz), 131.6 (d, J = 98.8 Hz), 130.9 (d, J = 8.8 Hz), 130.5 (d, J = 8.8 Hz), 129.0 (d, J = 1.3 Hz), 128.9, 49.5 (d, J = 3.8 Hz), 36.9 (d, J = 70.0 Hz), 29.8, 29.6 (d, J = 12.5 Hz); ^{31}P NMR (203 MHz, $CDCl_3$) δ 30.6; HRMS (FTMS-ESI) m/z: $[M+H]^+$ calcd for $C_{17}H_{19}NO_2P^+$ 300.1148, found 300.1132.

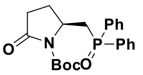

3g

Catalyst 3g: Purified by flash chromatography on silica gel, eluting with methanol/dichloromethane 10% (v/v); Colorless oil (1.71 g, 86% yield); $[\alpha]_D^{20}$ = 87.2 (c = 0.5 in DCM); ^{1}H NMR (500 MHz, $CDCl_3$) δ 7.92-8.00 (m, 4H), 7.69-7.81 (m, 6H), 4.11-4.16 (m, 1H), 2.66-2.77 (m, 2H), 2.44-2.56 (m, 3H), 2.02-2.08 (m, 1H), 1.35 (s, 9H); ^{13}C NMR (125 MHz, $CDCl_3$) δ 177.0, 156.6, 132.7 (d, J = 100.0 Hz), 132.4 (d, J = 3.8 Hz), 132.3 (d, J = 2.5 Hz), 131.6 (d, J = 98.8 Hz), 131.0 (d, J = 10.0 Hz), 130.5 (d, J = 10.0 Hz), 129.1 (d, J = 1.3 Hz), 129.0 (d, J = 2.5 Hz), 49.6 (d, J = 5.0 Hz), 36.9 (d, J = 70.0 Hz), 29.9, 29.7 (d, J = 12.5 Hz), 24.2, 23.6; ^{31}P NMR (203 MHz, $CDCl_3$) δ 29.3; HRMS (FTMS-ESI) m/z: $[M+H]^+$ calcd for $C_{22}H_{27}NO_4P^+$ 400.1672, found 400.1654.

2.2. Optimization studies

Figure S1. The effects of phosphine oxide[a]

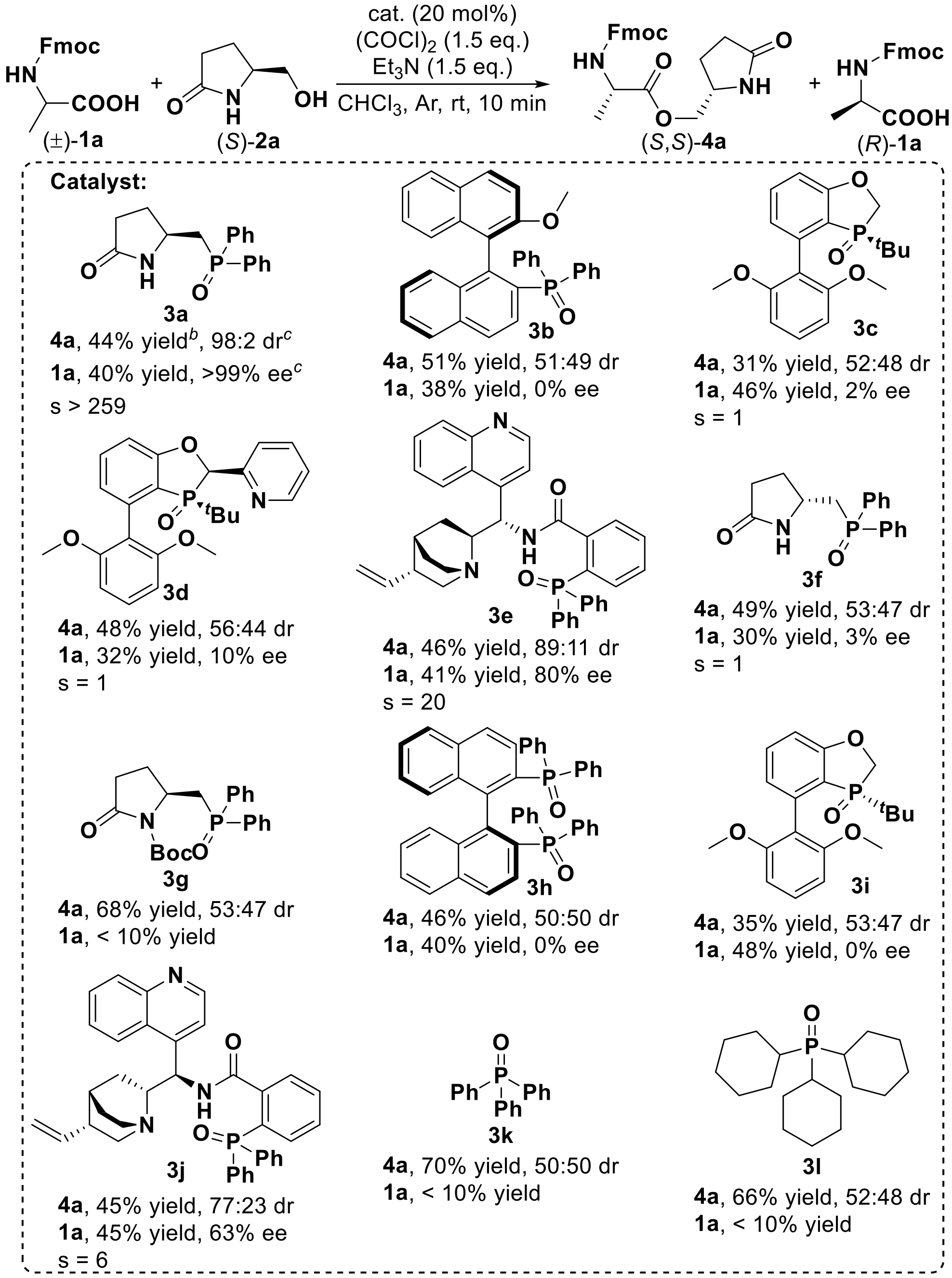

[a]Unless otherwise noted, all reactions were carried out using amino acid **1a** (0.5 mmol, 1.0 equiv.), *L*-pyroglutaminol **2a** (0.3 mmol, 0.6 equiv.) and catalyst in chloroform (1.0 mL) and oxalyl chloride (0.75 mmol, 1.5 equiv.) and triethylamine (0.75 mmol, 1.5 equiv.) were added in sequence at ambient temperature in argon. [b]Isolated yield. [c]Determined by HPLC on a chiral stationary phase.

Figure S2. The effects of alcohol[a]

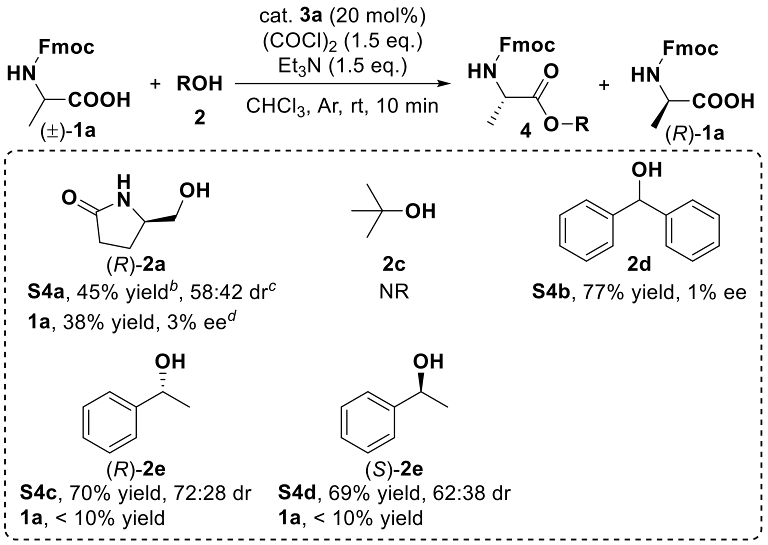

[a]Unless otherwise noted, all reactions were carried out using amino acid **1a** (0.5 mmol, 1.0 equiv.), alcohol **2** (0.3 mmol, 0.6 equiv.) and catalyst **3a** in chloroform (1.0 mL) and oxalyl chloride (0.75 mmol, 1.5 equiv.) and triethylamine (0.75 mmol, 1.5 equiv.) were added in sequence at ambient temperature in argon. [b]Isolated yield. [c]Determined by ^{1}H NMR. [d]Determined by HPLC on a chiral stationary phase.

Table S1. The effects of solvent[a]

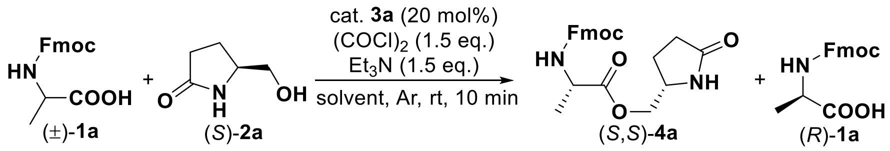

Entry	Solvent	**4a** Yield (%)[b]	**4a** dr[c]	**1a** Yield (%)[b]	**1a** ee (%)[c]	S
1	$CHCl_3$	44	98:2	40	>99	>259
2	PhMe	32	95:5	54	43	29
3	THF	35	93:7	49	51	22
4	MeCN	20	64:36	67	18	2
5	DCE	39	96:4	49	87	68

[a]Unless otherwise noted, all reactions were carried out using amino acid **1a** (0.5 mmol, 1.0 equiv.), *L*-pyroglutaminol **2a** (0.3 mmol, 0.6 equiv.) and catalyst **3a** in solvent (1.0 mL) and oxalyl chloride (0.75 mmol, 1.5 equiv.) and triethylamine (0.75 mmol, 1.5 equiv.) were added in sequence at ambient temperature in argon. [b]Isolated yield. [c]Determined by HPLC on a chiral stationary phase.

Table S2. The effects of catalyst equivalent[a]

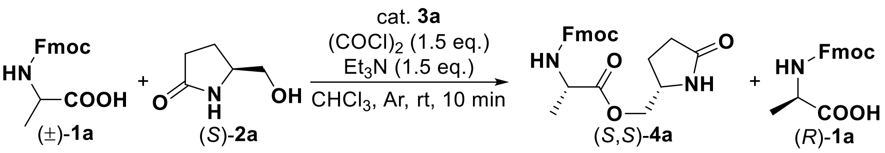

Entry	Catalyst equivalent	**4a** Yield (%)[b]	**4a** dr[c]	**1a** Yield (%)[b]	**1a** ee (%)[c]	S
1	20 mol%	44	98:2	40	>99	>259
2	10 mol%	39	97:3	42	92	107
3	Without catalyst	NR	-	-	-	-

[a]Unless otherwise noted, all reactions were carried out using amino acid **1a** (0.5 mmol, 1.0 equiv.), *L*-pyroglutaminol **2a** (0.3 mmol, 0.6 equiv.) and catalyst **3a** in chloroform (1.0 mL) and oxalyl chloride (0.75 mmol, 1.5 equiv.) and triethylamine (0.75 mmol, 1.5 equiv.) were added in sequence at ambient temperature in argon. [b]Isolated yield. [c]Determined by HPLC on a chiral stationary phase.

2.3. General procedure for the kinetic resolution of amino acids

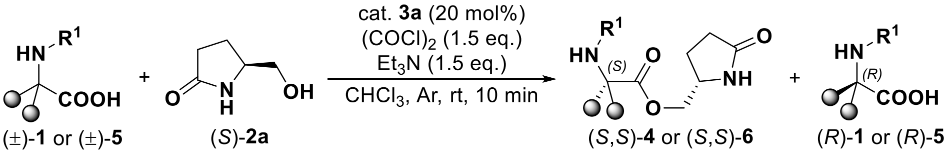

Racemic amino acids **1** or **5** (0.5 mmol, 1.0 equiv.), *L*-pyroglutaminol **2a** (0.3 mmol, 0.6 equiv.) and catalyst **3a** (0.1 mmol, 20 mol %) were well mixed in chloroform (1.0 mL). Then oxalyl chloride (0.75 mmol, 1.5 equiv.) and triethylamine (0.75 mmol, 1.5 equiv.) were added in sequence at ambient temperature under argon atmosphere. The resulting mixture was stirred at room temperature for 10 min. Subseqently, the mixture was partitioned between EtOAc (70 mL) and H_2O (30 mL) at room temperature. The organic layer was washed with saturated brine (30 mL×2), dried over Na_2SO_4, and concentrated *in vacuo*. The resulting residue was dissolved in dichloromethane (10 mL) and 1M aqueous solution NaOH (3 mL). The organic layer and the aqueous layer were separated. The organic layer was washed with saturated brine (10 mL×2), dried over Na_2SO_4, and concentrated *in vacuo*. The resulting residue was purified *via* silica gel colunm chromatography (MeOH/DCM = 5%-10%) to yield esters (*S*,*S*)-**4** or (*S*,*S*)-**6**. The aqueous layer was washed with dichloromethane (10 mL×2). The organic layer was discarded. The aqueous layer was made acidic with excess 1M aqueous solution HCl (to pH ~5) and was extracted with dichloromethane (10 mL×2). The combined

organic layer was washed with brine (20 mL×2), dried over Na_2SO_4, then filtered and evaporated to afford the recovered amino acids (*R*)-**1** or (*R*)-**5**.

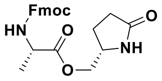
(*S*,*S*)-**4a**

Ester (*S*,*S*)-4a: Purified by flash chromatography on silica gel, eluting with MeOH/DCM 5%-10% (v/v); White solid (90.0 mg, 44% yield, 98:2 dr); m.p. 130-131 °C; $[\alpha]_D^{20}$ = 93.7 (c = 0.5 in DCM); ^{1}H NMR (500 MHz, $CDCl_3$) δ 8.00 (s, 1H), 7.75 (d, *J* = 7.5 Hz, 2H), 7.61 (d, *J* = 7.5 Hz, 2H), 7.38 (t, *J* = 7.5 Hz, 2H), 7.27-7.30 (m, 2H), 6.47 (d, *J* = 8.5 Hz, 1H), 4.43-4.50 (m, 2H), 4.32-4.35 (m, 1H), 4.18-4.24 (m, 2H), 3.92-4.00 (m, 2H), 2.29-2.40 (m, 2H), 2.15-2.22 (m, 1H), 1.71-1.78 (m, 1H), 1.44 (d, *J* = 7.0 Hz, 3H); ^{13}C NMR (125 MHz, $CDCl_3$) δ 179.0, 172.8, 156.3, 144.0, 143.8, 141.3, 127.7, 127.0(4), 126.9(9), 125.2, 120.0, 67.9, 66.9, 53.0, 49.9, 47.2, 30.0, 22.7, 18.5; HRMS (FTMS-ESI) m/z: $[M+Na]^+$ calcd for $C_{23}H_{24}N_2NaO_5^+$ 431.1577, found 431.1577; HPLC analysis (CHIRALCEL AD-H, *i*-propanol/*n*-hexane = 30/70, flow rate = 0.8 mL/min, wave length = 254 nm), t_R = 12.96 min (major), 14.88 min (minor).

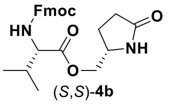
(*S*,*S*)-**4b**

Ester (*S*,*S*)-4b: Purified by flash chromatography on silica gel, eluting with MeOH/DCM 5%-10% (v/v); White solid (80.2 mg, 37% yield, 99:1 dr); m.p. 142-143 °C; $[\alpha]_D^{20}$ = 55.2 (c = 0.5 in DCM); ^{1}H NMR (500 MHz, $CDCl_3$) δ 7.95 (s, 1H), 7.75 (d, *J* = 7.5 Hz, 2H), 7.62 (t, *J* = 6.5 Hz, 2H), 7.38 (t, *J* = 7.5 Hz, 2H), 7.27-7.31 (m, 2H), 6.38 (d, *J* = 9.5 Hz, 1H), 4.35-4.46 (m, 3H), 4.22-4.27 (m, 2H), 3.88-3.92 (m, 2H), 2.32-2.35 (m, 2H), 2.16-2.25 (m, 2H), 1.70-1.77 (m, 1H), 0.98 (d, *J* = 6.5 Hz, 3H), 0.90 (d, *J* = 6.5 Hz, 3H); ^{13}C NMR (125 MHz, $CDCl_3$) δ 178.9, 171.7, 156.8, 144.1, 143.8, 141.4, 141.3, 127.7, 127.0(4), 126.9(8), 125.2, 125.1, 120.0, 67.8, 66.8, 59.4, 53.0, 47.3, 31.1, 29.9, 22.8, 19.2, 17.6; HRMS (FTMS-ESI) m/z: $[M+Na]^+$ calcd for $C_{25}H_{28}N_2NaO_5^+$ 459.1890, found 459.1863; HPLC analysis (CHIRALCEL AD-H, *i*-propanol/*n*-hexane = 30/70, flow rate = 0.8 mL/min, wave length = 254 nm), t_R = 13.15 min (major), 19.03 min (minor).

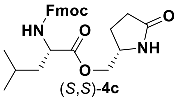
(*S*,*S*)-**4c**

Ester (*S*,*S*)-4c: Purified by flash chromatography on silica gel, eluting with MeOH/DCM 5%-10% (v/v); White solid (106.2 mg, 47% yield, 93:7 dr); m.p. 113-114 °C; $[\alpha]_D^{20}$ = 33.5 (c = 0.5 in DCM); ^{1}H NMR (500 MHz, $CDCl_3$) δ 8.09 (s, 1H), 7.75 (d, *J* = 7.5 Hz, 2H), 7.61 (dd, *J* = 7.5, 3.5 Hz, 2H), 7.37 (td, *J* = 7.5, 2.5 Hz, 2H), 7.27-7.30 (m, 2H), 6.43 (d, *J* = 9.0 Hz, 1H), 4.44-4.47 (m, 2H), 4.35-4.39 (m, 1H), 4.18-4.24 (m, 2H), 3.92-3.95 (m, 2H), 2.33 (t, *J* = 8.0 Hz, 2H), 2.14-2.21 (m, 1H), 1.64-1.75 (m, 3H), 1.55-1.61 (m, 1H), 0.95 (dd, *J* = 6.0, 4.0 Hz, 6H); ^{13}C NMR

(125 MHz, $CDCl_3$) δ 179.0, 172.8, 156.6, 144.1, 143.8, 141.4, 127.7, 127.0(2), 126.9(5), 125.1, 120.0, 67.9, 66.7, 53.1, 52.8, 47.3, 41.4, 30.0, 24.8, 23.0, 22.7, 21.7; HRMS (FTMS-ESI) m/z: $[M+H]^+$ calcd for $C_{26}H_{31}N_2O_5^+$ 451.2227, found 451.2233; HPLC analysis (CHIRALCEL OD-H, *i*-propanol/*n*-hexane = 30/70, flow rate = 0.8 mL/min, wave length = 254 nm), t_R = 21.17 min (major), 31.71 min (minor).

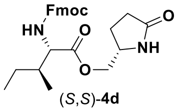

(*S*,*S*)-**4d**

Ester (*S*,*S*)-4d: Purified by flash chromatography on silica gel, eluting with MeOH/DCM 5%-10% (v/v); White solid (101.3 mg, 45% yield, 99:1 dr); m.p. 116-117 °C; $[\alpha]_D^{20}$ = 42.8 (c = 0.5 in DCM); ^{1}H NMR (500 MHz, $CDCl_3$) δ 8.27-8.35 (m, 1H), 7.74 (d, *J* = 7.5 Hz, 2H), 7.61 (t, *J* = 7.5 Hz, 2H), 7.37 (t, *J* = 7.5 Hz, 2H), 7.28 (t, *J* = 7.0 Hz, 2H), 6.56-6.61 (m, 1H), 4.41-4.47 (m, 2H), 4.37 (dd, *J* = 10.5, 7.0 Hz, 1H), 4.23 (dd, *J* = 13.0, 7.0 Hz, 2H), 3.85-3.91 (m, 2H), 2.30-2.34 (m, 2H), 2.13-2.17 (m, 1H), 1.97 (s, 1H), 1.67-1.74 (m, 1H), 1.37-1.44 (m, 1H), 1.14-1.23 (m, 1H), 0.95 (d, *J* = 7.0 Hz, 3H), 0.91 (t, *J* = 7.5 Hz, 3H), 0.84 (s, 1H); ^{13}C NMR (125 MHz, $CDCl_3$) δ 179.1, 171.6, 156.8, 144.1, 143.8, 141.4, 127.7, 127.0(4), 126.9(6), 125.2, 125.1, 120.0, 67.8, 66.7, 58.9, 53.1, 47.3, 37.9, 30.1, 25.1, 22.7, 15.8, 11.7; HRMS (FTMS-ESI) m/z: $[M+H]^+$ calcd for $C_{26}H_{31}N_2O_5^+$ 451.2227, found 451.2232; HPLC analysis (CHIRALCEL AD-H, *i*-propanol/*n*-hexane = 30/70, flow rate = 0.8 mL/min, wave length = 254 nm), t_R = 12.35 min (major), 18.39 min (minor).

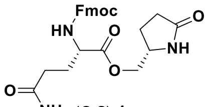

(*S*,*S*)-**4e**

Ester (*S*,*S*)-4e: Purified by flash chromatography on silica gel, eluting with MeOH/DCM 5%-10% (v/v); White solid (103.9 mg, 45% yield, >99:1 dr); m.p. 227-228 °C; $[\alpha]_D^{20}$ = 63.0 (c = 0.5 in DCM); ^{1}H NMR (500 MHz, DMSO-d_6) δ 7.90 (d, *J* = 7.0 Hz, 2H), 7.82 (d, *J* = 8.0 Hz, 1H), 7.74 (t, *J* = 7.5 Hz, 2H), 7.43 (t, *J* = 7.5 Hz, 2H), 7.34 (t, *J* = 7.5 Hz, 2H), 7.29 (s, 1H), 6.81 (s, 1H), 4.24-4.35 (m, 3H), 3.97-4.12 (m, 3H), 3.76 (s, 1H), 2.16-2.25 (m, 3H), 1.99-2.10 (m, 3H), 1.71-1.83 (m, 2H); ^{13}C NMR (125 MHz, DMSO-d_6) δ 177.4, 173.8, 172.6, 156.6, 144.3, 144.2, 141.2, 129.4, 128.1, 127.8, 127.6, 125.7(3), 125.7(1), 121.9, 120.6, 120.5, 67.5, 66.3, 54.0, 52.3, 47.1, 31.7, 29.9, 27.0, 23.2; HRMS (FTMS-ESI) m/z: $[M+H]^+$ calcd for $C_{25}H_{28}N_3O_6^+$ 466.1973, found 466.1961; HPLC analysis (CHIRALCEL OD-H, *i*-propanol/*n*-hexane = 30/70, flow rate = 0.8 mL/min, wave length = 254 nm), t_R = 11.43 min (major), 18.85 min (minor).

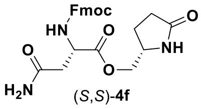

(*S*,*S*)-**4f**

Ester (*S*,*S*)-4f: Purified by flash chromatography on silica gel, eluting with MeOH/DCM 5%-10% (v/v); White solid (97.0 mg, 43% yield, >99:1 dr); m.p. 210-211 °C; $[\alpha]_D^{20}$ = 113.7 (c = 0.5 in DCM);

^{1}H NMR (500 MHz, $CDCl_3$) δ 8.99 (s, 1H), 7.76 (d, J = 7.5 Hz, 2H), 7.68 (d, J = 8.5 Hz, 1H), 7.63 (t, J = 8.5 Hz, 2H), 7.36-7.40 (m, 2H), 7.29 (t, J = 7.0 Hz, 2H), 4.75-4.77 (m, 1H), 4.41-4.49 (m, 2H), 4.24 (t, J = 7.0 Hz, 1H), 4.11-4.18 (m, 2H), 4.01 (s, 1H), 3.02 (dd, J = 16.5, 5.0 Hz, 1H), 2.92 (dd, J = 17.0, 5.0 Hz, 1H), 2.43-2.49 (m, 1H), 2.28-2.35 (m, 1H), 2.17-2.22 (m, 1H), 1.66-1.73 (m, 1H); ^{13}C NMR (125 MHz, $CDCl_3$) δ 179.9, 168.7, 156.4, 143.8, 143.6, 141.4, 127.8, 127.1, 127.0, 125.2, 125.1, 120.1, 116.6, 69.3, 67.1, 53.0, 50.9, 47.2, 30.1, 22.2, 21.7; HRMS (FTMS-ESI) m/z: $[M+H]^+$ calcd for $C_{24}H_{26}N_3O_6^+$ 452.1816, found 452.1800; HPLC analysis (CHIRALCEL OD-H, *i*-propanol/*n*-hexane = 30/70, flow rate = 0.8 mL/min, wave length = 254 nm), t_R = 23.98 min (major), 38.74 min (minor).

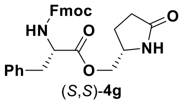

Ester (*S*,*S*)-4g: Purified by flash chromatography on silica gel, eluting with MeOH/DCM 5%-10% (v/v); White solid (114.2 mg, 47% yield, >99:1 dr); m.p. 183-184 °C; $[\alpha]_D^{20}$ = 77.9 (c = 0.5 in DCM); ^{1}H NMR (500 MHz, DMSO-d_6) δ 7.87-7.89 (m, 3H), 7.78 (s, 1H), 7.65 (t, J = 7.5 Hz, 2H), 7.41 (t, J = 7.5 Hz, 2H), 7.26-7.34 (m, 6H), 7.22 (t, J = 7.0 Hz, 1H), 4.31-4.35 (m, 1H), 4.23-4.28 (m, 2H), 4.17-4.21 (m, 1H), 4.02 (dd, J = 11.0, 4.5 Hz, 1H), 3.97 (dd, J = 11.0, 6.0 Hz, 1H), 3.70-3.75 (m, 1H), 3.11 (dd, J = 13.5, 4.5 Hz, 1H), 2.90 (dd, J = 13.5, 10.5 Hz, 1H), 2.17-2.23 (m, 1H), 2.05-2.12 (m, 2H), 1.65-1.72 (m, 1H); ^{13}C NMR (125 MHz, DMSO-d_6) δ 177.4, 172.1, 156.4, 144.2(1), 144.1(9), 141.2, 138.0, 129.6, 128.7, 128.1, 127.5, 127.0, 125.6(8), 125.6(6), 120.6, 67.7, 66.2, 55.9, 52.3, 47.0, 36.9, 29.9, 23.1; HRMS (FTMS-ESI) m/z: $[M+Na]^+$ calcd for $C_{29}H_{28}N_2NaO_5^+$ 507.1890, found 507.1884; HPLC analysis (CHIRALCEL AD-H, *i*-propanol/*n*-hexane = 30/70, flow rate = 0.8 mL/min, wave length = 254 nm), t_R = 10.62 min (major), 14.18 min (minor).

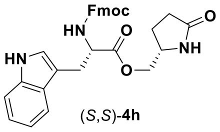

Ester (*S*,*S*)-4h: Purified by flash chromatography on silica gel, eluting with MeOH/DCM 5%-10% (v/v); White solid (111.7 mg, 43% yield, 94:6 dr); m.p. 240-241 °C; $[\alpha]_D^{20}$ = 91.1 (c = 0.5 in DCM); ^{1}H NMR (500 MHz, $CDCl_3$) δ 8.42 (s, 1H), 7.76 (d, J = 7.5 Hz, 2H), 7.55-7.58 (m, 3H), 7.37-7.40 (m, 3H), 7.28 (t, J = 7.5 Hz, 2H), 7.20 (t, J = 7.5 Hz, 1H), 7.13 (t, J = 7.5 Hz, 1H), 7.00 (s, 1H), 6.26 (s, 1H), 5.81 (d, J = 8.5 Hz, 1H), 4.72-4.76 (m, 1H), 4.37-4.43 (m, 2H), 4.20 (t, J = 7.0 Hz, 1H), 4.02 (dd, J = 11.0, 3.0 Hz, 1H), 3.83-3.87 (m, 1H), 3.58-3.60 (m, 1H), 3.34 (dd, J = 14.5, 5.5 Hz, 1H), 3.27 (dd, J = 14.5, 6.5 Hz, 1H), 2.00-2.20 (m, 3H), 1.51-1.58 (m, 1H); ^{13}C NMR (125 MHz, $CDCl_3$) δ 178.1, 172.1, 156.1, 143.9, 143.7, 141.3, 136.1, 127.7, 127.6, 127.1, 125.1, 123.0, 122.3, 120.0, 119.8,

118.6, 111.5, 109.9, 67.8, 67.0, 55.1, 52.4, 47.2, 29.4, 28.3, 22.6; HRMS (FTMS-ESI) m/z: $[M+H]^+$ calcd for $C_{31}H_{30}N_3NaO_5^+$ 524.2180, found 524.2166; HPLC analysis (CHIRALCEL AD-H, *i*-propanol/*n*-hexane = 30/70, flow rate = 0.8 mL/min, wave length = 254 nm), t_R = 13.51 min (major), 17.22 min (minor).

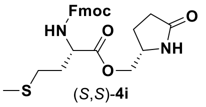

(*S*,*S*)-4i

Ester (*S*,*S*)-4i: Purified by flash chromatography on silica gel, eluting with MeOH/DCM 5%-10% (v/v); White solid (93.8 mg, 40% yield, 97:3 dr); m.p. 123-124 °C; $[\alpha]_D^{20}$ = 46.7 (c = 0.5 in DCM); ^{1}H NMR (500 MHz, $CDCl_3$) δ 8.37 (s, 1H), 7.75 (d, *J* = 7.5 Hz, 2H), 7.61 (t, *J* = 7.5 Hz, 2H), 7.35-7.39 (m, 2H), 7.29 (t, *J* = 7.5 Hz, 2H), 6.80 (d, *J* = 8.5 Hz, 1H), 4.56-4.60 (m, 1H), 4.45-4.49 (m, 1H), 4.39 (dd, *J* = 10.5, 7.5 Hz, 1H), 4.23 (t, *J* = 7.0 Hz, 1H), 4.16-4.18 (m, 1H), 3.92-3.99 (m, 2H), 2.54 (t, *J* = 7.5 Hz, 2H), 2.30-2.33 (m, 2H), 2.15-2.22 (m, 2H), 2.09 (s, 3H), 1.95-2.02 (m, 1H), 1.68-1.75 (m, 1H); ^{13}C NMR (125 MHz, $CDCl_3$) δ 179.2, 171.7, 156.6, 144.0, 143.8, 141.4, 127.7, 127.0(3), 126.9(5), 125.1(4), 125.1(0), 120.0(1), 119.9(9), 68.2, 66.7, 53.3, 53.1, 47.3, 31.9, 30.1, 22.6, 15.5; HRMS (FTMS-ESI) m/z: $[M+H]^+$ calcd for $C_{25}H_{29}N_2O_5S^+$ 469.1792, found 469.1790; HPLC analysis (CHIRALCEL OD-H, *i*-propanol/*n*-hexane = 30/70, flow rate = 0.8 mL/min, wave length = 254 nm), t_R = 11.52 min (major), 17.28 min (minor).

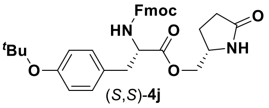

(*S*,*S*)-4j

Ester (*S*,*S*)-4j: Purified by flash chromatography on silica gel, eluting with MeOH/DCM 5%-10% (v/v); White solid (135.4 mg, 49% yield, >99:1 dr); m.p. 193-194 °C; $[\alpha]_D^{20}$ = 33.9 (c = 0.5 in DCM); ^{1}H NMR (500 MHz, $CDCl_3$) δ 7.88 (s, 1H), 7.74 (d, *J* = 7.5 Hz, 2H), 7.57 (d, *J* = 6.0 Hz, 2H), 7.37 (t, *J* = 7.0 Hz, 2H), 7.26-7.29 (m, 2H), 7.04 (d, *J* = 8.0 Hz, 2H), 6.89 (d, *J* = 8.5 Hz, 2H), 6.34 (d, *J* = 8.5 Hz, 1H), 4.66-4.71 (m, 1H), 4.36-4.39 (m, 2H), 4.18 (t, *J* = 7.0 Hz, 1H), 4.13 (dd, *J* = 11.0, 3.5 Hz, 1H), 3.96-4.00 (m, 1H), 3.85 (d, *J* = 4.5 Hz, 1H), 3.11 (dd, *J* = 14.0, 5.5 Hz, 1H), 3.04 (dd, *J* = 14.0, 7.5 Hz, 1H), 2.28 (t, *J* = 8.0 Hz, 2H), 2.11-2.15 (m, 1H), 1.65-1.73 (m, 1H), 1.30 (s, 9H); ^{13}C NMR (125 MHz, $CDCl_3$) δ 178.8, 171.5, 156.2, 154.4, 144.0, 143.8, 141.3, 131.0, 129.8, 127.7, 127.0, 125.2, 125.1, 124.2, 120.0, 78.5, 68.2, 66.8, 55.3, 52.9, 47.2, 37.6, 29.9, 28.8, 22.6; HRMS (FTMS-ESI) m/z: $[M+Na]^+$ calcd for $C_{33}H_{36}N_2NaO_6^+$ 579.2466, found 579.2465; HPLC analysis (CHIRALCEL AD-H, *i*-propanol/*n*-hexane = 30/70, flow rate = 0.8 mL/min, wave length = 254 nm), t_R = 15.33 min (major), 20.42 min (minor).

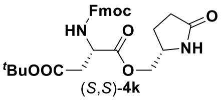

(*S*,*S*)-4k

Ester (*S*,*S*)-4k: Purified by flash chromatography on silica gel, eluting with MeOH/DCM 5%-10% (v/v); White solid (117.6 mg,

46% yield, 99:1 dr); m.p. 132-133 °C; $[\alpha]_D^{20}$ = 63.2 (c = 0.5 in DCM); ^{1}H NMR (500 MHz, $CDCl_3$) δ 7.75 (d, J = 7.5 Hz, 2H), 7.61 (d, J = 8.0 Hz, 3H), 7.39 (t, J = 7.5 Hz, 2H), 7.28-7.31 (m, 2H), 6.49 (d, J = 8.5 Hz, 1H), 4.68-4.72 (m, 1H), 4.44 (dd, J = 10.5, 7.5 Hz, 1H), 4.35 (dd, J = 10.5, 7.5 Hz, 1H), 4.24 (t, J = 7.0 Hz, 1H), 4.18 (dd, J = 10.5, 3.0 Hz, 1H), 4.02-4.06 (m, 1H), 3.95-3.96 (m, 1H), 2.91 (dd, J = 16.5, 5.0 Hz, 1H), 2.80 (dd, J = 16.5, 4.5 Hz, 1H), 2.29-2.40 (m, 2H), 2.16-2.23 (m, 1H), 1.68-1.75 (m, 1H), 1.45 (s, 9H); ^{13}C NMR (125 MHz, $CDCl_3$) δ 178.6, 170.8, 170.1, 156.3, 143.9, 143.8, 141.3, 127.7, 127.1, 127.0, 125.2, 120.0, 81.9, 68.5, 67.1, 52.8, 50.9, 47.2, 38.2, 29.7, 28.1, 22.7; HRMS (FTMS-ESI) m/z: $[M+Na]^+$ calcd for $C_{28}H_{32}N_2NaO_7^+$ 531.2102, found 531.2103; HPLC analysis (CHIRALCEL AD-H, *i*-propanol/*n*-hexane = 30/70, flow rate = 0.8 mL/min, wave length = 254 nm), t_R = 11.34 min (major), 16.49 min (minor).

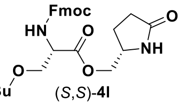
(S,S)-4l

Ester (*S,S*)-4l: Purified by flash chromatography on silica gel, eluting with MeOH/DCM 5%-10% (v/v); White solid (93.6 mg, 39% yield, >99:1 dr); m.p. 80-81 °C; $[\alpha]_D^{20}$ = 104.7 (c = 0.5 in DCM); ^{1}H NMR (500 MHz, $CDCl_3$) δ 7.76 (d, J = 7.5 Hz, 2H), 7.62-7.64 (m, 2H), 7.40 (t, J = 7.5 Hz, 2H), 7.31 (t, J = 7.5 Hz, 2H), 6.90 (s, 1H), 5.97 (d, J = 8.5 Hz, 1H), 4.51-4.53 (m, 1H), 4.37-4.46 (m, 2H), 4.24-4.27 (m, 2H), 3.94-3.99 (m, 2H), 3.85 (dd, J = 9.0, 3.0 Hz, 1H), 3.62 (dd, J = 9.0, 3.0 Hz, 1H), 2.30-2.41 (m, 2H), 2.19-2.26 (m, 1H), 1.72-1.79 (m, 1H), 1.17 (s, 9H); ^{13}C NMR (125 MHz, $CDCl_3$) δ 178.1, 170.5, 156.3, 144.0, 143.8, 141.3, 127.7, 127.0(8), 127.0(5), 125.2, 120.0, 73.8, 68.1, 67.1, 62.3, 54.9, 52.5, 47.2, 29.6, 27.4, 22.9; HRMS (FTMS-ESI) m/z: $[M+H]^+$ calcd for $C_{27}H_{33}N_2O_6^+$ 481.2333, found 481.2327; HPLC analysis (CHIRALCEL AD-H, *i*-propanol/*n*-hexane = 30/70, flow rate = 0.8 mL/min, wave length = 254 nm), t_R = 12.24 min (major), 17.38 min (minor).

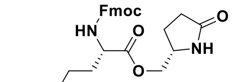
(S,S)-4m

Ester (*S,S*)-4m: Purified by flash chromatography on silica gel, eluting with MeOH/DCM 5%-10% (v/v); White solid (112.8 mg, 43% yield, 96:4 dr); m.p. 125-126 °C; $[\alpha]_D^{20}$ = 52.5 (c = 0.5 in DCM); ^{1}H NMR (500 MHz, $CDCl_3$) δ 8.03 (s, 1H), 7.75 (d, J = 7.5 Hz, 2H), 4.11 (dd, J = 7.0, 3.0 Hz, 2H), 7.37 (td, J = 7.5, 3.0 Hz, 2H), 7.29 (t, J = 7.0 Hz, 2H), 6.62 (d, J = 8.5 Hz, 1H), 4.42-4.48 (m, 2H), 4.33-4.36 (m, 1H), 4.22 (t, J = 7.0 Hz, 1H), 4.18 (dd, J = 10.5, 3.0 Hz, 1H), 3.92-4.00 (m, 2H), 2.28-2.40 (m, 4H), 2.15-2.21 (m, 2H), 1.95-2.02 (m, 2H), 1.69-1.76 (m, 1H), 1.45 (s, 9H); ^{13}C NMR (125 MHz, $CDCl_3$) δ 179.0, 172.1, 171.6, 156.6, 144.0, 143.8, 141.3, 127.7, 127.0(4), 126.9(7), 125.2, 120.0, 80.8, 68.1, 66.8, 53.8, 53.0,

47.2, 31.7, 29.9, 28.1, 27.5, 22.6; HRMS (FTMS-ESI) m/z: $[M+Na]^+$ calcd for $C_{29}H_{34}N_2NaO_7^+$ 545.2258, found 545.2232; HPLC analysis (CHIRALCEL AD-H, *i*-propanol/*n*-hexane = 30/70, flow rate = 0.8 mL/min, wave length = 254 nm), t_R = 13.40 min (major), 19.33 min (minor).

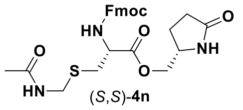
(*S*,*S*)-**4n**

Ester (*S*,*S*)-4n: Purified by flash chromatography on silica gel, eluting with MeOH/DCM 5%-10% (v/v); White solid (115.0 mg, 45% yield, >99:1 dr); m.p. 189-190 °C; $[\alpha]_D^{20}$ = 74.7 (c = 0.5 in DCM); ^{1}H NMR (500 MHz, DMSO-*d6*) δ 8.54 (t, *J* = 6.5 Hz, 1H), 7.90 (d, *J* = 8.0 Hz, 3H), 7.78 (s, 1H), 7.73 (d, *J* = 7.5 Hz, 2H), 7.43 (t, *J* = 7.5 Hz, 2H), 7.34 (t, *J* = 7.5 Hz, 2H), 4.30-4.40 (m, 4H), 4.19-4.27 (m, 2H), 4.06 (dd, *J* = 11.0, 4.5 Hz, 1H), 3.98 (dd, *J* = 11.0, 6.5 Hz, 1H), 3.74-3.78 (m, 1H), 3.04 (dd, *J* = 14.0, 4.5 Hz, 1H), 2.83 (dd, *J* = 14.0, 9.5 Hz, 1H), 2.18-2.27 (m, 1H), 2.07-2.13 (m, 2H), 1.87 (s, 3H), 1.70-1.76 (m, 1H); ^{13}C NMR (125 MHz, DMSO-*d6*) δ 177.4, 171.2, 170.1, 156.5, 144.2, 141.2, 128.1, 127.6, 125.7, 120.6, 67.9, 66.3, 54.5, 52.3, 47.1, 40.6, 32.0, 29.9, 23.0; HRMS (FTMS-ESI) m/z: $[M+H]^+$ calcd for $C_{26}H_{30}N_3O_6S^+$ 512.1850, found 512.1827; HPLC analysis (CHIRALCEL OD-H, *i*-propanol/*n*-hexane = 30/70, flow rate = 0.8 mL/min, wave length = 254 nm), t_R = 11.59 min (major), 16.45 min (minor).

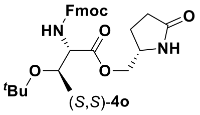
(*S*,*S*)-**4o**

Ester (*S*,*S*)-4o: Purified by flash chromatography on silica gel, eluting with MeOH/DCM 5%-10% (v/v); White solid (108.9 mg, 44% yield, 97:3 dr); m.p. 117-118 °C; $[\alpha]_D^{20}$ = 95.9 (c = 0.5 in DCM); ^{1}H NMR (500 MHz, $CDCl_3$) δ 7.79 (d, *J* = 7.5 Hz, 2H), 7.67 (d, *J* = 7.5 Hz, 2H), 7.41 (t, *J* = 7.5 Hz, 2H), 7.32-7.36 (m, 3H), 6.13 (d, *J* = 9.5 Hz, 1H), 4.47 (dd, *J* = 10.5, 7.0 Hz, 1H), 4.41 (dd, *J* = 10.5, 7.0 Hz, 1H), 4.34 (dd, *J* = 9.5, 2.0 Hz, 1H), 4.21-4.30 (m, 3H), 3.90-3.97 (m, 2H), 2.34-2.42 (m, 2H), 2.20-2.28 (m, 1H), 1.73-1.80 (m, 1H), 1.23 (d, *J* = 6.5 Hz, 3H), 1.17 (s, 9H); ^{13}C NMR (125 MHz, $CDCl_3$) δ 178.2, 171.0, 157.0, 144.1, 143.8, 141.3, 127.7, 127.1, 127.0, 125.2, 119.9(9), 119.9(8), 74.2, 68.3, 67.5, 67.2, 60.1, 52.6, 47.2, 29.7, 28.5, 22.9, 20.6; HRMS (FTMS-ESI) m/z: $[M+Na]^+$ calcd for $C_{28}H_{34}N_2NaO_6^+$ 517.2309, found 517.2321; HPLC analysis (CHIRALCEL AD-H, *i*-propanol/*n*-hexane = 30/70, flow rate = 0.8 mL/min, wave length = 254 nm), t_R = 10.66 min (major), 21.37 min (minor).

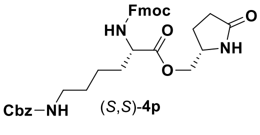
(*S*,*S*)-**4p**

Ester (*S*,*S*)-4p: Purified by flash chromatography on silica gel, eluting with MeOH/DCM 5%-10% (v/v); White solid (140.8 mg, 47% yield, >99:1 dr); m.p. 145-146 °C; $[\alpha]_D^{20}$ = 133.6 (c = 0.5 in

DCM); ^{1}H NMR (500 MHz, $CDCl_3$) δ 7.77 (d, J = 7.5 Hz, 2H), 7.63 (dd, J = 7.0, 3.0 Hz, 2H), 7.40 (t, J = 7.5 Hz, 2H), 7.35-7.36 (m, 4H), 7.29-7.33 (m, 3H), 6.35 (d, J = 0.5 Hz, 1H), 5.06-5.16 (m, 2H), 4.37-4.49 (m, 3H), 4.19-4.25 (m, 2H), 3.87-3.94 (m, 2H), 3.19-3.25 (m, 2H), 2.31-2.36 (m, 2H), 2.17-2.18 (m, 1H), 1.72-1.87 (m, 3H), 1.40-1.57 (m, 4H); ^{13}C NMR (125 MHz, $CDCl_3$) δ 179.1, 172.2, 156.6, 156.5, 144.0, 143.8, 141.3, 136.7, 128.6, 128.1, 127.7, 127.1, 127.0, 125.1, 120.0, 67.8, 66.8, 66.6, 54.0, 53.0, 47.2, 40.5, 32.0, 30.0, 29.3, 22.7, 22.4; HRMS (FTMS-ESI) m/z: $[M+Na]^+$ calcd for $C_{34}H_{37}N_3NaO_7^+$ 622.2524, found 622.2504; HPLC analysis (CHIRALCEL OD-H, *i*-propanol/*n*-hexane = 30/70, flow rate = 0.8 mL/min, wave length = 254 nm), t_R = 11.93 min (major), 13.65 min (minor).

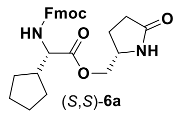

Ester (*S*,*S*)-6a: Purified by flash chromatography on silica gel, eluting with MeOH/DCM 5%-10% (v/v); White solid (91.7 mg, 40% yield, 99:1 dr); m.p. 93-94 °C; $[\alpha]_D^{20}$ = 76.7 (c = 0.5 in DCM); ^{1}H NMR (500 MHz, $CDCl_3$) δ 8.11 (s, 1H), 7.77 (d, J = 7.5 Hz, 2H), 7.63 (t, J = 7.0 Hz, 2H), 7.39-7.41 (m, 2H), 7.30-7.33 (m, 2H), 6.51 (d, J = 9.0 Hz, 1H), 4.45 (dd, J = 7.0, 3.0 Hz, 2H), 4.38-4.41 (m, 1H), 4.24-4.29 (m, 2H), 3.90-3.93 (m, 2H), 2.32-2.36 (m, 3H), 2.17-2.22 (m, 1H), 1.56-1.76 (m, 7H), 1.37-1.40 (m, 2H); ^{13}C NMR (125 MHz, $CDCl_3$) δ 179.0, 172.0, 156.7, 144.1, 143.8, 141.4, 141.3, 127.7, 127.0(3), 126.9(6), 125.2, 125.1, 119.9(9), 119.9(6), 67.7, 66.7, 57.5, 53.1, 47.3, 42.2, 30.0, 29.1, 28.3, 25.6, 25.3, 22.8; HRMS (FTMS-ESI) m/z: $[M+Na]^+$ calcd for $C_{27}H_{30}N_2NaO_5^+$ 485.2047, found 485.2034; HPLC analysis (CHIRALCEL OD-H, *i*-propanol/*n*-hexane = 30/70, flow rate = 0.8 mL/min, wave length = 254 nm), t_R = 22.08 min (major), 25.64 min (minor).

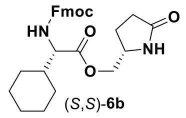

Ester (*S*,*S*)-6b: Purified by flash chromatography on silica gel, eluting with MeOH/DCM 5%-10% (v/v); White solid (92.8 mg, 39% yield, >99:1 dr); m.p. 90-91 °C; $[\alpha]_D^{20}$ = 43.4 (c = 0.5 in DCM); ^{1}H NMR (500 MHz, $CDCl_3$) δ 7.99 (s, 1H), 7.78 (d, J = 7.5 Hz, 2H), 7.64 (t, J = 7.5 Hz, 2H), 7.40 (t, J = 7.5 Hz, 2H), 7.29-7.33 (m, 2H), 6.39 (d, J = 9.5 Hz, 1H), 4.37-4.49 (m, 3H), 4.24-4.29 (m, 2H), 3.89-3.95 (m, 2H), 2.34-2.37 (m, 2H), 2.18-2.22 (m, 1H), 1.86-1.89 (m, 1H), 1.69-1.79 (m, 5H), 1.59 (d, J = 12.5 Hz, 1H), 1.22-1.31 (m, 2H), 1.06-1.16 (m, 3H); ^{13}C NMR (125 MHz, $CDCl_3$) δ 178.9, 171.7, 156.7, 144.1, 143.8, 141.4, 141.3, 127.7, 127.0(3), 126.9(7), 125.2, 125.1, 120.0(0), 119.9(6), 67.7, 66.7, 59.1, 53.1, 47.3, 40.8, 29.9, 29.6, 28.1, 26.0(1), 25.9(8), 25.9(5), 22.8; HRMS (FTMS-ESI) m/z: $[M+Na]^+$ calcd for $C_{28}H_{32}N_2NaO_5^+$ 499.2203, found 499.2206; HPLC analysis

(CHIRALCEL OD-H, *i*-propanol/*n*-hexane = 30/70, flow rate = 0.8 mL/min, wave length = 254 nm), t_R = 17.81 min (major), 27.37 min (minor).

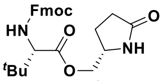

(*S,S*)-**6c**

Ester (*S,S*)-6c: Purified by flash chromatography on silica gel, eluting with MeOH/DCM 5%-10% (v/v); White solid (89.3 mg, 40% yield, >99:1 dr); m.p. 104-105 °C; $[\alpha]_D^{20}$ = 43.3 (c = 0.5 in DCM); ^{1}H NMR (500 MHz, $CDCl_3$) δ 7.78 (d, J = 7.5 Hz, 2H), 7.74 (s, 1H), 7.63 (t, J = 7.0 Hz, 2H), 7.41 (t, J = 7.5 Hz, 2H), 7.32 (td, J = 7.5, 3.0 Hz, 2H), 6.27 (d, J = 9.5 Hz, 1H), 4.44 (d, J = 7.0 Hz, 2H), 4.32 (d, J = 7.5 Hz, 1H), 4.24-4.26 (m, 2H), 3.87-3.91 (m, 2H), 2.34-2.39 (m, 2H), 2.19-2.22 (m, 1H), 1.76-1.79 (m, 1H), 1.03 (s, 9H); ^{13}C NMR (125 MHz, $CDCl_3$) δ 178.7, 171.0, 156.5, 144.0, 143.8, 141.4, 141.3, 127.7, 127.1, 127.0, 125.2, 125.1, 120.0, 67.5, 66.9, 62.6, 53.0, 47.3, 34.7, 29.9, 26.6, 22.9; HRMS (FTMS-ESI) m/z: $[M+H]^+$ calcd for $C_{26}H_{31}N_2O_5^+$ 451.2227, found 451.2232; HPLC analysis (CHIRALCEL AD-H, *i*-propanol/*n*-hexane = 30/70, flow rate = 0.8 mL/min, wave length = 254 nm), t_R = 12.29 min (major), 20.26 min (minor).

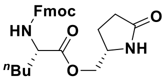

(*S,S*)-**6d**

Ester (*S,S*)-6d: Purified by flash chromatography on silica gel, eluting with MeOH/DCM 5%-10% (v/v); White solid (100.6 mg, 45% yield, 99:1 dr); m.p. 106-107 °C; $[\alpha]_D^{20}$ = 52.7 (c = 0.5 in DCM); ^{1}H NMR (500 MHz, $CDCl_3$) δ 8.16 (s, 1H), 7.78 (d, J = 8.0 Hz, 2H), 7.63-7.66 (m, 2H), 7.40 (t, J = 7.5 Hz, 2H), 7.31-7.33 (m, 2H), 6.53 (d, J = 9.0 Hz, 1H), 4.45-4.50 (m, 2H), 4.38-4.41 (m, 1H), 4.23-4.27 (m, 2H), 3.95-3.99 (m, 2H), 2.36 (t, J = 8.0 Hz, 2H), 2.18-2.22 (m, 1H), 1.87-1.91 (m, 1H), 1.69-1.76 (m, 2H), 1.37-1.39 (m, 4H), 0.93 (t, J = 6.5 Hz, 3H); ^{13}C NMR (125 MHz, $CDCl_3$) δ 179.0, 172.4, 156.6, 144.1, 143.8, 141.3, 127.7, 127.0(3), 126.9(6), 125.2, 120.0, 67.9, 66.8, 54.2, 53.1, 47.3, 32.2, 30.0, 27.6, 22.7, 22.3, 13.9; HRMS (FTMS-ESI) m/z: $[M+H]^+$ calcd for $C_{26}H_{31}N_2O_5^+$ 451.2227, found 451.2231; HPLC analysis (CHIRALCEL OD-H, *i*-propanol/*n*-hexane = 30/70, flow rate = 0.8 mL/min, wave length = 254 nm), t_R = 20.93 min (major), 30.08 min (minor).

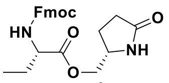

(*S,S*)-**6e**

Ester (*S,S*)-6e: Purified by flash chromatography on silica gel, eluting with MeOH/DCM 5%-10% (v/v); White solid (95.0 mg, 45% yield, >99:1 dr); m.p. 122-123 °C; $[\alpha]_D^{20}$ = 73.6 (c = 0.5 in DCM); ^{1}H NMR (500 MHz, $CDCl_3$) δ 8.08 (s, 1H), 7.78 (d, J = 7.5 Hz, 2H), 7.64 (dd, J = 7.0, 3.5 Hz, 2H), 7.41 (t, J = 7.5 Hz, 2H), 7.29-7.33 (m, 2H), 6.49 (d, J = 8.5 Hz, 1H), 4.38-4.49 (m, 3H), 4.23-4.27 (m, 2H), 3.96-3.99 (m, 2H), 2.35-2.38 (m, 2H), 2.17-2.26 (m, 1H), 1.91-1.96 (m, 1H), 1.73-1.79 (m, 2H), 0.99 (t, J = 7.5 Hz, 3H); ^{13}C NMR (125 MHz, $CDCl_3$) δ 179.0,

172.1, 156.5, 144.1, 143.8, 141.3, 127.7, 127.0(3), 126.9(7), 125.2, 120.0(0), 119.9(9), 67.9, 66.8, 55.4, 53.1, 47.3, 30.0, 25.8, 22.7, 9.8; HRMS (FTMS-ESI) m/z: $[M+H]^+$ calcd for $C_{24}H_{27}N_2O_5^+$ 423.1914, found 423.1910; HPLC analysis (CHIRALCEL AD-H, *i*-propanol/*n*-hexane = 30/70, flow rate = 0.8 mL/min, wave length = 254 nm), t_R = 14.75 min (major), 18.31 min (minor).

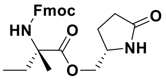

(*S*,*S*)-**6f**

Ester (*S*,*S*)-6f: Purified by flash chromatography on silica gel, eluting with MeOH/DCM 5%-10% (v/v); White solid (82.8 mg, 38% yield, >99:1 dr); m.p. 136-137 °C; $[\alpha]_D^{20}$ = 95.9 (c = 0.5 in DCM); ^{1}H NMR (500 MHz, $CDCl_3$) δ 7.78 (d, J = 7.5 Hz, 2H), 7.63 (d, J = 7.5 Hz, 2H), 7.42 (t, J = 7.5 Hz, 2H), 7.33 (t, J = 7.5 Hz, 2H), 6.69 (s, 1H), 5.53 (s, 1H), 4.45 (s, 2H), 4.24 (t, J = 6.5 Hz, 2H), 3.95 (s, 2H), 2.31-2.42 (m, 2H), 2.20-2.28 (m, 1H), 1.77-1.98 (m, 3H), 1.52-1.54 (m, 3H), 0.86 (s, 3H); ^{13}C NMR (125 MHz, $CDCl_3$) δ 178.0, 173.8, 155.2, 143.9, 143.8, 141.4, 127.7, 127.1, 125.1, 120.0, 68.0, 66.6, 60.2, 52.6, 47.2, 30.5, 29.6, 23.0, 22.5, 8.2; HRMS (FTMS-ESI) m/z: $[M+Na]^+$ calcd for $C_{25}H_{28}N_2NaO_5^+$ 459.1890, found 459.1900; HPLC analysis (CHIRALCEL AD-H, *i*-propanol/*n*-hexane = 30/70, flow rate = 0.8 mL/min, wave length = 254 nm), t_R = 8.44 min (major), 9.33 min (minor).

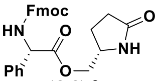

(*S*,*S*)-**6g**

Ester (*S*,*S*)-6g: Purified by flash chromatography on silica gel, eluting with MeOH/DCM 5%-10% (v/v); White solid (94.5 mg, 40% yield, >99:1 dr); m.p. 160-161 °C; $[\alpha]_D^{20}$ = 75.8 (c = 0.5 in DCM); ^{1}H NMR (500 MHz, $CDCl_3$) δ 7.78 (d, J = 7.5 Hz, 2H), 7.62 (d, J = 7.0 Hz, 2H), 7.52 (s, 1H), 7.36-7.44 (m, 7H), 7.30-7.32 (m, 2H), 6.78 (d, J = 7.5 Hz, 1H), 5.52 (d, J = 8.0 Hz, 1H), 4.47 (dd, J = 10.5, 7.0 Hz, 1H), 4.33-4.40 (m, 2H), 4.23-4.25 (m, 1H), 3.92-3.96 (m, 1H), 2.12-2.31 (m, 4H), 1.62-1.67 (m, 1H); ^{13}C NMR (125 MHz, $CDCl_3$) δ 178.7, 170.6, 155.9, 143.9, 143.8, 141.3, 136.5, 129.1, 128.7, 127.7, 127.3, 127.1, 127.0, 125.1, 120.0, 67.9, 67.1, 58.3, 53.0, 47.2, 29.6, 22.6; HRMS (FTMS-ESI) m/z: $[M+H]^+$ calcd for $C_{28}H_{27}N_2O_5^+$ 471.1914, found 471.1920; HPLC analysis (CHIRALCEL AD-H, *i*-propanol/*n*-hexane = 30/70, flow rate = 0.8 mL/min, wave length = 254 nm), t_R = 17.63 min (major), 20.44 min (minor).

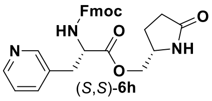

(*S*,*S*)-**6h**

Ester (*S*,*S*)-6h: Purified by flash chromatography on silica gel, eluting with MeOH/DCM 5%-10% (v/v); White solid (100.2 mg, 41% yield, >99:1 dr); m.p. 160-161 °C; $[\alpha]_D^{20}$ = 97.3 (c = 0.5 in DCM); ^{1}H NMR (500 MHz, DMSO-d_6) δ 8.50 (d, J = 1.0 Hz, 1H), 8.45 (d, J = 3.5 Hz, 1H), 7.93 (d, J = 8.5 Hz, 1H), 7.89 (d, J = 7.5 Hz, 2H), 7.81 (s, 1H), 7.68 (d, J = 8.0 Hz, 1H), 7.64 (t, J = 7.5 Hz, 2H),

7.42 (t, J = 7.5 Hz, 2H), 7.29-7.34 (m, 3H), 4.37-4.41 (m, 1H), 4.17-4.29 (m, 3H), 4.05 (dd, J = 11.0, 4.5 Hz, 1H), 4.00 (dd, J = 11.0, 6.5 Hz, 1H), 3.75 (s, 1H), 3.17 (dd, J = 14.0, 4.5 Hz, 1H), 2.93 (dd, J = 14.0, 11.0 Hz, 1H), 2.18-2.24 (m, 1H), 2.06-2.13 (m, 2H), 1.66-1.72 (m, 1H); ^{13}C NMR (125 MHz, DMSO-d_6) δ 177.4, 171.8, 156.4, 150.7, 148.2, 144.1(9), 144.1(5), 141.2, 137.2, 133.6, 128.1, 127.5, 125.6, 123.8, 120.6, 67.9, 66.2, 55.4, 52.3, 47.0, 34.0, 29.9, 23.0; HRMS (FTMS-ESI) m/z: [M+H]$^+$ calcd for $C_{28}H_{28}N_3O_5^+$ 486.2023, found 486.2022; HPLC analysis (CHIRALCEL AD-H, *i*-propanol/*n*-hexane = 30/70, flow rate = 0.8 mL/min, wave length = 254 nm), t_R = 10.62 min (major), 13.65 min (minor).

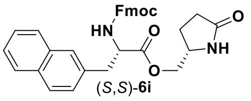

Ester (*S,S*)-6i: Purified by flash chromatography on silica gel, eluting with MeOH/DCM 5%-10% (v/v); White solid (112.1 mg, 42% yield, >99:1 dr); m.p. 197-198 °C; $[\alpha]_D^{20}$ = 143.6 (c = 0.5 in DCM); ^{1}H NMR (500 MHz, $CDCl_3$) δ 7.88 (s, 1H), 7.83-7.85 (m, 1H), 7.79-7.81 (m, 2H), 7.76 (d, J = 7.5 Hz, 2H), 7.65 (s, 1H), 7.55 (d, J = 6.5 Hz, 2H), 7.48-7.50 (m, 2H), 7.38 (t, J = 7.5 Hz, 2H), 7.34 (d, J = 8.0 Hz, 1H), 7.25 (t, J = 7.5 Hz, 2H), 6.50 (d, J = 9.0 Hz, 1H), 4.85-4.89 (m, 1H), 4.39 (d, J = 6.5 Hz, 2H), 4.19 (t, J = 6.5 Hz, 1H), 4.13 (dd, J = 10.5, 3.5 Hz, 1H), 3.90-3.94 (m, 1H), 3.78-3.79 (m, 1H), 3.29-3.38 (m, 2H), 2.15-2.22 (m, 2H), 2.02-2.08 (m, 1H), 1.49-1.57 (m, 1H); ^{13}C NMR (125 MHz, $CDCl_3$) δ 178.7, 171.5, 156.3, 143.9, 143.8, 141.3(2), 141.2(8), 133.8, 133.5, 132.5, 128.3, 128.1, 127.8, 127.7, 127.6, 127.3, 126.9(9), 126.9(6), 126.2, 125.8, 125.2, 125.1, 120.0, 68.2, 66.8, 55.2, 52.9, 47.2, 38.5, 29.8, 22.6; HRMS (FTMS-ESI) m/z: [M+Na]$^+$ calcd for $C_{33}H_{30}N_2NaO_5^+$ 557.2047, found 557.2061; HPLC analysis (CHIRALCEL AD-H, *i*-propanol/*n*-hexane = 30/70, flow rate = 0.8 mL/min, wave length = 254 nm), t_R = 25.22 min (major), 41.10 min (minor).

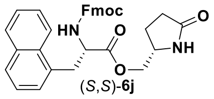

Ester (*S,S*)-6j: Purified by flash chromatography on silica gel, eluting with MeOH/DCM 5%-10% (v/v); White solid (123.9 mg, 46% yield, 91:9 dr); m.p. 213-214 °C; $[\alpha]_D^{20}$ = 95.3 (c = 0.5 in DCM); ^{1}H NMR (500 MHz, $CDCl_3$) δ 8.16 (d, J = 8.5 Hz, 1H), 7.90 (d, J = 8.0 Hz, 1H), 7.76-7.81 (m, 3H), 7.51-7.60 (m, 5H), 7.35-7.43 (m, 4H), 7.27-7.31 (m, 2H), 6.42 (d, J = 8.0 Hz, 1H), 4.90 (q, J = 7.5 Hz, 1H), 4.33-4.40 (m, 2H), 4.16-4.20 (m, 1H), 4.10 (dd, J = 11.0, 3.5 Hz, 1H), 3.91 (t, J = 10.0 Hz, 1H), 3.68-3.72 (m, 2H), 3.55 (dd, J = 14.0, 8.0 Hz, 1H), 2.23-2.27 (m, 2H), 2.04-2.11 (m, 1H), 1.54-1.61 (m, 1H); ^{13}C NMR (125 MHz, $CDCl_3$) δ 178.7, 171.7, 156.2, 143.9, 143.8, 141.3, 133.9, 132.6, 132.1, 129.0, 128.0, 127.7, 127.5, 127.0,

126.5, 125.9, 125.3, 125.2, 125.1, 123.5, 120.0, 68.1, 66.9, 55.1, 52.8, 47.2, 35.6, 29.7, 22.6; HRMS (FTMS-ESI) m/z: $[M+Na]^+$ calcd for $C_{33}H_{30}N_2NaO_5^+$ 557.2047, found 557.2039; HPLC analysis (CHIRALCEL AD-H, *i*-propanol/*n*-hexane = 30/70, flow rate = 0.8 mL/min, wave length = 254 nm), t_R = 20.27 min (major), 32.40 min (minor).

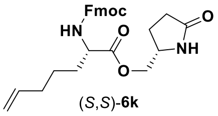

Ester (*S*,*S*)-6k: Purified by flash chromatography on silica gel, eluting with MeOH/DCM 5%-10% (v/v); White solid (100.9 mg, 44% yield, >99:1 dr); m.p. 100-101 °C; $[\alpha]_D^{20}$ = 71.9 (c = 0.5 in DCM); ^{1}H NMR (500 MHz, $CDCl_3$) δ 7.78 (d, J = 7.5 Hz, 2H), 7.63 (d, J = 7.0 Hz, 2H), 7.42 (t, J = 7.5 Hz, 2H), 7.33 (t, J = 7.5 Hz, 2H), 6.78 (s, 1H), 5.74-5.80 (m, 1H), 5.58 (s, 1H), 4.99-5.04 (m, 2H), 4.45 (s, 2H), 4.22-4.25 (m, 2H), 3.94 (s, 2H), 2.33-2.38 (m, 2H), 2.21-2.25 (m, 1H), 2.05 (s, 2H), 1.77-1.84 (m, 2H), 1.28-1.39 (m, 4H); ^{13}C NMR (125 MHz, $CDCl_3$) δ 178.0, 173.9, 155.2, 143.9, 143.8, 141.4, 137.9, 127.7, 127.1, 125.1, 120.0, 115.3, 68.1, 66.6, 59.7, 52.6, 47.2, 36.9, 33.4, 29.6, 23.1, 23.0; HRMS (FTMS-ESI) m/z: $[M+H]^+$ calcd for $C_{27}H_{31}N_2O_5^+$ 463.2227, found 463.2216; HPLC analysis (CHIRALCEL AD-H, *i*-propanol/*n*-hexane = 30/70, flow rate = 0.8 mL/min, wave length = 254 nm), t_R = 11.38 min (major), 12.65 min (minor).

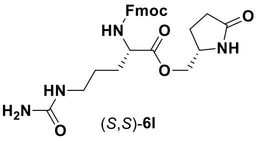

Ester (*S*,*S*)-6l: Purified by flash chromatography on silica gel, eluting with MeOH/DCM 5%-10% (v/v); White solid (108.4 mg, 44% yield, >99:1 dr); m.p. 273-274 °C; $[\alpha]_D^{20}$ = 44.6 (c = 0.5 in DCM); ^{1}H NMR (500 MHz, DMSO-d_6) δ 7.90 (d, J = 7.5 Hz, 2H), 7.82 (d, J = 8.0 Hz, 1H), 7.79 (s, 1H), 7.73 (d, J = 7.5 Hz, 2H), 7.43 (t, J = 7.5 Hz, 2H), 7.34 (t, J = 7.5 Hz, 2H), 5.95 (s, 1H), 5.42 (s, 2H), 4.23-4.35 (m, 3H), 3.95-4.12 (m, 3H), 3.75 (s, 1H), 2.96-2.98 (m, 2H), 2.18-2.26 (m, 1H), 2.05-2.13 (m, 2H), 1.70-1.74 (m, 2H), 1.57-1.65 (m, 1H), 1.39-1.46 (m, 2H); ^{13}C NMR (125 MHz, DMSO-d_6) δ 177.4, 172.7, 159.2, 156.6, 144.3, 144.2, 141.2, 128.1, 127.5, 125.7, 120.6, 67.5, 66.2, 54.2, 52.4, 47.1, 29.9, 28.7, 27.1, 23.1; HRMS (FTMS-ESI) m/z: $[M+H]^+$ calcd for $C_{26}H_{31}N_4O_6^+$ 495.2238, found 495.2260; HPLC analysis (CHIRALCEL AD-H, *i*-propanol/*n*-hexane = 30/70, flow rate = 0.8 mL/min, wave length = 254 nm), t_R = 16.48 min (major), 23.25 min (minor).

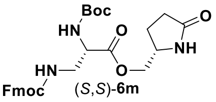

Ester (*S*,*S*)-6m: Purified by flash chromatography on silica gel, eluting with MeOH/DCM 5%-10% (v/v); White solid (104.6 mg, 40% yield, 98:2 dr); m.p. 70-71 °C; $[\alpha]_D^{20}$ = 43.1 (c = 0.5 in DCM); ^{1}H NMR (500 MHz, $CDCl_3$) δ 7.77 (d, J = 7.5 Hz, 2H), 7.58-7.60 (m, 3H), 7.41 (t, J = 7.5 Hz, 2H), 7.32 (t,

J = 7.5 Hz, 2H), 6.02 (d, J = 4.5 Hz, 1H), 5.87 (s, 1H), 4.38-4.40 (m, 3H), 4.32 (d, J = 9.5 Hz, 1H), 4.20 (t, J = 6.5 Hz, 1H), 3.89-3.95 (m, 2H), 3.58-3.70 (m, 2H), 2.28-2.38 (m, 2H), 2.18-2.22 (m, 1H), 1.73-1.75 (m, 1H), 1.47 (s, 9H); ^{13}C NMR (125 MHz, $CDCl_3$) δ 178.8, 170.6, 157.3, 155.7, 143.8, 141.3(0), 141.2(8), 127.8, 127.0(9), 127.0(7), 125.1, 120.0, 80.2, 68.4, 67.0, 54.5, 52.9, 47.2, 42.7, 29.8, 28.4, 22.8; HRMS (FTMS-ESI) m/z: $[M+Na]^+$ calcd for $C_{28}H_{33}N_3NaO_7^+$ 546.2211, found 546.2219; HPLC analysis (CHIRALCEL AD-H, *i*-propanol/*n*-hexane = 30/70, flow rate = 0.8 mL/min, wave length = 254 nm), t_R = 13.50 min (major), 18.61 min (minor).

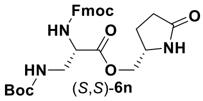

(*S*,*S*)-**6n**

Ester (*S*,*S*)-6n: Purified by flash chromatography on silica gel, eluting with MeOH/DCM 5%-10% (v/v); White solid (110.6 mg, 42% yield, >99:1 dr); m.p. 83-84 °C; $[\alpha]_D^{20}$ = 65.6 (c = 0.5 in DCM); ^{1}H NMR (500 MHz, $CDCl_3$) δ 7.78 (d, J = 7.5 Hz, 2H), 7.68 (s, 1H), 7.64 (d, J = 7.5 Hz, 2H), 7.41 (t, J = 7.5 Hz, 2H), 7.32 (t, J = 7.5 Hz, 2H), 6.56 (d, J = 5.5 Hz, 1H), 5.26 (s, 1H), 4.38-4.46 (m, 3H), 4.32 (d, J = 9.0 Hz, 1H), 4.25 (t, J = 7.0 Hz, 1H), 3.94-3.97 (m, 2H), 3.67-3.68 (m, 1H), 3.51-3.54 (m, 1H), 2.31-2.42 (m, 2H), 2.23-2.24 (m, 1H), 1.76-1.77 (m, 1H), 1.45 (s, 9H); ^{13}C NMR (125 MHz, $CDCl_3$) δ 178.8, 170.3, 156.6, 156.3, 144.0, 143.7, 141.4, 141.3, 127.7, 127.1, 125.2, 120.0(1), 119.9(9), 80.0, 68.4, 67.0, 55.1, 52.8, 47.2, 42.1, 29.8, 28.3, 22.8; HRMS (FTMS-ESI) m/z: $[M+Na]^+$ calcd for $C_{28}H_{33}N_3NaO_7^+$ 546.2211, found 546.2215; HPLC analysis (CHIRALCEL AD-H, *i*-propanol/*n*-hexane = 30/70, flow rate = 0.8 mL/min, wave length = 254 nm), t_R = 9.71 min (minor), 13.08 min (major).

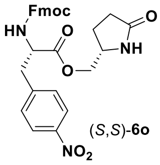

(*S*,*S*)-**6o**

Ester (*S*,*S*)-6o: Purified by flash chromatography on silica gel, eluting with MeOH/DCM 5%-10% (v/v); Yellowy solid (124.8 mg, 47% yield, >99:1 dr); m.p. 202-203 °C; $[\alpha]_D^{20}$ = 73.7 (c = 0.5 in DCM); ^{1}H NMR (500 MHz, DMSO-*d*$_6$) δ 8.15 (d, J = 8.5 Hz, 2H), 7.87-7.91 (m, 3H), 7.79 (s, 1H), 7.62 (dd, J = 7.5, 4.0 Hz, 2H), 7.54 (d, J = 8.5 Hz, 2H), 7.41 (t, J = 7.5 Hz, 2H), 7.28-7.32 (m, 2H), 4.42-4.47 (m, 1H), 4.23-4.32 (m, 2H), 4.18 (t, J = 6.5 Hz, 1H), 4.05 (dd, J = 11.0, 4.5 Hz, 1H), 4.00 (dd, J = 11.0, 6.0 Hz, 1H), 3.75-3.77 (m, 1H), 3.28 (dd, J = 13.5, 4.5 Hz, 1H), 3.04 (dd, J = 13.5, 11.0 Hz, 1H), 2.17-2.23 (m, 1H), 2.05-2.12 (m, 2H), 1.67-1.72 (m, 1H); ^{13}C NMR (125 MHz, DMSO-*d*$_6$) δ 177.4, 171.6, 156.4, 146.8, 146.4, 144.2, 144.1, 141.2(1), 141.1(9), 131.0, 128.1, 127.5, 125.6, 123.8, 120.6, 67.9, 66.1, 55.2, 52.3, 47.0, 36.5, 29.9, 23.0; HRMS (FTMS-ESI) m/z: $[M+Na]^+$ calcd for $C_{29}H_{27}N_3NaO_7^+$ 552.1741, found 552.1757; HPLC analysis

(CHIRALCEL AD-H, *i*-propanol/*n*-hexane = 30/70, flow rate = 0.8 mL/min, wave length = 254 nm), t_R = 23.83 min (minor), 37.94 min (major).

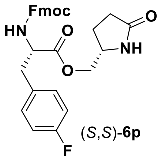
(*S*,*S*)-6p

Ester (*S*,*S*)-6p: Purified by flash chromatography on silica gel, eluting with MeOH/DCM 5%-10% (v/v); White solid (115.0 mg, 46% yield, 91:9 dr); m.p. 192-193 °C; $[\alpha]_D^{20}$ = 44.5 (c = 0.5 in DCM); ^{1}H NMR (500 MHz, DMSO-d_6) δ 7.86-7.90 (m, 3H), 7.80 (s, 1H), 7.65 (dd, *J* = 7.5, 3.0 Hz, 2H), 7.42 (t, *J* = 7.5 Hz, 2H), 7.29-7.34 (m, 4H), 7.11 (t, *J* = 9.0 Hz, 2H), 4.18-4.35 (m, 4H), 4.04 (dd, *J* = 11.0, 4.5 Hz, 1H), 3.98 (dd, *J* = 11.0, 6.5 Hz, 1H), 3.72-3.75 (m, 1H), 3.11 (dd, *J* = 14.0, 4.5 Hz, 1H), 2.89 (dd, *J* = 14.0, 10.5 Hz, 1H), 2.18-2.24 (m, 1H), 2.06-2.13 (m, 2H), 1.66-1.72 (m, 1H); ^{13}C NMR (125 MHz, DMSO-d_6) δ 177.4, 172.0, 162.5, 160.6, 156.4, 144.2(3), 144.1(6), 141.2, 134.1(7), 134.1(5), 131.5, 131.4, 128.1, 127.5, 125.6, 120.6, 115.5, 115.3, 67.8, 66.1, 55.9, 52.3, 47.1, 36.0, 29.9, 23.1; HRMS (FTMS-ESI) m/z: $[M+H]^+$ calcd for $C_{29}H_{28}FN_2O_5^+$ 503.1977, found 503.1969; HPLC analysis (CHIRALCEL OD-H, *i*-propanol/*n*-hexane = 30/70, flow rate = 0.8 mL/min, wave length = 254 nm), t_R = 14.71 min (major), 19.92 min (minor).

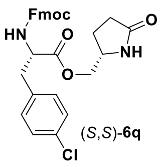
(*S*,*S*)-6q

Ester (*S*,*S*)-6q: Purified by flash chromatography on silica gel, eluting with MeOH/DCM 5%-10% (v/v); White solid (116.8 mg, 45% yield, >99:1 dr); m.p. 208-209 °C; $[\alpha]_D^{20}$ = 83.5 (c = 0.5 in DCM); ^{1}H NMR (500 MHz, DMSO-d_6) δ 7.86-7.90 (m, 3H), 7.80 (s, 1H), 7.64 (dd, *J* = 6.5, 5.5 Hz, 2H), 7.42 (t, *J* = 7.5 Hz, 2H), 7.32-7.35 (m, 3H), 7.28-7.31 (m, 3H), 4.18-4.36 (m, 4H), 4.03 (dd, *J* = 11.0, 4.5 Hz, 1H), 3.98 (dd, *J* = 11.0, 6.5 Hz, 1H), 3.72-3.75 (m, 1H), 3.12 (dd, *J* = 13.5, 4.5 Hz, 1H), 2.89 (dd, *J* = 13.5, 10.5 Hz, 1H), 2.17-2.24 (m, 1H), 2.06-2.12 (m, 2H), 1.65-1.70 (m, 1H); ^{13}C NMR (125 MHz, DMSO-d_6) δ 177.4, 171.9, 156.4, 144.2(3), 144.1(5), 141.2, 137.1, 131.7, 131.5, 128.6, 128.1, 127.5, 125.6, 120.6, 67.8, 66.1, 55.7, 52.3, 47.0, 36.1, 29.9, 23.1; HRMS (FTMS-ESI) m/z: $[M+H]^+$ calcd for $C_{29}H_{28}ClN_2O_5^+$ 519.1681, found 519.1685; HPLC analysis (CHIRALCEL AD-H, *i*-propanol/*n*-hexane = 30/70, flow rate = 0.8 mL/min, wave length = 254 nm), t_R = 19.39 min (major), 23.77 min (minor).

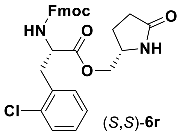
(*S*,*S*)-6r

Ester (*S*,*S*)-6r: Purified by flash chromatography on silica gel, eluting with MeOH/DCM 5%-10% (v/v); White solid (116.6 mg, 45% yield, 98:2 dr); m.p. 199-200 °C; $[\alpha]_D^{20}$ = 56.3 (c = 0.5 in DCM); ^{1}H NMR (500 MHz, DMSO-d_6) δ 7.94 (d, *J* = 8.5 Hz, 1H), 7.89 (d, *J* = 8.0 Hz, 2H), 7.74 (s, 1H), 7.65 (dd, *J* =11.0, 7.5 Hz, 2H), 7.40-7.44 (m, 3H), 7.36-7.38 (m, 1H), 7.26-7.34 (m,

4H), 4.42-4.46 (m, 1H), 4.16-4.29 (m, 3H), 4.00-4.06 (m, 2H), 3.73-3.76 (m, 1H), 3.31 (dd, J = 14.0, 5.0 Hz, 1H), 3.00 (dd, J = 14.0, 10.5 Hz, 1H), 2.18-2.24 (m, 1H), 2.06-2.12 (m, 2H), 1.69-1.74 (m, 1H); ^{13}C NMR (125 MHz, DMSO-d_6) δ 177.3, 171.9, 156.4, 144.2(0), 144.1(6), 141.2, 135.4, 133.8, 132.4, 129.8, 129.1, 128.1, 127.5, 125.6, 120.6, 67.7, 66.2, 53.9, 52.3, 47.0, 34.9, 29.9, 23.2; HRMS (FTMS-ESI) m/z: $[M+H]^+$ calcd for $C_{29}H_{27}ClN_2O_5^+$ 519.1681, found 519.1690; HPLC analysis (CHIRALCEL AD-H, *i*-propanol/*n*-hexane = 30/70, flow rate = 0.8 mL/min, wave length = 254 nm), t_R = 19.46 min (major), 32.18 min (minor).

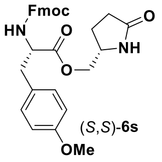

Ester (*S*,*S*)-6s: Purified by flash chromatography on silica gel, eluting with MeOH/DCM 5%-10% (v/v); White solid (102.8 mg, 40% yield, >99:1 dr); m.p. 170-171 °C; $[\alpha]_D^{20}$ = 59.1 (c = 0.5 in DCM); ^{1}H NMR (500 MHz, $CDCl_3$) δ 7.84 (s, 1H), 7.78 (d, J = 7.5 Hz, 2H), 7.59 (d, J = 7.5 Hz, 2H), 7.41 (t, J = 7.5 Hz, 2H), 7.29-7.32 (m, 2H), 7.09 (d, J = 8.0 Hz, 2H), 6.84 (d, J = 8.0 Hz, 2H), 6.32 (d, J = 8.5 Hz, 1H), 4.68-4.72 (m, 1H), 4.37-4.44 (m, 2H), 4.18-4.23 (m, 2H), 3.89-3.98 (m, 2H), 3.78 (s, 3H), 3.13 (dd, J = 14.0, 5.5 Hz, 1H), 3.05 (dd, J = 14.0, 7.0 Hz, 1H), 2.31-2.34 (m, 2H), 2.14-2.21 (m, 1H), 1.67-1.74 (m, 1H); ^{13}C NMR (125 MHz, $CDCl_3$) δ 178.8, 171.5, 158.7, 156.2, 144.0, 143.8, 141.3, 130.3, 128.1, 127.7, 127.0, 125.2, 125.1, 120.0, 114.0, 68.1, 66.8, 55.4, 55.3, 52.9, 47.2, 37.5, 29.9, 22.7; HRMS (FTMS-ESI) m/z: $[M+Na]^+$ calcd for $C_{30}H_{30}N_2NaO_6^+$ 537.1996, found 537.1999; HPLC analysis (CHIRALCEL AD-H, *i*-propanol/*n*-hexane = 30/70, flow rate = 0.8 mL/min, wave length = 254 nm), t_R = 30.97 min (major), 42.96 min (minor).

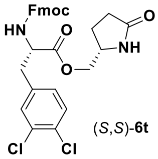

Ester (*S*,*S*)-6t: Purified by flash chromatography on silica gel, eluting with MeOH/DCM 5%-10% (v/v); White solid (113.4 mg, 41% yield, >99:1 dr); m.p. 255-256 °C; $[\alpha]_D^{20}$ = 103.2 (c = 0.5 in DCM); ^{1}H NMR (500 MHz, DMSO-d_6) δ 7.88-7.90 (m, 3H), 7.81 (s, 1H), 7.62 (dd, J = 7.5, 3.0 Hz, 2H), 7.58 (d, J = 2.0 Hz, 1H), 7.54 (d, J = 8.0 Hz, 1H), 7.41 (t, J = 7.5 Hz, 2H), 7.25-7.33 (m, 3H), 4.36-4.40 (m, 1H), 4.23-4.30 (m, 2H), 4.19 (t, J = 7.0 Hz, 1H), 4.05 (dd, J = 11.0, 5.0 Hz, 1H), 3.99 (dd, J = 11.0, 6.5 Hz, 1H), 3.75 (s, 1H), 3.15 (dd, J = 14.0, 4.5 Hz, 1H), 2.89 (dd, J = 14.0, 11.0 Hz, 1H), 2.18-2.24 (m, 1H), 2.06-2.13 (m, 2H), 1.66-1.72 (m, 1H); ^{13}C NMR (125 MHz, DMSO-d_6) δ 177.4, 171.7, 156.4, 144.2, 144.1, 141.2(0), 141.1(8), 139.3, 131.7, 131.2, 130.8, 130.1, 129.7, 128.1, 127.4(9), 127.4(7), 125.6, 120.6, 67.9, 66.2, 55.4, 52.3, 47.0, 35.7, 29.9, 23.0; HRMS (FTMS-ESI) m/z: $[M+Na]^+$ calcd for $C_{29}H_{26}Cl_2N_2NaO_5^+$ 575.1111, found 575.1102; HPLC

analysis (CHIRALCEL AD-H, *i*-propanol/*n*-hexane = 30/70, flow rate = 0.8 mL/min, wave length = 254 nm), t_R = 10.00 min (major), 16.78 min (minor).

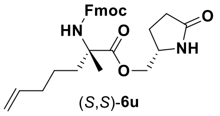

(*S,S*)-6u

Ester (*S,S*)-6u: Purified by flash chromatography on silica gel, eluting with MeOH/DCM 5%-10% (v/v); White solid (102.0 mg, 43% yield, 95:5 dr); m.p. 131-132 °C; $[\alpha]_D^{20}$ = 68.7 (c = 0.5 in DCM); ^{1}H NMR (500 MHz, $CDCl_3$) δ 7.79 (d, J = 7.5 Hz, 2H), 7.63 (d, J = 7.5 Hz, 2H), 7.42 (t, J = 7.5 Hz, 2H), 7.34 (t, J = 7.5 Hz, 2H), 6.62 (s, 1H), 5.74-5.80 (m, 1H), 5.51 (s, 1H), 4.99-5.05 (m, 2H), 4.44-4.45 (m, 2H), 4.23 (t, J = 6.5 Hz, 2H), 3.93-4.01 (m, 2H), 2.31-2.43 (m, 2H), 2.20-2.28 (m, 1H), 2.06 (s, 2H), 1.74-1.98 (m, 3H), 1.54 (s, 3H), 1.36-1.41 (m, 1H), 1.28-1.31 (m, 1H); ^{13}C NMR (125 MHz, $CDCl_3$) δ 177.9, 173.9, 155.1, 143.9, 143.8, 141.4, 137.9, 127.7(3), 127.7(2), 127.0(9), 127.0(8), 125.0, 120.0, 115.3, 68.0, 66.6, 59.7, 52.6, 47.2, 36.7, 33.4, 29.5, 23.1(3), 23.0(7), 23.0; HRMS (FTMS-ESI) m/z: $[M+Na]^+$ calcd for $C_{28}H_{32}N_2NaO_5^+$ 499.2203, found 499.2201; HPLC analysis (CHIRALCEL OD-H, *i*-propanol/*n*-hexane = 30/70, flow rate = 0.8 mL/min, wave length = 254 nm), t_R = 13.20 min (major), 19.13 min (minor).

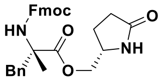

(*S,S*)-6v

Ester (*S,S*)-6v: Purified by flash chromatography on silica gel, eluting with MeOH/DCM 5%-10% (v/v); White solid (95.0 mg, 38% yield, 96:4 dr); m.p. 211-212 °C; $[\alpha]_D^{20}$ = 82.7 (c = 0.5 in DCM); ^{1}H NMR (500 MHz, $CDCl_3$) δ 7.80 (d, J = 7.5 Hz, 2H), 7.62-7.64 (m, 2H), 7.43 (t, J = 7.5 Hz, 2H), 7.34 (t, J = 7.5 Hz, 2H), 7.24-7.27 (m, 3H), 6.99-7.08 (m, 2H), 6.82 (d, J = 0.5 Hz, 1H), 5.40 (s, 1H), 4.45-4.55 (m, 2H), 4.26 (t, J = 6.5 Hz, 1H), 4.06-4.15 (m, 2H), 3.91 (d, J = 2.0 Hz, 1H), 3.25-3.32 (m, 2H), 2.29-2.41 (m, 2H), 2.19-2.26 (m, 1H), 1.74-1.75 (m, 1H), 1.52 (s, 3H); ^{13}C NMR (125 MHz, $CDCl_3$) δ 178.0, 173.4, 155.2, 143.8(2), 143.7(8), 141.4, 135.7, 130.3, 128.4, 127.8, 127.1, 125.1, 120.0, 68.2, 66.6, 60.1, 52.6, 47.3, 41.8, 29.6, 23.3, 23.0; HRMS (FTMS-ESI) m/z: $[M+H]^+$ calcd for $C_{30}H_{31}N_2O_5^+$ 499.2227, found 499.2216; HPLC analysis (CHIRALCEL AD-H, *i*-propanol/*n*-hexane = 30/70, flow rate = 0.8 mL/min, wave length = 254 nm), t_R = 11.40 min (major), 17.16 min (minor).

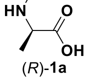

(*R*)-1a

Amino acid (*R*)-1a: White solid (62.5 mg, 40% yield, >99% ee); $[\alpha]_D^{20}$ = -14.9 (c = 0.5 in H_2O); ^{1}H NMR (500 MHz, DMSO-d_6) δ 12.58 (*br* s, 1H), 7.91 (d, J = 7.5 Hz, 2H), 7.75 (t, J = 6.5 Hz, 2H), 7.69 (d, J = 7.5 Hz, 1H), 7.44 (t, J = 7.5 Hz, 2H), 7.35 (t, J = 7.5 Hz, 2H), 4.28-4.32 (m, 2H), 4.23-4.26 (m, 1H), 4.00-4.06 (m, 1H), 1.31 (d, J = 7.5 Hz, 3H); HPLC analysis (CHIRALCEL AS-H, *i*-propanol/*n*-hexane = 10/90, flow rate = 0.8 mL/min, wave length = 254 nm), t_R = 19.91 min (minor),

27.45 min (major).

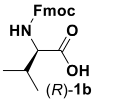
Amino acid (*R*)-1b: White solid (71.4 mg, 42% yield, 96% ee); ^{1}H NMR (500 MHz, DMSO-d_6) δ 12.62 (*br* s, 1H), 7.91 (d, *J* = 7.5 Hz, 2H), 7.78 (d, *J* = 7.0 Hz, 2H), 7.64 (d, *J* = 8.5 Hz, 1H), 7.44 (t, *J* = 7.25 Hz, 2H), 7.35 (td, *J* = 7.5, 2.0 Hz, 2H), 4.25-4.30 (m, 3H), 3.91 (dd, *J* = 8.5, 6.5 Hz, 1H), 2.07-2.13 (m, 1H), 0.94 (s, 3H), 0.93 (s, 3H); HPLC analysis (CHIRALCEL AD-H, *i*-propanol/*n*-hexane = 10/90, flow rate = 0.8 mL/min, wave length = 254 nm), t_R = 20.16 min (minor), 22.37 min (major).

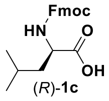
Amino acid (*R*)-1c: White solid (61.8 mg, 35% yield, 96% ee); ^{1}H NMR (500 MHz, DMSO-d_6) δ 12.57 (*br* s, 1H), 7.91 (d, *J* = 7.5 Hz, 2H), 7.75 (d, *J* = 7.5 Hz, 2H), 7.66 (d, *J* = 8.0 Hz, 1H), 7.44 (t, *J* = 7.5 Hz, 2H), 7.35 (td, *J* = 7.5, 2.0 Hz, 2H), 4.23-4.34 (m, 3H), 3.98-4.02 (m, 1H), 1.65-1.71 (m, 1H), 1.57-1.63 (m, 1H), 1.47-1.52 (m, 1H), 0.92 (d, *J* = 7.0 Hz, 3H), 0.87 (d, *J* = 6.5 Hz, 3H); HPLC analysis (CHIRALCEL AD-H, *i*-propanol/*n*-hexane = 10/90, flow rate = 0.8 mL/min, wave length = 254 nm), t_R = 15.42 min (major), 17.83 min (minor).

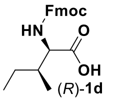
Amino acid (*R*)-1d: White solid (63.0 mg, 36% yield, 97% ee); ^{1}H NMR (500 MHz, DMSO-d_6) δ 12.65 (*br* s, 1H), 7.91 (d, *J* = 7.5 Hz, 2H), 7.78 (d, *J* = 7.5 Hz, 2H), 7.57 (d, *J* = 9.0 Hz, 1H), 7.44 (t, *J* = 7.5 Hz, 2H), 7.33-7.37 (m, 2H), 4.25-4.31 (m, 3H), 4.11-4.14 (m, 1H), 1.89-1.94 (m, 1H), 1.35-1.41 (m, 1H), 1.18-1.23 (m, 1H), 0.87-0.91 (m, 6H); HPLC analysis (CHIRALCEL AD-H, *i*-propanol/*n*-hexane = 10/90, flow rate = 0.8 mL/min, wave length = 254 nm), t_R = 18.78 min (minor), 21.94 min (major).

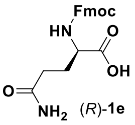
Amino acid (*R*)-1e: White solid (66.8 mg, 36% yield, 84% ee); ^{1}H NMR (500 MHz, DMSO-d_6) δ 12.68 (*br* s, 1H), 7.91 (d, *J* = 7.5 Hz, 2H), 7.76 (d, *J* = 7.5 Hz, 2H), 7.73 (d, *J* = 8.0 Hz, 1H), 7.44 (t, *J* = 7.5 Hz, 2H), 7.36 (t, *J* = 7.5 Hz, 3H), 6.84 (*br* s, 1H), 4.24-4.34 (m, 3H), 3.99-4.03 (m, 1H), 2.22 (t, *J* = 7.5 Hz, 2H), 2.00-2.07 (m, 1H), 1.79-1.86 (m, 1H); HPLC analysis (CHIRALCEL OJ-H, *i*-propanol/*n*-hexane = 30/70, flow rate = 0.8 mL/min, wave length = 254 nm), t_R = 32.98 min (minor), 37.34 min (major).

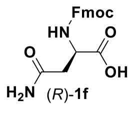
Amino acid (*R*)-1f: White solid (62.3 mg, 35% yield, >99% ee); ^{1}H NMR (500 MHz, DMSO-d_6) δ 12.77 (*br* s, 1H), 7.91 (d, *J* = 7.5 Hz, 2H), 7.74 (d, *J* = 7.5 Hz, 2H), 7.56 (d, *J* = 8.0 Hz, 1H), 7.44 (t, *J* = 7.5 Hz, 2H), 7.34-7.39 (m, 3H),

6.96 (*br* s, 1H), 4.35-4.39 (m, 1H), 4.23-4.30 (m, 3H), 2.60 (dd, *J* = 15.5, 5.5 Hz, 1H), 2.49 (dd, *J* = 15.5, 8.0 Hz, 1H); HPLC analysis (CHIRALCEL AD-H, *i*-propanol/*n*-hexane = 10/90, flow rate = 0.8 mL/min, wave length = 254 nm), t_R = 24.87 min (major), 30.19 min (minor).

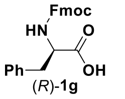

Amino acid (*R*)-1g: White solid (64.0 mg, 33% yield, >99% ee); ^{1}H NMR (500 MHz, DMSO-d_6) δ 12.80 (*br* s, 1H), 7.90 (d, *J* = 7.5 Hz, 2H), 7.77 (d, *J* = 8.5 Hz, 1H), 7.67 (t, *J* = 8.5 Hz, 2H), 7.41-7.45 (m, 2H), 7.29-7.35 (m, 6H), 7.21-7.25 (m, 1H), 4.17-4.23 (m, 4H), 3.11 (dd, *J* = 14.0, 4.0 Hz, 1H), 2.90 (dd, *J* = 13.5, 11.0 Hz, 1H); HPLC analysis (CHIRALCEL AD-H, *i*-propanol/*n*-hexane = 10/90, flow rate = 0.8 mL/min, wave length = 254 nm), t_R = 21.66 min (major), 24.63 min (minor).

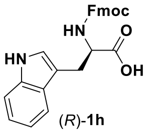

Amino acid (*R*)-1h: White solid (81.4 mg, 38% yield, 97% ee); ^{1}H NMR (500 MHz, DMSO-d_6) δ 12.75 (s, 1H), 10.90 (s, 1H), 7.90 (d, *J* = 7.5 Hz, 2H), 7.74 (d, *J* = 8.0 Hz, 1H), 7.69 (dd, *J* = 13.0, 7.5 Hz, 2H), 7.61 (d, *J* = 8.0 Hz, 1H), 7.38-7.45 (m, 3H), 7.29-7.36 (m, 2H), 7.23 (s, 1H), 7.11 (t, *J* = 7.5 Hz, 1H), 7.02 (t, *J* = 7.5 Hz, 1H), 4.27-4.31 (m, 1H), 4.19-4.25 (m, 3H), 3.25 (dd, *J* = 14.5, 4.0 Hz, 1H), 3.08 (dd, *J* = 14.5, 10.0 Hz, 1H); HPLC analysis (CHIRALCEL AS-H, *i*-propanol/*n*-hexane = 30/70, flow rate = 0.8 mL/min, wave length = 254 nm), t_R = 7.80 min (minor), 9.62 min (major).

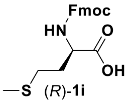

Amino acid (*R*)-1i: White solid (85.0 mg, 46% yield, 98% ee); ^{1}H NMR (500 MHz, DMSO-d_6) δ 12.71 (*br* s, 1H), 7.91 (d, *J* = 7.5 Hz, 2H), 7.75 (d, *J* = 6.5 Hz, 2H), 7.71 (d, *J* = 8.5 Hz, 1H), 7.44 (t, *J* = 7.5 Hz, 2H), 7.35 (t, *J* = 7.5 Hz, 2H), 4.32 (d, *J* = 7.0 Hz, 2H), 4.22-4.27 (m, 1H), 4.11-4.15 (m, 1H), 2.49-2.56 (m, 2H), 2.06 (s, 3H), 1.95-2.01 (m, 1H), 1.86-1.94 (m, 1H); HPLC analysis (CHIRALCEL OJ-H, *i*-propanol/*n*-hexane = 20/80, flow rate = 0.8 mL/min, wave length = 254 nm), t_R = 15.01 min (minor), 19.29 min (major).

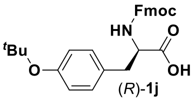

Amino acid (*R*)-1j: White solid (91.8 mg, 40% yield, >99% ee); ^{1}H NMR (500 MHz, DMSO-d_6) δ 12.78 (*br* s, 1H), 7.90 (d, *J* = 7.5 Hz, 2H), 7.76 (d, *J* = 8.5 Hz, 1H), 7.67-7.70 (m, 2H), 7.43 (t, *J* = 7.5 Hz, 2H), 7.30-7.35 (m, 2H), 7.19 (d, *J* = 8.0 Hz, 2H), 6.86 (d, *J* = 8.5 Hz, 2H), 4.15-4.22 (m, 4H), 3.06 (dd, *J* = 13.5, 4.0 Hz, 1H), 2.85 (dd, *J* = 13.5, 11.0 Hz, 1H), 1.23 (s, 9H); HPLC analysis (CHIRALCEL AD-H, *i*-propanol/*n*-hexane = 10/90, flow rate = 0.8 mL/min, wave length = 254 nm), t_R = 19.65 min (major), 20.35 min (minor).

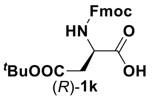

Amino acid (*R*)-1k: White solid (76.4 mg, 37% yield, >99% ee); ^{1}H NMR (500 MHz, DMSO-d_6) δ 12.81 (*br* s, 1H), 7.91 (d, J = 7.5 Hz, 2H), 7.72 (t, J = 6.0 Hz, 3H), 7.44 (t, J = 7.5 Hz, 2H), 7.34 (t, J = 7.5 Hz, 2H), 4.32-4.39 (m, 3H), 4.25 (t, J = 7.0 Hz, 1H), 2.72 (dd, J = 15.5, 5.5 Hz, 1H), 2.54-2.58 (m, 1H), 1.40 (s, 9H); HPLC analysis (CHIRALCEL AD-H, *i*-propanol/*n*-hexane = 10/90, flow rate = 0.8 mL/min, wave length = 254 nm), t_R = 19.57 min (minor), 22.07 min (major).

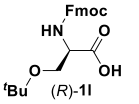

Amino acid (*R*)-1l: White solid (78.9 mg, 41% yield, >99% ee); ^{1}H NMR (500 MHz, DMSO-d_6) δ 12.79 (*br* s, 1H), 7.91 (d, J = 7.5 Hz, 2H), 7.77 (d, J = 7.0 Hz, 2H), 7.44 (t, J = 7.5 Hz, 3H), 7.35 (t, J = 7.5 Hz, 2H), 4.23-4.30 (m, 3H), 4.11-4.15 (m, 1H), 3.57-3.63 (m, 2H), 1.14 (s, 9H); HPLC analysis (CHIRALCEL OJ-H, *i*-propanol/*n*-hexane = 5/95, flow rate = 0.8 mL/min, wave length = 254 nm), t_R = 15.28 min (minor), 20.32 min (major).

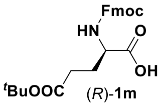

Amino acid (*R*)-1m: White solid (72.3 mg, 34% yield, >99% ee); ^{1}H NMR (500 MHz, DMSO-d_6) δ 12.69 (*br* s, 1H), 7.92 (d, J = 7.5 Hz, 2H), 7.75 (dd, J = 7.0, 3.0 Hz, 2H), 7.67 (d, J = 8.0 Hz, 1H), 7.44 (t, J = 7.5 Hz, 2H), 7.35 (t, J = 7.5 Hz, 2H), 4.23-4.32 (m, 3H), 3.99-4.03 (m, 1H), 2.28-2.33 (m, 2H), 1.96-2.01 (m, 1H), 1.75-1.83 (m, 1H), 1.41 (s, 9H); HPLC analysis (CHIRALCEL OJ-H, *i*-propanol/*n*-hexane = 5/95, flow rate = 0.8 mL/min, wave length = 254 nm), t_R = 42.48 min (major), 57.65 min (minor).

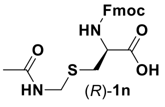

Amino acid (*R*)-1n: White solid (82.0 mg, 40% yield, 88% ee); ^{1}H NMR (500 MHz, DMSO-d_6) δ 12.86 (*br* s, 1H), 8.54 (t, J = 6.0 Hz, 1H), 7.91 (d, J = 7.5 Hz, 2H), 7.75-7.78 (m, 3H), 7.44 (t, J = 7.5 Hz, 2H), 7.35 (t, J = 7.5 Hz, 2H), 4.20-4.32 (m, 6H), 3.03 (dd, J = 13.5, 4.5 Hz, 1H), 2.83 (dd, J = 13.5, 10.0 Hz, 1H), 1.86 (s, 3H); HPLC analysis (CHIRALCEL AS-H, *i*-propanol/*n*-hexane = 30/70, flow rate = 0.8 mL/min, wave length = 254 nm), t_R = 8.02 min (minor), 10.87 min (major).

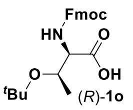

Amino acid (*R*)-1o: White solid (81.6 mg, 41% yield, 89% ee); ^{1}H NMR (500 MHz, DMSO-d_6) δ 12.65 (*br* s, 1H), 7.91 (d, J = 7.5 Hz, 2H), 7.79 (d, J = 7.0 Hz, 2H), 7.44 (t, J = 7.25 Hz, 2H), 7.35 (t, J = 7.25 Hz, 2H), 6.91 (d, J = 9.0 Hz, 1H), 4.24-4.34 (m, 3H), 4.09-4.12 (m, 1H), 4.03 (dd, J = 9.5, 2.5 Hz, 1H), 1.11-1.14 (m, 12H); HPLC analysis (CHIRALCEL AD-H, *i*-propanol/*n*-hexane = 10/90, flow rate = 0.8 mL/min, wave length = 254 nm), t_R = 10.54 min (minor), 14.80 min (major).

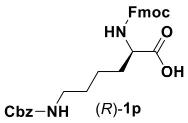

Amino acid (*R*)-1p: White solid (103.4 mg, 41% yield, 87% ee); ^{1}H NMR (500 MHz, DMSO-d_6) δ 12.60 (*br* s, 1H), 7.91 (d, J = 7.5 Hz, 2H), 7.75 (d, J = 7.5 Hz, 2H), 7.64 (d, J = 7.5 Hz, 1H), 7.44 (t, J = 7.5 Hz, 2H), 7.31-7.39 (m, 7H), 7.26-7.28 (m, 1H), 5.02 (s, 2H), 4.30 (d, J = 7.0 Hz, 2H), 4.24 (t, J = 7.0 Hz, 1H), 3.91-3.95 (m, 1H), 2.99-3.01 (m, 2H), 1.70-1.72 (m, 1H), 1.61-1.64 (m, 1H), 1.32-1.45 (m, 4H); HPLC analysis (CHIRALCEL OJ-H, *i*-propanol/*n*-hexane = 30/70, flow rate = 0.8 mL/min, wave length = 254 nm), t_R = 9.98 min (major), 15.86 min (minor).

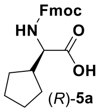

Amino acid (*R*)-5a: White solid (77.0 mg, 42% yield, 94% ee); ^{1}H NMR (500 MHz, DMSO-d_6) δ 12.55 (*br* s, 1H), 7.91 (d, J = 7.5 Hz, 2H), 7.76 (d, J = 7.5 Hz, 2H), 7.72 (d, J = 8.5 Hz, 1H), 7.44 (t, J = 7.5 Hz, 2H), 7.35 (td, J = 7.0, 3.0 Hz, 2H), 4.23-4.33 (m, 3H), 3.88 (t, J = 8.5 Hz, 1H), 2.18-2.23 (m, 1H), 1.50-1.74 (m, 6H), 1.30-1.37 (m, 2H); HPLC analysis (CHIRALCEL AD-H, *i*-propanol/*n*-hexane = 10/90, flow rate = 0.8 mL/min, wave length = 254 nm), t_R = 21.95 min (major), 24.26 min (minor).

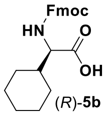

Amino acid (*R*)-5b: White solid (79.6 mg, 42% yield, 91% ee); ^{1}H NMR (500 MHz, DMSO-d_6) δ 12.60 (*br* s, 1H), 7.91 (d, J = 7.5 Hz, 2H), 7.78 (d, J = 7.5 Hz, 2H), 7.62 (d, J = 8.5 Hz, 1H), 7.44 (t, J = 7.5 Hz, 2H), 7.35 (td, J = 7.0, 2.5 Hz, 2H), 4.22-4.32 (m, 3H), 3.90 (t, J = 7.5 Hz, 1H), 1.71 (d, J = 11.5 Hz, 3H), 1.62 (d, J = 11.5 Hz, 3H), 1.05-1.24 (m, 5H); HPLC analysis (CHIRALCEL AD-H, *i*-propanol/*n*-hexane = 10/90, flow rate = 0.8 mL/min, wave length = 254 nm), t_R = 21.66 min (major), 25.50 min (minor).

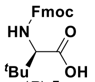

Amino acid (*R*)-5c: White solid (65.3 mg, 37% yield, 90% ee); ^{1}H NMR (500 MHz, DMSO-d_6) δ 12.62 (*br* s, 1H), 7.91 (d, J = 7.5 Hz, 2H), 7.80 (dd, J = 7.5, 3.5 Hz, 2H), 7.63 (d, J = 9.5 Hz, 1H), 7.44 (t, J = 7.5 Hz, 2H), 7.33-7.37 (m, 2H), 4.25-4.30 (m, 3H), 3.91 (d, J = 9.5 Hz, 1H), 1.01 (s, 9H); HPLC analysis (CHIRALCEL AD-H, *i*-propanol/*n*-hexane = 10/90, flow rate = 0.8 mL/min, wave length = 254 nm), t_R = 17.66 min (minor), 22.79 min (major).

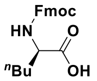

Amino acid (*R*)-5d: White solid (63.2 mg, 36% yield, >99% ee); ^{1}H NMR (500 MHz, DMSO-d_6) δ 12.64 (*br* s, 1H), 7.91 (d, J = 7.5 Hz, 2H), 7.75 (d, J = 7.0 Hz, 2H), 7.63 (d, J = 8.0 Hz, 1H), 7.44 (t, J = 7.5 Hz, 2H), 7.35 (t, J = 7.5 Hz, 2H), 4.29-4.33 (m, 2H), 4.23-4.27 (m, 1H), 3.92-3.96 (m, 1H), 1.71-1.74 (m, 1H), 1.61-

1.64 (m, 1H), 1.28-1.33 (m, 4H), 0.87-0.90 (s, 3H); HPLC analysis (CHIRALCEL OJ-H, *i*-propanol/*n*-hexane = 30/70, flow rate = 0.8 mL/min, wave length = 254 nm), t_R = 5.67 min (minor), 7.99 min (major).

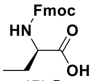

(*R*)-5e

Amino acid (*R*)-5e: White solid (63.5 mg, 39% yield, 86% ee); ^{1}H NMR (500 MHz, DMSO-d_6) δ 12.58 (*br* s, 1H), 7.91 (d, *J* = 7.5 Hz, 2H), 7.76 (dd, *J* = 7.0, 3.5 Hz, 2H), 7.65 (d, *J* = 8.0 Hz, 1H), 7.44 (t, *J* = 7.5 Hz, 2H), 7.35 (t, *J* = 7.5 Hz, 2H), 4.29-4.31 (m, 2H), 4.23-4.26 (m, 1H), 3.88-3.92 (m, 1H), 1.74-1.79 (m, 1H), 1.61-1.67 (m, 1H), 0.93 (t, *J* = 7.5 Hz, 3H); HPLC analysis (CHIRALCEL AD-H, *i*-propanol/*n*-hexane = 10/90, flow rate = 0.8 mL/min, wave length = 254 nm), t_R = 16.91 min (minor), 18.18 min (major).

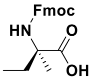

(*R*)-5f

Amino acid (*R*)-5f: White solid (76.6 mg, 45% yield, 90% ee); ^{1}H NMR (500 MHz, DMSO-d_6) δ 12.40 (*br* s, 1H), 7.91 (d, *J* = 7.5 Hz, 2H), 7.75 (d, *J* = 7.5 Hz, 2H), 7.44 (t, *J* = 7.5 Hz, 3H), 7.35 (t, *J* = 7.5 Hz, 2H), 4.22-4.29 (m, 3H), 1.82-1.86 (m, 1H), 1.71-1.75 (m, 1H), 1.34 (s, 3H), 0.81 (t, *J* = 7.0 Hz, 3H); HPLC analysis (CHIRALCEL AD-H, *i*-propanol/*n*-hexane = 5/95, flow rate = 1.0 mL/min, wave length = 254 nm), t_R = 30.05 min (minor), 32.57 min (major).

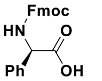

(*R*)-5g

Amino acid (*R*)-5g: White solid (70.9 mg, 38% yield, 95% ee); ^{1}H NMR (500 MHz, DMSO-d_6) δ 12.94 (*br* s, 1H), 8.27 (d, *J* = 8.0 Hz, 1H), 7.91 (d, *J* = 7.5 Hz, 2H), 7.79 (d, *J* = 7.5 Hz, 2H), 7.39-7.47 (m, 6H), 7.31-7.37 (m, 3H), 5.21 (d, *J* = 8.0 Hz, 1H), 4.23-4.34 (m, 3H); HPLC analysis (CHIRALCEL AD-H, *i*-propanol/*n*-hexane = 10/90, flow rate = 0.8 mL/min, wave length = 254 nm), t_R = 32.09 min (minor), 43.05 min (major).

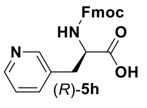

Amino acid (*R*)-5h: White solid (85.6 mg, 44% yield, 92% ee); ^{1}H NMR (500 MHz, DMSO-d_6) δ 12.90 (*br* s, 1H), 8.52 (d, *J* = 1.5 Hz, 1H), 8.44-8.45 (m, 1H), 7.90 (d, *J* = 7.5 Hz, 2H), 7.84 (d, *J* = 8.5 Hz, 1H), 7.71 (d, *J* = 8.0 Hz, 1H), 7.65 (t, *J* = 8.5 Hz, 2H), 7.41-7.44 (m, 2H), 7.30-7.35 (m, 3H), 4.17-4.26 (m, 4H), 3.15 (dd, *J* = 14.0, 4.5 Hz, 1H), 2.92 (dd, *J* = 14.0, 11.0 Hz, 1H); HPLC analysis (CHIRALCEL OJ-H, *i*-propanol/*n*-hexane = 30/70, flow rate = 0.8 mL/min, wave length = 254 nm), t_R = 31.40 min (major), 35.59 min (minor).

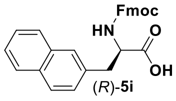

Amino acid (*R*)-5i: White solid (92.2 mg, 42% yield, 94% ee); ^{1}H NMR (500 MHz, DMSO-d_6) δ 12.84 (*br* s, 1H), 7.84-7.90 (m, 6H), 7.81

(s, 1H), 7.64 (d, J = 7.5 Hz, 1H), 7.61 (d, J = 7.5 Hz, 1H), 7.47-7.52 (m, 3H), 7.37-7.42 (m, 2H), 7.28 (t, J = 7.5 Hz, 1H), 7.20 (t, J = 7.5 Hz, 1H), 4.33-4.37 (m, 1H), 4.15-4.21 (m, 3H), 3.30 (dd, J = 13.5, 4.0 Hz, 1H), 3.09 (dd, J = 13.5, 11.0 Hz, 1H); HPLC analysis (CHIRALCEL AD-H, *i*-propanol/*n*-hexane = 10/90, flow rate = 0.8 mL/min, wave length = 254 nm), t_R = 30.34 min (major), 34.08 min (minor).

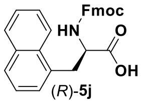

Amino acid (*R*)-5j: White solid (80.4 mg, 37% yield, 96% ee); ^{1}H NMR (500 MHz, DMSO-d_6) δ 12.95 (*br* s, 1H), 8.17 (d, J = 8.5 Hz, 1H), 7.96 (d, J = 8.5 Hz, 1H), 7.89-7.92 (m, 3H), 7.84 (d, J = 8.0 Hz, 1H), 7.67 (d, J = 7.5 Hz, 1H), 7.61-7.63 (m, 2H), 7.55 (t, J = 7.5 Hz, 1H), 7.50 (d, J = 7.0 Hz, 1H), 7.40-7.46 (m, 3H), 7.34 (t, J = 7.5 Hz, 1H), 7.28 (t, J = 7.5 Hz, 1H), 4.33-4.38 (m, 1H), 4.16-4.21 (m, 3H), 3.70 (dd, J = 14.0, 3.5 Hz, 1H), 3.30 (dd, J = 14.0, 11.0 Hz, 1H); HPLC analysis (CHIRALCEL AD-H, *i*-propanol/*n*-hexane = 10/90, flow rate = 0.8 mL/min, wave length = 254 nm), t_R = 26.83 min (major), 32.24 min (minor).

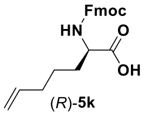

Amino acid (*R*)-5k: White solid (71.4 mg, 39% yield, 97% ee); ^{1}H NMR (500 MHz, DMSO-d_6) δ 12.42 (*br* s, 1H), 7.91 (d, J = 7.5 Hz, 2H), 7.74 (d, J = 7.5 Hz, 2H), 7.44 (t, J = 7.5 Hz, 3H), 7.35 (t, J = 7.5 Hz, 2H), 5.75-5.84 (m, 1H), 5.02 (d, J = 17.5 Hz, 1H), 4.97 (d, J = 10.0 Hz, 1H), 4.21-4.29 (m, 3H), 2.01-2.02 (m, 2H), 1.78-1.81 (m, 1H), 1.68-1.71 (m, 1H), 1.35 (s, 3H); HPLC analysis (CHIRALCEL AD-H, *i*-propanol/*n*-hexane = 10/90, flow rate = 0.8 mL/min, wave length = 254 nm), t_R = 10.08 min (minor), 13.20 min (major).

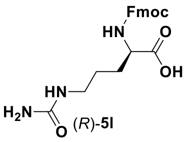

Amino acid (*R*)-5l: White solid (70.1 mg, 35% yield, >99% ee); ^{1}H NMR (500 MHz, DMSO-d_6) δ 12.63 (*br* s, 1H), 7.91 (d, J = 7.5 Hz, 2H), 7.76 (d, J = 7.5 Hz, 2H), 7.70 (d, J = 8.0 Hz, 1H), 7.44 (t, J = 7.5 Hz, 2H), 7.36 (t, J = 7.5 Hz, 2H), 5.97-5.99 (m, 1H), 5.42 (s, 2H), 4.29-4.31 (m, 2H), 4.23-4.26 (m, 1H), 3.94-3.98 (m, 1H), 2.96-3.00 (m, 2H), 1.71-1.76 (m, 1H), 1.57-1.63 (m, 1H), 1.40-1.49 (m, 2H); HPLC analysis (CHIRALCEL AD-H, *i*-propanol/*n*-hexane = 10/90, flow rate = 0.8 mL/min, wave length = 254 nm), t_R = 37.32 min (major), 53.29 min (minor).

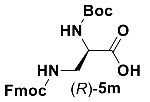

Amino acid (*R*)-5m: White solid (76.7 mg, 36% yield, >99% ee); ^{1}H NMR (500 MHz, DMSO-d_6) δ 7.91 (d, J = 7.5 Hz, 2H), 7.70 (d, J = 7.0 Hz, 2H), 7.44 (t, J = 7.5 Hz, 2H), 7.33-7.40 (m, 3H), 6.98 (d, J = 8.0 Hz, 1H), 4.27-4.33 (m, 2H), 4.22-4.24 (m, 1H), 4.05-4.09 (m, 1H), 3.33-3.38 (m, 2H), 1.40 (s, 9H);

HPLC analysis (CHIRALCEL AD-H, *i*-propanol/*n*-hexane = 10/90, flow rate = 0.8 mL/min, wave length = 254 nm), t_R = 19.85 min (major), 36.09 min (minor).

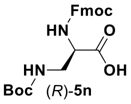

Amino acid (*R*)-5n: White solid (83.1 mg, 39% yield, 89% ee); ^{1}H NMR (500 MHz, DMSO-d_6) δ 7.91 (d, J = 7.5 Hz, 2H), 7.74 (d, J = 7.0 Hz, 2H), 7.49 (d, J = 8.0 Hz, 1H), 7.44 (t, J = 7.5 Hz, 2H), 7.35 (t, J = 7.5 Hz, 2H), 6.86 (t, J = 5.5 Hz, 1H), 4.31-4.32 (m, 2H), 4.25 (t, J = 7.0 Hz, 1H), 4.08-4.12 (m, 1H), 3.32-3.33 (m, 2H), 1.39 (s, 9H); HPLC analysis (CHIRALCEL AD-H, *i*-propanol/*n*-hexane = 10/90, flow rate = 0.8 mL/min, wave length = 254 nm), t_R = 14.78 min (major), 23.44 min (minor).

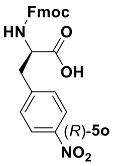

Amino acid (*R*)-5o: White solid (73.9 mg, 34% yield, >99% ee); ^{1}H NMR (500 MHz, DMSO-d_6) δ 12.92 (*br* s, 1H), 8.16 (d, J = 8.5 Hz, 2H), 7.89 (d, J = 7.5 Hz, 2H), 7.81 (d, J = 8.5 Hz, 1H), 7.63 (dd, J = 7.5, 3.5 Hz, 2H), 7.57 (d, J = 8.5 Hz, 2H), 7.42 (t, J = 7.5 Hz, 2H), 7.28-7.33 (m, 2H), 4.17-4.32 (m, 4H), 3.27 (dd, J = 13.5, 4.0 Hz, 1H), 3.04 (dd, J = 13.5, 11.0 Hz, 1H); HPLC analysis (CHIRALCEL OJ-H, *i*-propanol/*n*-hexane = 30/70, flow rate = 0.8 mL/min, wave length = 254 nm), t_R = 25.69 min (minor), 29.65 min (major).

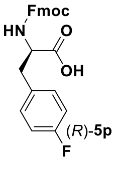

Amino acid (*R*)-5p: White solid (81.0 mg, 40% yield, 92% ee); ^{1}H NMR (500 MHz, DMSO-d_6) δ 12.83 (*br* s, 1H), 7.90 (d, J = 7.5 Hz, 2H), 7.75 (d, J = 8.5 Hz, 1H), 7.64-7.67 (m, 2H), 7.43 (t, J = 7.5 Hz, 2H), 7.30-7.35 (m, 4H), 7.11 (t, J = 9.0 Hz, 2H), 4.15-4.23 (m, 4H), 3.10 (dd, J = 13.5, 4.0 Hz, 1H), 2.88 (dd, J = 13.5, 11.0 Hz, 1H); HPLC analysis (CHIRALCEL AD-H, *i*-propanol/*n*-hexane = 10/90, flow rate = 0.8 mL/min, wave length = 254 nm), t_R = 22.98 min (major), 29.43 min (minor).

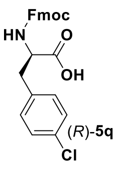

Amino acid (*R*)-5q: White solid (85.7 mg, 41% yield, 89% ee); ^{1}H NMR (500 MHz, DMSO-d_6) δ 12.82 (*br* s, 1H), 7.90 (d, J = 7.5 Hz, 2H), 7.77 (d, J = 8.5 Hz, 1H), 7.65 (t, J = 7.0 Hz, 2H), 7.43 (t, J = 7.5 Hz, 2H), 7.30-7.36 (m, 6H), 4.18-4.24 (m, 4H), 3.11 (dd, J = 8.5, 4.0 Hz, 1H), 2.86-2.91 (m, 1H); HPLC analysis (CHIRALCEL AD-H, *i*-propanol/*n*-hexane = 10/90, flow rate = 0.8 mL/min, wave length = 254 nm), t_R = 24.92 min (major), 29.18 min (minor).

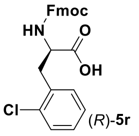

Amino acid (*R*)-5r: White solid (83.6 mg, 40% yield, >99% ee); ^{1}H NMR (500 MHz, DMSO-d_6) δ 12.89 (*br* s, 1H), 7.90 (d, J = 8.0 Hz, 2H), 7.83 (d,

J = 8.5 Hz, 1H), 7.66 (dd, J = 14.5, 7.5 Hz, 2H), 7.41-7.44 (m, 4H), 7.25-7.36 (m, 4H), 4.29-4.32 (m, 1H), 4.15-4.22 (m, 3H), 3.32 (dd, J = 13.5, 4.0 Hz, 1H), 2.96 (dd, J = 13.5, 11.5 Hz, 1H); HPLC analysis (CHIRALCEL AD-H, *i*-propanol/*n*-hexane = 10/90, flow rate = 0.8 mL/min, wave length = 254 nm), t_R = 27.53 min (major), 30.39 min (minor).

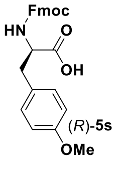

Amino acid (*R*)-5s: White solid (85.5 mg, 41% yield, 88% ee); ^{1}H NMR (500 MHz, DMSO-d_6) δ 12.74 (*br* s, 1H), 7.90 (d, J = 7.5 Hz, 2H), 7.72 (d, J = 8.5 Hz, 1H), 7.67 (t, J = 7.5 Hz, 2H), 7.43 (t, J = 7.5 Hz, 2H), 7.30-7.35 (m, 2H), 7.20 (d, J = 8.0 Hz, 2H), 6.85 (d, J = 8.0 Hz, 2H), 4.11-4.23 (m, 4H), 3.72 (s, 3H), 3.03 (dd, J = 13.5, 4.0 Hz, 1H), 2.82 (dd, J = 13.5, 11.0 Hz, 1H); HPLC analysis (CHIRALCEL AD-H, *i*-propanol/*n*-hexane = 10/90, flow rate = 0.8 mL/min, wave length = 254 nm), t_R = 31.08 min (major), 34.34 min (minor).

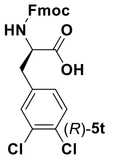

Amino acid (*R*)-5t: White solid (93.5 mg, 41% yield, >99% ee); ^{1}H NMR (500 MHz, DMSO-d_6) δ 7.90 (d, J = 7.5 Hz, 2H), 7.74 (d, J = 8.5 Hz, 1H), 7.63-7.65 (m, 2H), 7.59 (s, 1H), 7.54 (d, J = 8.0 Hz, 1H), 7.43 (t, J = 7.5 Hz, 2H), 7.28-7.34 (m, 3H), 4.18-4.24 (m, 4H), 3.14 (dd, J = 13.5, 4.0 Hz, 1H), 2.89 (dd, J = 13.5, 11.0 Hz, 1H); HPLC analysis (CHIRALCEL AD-H, *i*-propanol/*n*-hexane = 10/90, flow rate = 0.8 mL/min, wave length = 254 nm), t_R = 23.38 min (major), 26.81 min (minor).

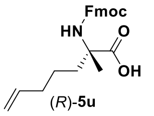

Amino acid (*R*)-5u: White solid (77.4 mg, 41% yield, 97% ee); ^{1}H NMR (500 MHz, DMSO-d_6) δ 12.40 (*br* s, 1H), 7.90 (d, J = 7.5 Hz, 2H), 7.73 (d, J = 7.5 Hz, 2H), 7.43 (t, J = 7.5 Hz, 3H), 7.34 (td, J = 7.5, 0.5 Hz, 2H), 5.75-5.83 (m, 1H), 5.01 (d, J = 17.0 Hz, 1H), 4.96 (d, J = 10.5 Hz, 1H), 4.20-4.28 (m, 3H), 2.00-2.01 (m, 2H), 1.77-1.82 (m, 1H), 1.67-1.72 (m, 1H), 1.32-1.35 (m, 5H); HPLC analysis (CHIRALCEL AS-H, *i*-propanol/*n*-hexane = 10/90, flow rate = 1.0 mL/min, wave length = 254 nm), t_R = 18.88 min (minor), 22.10 min (major).

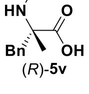

Amino acid (*R*)-5v: White solid (79.9 mg, 40% yield, 88% ee); ^{1}H NMR (500 MHz, DMSO-d_6) δ 12.57 (*br* s, 1H), 7.92 (dd, J = 7.0, 3.0 Hz, 2H), 7.74 (d, J = 7.5 Hz, 2H), 7.44 (t, J = 7.5 Hz, 2H), 7.36 (td, J = 7.5, 2.5 Hz, 2H), 7.21-7.22 (m, 3H), 7.01 (s, 2H), 4.52 (dd, J = 10.5, 6.5 Hz, 1H), 4.31-4.34 (m, 1H), 4.26 (t, J = 6.5 Hz, 1H), 3.28 (d, J = 13.0 Hz, 1H), 2.95 (d, J = 13.5 Hz, 1H), 1.19 (s, 3H); HPLC analysis (CHIRALCEL AD-H, *i*-propanol/*n*-hexane = 30/70, flow rate = 1.0 mL/min,

wave length = 254 nm), t_R = 8.06 min (minor), 10.68 min (major).

2.4. General procedure for the reaction of amino acid and alcohols

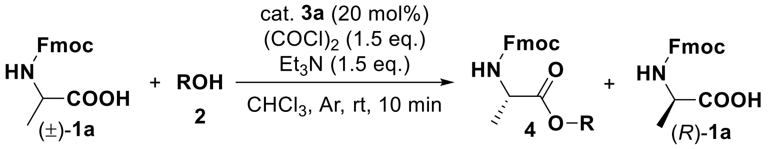

Racemic amino acid **1a** (0.5 mmol, 1.0 equiv.), alcohols **2** (0.3 mmol, 0.6 equiv.) and catalyst **3a** (0.1 mmol, 20 mol %) were well mixed in chloroform (1.0 mL). Then oxalyl chloride (0.75 mmol, 1.5 equiv.) and triethylamine (0.75 mmol, 1.5 equiv.) were added in sequence at ambient temperature under argon atmosphere. The resulting mixture was stirred at room temperature for 10 min. Subseqently, the mixture was partitioned between EtOAc (70 mL) and H_2O (30 mL) at room temperature. The organic layer was washed with saturated brine (30 mL×2), dried over Na_2SO_4, and concentrated *in vacuo*. The resulting residue was dissolved in dichloromethane (10 mL) and 1M aqueous solution NaOH (3 mL). The organic layer and the aqueous layer were separated. The organic layer was washed with saturated brine (10 mL×2), dried over Na_2SO_4, and concentrated *in vacuo*. The resulting residue was purified *via* silica gel colunm chromatography to yield esters (*S*,*S*)-**4**. The aqueous layer was washed with dichloromethane (10 mL×2). The organic layer was discarded. The aqueous layer was made acidic with excess 1M aqueous solution HCl (to pH ~5) and was extracted with dichloromethane (10 mL×2). The combined organic layer was washed with brine (20 mL×2), dried over Na_2SO_4, then filtered and evaporated to afford the recovered amino acids (*R*)-**1a**.

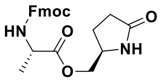

(*S*,*R*)-**S4a**

Ester (*S*,*R*)-S4a: Purified by flash chromatography on silica gel, eluting with MeOH/DCM 5%-10% (v/v); White solid (92.3 mg, 45% yield, 58:42 dr); m.p. 86-88 °C; ^{1}H NMR (500 MHz, $CDCl_3$, *mixture of two diastereomers*) δ 7.98 (s, 0.9H), 7.75 (d, J = 7.5 Hz, 4.4H), 7.54-7.60 (m, 4.0H), 7.38 (t, J = 7.5 Hz, 4.0H), 7.26-7.31 (m, 3.9H), 6.46 (d, J = 8.5 Hz, 1.1H), 6.17 (d, J = 8.0 Hz, 0.8H), 4.40-4.48 (m, 4.1H), 4.31-4.36 (m, 2.8H), 4.22-4.23 (m, 2.2H), 4.17 (d, J = 8.5 Hz, 1.0H), 3.92-3.98 (m, 2.9H), 3.82 (t, J = 10.0 Hz, 1.1H), 2.31-2.36 (m, 4.1H), 2.17-2.22 (m, 2.0H), 1.67-1.74 (m, 2.0H), 1.44 (t, J = 7.5 Hz, 6.0H); ^{13}C NMR (125 MHz, $CDCl_3$, *mixture of two diastereomers*) δ 178.6, 178.5, 172.6(3), 172.5(5), 156.1, 155.9, 143.9, 143.8, 141.4, 127.7, 127.1, 127.0, 125.1, 120.0, 68.0, 67.9, 67.0, 66.9,

52.9(4), 52.8(7), 49.9, 47.3, 47.2, 29.8, 29.6, 22.9, 22.8, 18.4; HRMS (FTMS-ESI) m/z: $[M+Na]^+$ calcd for $C_{23}H_{24}N_2NaO_5^+$ 431.1577, found 431.1557.

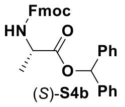

Ester (*S*)-S4b: Purified by flash chromatography on silica gel, eluting with ethyl acetate/hexane 10%-20% (v/v); Colorless oil (183.1 mg, 77% yield, 1% ee); ^{1}H NMR (500 MHz, $CDCl_3$) δ 7.74 (d, J = 7.5 Hz, 2H), 7.57 (d, J = 7.5 Hz, 2H), 7.36-7.38 (m, 4H), 7.22-7.31 (m, 10H), 6.89 (s, 1H), 5.38 (d, J = 7.5 Hz, 1H), 4.52 (t, J = 7.5 Hz, 1H), 4.32-4.40 (m, 2H), 4.19 (t, J = 7.0 Hz, 1H), 1.45 (d, J = 7.0 Hz, 3H); ^{13}C NMR (125 MHz, $CDCl_3$) δ 172.0, 155.6, 144.0, 143.9, 141.4, 139.7, 139.5, 128.6, 128.5, 128.1, 127.7, 127.5, 127.0(9), 127.0(5), 126.6, 125.1, 120.0, 78.1, 76.3, 67.1, 47.3, 18.6; HRMS (FTMS-ESI) m/z: $[M+H]^+$ calcd for $C_{31}H_{28}NO_4^+$ 478.2013, found 478.2036; HPLC analysis (CHIRALCEL OD-H, *i*-propanol/*n*-hexane = 30/70, flow rate = 0.8 mL/min, wave length = 254 nm), t_R = 14.20 min (minor), 17.40 min (major).

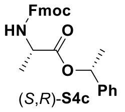

Ester (*S*,*R*)-S4c: Purified by flash chromatography on silica gel, eluting with ethyl acetate/hexane 10%-20% (v/v); Colorless oil (145.8 mg, 70% yield, 72:28 dr); ^{1}H NMR (500 MHz, $CDCl_3$, *mixture of two diastereomers*) δ 7.75 (d, J = 7.5 Hz, 4.0H), 7.57-7.58 (m, 3.8H), 7.29-7.40 (m, 20.2H), 5.89-5.94 (m, 1.9H), 5.38-5.41 (m, 1.7H), 4.86-4.90 (m, 0.6H), 4.34-4.44 (m, 5.1H), 4.20-4.21 (m, 2.1H), 1.56 (t, J = 6.5 Hz, 6.2H), 1.45-1.49 (m, 5.0H), 1.37 (d, J = 6.5 Hz, 3.1H); ^{13}C NMR (125 MHz, $CDCl_3$, *mixture of two diastereomers*) δ 172.2, 155.6(3), 155.5(6), 146.0, 144.0, 143.9, 141.4, 141.2, 128.6(0), 128.5(8), 128.5, 128.1(3), 128.0(8), 127.7, 127.4, 127.1, 126.1, 126.0, 125.4, 125.1, 120.0, 73.7, 73.6, 70.4, 67.1, 47.3, 25.2, 22.1, 22.0, 18.8, 18.6; HRMS (FTMS-ESI) m/z: $[M+H]^+$ calcd for $C_{26}H_{26}NO_4^+$ 416.1856, found 416.1836.

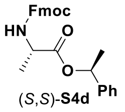

Ester (*S*,*S*)-S4d: Purified by flash chromatography on silica gel, eluting with ethyl acetate/hexane 10%-20% (v/v); Colorless oil (143.3 mg, 69% yield, 62:38 dr); ^{1}H NMR (500 MHz, $CDCl_3$, *mixture of two diastereomers*) δ 7.75 (d, J = 7.5 Hz, 2.0H), 7.57-7.59 (m, 1.9H), 7.37-7.40 (m, 2.2H), 7.32-7.36 (m, 6.0H), 7.27-7.30 (m, 2.4H), 5.89-5.93 (m, 1.0H), 5.37-5.40 (m, 0.9H), 4.35-4.46 (m, 3.0H), 4.20-4.22 (m, 1.0H), 1.55-1.57 (m, 3.1H), 1.45-1.49 (m, 2.6H); ^{13}C NMR (125 MHz, $CDCl_3$, *mixture of two diastereomers*) δ 172.2, 155.7, 145.9, 144.0, 143.9, 141.4, 141.2, 140.9, 128.5(9), 128.5(8), 128.5, 128.1(2), 128.0(8), 127.7, 127.4, 127.1, 126.1, 126.0, 125.4, 125.1, 120.0, 73.7, 73.6, 70.4, 47.3, 25.2, 22.1, 22.0, 18.8, 18.6; HRMS

(FTMS-ESI) m/z: $[M+H]^+$ calcd for $C_{26}H_{26}NO_4^+$ 416.1856, found 416.1833.

2.5. Synthetic procedure of 5 mmol scale model reaction

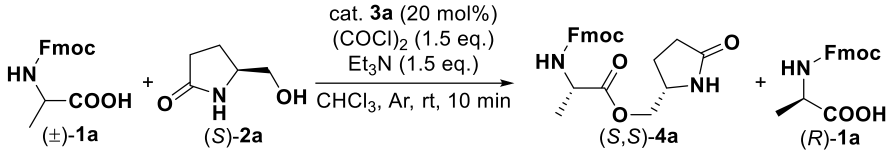

Racemic amino acid **1a** (1.56 g, 5.0 mmol, 1.0 equiv.), *L*-pyroglutaminol **2a** (0.35 g, 3.0 mmol, 0.6 equiv.) and catalyst **3a** (0.30 g, 1.0 mmol, 20 mol %) were well mixed in chloroform (10.0 mL). Then oxalyl chloride (0.95 g, 7.5 mmol, 1.5 equiv.) and triethylamine (0.76 g, 7.5 mmol, 1.5 equiv.) were added in sequence at ambient temperature under argon atmosphere. The resulting mixture was stirred at room temperature for 10 min. Subseqently, the mixture was partitioned between EtOAc (150 mL) and H_2O (50 mL) at room temperature. The organic layer was washed with saturated brine (50 mL×2), dried over Na_2SO_4, and concentrated *in vacuo*. The resulting residue was dissolved in dichloromethane (100 mL) and 1M aqueous solution NaOH (30 mL). The organic layer and the aqueous layer were separated. The organic layer was washed with saturated brine (30 mL×2), dried over Na_2SO_4, and concentrated *in vacuo*. The resulting residue was purified *via* silica gel colunm chromatography (MeOH/DCM = 5%-10%) to yield ester (*S*,*S*)-**4a** in 44% yield (0.90 g, white solid) with 98:2 dr. The aqueous layer was washed with dichloromethane (30 mL×2). The organic layer was discarded. The aqueous layer was made acidic with excess 1M aqueous solution HCl (to pH ~5) and was extracted with dichloromethane (50 mL×2). The combined organic layer was washed with brine (40 mL×2), dried over Na_2SO_4, then filtered and evaporated to afford the recovered amino acid (*R*)-**1a** in 40% yield (0.62 g, white solid) with >99% ee.

2.6. Control experiments

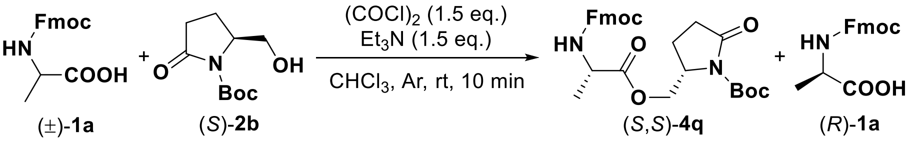

Racemic amino acid **1a** (0.5 mmol, 1.0 equiv.), *N*-Boc-*L*-pyroglutaminol **2b** (0.3 mmol, 0.6 equiv.) and catalyst **3a** (0.1 mmol, 20 mol %) were well mixed in chloroform (1.0 mL). Then oxalyl chloride (0.75 mmol, 1.5 equiv.) and triethylamine (0.75 mmol, 1.5

equiv.) were added in sequence at ambient temperature under argon atmosphere. The resulting mixture was stirred at room temperature for 10 min. Subseqently, the mixture was partitioned between EtOAc (70 mL) and H_2O (30 mL) at room temperature. The organic layer was washed with saturated brine (30 mL×2), dried over Na_2SO_4, and concentrated *in vacuo*. The resulting residue was dissolved in dichloromethane (10 mL) and 1M aqueous solution NaOH (3 mL). The organic layer and the aqueous layer were separated. The organic layer was washed with saturated brine (10 mL×2), dried over Na_2SO_4, and concentrated *in vacuo*. The resulting residue was purified *via* silica gel colunm chromatography (MeOH/DCM = 5%-10%) to yield ester (*S*,*S*)-**4q** in 77% yield (195.0 mg, colorless oil) with 51:49 dr. The aqueous layer was washed with dichloromethane (10 mL×2). The organic layer was discarded. The aqueous layer was made acidic with excess 1M aqueous solution HCl (to pH ~5) and was extracted with dichloromethane (10 mL×2). The combined organic layer was washed with brine (20 mL×2), dried over Na_2SO_4, then filtered and evaporated to afford the recovered amino acid (*R*)-**1a** in < 10% yield.

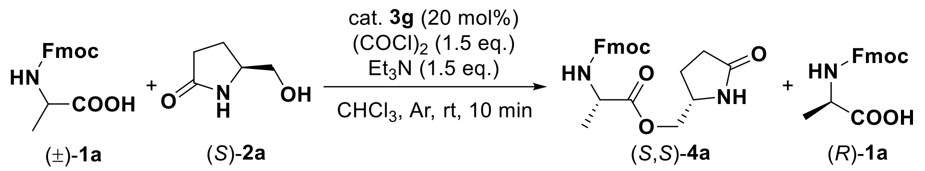

Racemic amino acid **1a** (0.5 mmol, 1.0 equiv.), *L*-pyroglutaminol **2a** (0.3 mmol, 0.6 equiv.) and catalyst **3g** (0.1 mmol, 20 mol %) were well mixed in chloroform (1.0 mL). Then oxalyl chloride (0.75 mmol, 1.5 equiv.) and triethylamine (0.75 mmol, 1.5 equiv.) were added in sequence at ambient temperature under argon atmosphere. The resulting mixture was stirred at room temperature for 10 min. Subseqently, the mixture was partitioned between EtOAc (70 mL) and H_2O (30 mL) at room temperature. The organic layer was washed with saturated brine (30 mL×2), dried over Na_2SO_4, and concentrated *in vacuo*. The resulting residue was dissolved in dichloromethane (10 mL) and 1M aqueous solution NaOH (3 mL). The organic layer and the aqueous layer were separated. The organic layer was washed with saturated brine (10 mL×2), dried over Na_2SO_4, and concentrated *in vacuo*. The resulting residue was purified *via* silica gel colunm chromatography (MeOH/DCM = 5%-10%) to yield ester (*S*,*S*)-**4a** in 68% yield (139.1 mg, white solid) with 53:47 dr. The aqueous layer was washed with dichloromethane (10 mL×2). The organic layer was discarded. The aqueous layer was made acidic with excess 1M aqueous solution HCl (to pH ~5) and was extracted with dichloromethane

(10 mL×2). The combined organic layer was washed with brine (20 mL×2), dried over Na_2SO_4, then filtered and evaporated to afford the recovered amino acid (*R*)-**1a** in < 10% yield.

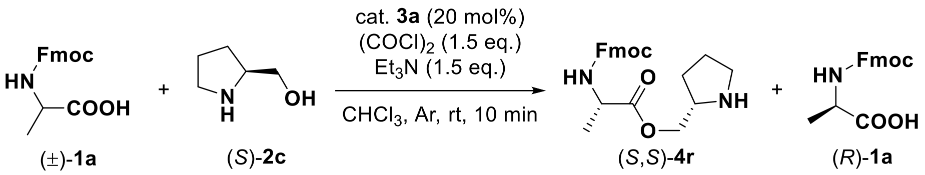

Racemic amino acid **1a** (0.5 mmol, 1.0 equiv.), *L*-prolinol **2c** (0.3 mmol, 0.6 equiv.) and catalyst **3a** (0.1 mmol, 20 mol %) were well mixed in chloroform (1.0 mL). Then oxalyl chloride (0.75 mmol, 1.5 equiv.) and triethylamine (0.75 mmol, 1.5 equiv.) were added in sequence at ambient temperature under argon atmosphere. The resulting mixture was stirred at room temperature for 10 min. Subseqently, the mixture was partitioned between EtOAc (70 mL) and H_2O (30 mL) at room temperature. The organic layer was washed with saturated brine (30 mL×2), dried over Na_2SO_4, and concentrated *in vacuo*. The resulting residue was dissolved in dichloromethane (10 mL) and 1M aqueous solution NaOH (3 mL). The organic layer and the aqueous layer were separated. The organic layer was washed with saturated brine (10 mL×2), dried over Na_2SO_4, and concentrated *in vacuo*. The resulting residue was purified *via* silica gel colunm chromatography (MeOH/DCM = 1%-3%) to yield ester (*S,S*)-**4r** in 67% yield (132.5 mg, colorless oil) with 55:45 dr. The aqueous layer was washed with dichloromethane (10 mL×2). The organic layer was discarded. The aqueous layer was made acidic with excess 1M aqueous solution HCl (to pH ~5) and was extracted with dichloromethane (10 mL×2). The combined organic layer was washed with brine (20 mL×2), dried over Na_2SO_4, then filtered and evaporated to afford the recovered amino acid (*R*)-**1a** in < 10% yield.

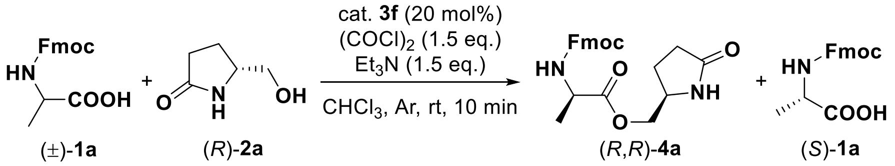

Racemic amino acid **1a** (0.5 mmol, 1.0 equiv.), *D*-pyroglutaminol **2a** (0.3 mmol, 0.6 equiv.) and catalyst **3f** (0.1 mmol, 20 mol %) were well mixed in chloroform (1.0 mL). Then oxalyl chloride (0.75 mmol, 1.5 equiv.) and triethylamine (0.75 mmol, 1.5 equiv.) were added in sequence at ambient temperature under argon atmosphere. The resulting

mixture was stirred at room temperature for 10 min. Subseqently, the mixture was partitioned between EtOAc (70 mL) and H_2O (30 mL) at room temperature. The organic layer was washed with saturated brine (30 mL×2), dried over Na_2SO_4, and concentrated *in vacuo*. The resulting residue was dissolved in dichloromethane (10 mL) and 1M aqueous solution NaOH (3 mL). The organic layer and the aqueous layer were separated. The organic layer was washed with saturated brine (10 mL×2), dried over Na_2SO_4, and concentrated *in vacuo*. The resulting residue was purified *via* silica gel colunm chromatography (MeOH/DCM = 5%-10%) to yield ester (*R*,*R*)-**4a** in 41% yield (83.9 mg, white solid) with 99:1 dr. The aqueous layer was washed with dichloromethane (10 mL×2). The organic layer was discarded. The aqueous layer was made acidic with excess 1M aqueous solution HCl (to pH ~5) and was extracted with dichloromethane (10 mL×2). The combined organic layer was washed with brine (20 mL×2), dried over Na_2SO_4, then filtered and evaporated to afford the recovered amino acid (*S*)-**1a** in 42% yield (65.4 mg, white solid) with >99% ee.

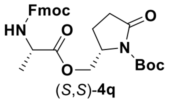

(*S*,*S*)-**4q**

Ester (*S*,*S*)-4q: Purified by flash chromatography on silica gel, eluting with MeOH/DCM 5%-10% (v/v); Colorless oil (195.0 mg, 77% yield, 51:49 dr); ^{1}H NMR (500 MHz, $CDCl_3$, *mixture of two diastereomers*) δ 7.76 (d, J = 7.5 Hz, 2.0H), 7.60 (d, J = 6.5 Hz, 2.0H), 7.40 (t, J = 7.5 Hz, 2.0H), 7.31 (t, J = 7.5 Hz, 2.1H), 5.30 (d, J = 6.5 Hz, 0.9H), 4.39-4.50 (m, 5.2H), 4.27-4.29 (m, 0.9H), 4.22 (t, J = 7.0 Hz, 1.2H), 2.57-2.71 (m, 1.0H), 2.40-2.48 (m, 1.1H), 2.12-2.22 (m, 1.1H), 1.88-1.96 (m, 1.0H), 1.54 (s, 9.3H), 1.41-1.45 (m, 2.9H); ^{13}C NMR (125 MHz, $CDCl_3$, *mixture of two diastereomers*) δ 173.6, 173.4, 172.8, 172.6, 155.6(3), 155.6(0), 149.8, 149.7, 143.9, 143.8, 141.4, 127.7, 127.0(8), 127.0(6), 125.0, 120.0, 83.5, 67.1, 65.6, 65.5, 56.0, 55.9, 49.8, 47.2, 31.6, 31.4, 28.0, 20.8, 20.7, 18.5, 18.3; HRMS (FTMS-ESI) m/z: $[M+Na]^+$ calcd for $C_{28}H_{32}N_2NaO_7^+$ 531.2102, found 531.2094; HPLC analysis (CHIRALCEL OD-H, *i*-propanol/*n*-hexane = 30/70, flow rate = 0.8 mL/min, wave length = 254 nm), t_R = 22.36 min (major), 27.75 min (minor).

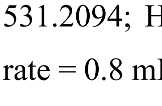

(*S*,*S*)-**4r**

Ester (*S*,*S*)-4r: Purified by flash chromatography on silica gel, eluting with MeOH/DCM 1%-3% (v/v); Colorless oil (132.5 mg, 67% yield, 55:45 dr); $[\alpha]_D^{20}$ = 3.9 (c = 0.5 in DCM); ^{1}H NMR (500 MHz, $CDCl_3$, *mixture of two diastereomers*) δ 7.76 (d, J = 7.5 Hz, 2.0H), 7.59-7.60 (m, 2.0H), 7.39 (t, J = 7.5 Hz, 2.0H), 7.31 (td, J = 7.0, 2.5 Hz, 2.0H), 5.81-5.85 (m, 0.8H), 4.68 (d, J = 0.5 Hz, 0.5H), 4.49-4.57 (m, 1.4H), 4.33-4.37 (m, 2.0H), 4.14-4.26 (m, 2.0H), 3.67-3.76 (m, 1.4H),

3.53-3.63 (m, 2.0H), 3.42-3.47 (m, 0.5H), 2.01-2.08 (m, 1.1H), 1.95-2.00 (m, 1.2H), 1.84-1.91 (m, 1.2H), 1.60-1.71 (m, 0.9H), 1.38-1.40 (m, 1.5H), 1.34-1.36 (m, 1.5H); ^{13}C NMR (125 MHz, $CDCl_3$, *mixture of two diastereomers*) δ 173.6, 172.9, 155.7, 144.0, 143.9, 141.3, 127.7, 127.1, 125.1, 120.0, 67.0, 66.6, 66.3, 61.7, 61.3, 48.8, 48.6, 47.8, 47.7, 47.3, 28.2, 27.9, 24.5, 24.3, 18.9, 18.2; HRMS (FTMS-ESI) m/z: $[M+Na]^+$ calcd for $C_{23}H_{26}N_2NaO_4^+$ 417.1785, found 417.1784; HPLC analysis (CHIRALCEL AD-H, *i*-propanol/*n*-hexane = 10/90, flow rate = 1.0 mL/min, wave length = 254 nm), t_R = 22.14 min (major), 27.30 min (minor).

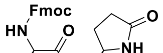
(*R*,*R*)-4a

Ester (*R*,*R*)-4a: Purified by flash chromatography on silica gel, eluting with MeOH/DCM 5%-10% (v/v); White solid (83.9 mg, 41% yield, 99:1 dr); m.p. 130-131 °C; $[\alpha]_D^{20}$ = -92.3 (c = 0.5 in DCM); ^{1}H NMR (500 MHz, $CDCl_3$) δ 8.06 (s, 1H), 7.78 (d, *J* = 7.5 Hz, 2H), 7.64 (d, *J* = 7.5 Hz, 2H), 7.41 (t, *J* = 7.5 Hz, 2H), 7.30-7.34 (m, 2H), 6.52 (d, *J* = 8.5 Hz, 1H), 4.46-4.52 (m, 2H), 4.35-4.39 (m, 1H), 4.22-4.27 (m, 2H), 3.95-4.03 (m, 2H), 2.34-2.42 (m, 2H), 2.18-2.25 (m, 1H), 1.74-1.81 (m, 1H), 1.47 (d, *J* = 7.5 Hz, 3H); ^{13}C NMR (125 MHz, $CDCl_3$) δ 179.0, 172.8, 156.3, 144.0, 143.8, 141.3, 127.7, 127.0(4), 126.9(9), 125.2, 120.0, 67.9, 66.9, 53.0, 49.9, 47.2, 30.0, 22.7, 18.5; HRMS (FTMS-ESI) m/z: $[M+Na]^+$ calcd for $C_{23}H_{24}N_2NaO_5^+$ 431.1577, found 431.1581; HPLC analysis (CHIRALCEL OD-H, *i*-propanol/*n*-hexane = 30/70, flow rate = 0.8 mL/min, wave length = 254 nm), t_R = 13.34 min (major), 19.03 min (minor).

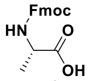
(*S*)-1a

Amino acid (*S*)-1a: White solid (65.4 mg, 42% yield, >99% ee); $[\alpha]_D^{20}$ = 14.9 (c = 0.5 in H_2O); ^{1}H NMR (500 MHz, DMSO-*d*$_6$) δ 12.54 (*br* s, 1H), 7.91 (d, *J* = 7.5 Hz, 2H), 7.73-7.75 (m, 2H), 7.68 (d, *J* = 7.5 Hz, 1H), 7.43 (t, *J* = 7.5 Hz, 2H), 7.35 (t, *J* = 7.5 Hz, 2H), 4.29-4.31 (m, 2H), 4.22-4.25 (m, 1H), 3.99-4.05 (m, 1H), 1.29 (d, *J* = 7.0 Hz, 3H); HPLC analysis (CHIRALCEL AS-H, *i*-propanol/*n*-hexane = 10/90, flow rate = 0.8 mL/min, wave length = 254 nm), t_R = 19.53 min (major), 24.79 min (minor).

2.7. High-resolution mass spectrometry of the intermediates A and B in Figure 7

Figure S3. High-resolution mass spectrometry of the intermediate **A**

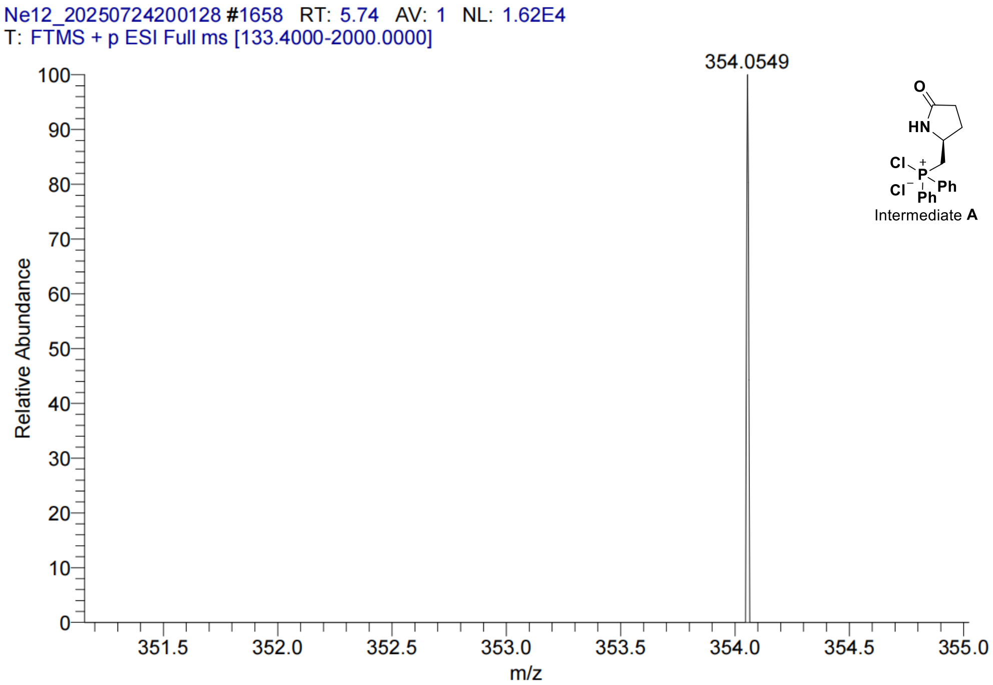

HRMS (FTMS-ESI) m/z: $[M+H]^+$ calcd for $C_{17}H_{19}Cl_2NOP^+$ 354.0576, found 354.0549.

Figure S4. High-resolution mass spectrometry of the intermediate **B**

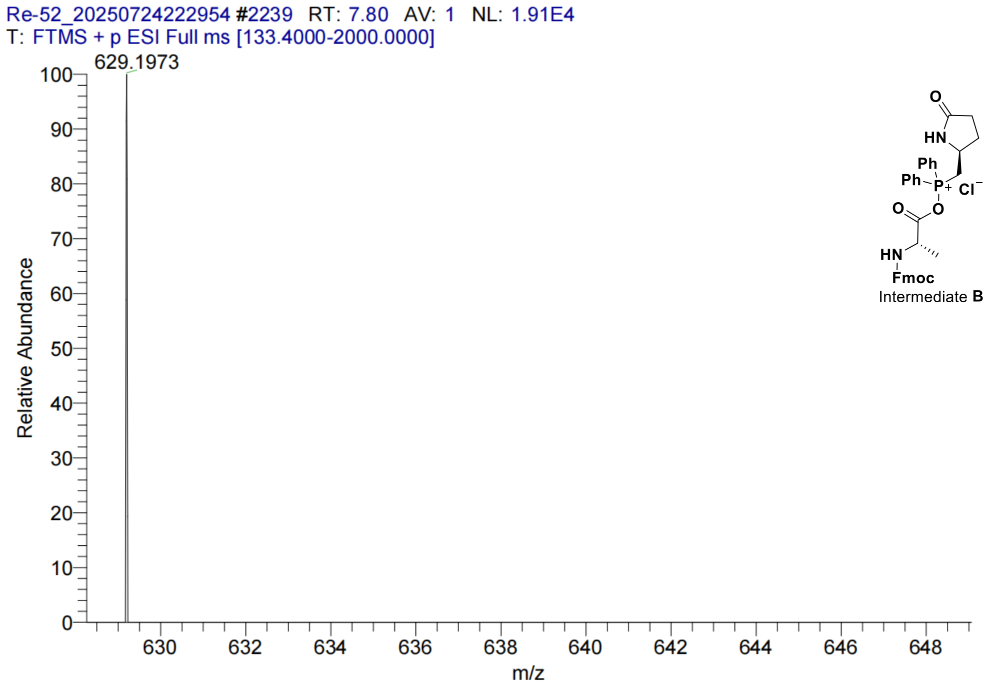

HRMS (FTMS-ESI) m/z: $[M+H]^+$ calcd for $C_{35}H_{35}ClN_2O_5P^+$ 629.1967, found 629.1973.

3. NMR Spectra

500 MHz, $CDCl_3$, ^{1}H NMR

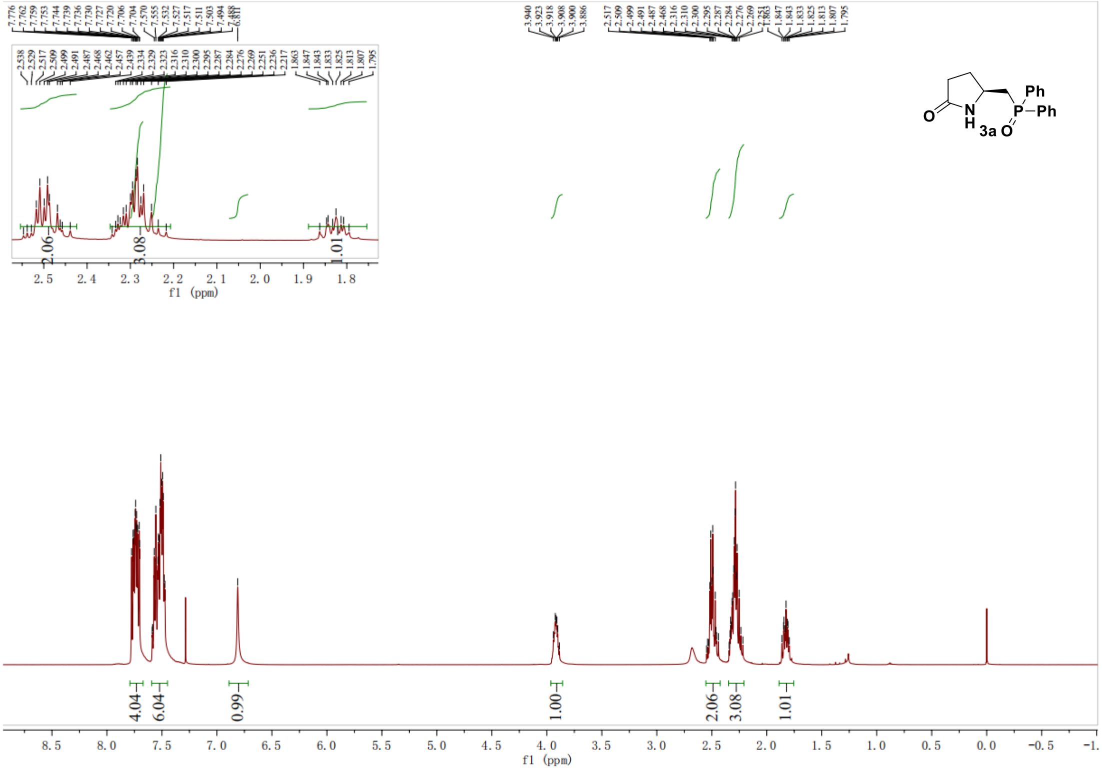

125 MHz, $CDCl_3$, ^{13}C NMR

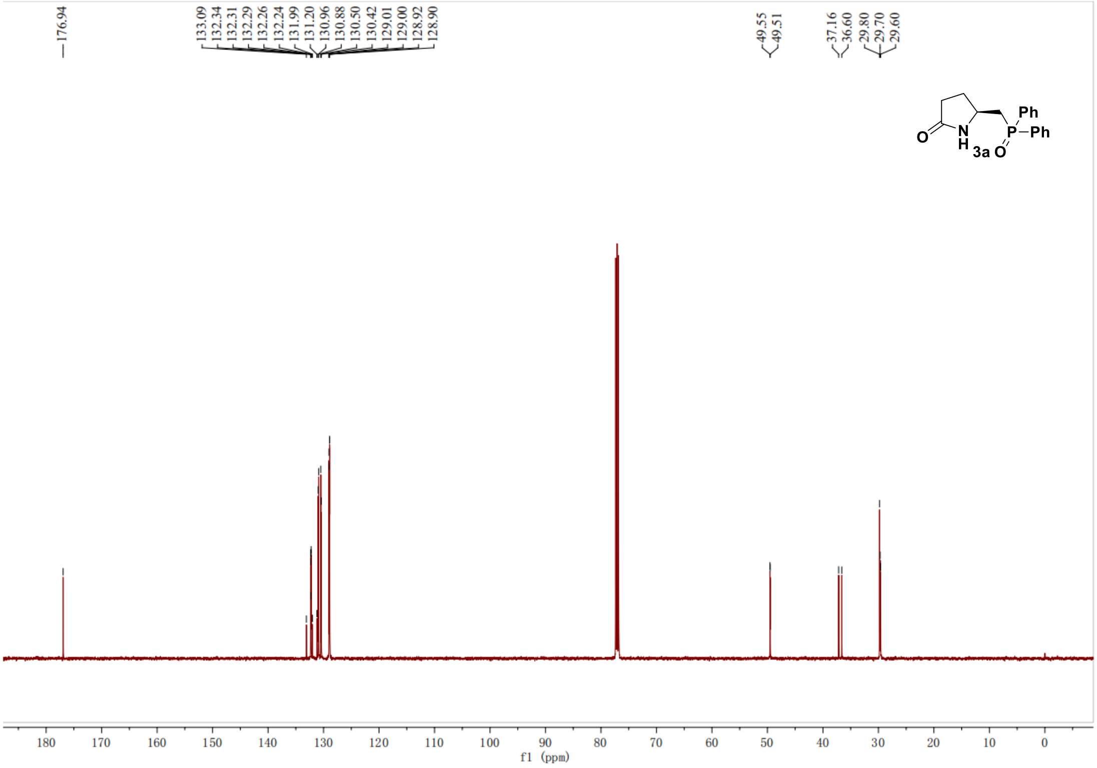

203 MHz, $CDCl_3$, ^{31}P NMR

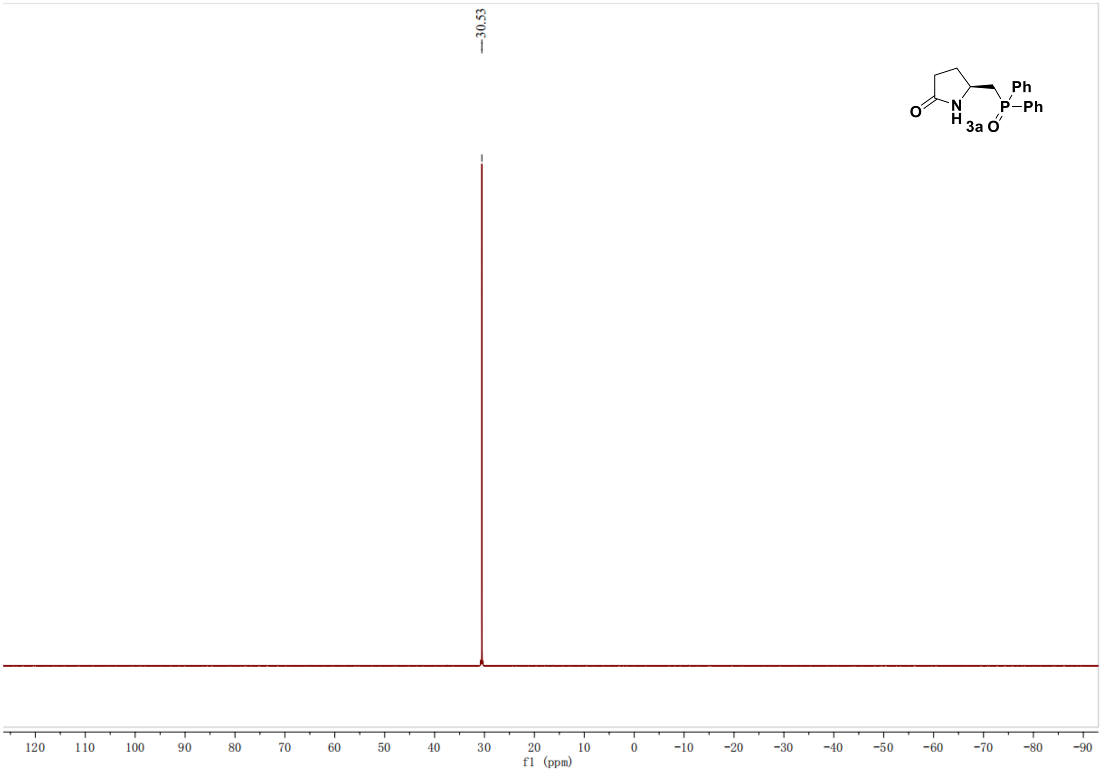

500 MHz, $CDCl_3$, ^{1}H NMR

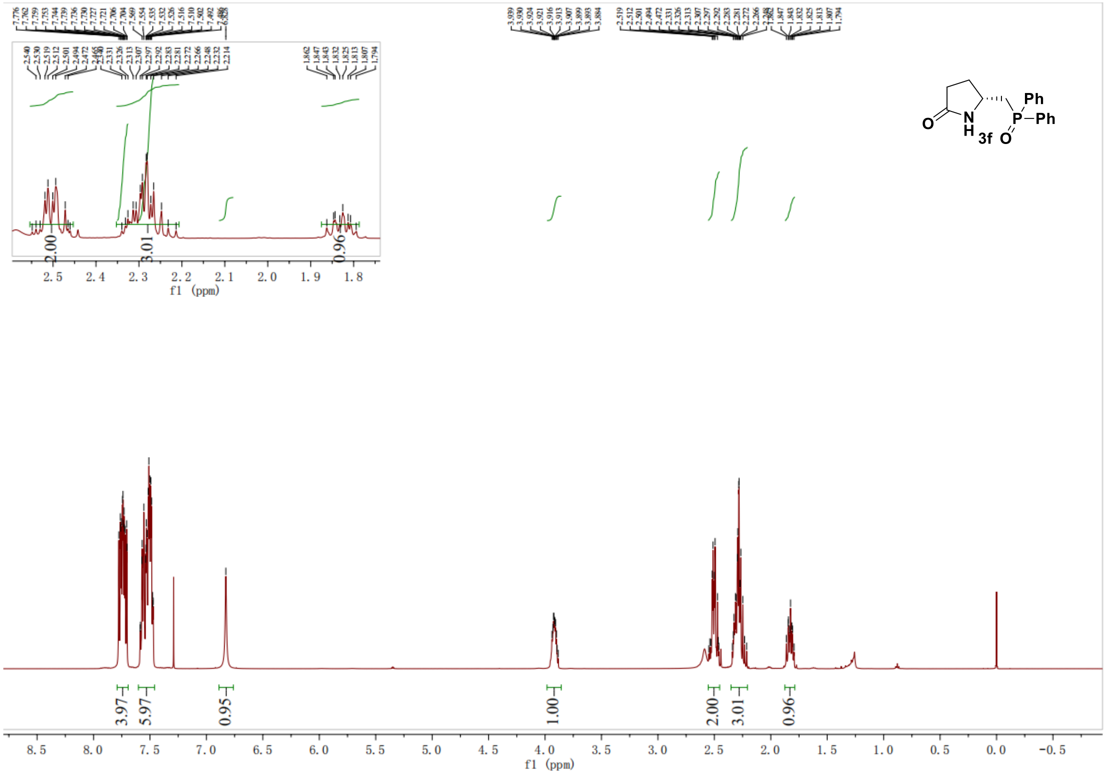

125 MHz, $CDCl_3$, ^{13}C NMR

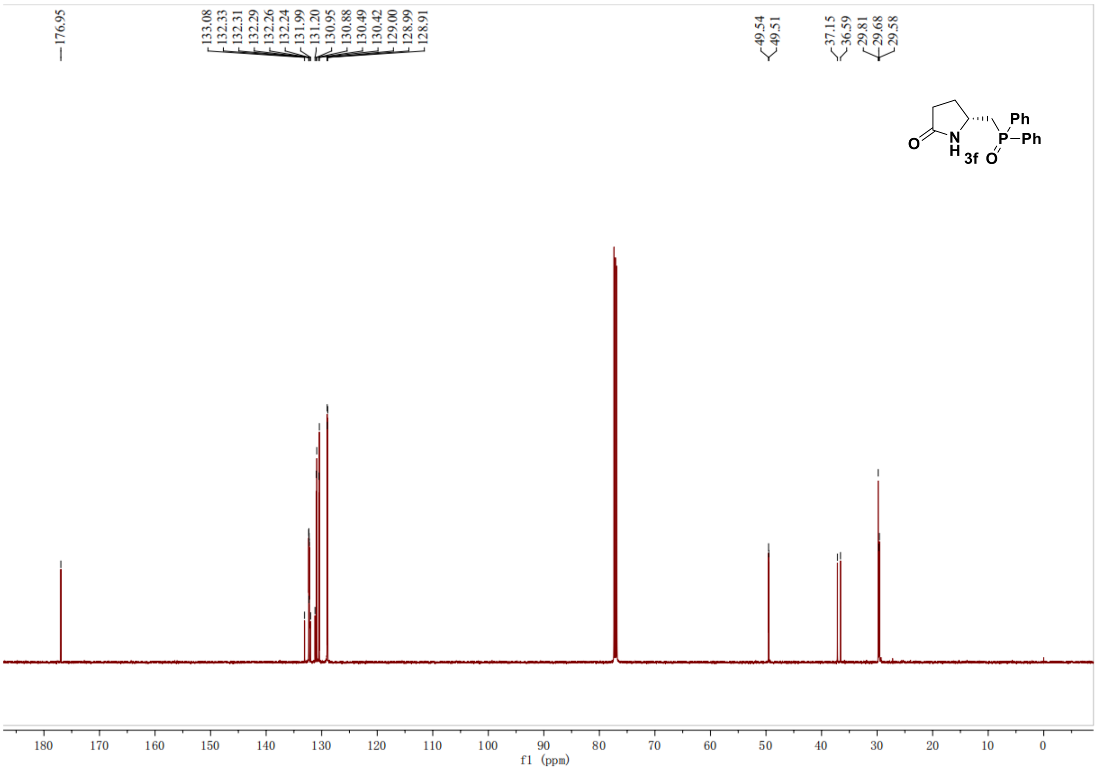

203 MHz, $CDCl_3$, ^{31}P NMR

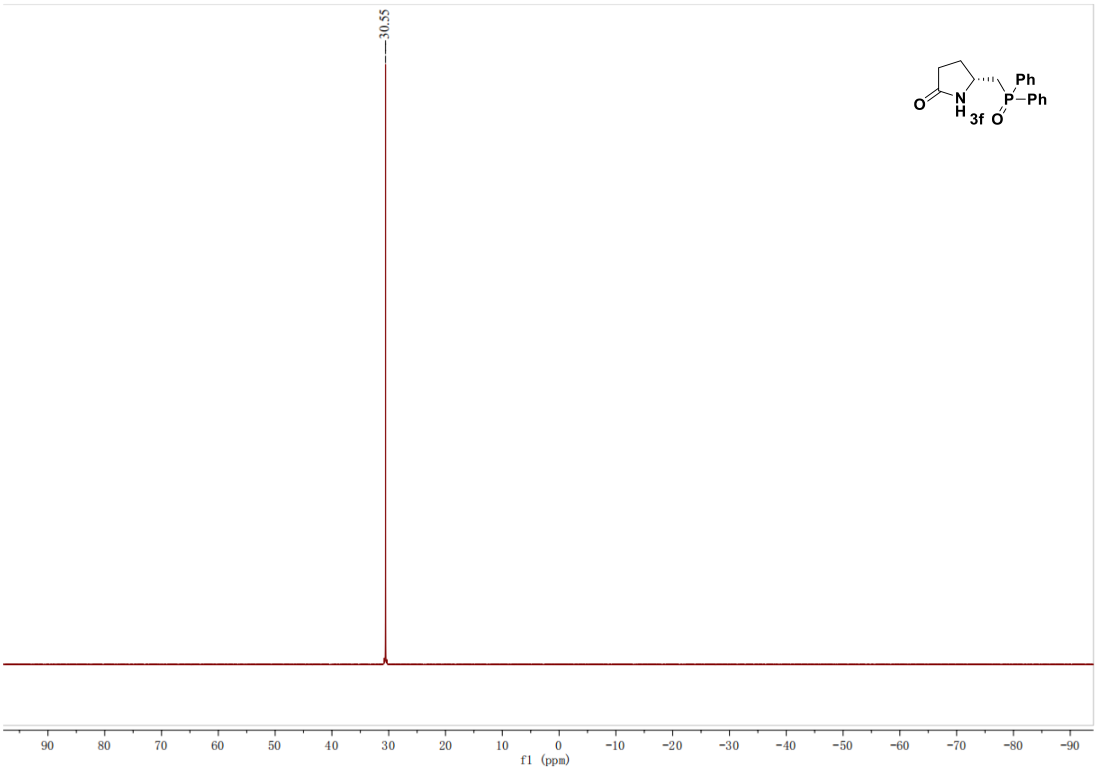

500 MHz, $CDCl_3$, ^{1}H NMR

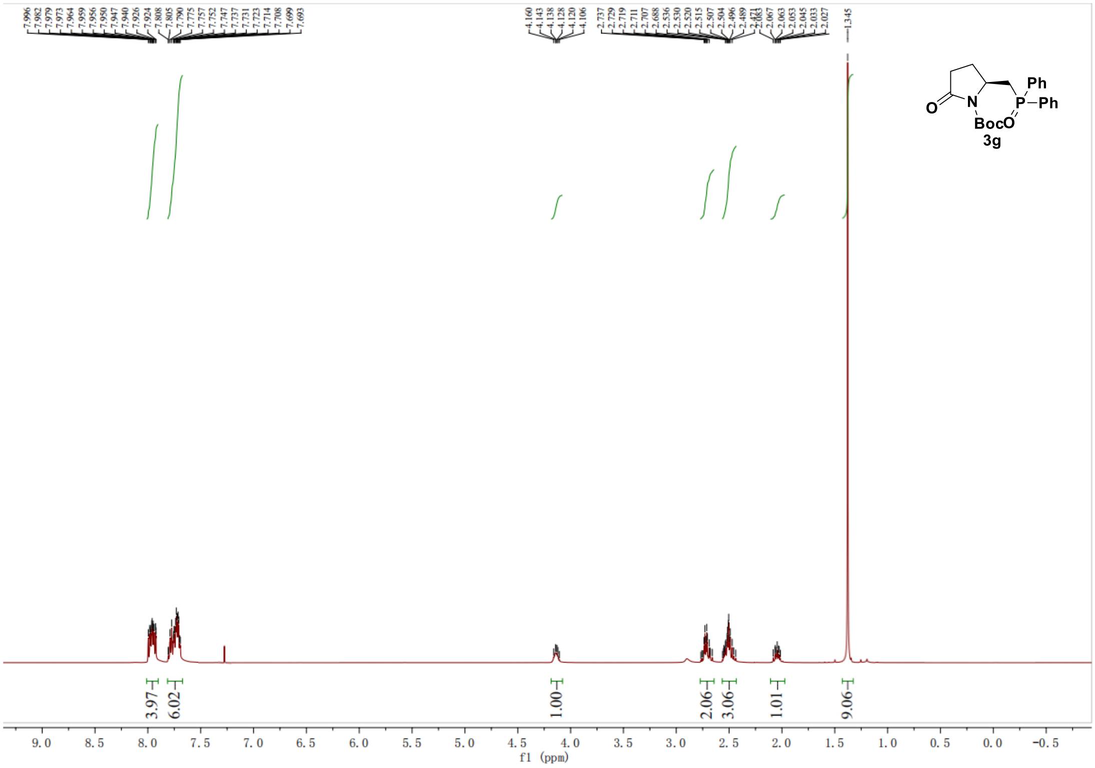

125 MHz, $CDCl_3$, ^{13}C NMR

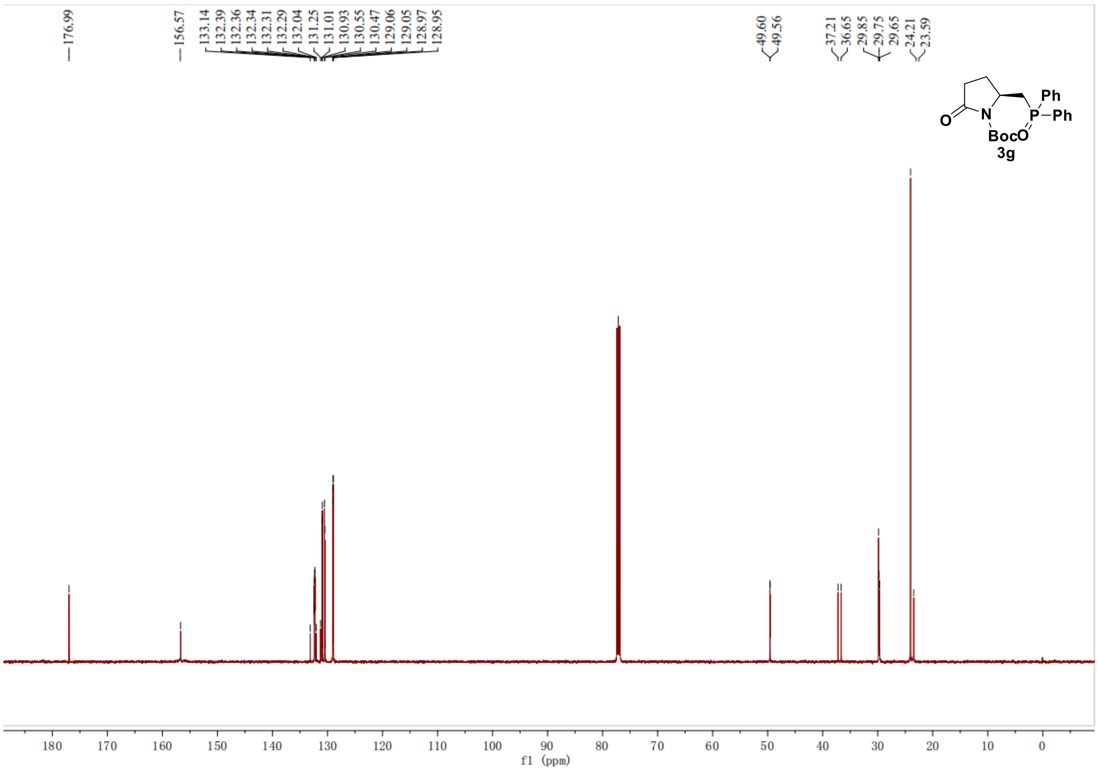

203 MHz, $CDCl_3$, ^{31}P NMR

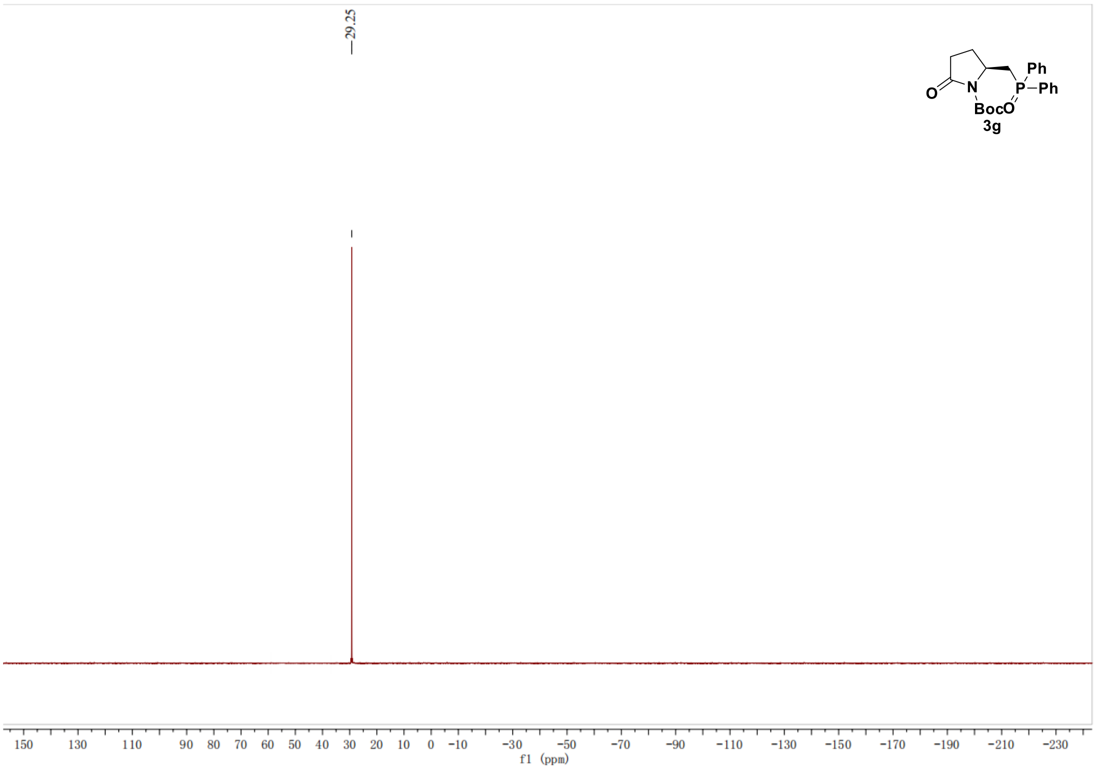

500 MHz, $CDCl_3$, ^1H NMR

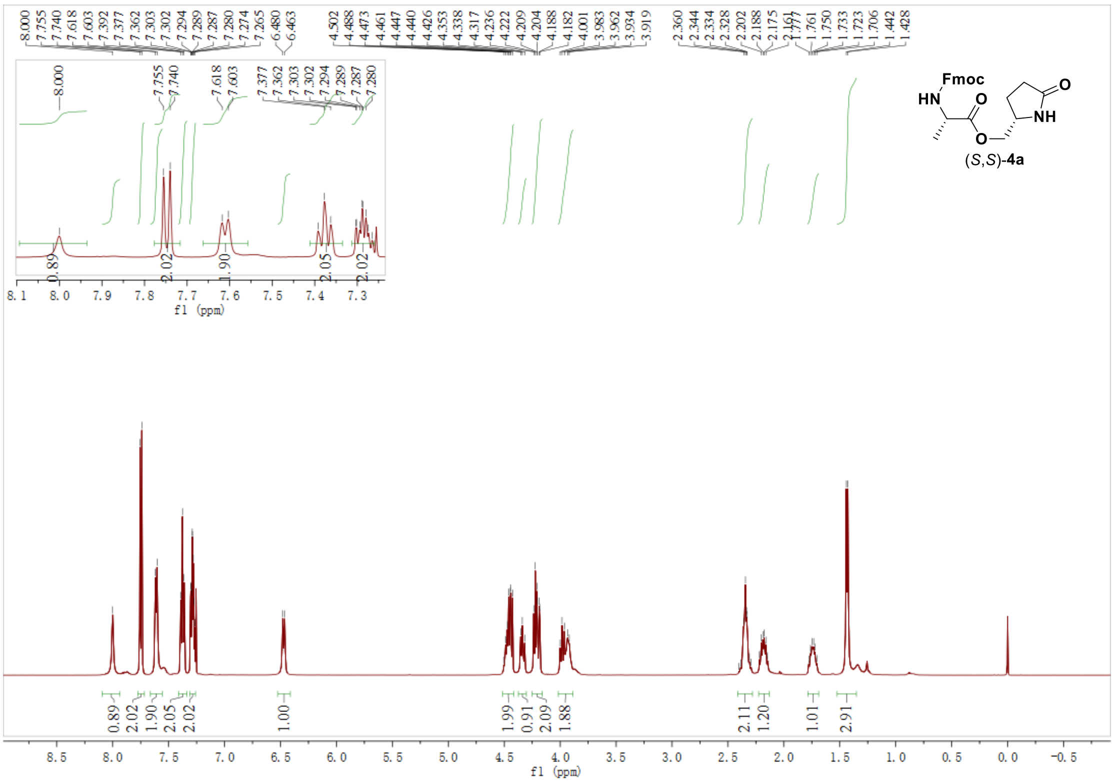

125 MHz, $CDCl_3$, ^{13}C NMR

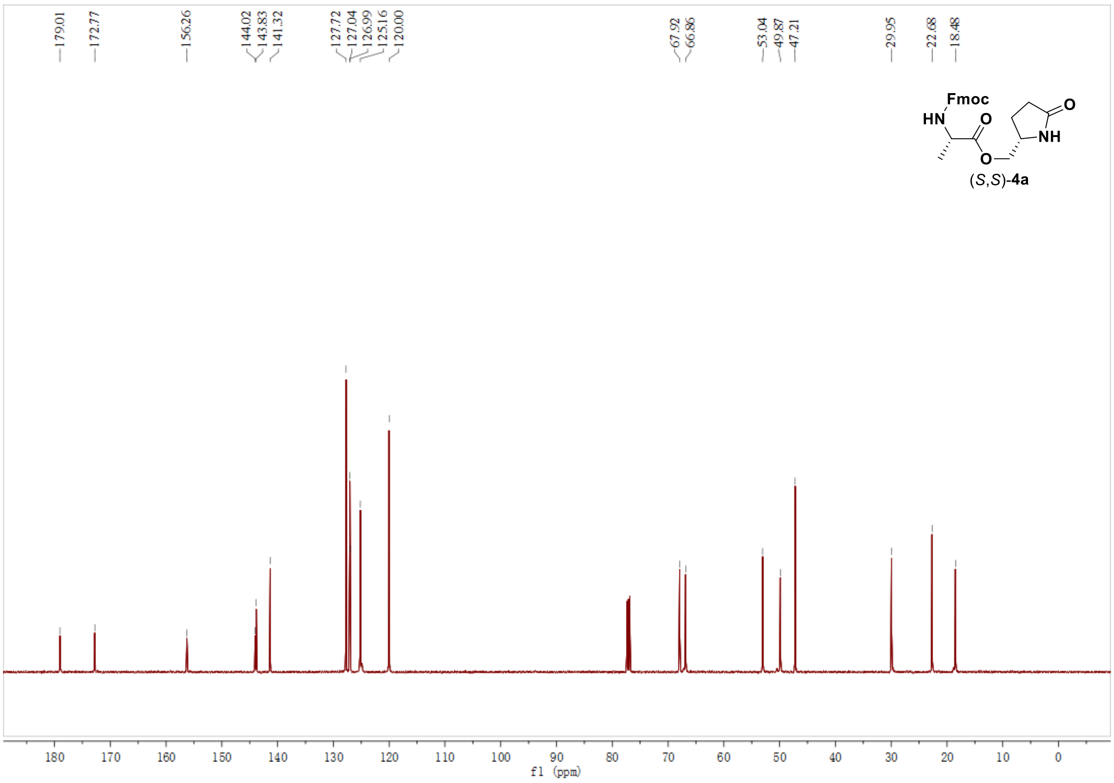

500 MHz, $CDCl_3$, ^{1}H NMR

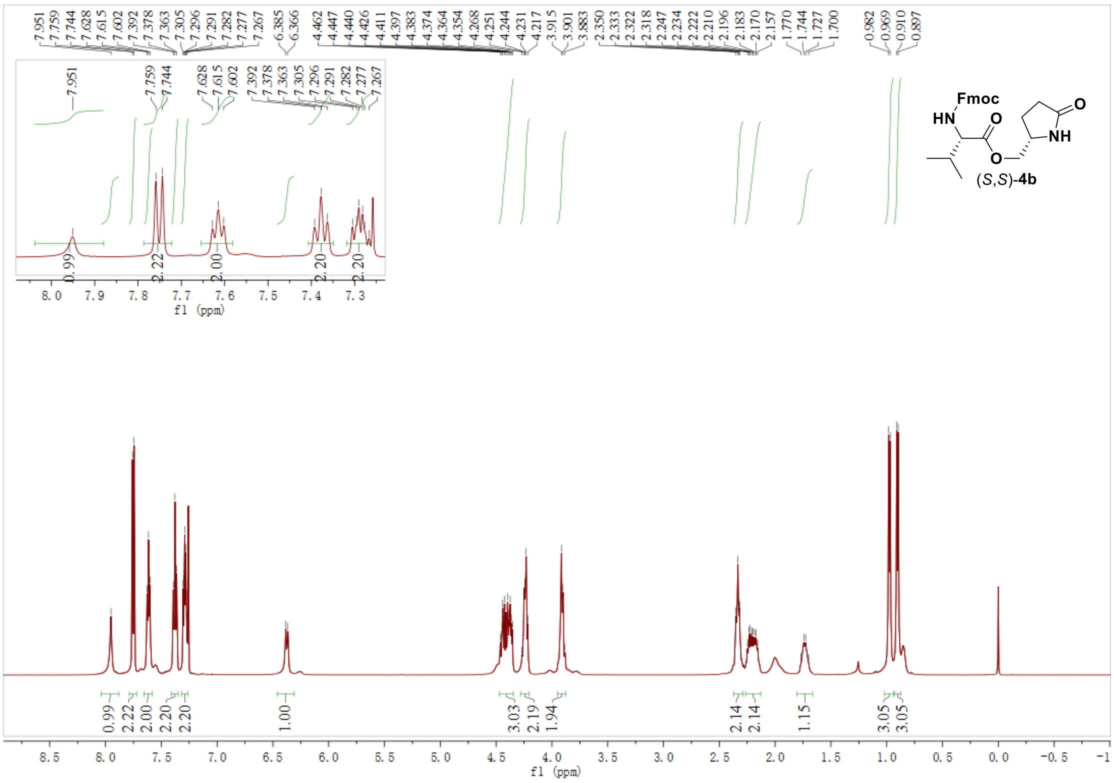

125 MHz, $CDCl_3$, ^{13}C NMR

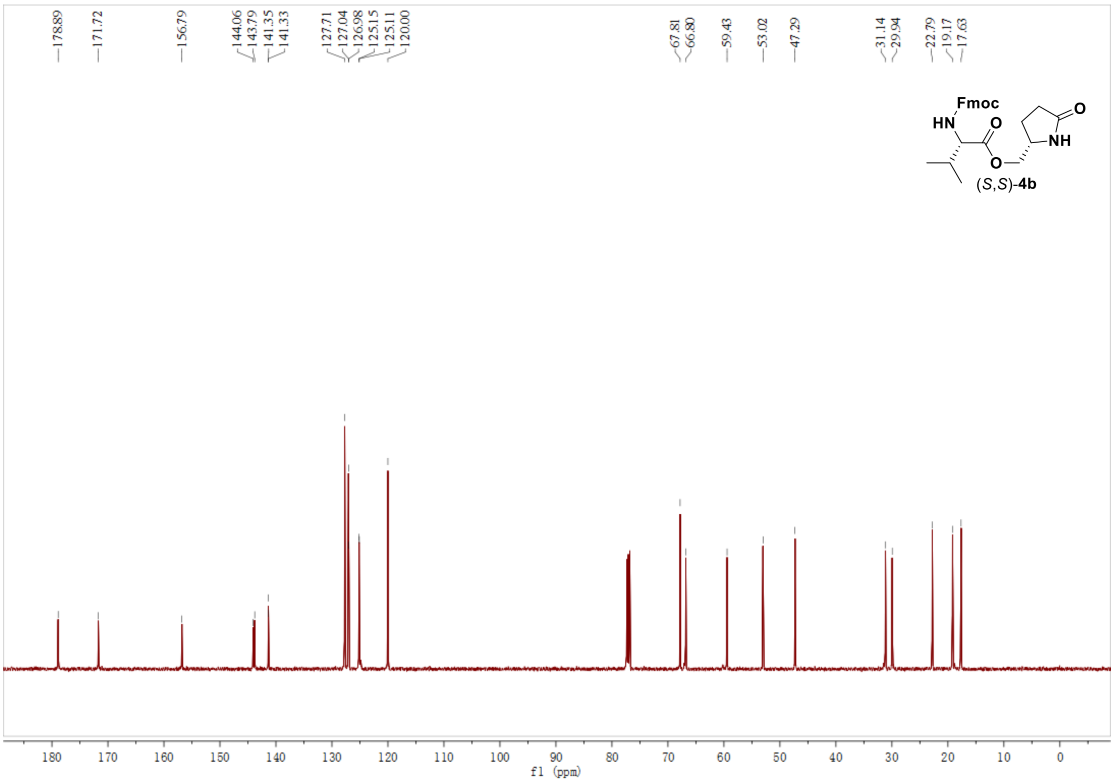

500 MHz, $CDCl_3$, ^1H NMR

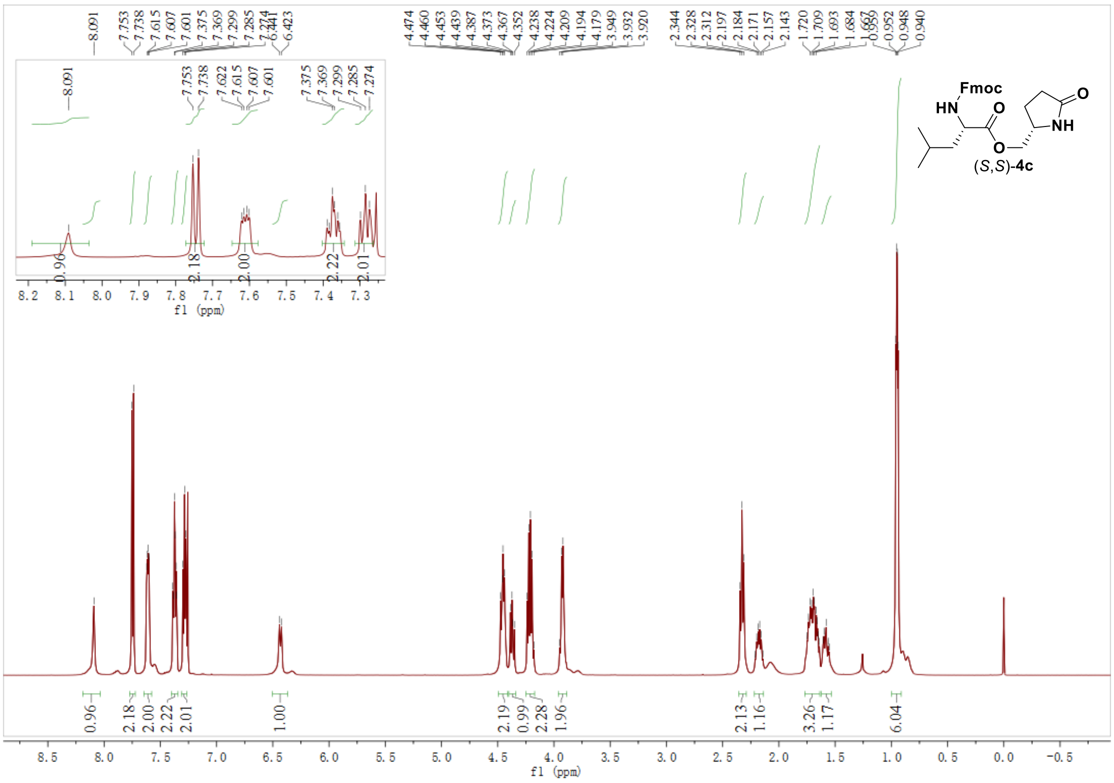

125 MHz, $CDCl_3$, ^{13}C NMR

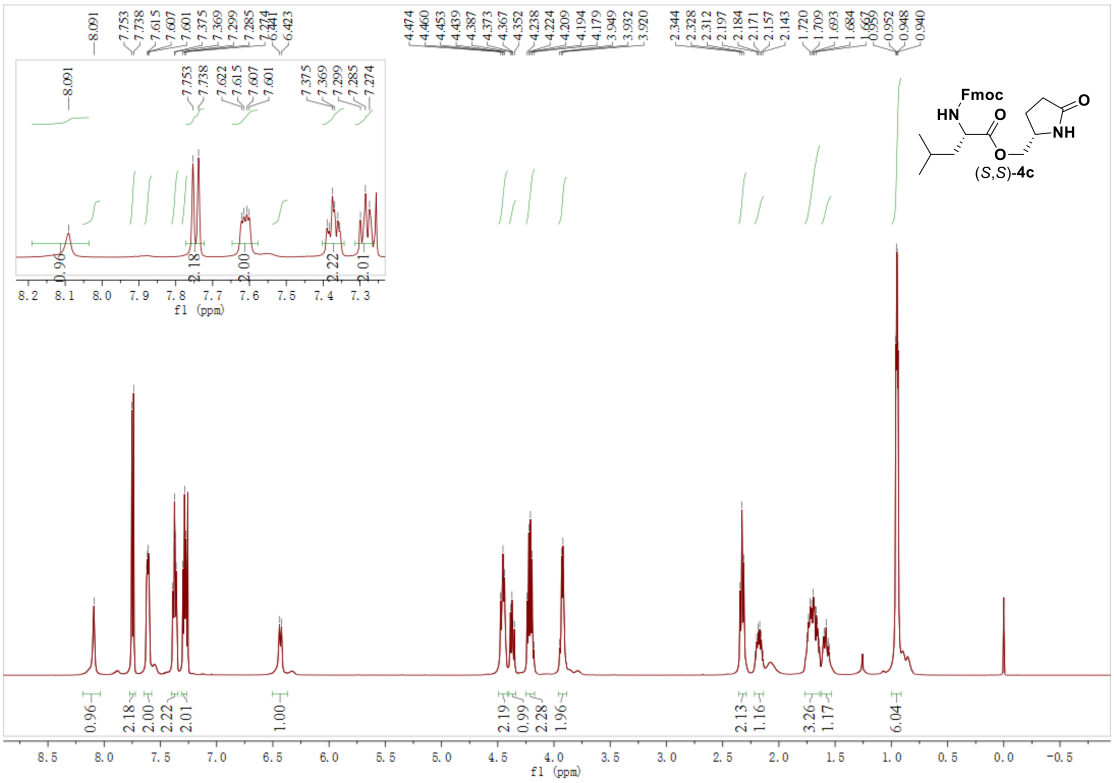

500 MHz, $CDCl_3$, ^1H NMR

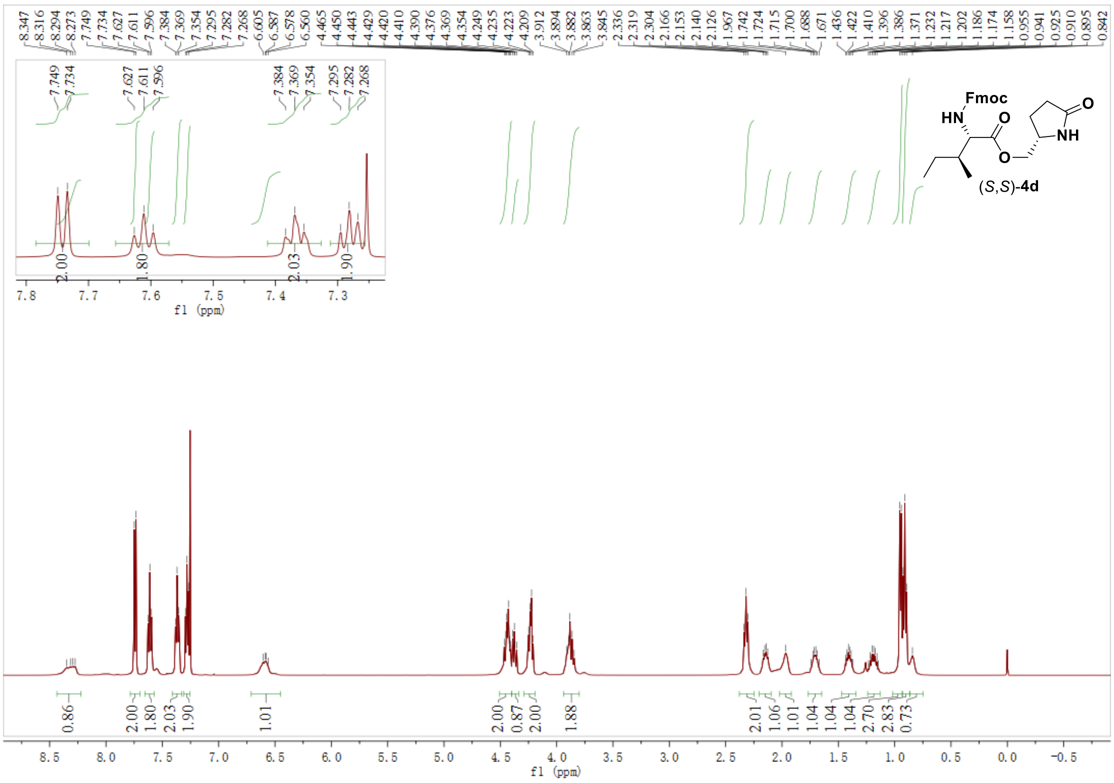

125 MHz, $CDCl_3$, ^{13}C NMR

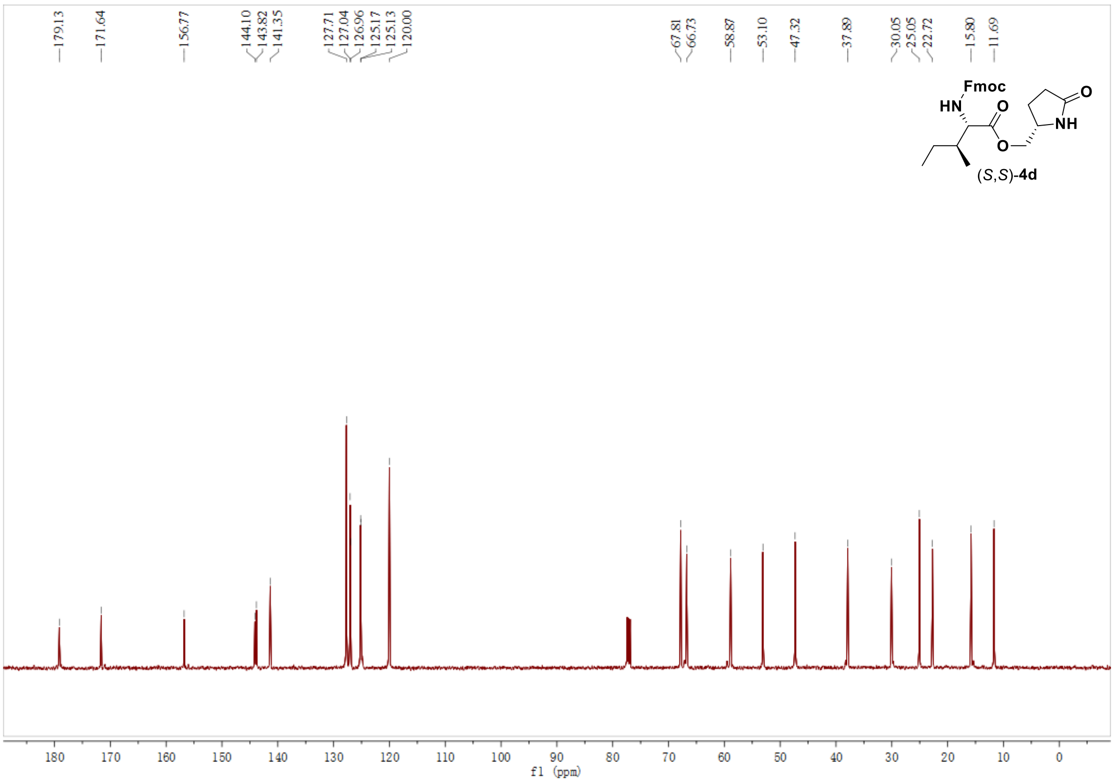

500 MHz, DMSO-d_6, ^{1}H NMR

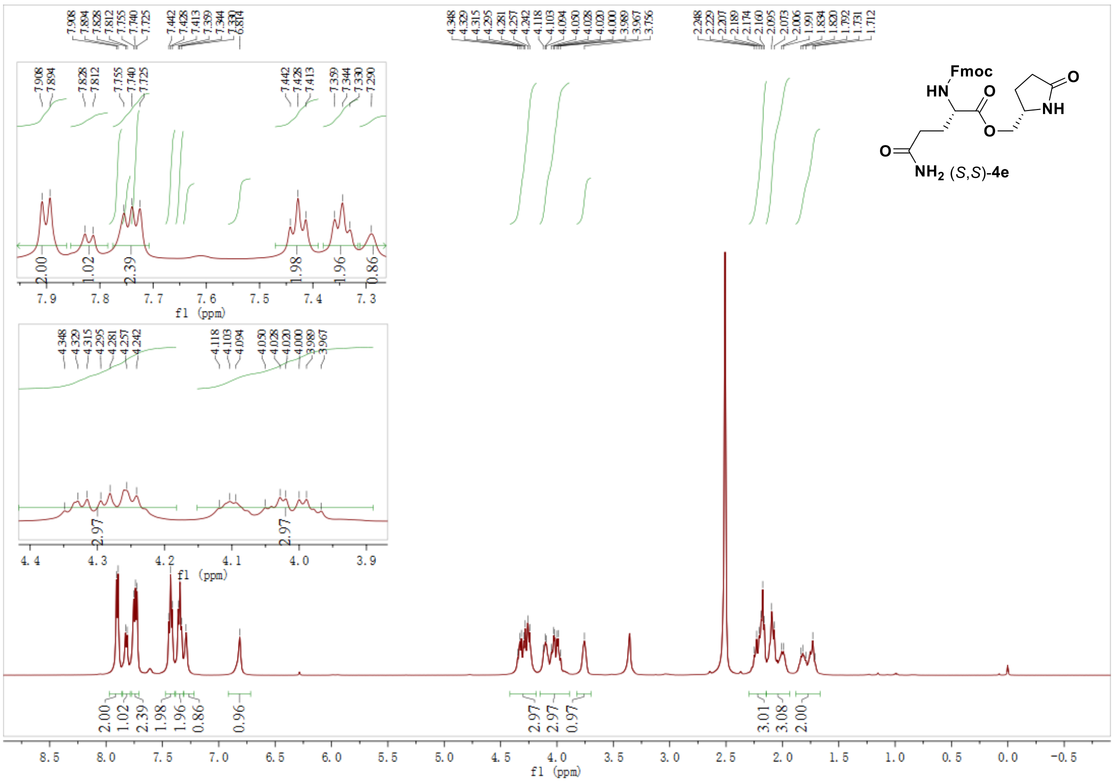

125 MHz, DMSO-d_6, ^{13}C NMR

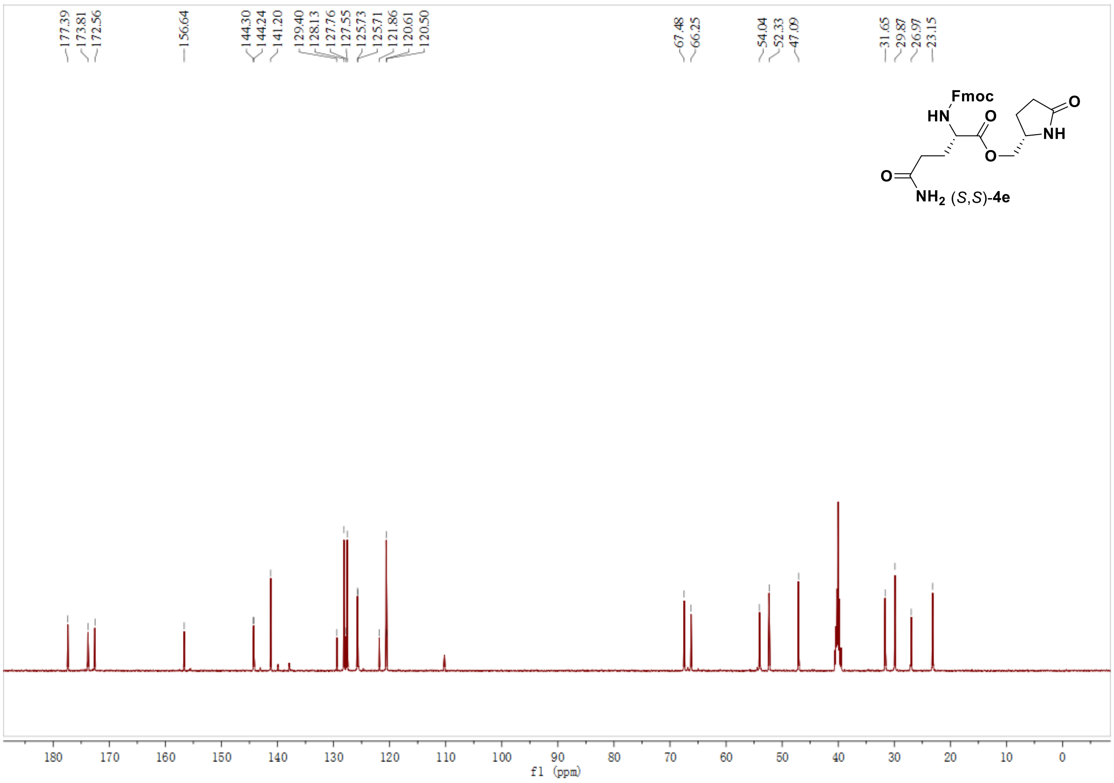

500 MHz, $CDCl_3$, ^1H NMR

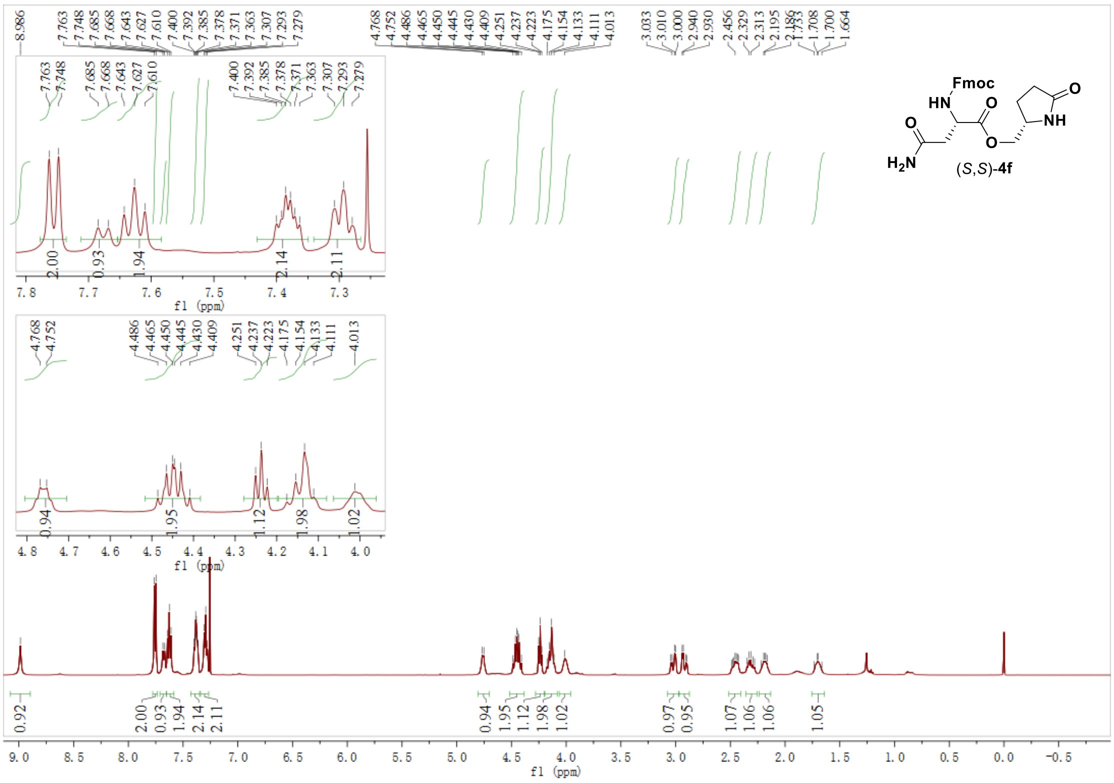

125 MHz, $CDCl_3$, ^{13}C NMR

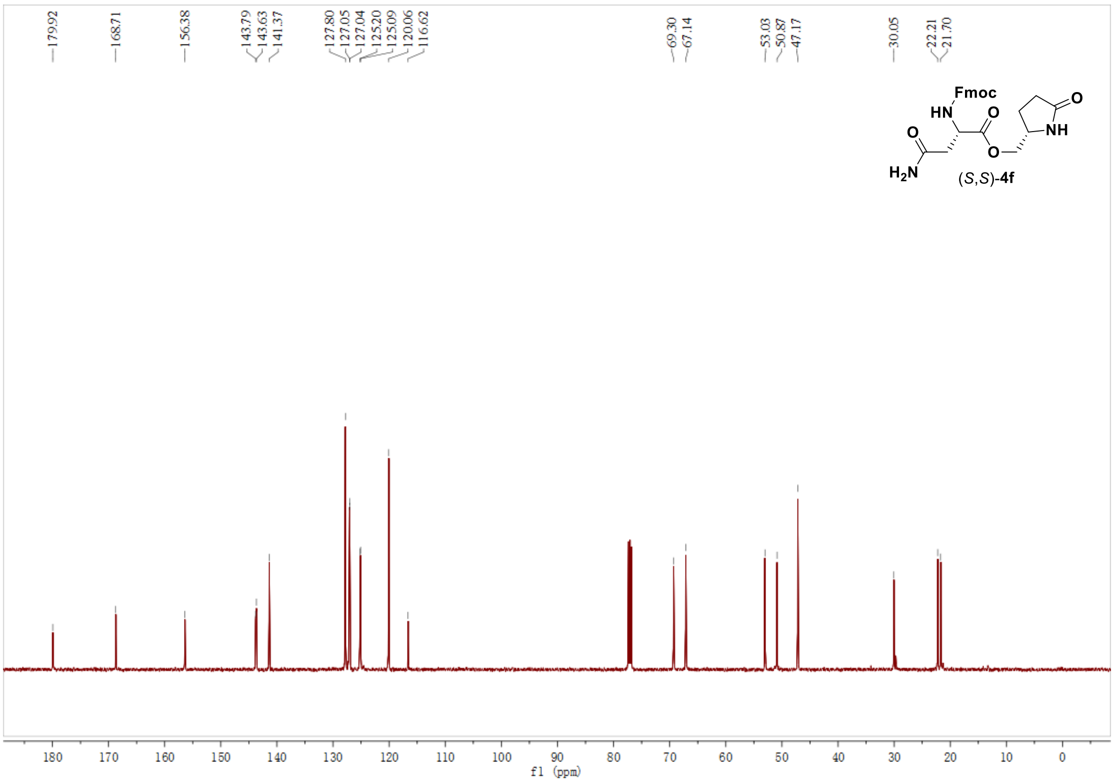

500 MHz, DMSO-d_6, ^{1}H NMR

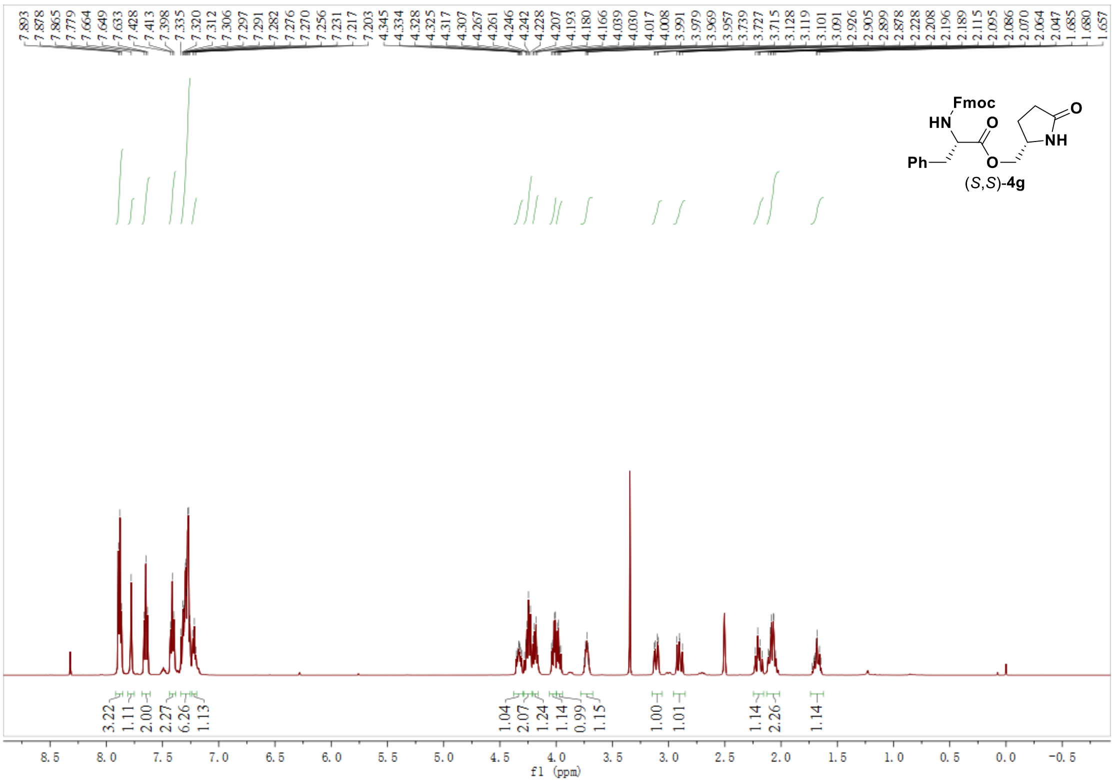

125 MHz, DMSO-d_6, ^{13}C NMR

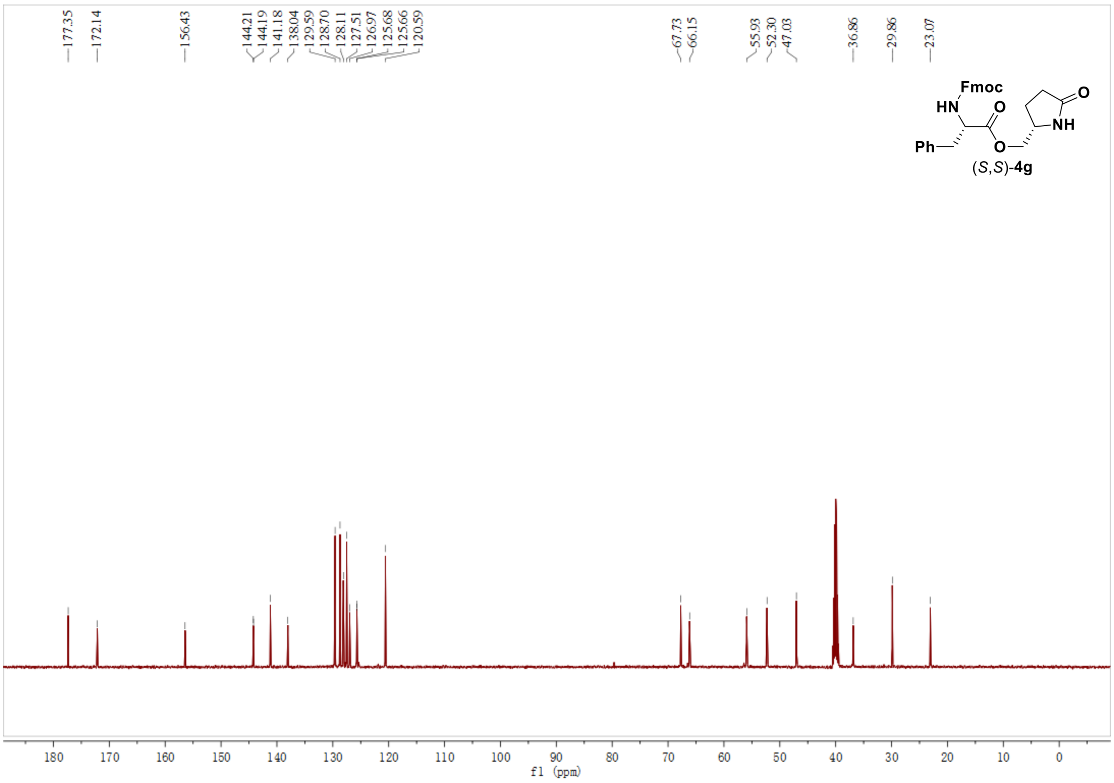

500 MHz, $CDCl_3$, ^{1}H NMR

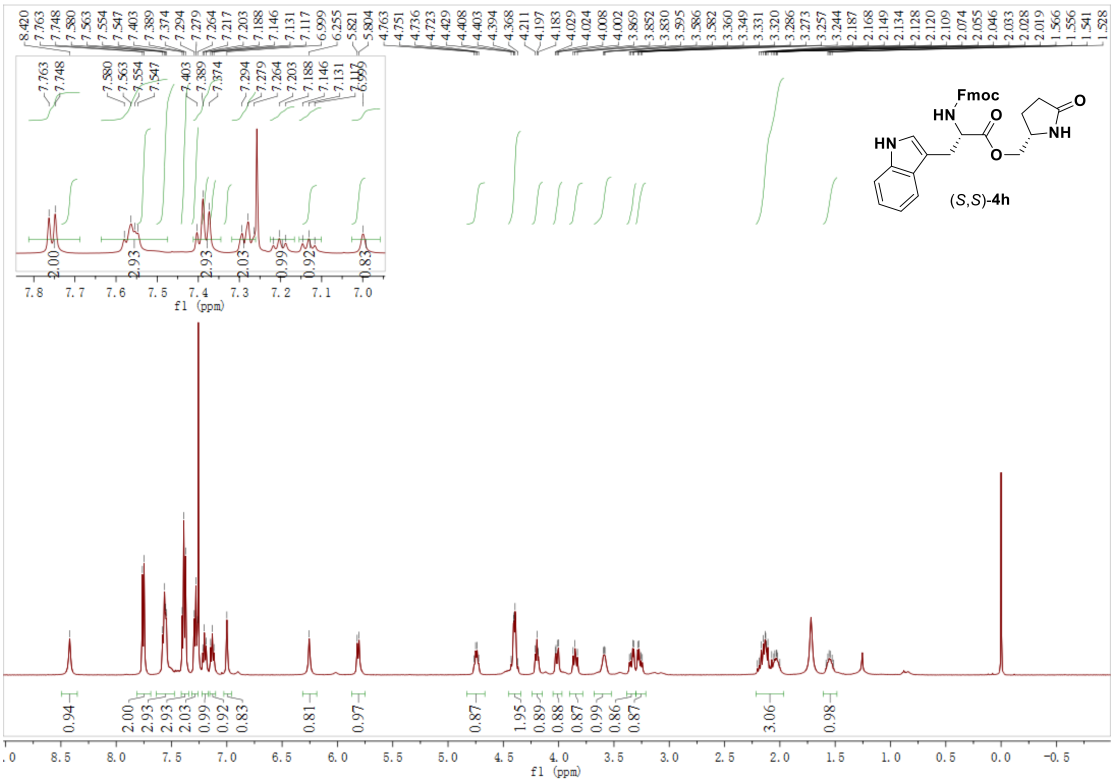

125 MHz, $CDCl_3$, ^{13}C NMR

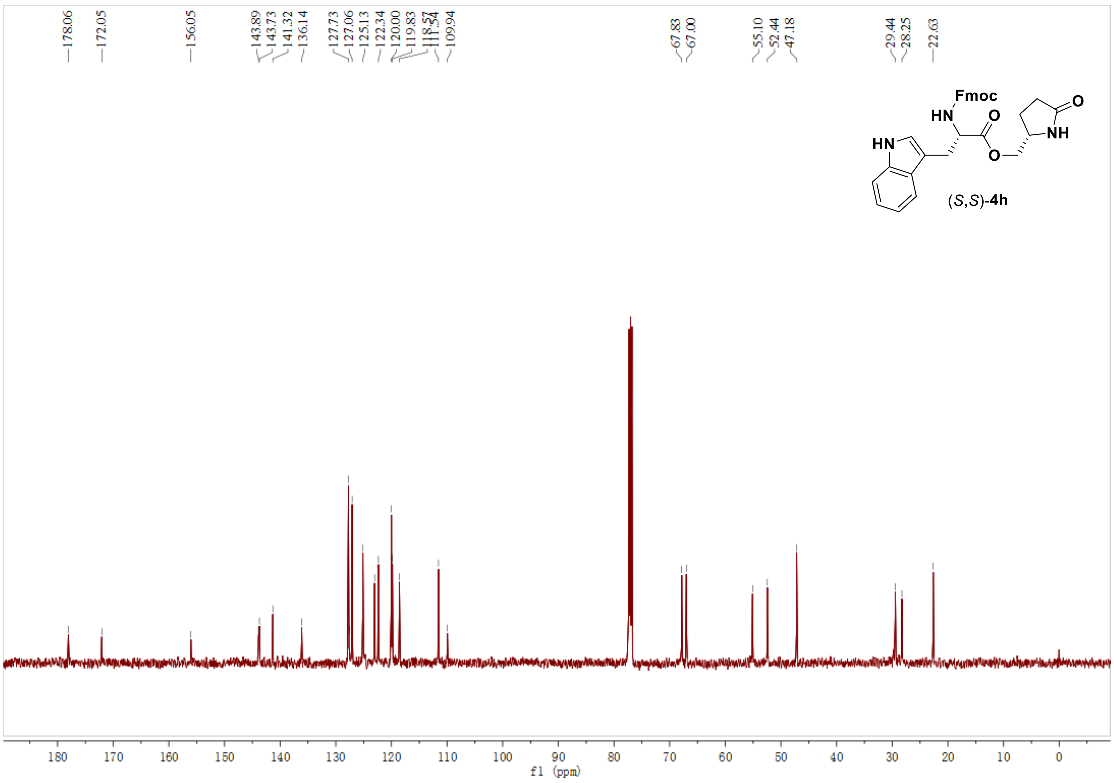

500 MHz, $CDCl_3$, ^{1}H NMR

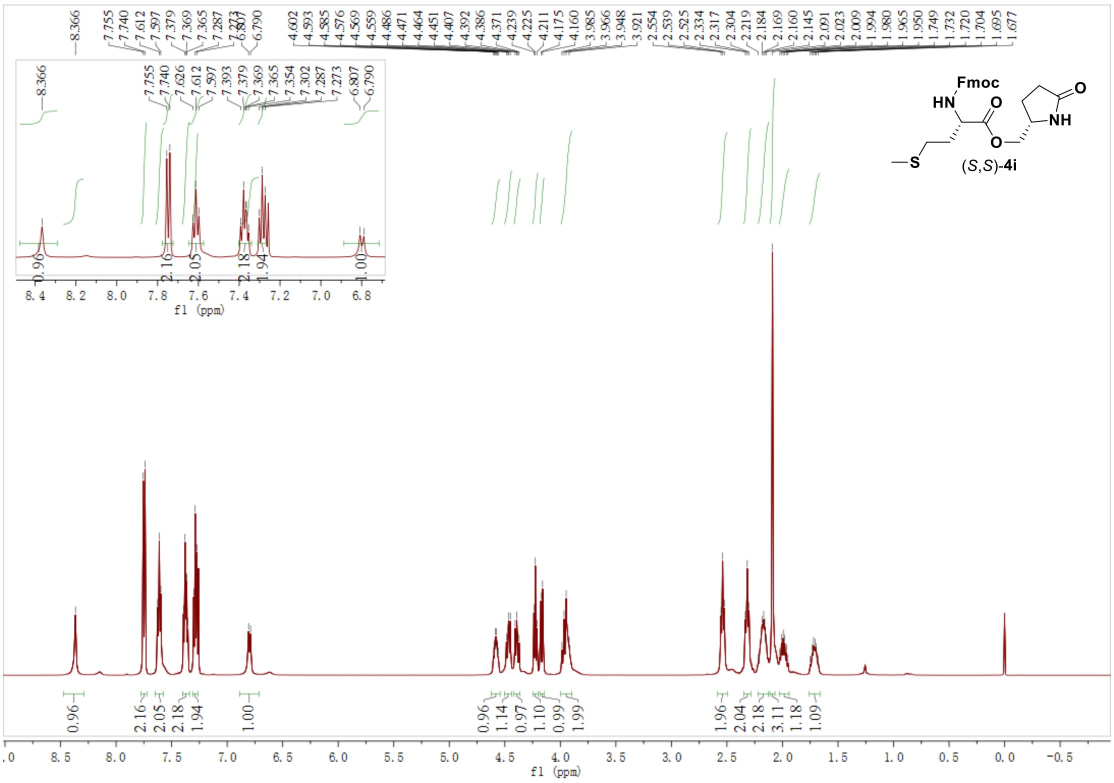

125 MHz, $CDCl_3$, ^{13}C NMR

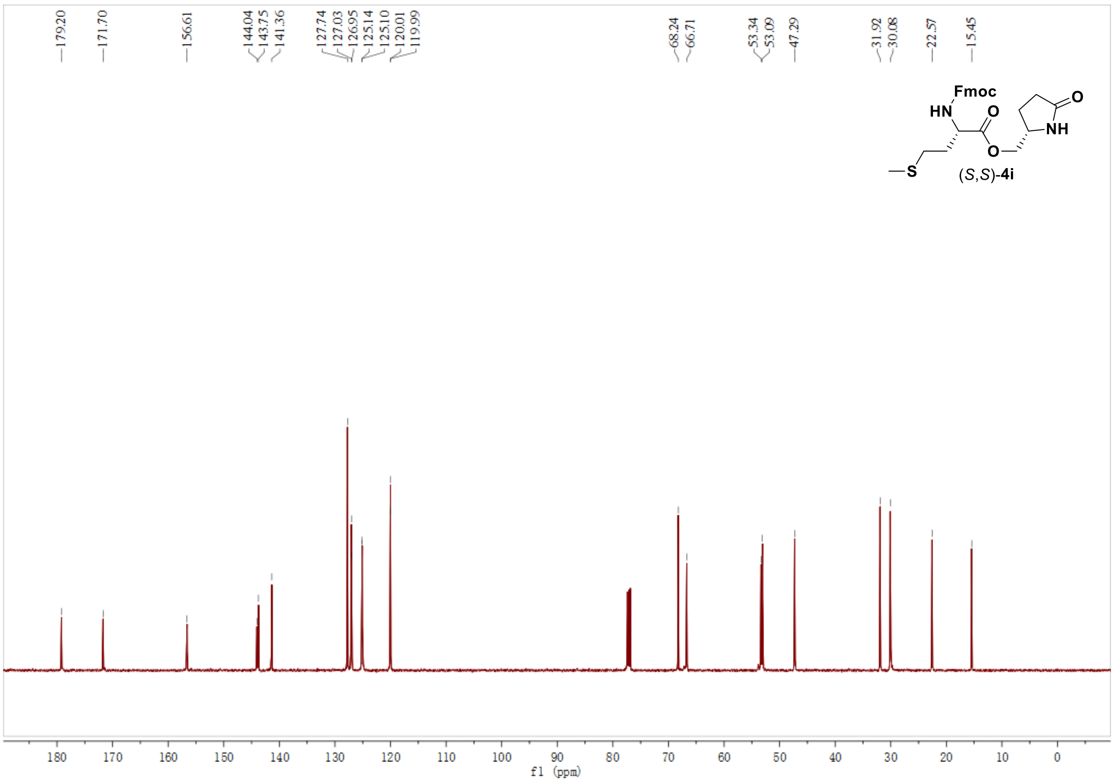

500 MHz, $CDCl_3$, ^1H NMR

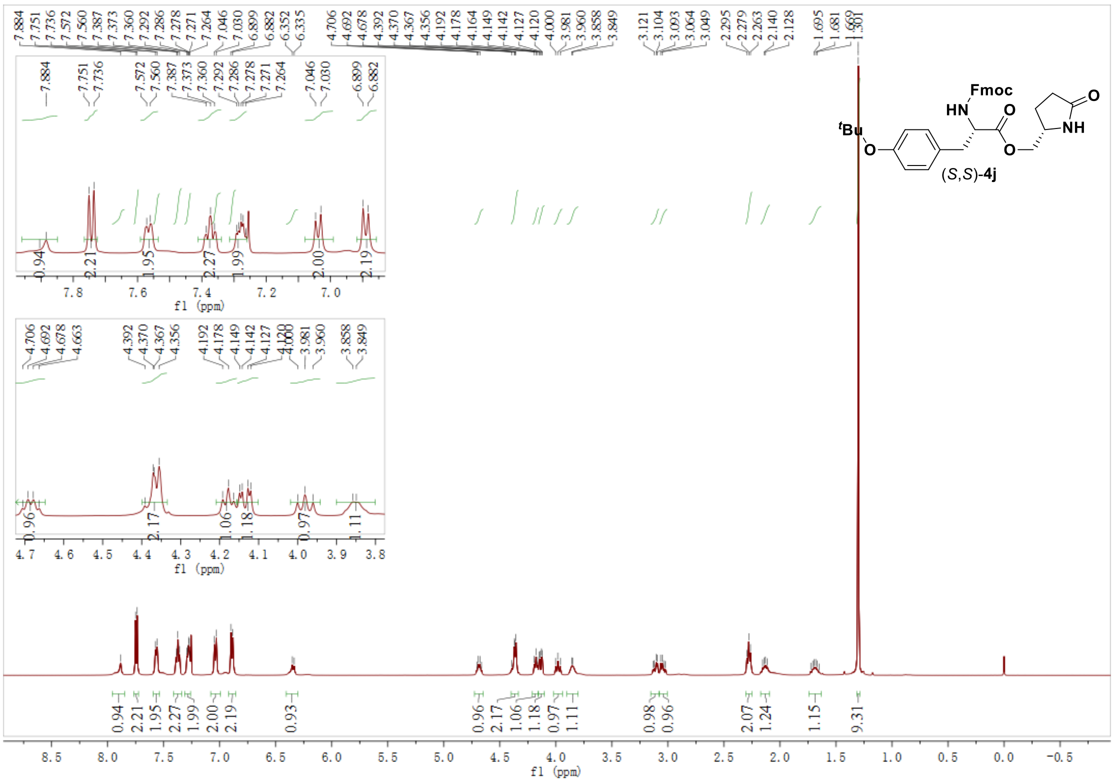

125 MHz, $CDCl_3$, ^{13}C NMR

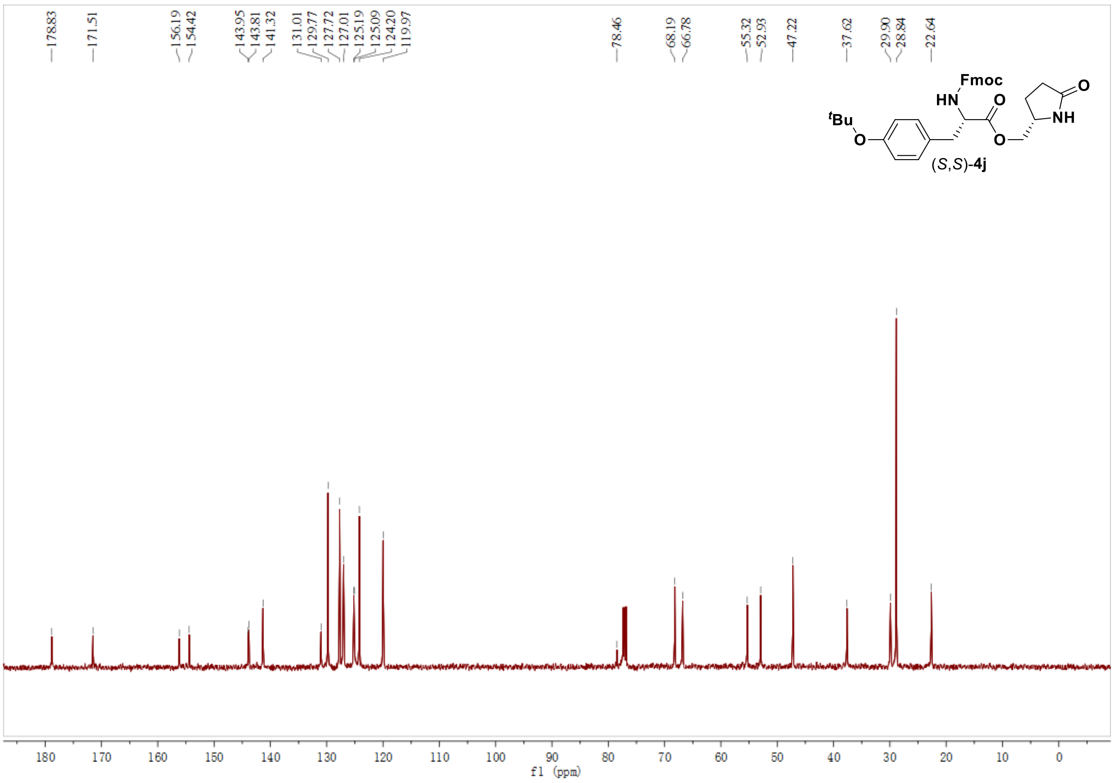

500 MHz, $CDCl_3$, ^{1}H NMR

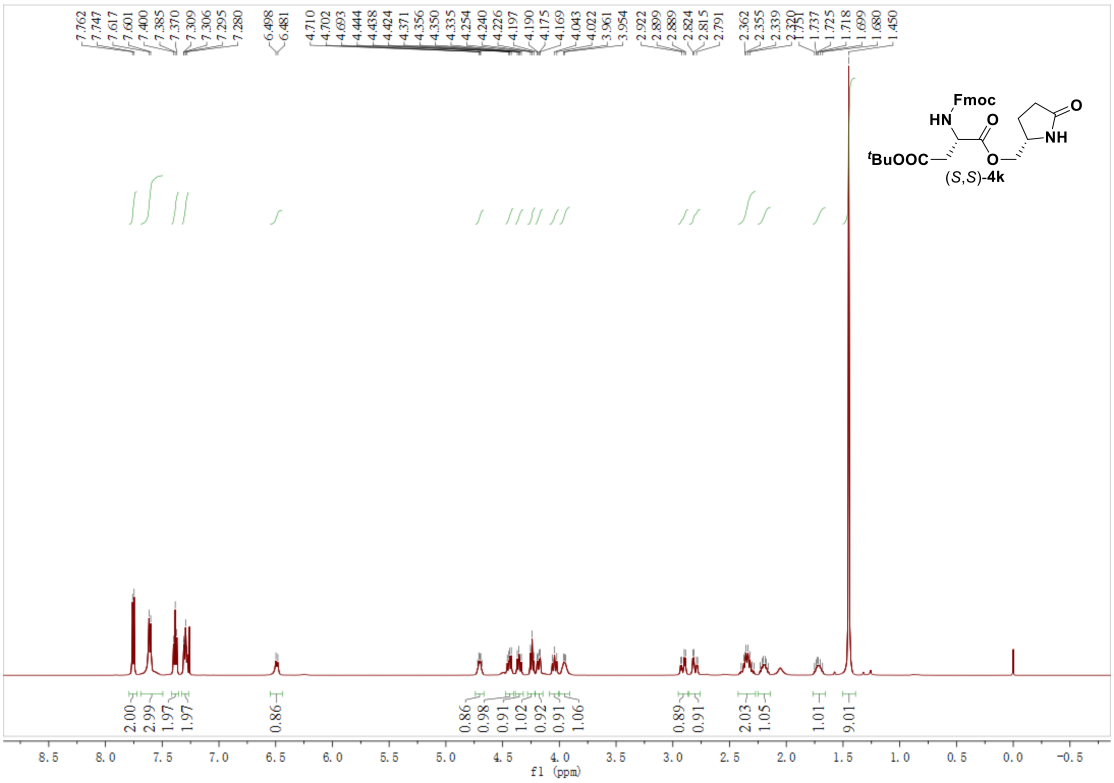

125 MHz, $CDCl_3$, ^{13}C NMR

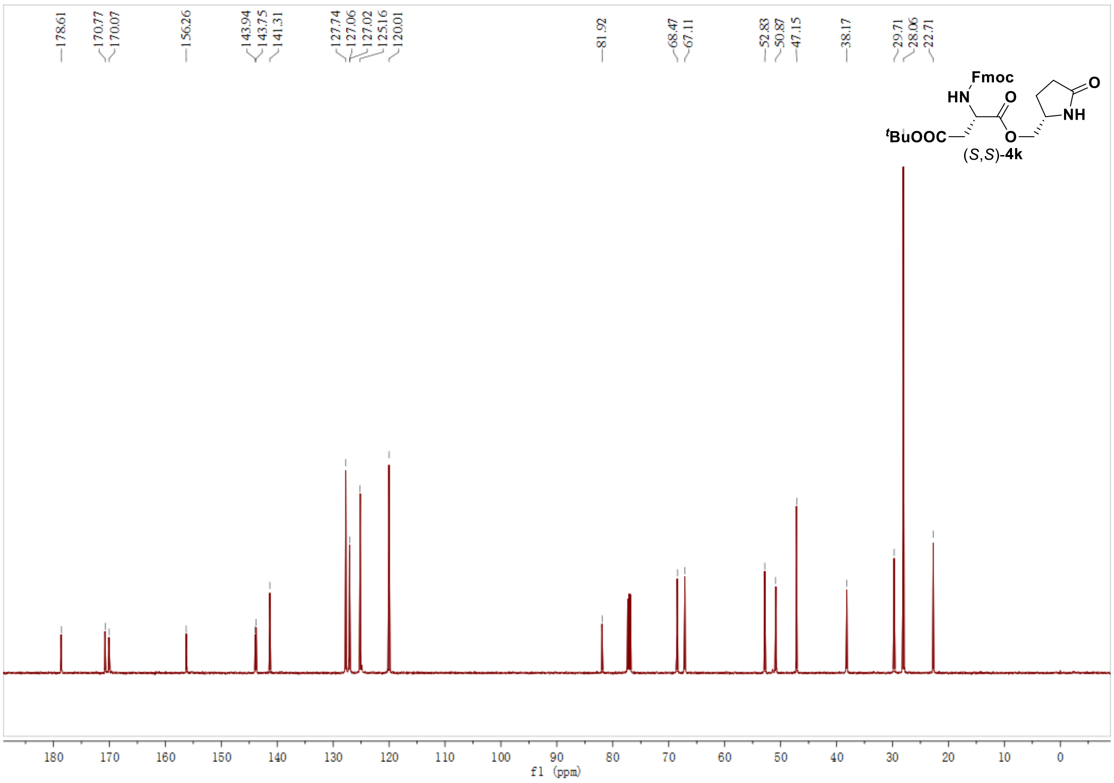

500 MHz, $CDCl_3$, ^1H NMR

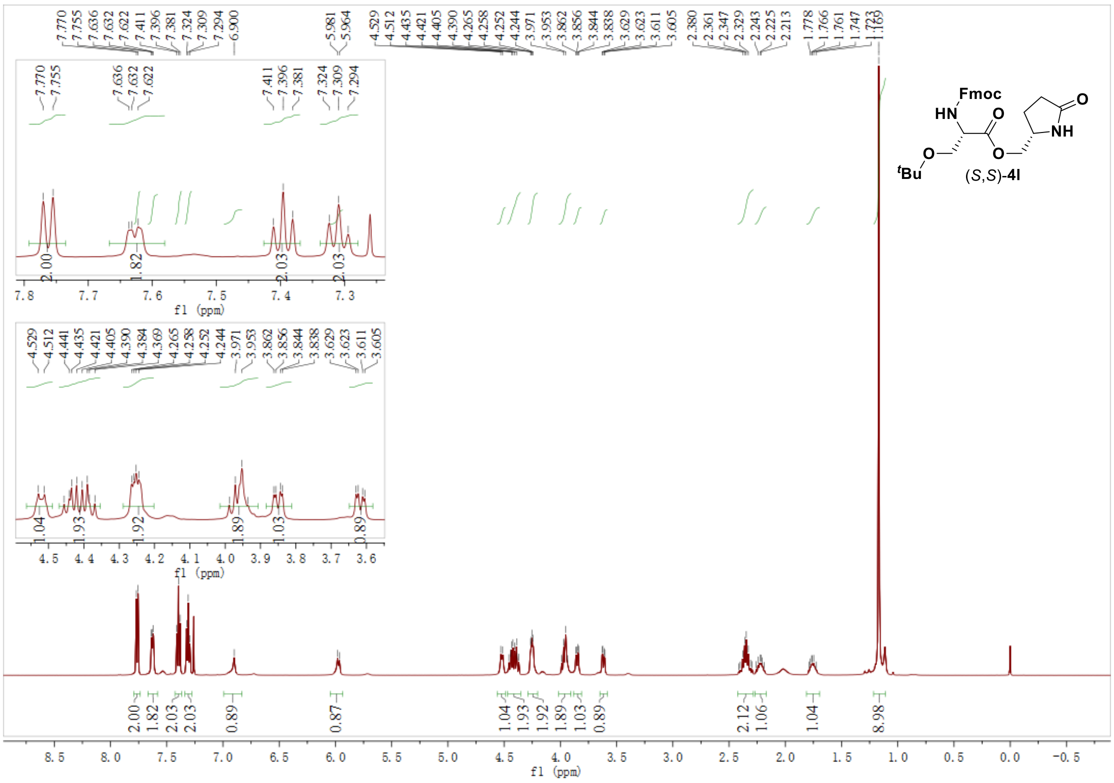

125 MHz, $CDCl_3$, ^{13}C NMR

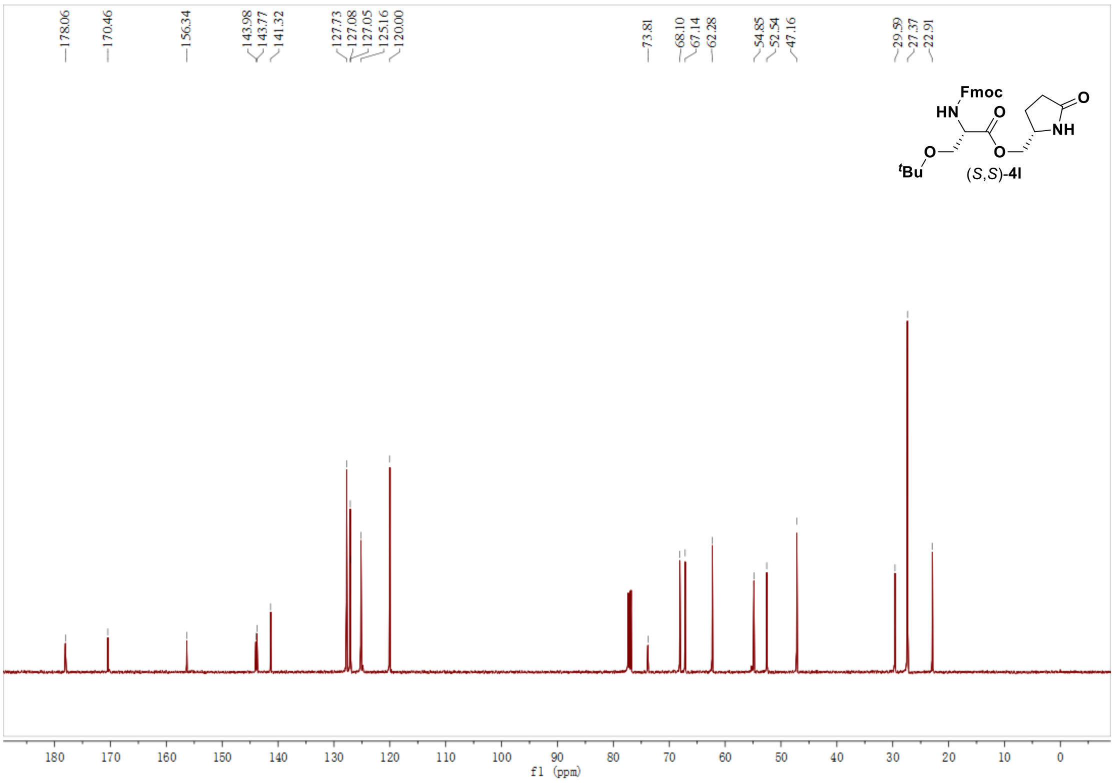

500 MHz, $CDCl_3$, ^1H NMR

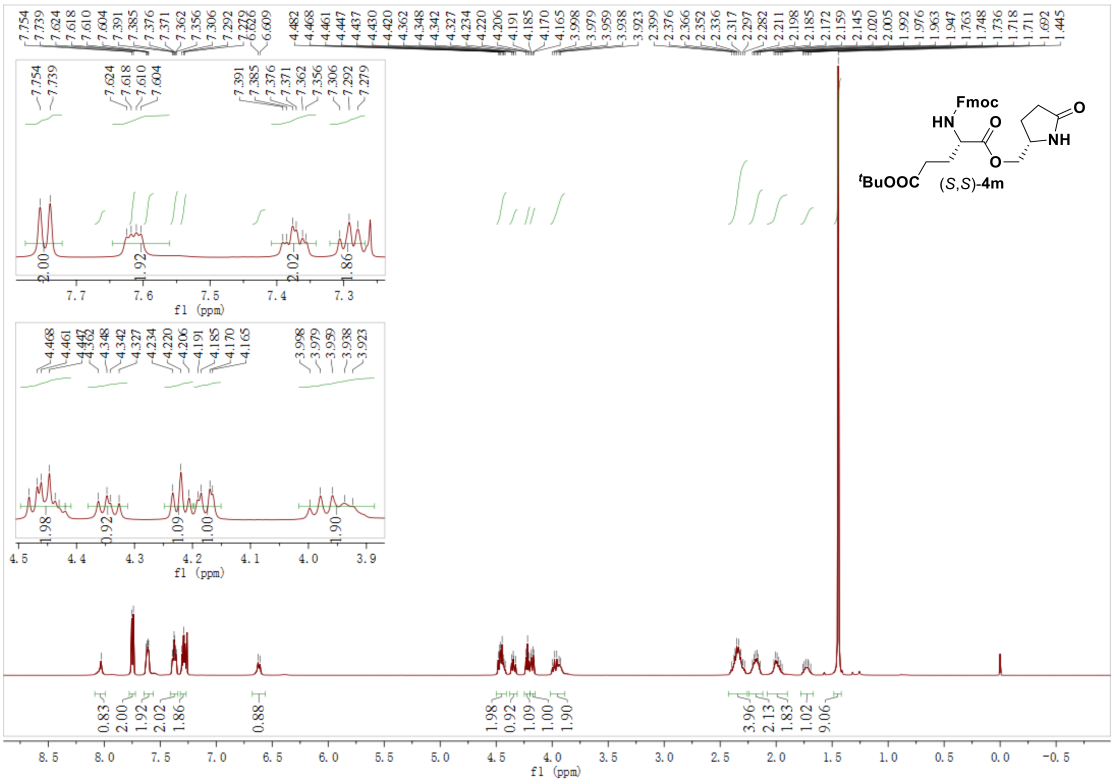

125 MHz, $CDCl_3$, ^{13}C NMR

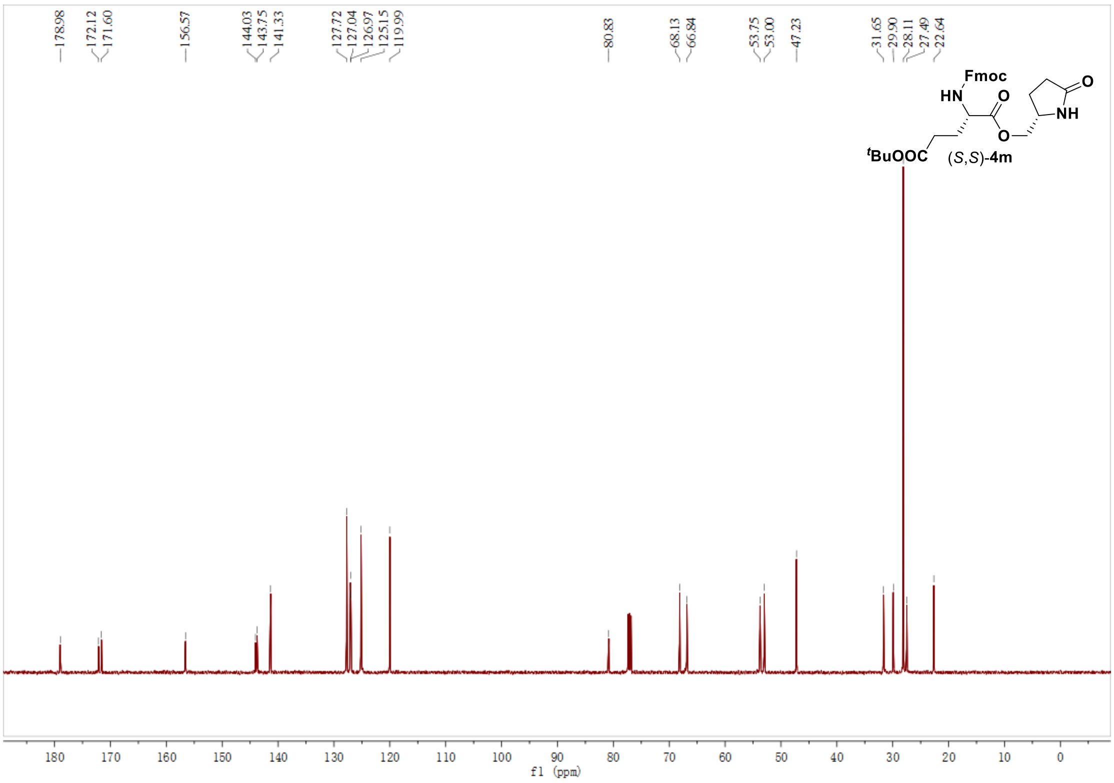

500 MHz, DMSO-d_6, ^{1}H NMR

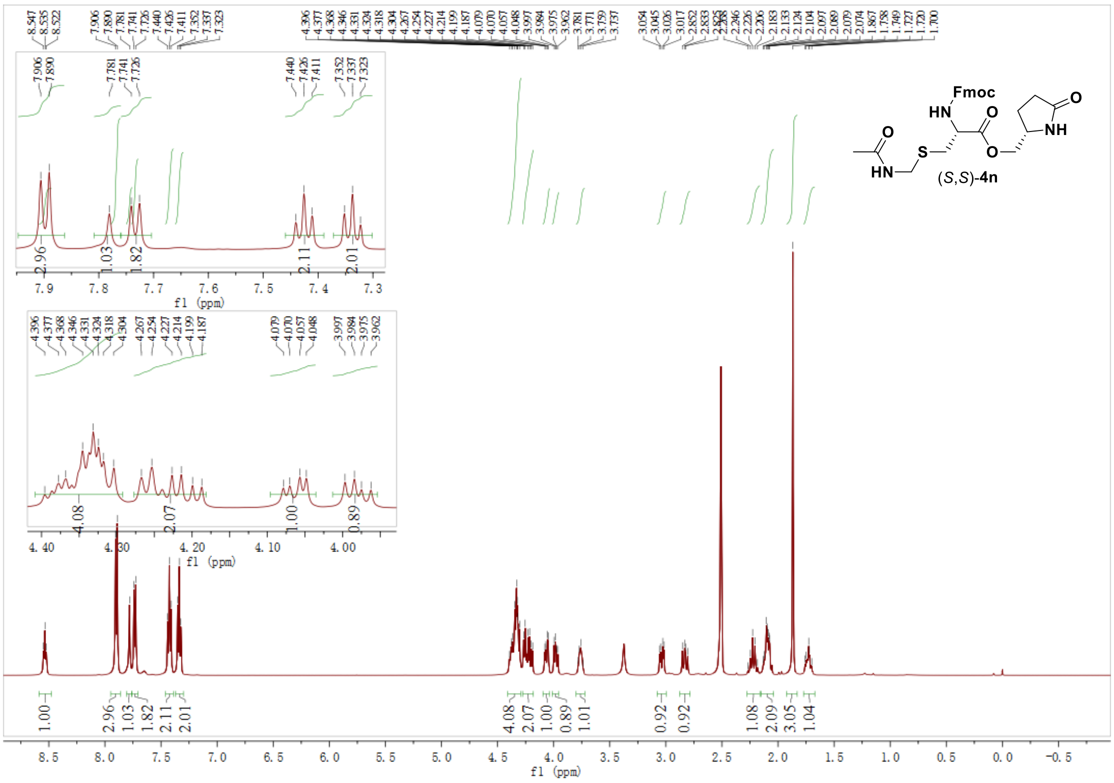

125 MHz, DMSO-d_6, ^{13}C NMR

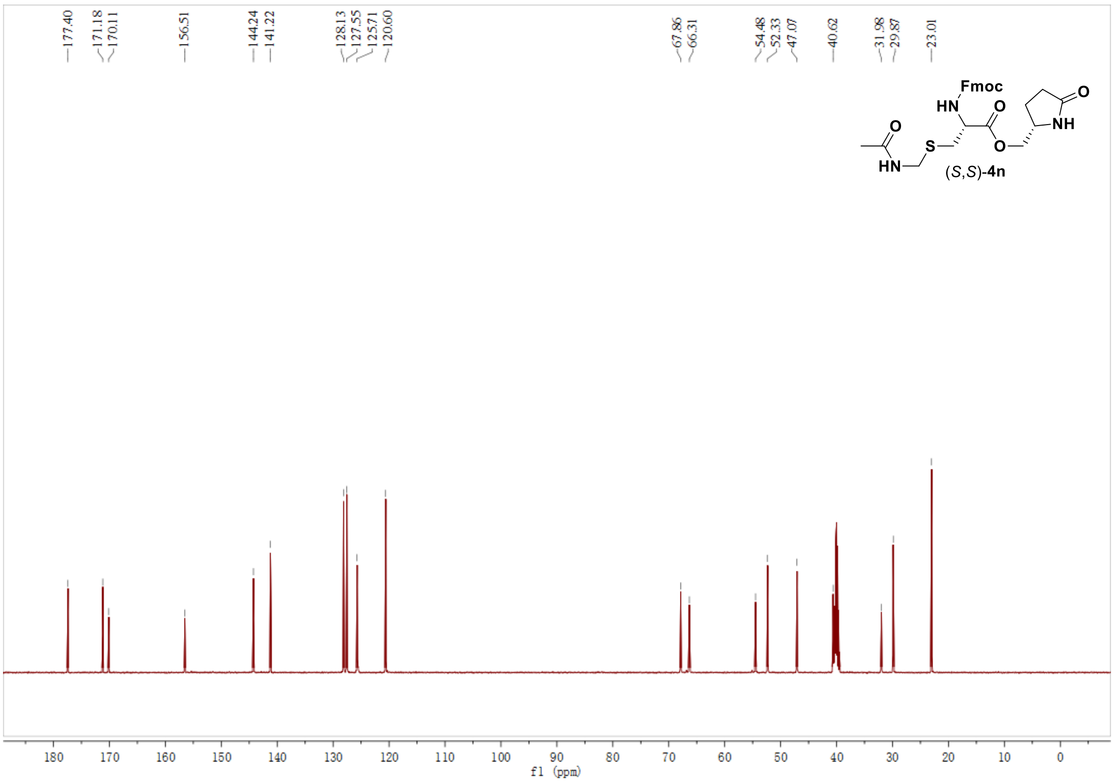

500 MHz, $CDCl_3$, ^1H NMR

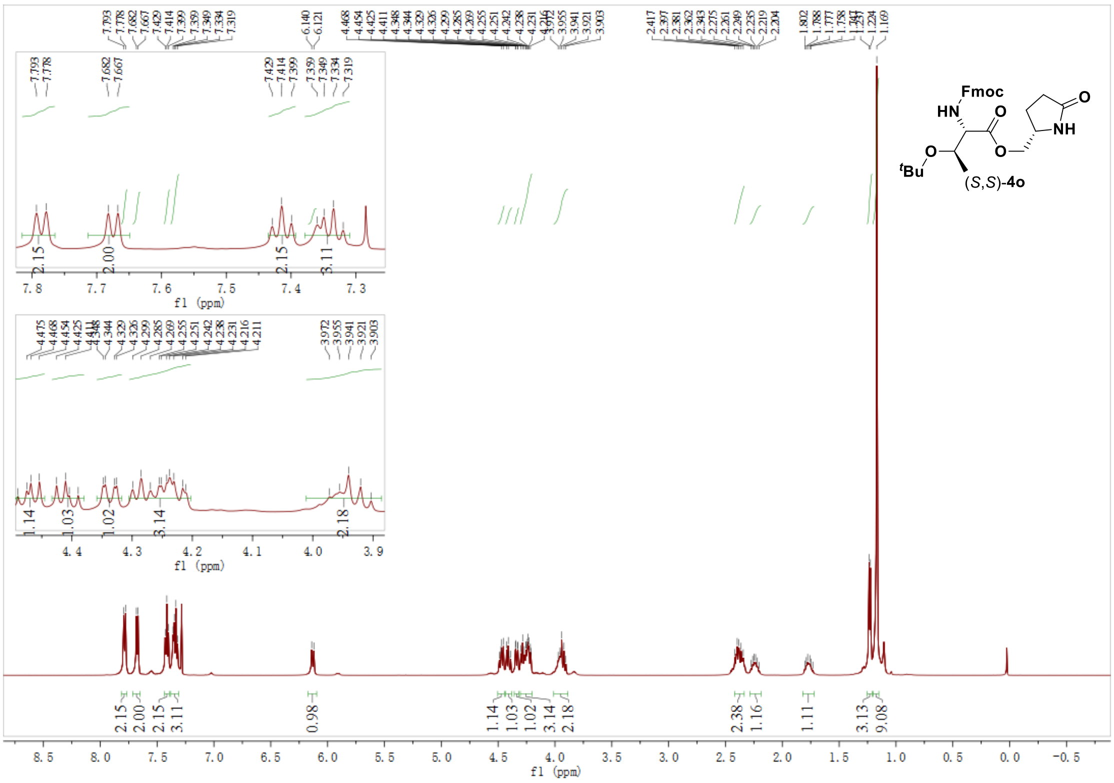

125 MHz, $CDCl_3$, ^{13}C NMR

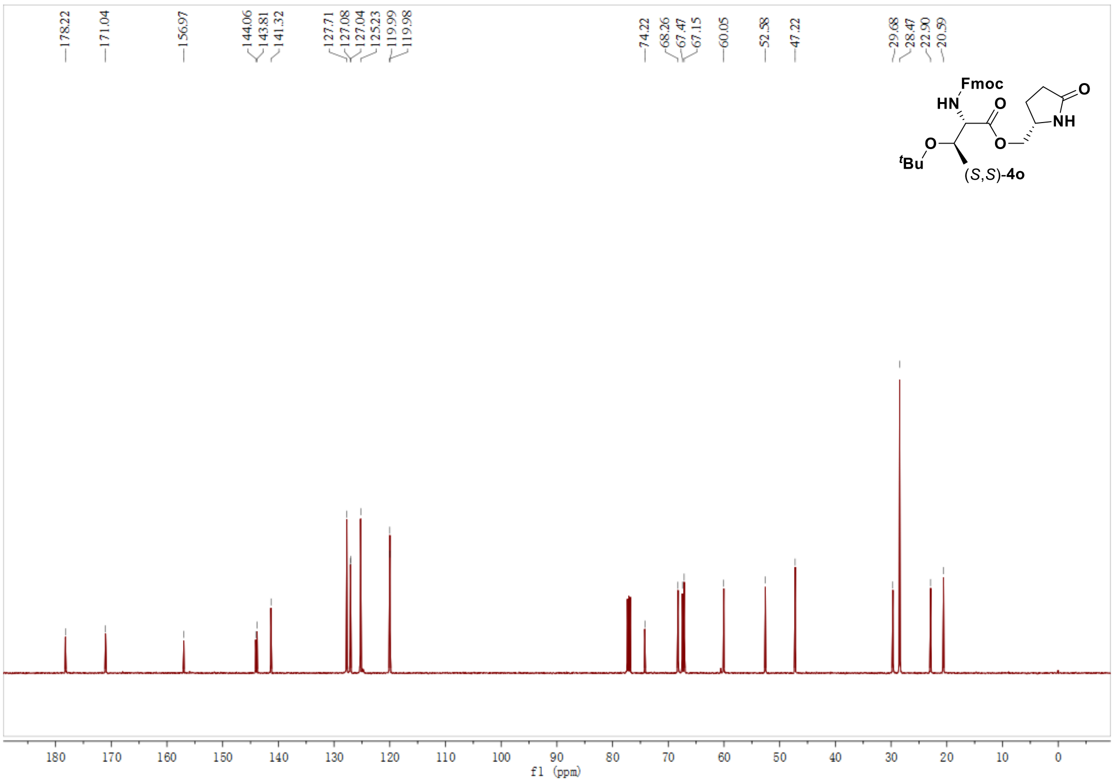

500 MHz, $CDCl_3$, ^{1}H NMR

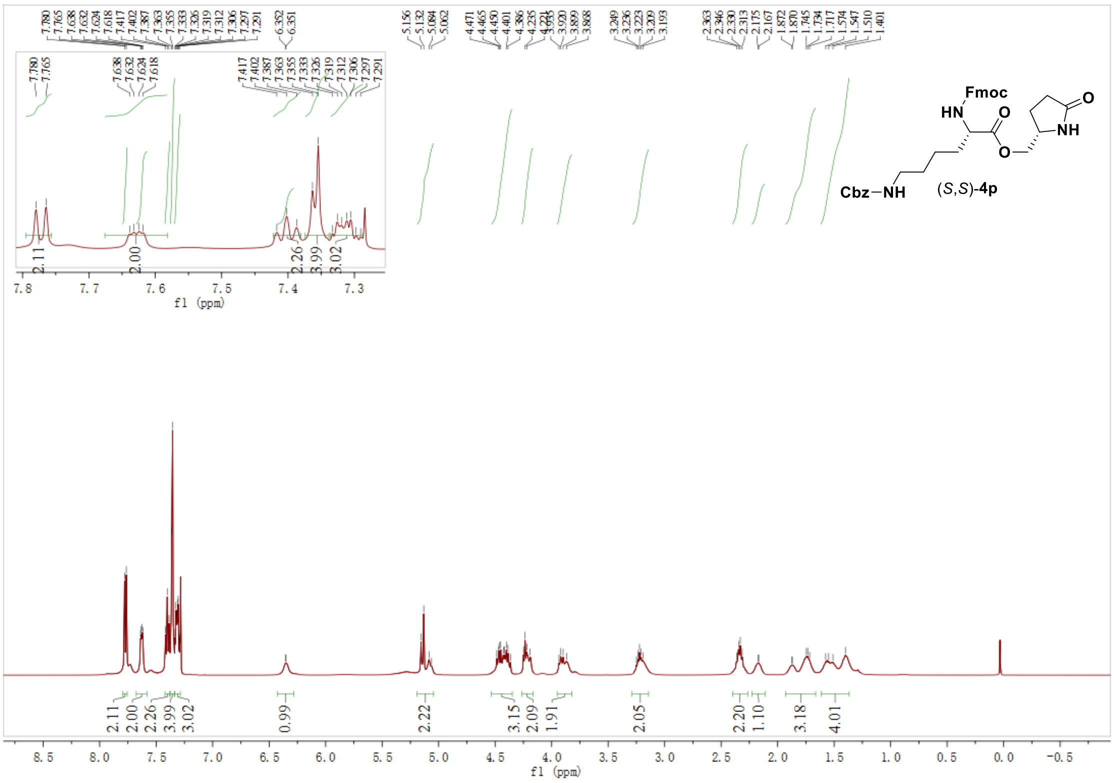

125 MHz, $CDCl_3$, ^{13}C NMR

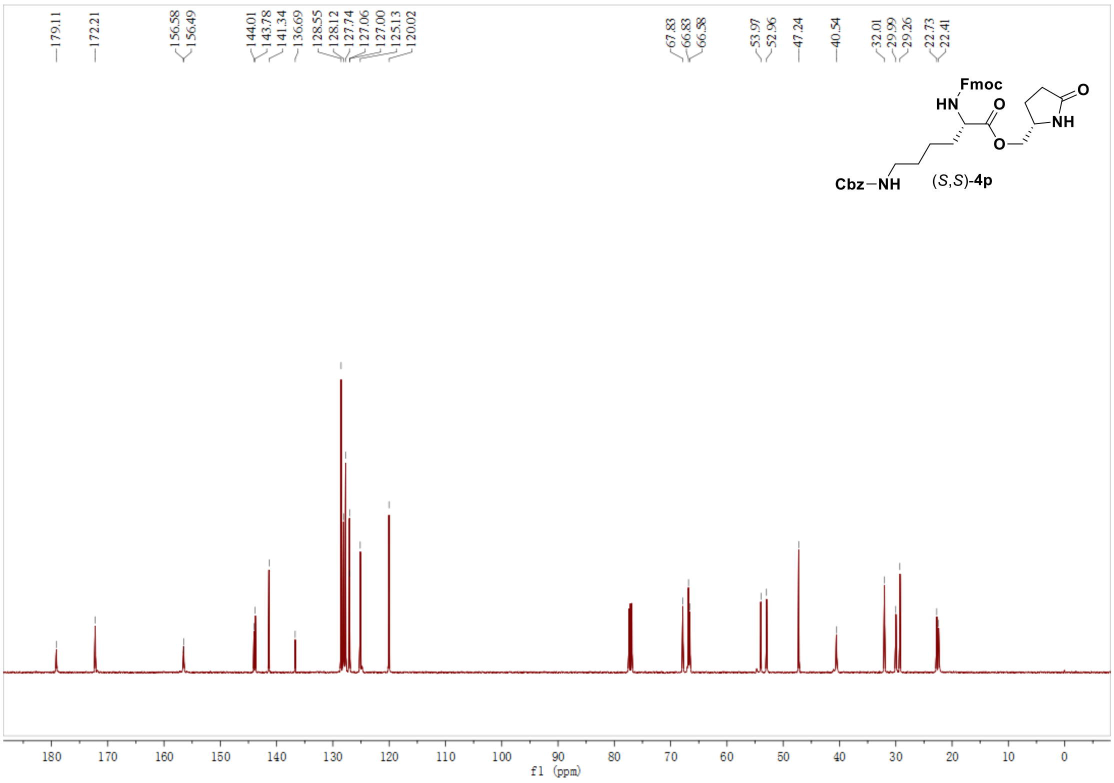

500 MHz, $CDCl_3$, ^1H NMR

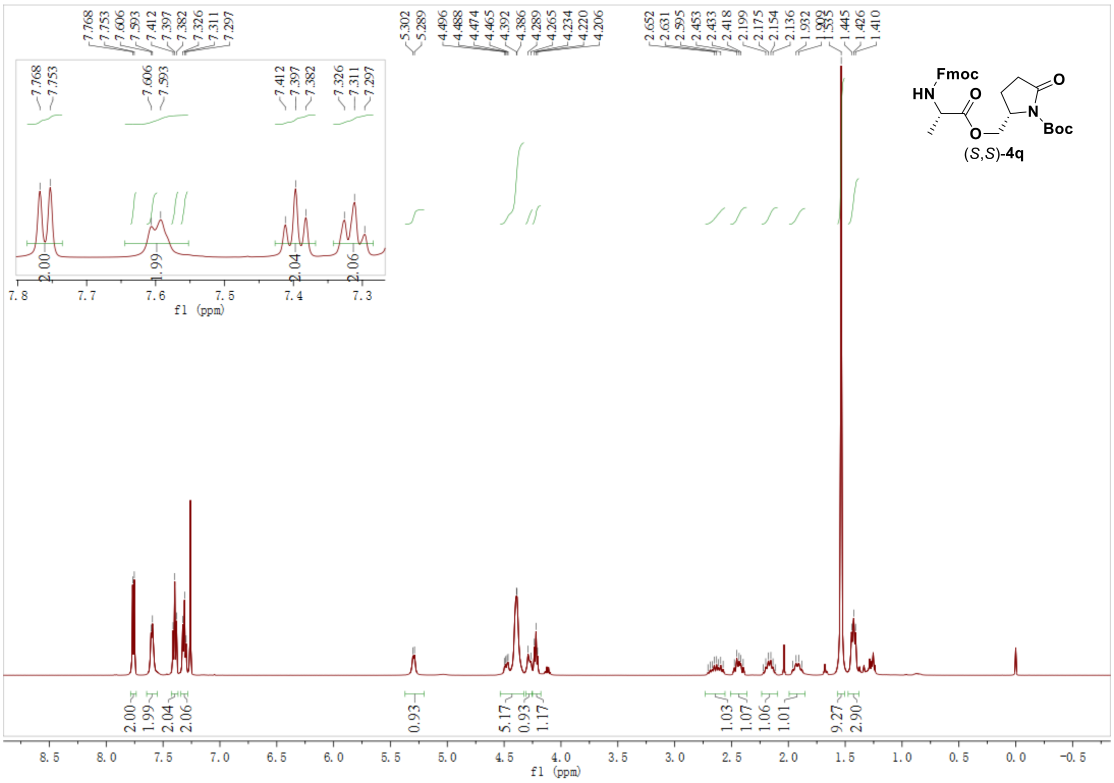

125 MHz, $CDCl_3$, ^{13}C NMR

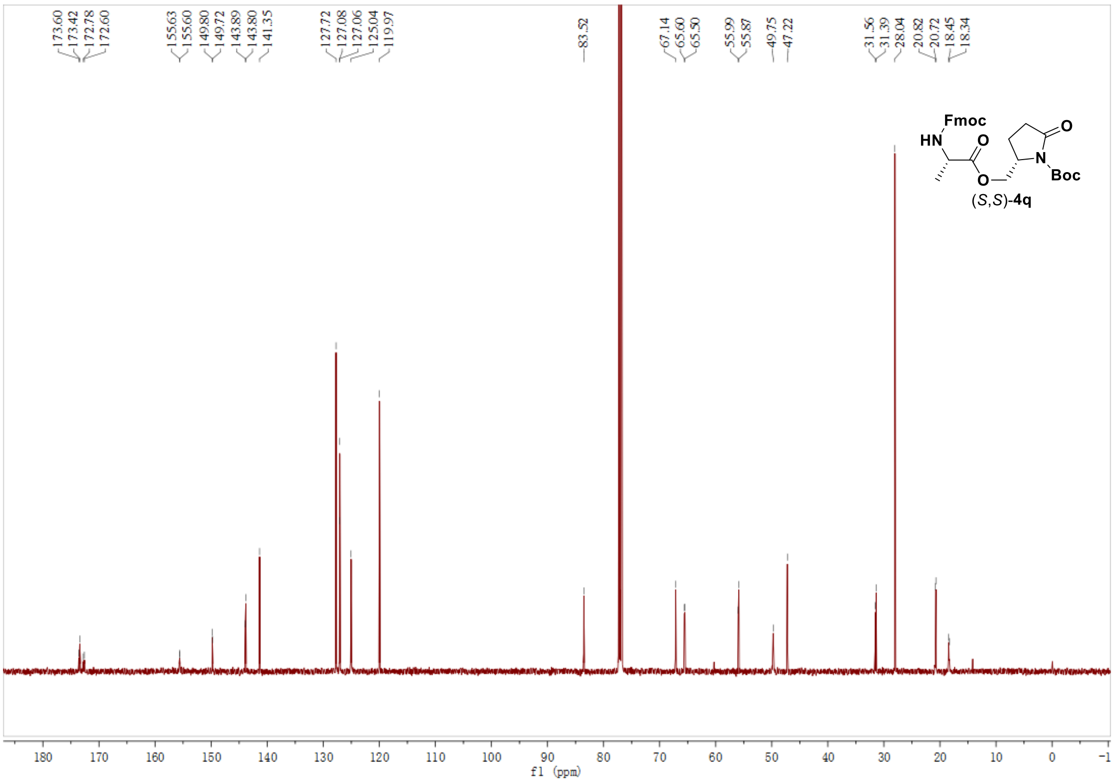

500 MHz, $CDCl_3$, ^1H NMR

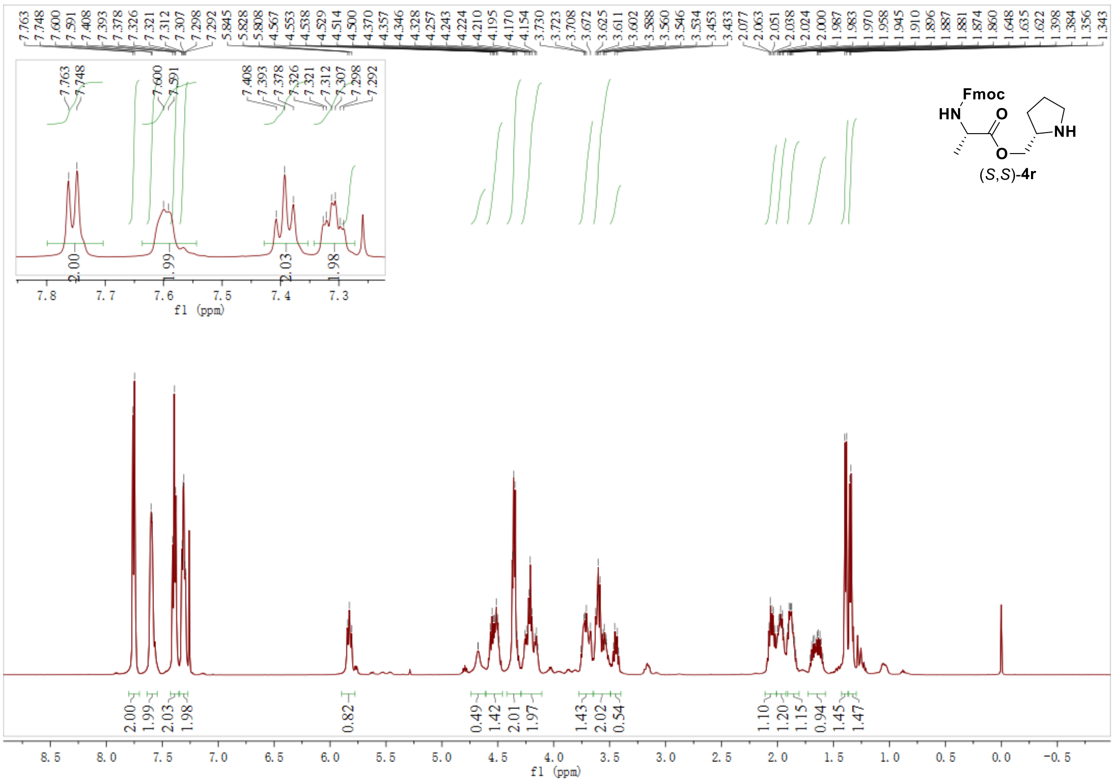

125 MHz, $CDCl_3$, ^{13}C NMR

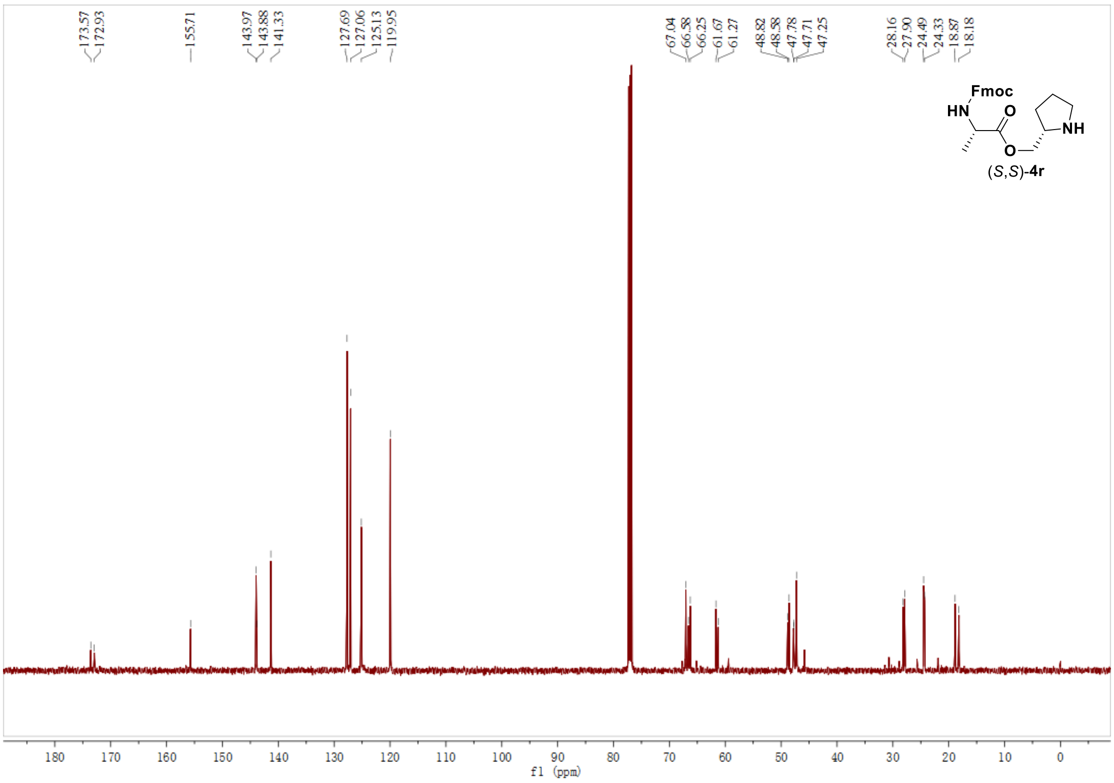

500 MHz, $CDCl_3$, ^1H NMR

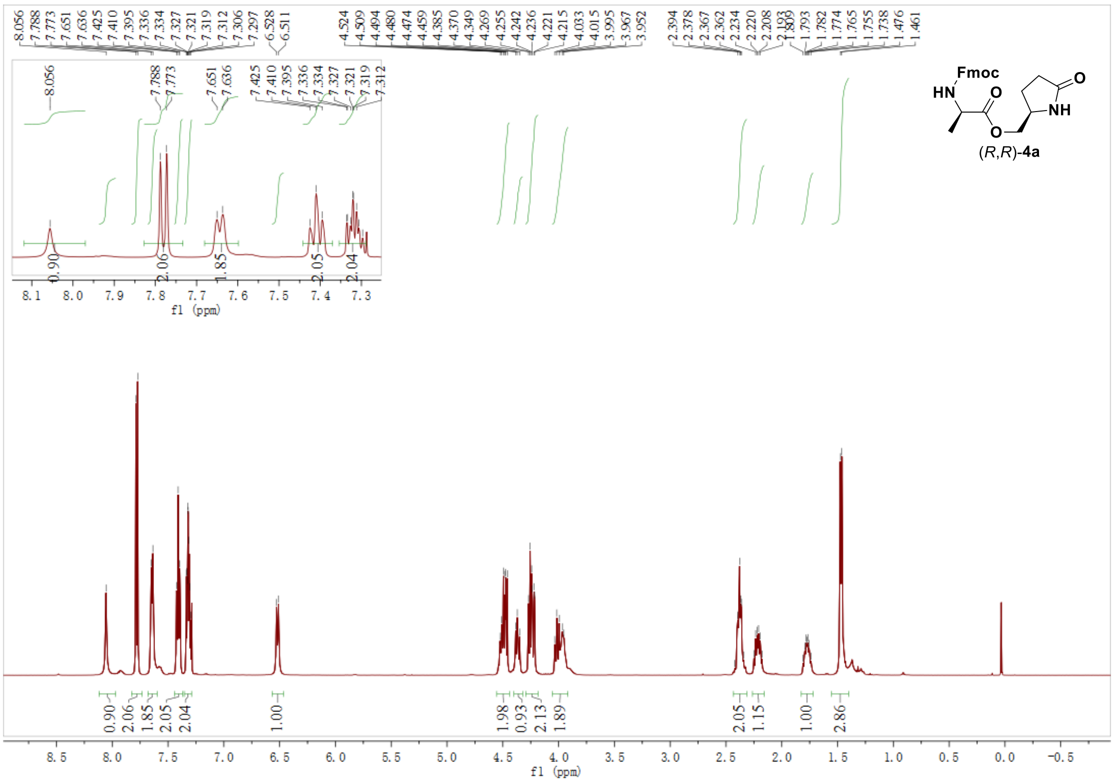

125 MHz, $CDCl_3$, ^{13}C NMR

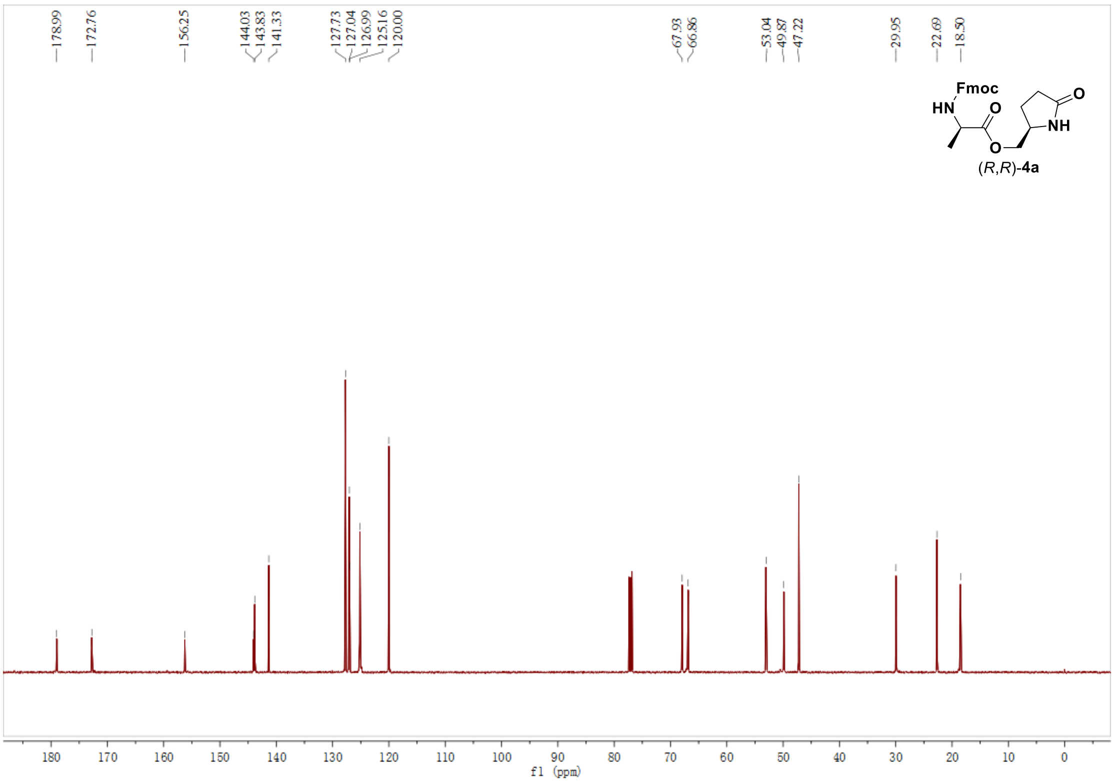

500 MHz, CDCl$_3$, ^{1}H NMR

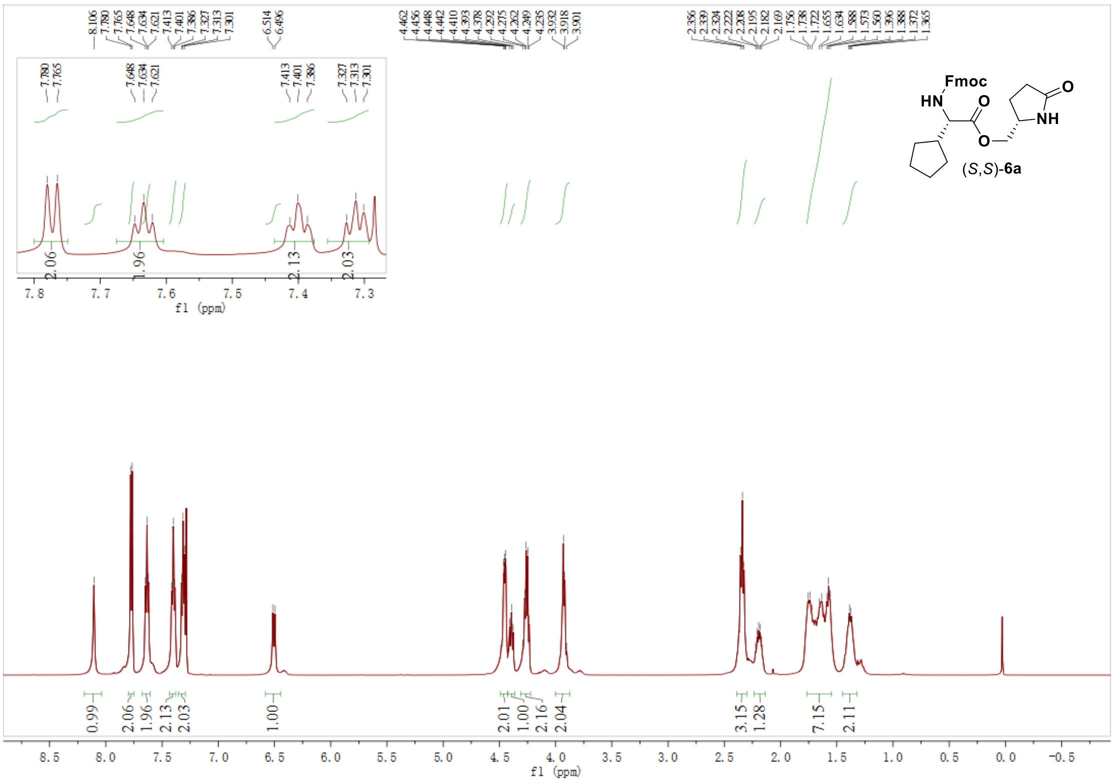

125 MHz, CDCl$_3$, ^{13}C NMR

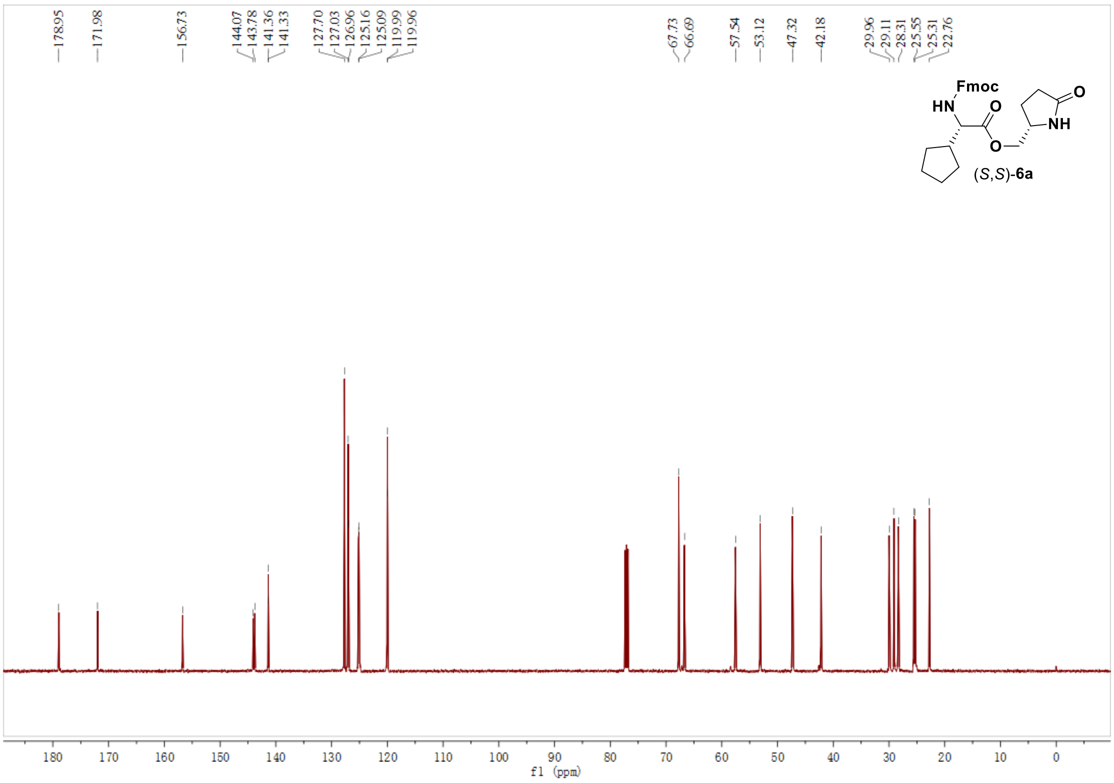

500 MHz, $CDCl_3$, ^{1}H NMR

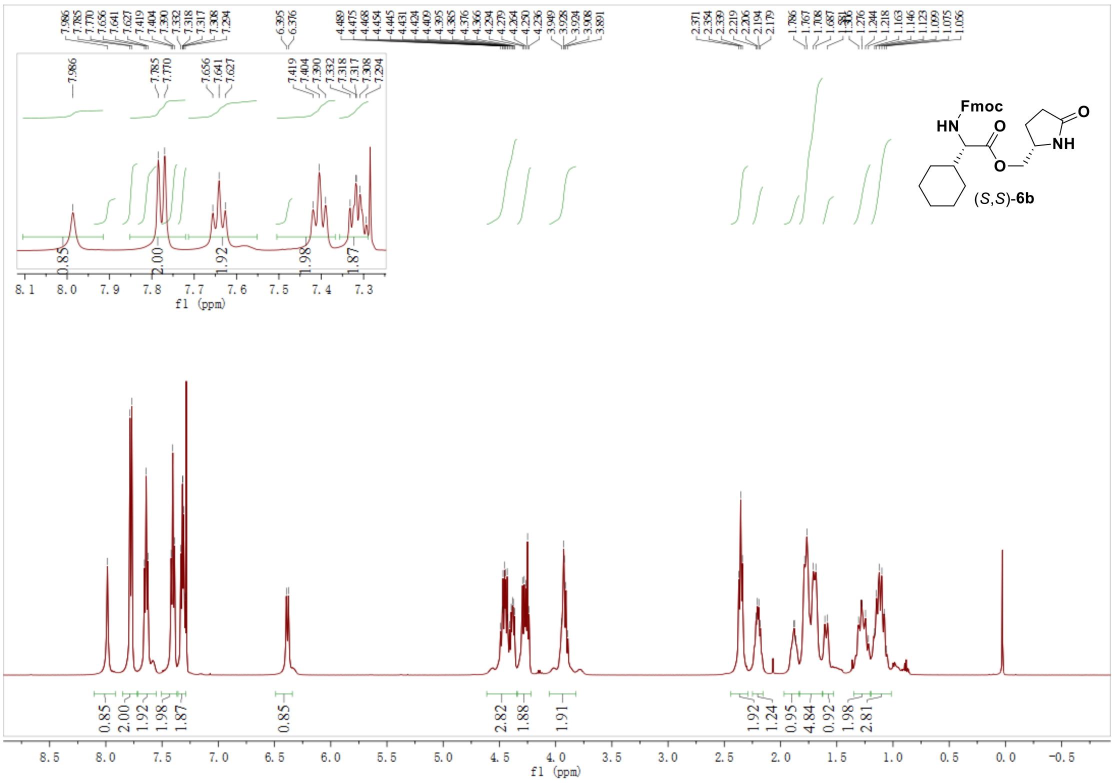

125 MHz, $CDCl_3$, ^{13}C NMR

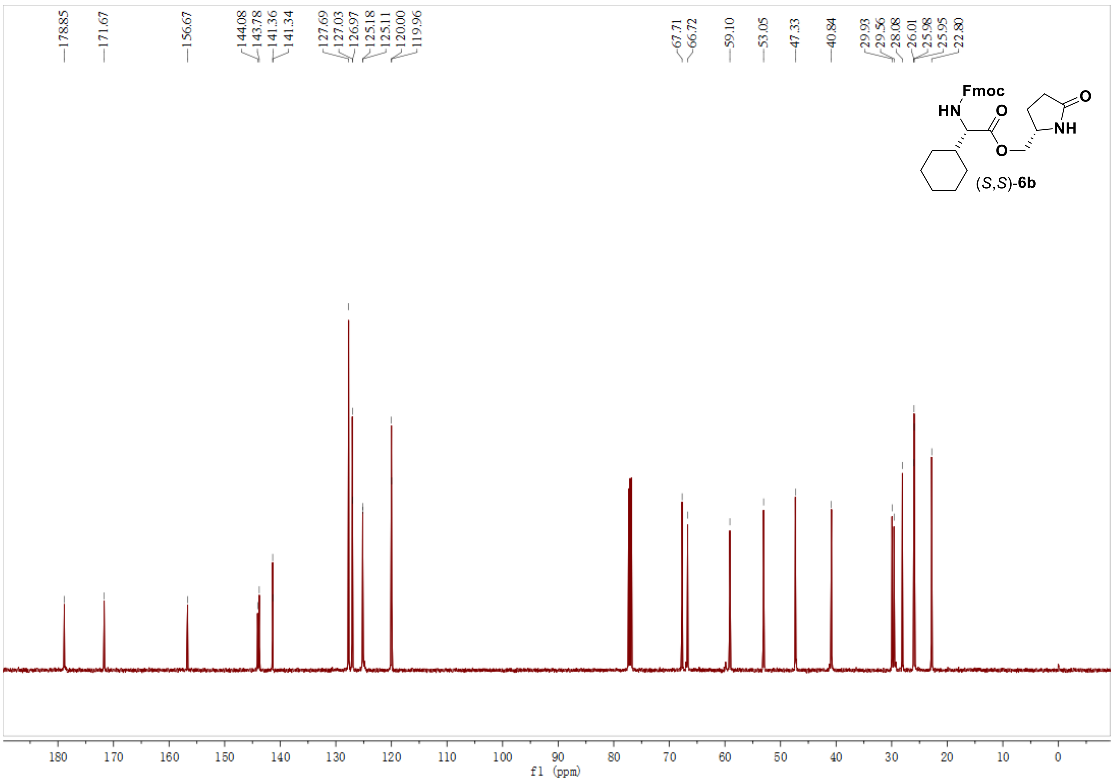

500 MHz, CDCl3, ^{1}H NMR

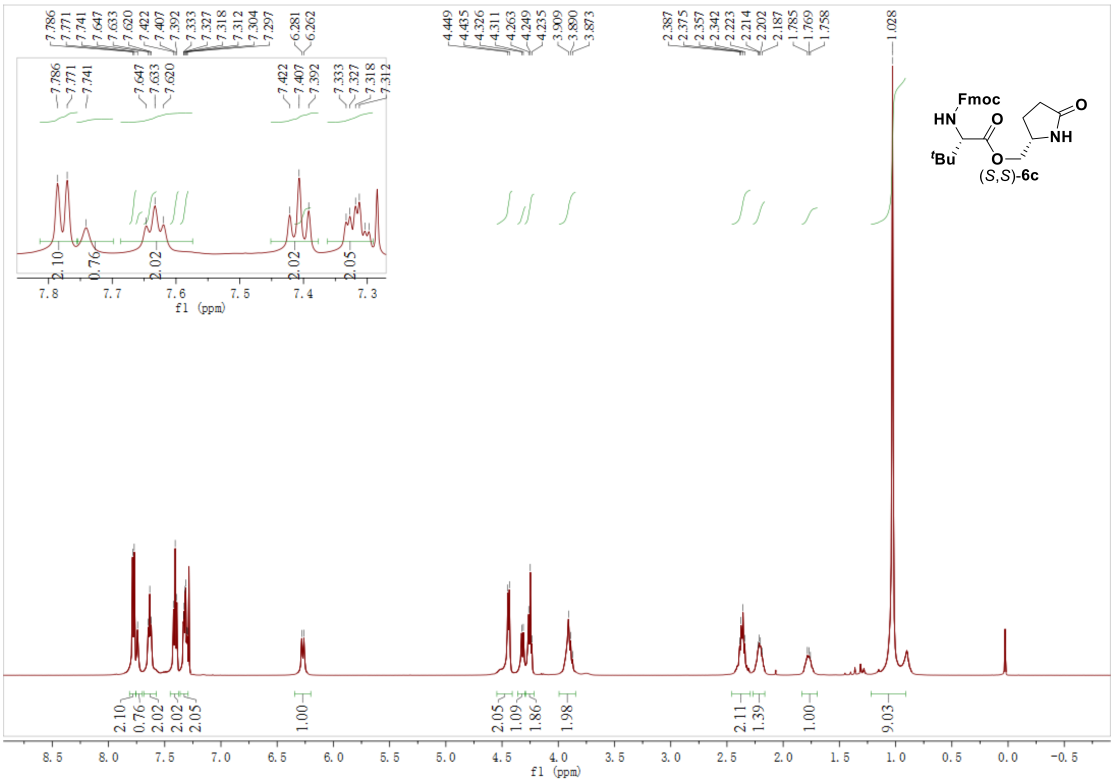

125 MHz, CDCl3, ^{13}C NMR

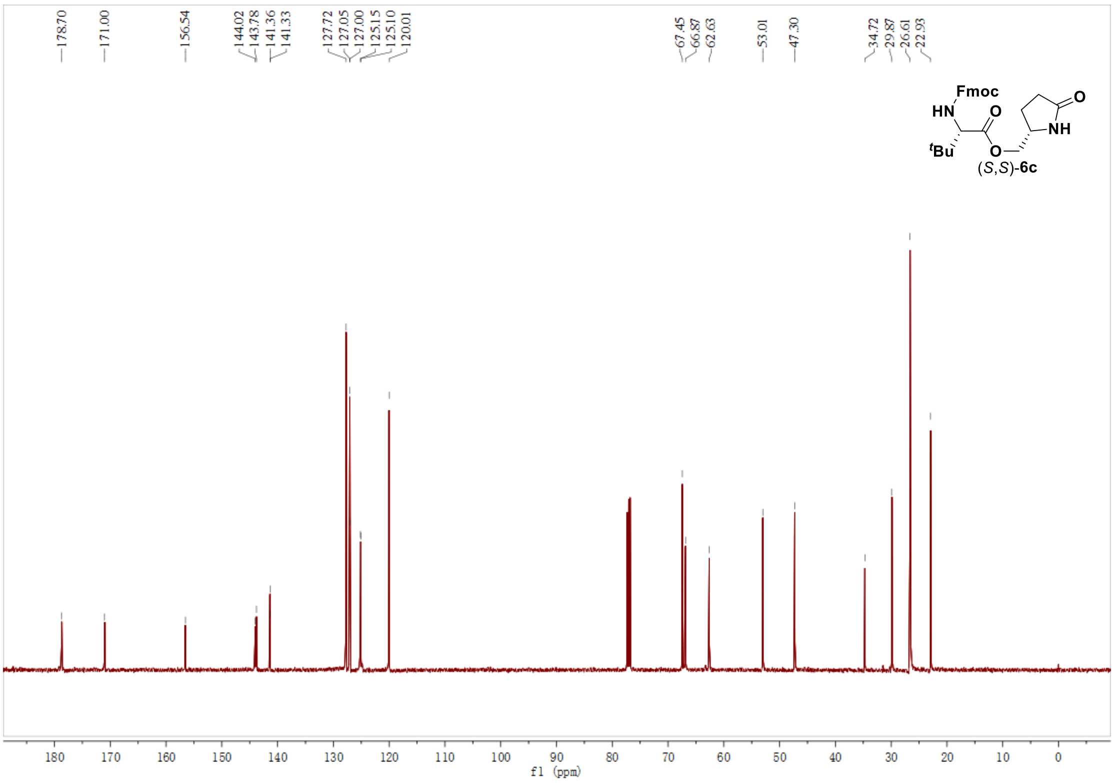

500 MHz, $CDCl_3$, ^1H NMR

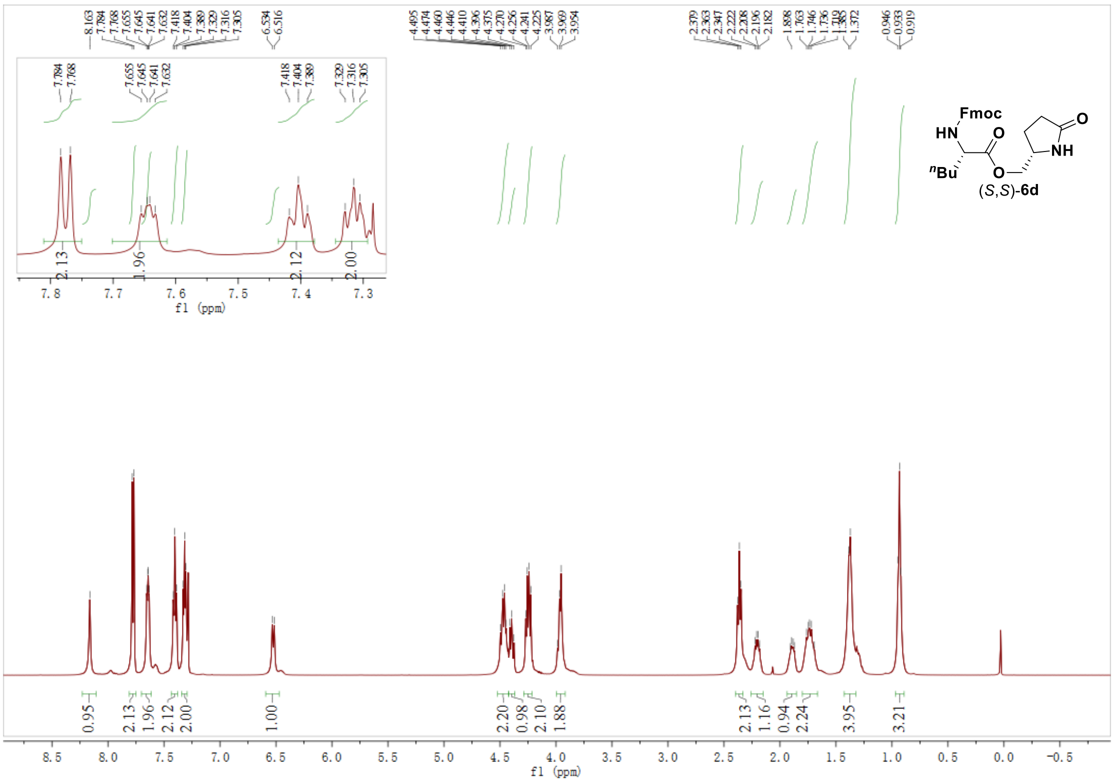

125 MHz, $CDCl_3$, ^{13}C NMR

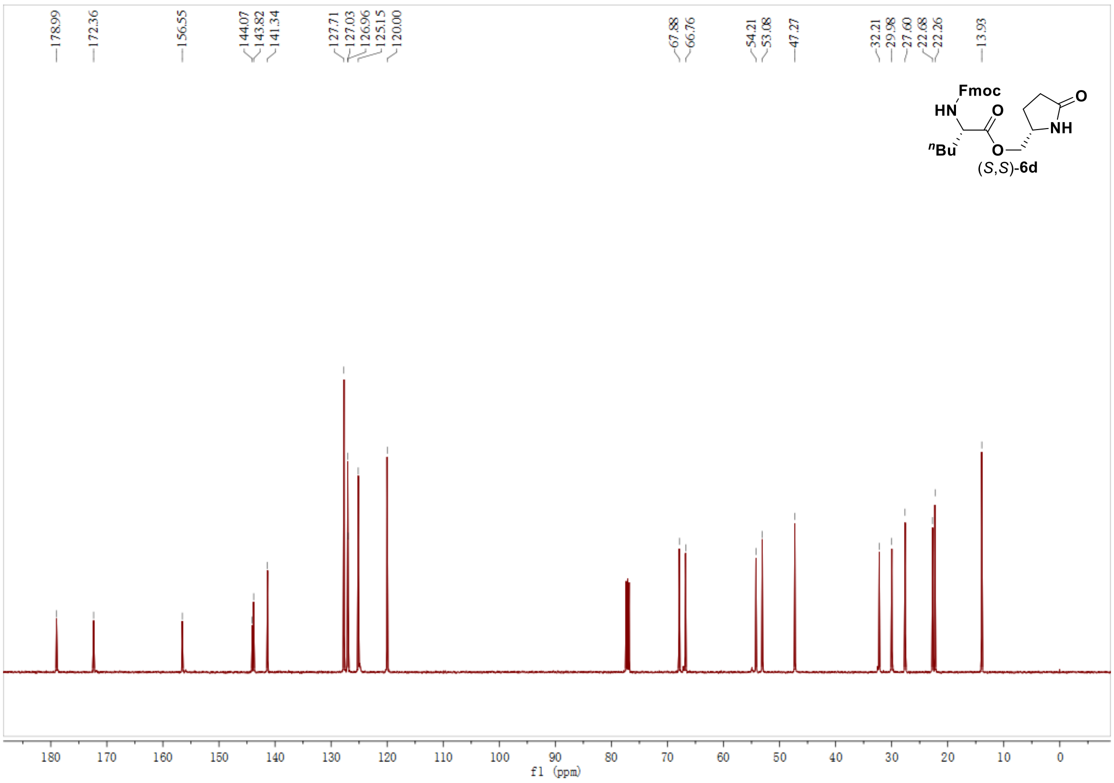

500 MHz, $CDCl_3$, ^{1}H NMR

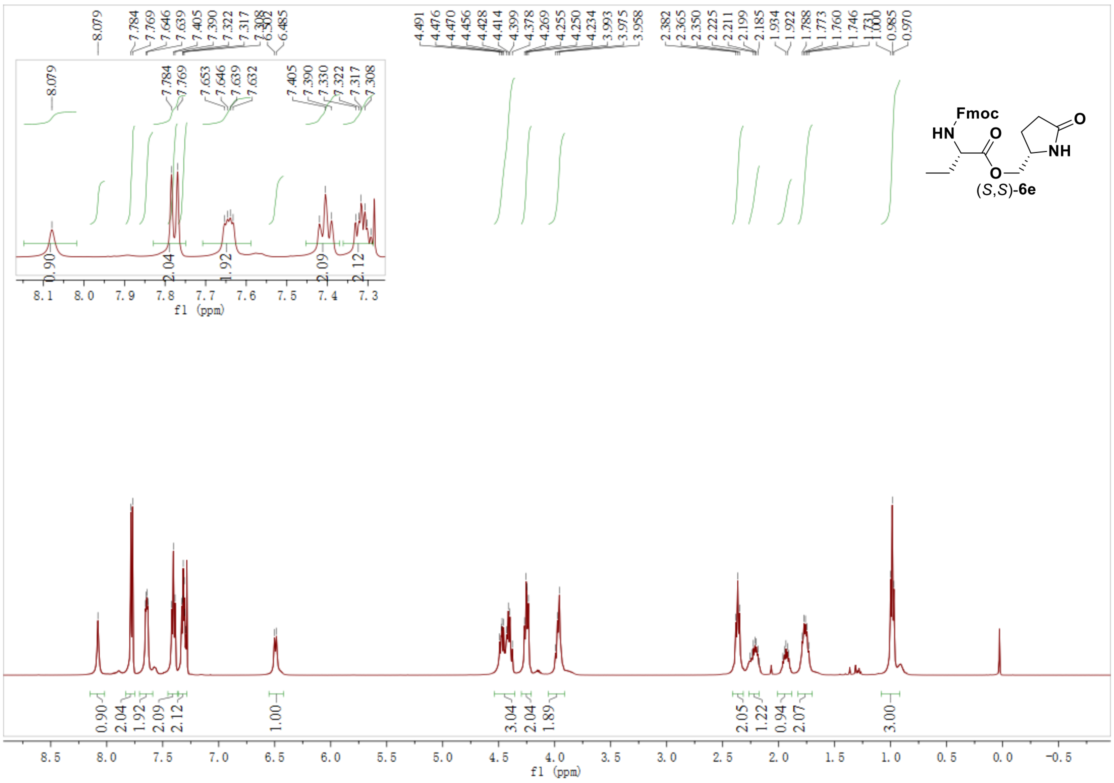

125 MHz, $CDCl_3$, ^{13}C NMR

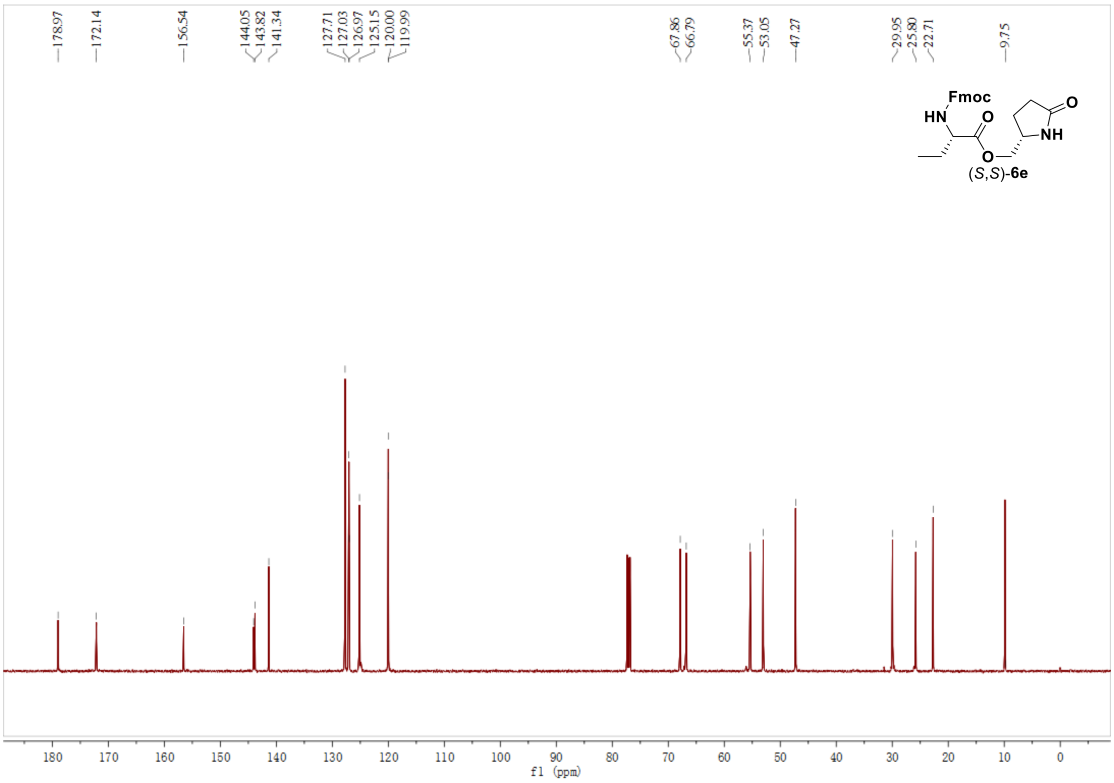

500 MHz, $CDCl_3$, ^1H NMR

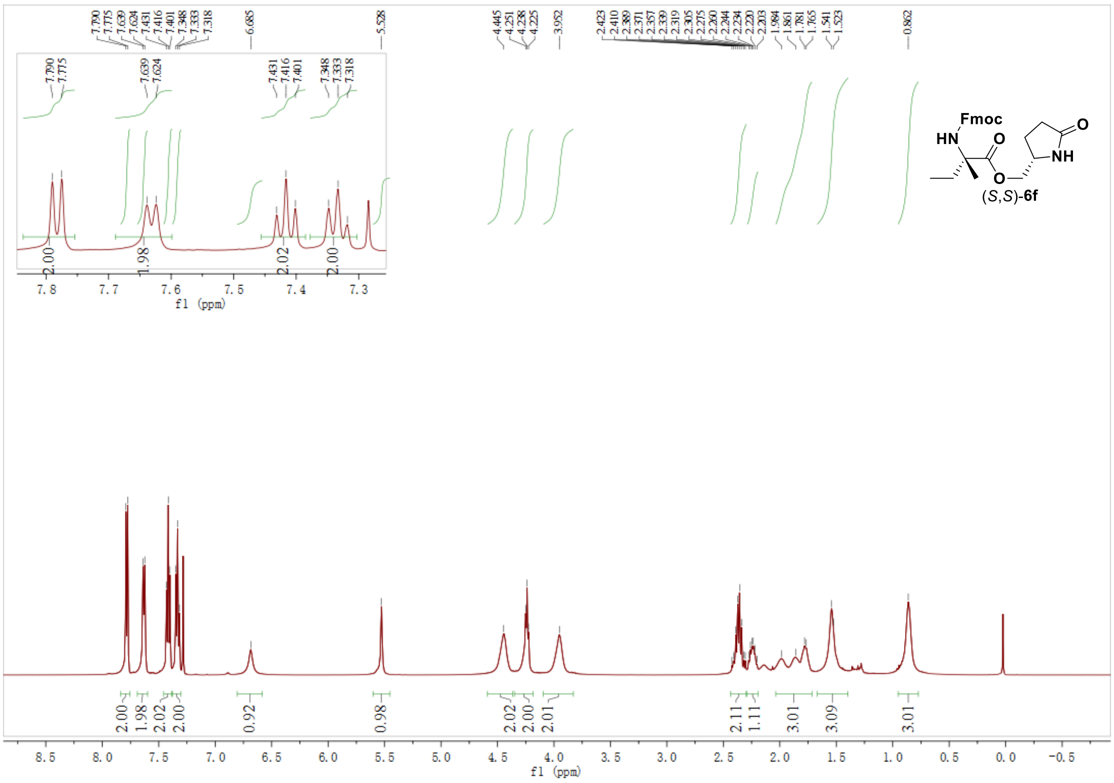

125 MHz, $CDCl_3$, ^{13}C NMR

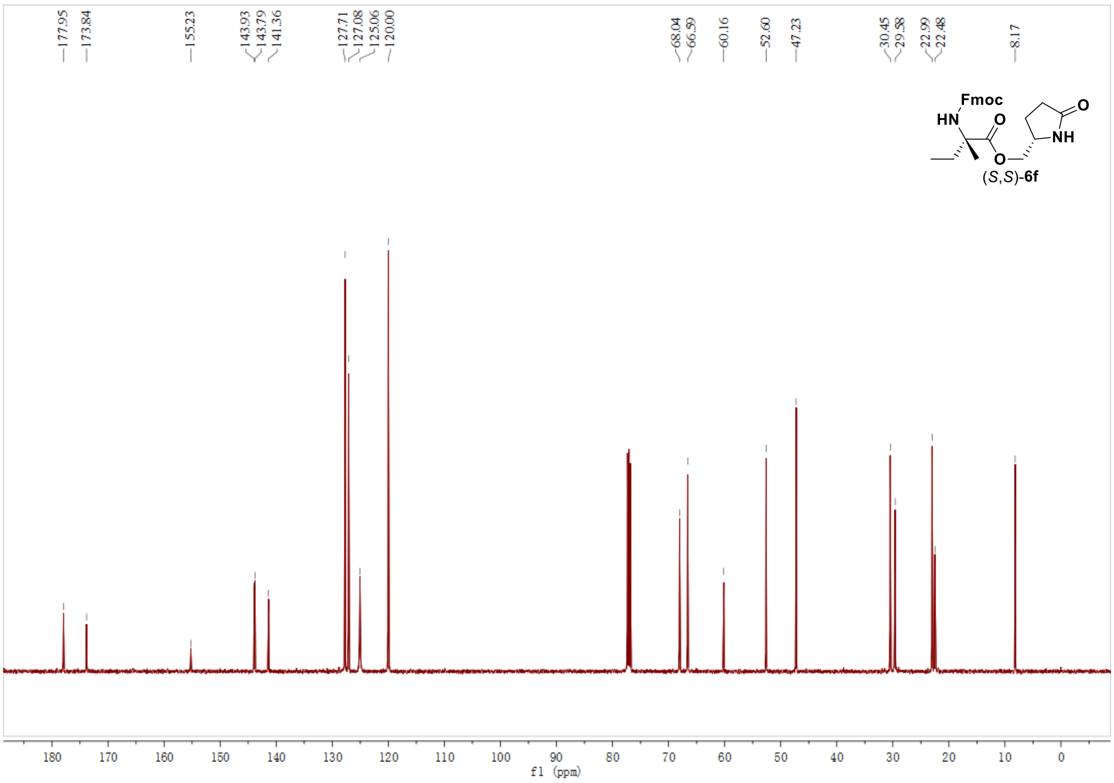

500 MHz, $CDCl_3$, ^{1}H NMR

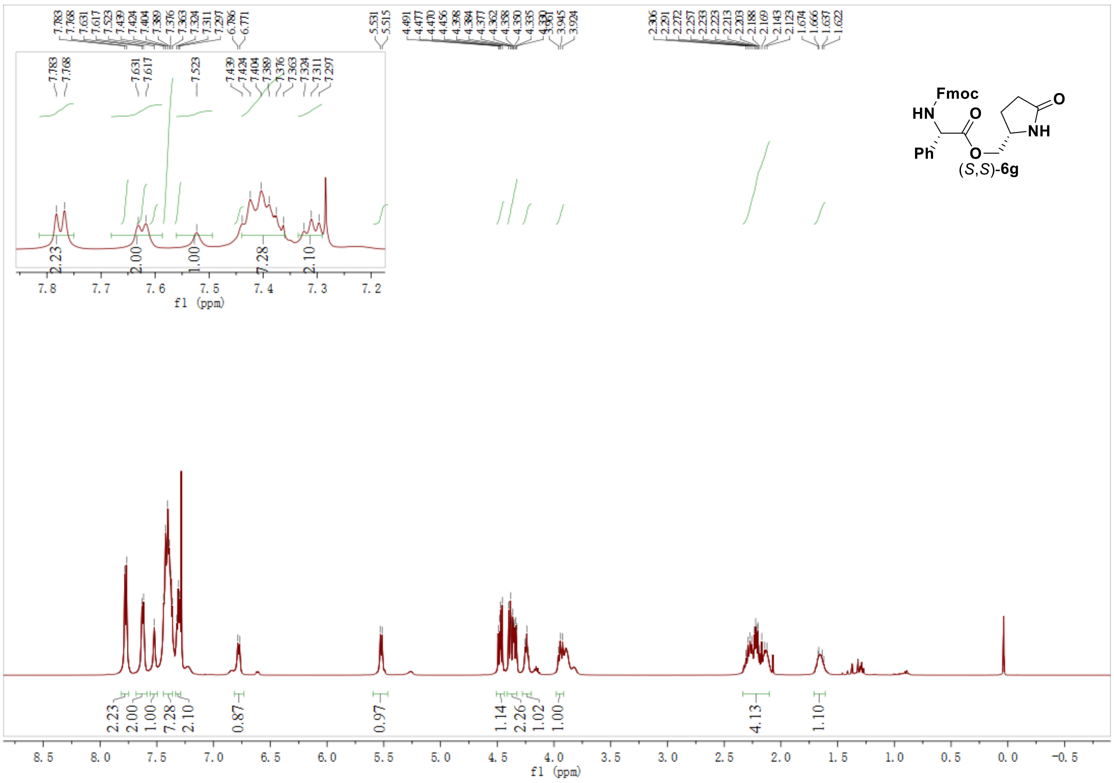

125 MHz, $CDCl_3$, ^{13}C NMR

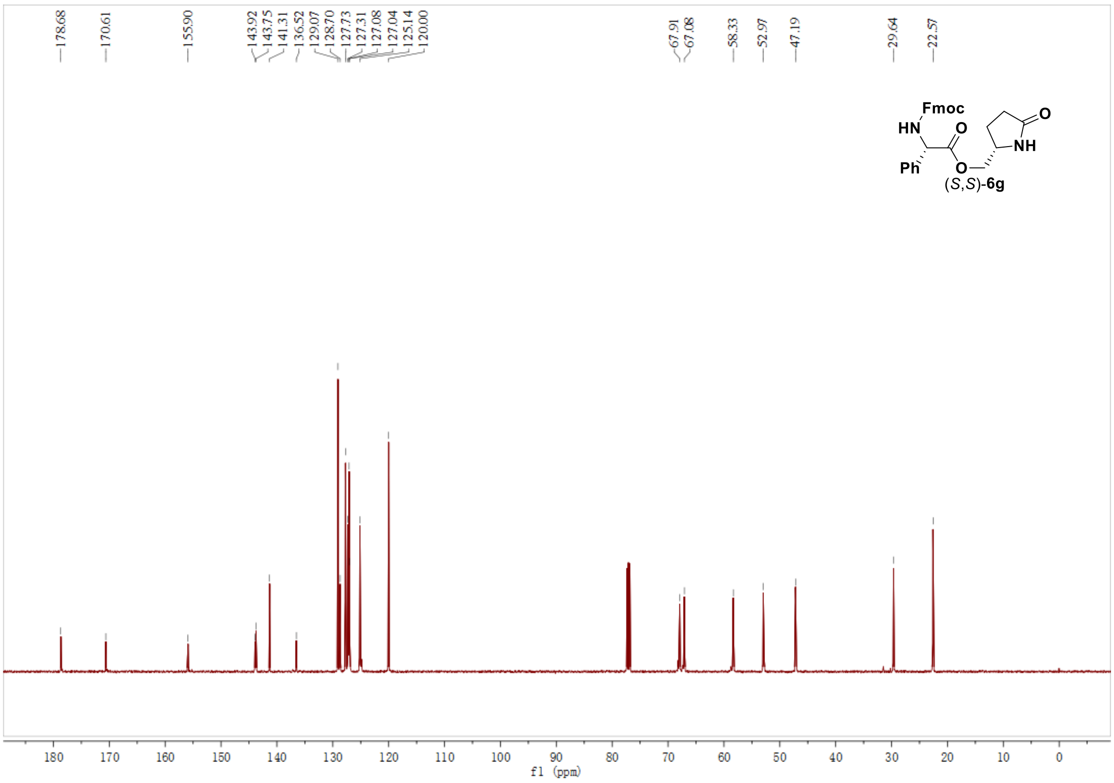

500 MHz, DMSO-d_6, ^{1}H NMR

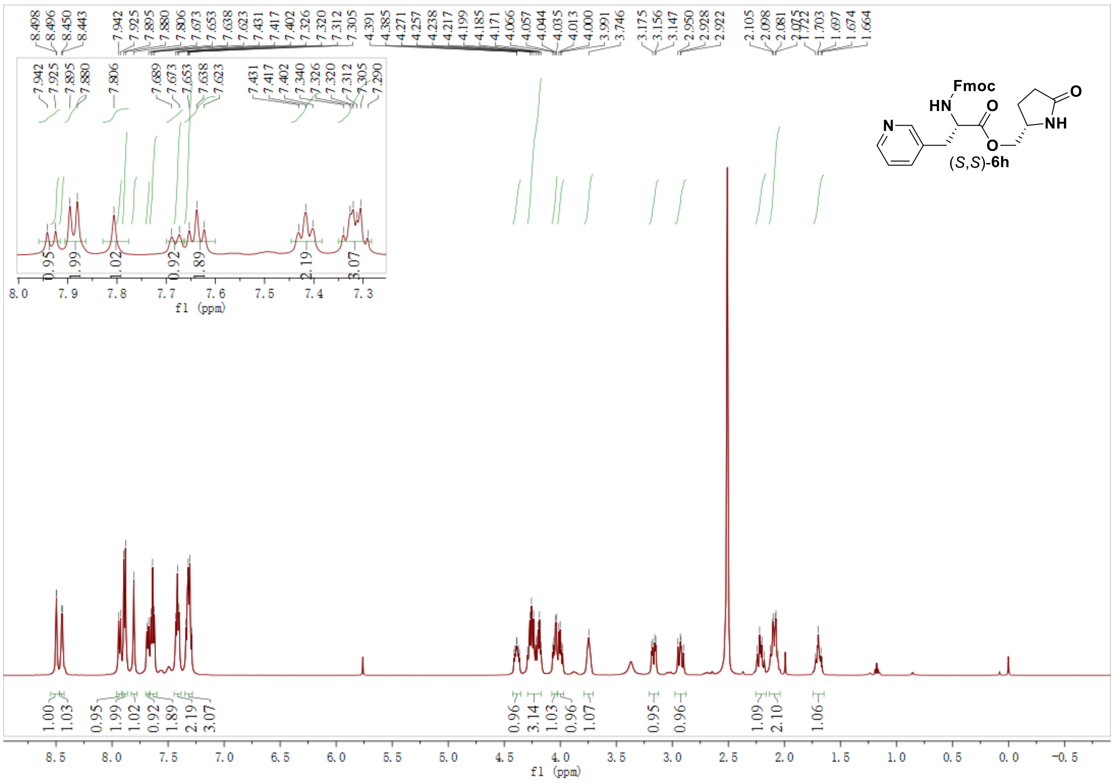

125 MHz, DMSO-d_6, ^{13}C NMR

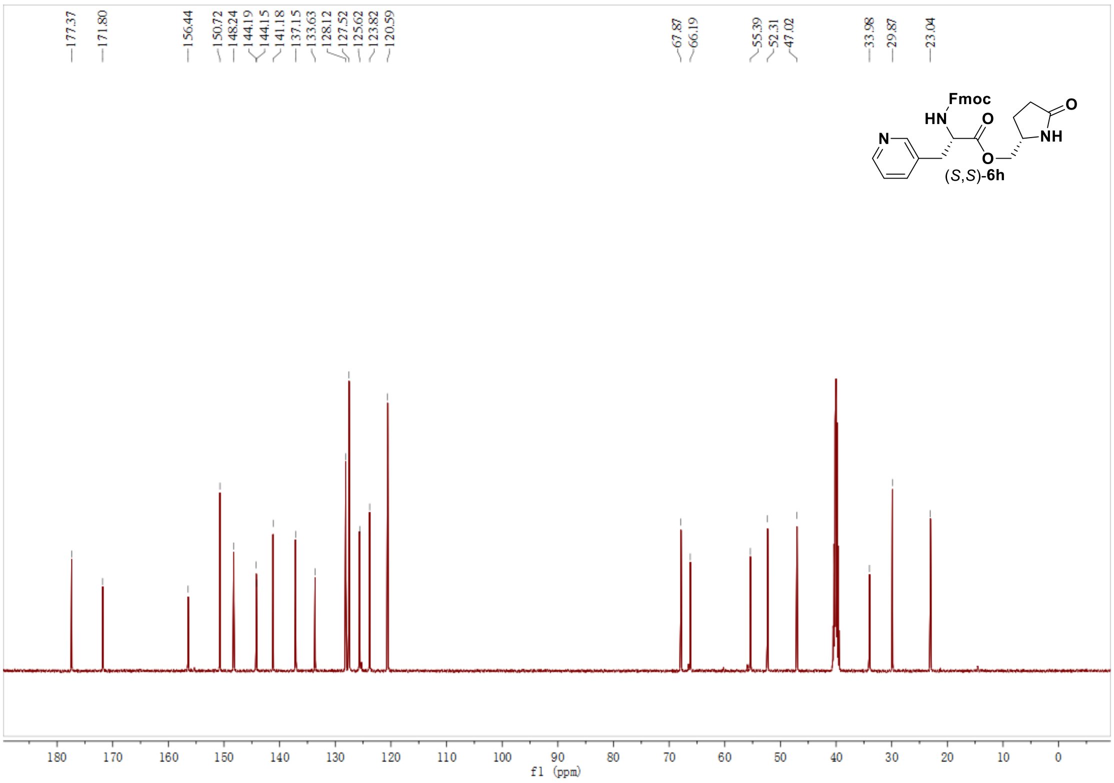

500 MHz, $CDCl_3$, ^1H NMR

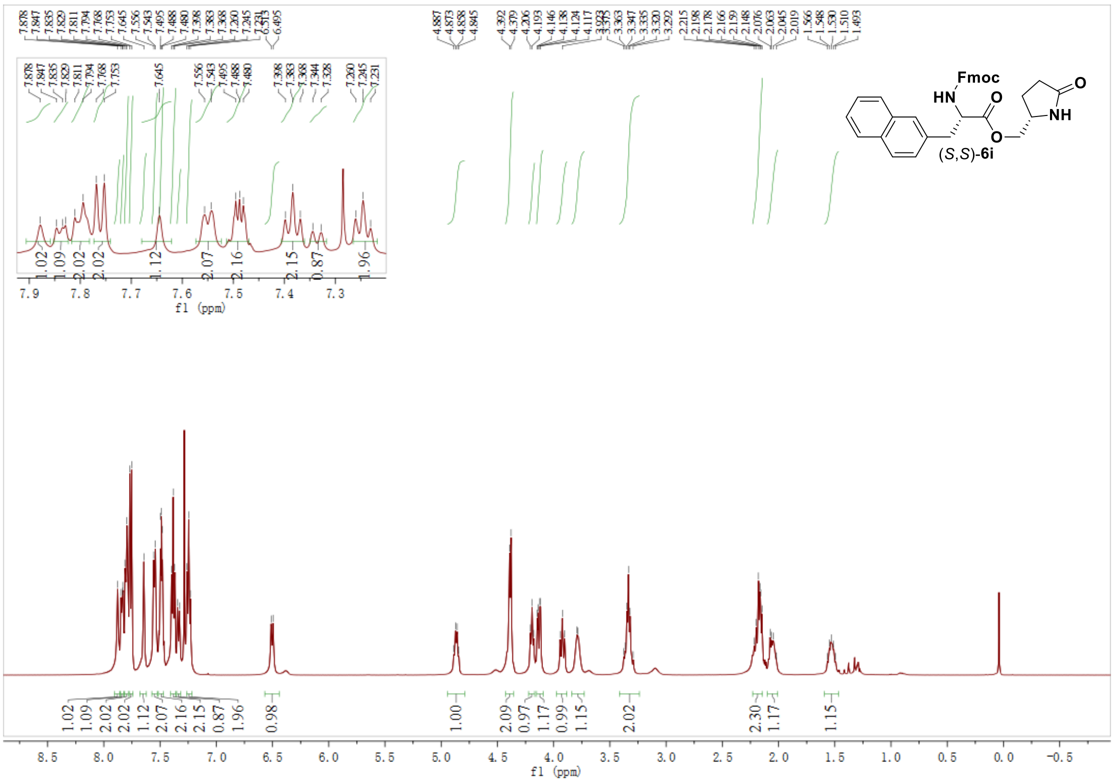

125 MHz, $CDCl_3$, ^{13}C NMR

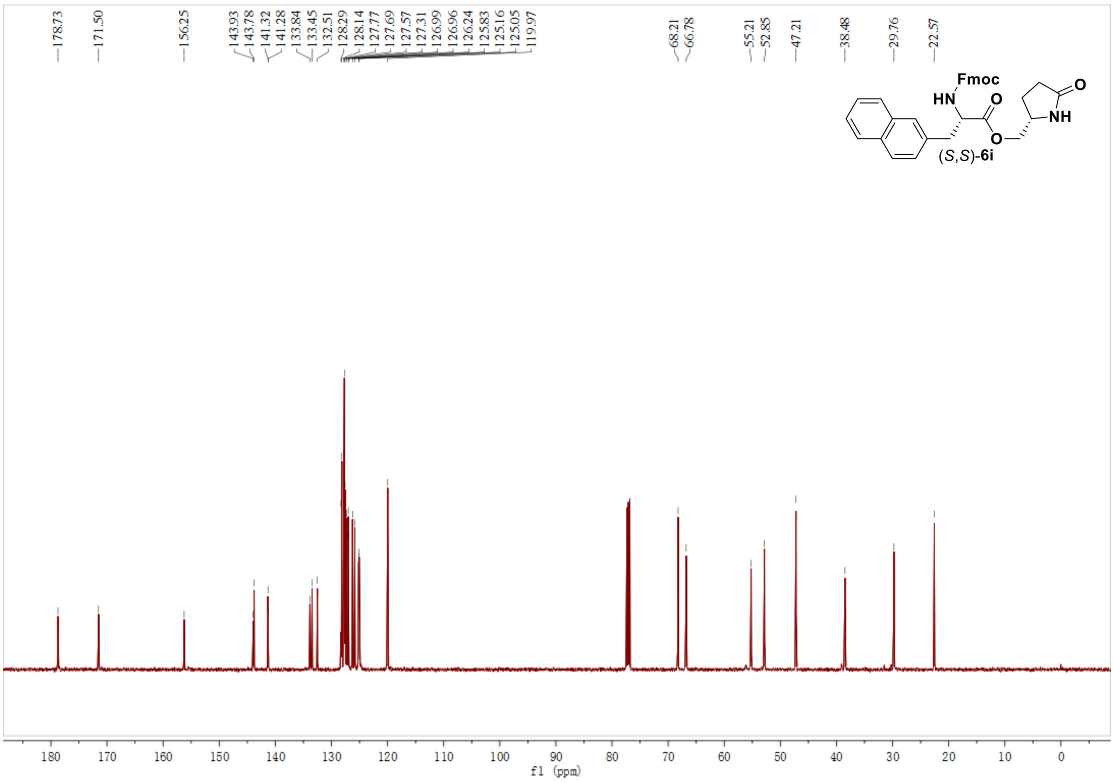

500 MHz, $CDCl_3$, ^1H NMR

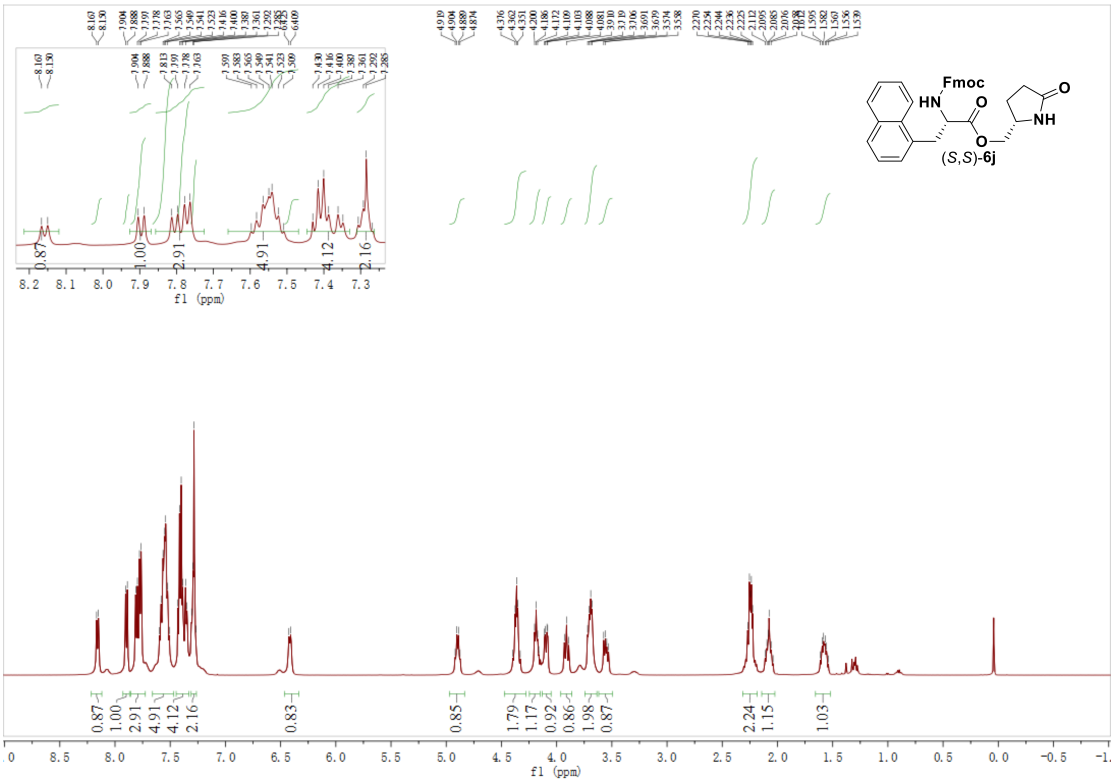

125 MHz, $CDCl_3$, ^{13}C NMR

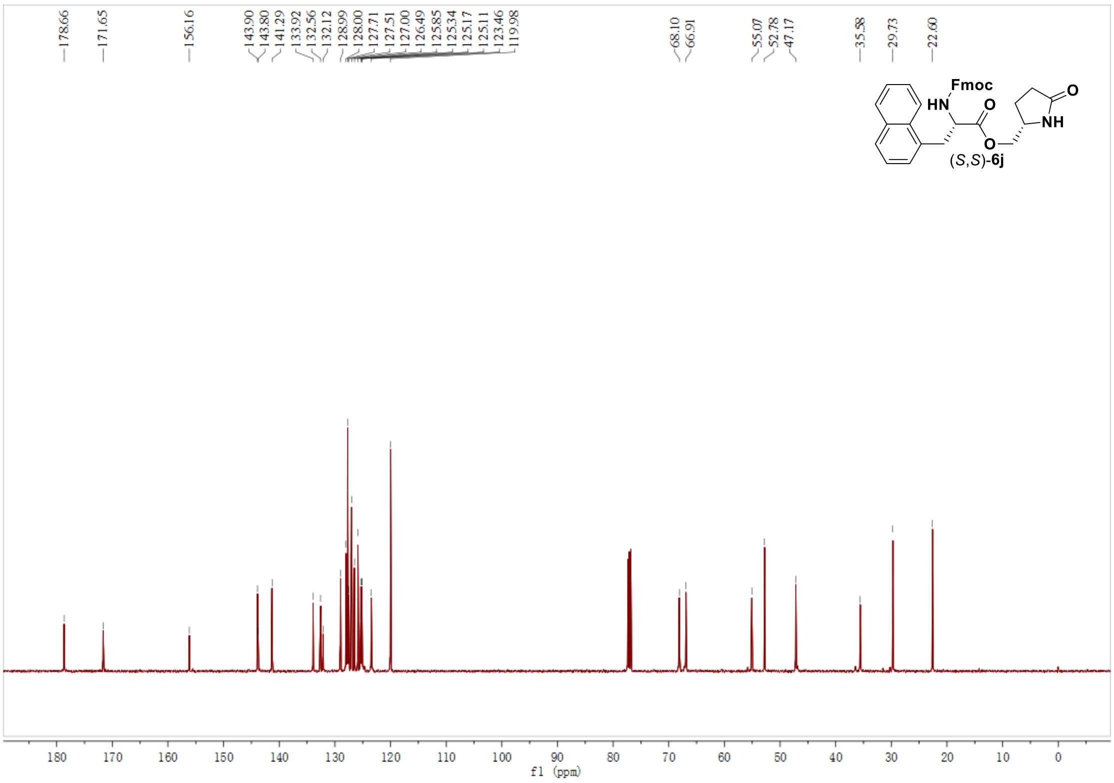

500 MHz, $CDCl_3$, ^1H NMR

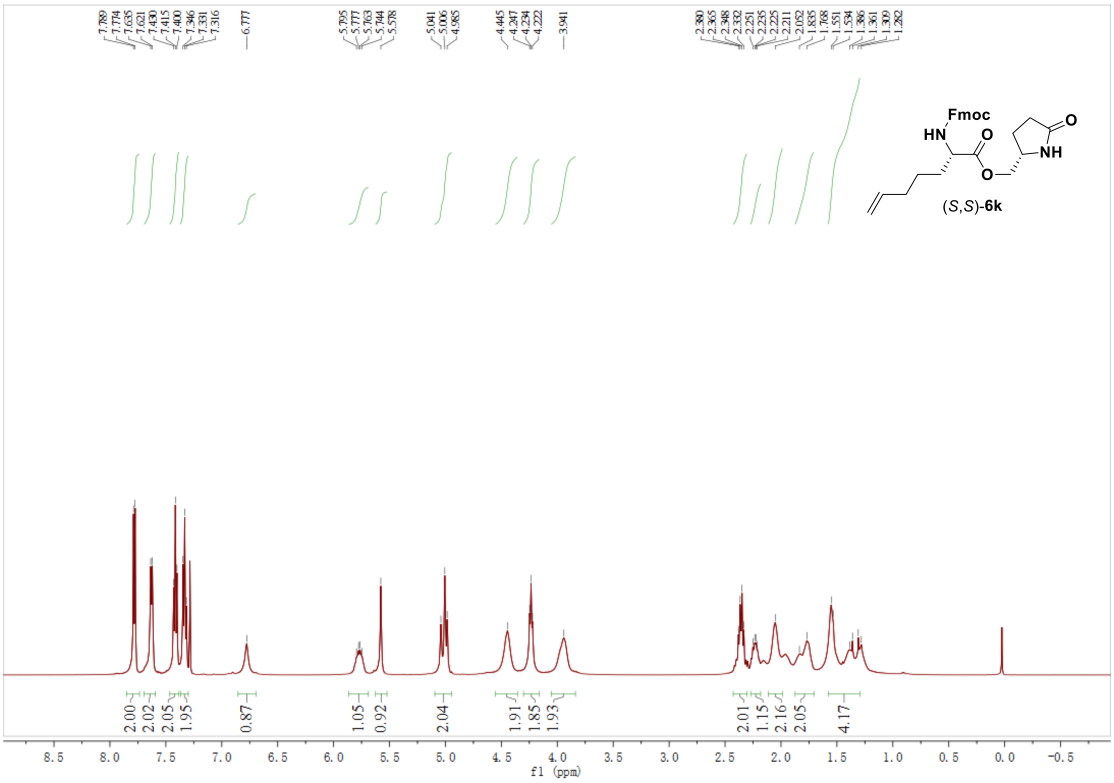

125 MHz, $CDCl_3$, ^{13}C NMR

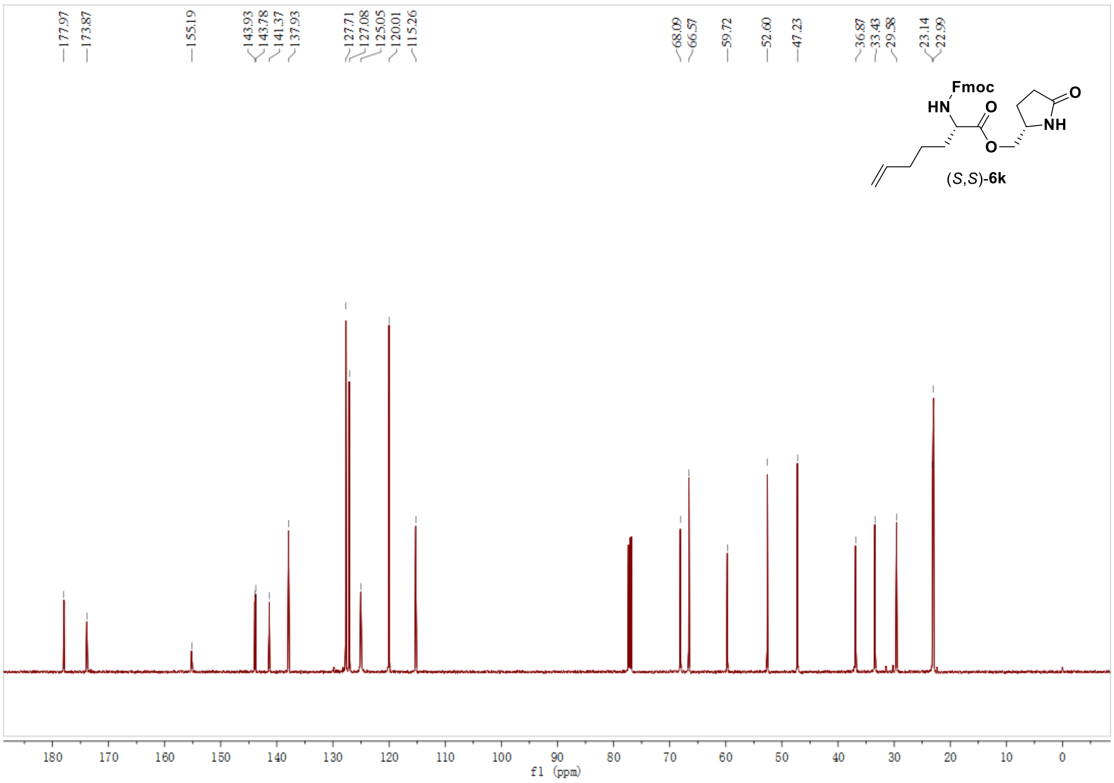

500 MHz, DMSO-d_6, ^{1}H NMR

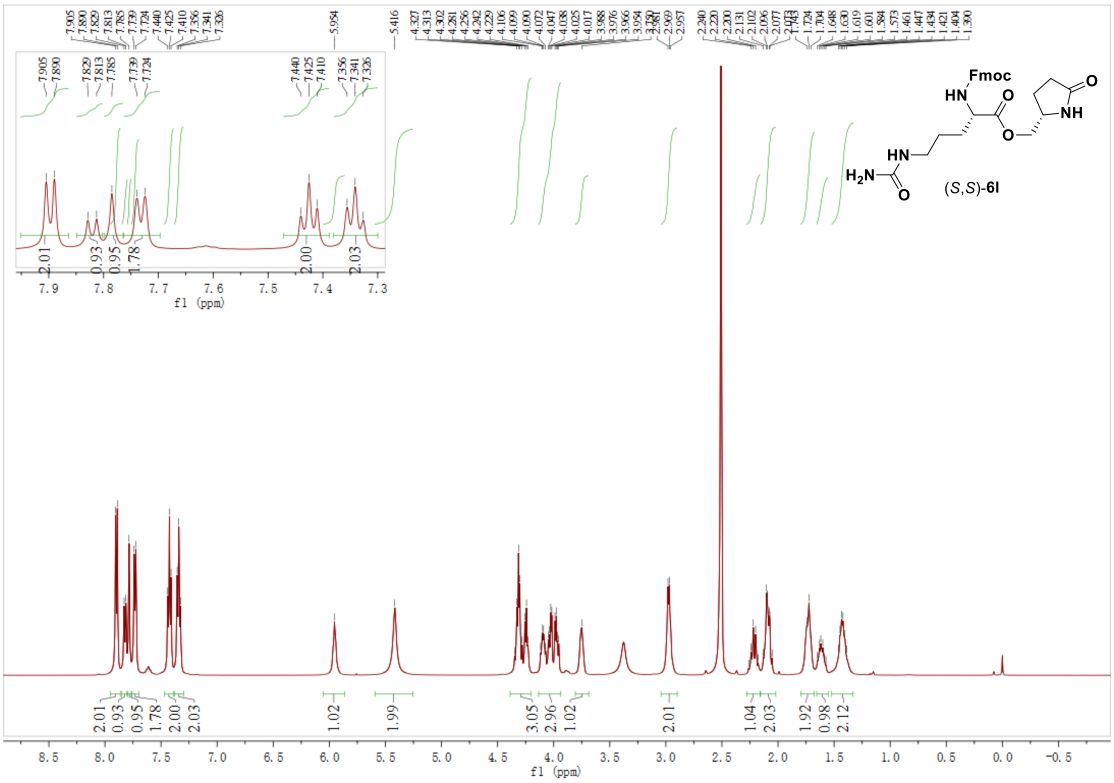

125 MHz, DMSO-d_6, ^{13}C NMR

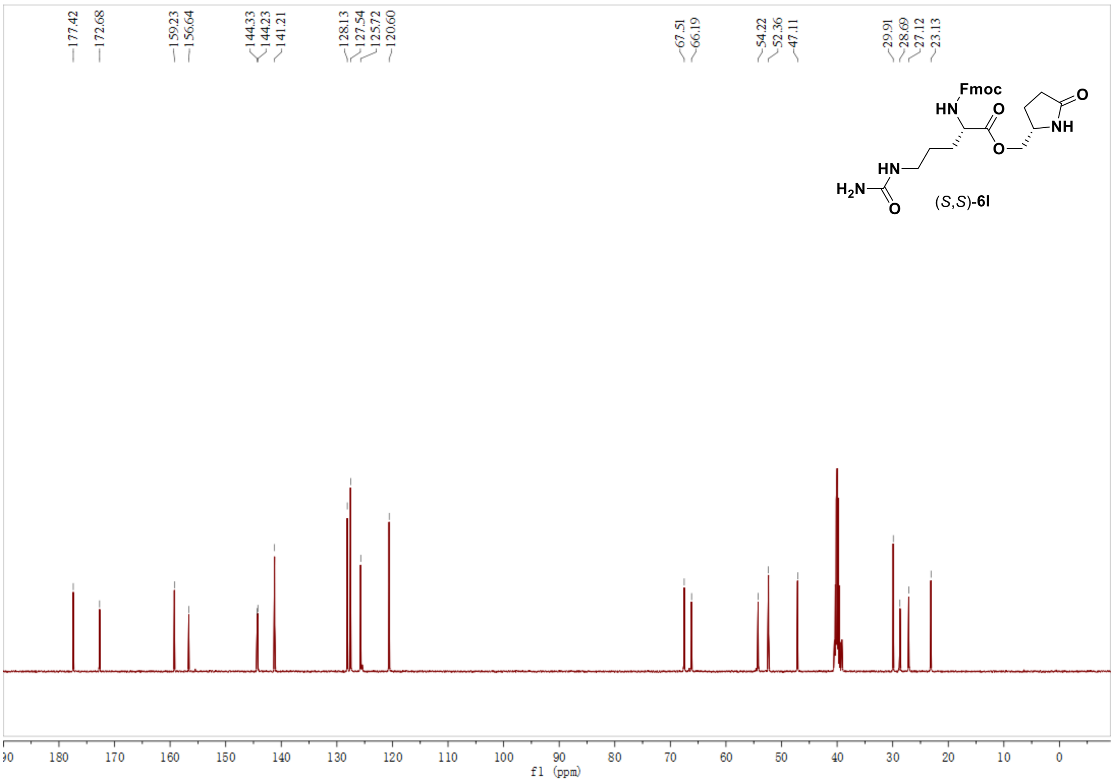

500 MHz, $CDCl_3$, ^{1}H NMR

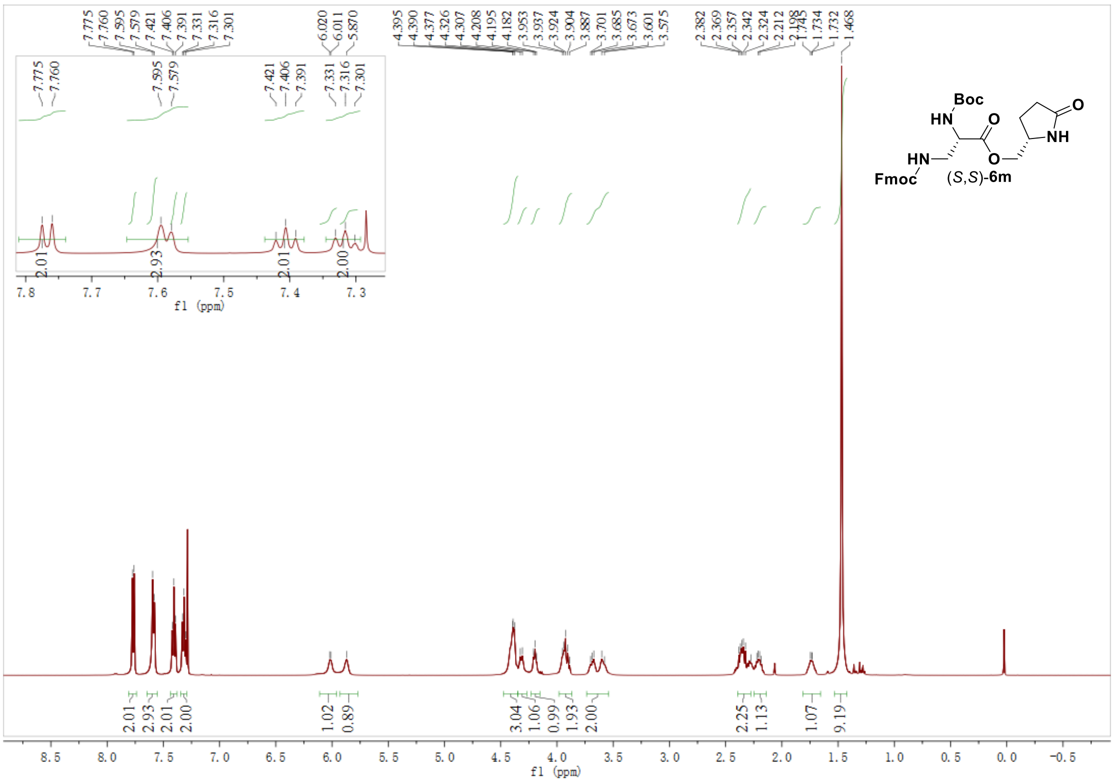

125 MHz, $CDCl_3$, ^{13}C NMR

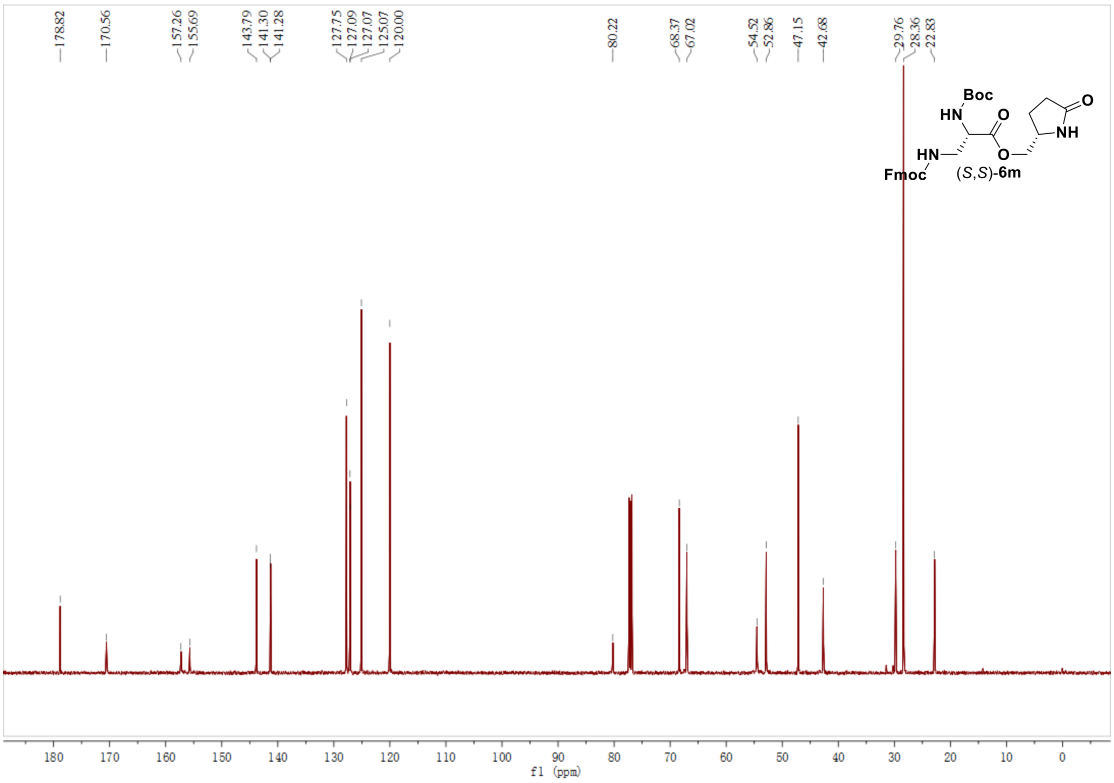

500 MHz, CDCl₃, ¹H NMR

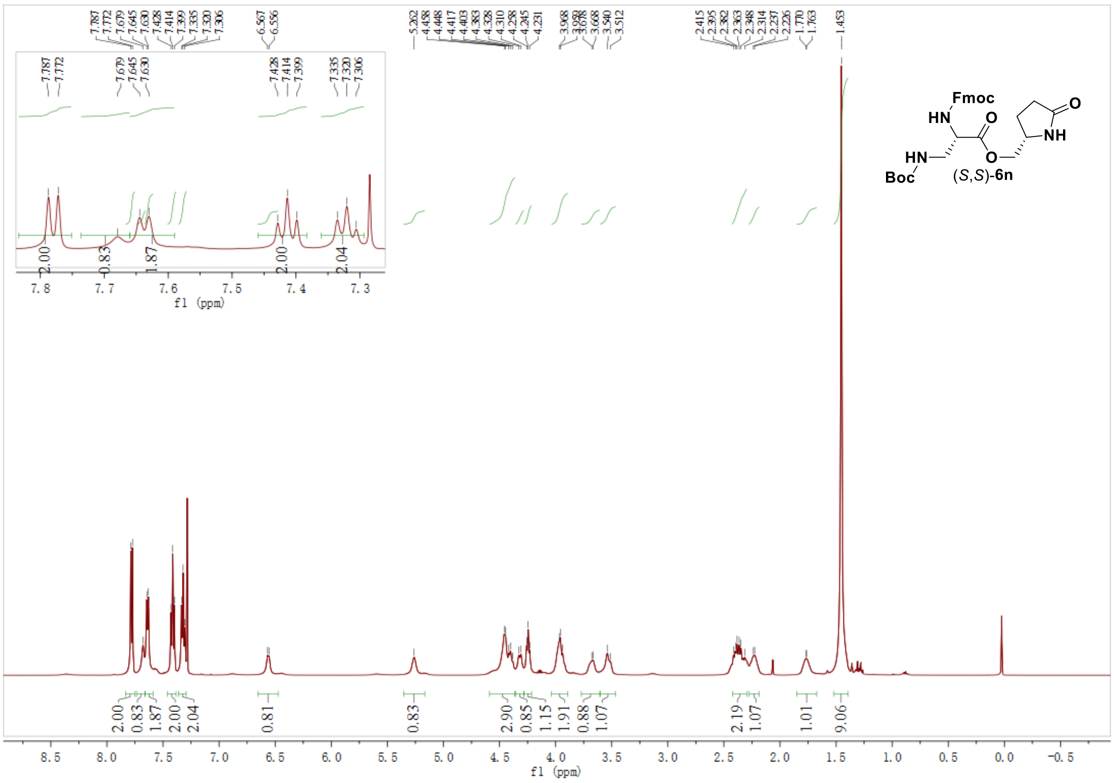

125 MHz, CDCl₃, ¹³C NMR

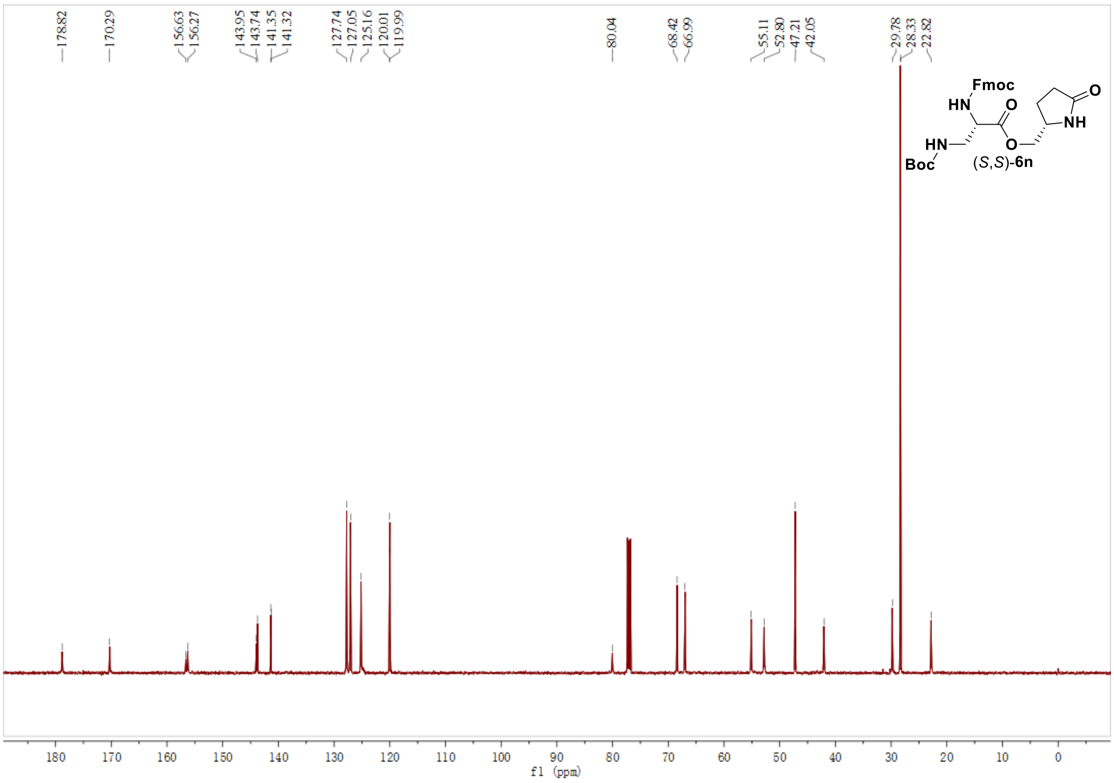

500 MHz, DMSO-d_6, ^1H NMR

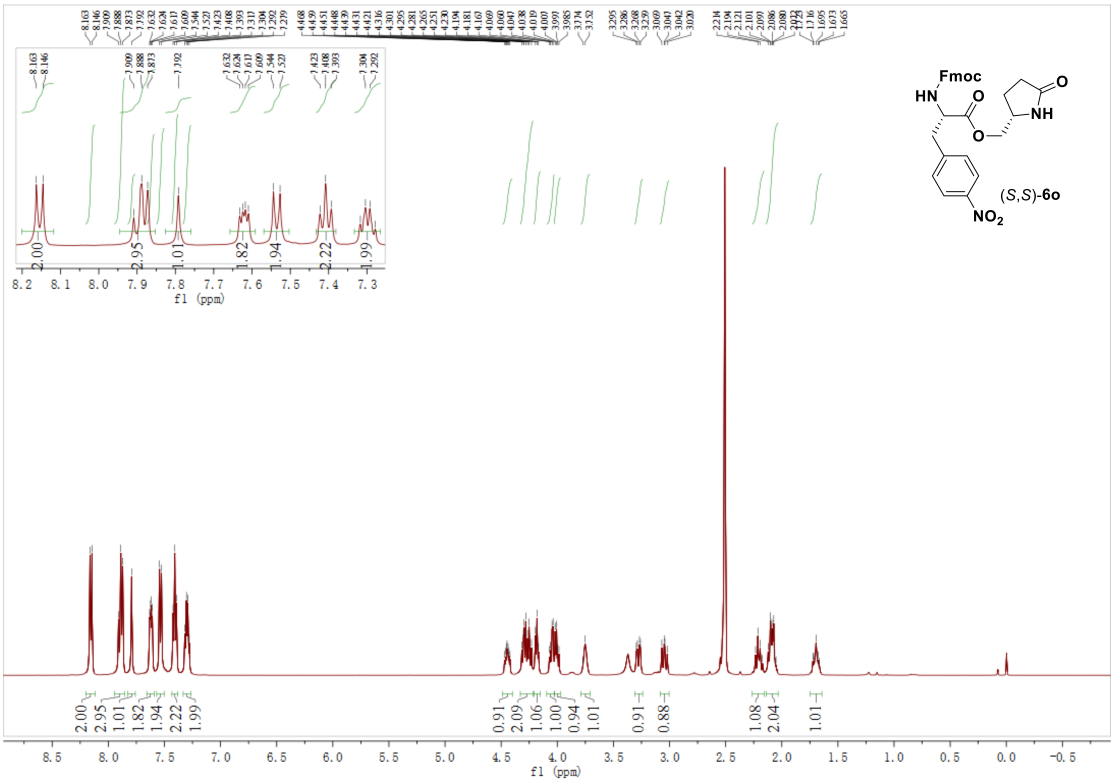

125 MHz, DMSO-d_6, ^{13}C NMR

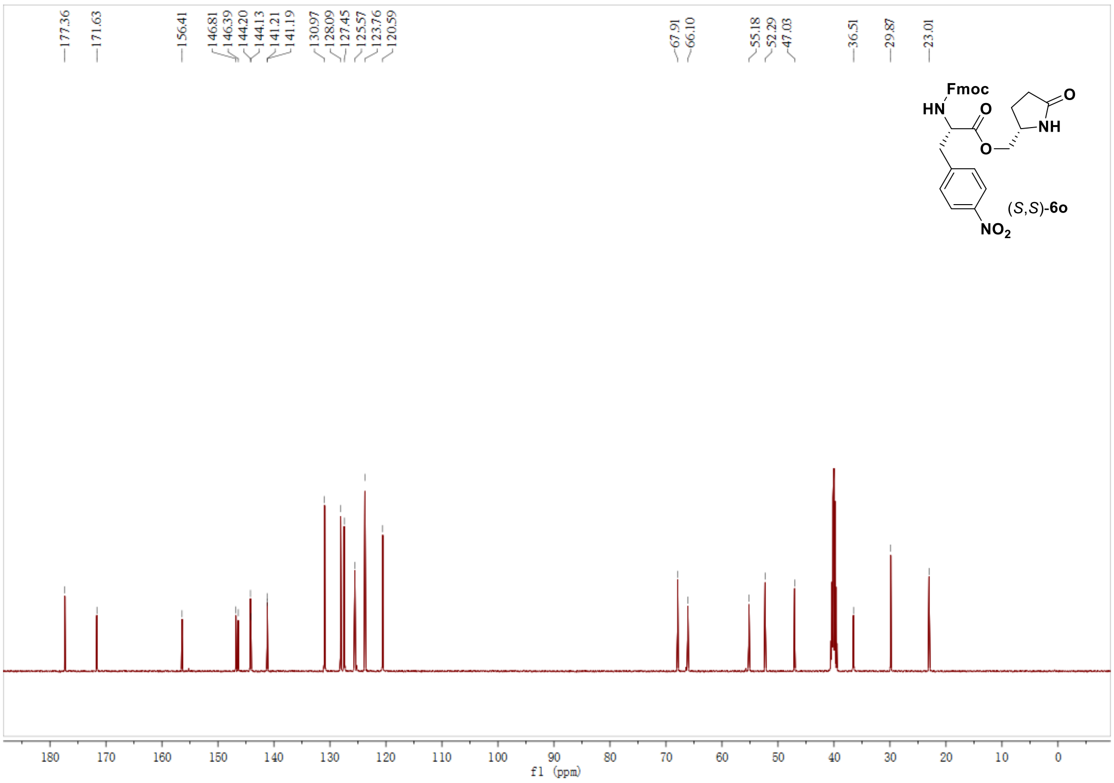

500 MHz, DMSO-d_6, ^{1}H NMR

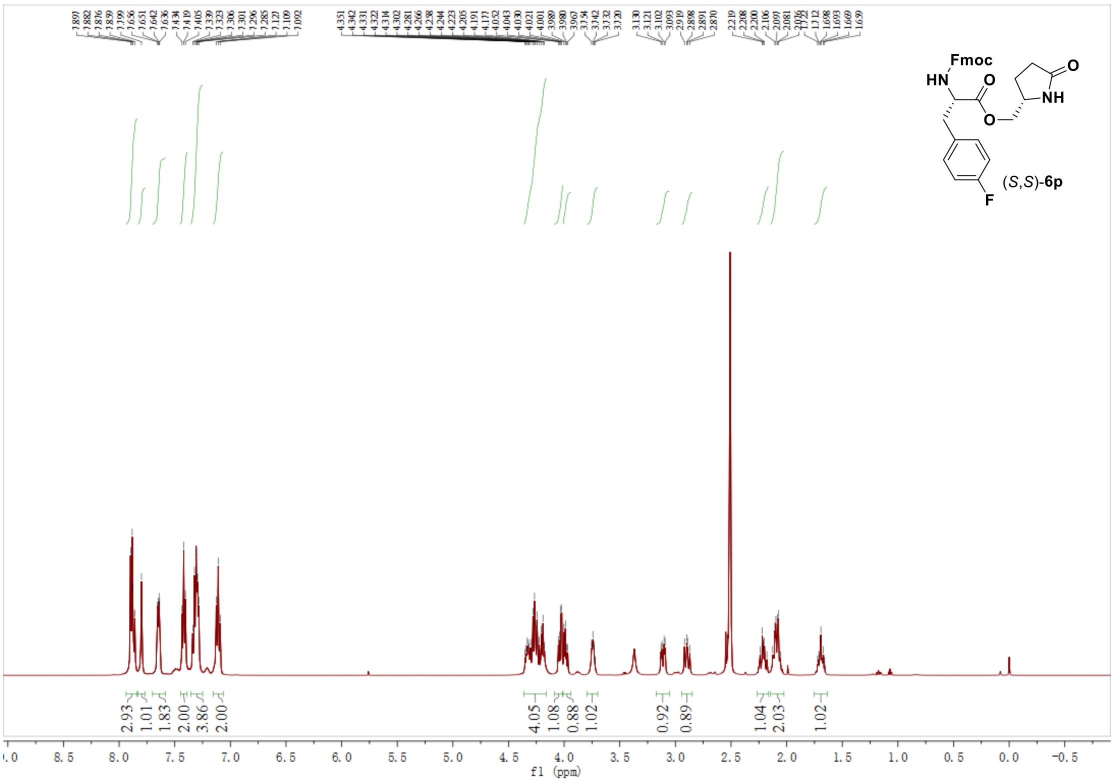

125 MHz, DMSO-d_6, ^{13}C NMR

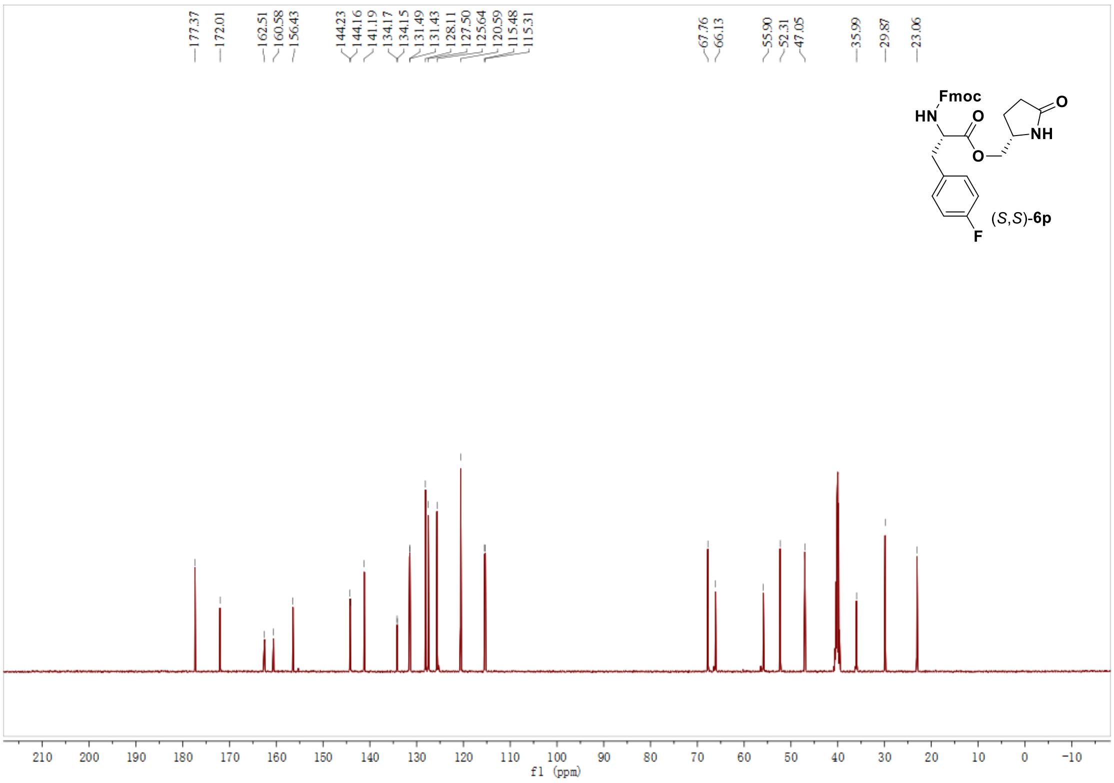

500 MHz, DMSO-d_6, ^{1}H NMR

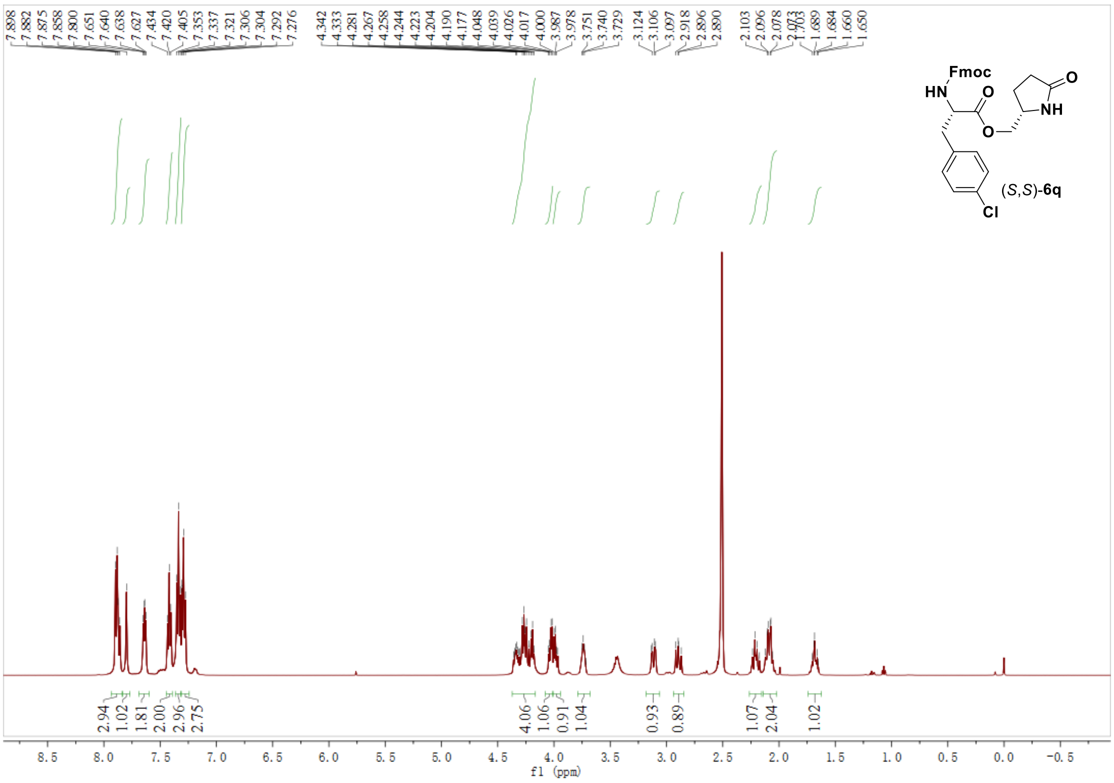

125 MHz, DMSO-d_{6}3, ^{13}C NMR

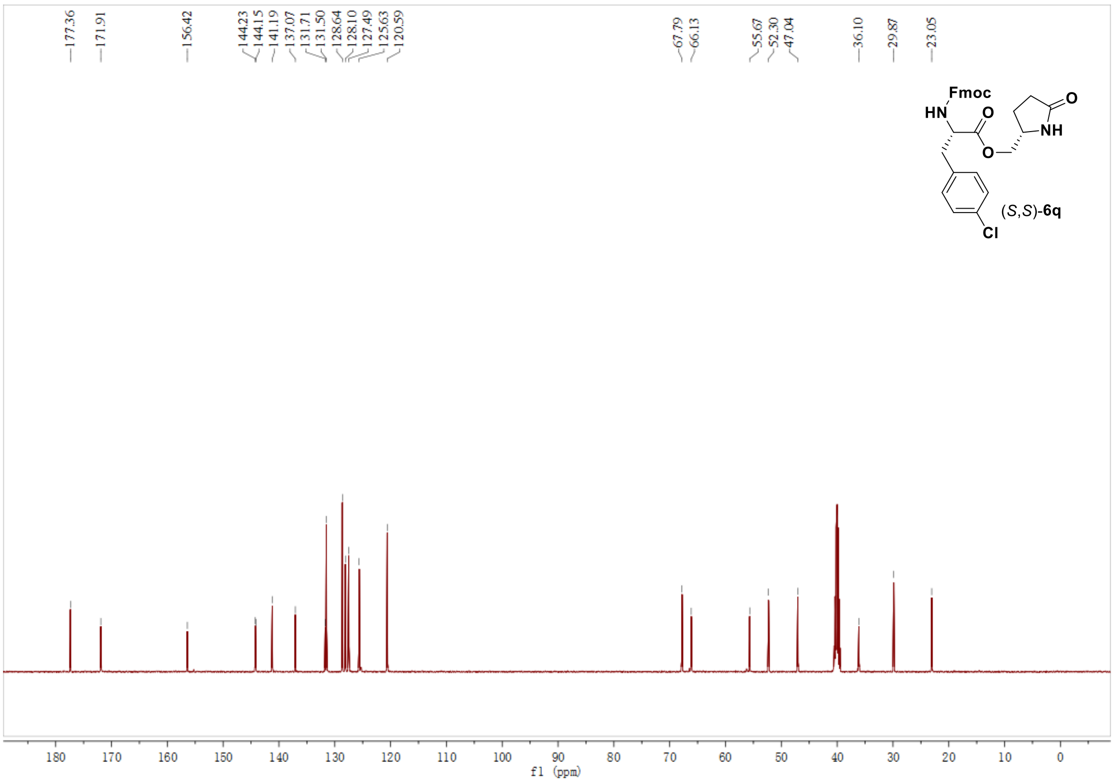

500 MHz, DMSO-d_6, ^{1}H NMR

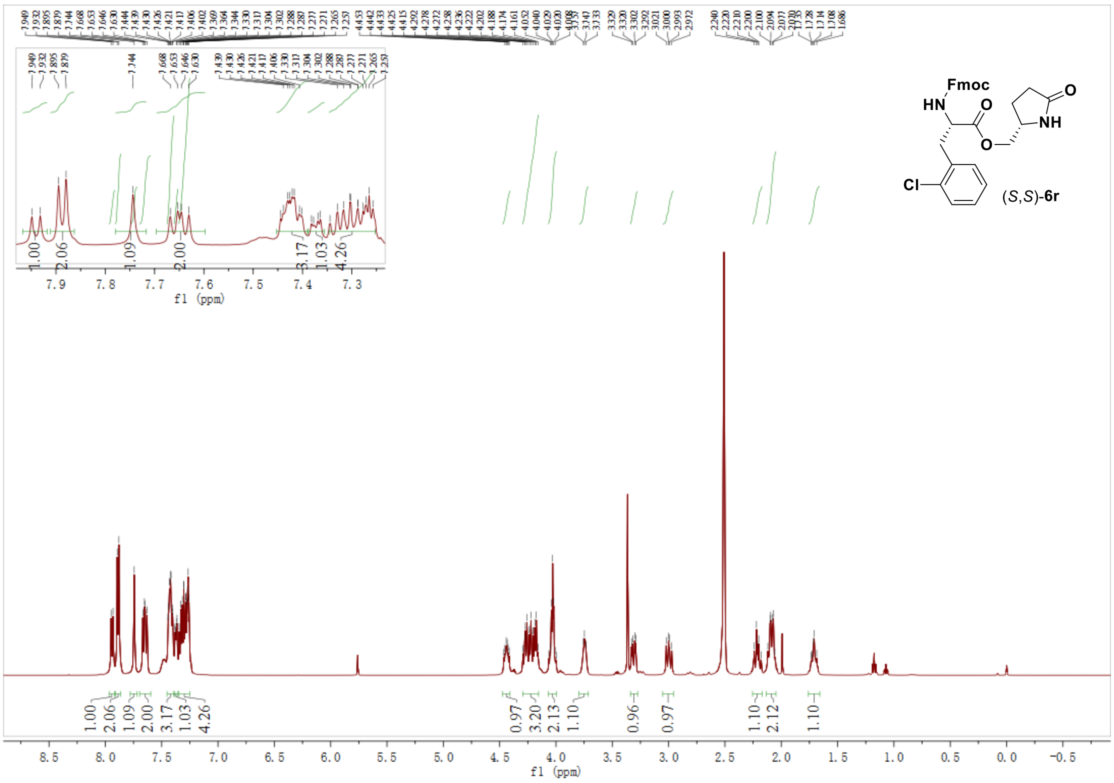

125 MHz, DMSO-d_6, ^{13}C NMR

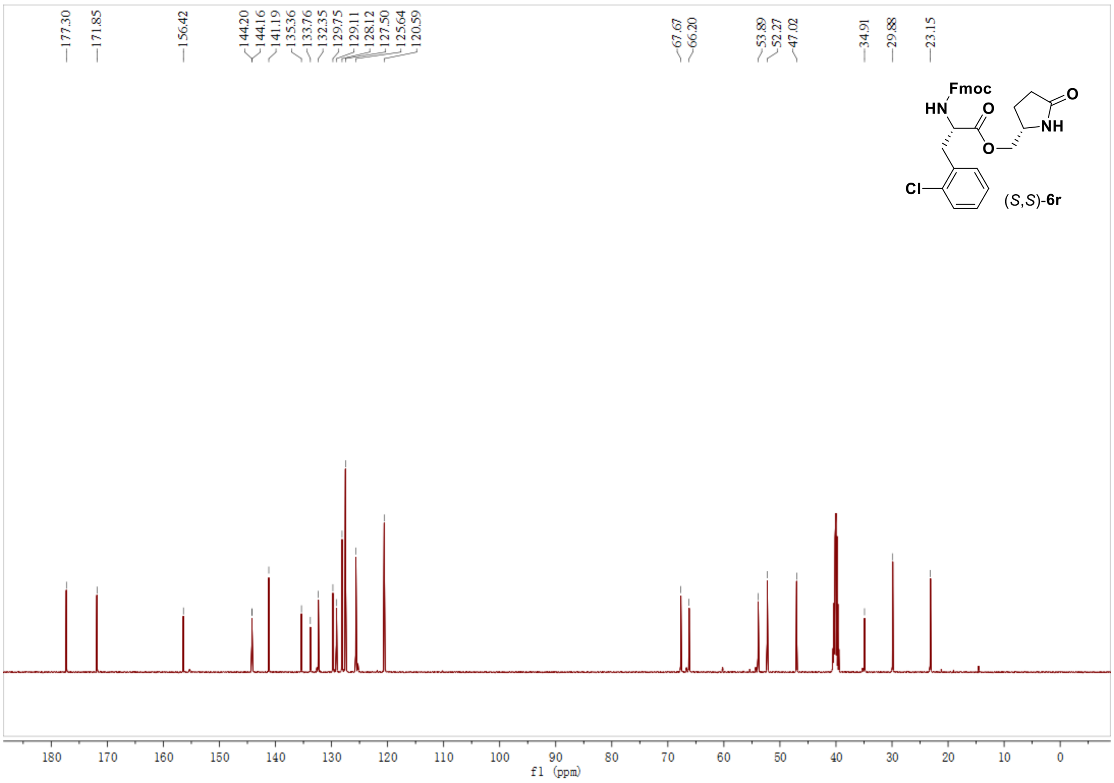

500 MHz, CDCl$_3$, ^{1}H NMR

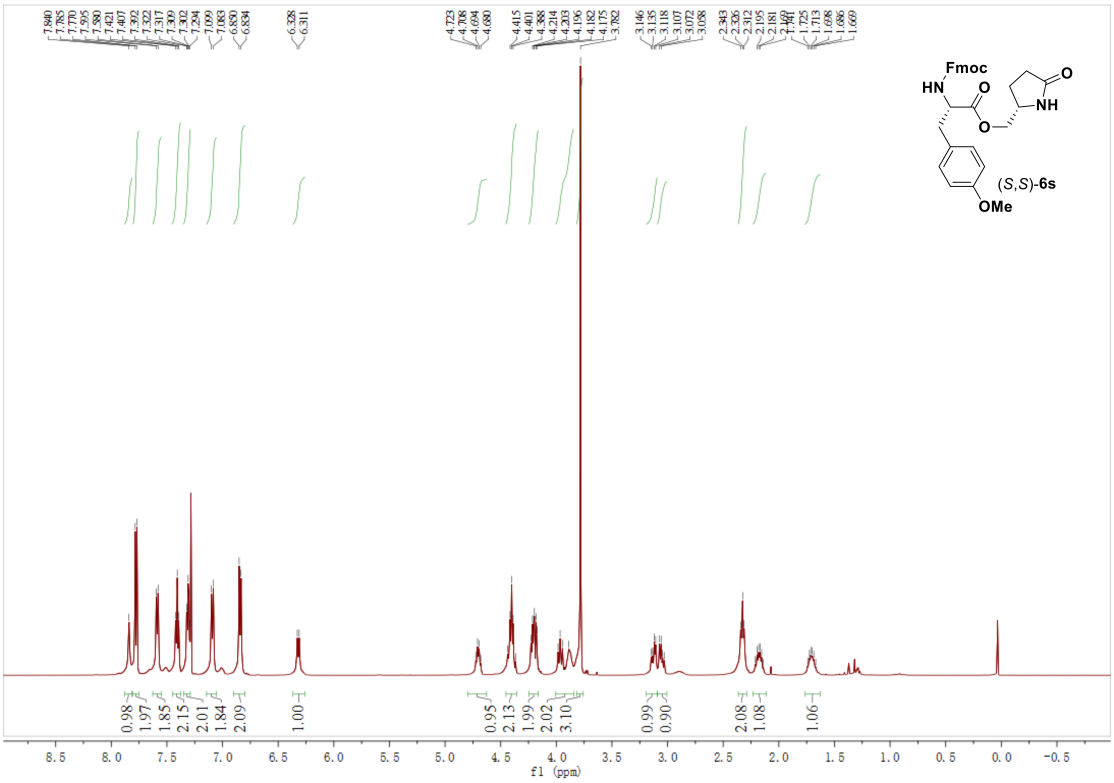

125 MHz, CDCl$_3$, ^{13}C NMR

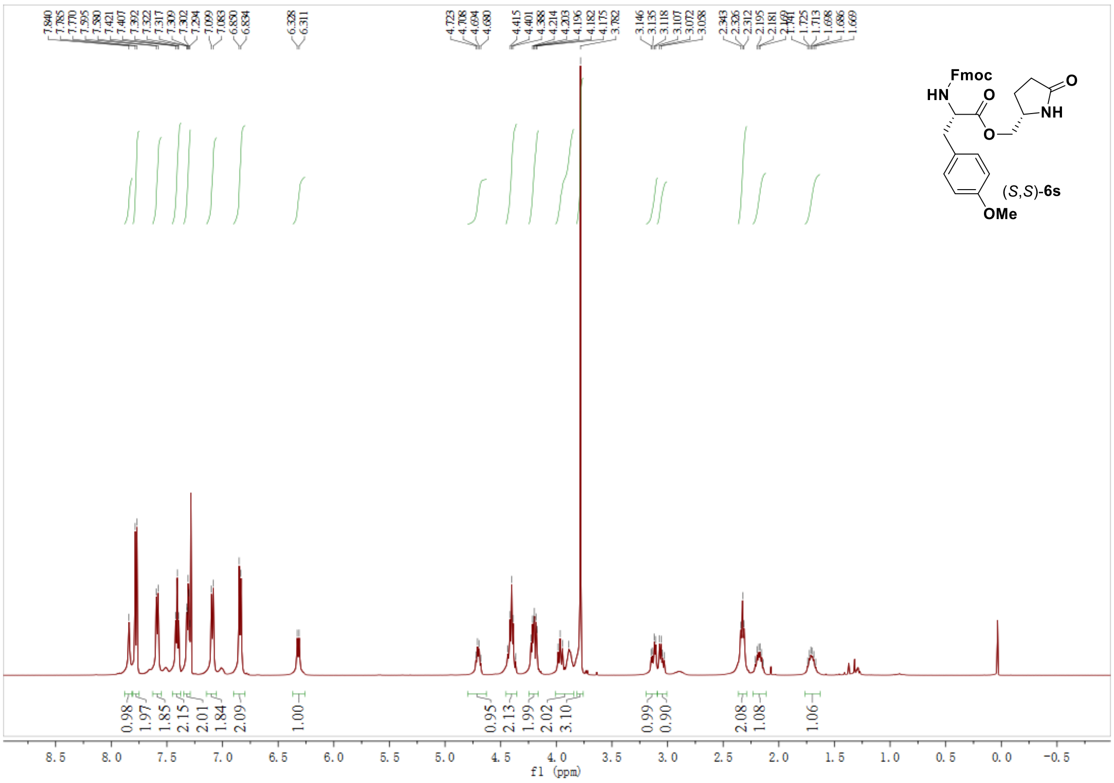

500 MHz, DMSO-d_6, ^{1}H NMR

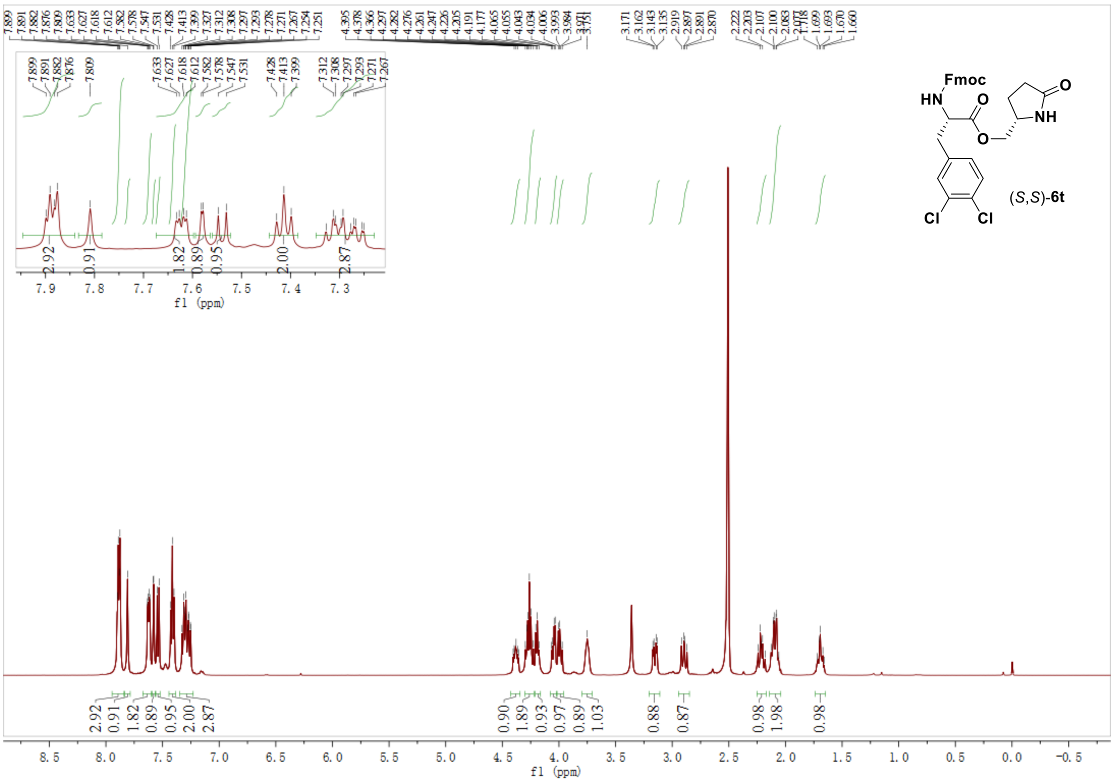

125 MHz, DMSO-d_6, ^{13}C NMR

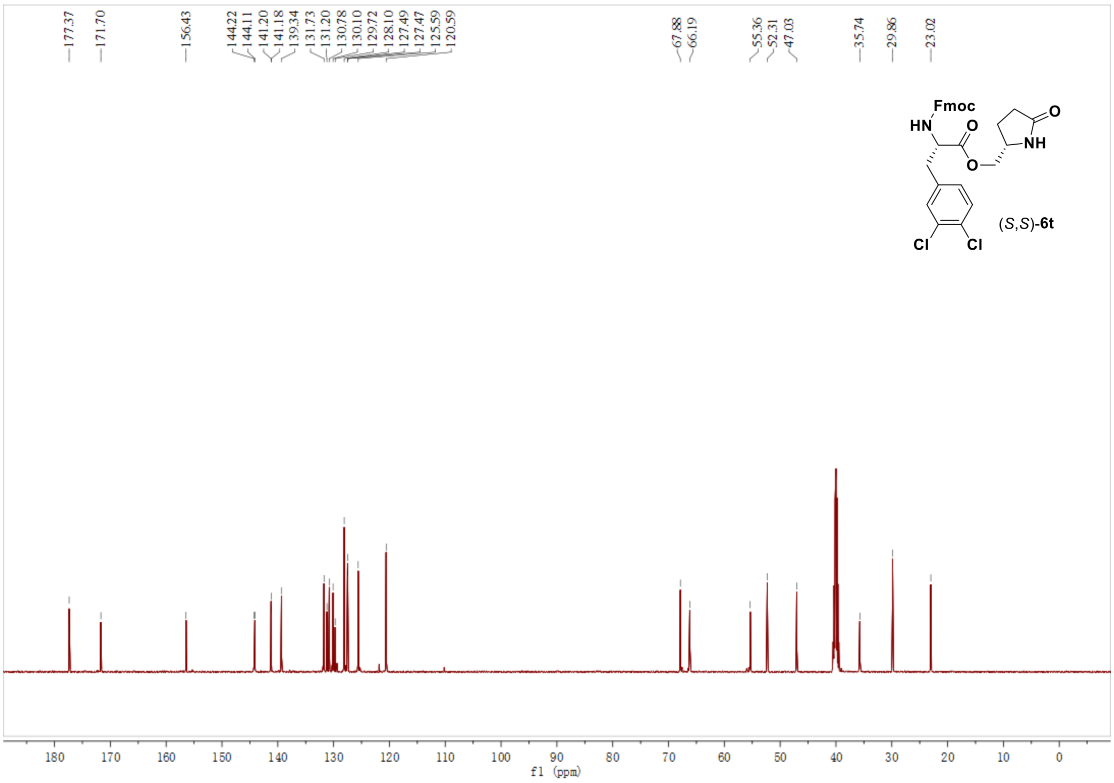

500 MHz, CDCl3, 1H NMR

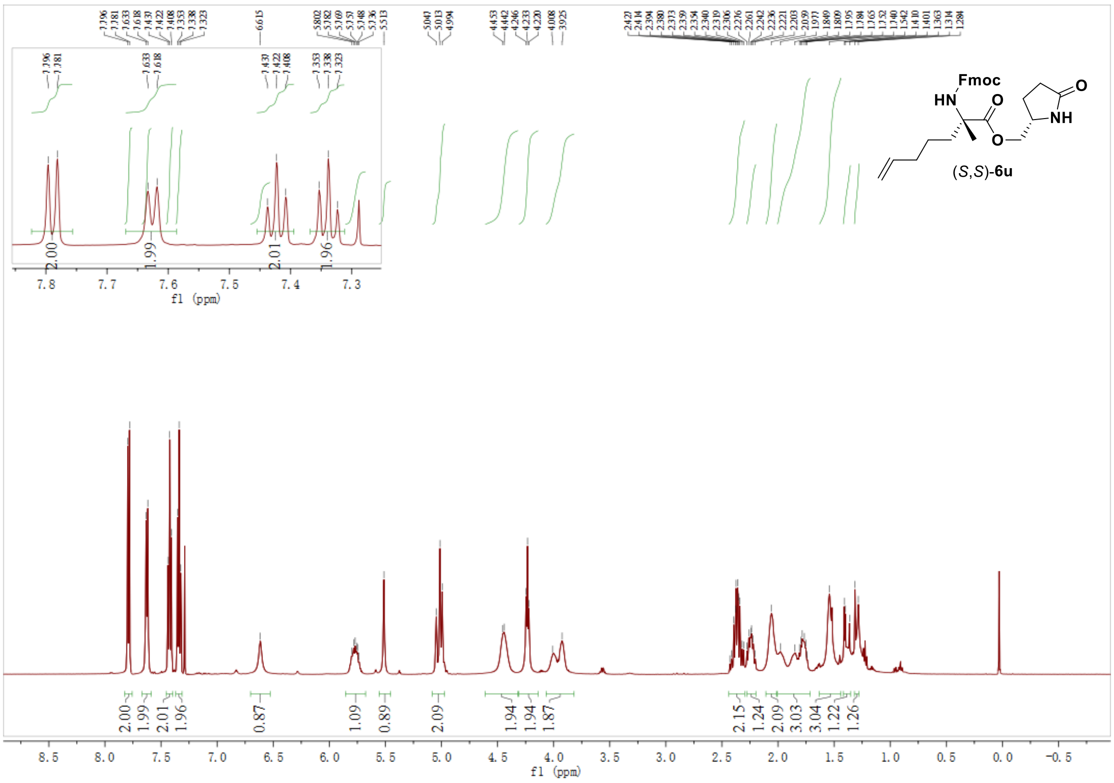

125 MHz, CDCl3, 13C NMR

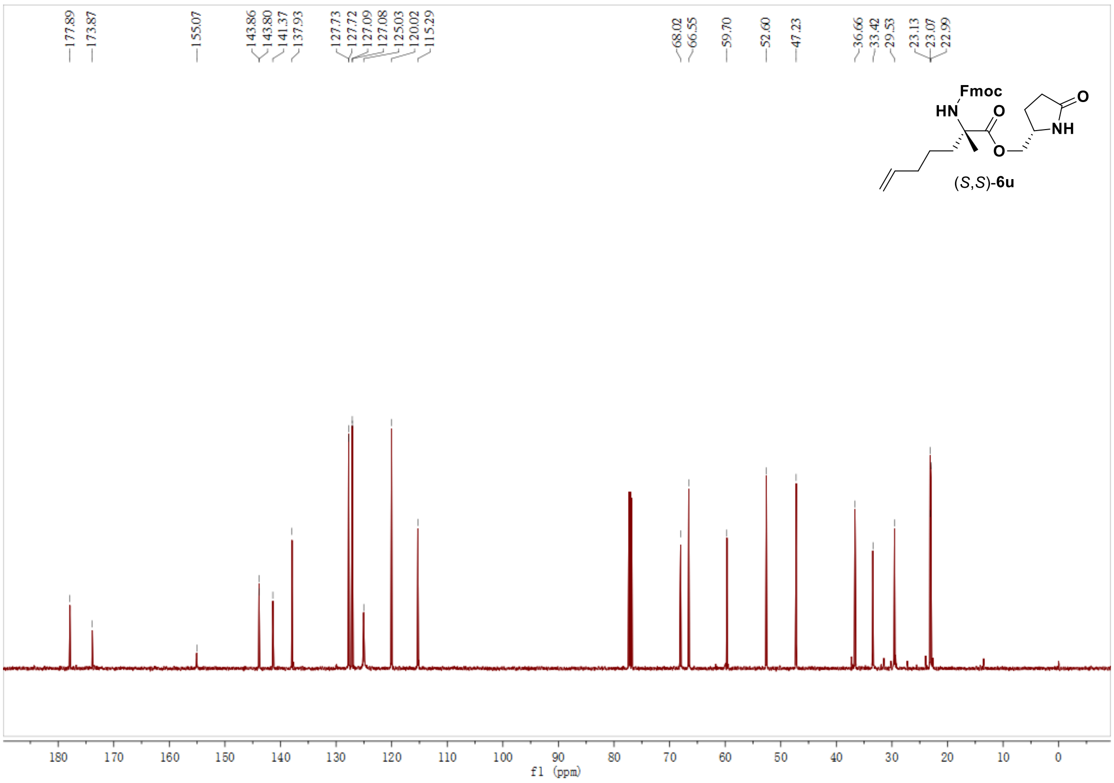

500 MHz, $CDCl_3$, ^1H NMR

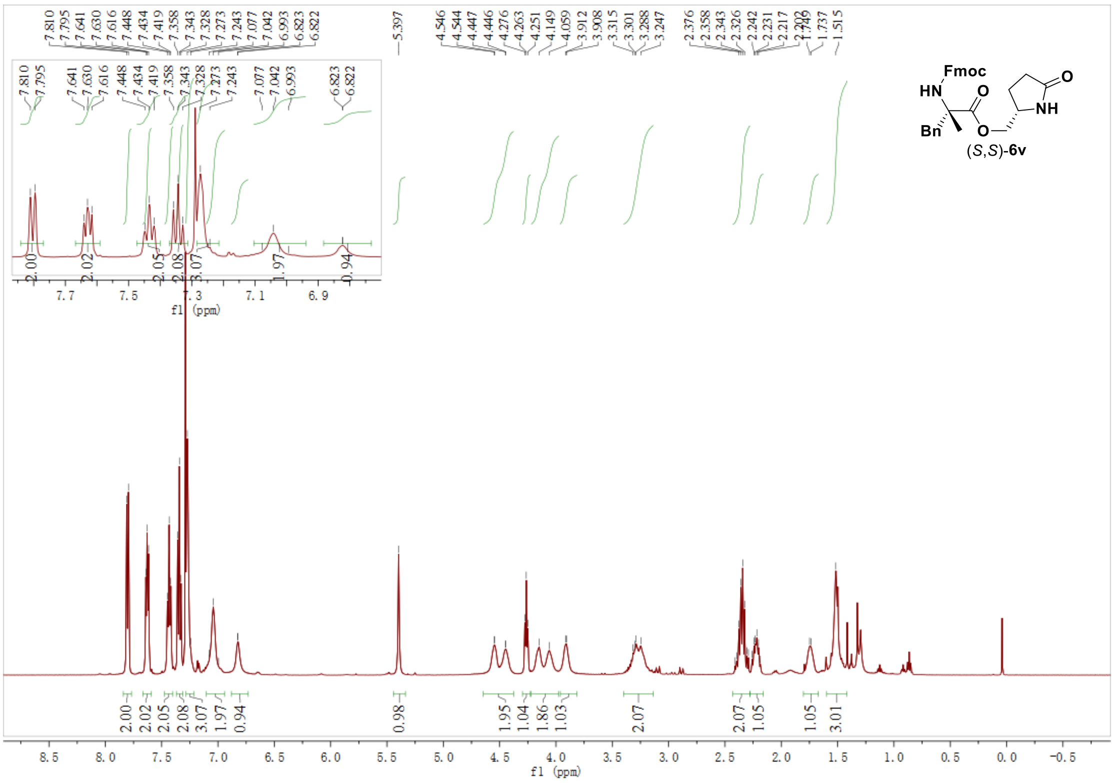

125 MHz, $CDCl_3$, ^{13}C NMR

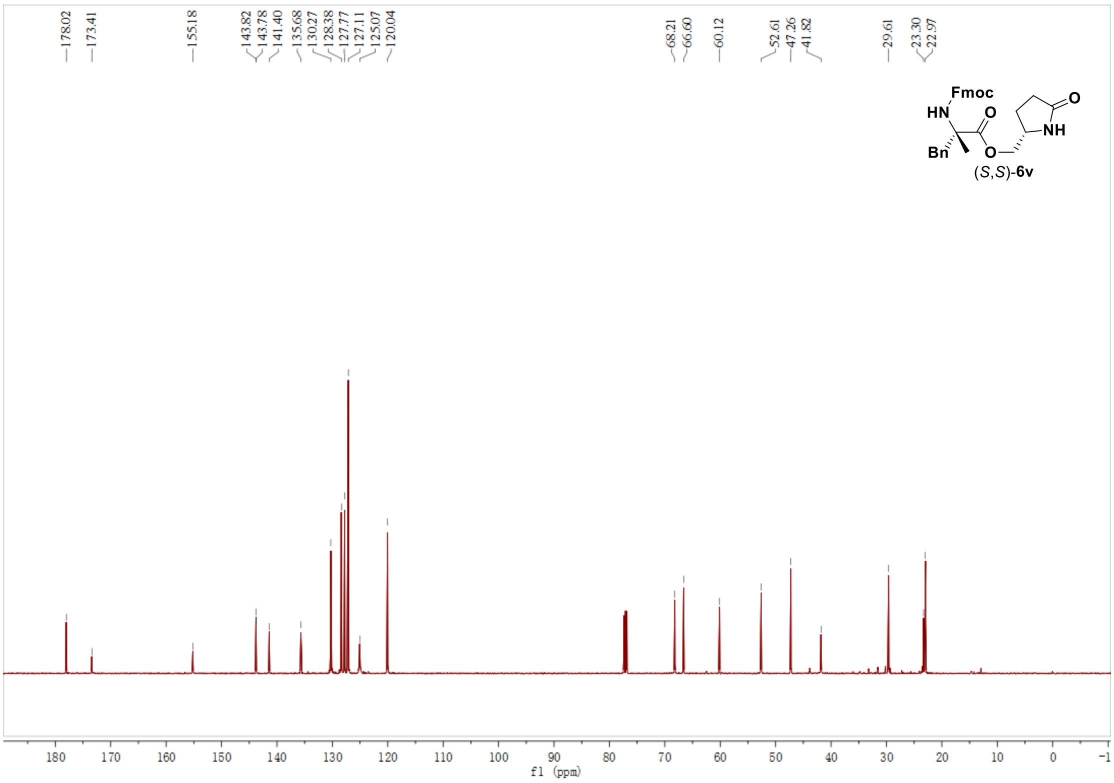

500 MHz, DMSO-d_6, ^{1}H NMR

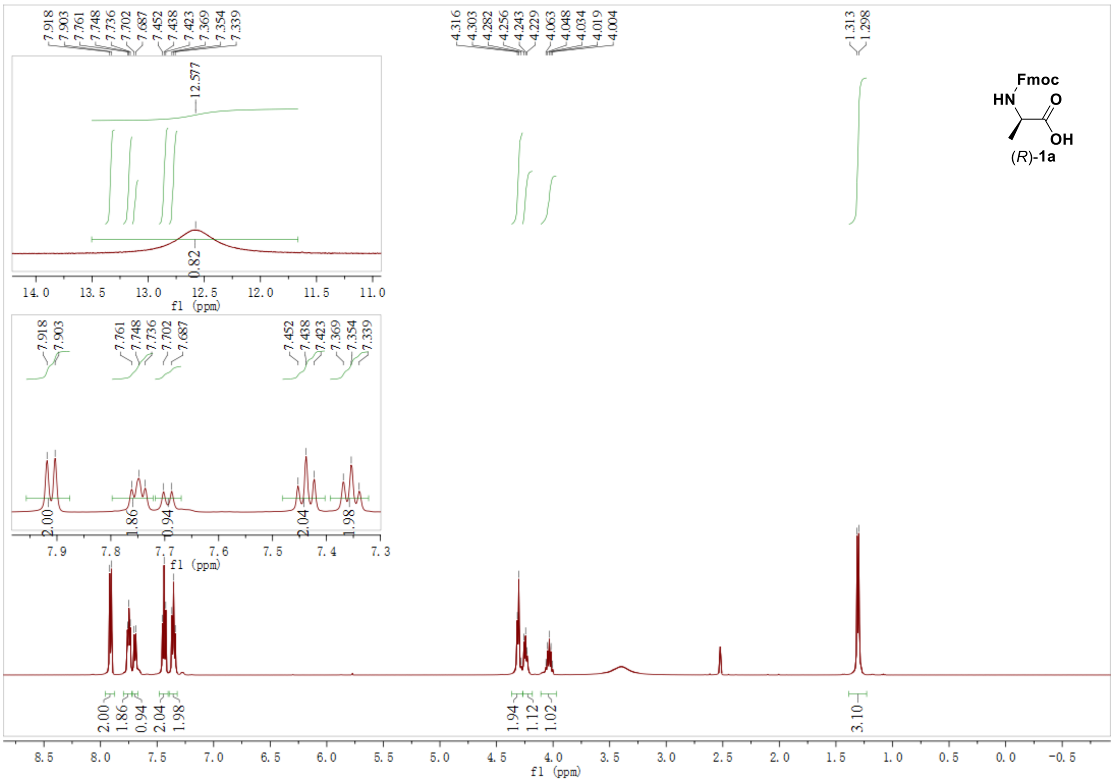

500 MHz, DMSO-d_6, ^{1}H NMR

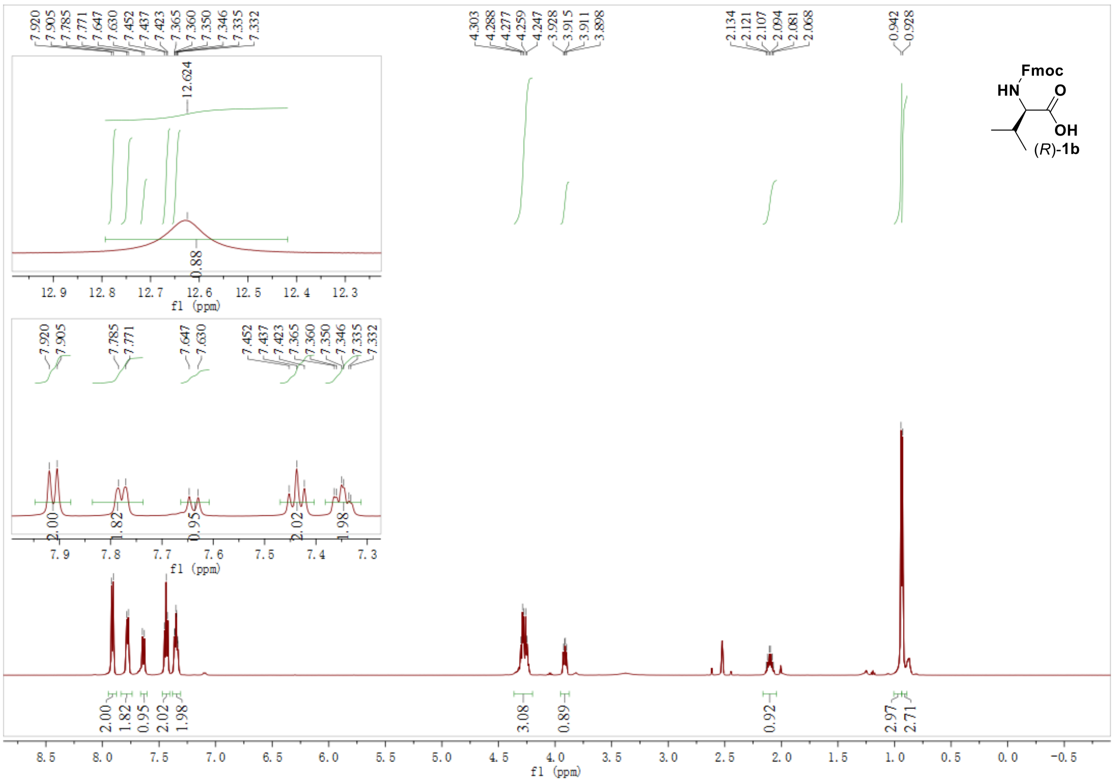

500 MHz, DMSO-d_6, ^1H NMR

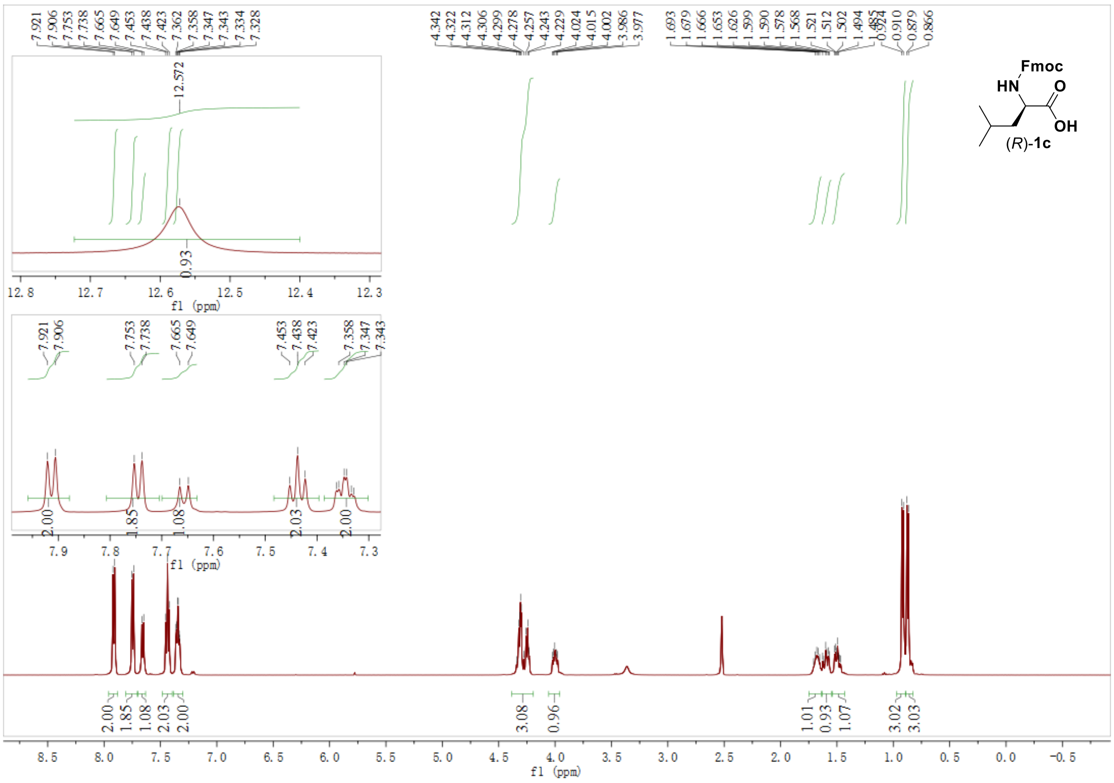

500 MHz, DMSO-d_6, ^1H NMR

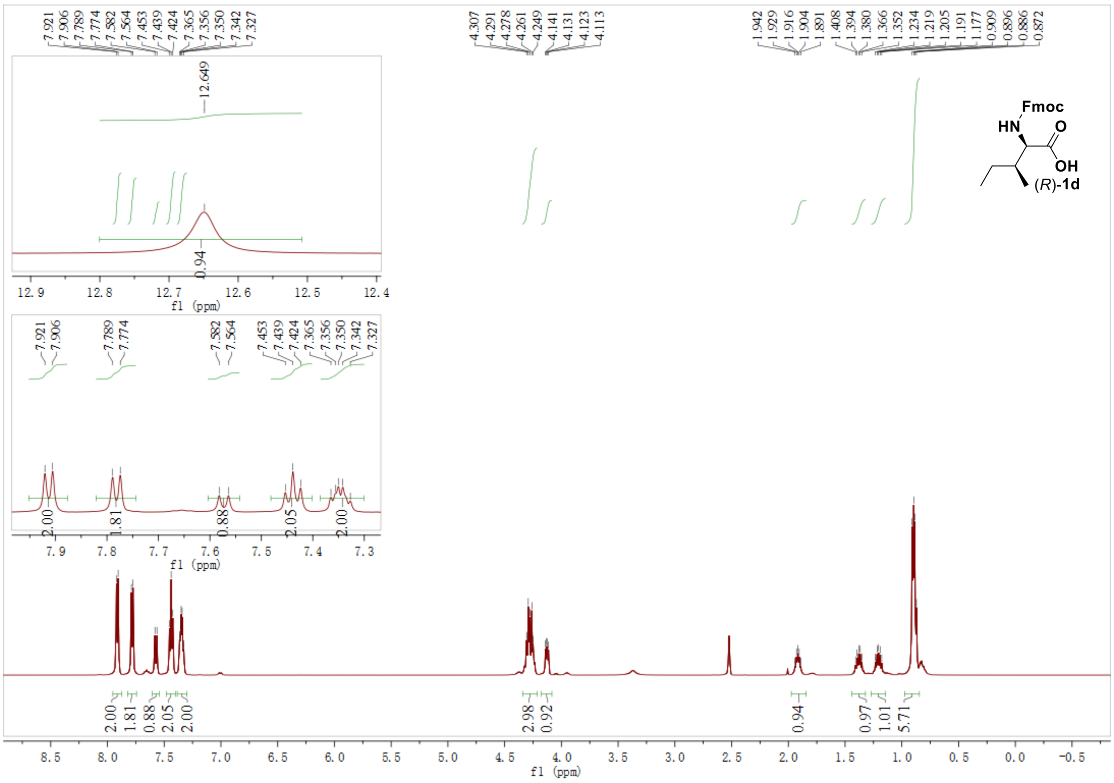

500 MHz, DMSO-d_6, ^{1}H NMR

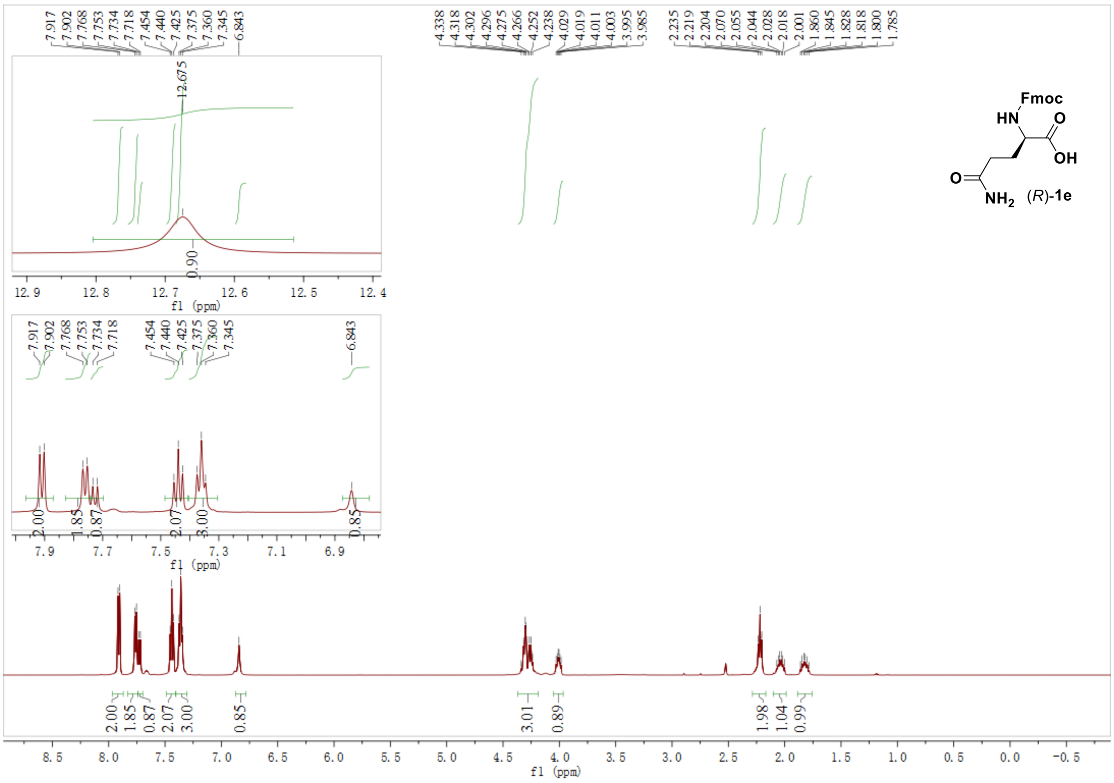

500 MHz, DMSO-d_6, ^{1}H NMR

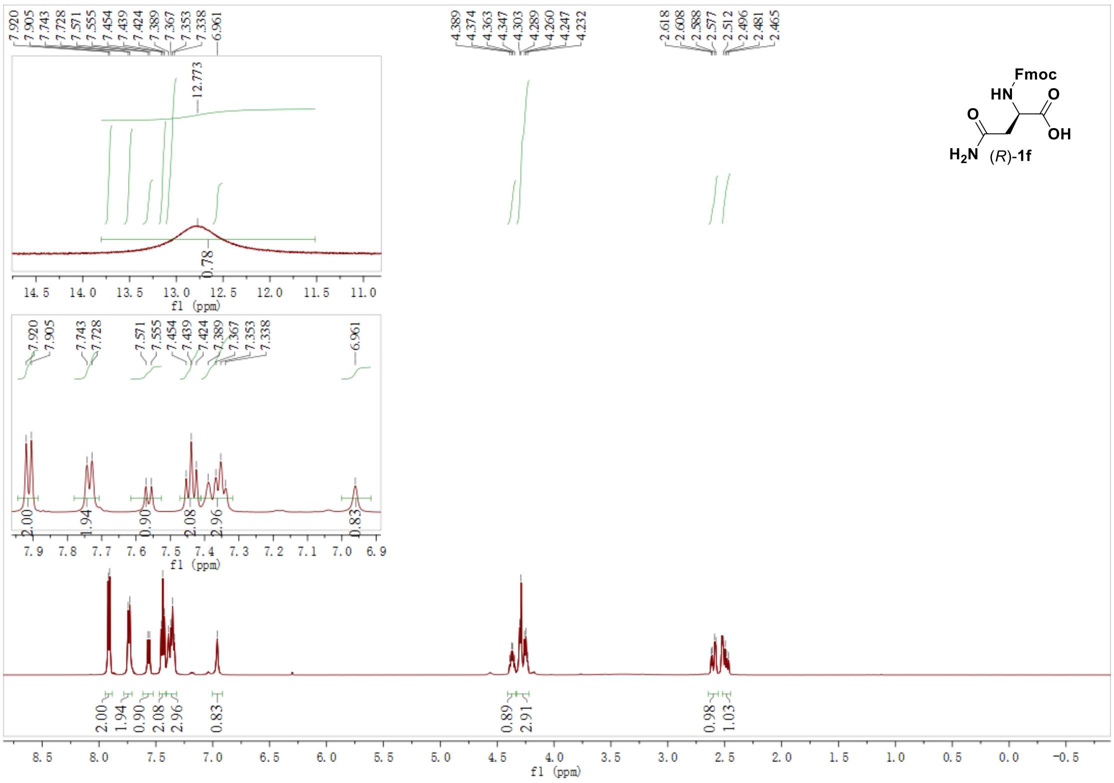

500 MHz, DMSO-d_6, ^{1}H NMR

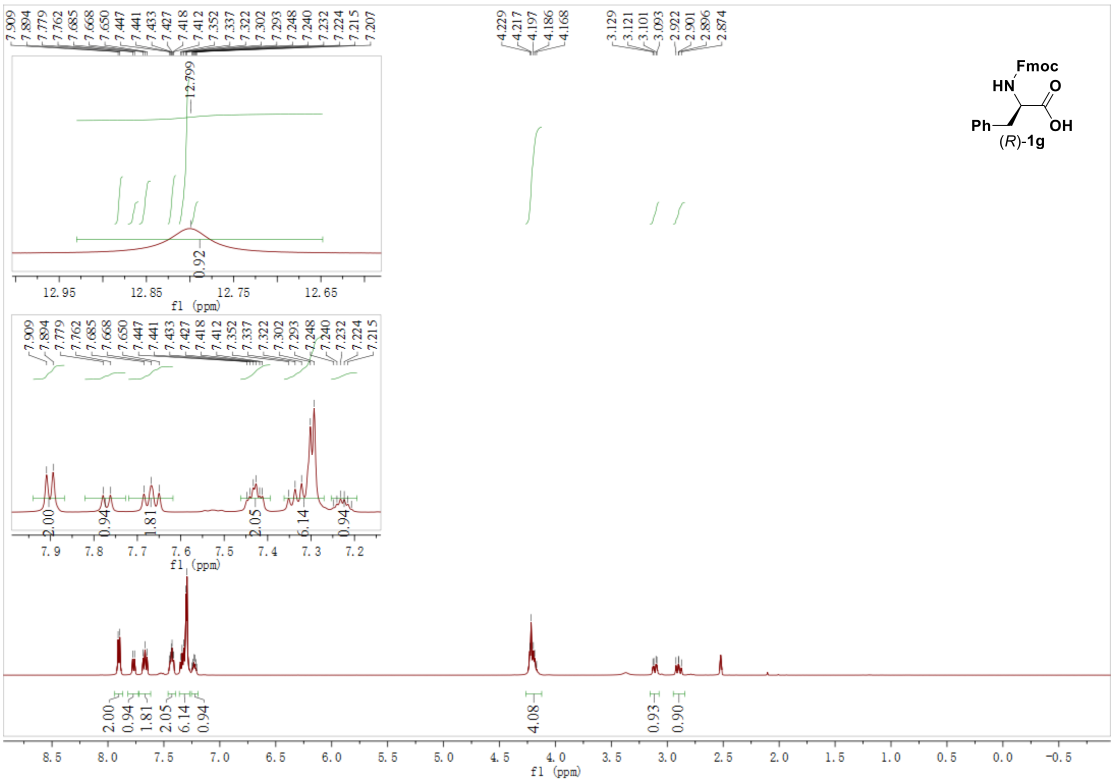

500 MHz, DMSO-d_6, ^{1}H NMR

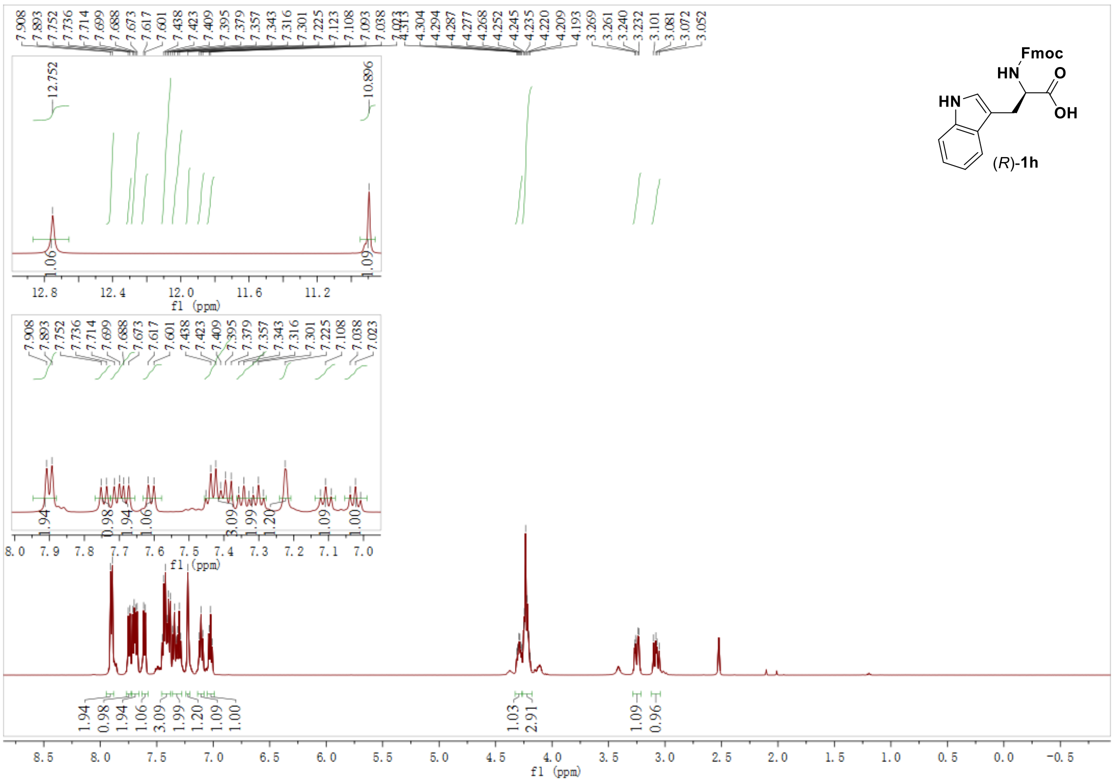

500 MHz, DMSO-d_6, ^{1}H NMR

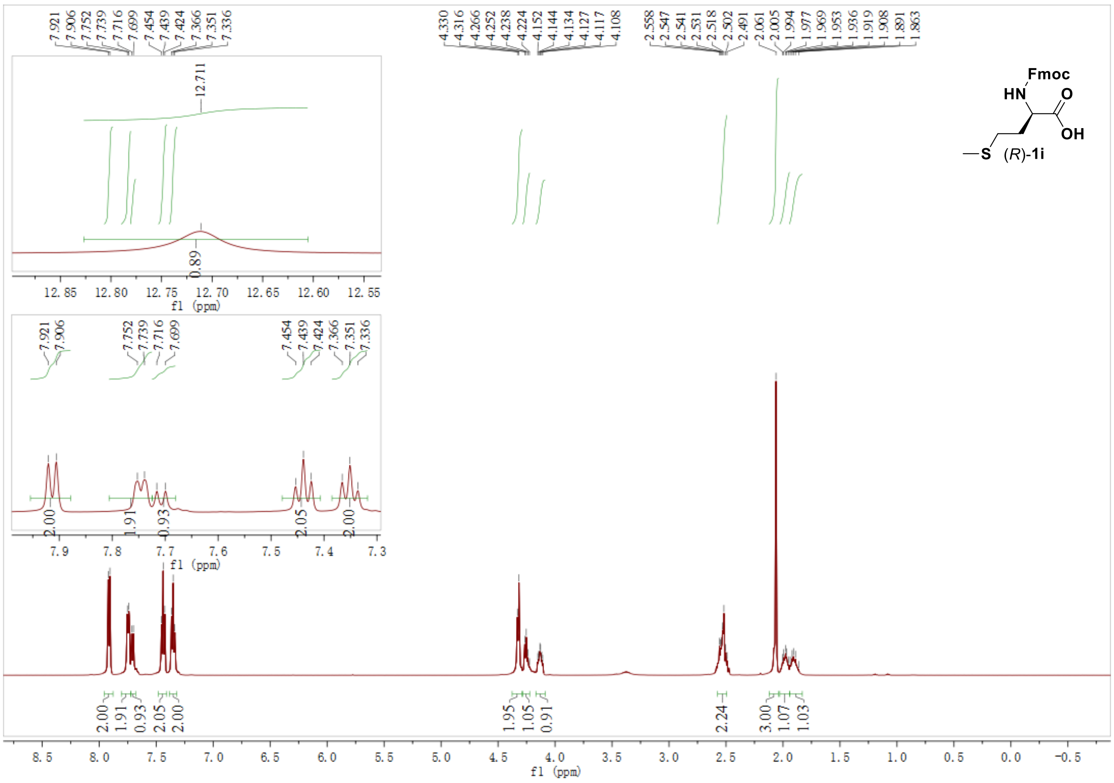

500 MHz, DMSO-d_6, ^{1}H NMR

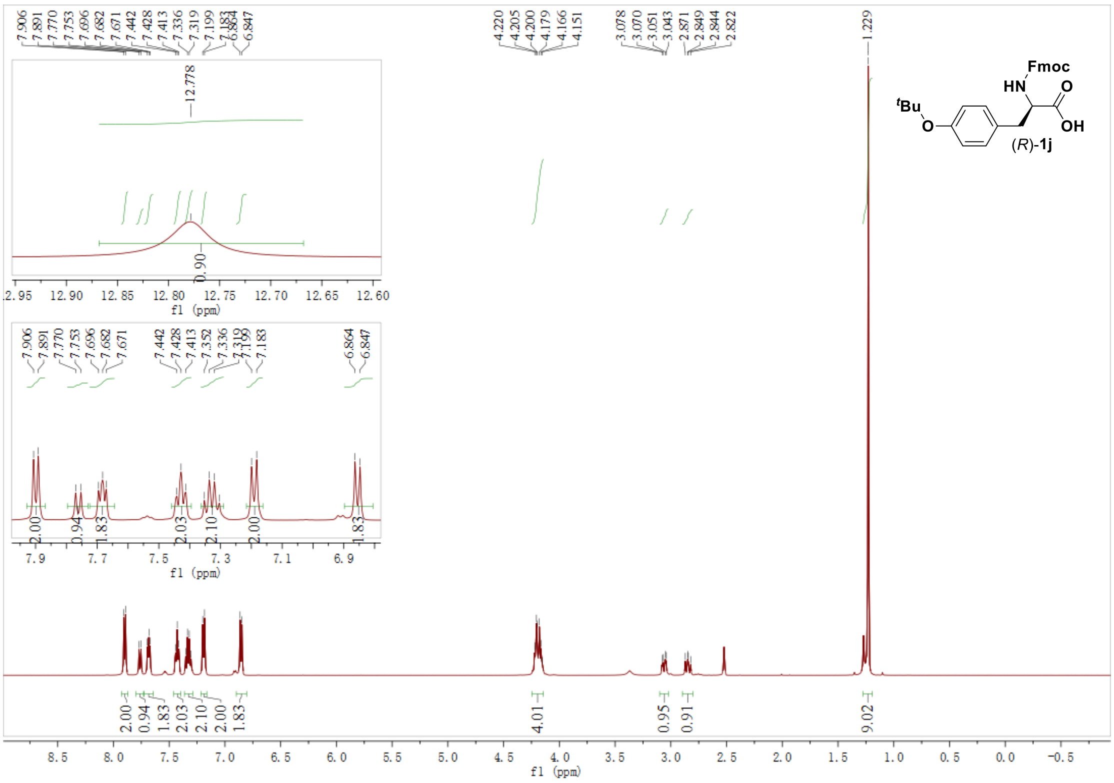

500 MHz, DMSO-d_6, ^{1}H NMR

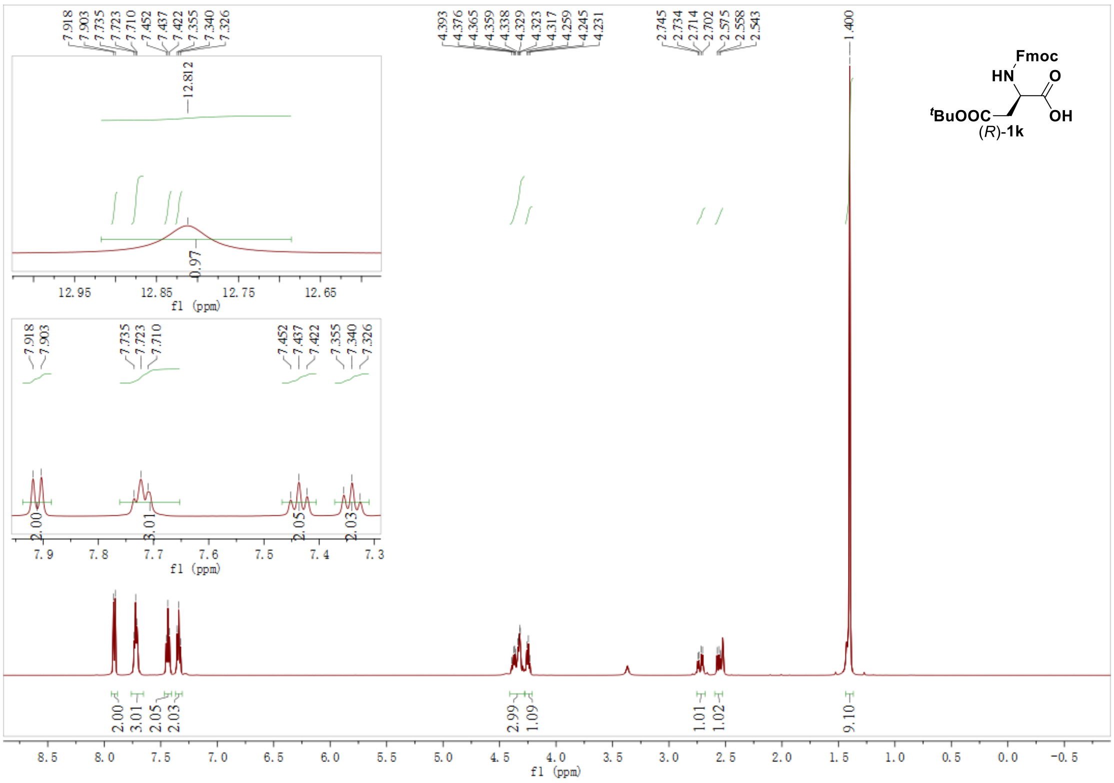

500 MHz, DMSO-d_6, ^{1}H NMR

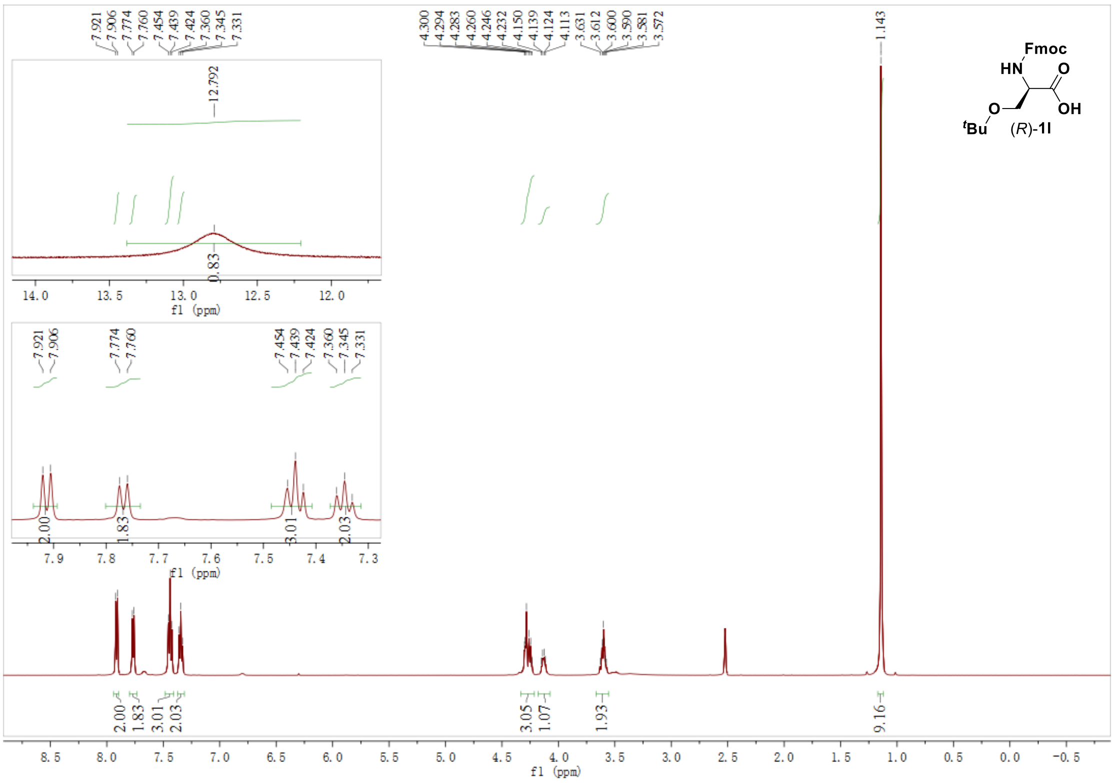

500 MHz, DMSO-d_6, ^{1}H NMR

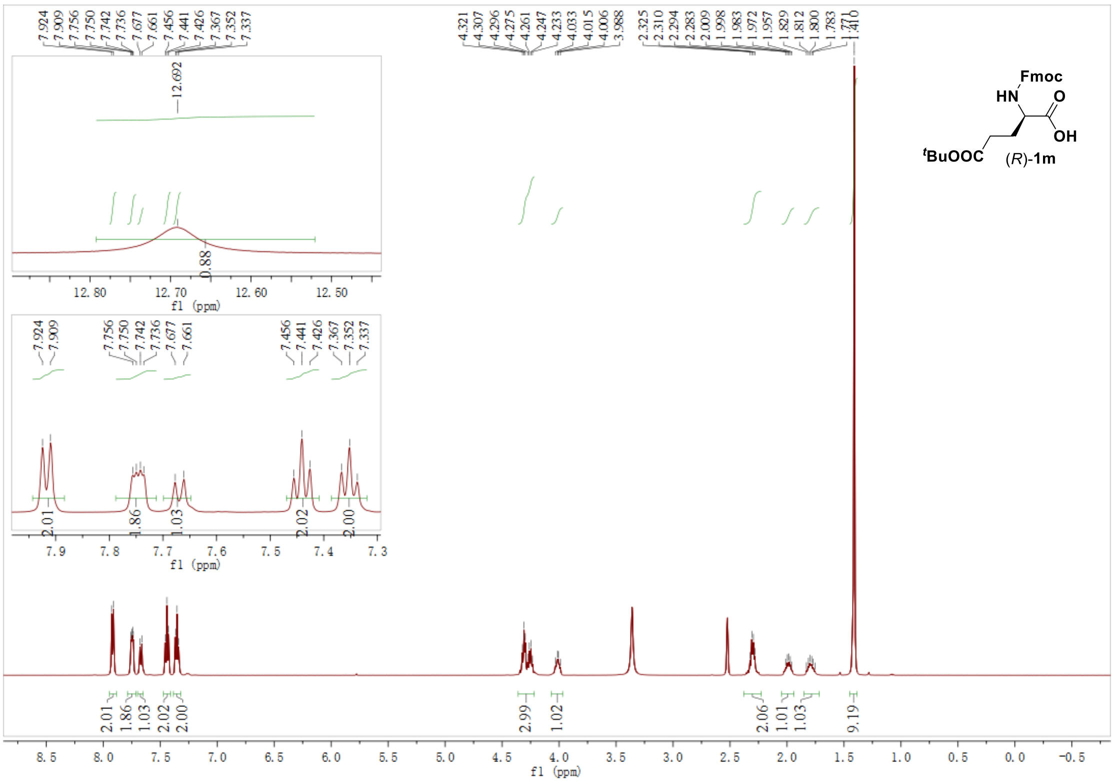

500 MHz, DMSO-d_6, ^{1}H NMR

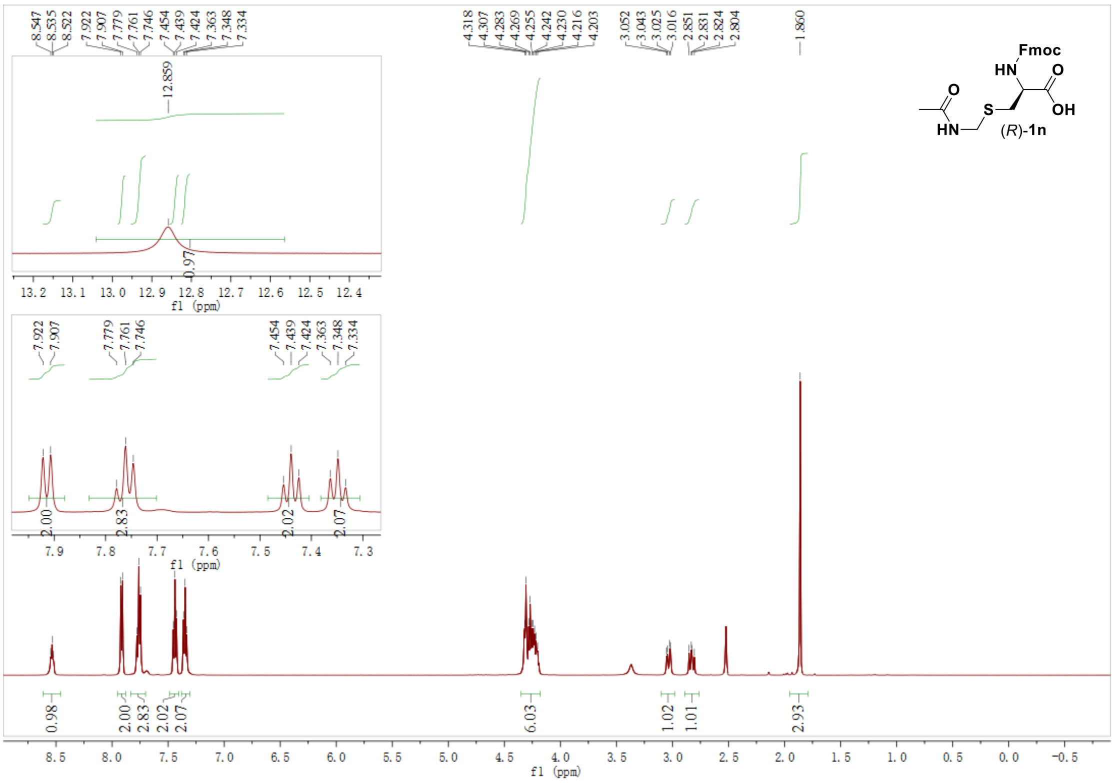

500 MHz, DMSO-d_6, ^{1}H NMR

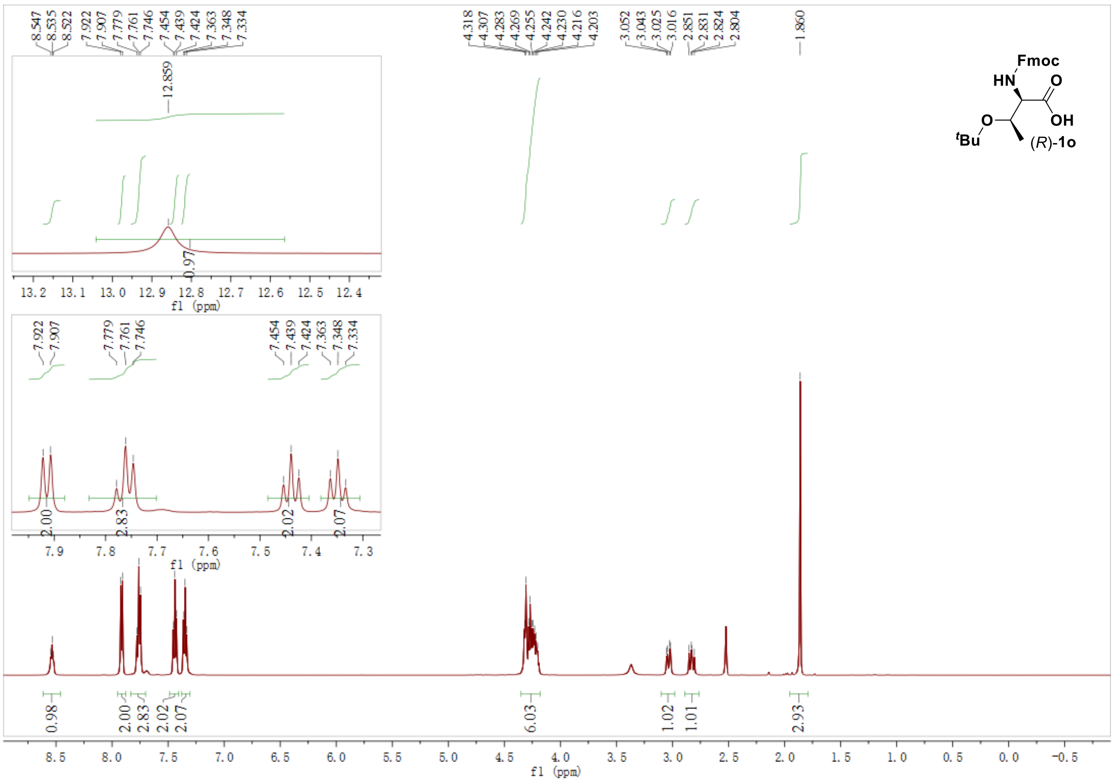

500 MHz, DMSO-d_6, ^{1}H NMR

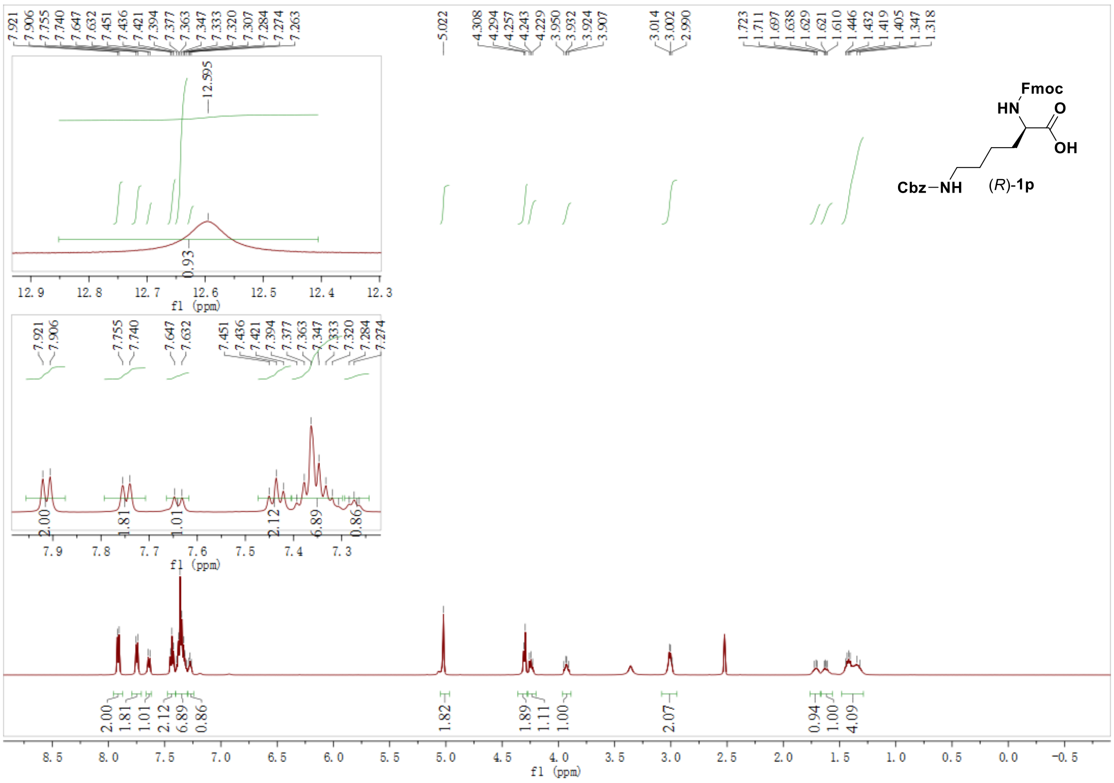

500 MHz, DMSO-d_6, ^{1}H NMR

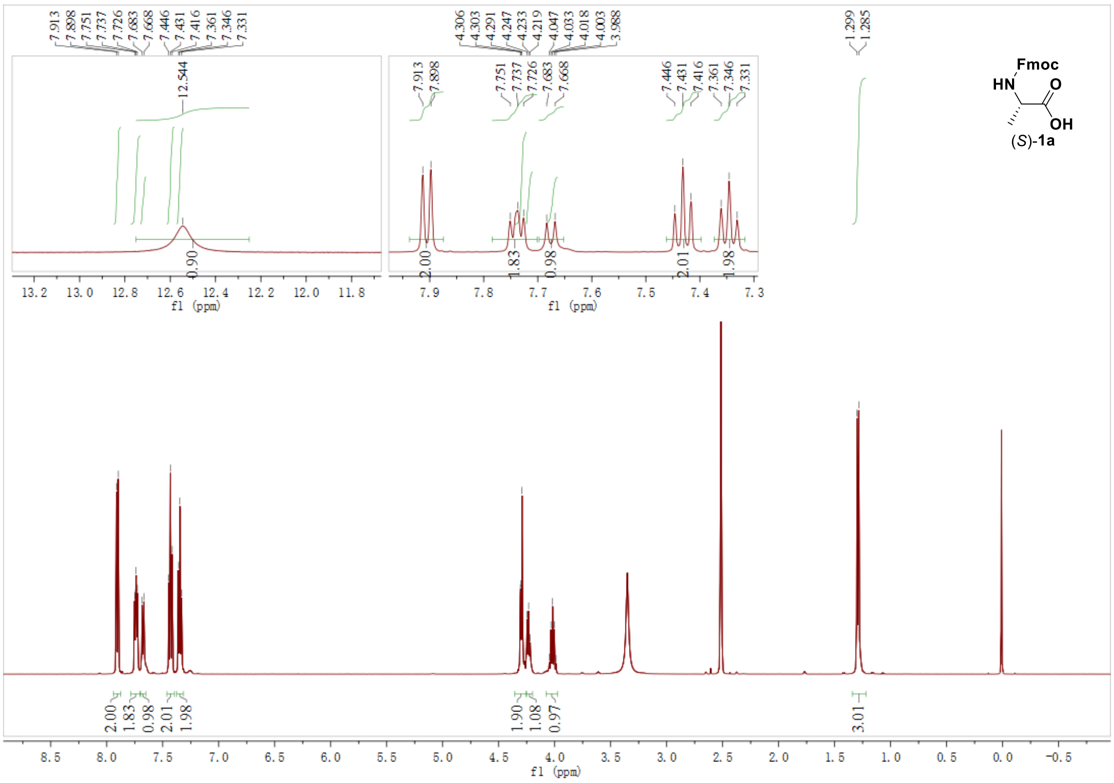

500 MHz, DMSO-d_6, ^{1}H NMR

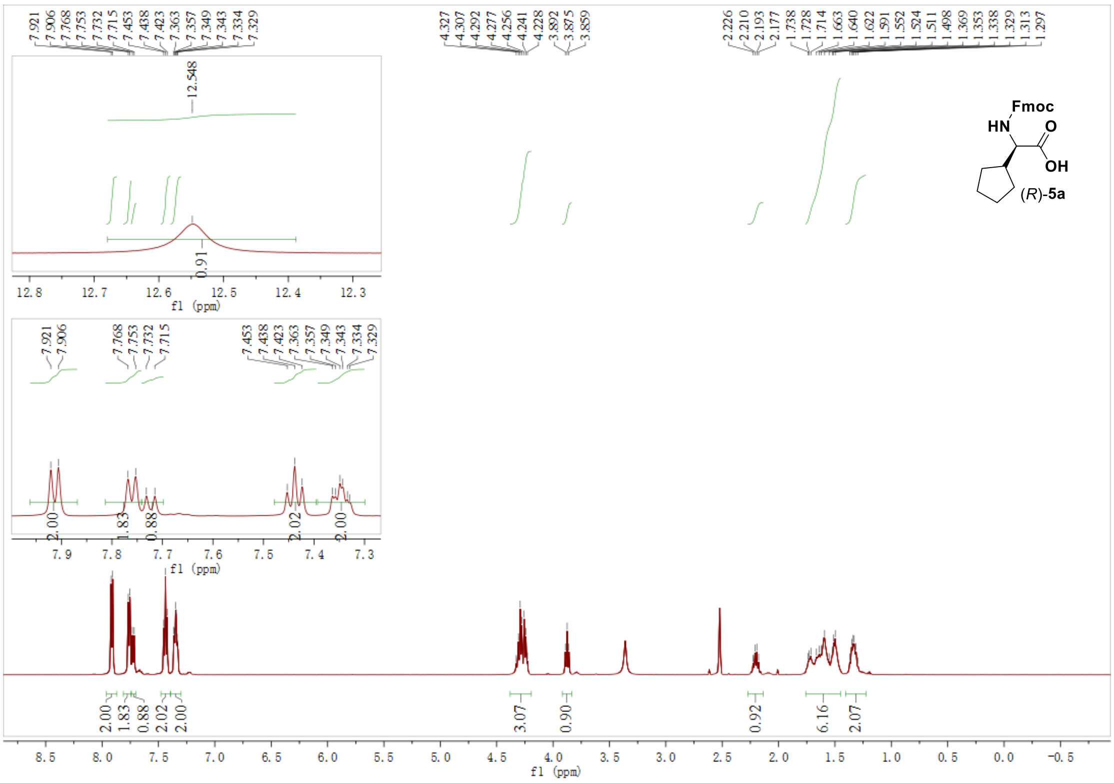

500 MHz, DMSO-d_6, ^{1}H NMR

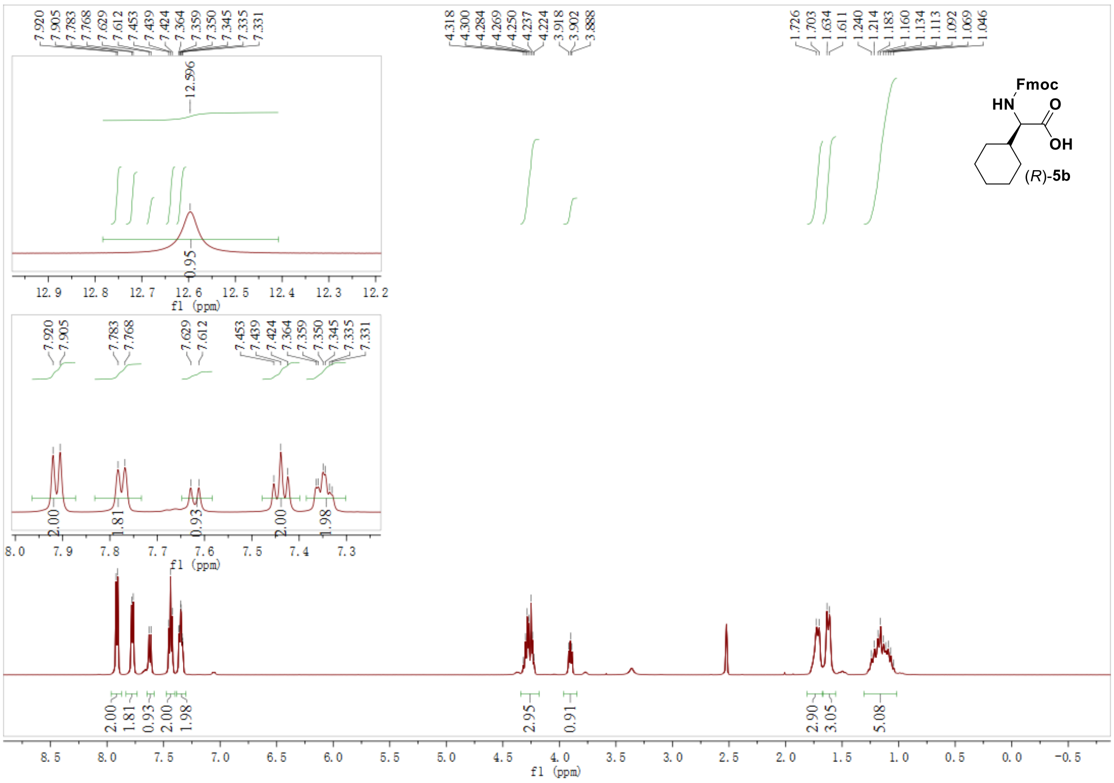

500 MHz, DMSO-d_6, ^{1}H NMR

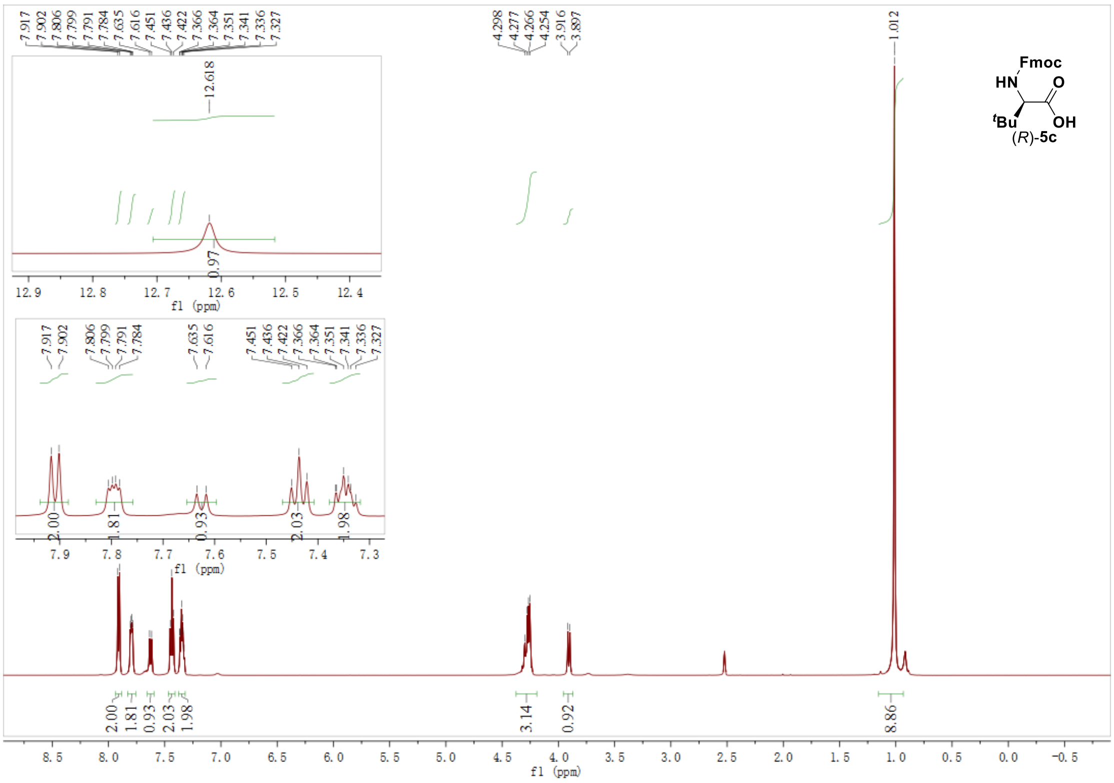

500 MHz, DMSO-d_6, ^1H NMR

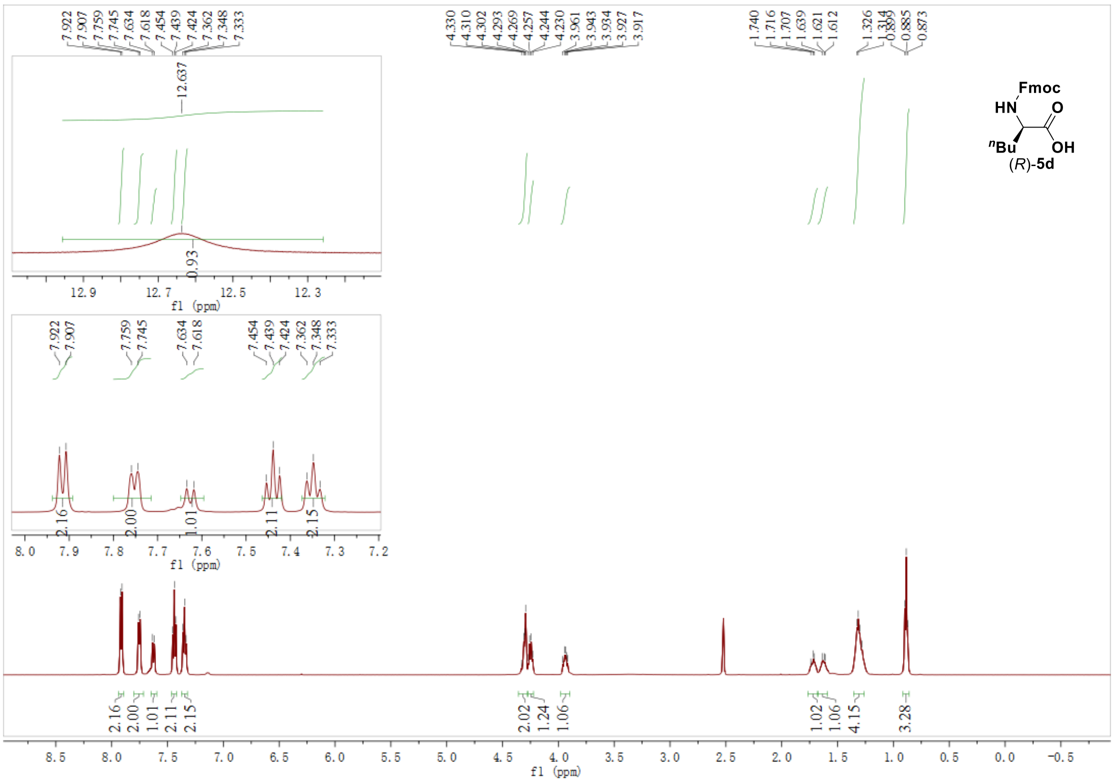

500 MHz, DMSO-d_6, ^1H NMR

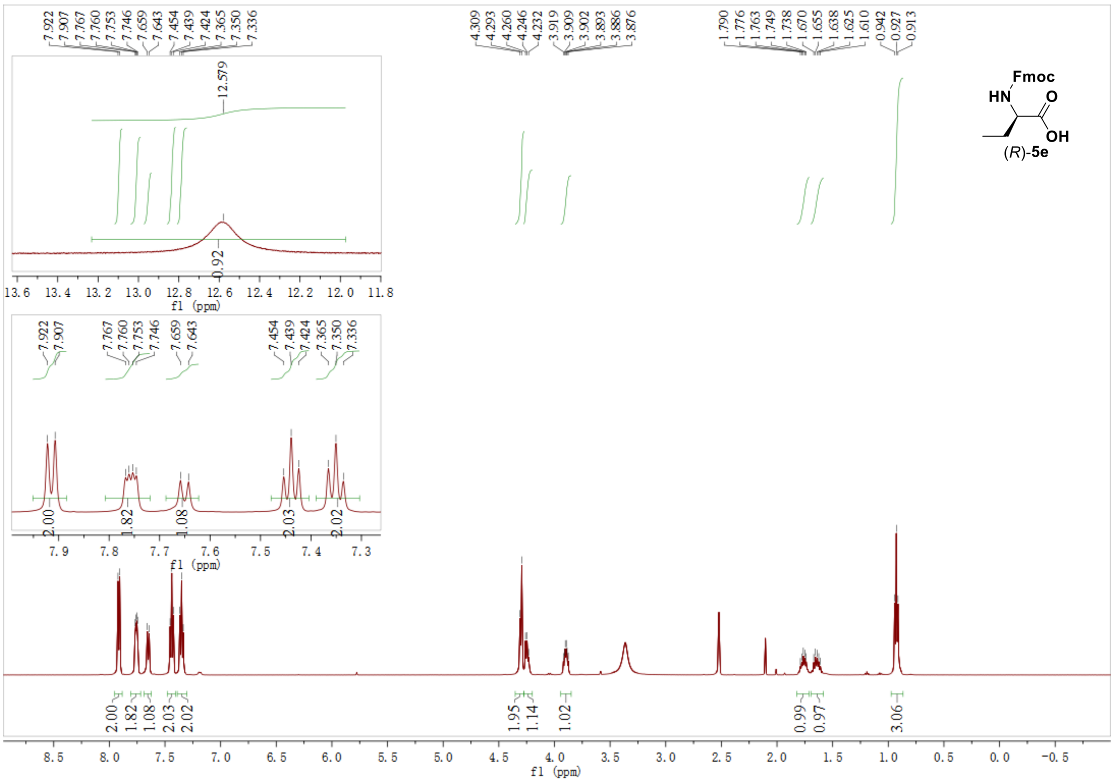

500 MHz, DMSO-d_6, ^{1}H NMR

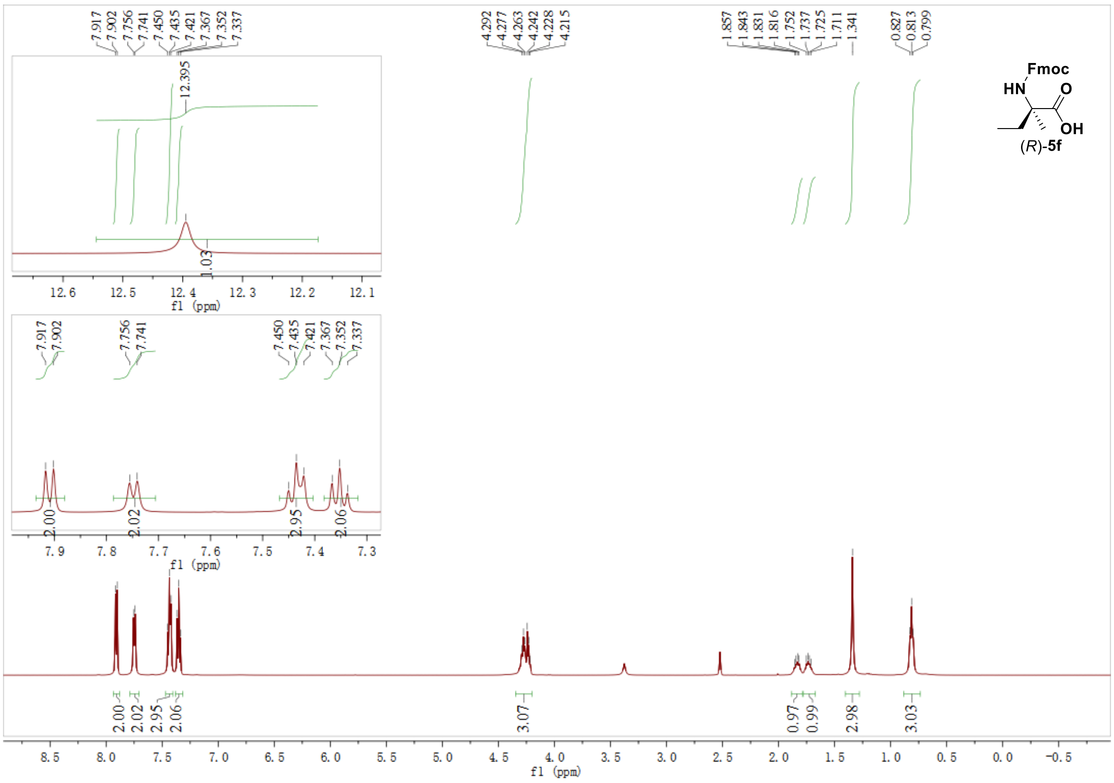

500 MHz, DMSO-d_6, ^{1}H NMR

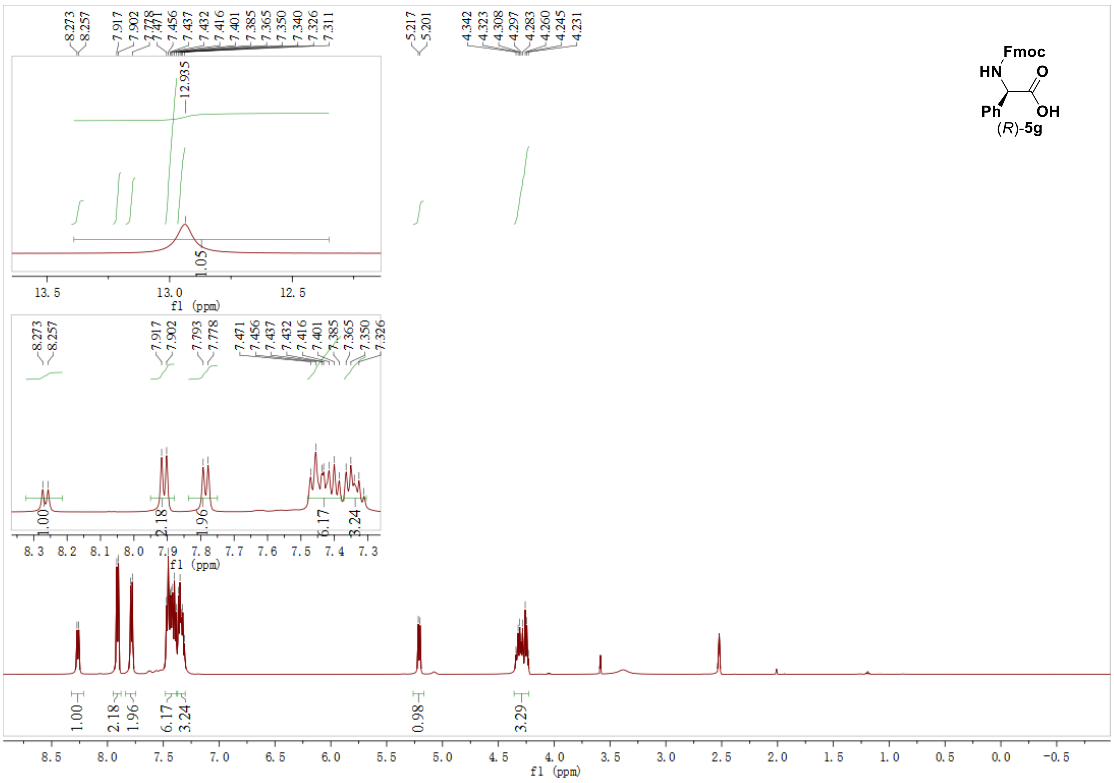

500 MHz, DMSO-d_6, ^{1}H NMR

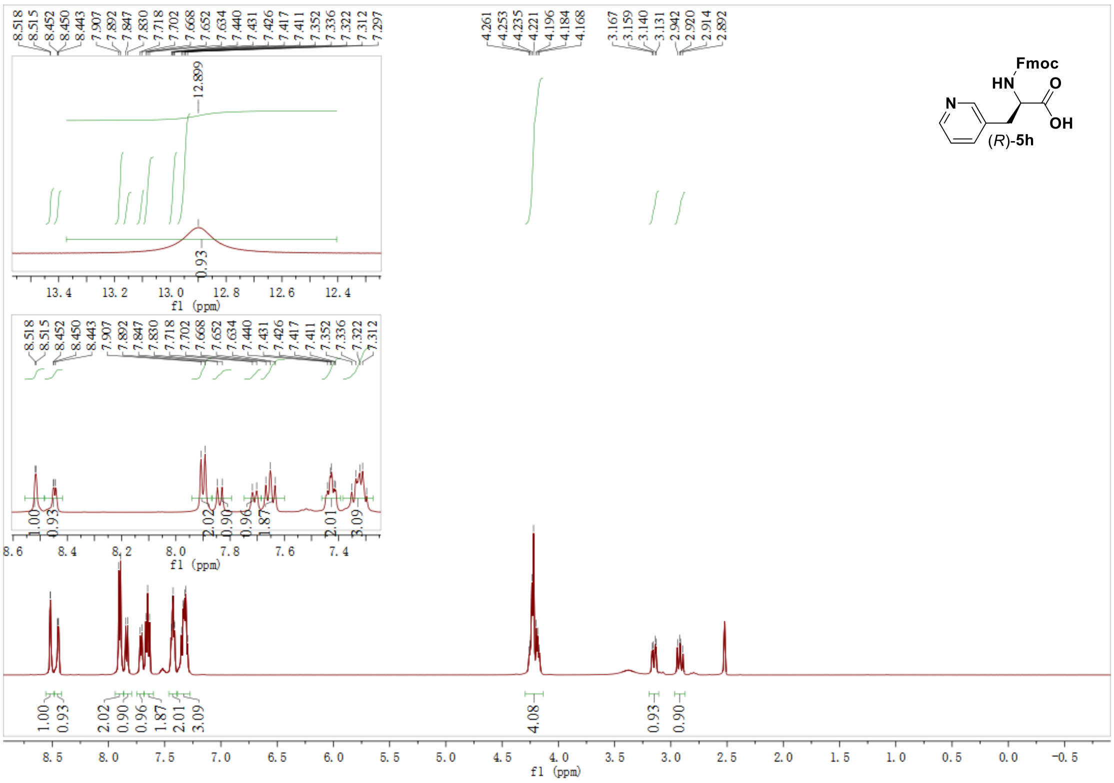

500 MHz, DMSO-d_6, ^{1}H NMR

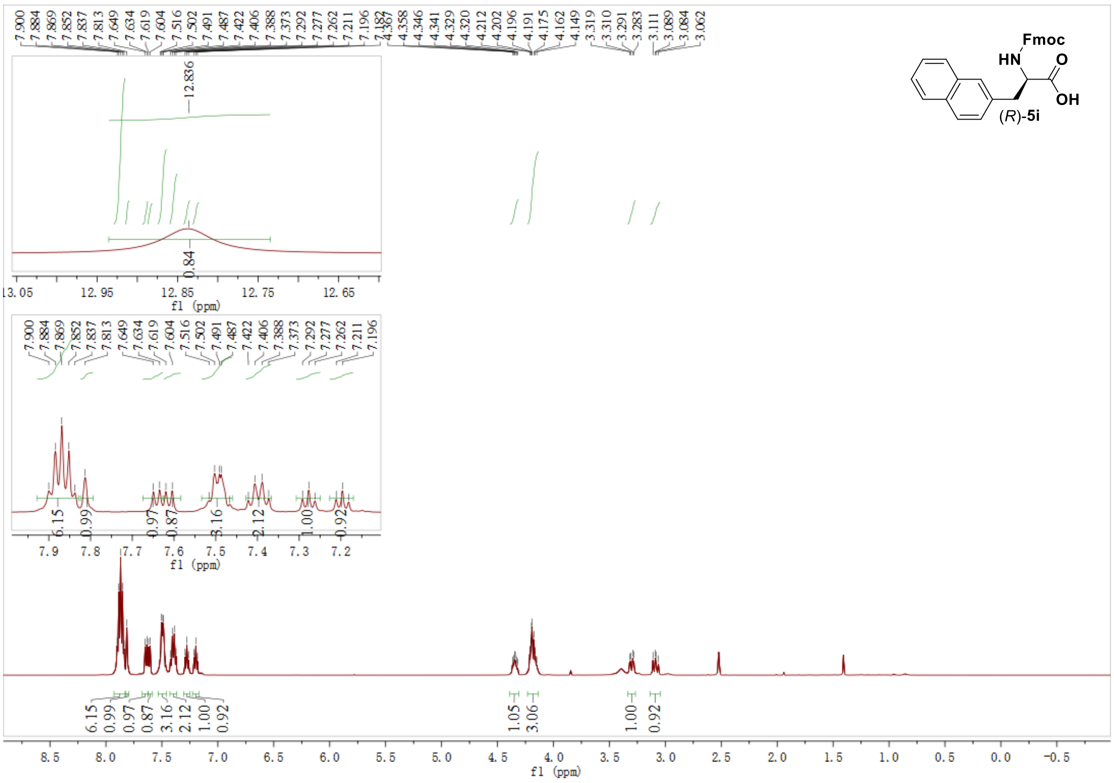

500 MHz, DMSO-d_6, ^{1}H NMR

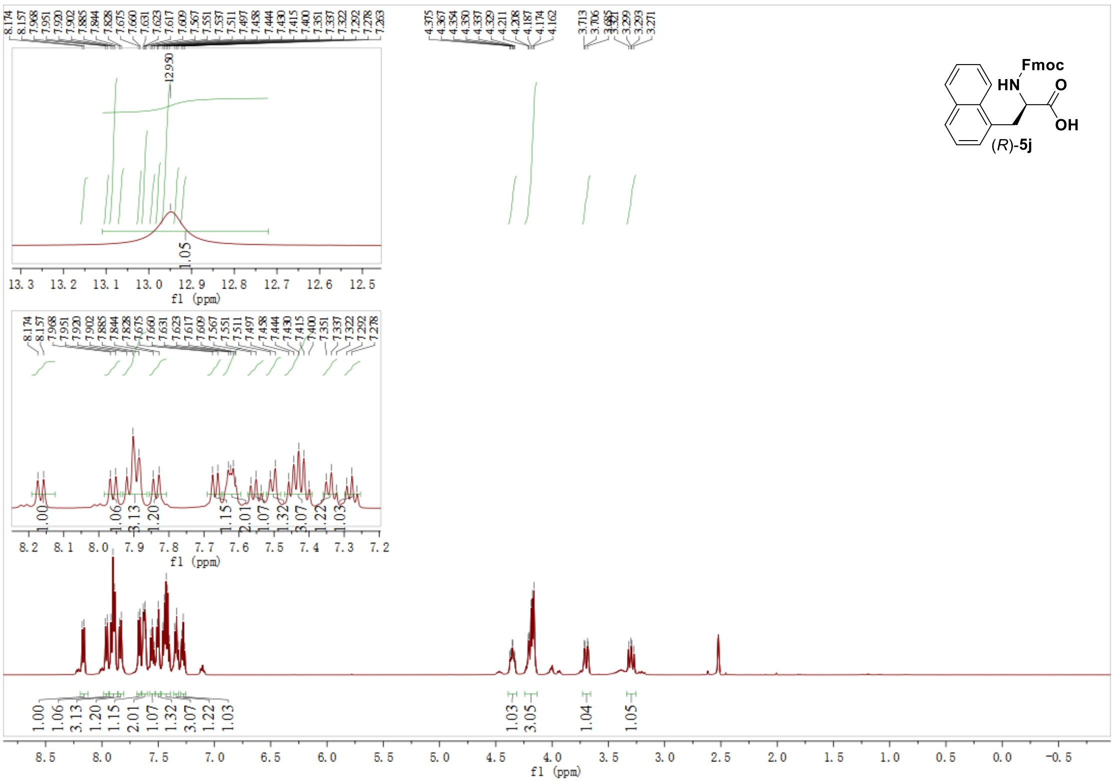

500 MHz, DMSO-d_6, ^{1}H NMR

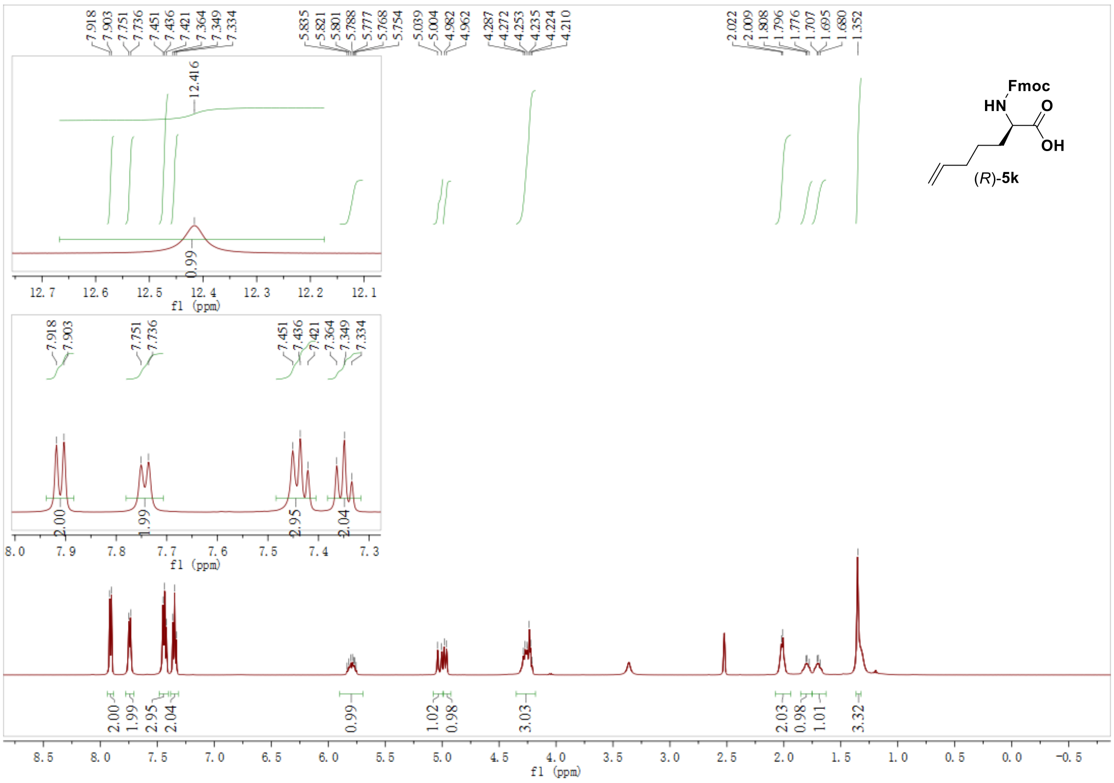

500 MHz, DMSO-d_6, ^{1}H NMR

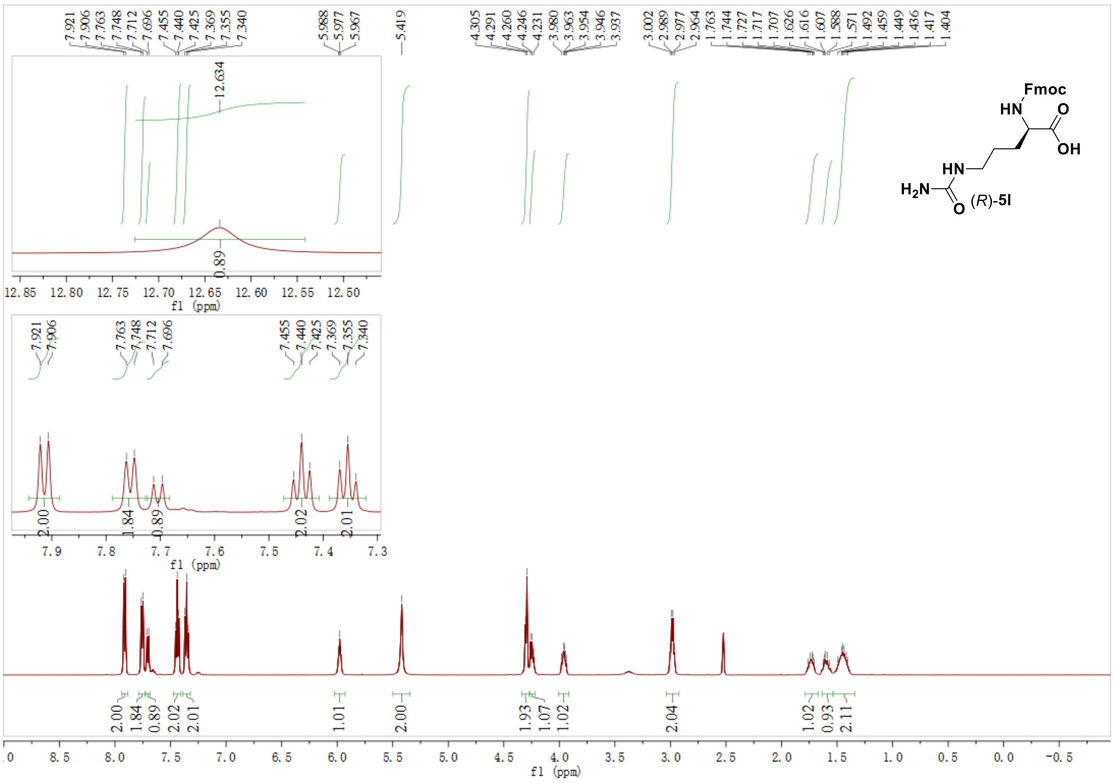

500 MHz, DMSO-d_6, ^{1}H NMR

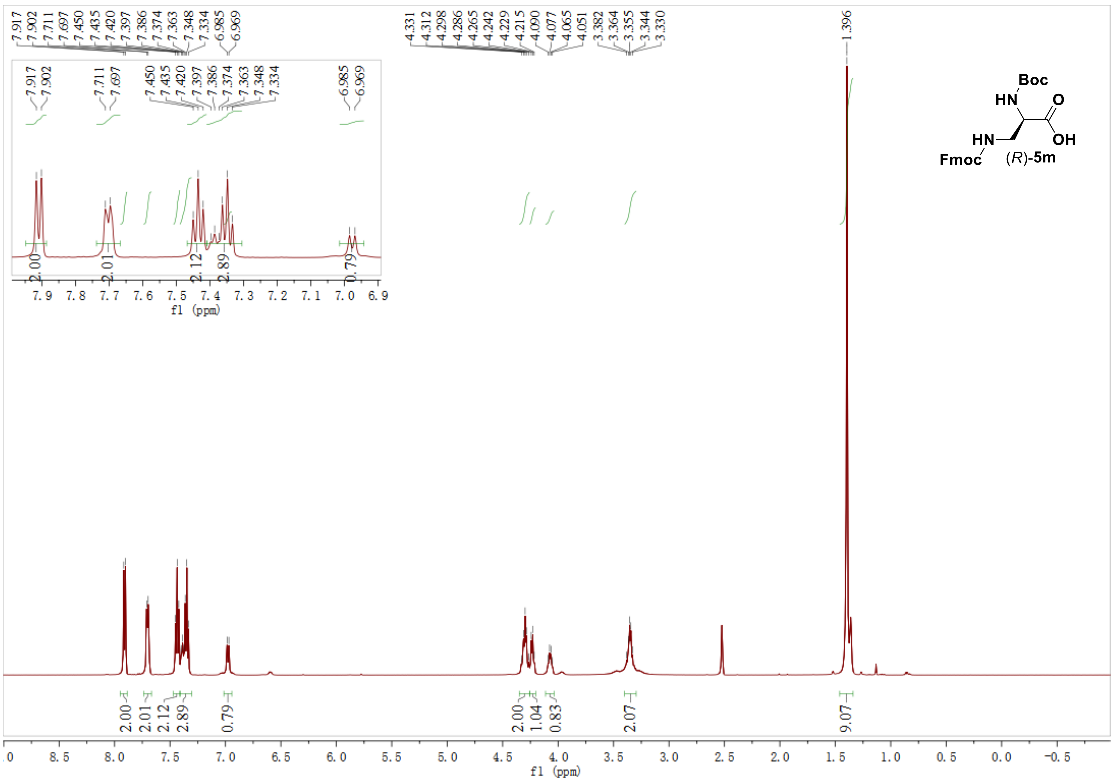

500 MHz, DMSO-d_6, ^{1}H NMR

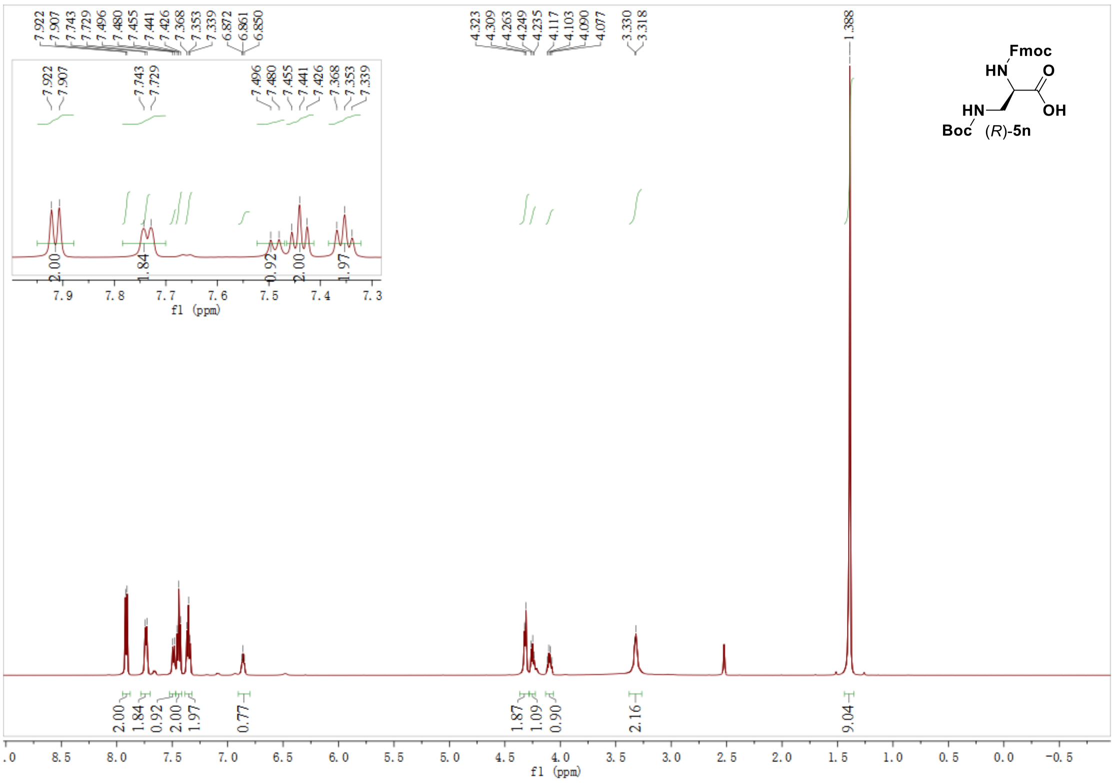

500 MHz, DMSO-d_6, ^{1}H NMR

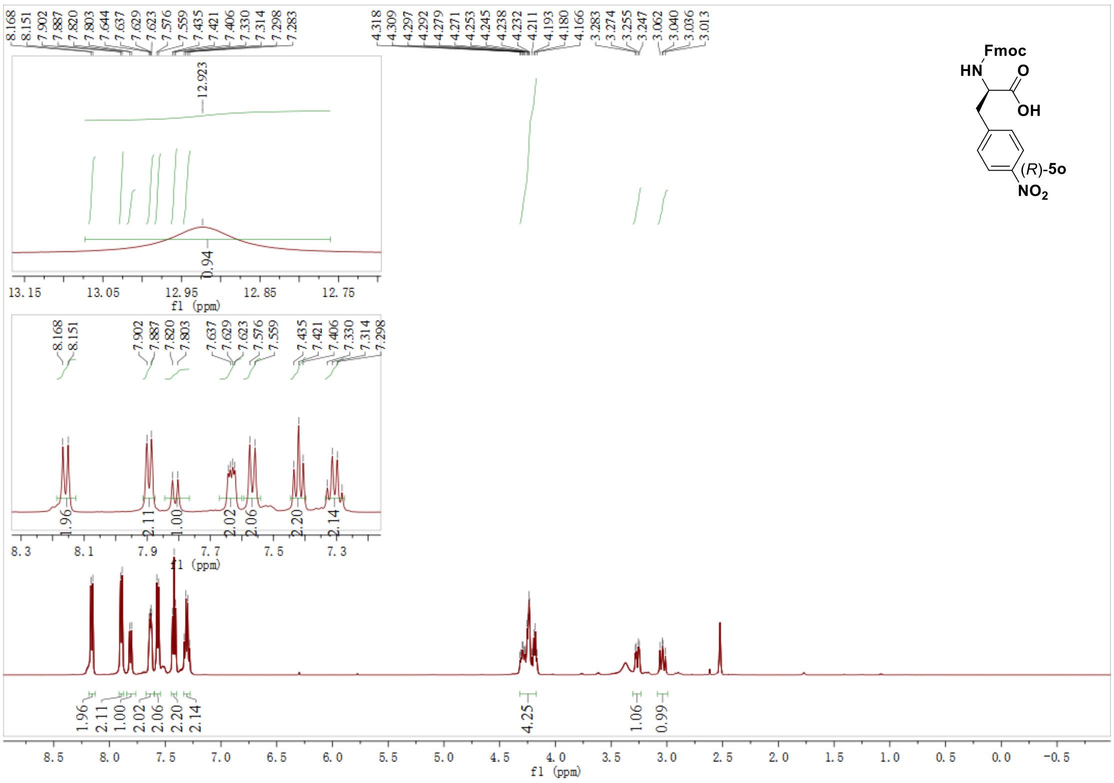

500 MHz, DMSO-d_6, ^{1}H NMR

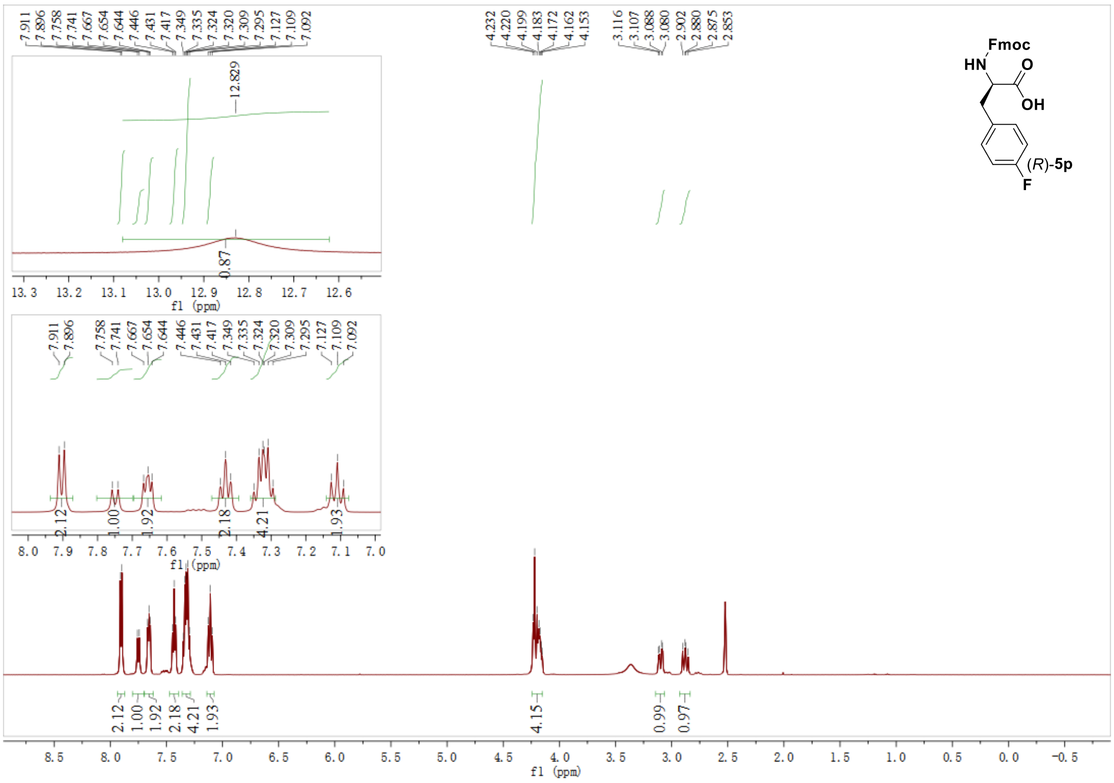

500 MHz, DMSO-d_6, ^{1}H NMR

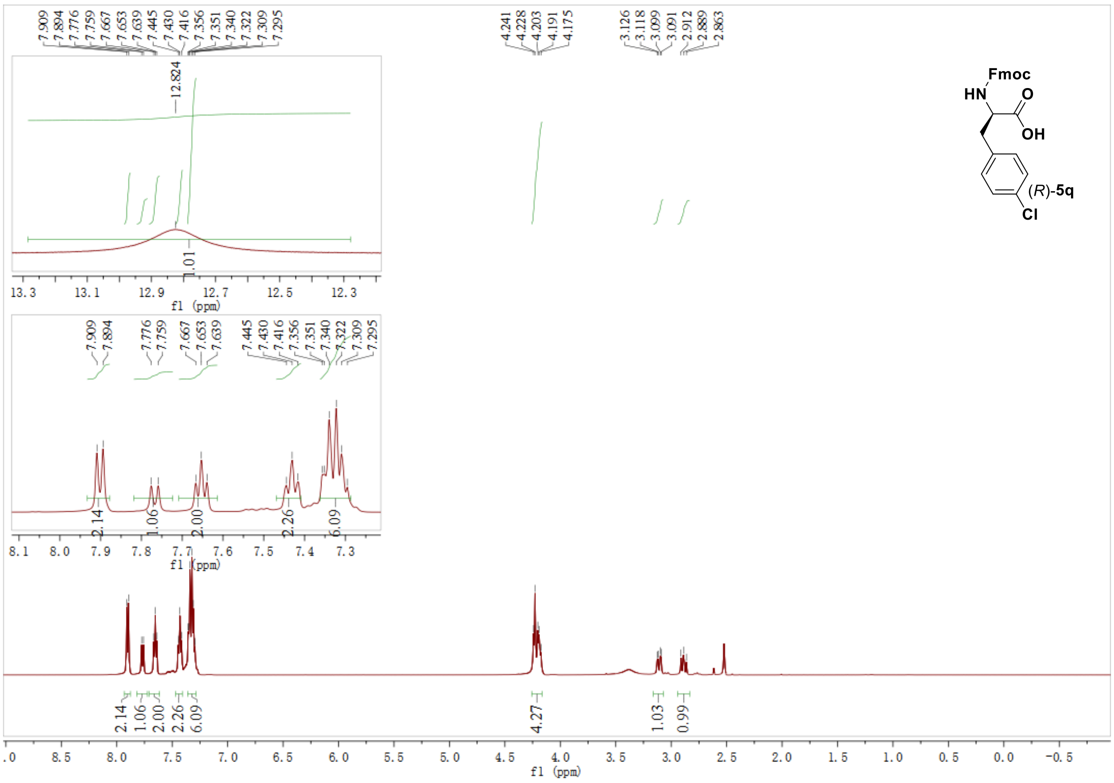

500 MHz, DMSO-d_6, ^{1}H NMR

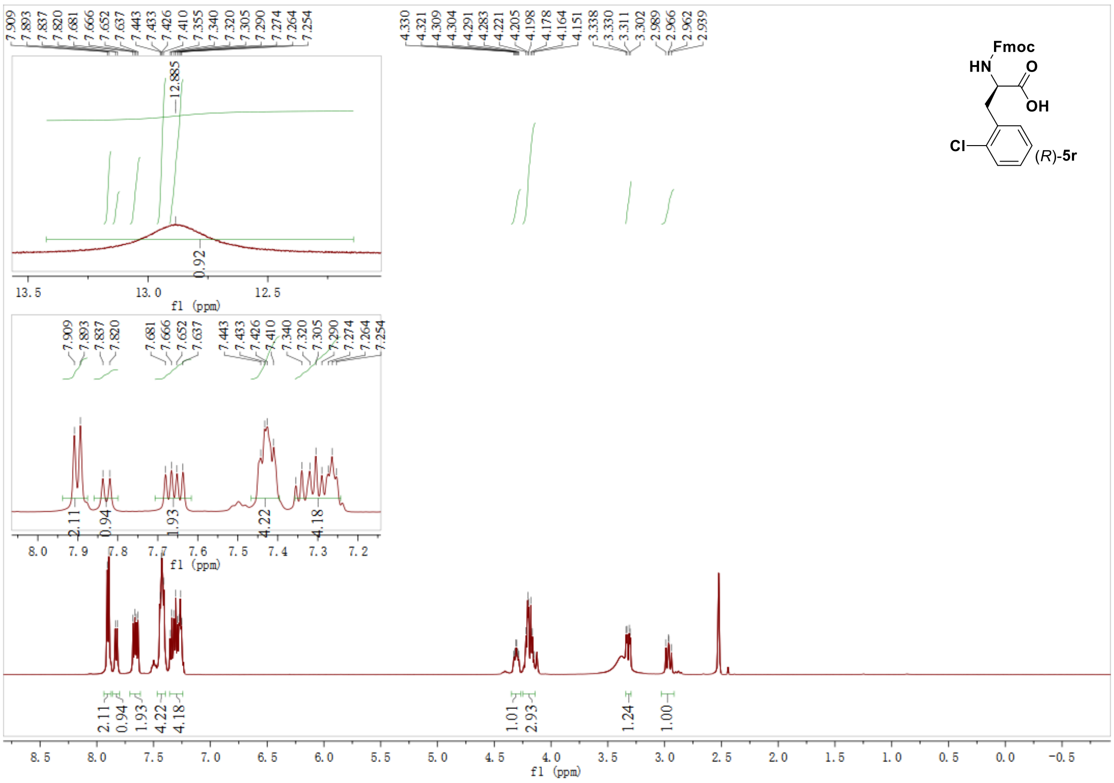

500 MHz, DMSO-d_6, ^{1}H NMR

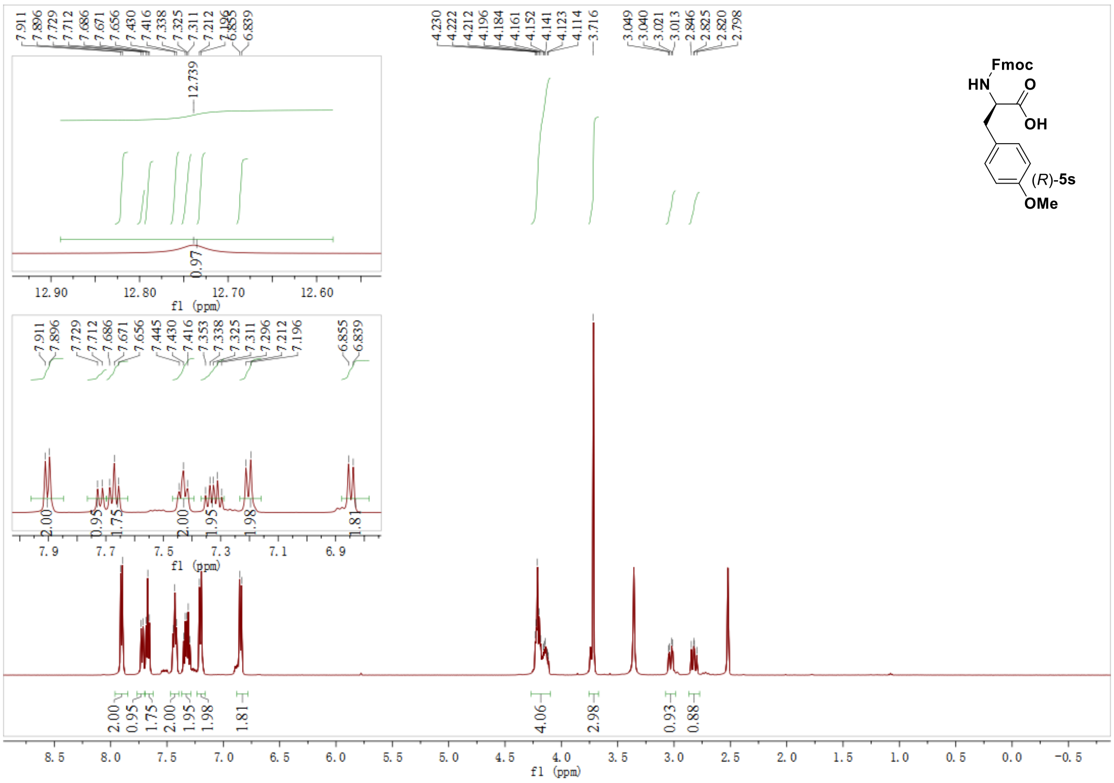

500 MHz, DMSO-d_6, ^{1}H NMR

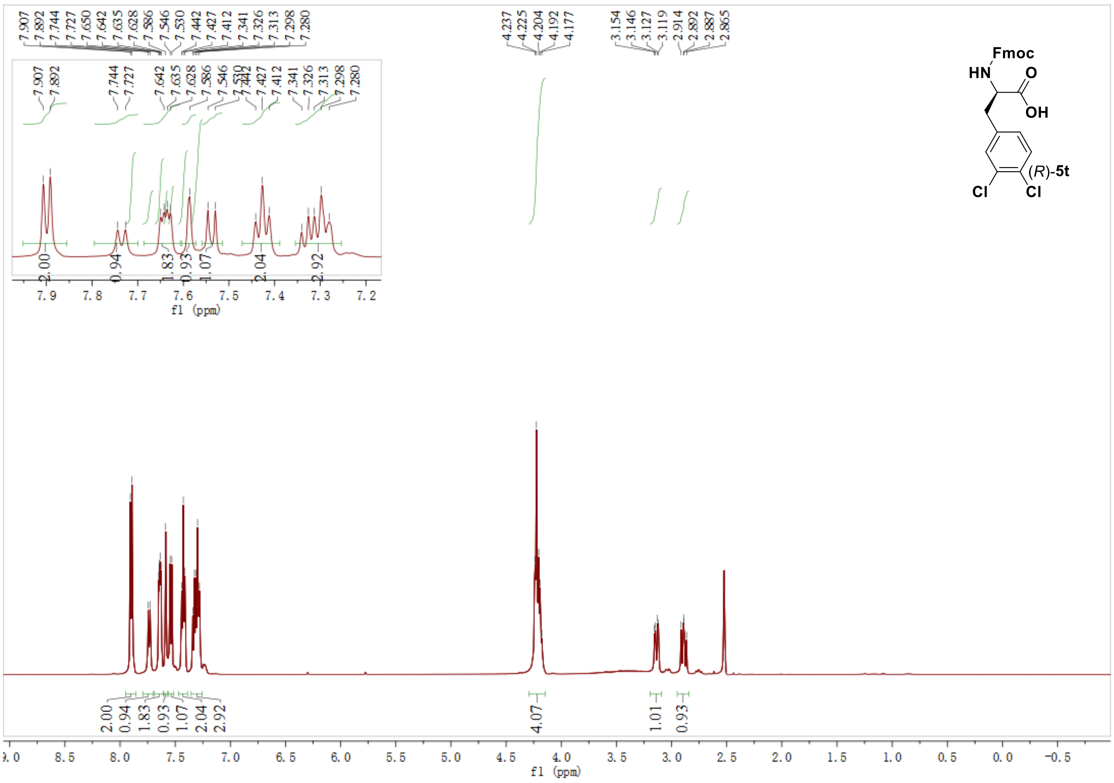

500 MHz, DMSO-d_6, ^{1}H NMR

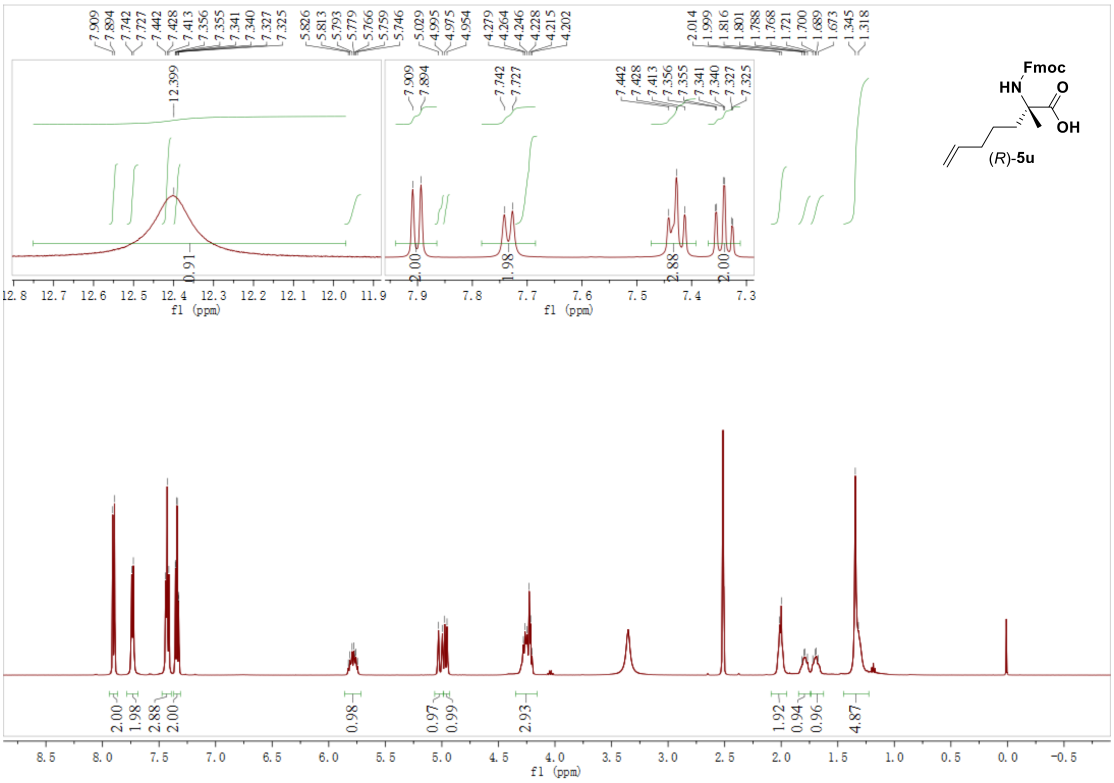

500 MHz, DMSO-d_6, ^{1}H NMR

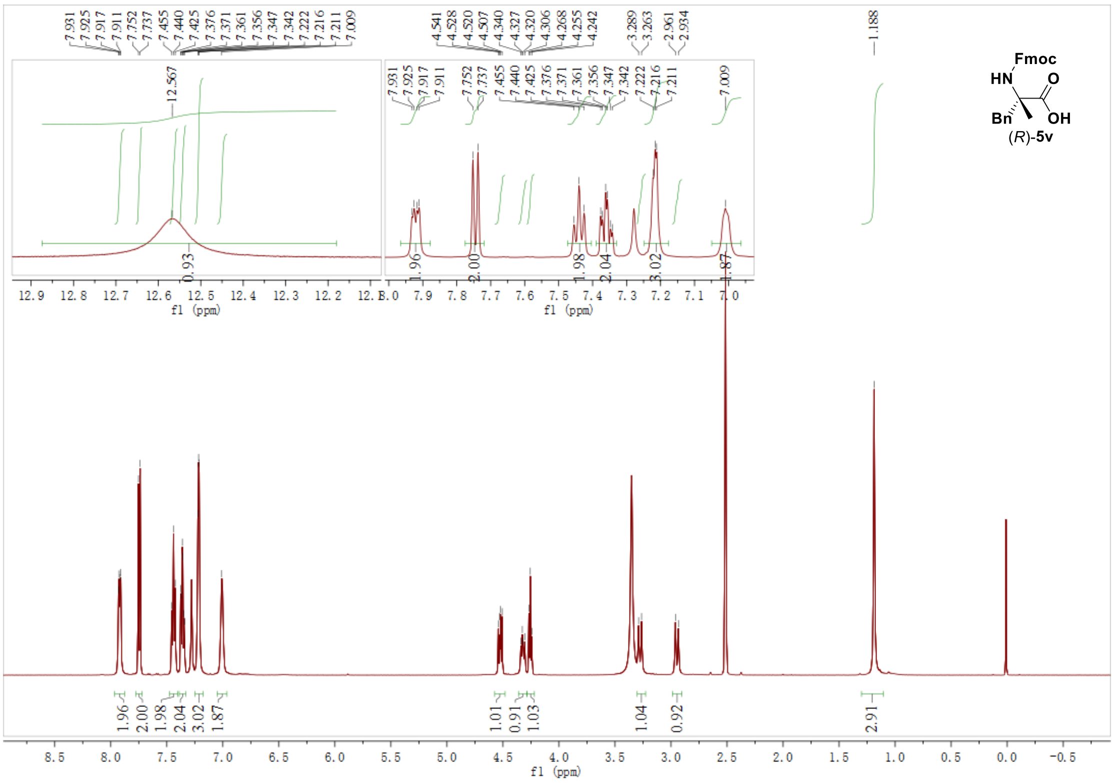

500 MHz, $CDCl_3$, ^{1}H NMR

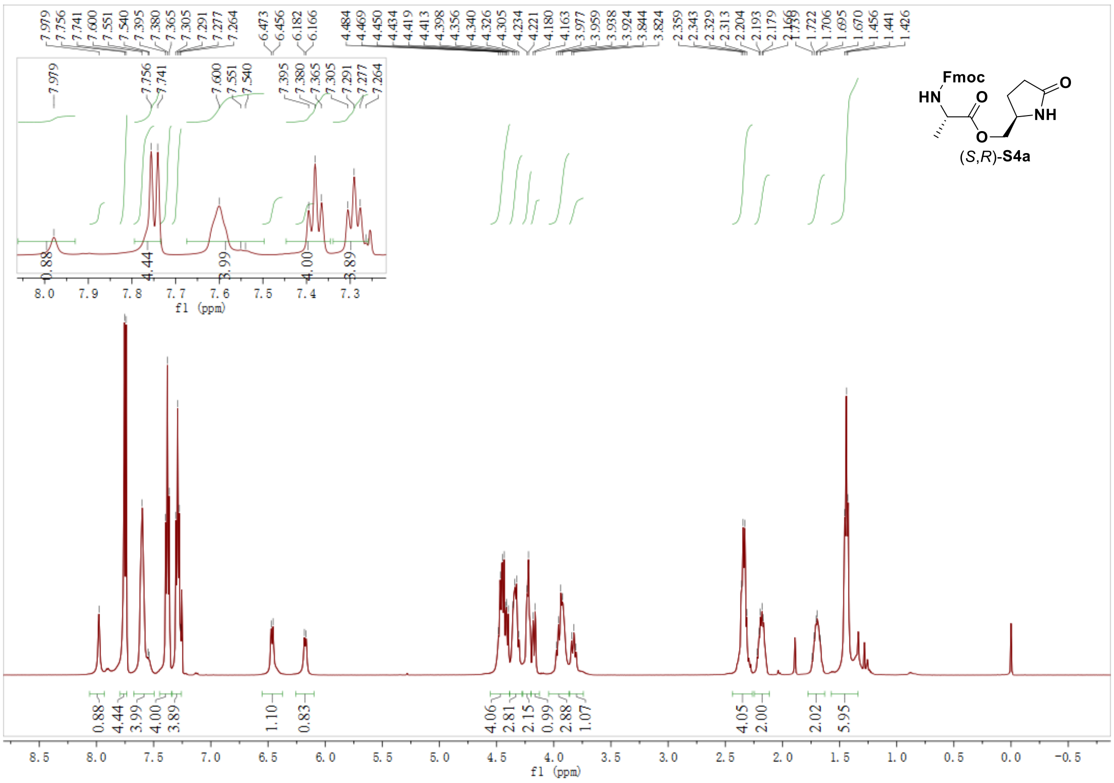

125 MHz, $CDCl_3$, ^{13}C NMR

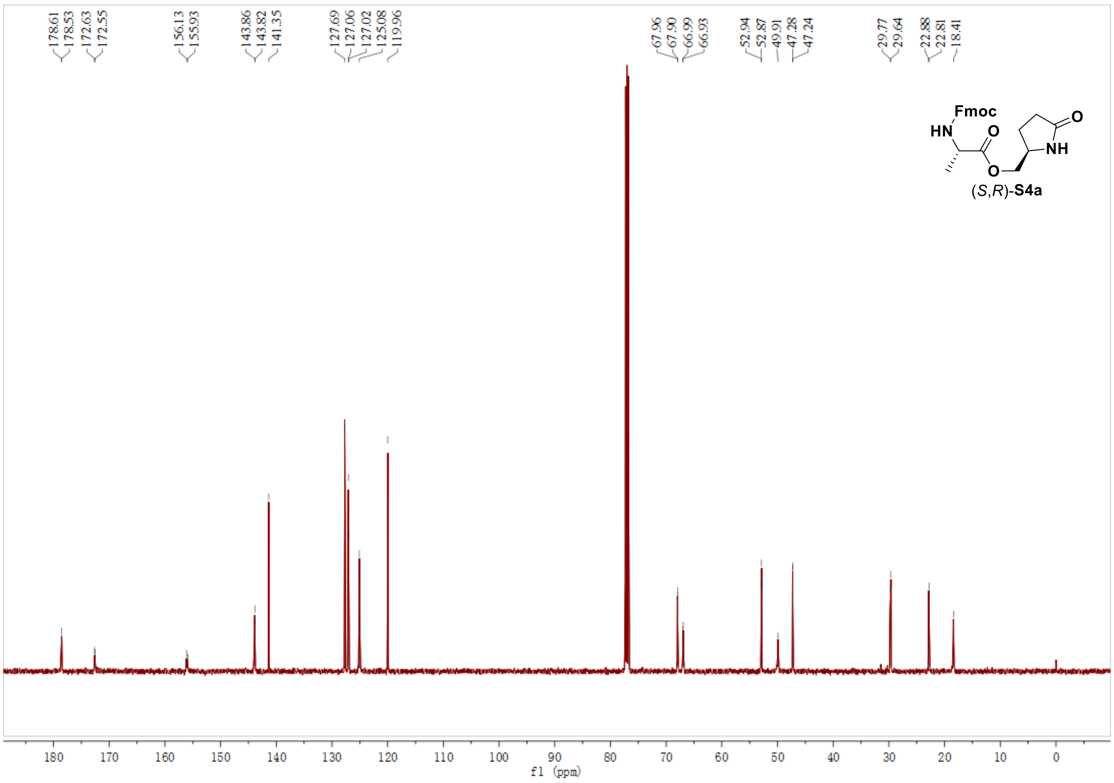

500 MHz, $CDCl_3$, ^1H NMR

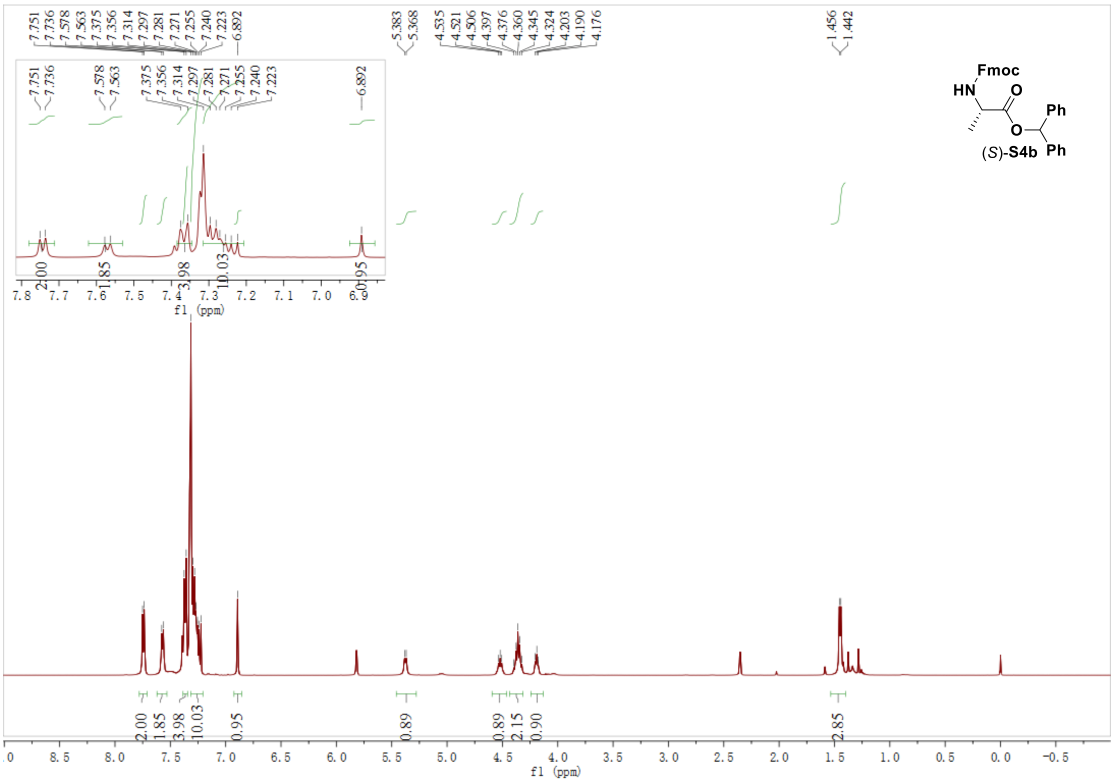

125 MHz, $CDCl_3$, ^{13}C NMR

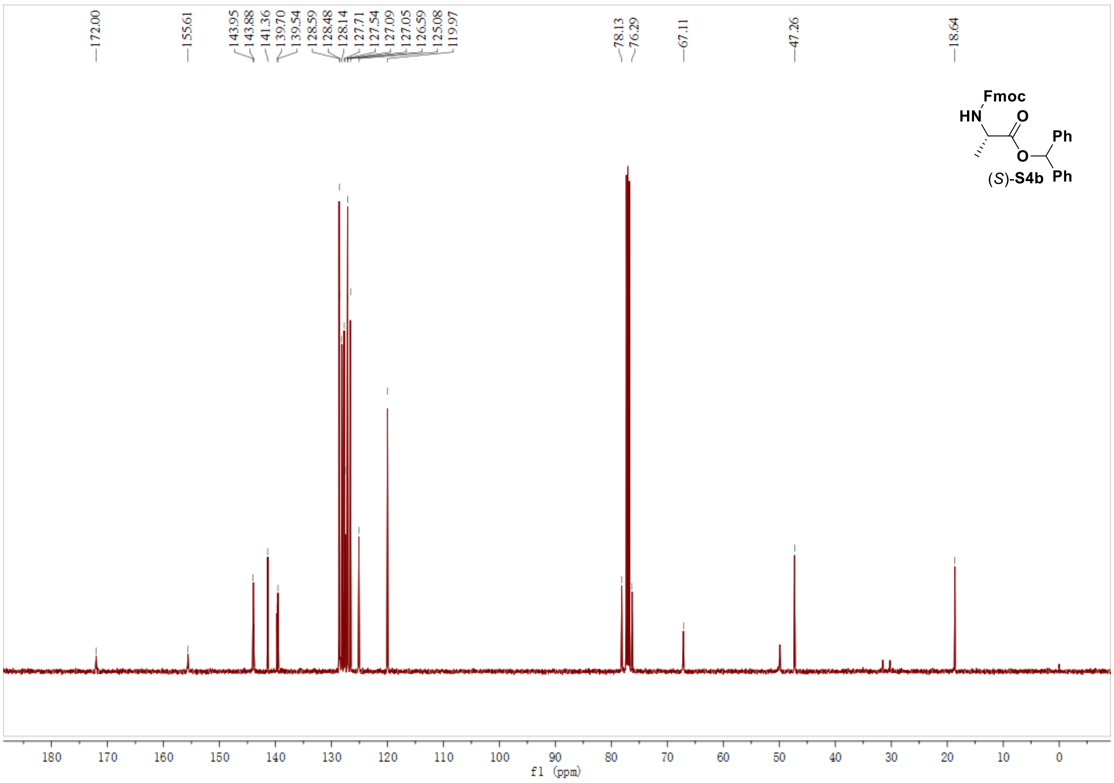

500 MHz, CDCl$_3$, ^{1}H NMR

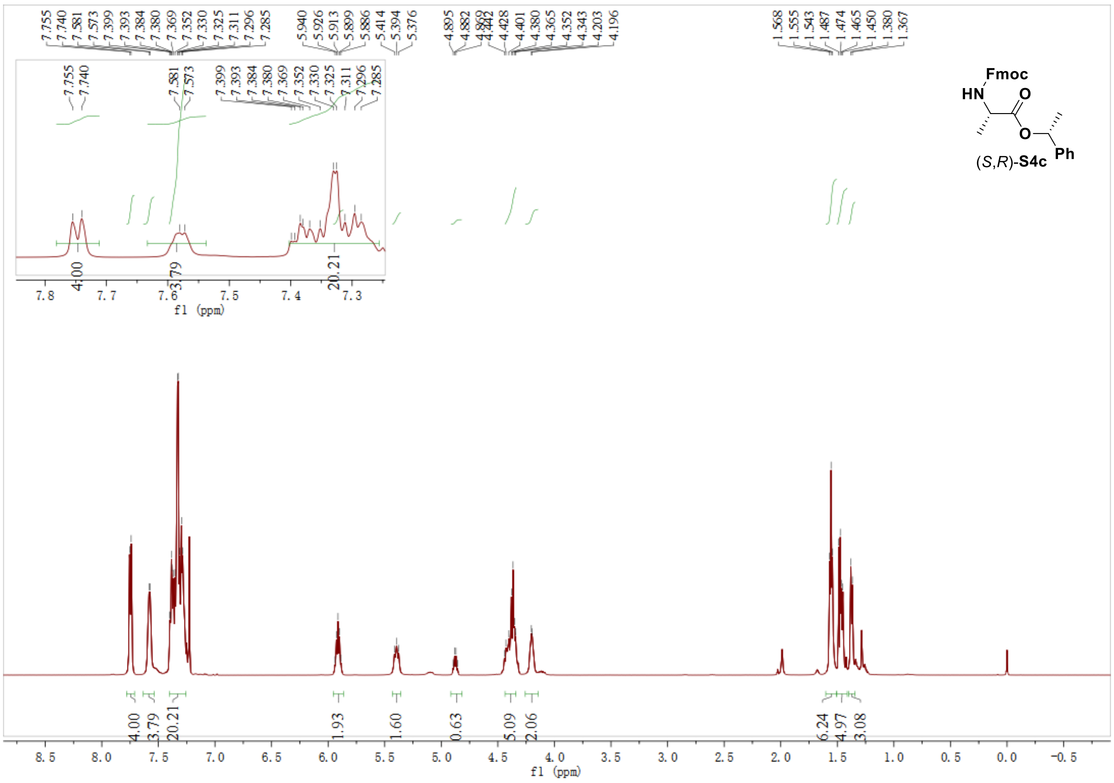

125 MHz, CDCl$_3$, ^{13}C NMR

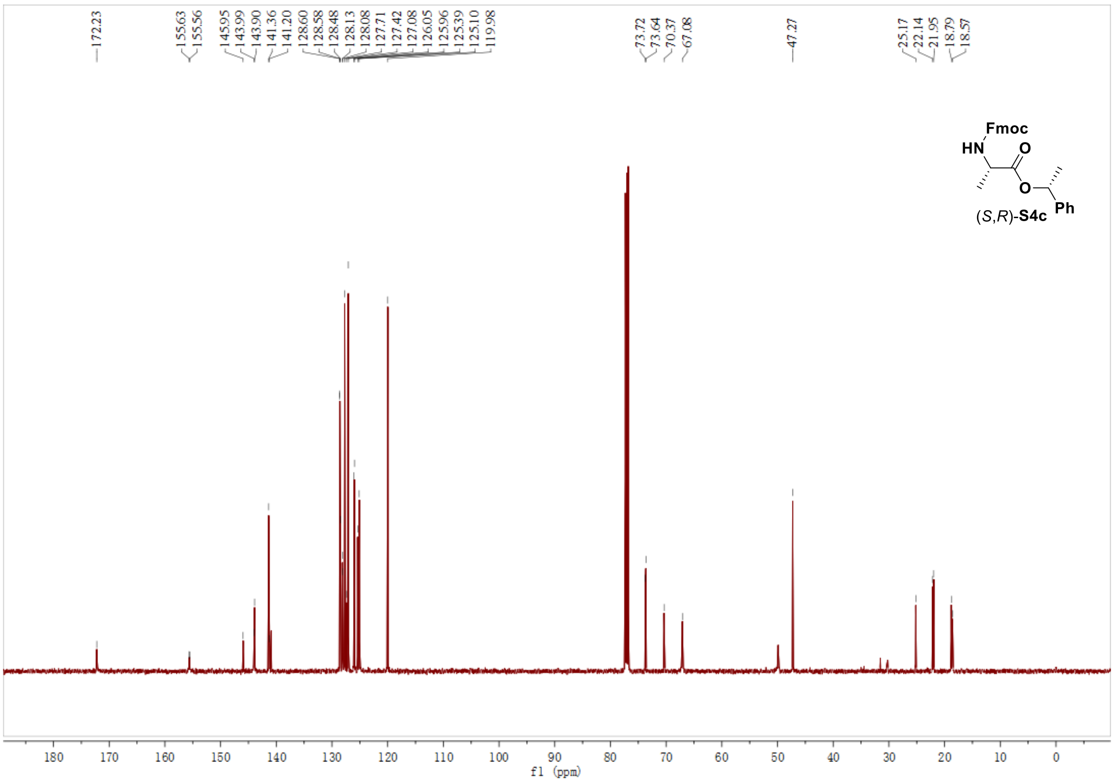

500 MHz, $CDCl_3$, ^{1}H NMR

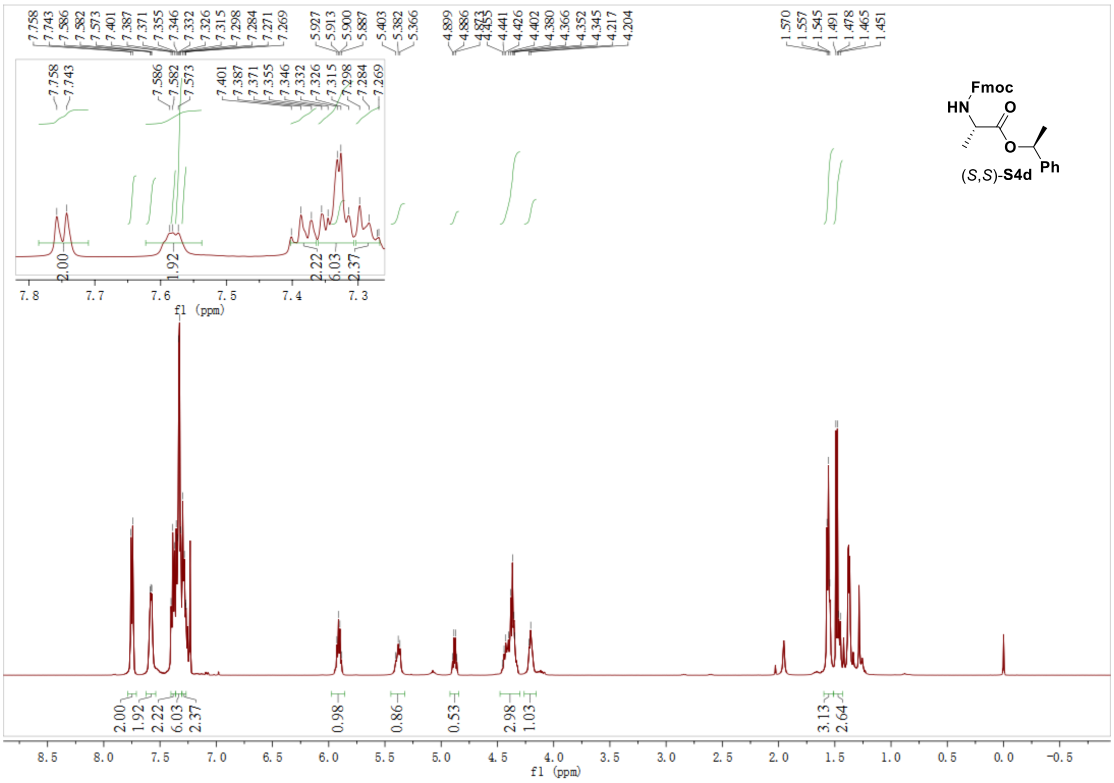

125 MHz, $CDCl_3$, ^{13}C NMR

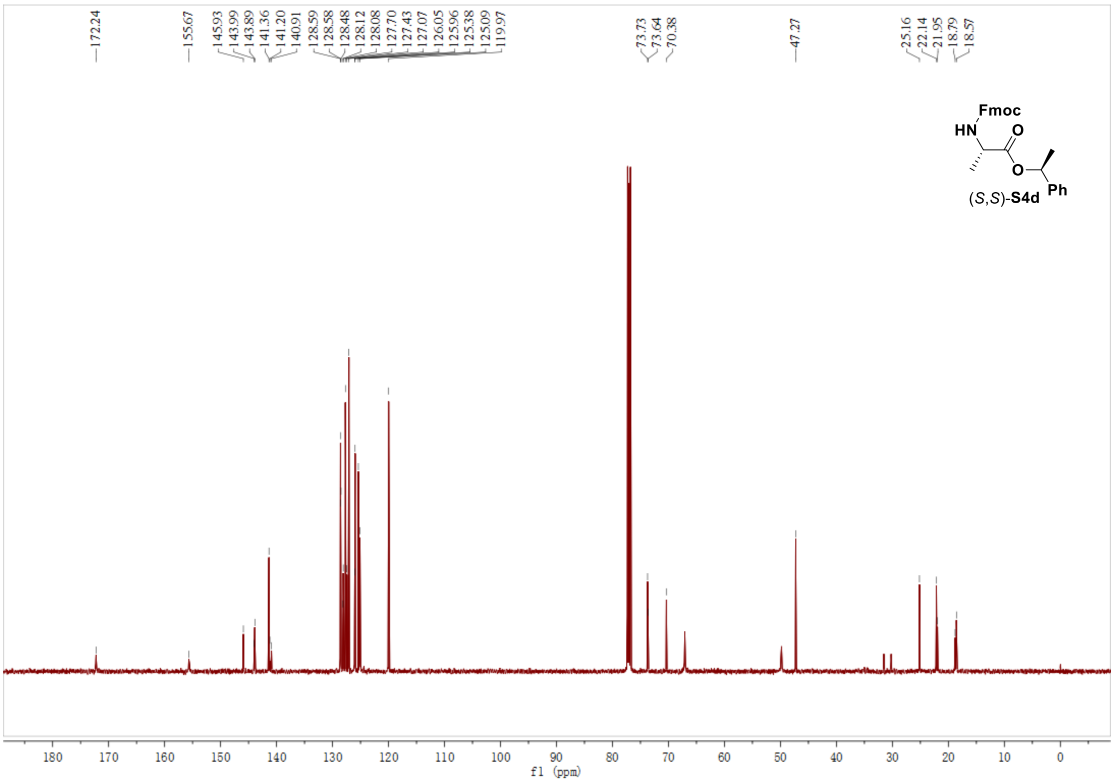

500 MHz, $CDCl_3$, ^1H NMR

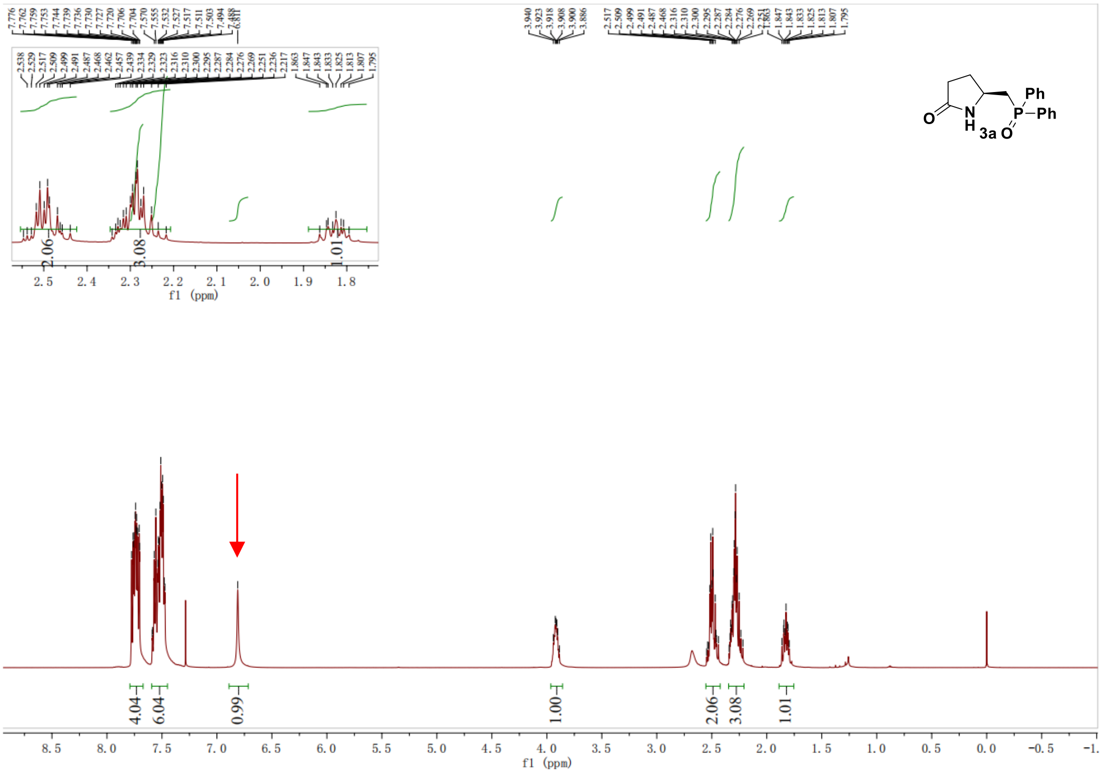

500 MHz, $CDCl_3$, ^1H NMR

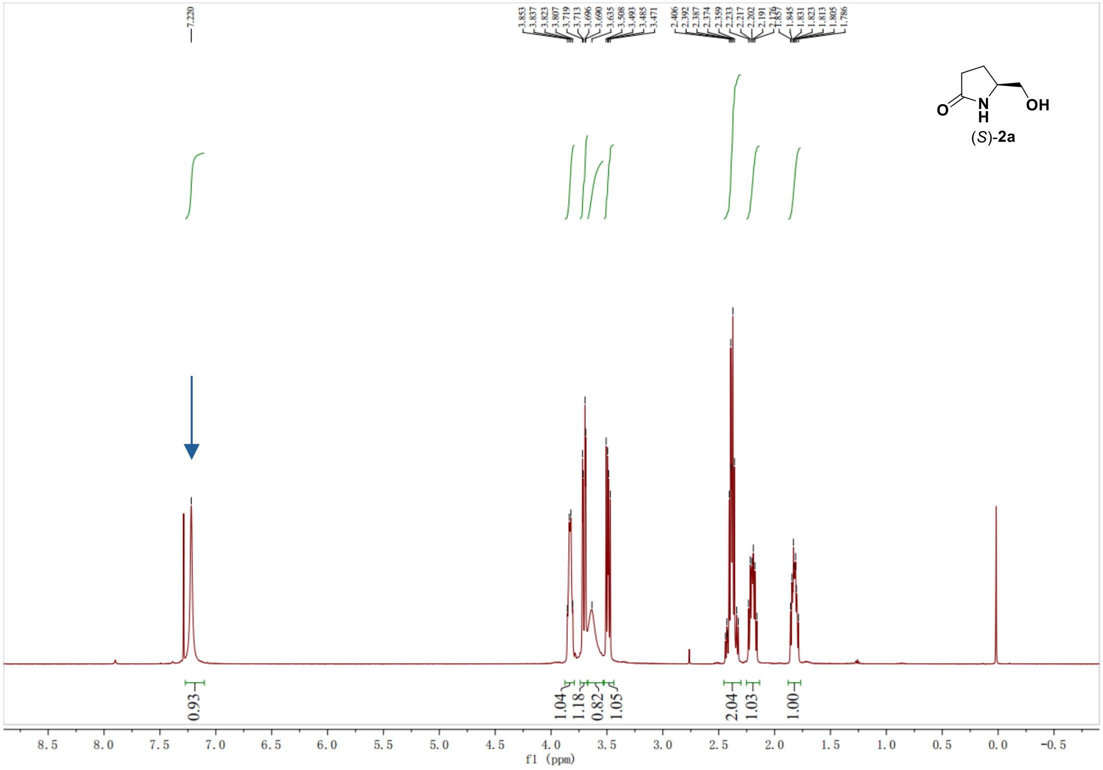

500 MHz, $CDCl_3$, ^1H NMR

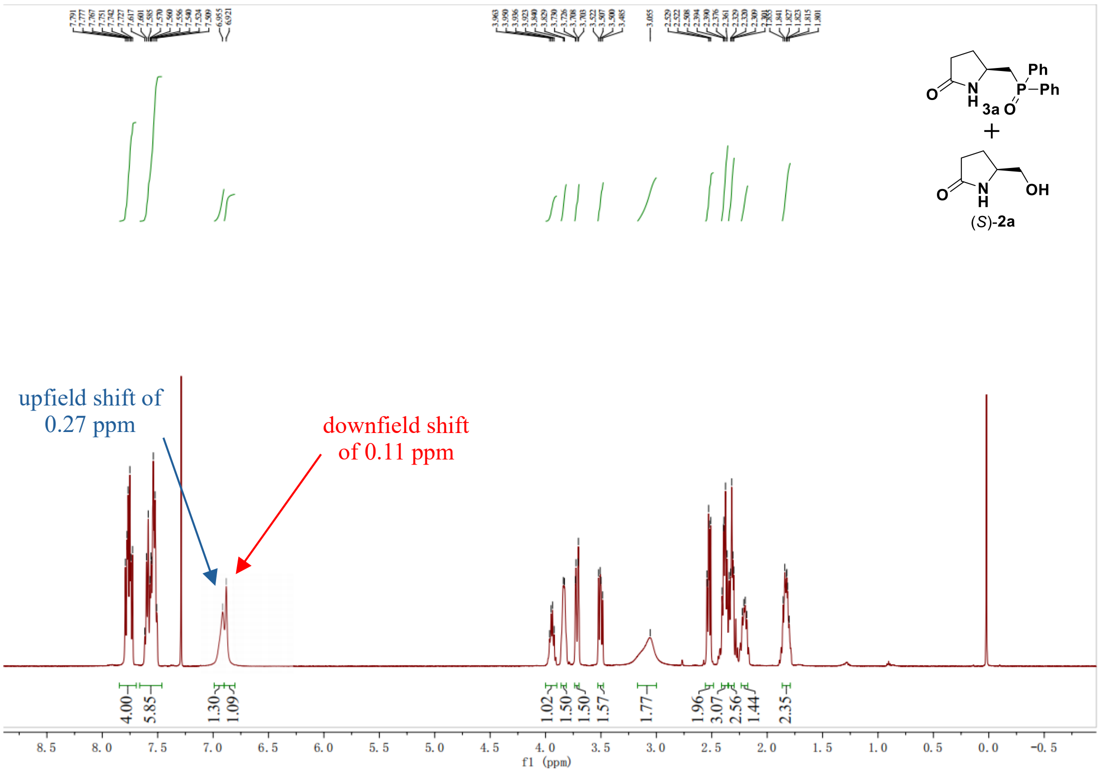

4. Crude ^{1}H NMR Spectra

500 MHz, DMSO-d_6, ^{1}H NMR

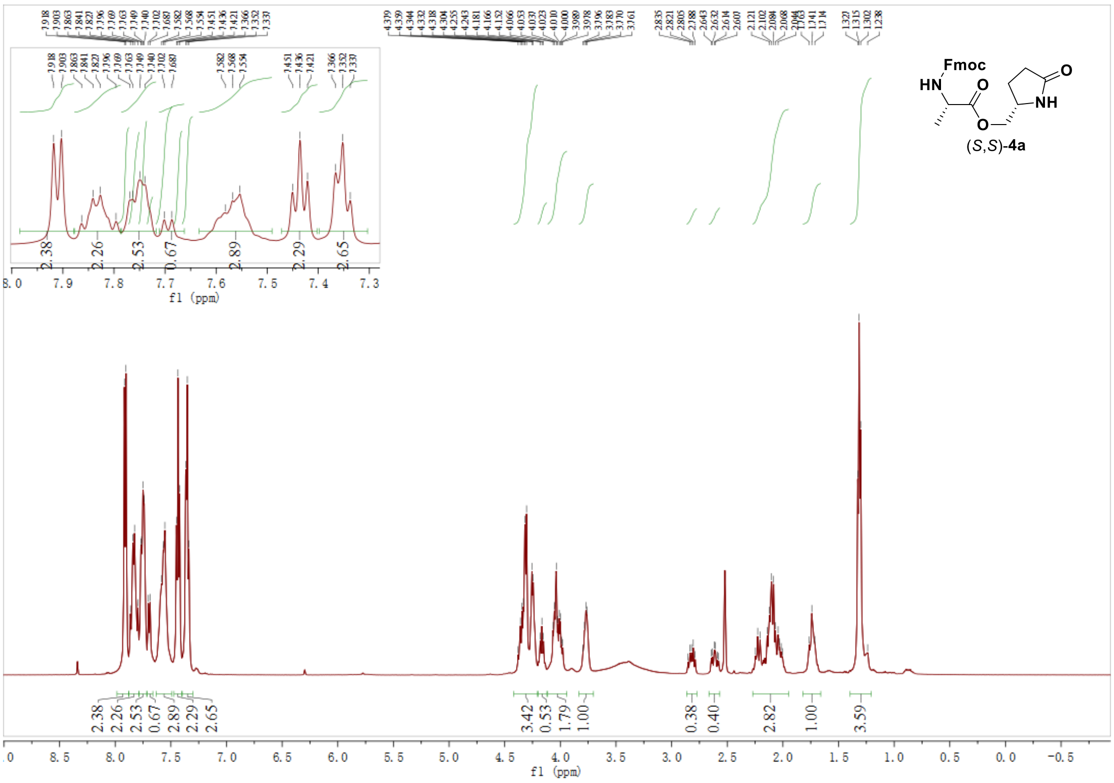

500 MHz, DMSO-d_6, ^{1}H NMR

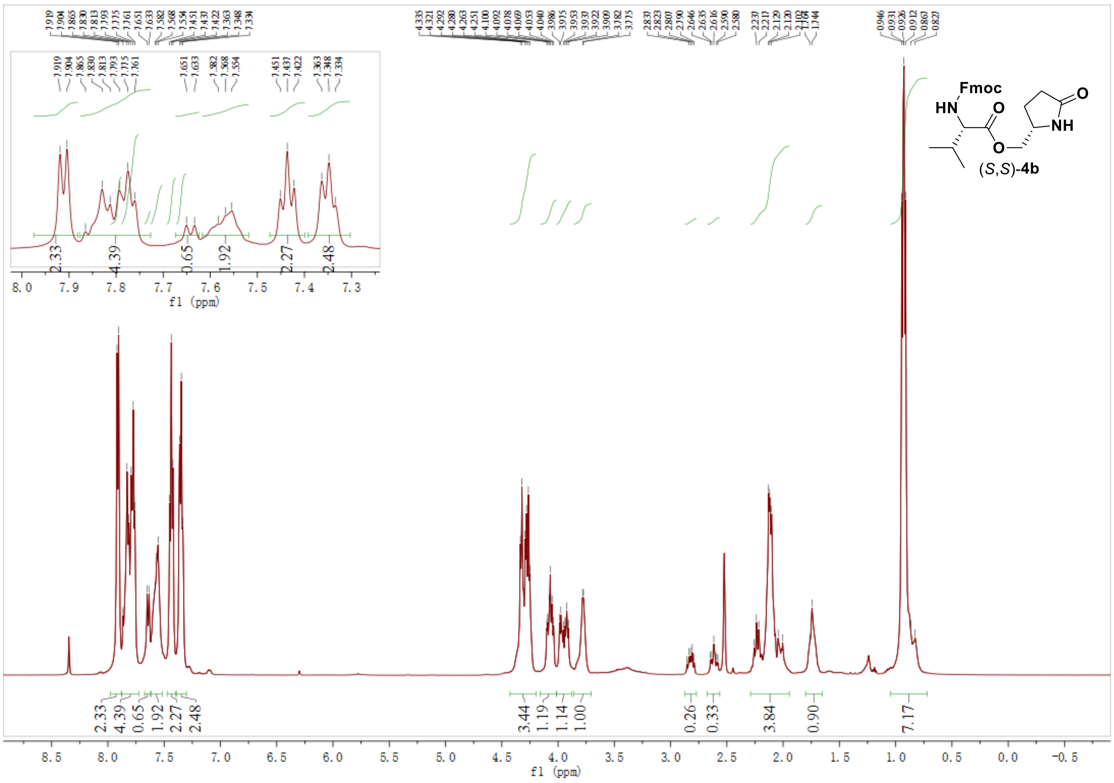

500 MHz, DMSO-d_6, ^{1}H NMR

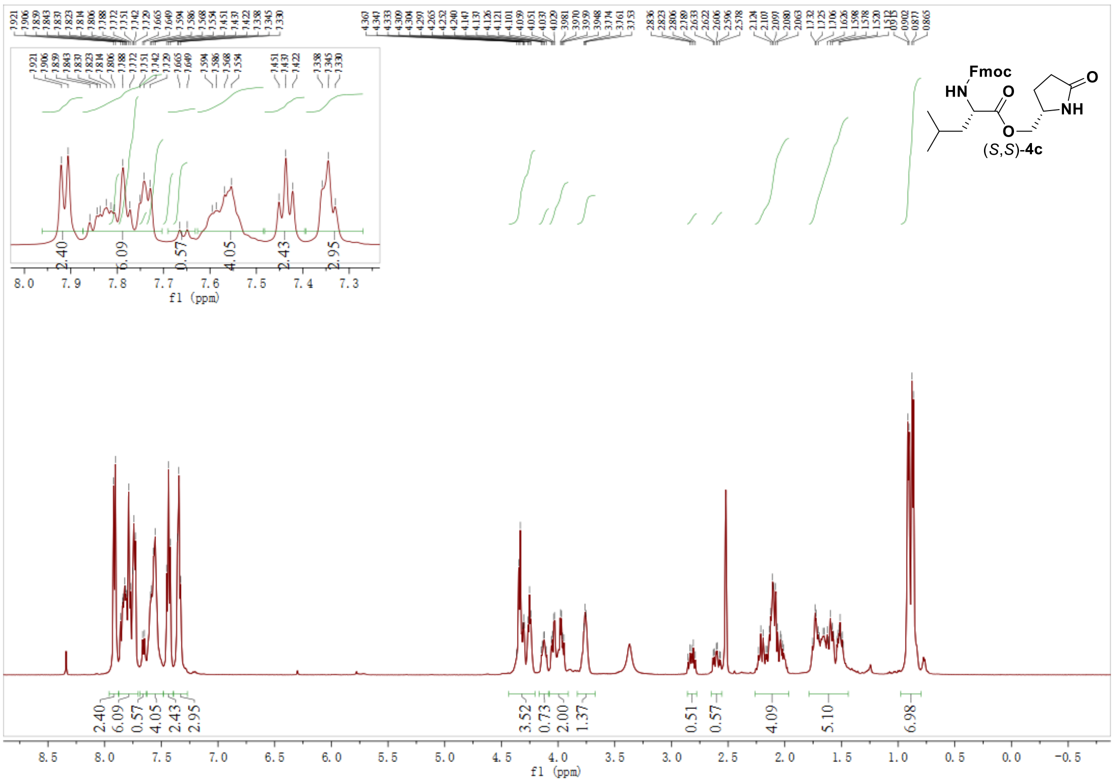

500 MHz, DMSO-d_6, ^{1}H NMR

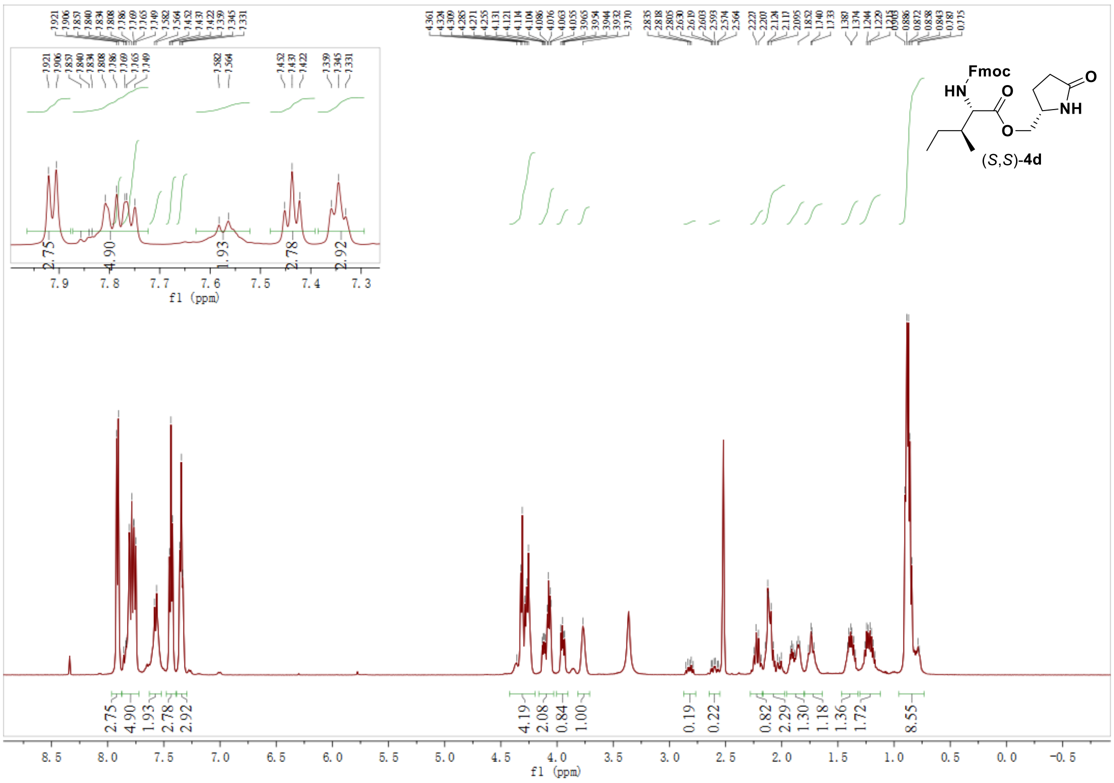

500 MHz, DMSO-d_6, ^{1}H NMR

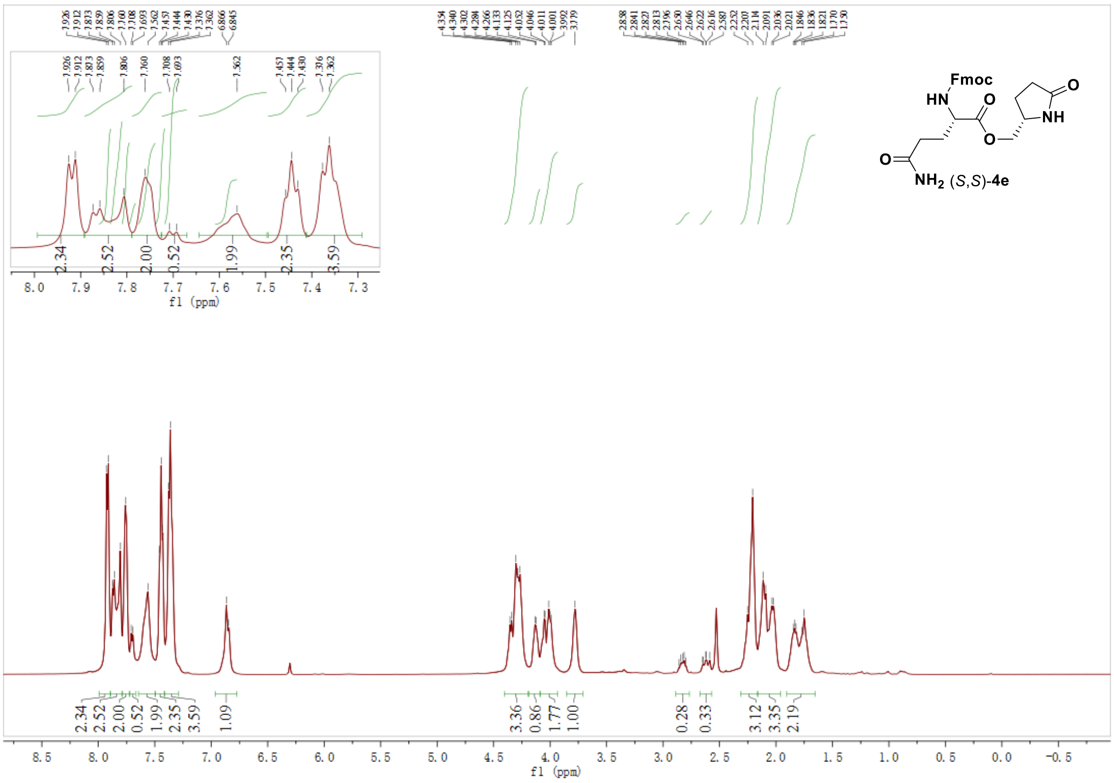

500 MHz, DMSO-d_6, ^{1}H NMR

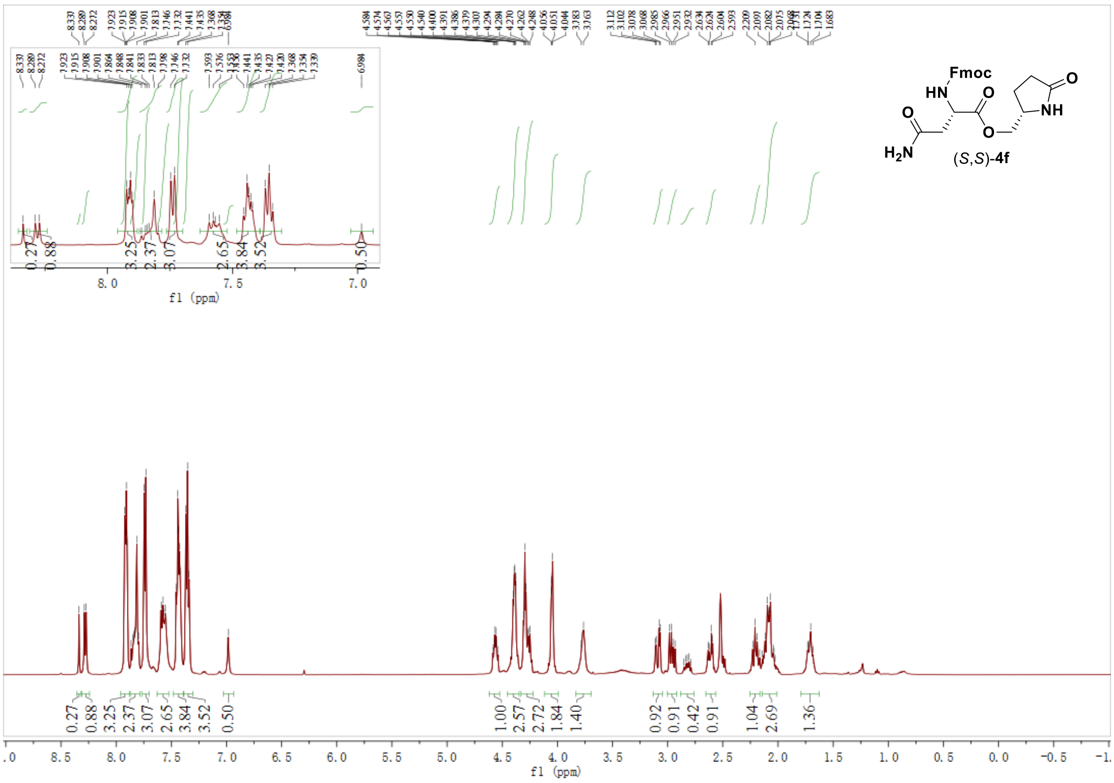

500 MHz, DMSO-d_6, ^{1}H NMR

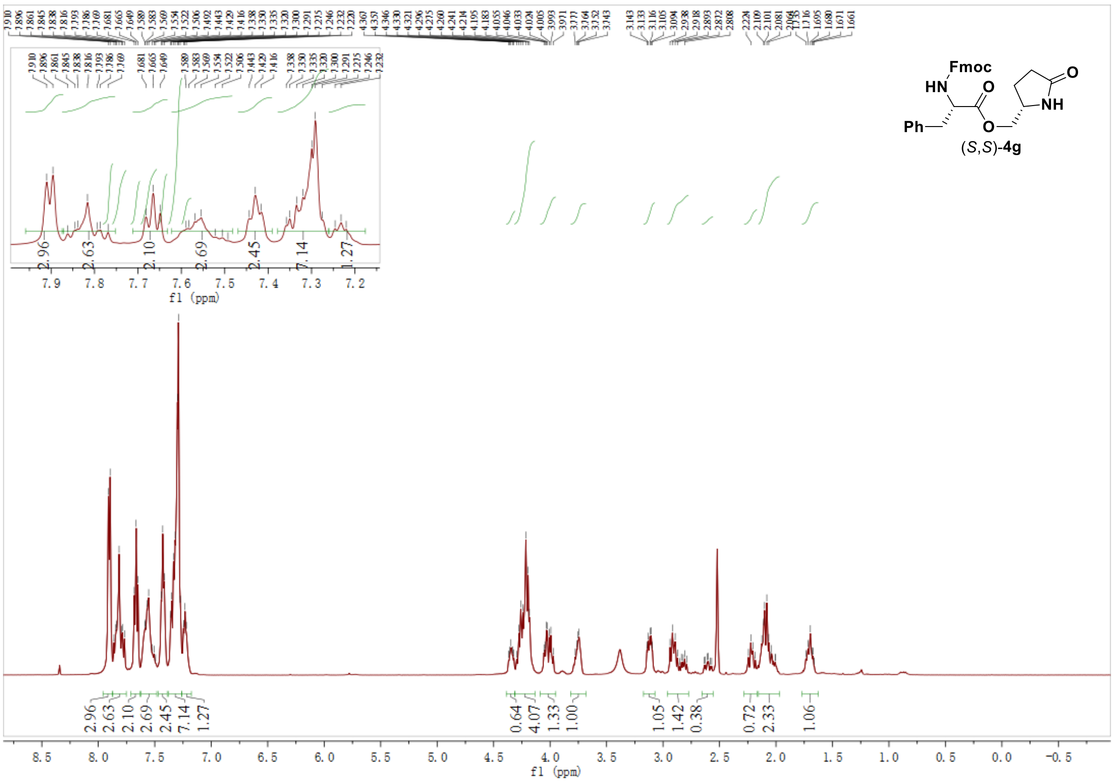

500 MHz, DMSO-d_6, ^{1}H NMR

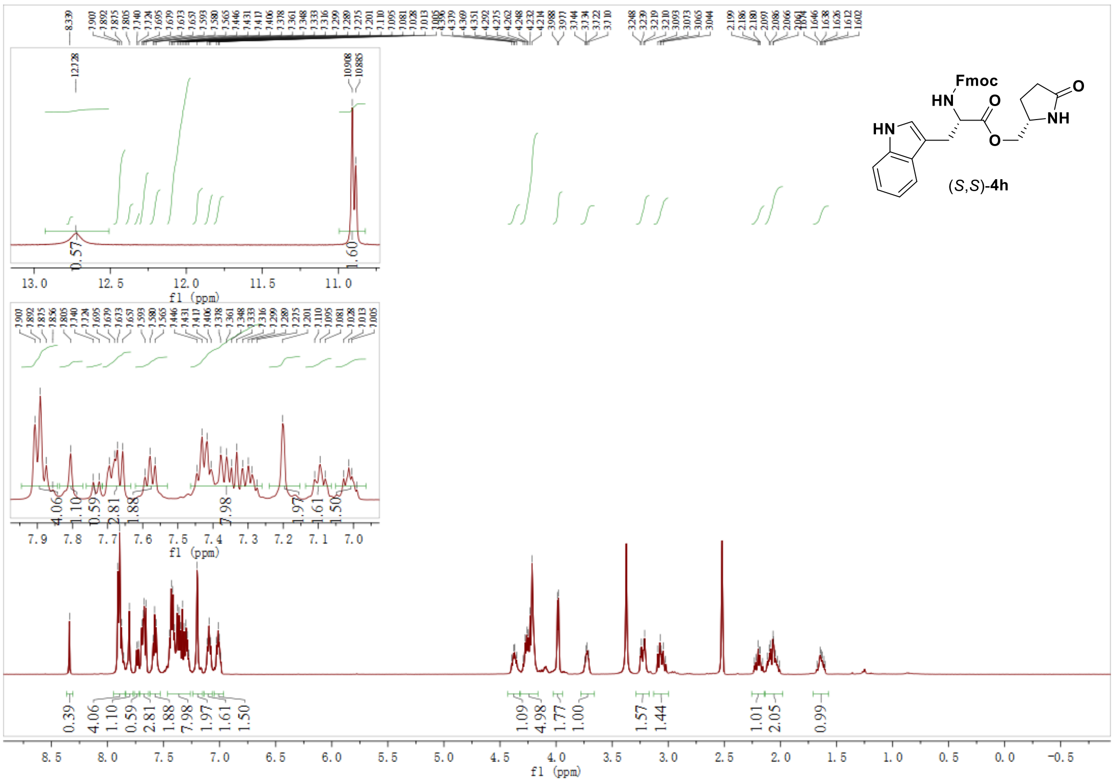

500 MHz, DMSO-d_6, ^{1}H NMR

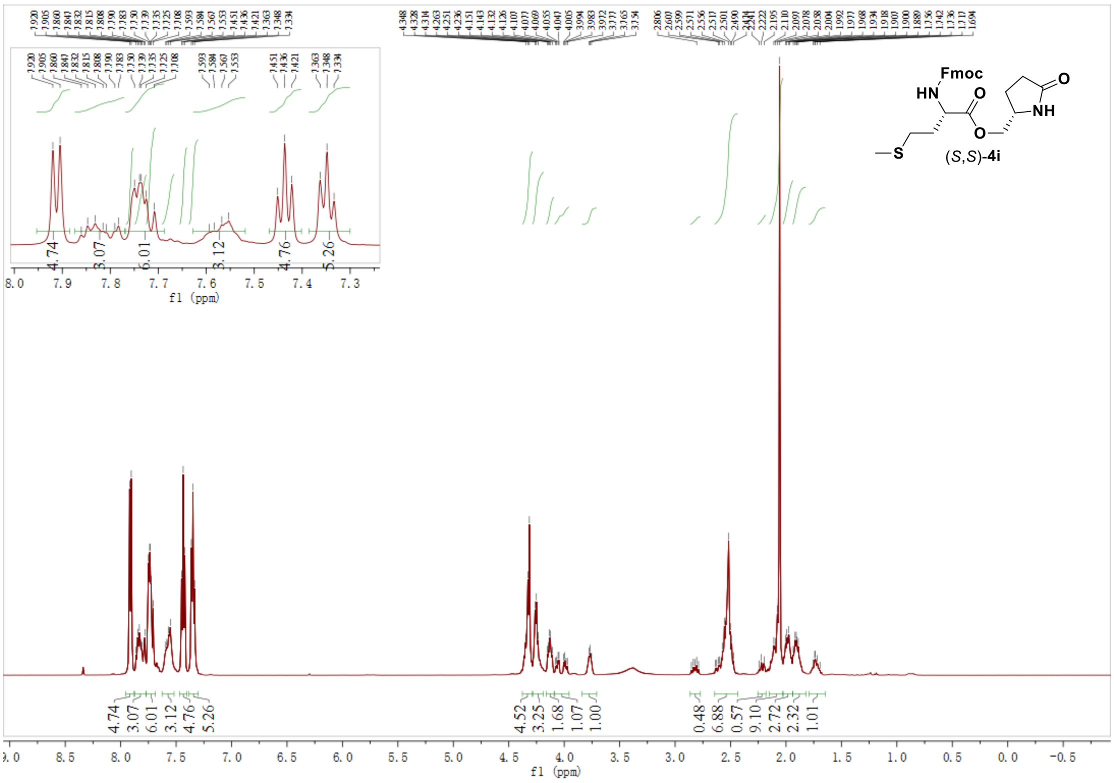

500 MHz, DMSO-d_6, ^{1}H NMR

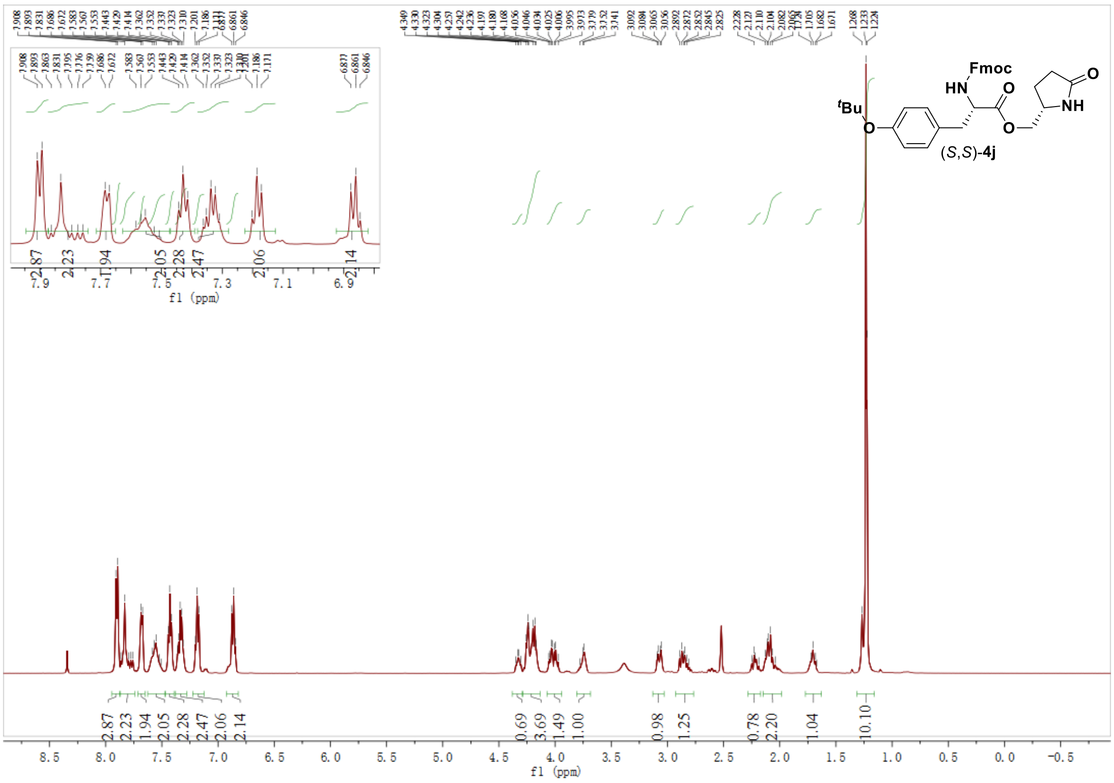

500 MHz, DMSO-d_6, ^{1}H NMR

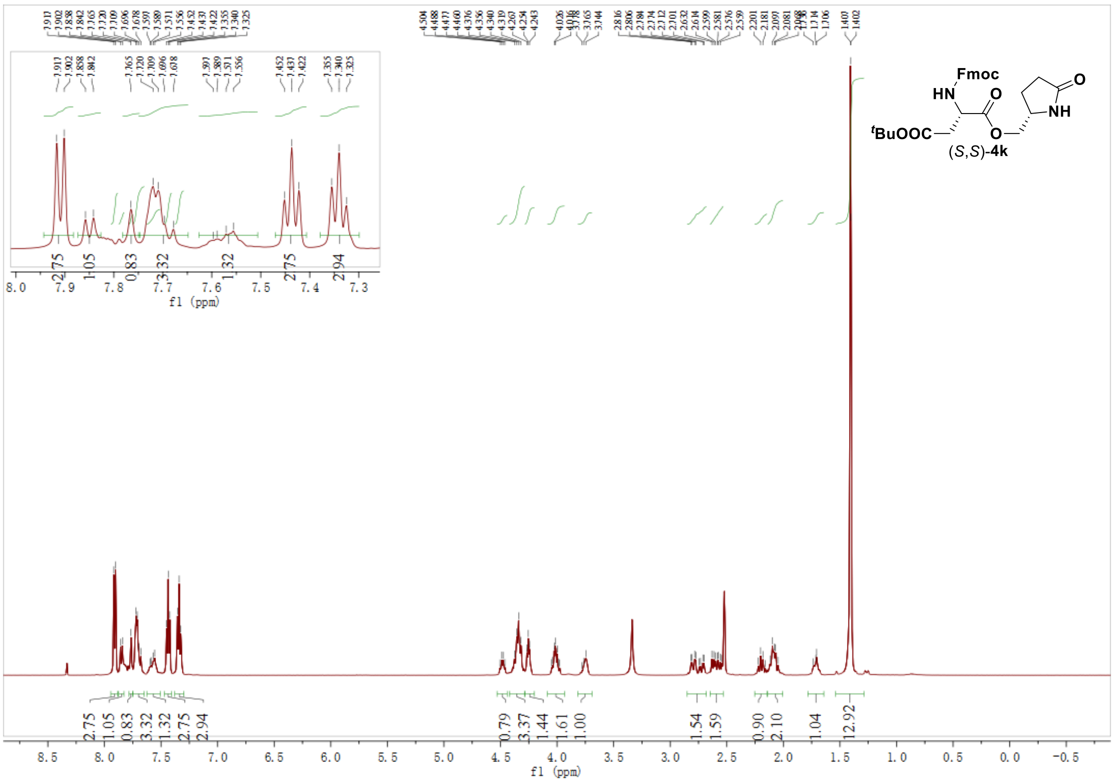

500 MHz, DMSO-d_6, ^{1}H NMR

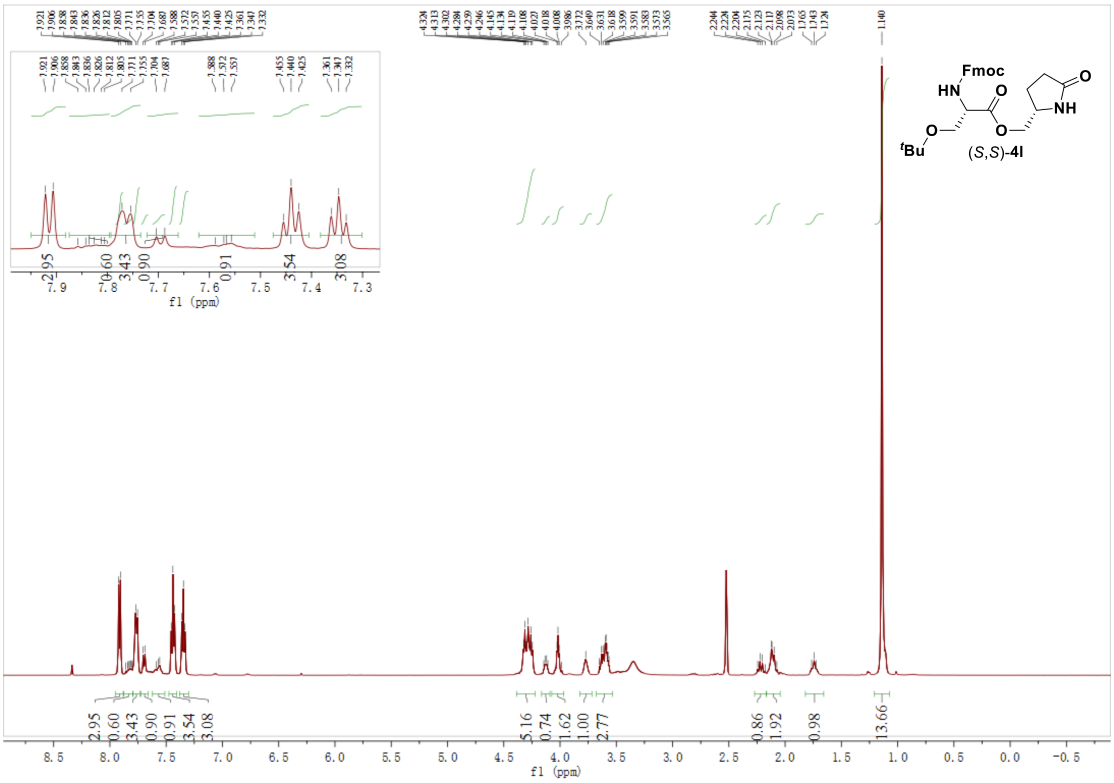

500 MHz, DMSO-d_6, ^{1}H NMR

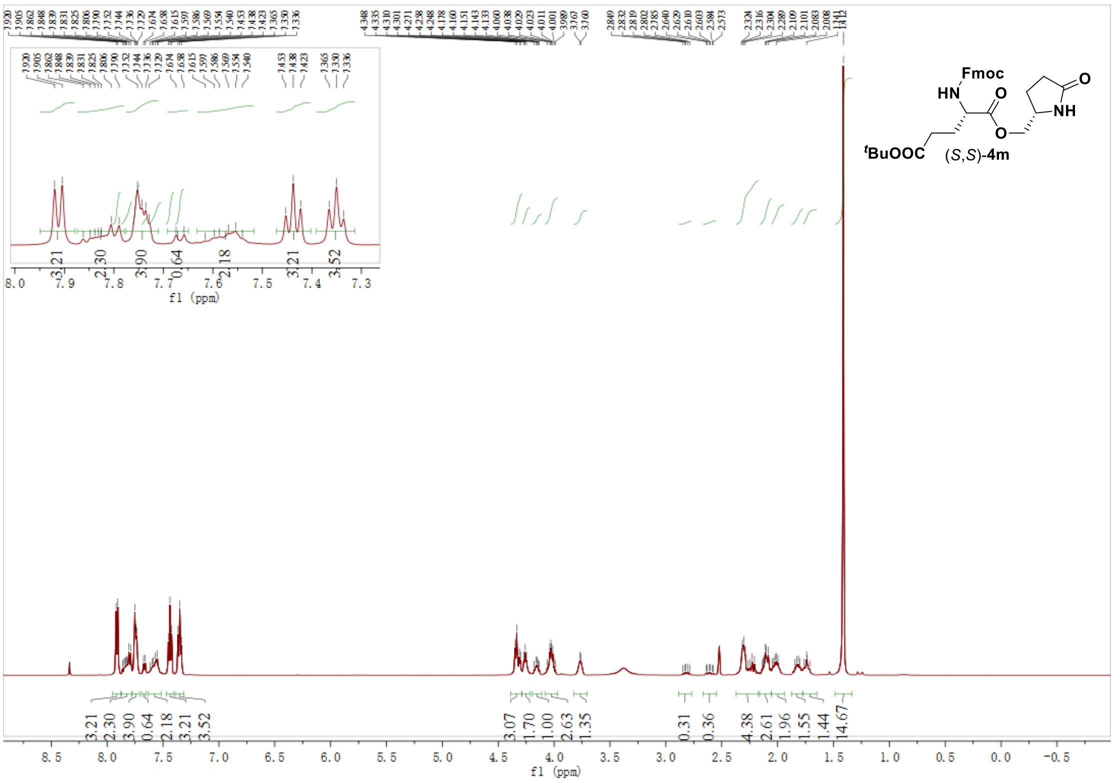

500 MHz, DMSO-d_6, ^{1}H NMR

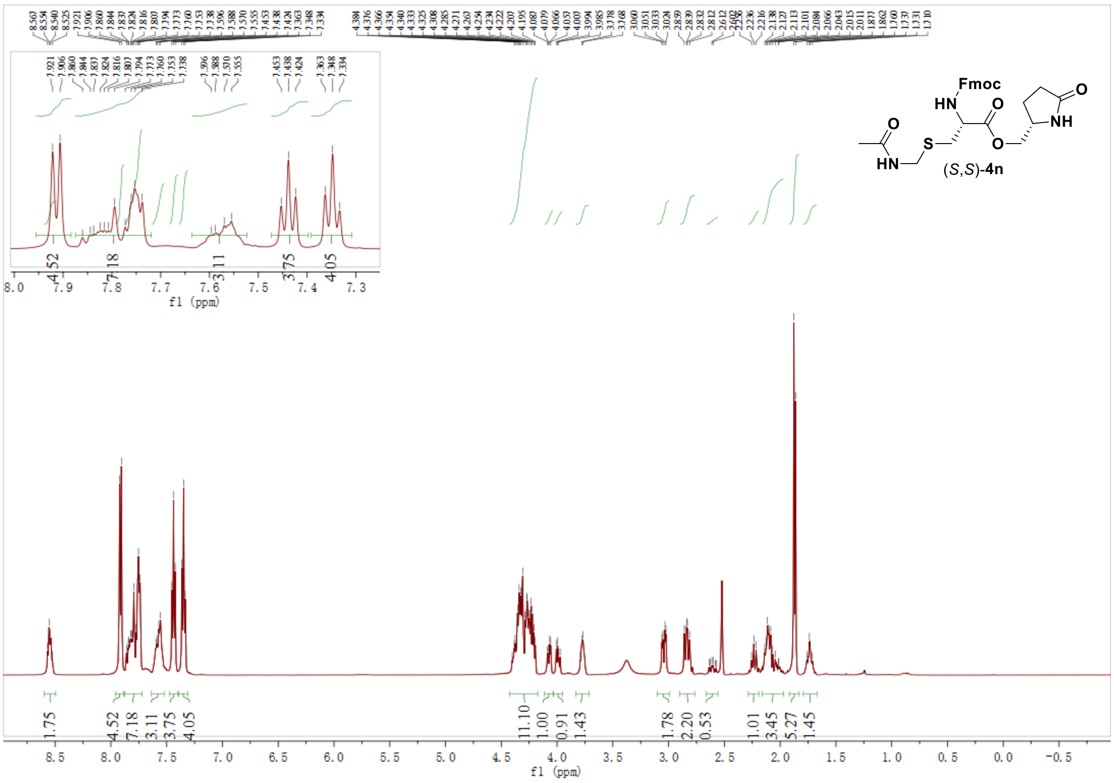

500 MHz, DMSO-d_6, ^{1}H NMR

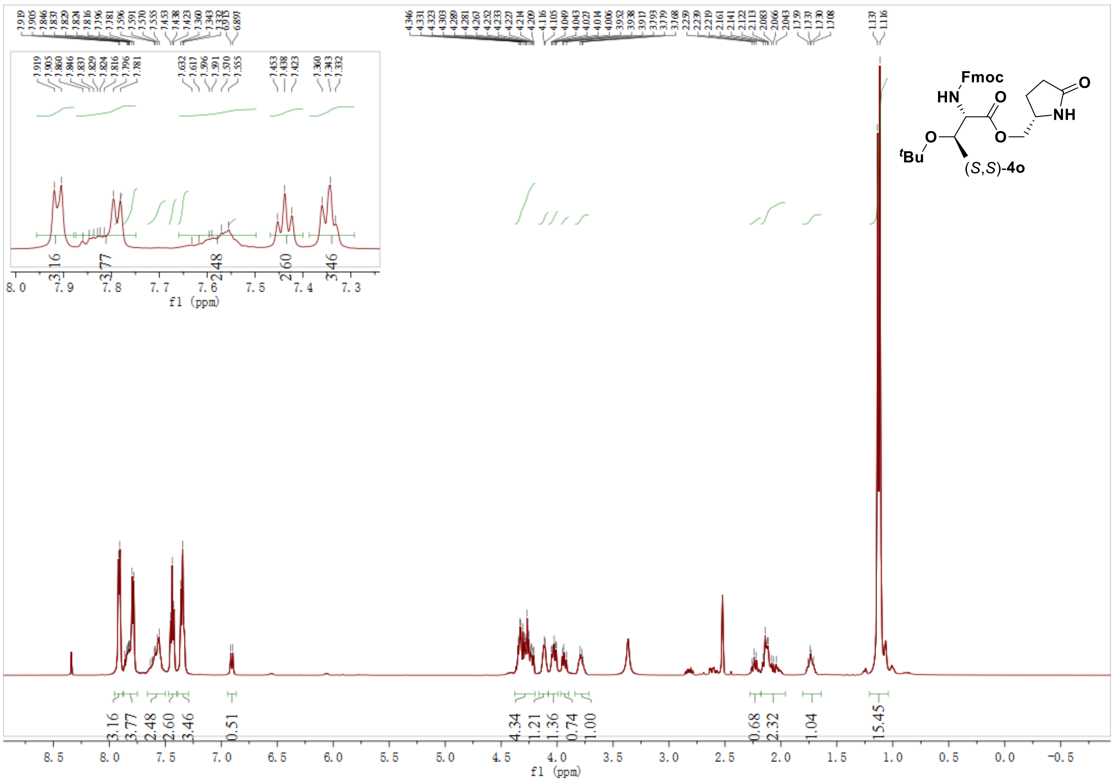

500 MHz, DMSO-d_6, ^{1}H NMR

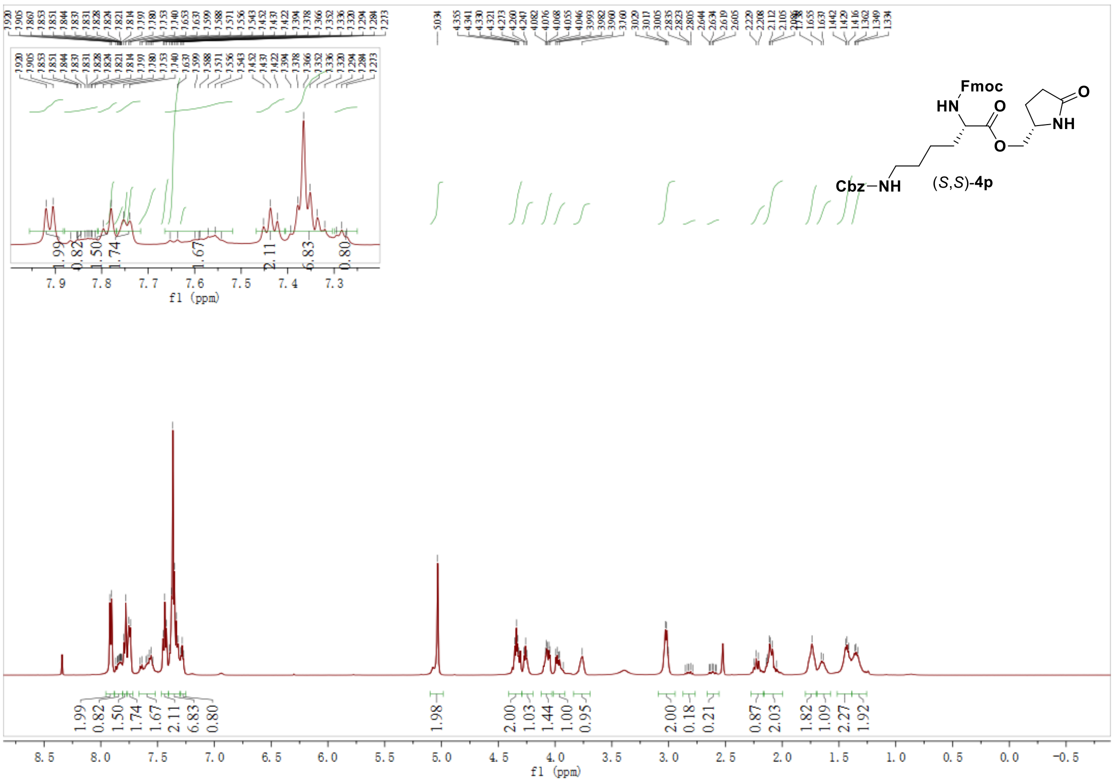

500 MHz, DMSO-d_6, ^{1}H NMR

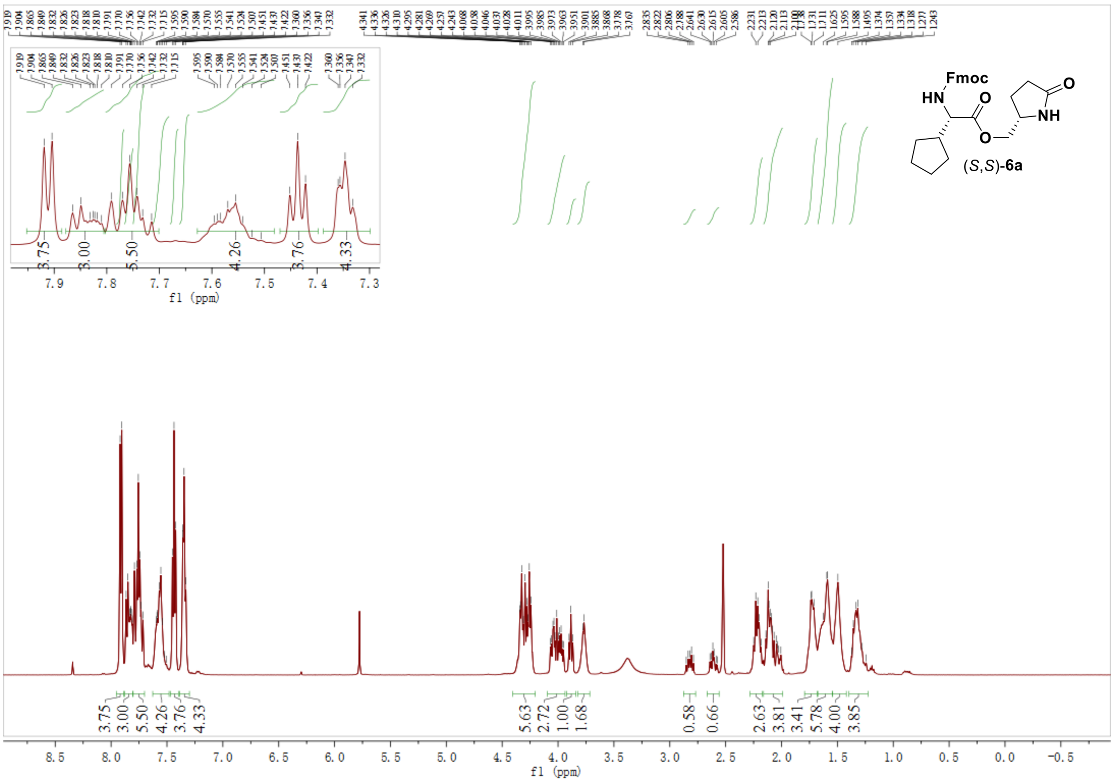

500 MHz, DMSO-d_6, ^{1}H NMR

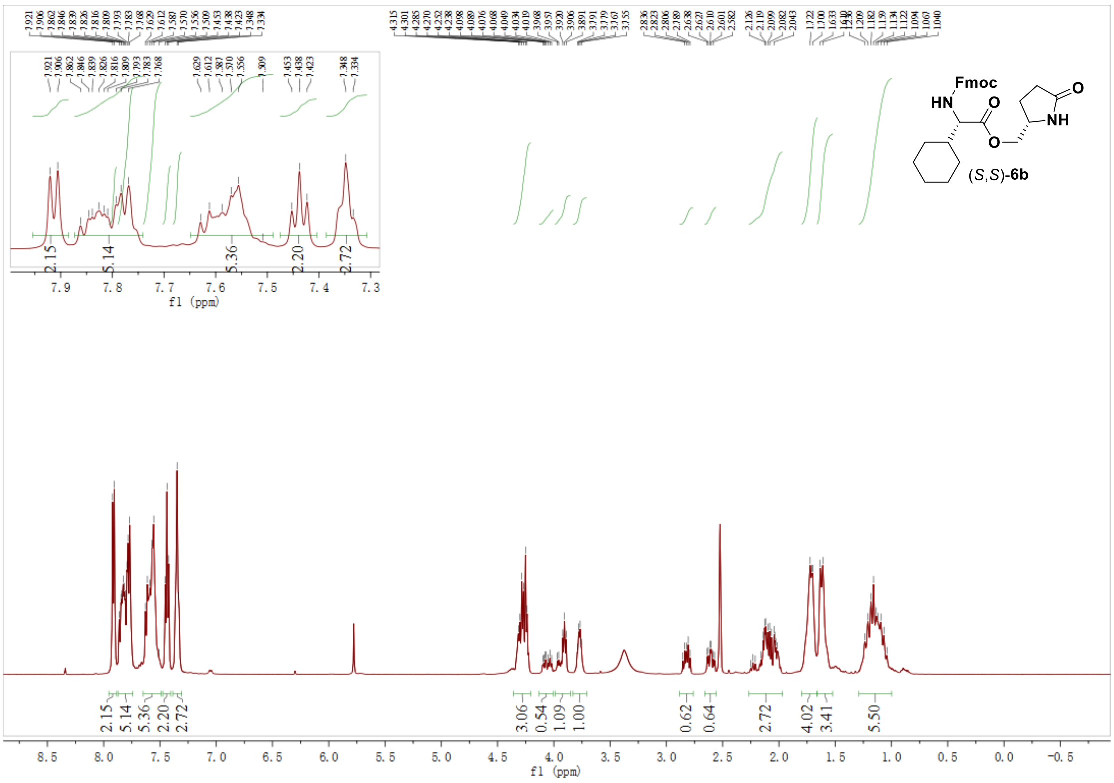

500 MHz, DMSO-d_6, ^{1}H NMR

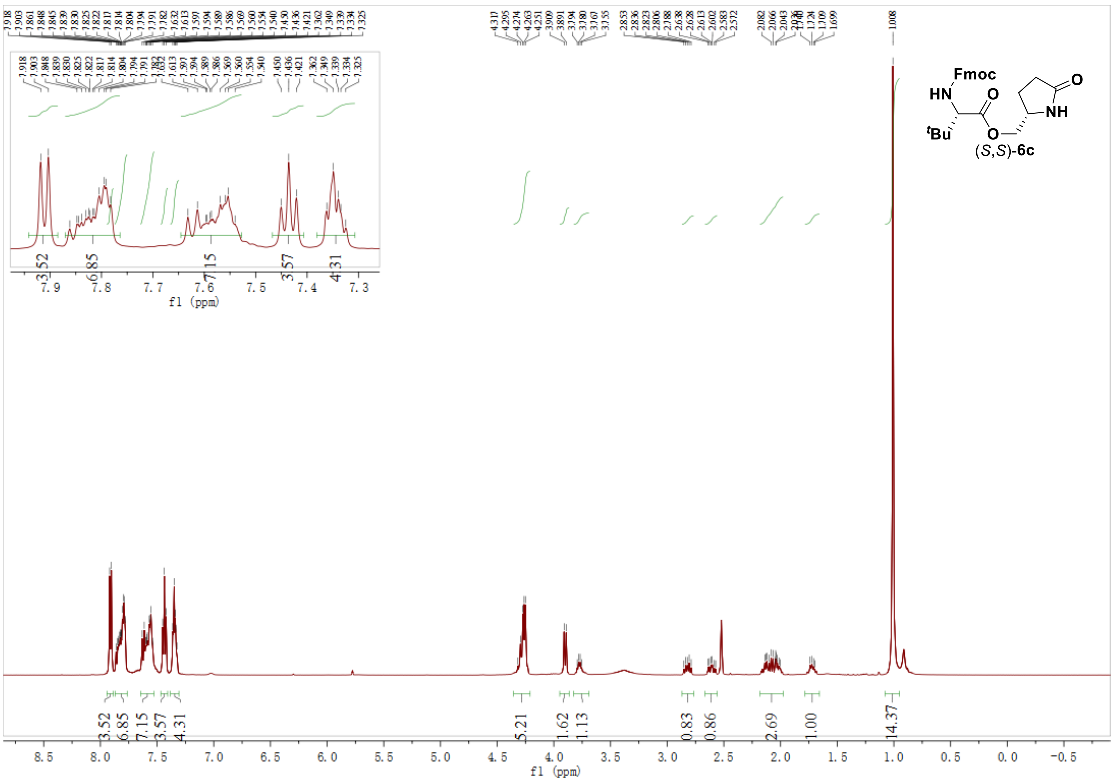

500 MHz, DMSO-d_6, ^{1}H NMR

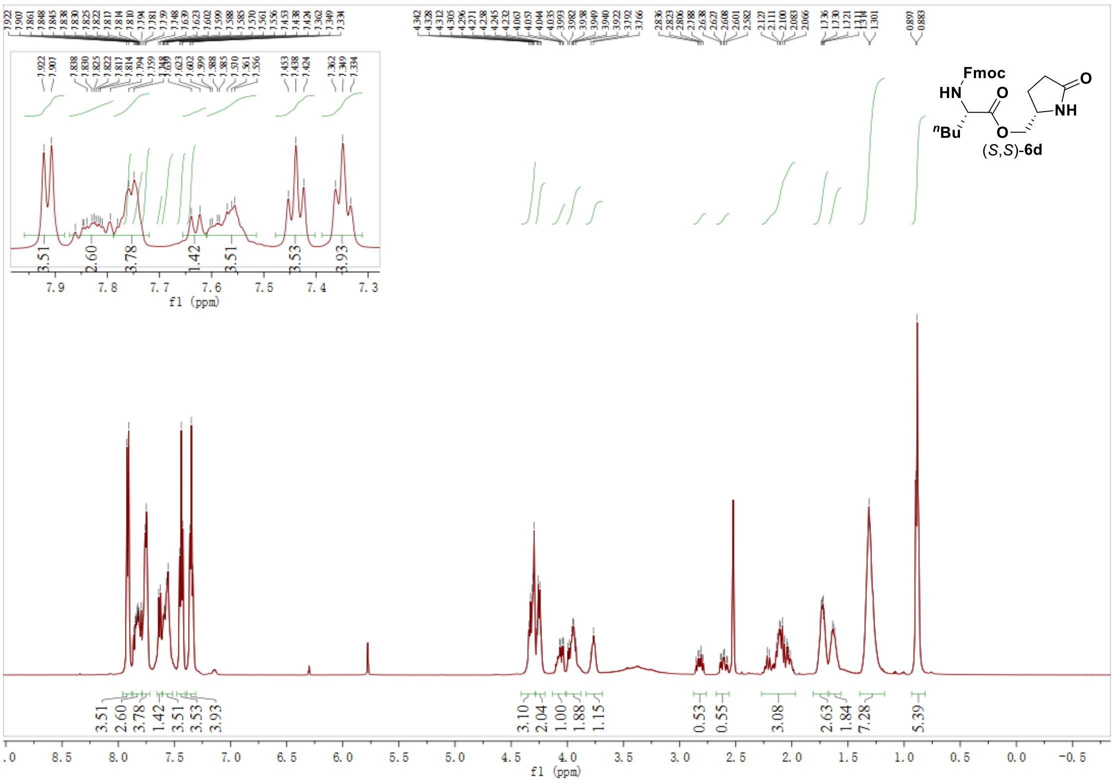

500 MHz, DMSO-d_6, ^{1}H NMR

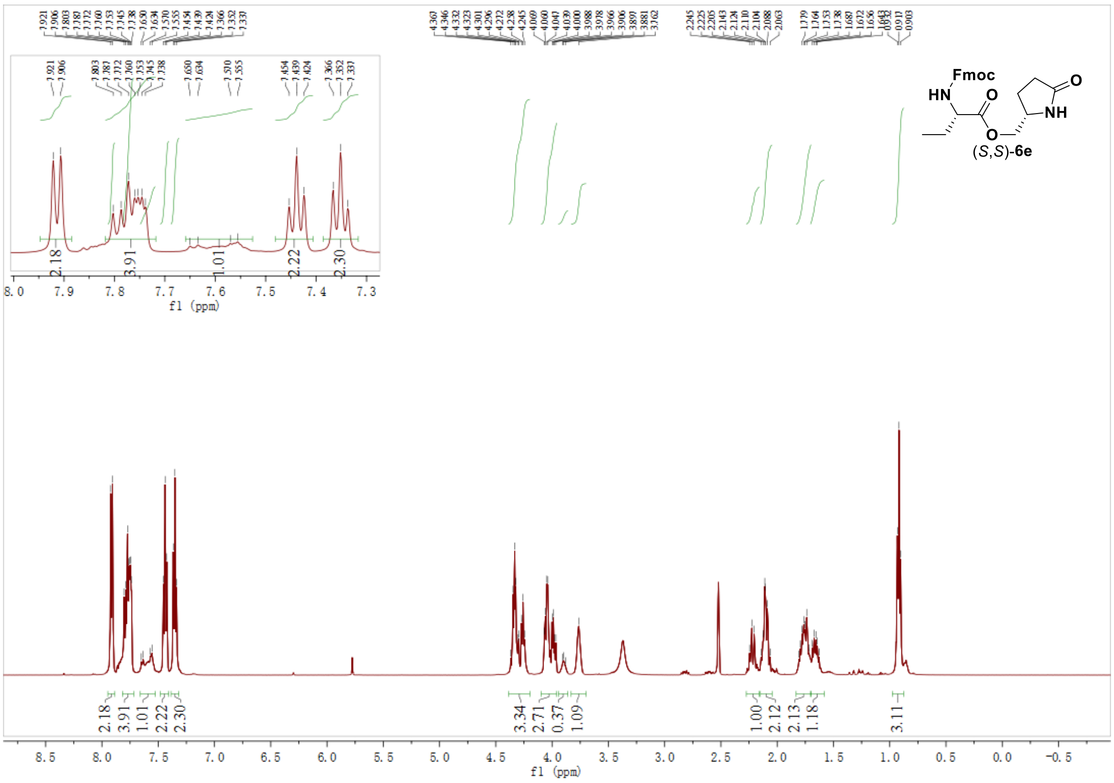

500 MHz, DMSO-d_6, ^{1}H NMR

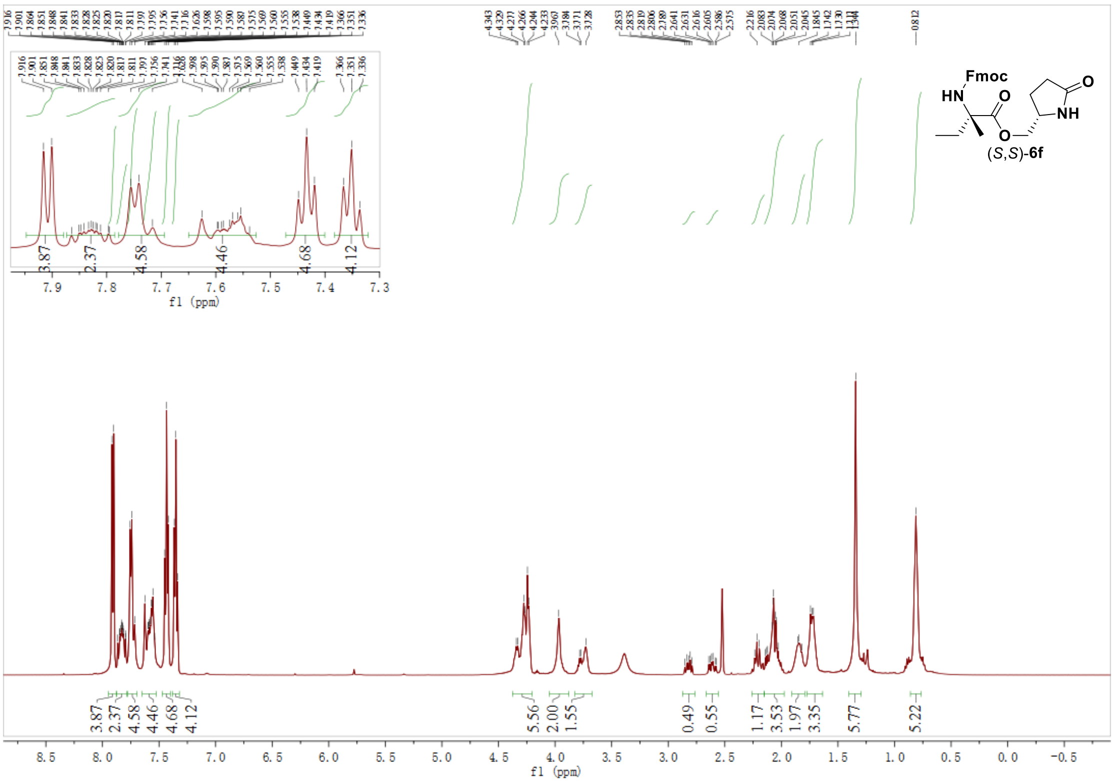

500 MHz, DMSO-d_6, ^{1}H NMR

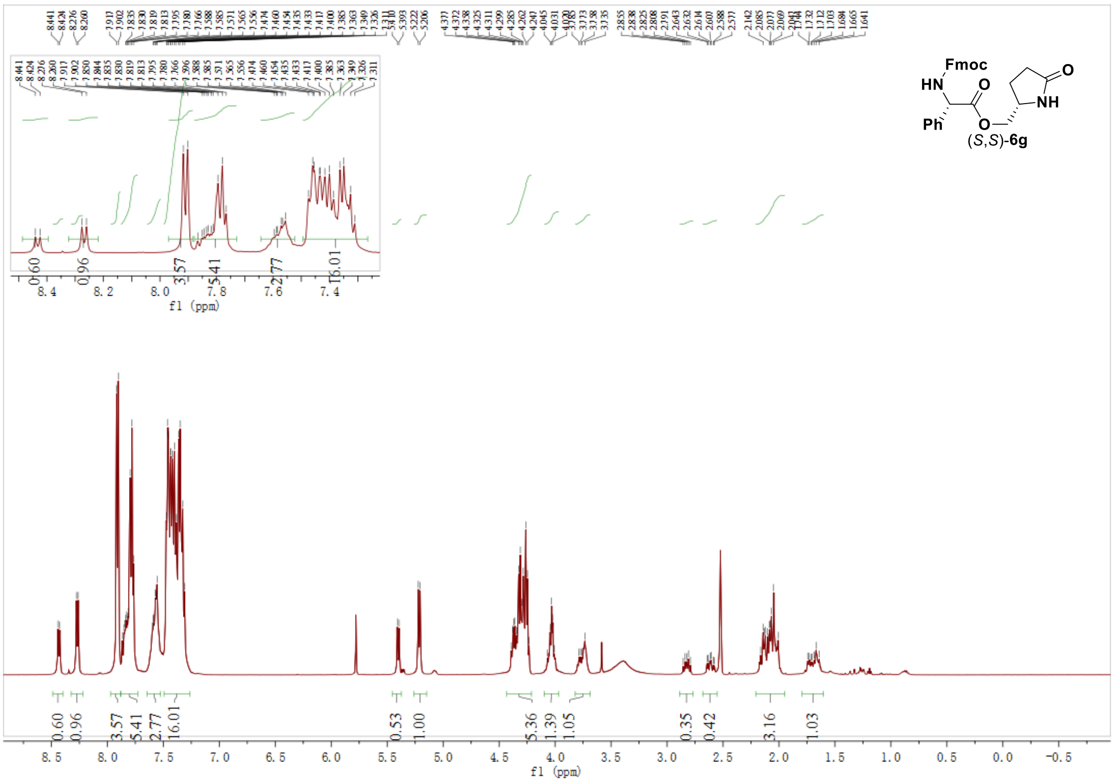

500 MHz, DMSO-d_6, ^{1}H NMR

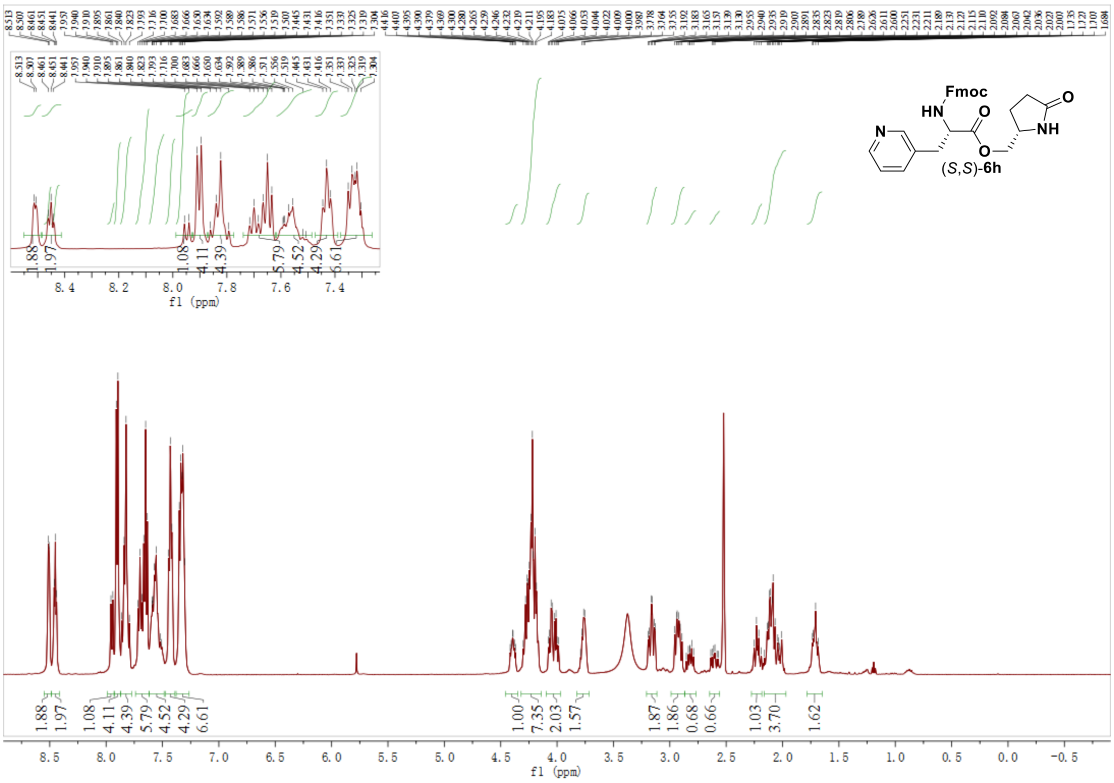

500 MHz, DMSO-d_6, ^{1}H NMR

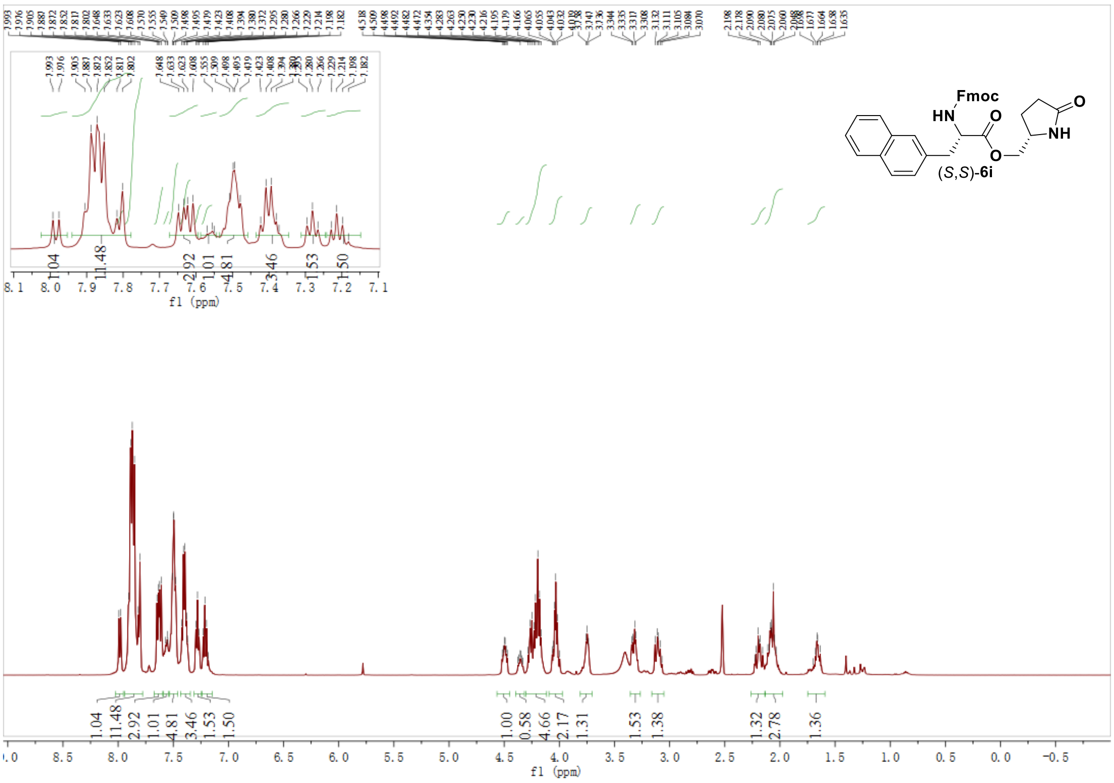

500 MHz, DMSO-d_6, ^{1}H NMR

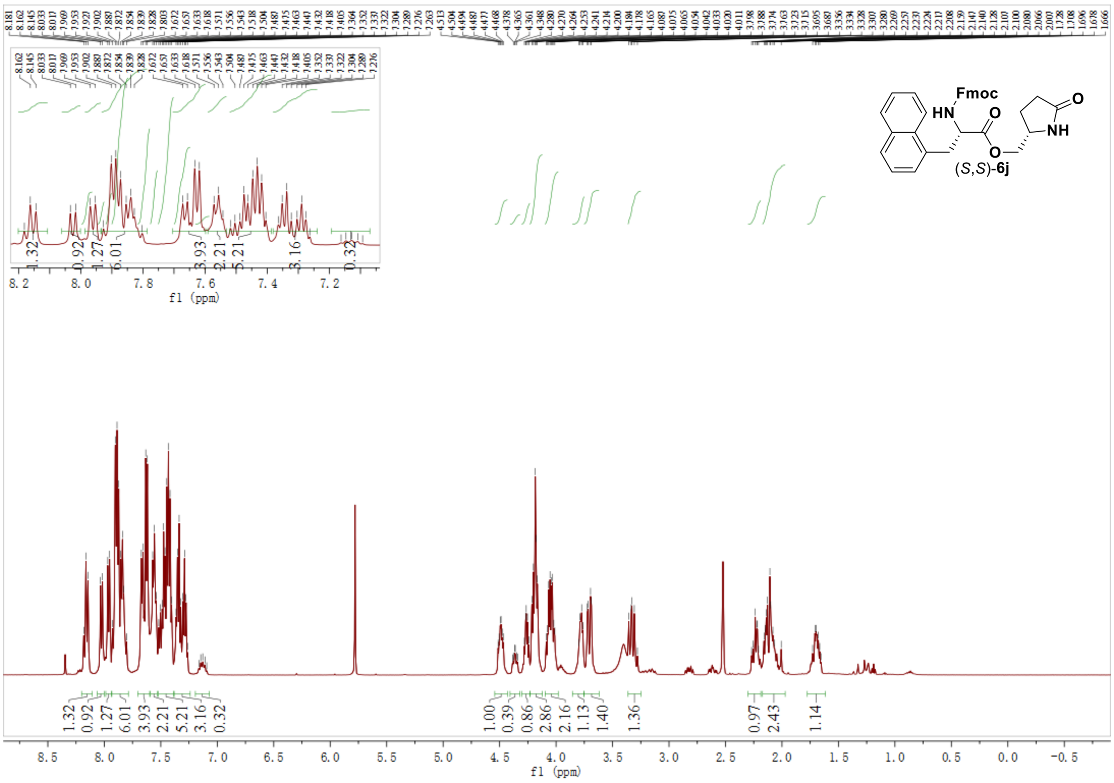

500 MHz, DMSO-d_6, ^{1}H NMR

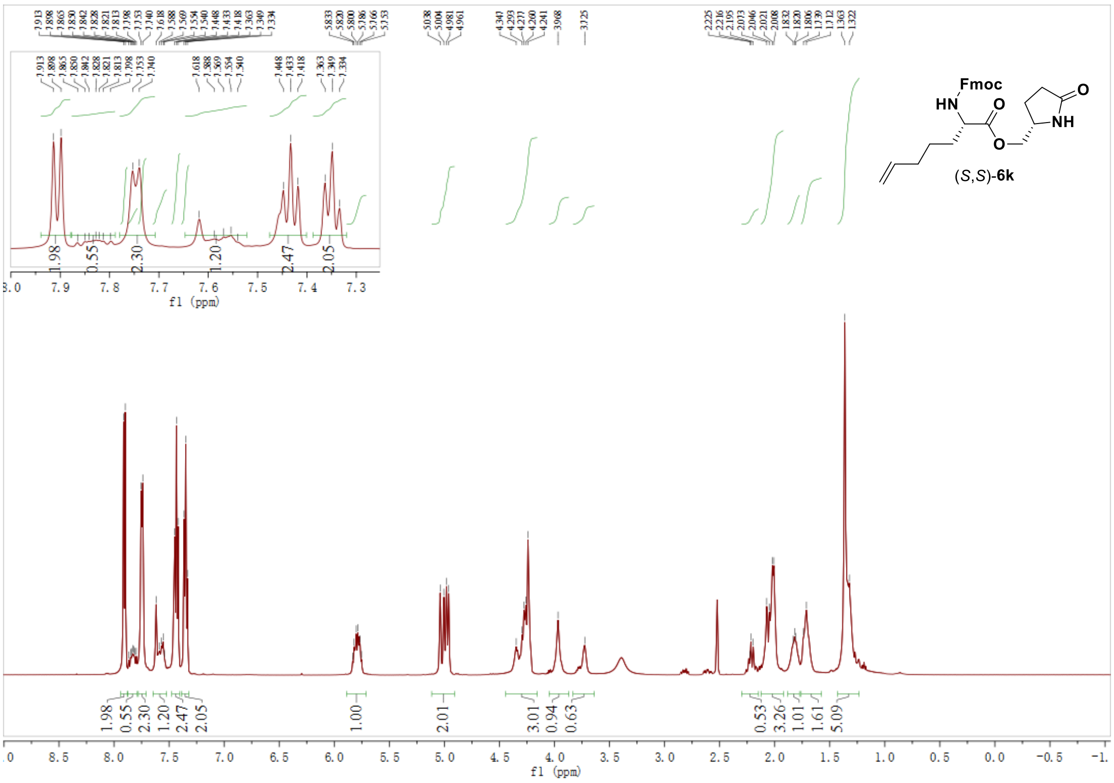

500 MHz, DMSO-d_6, ^{1}H NMR

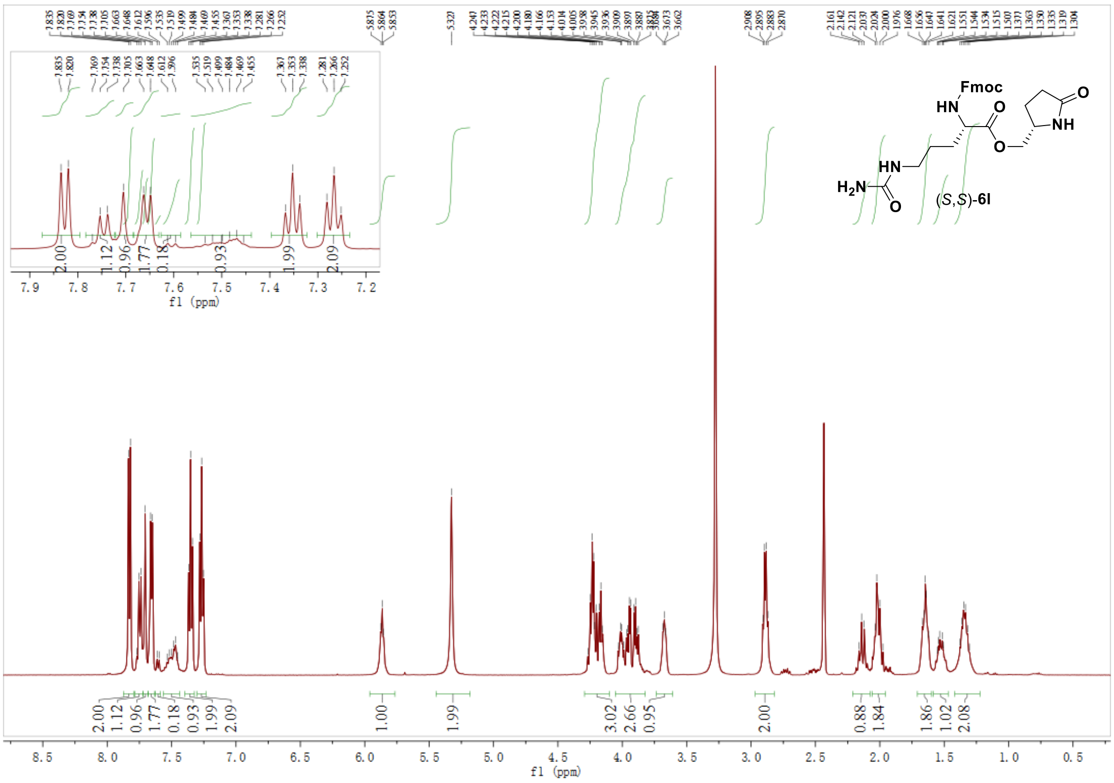

500 MHz, DMSO-d_6, ^{1}H NMR

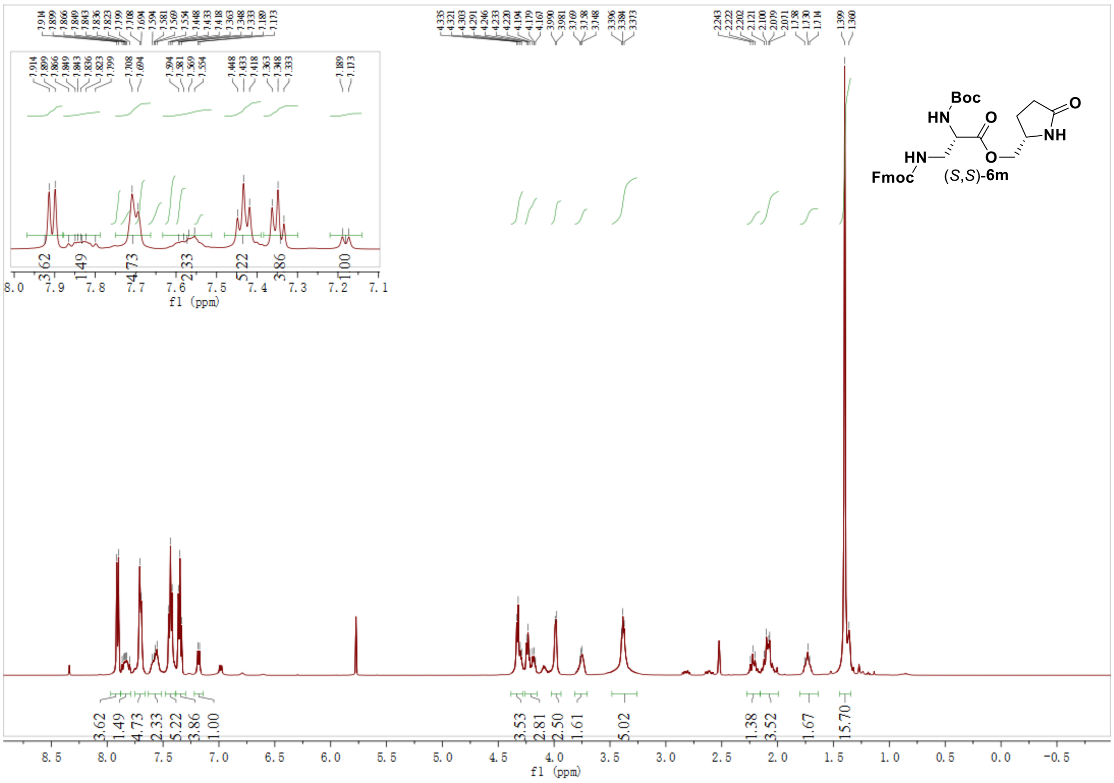

500 MHz, DMSO-d_6, ^{1}H NMR

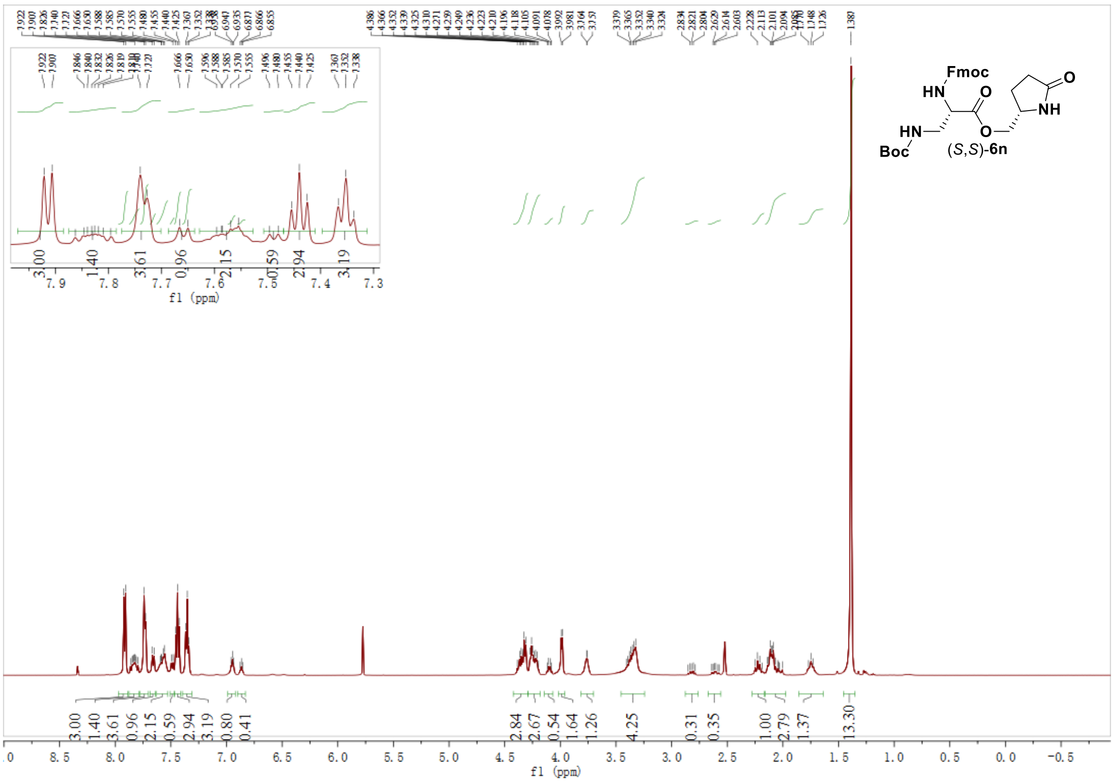

500 MHz, DMSO-d_6, ^{1}H NMR

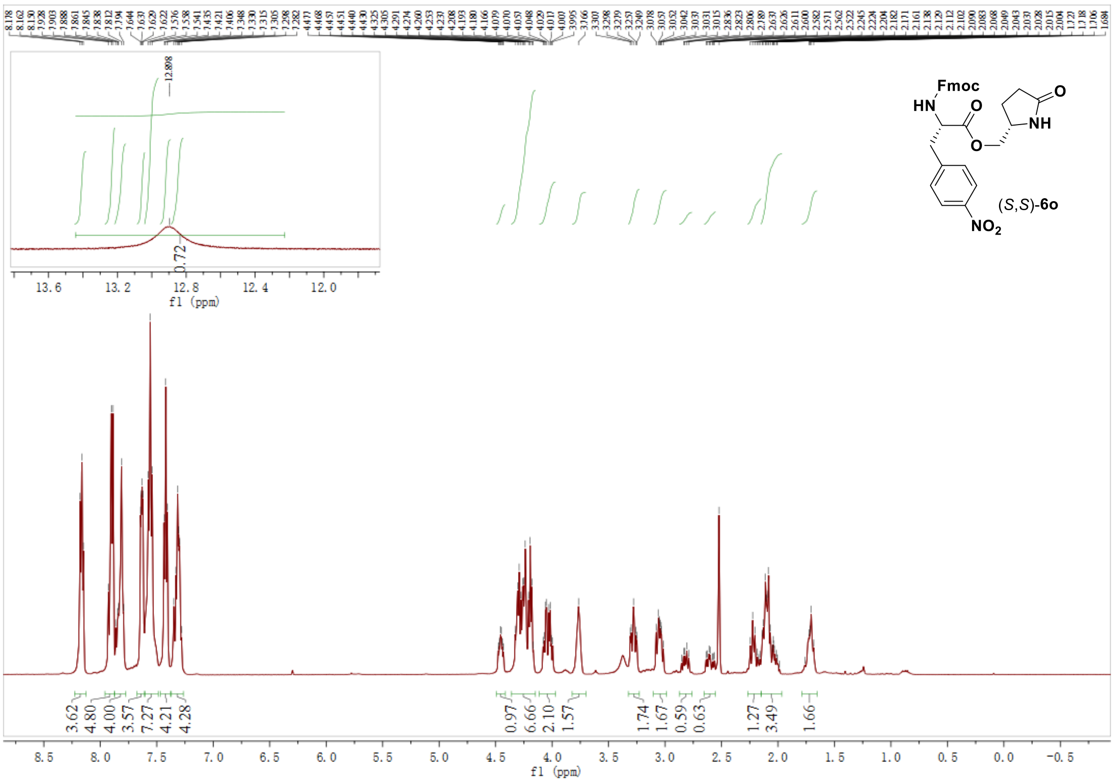

500 MHz, DMSO-d_6, ^{1}H NMR

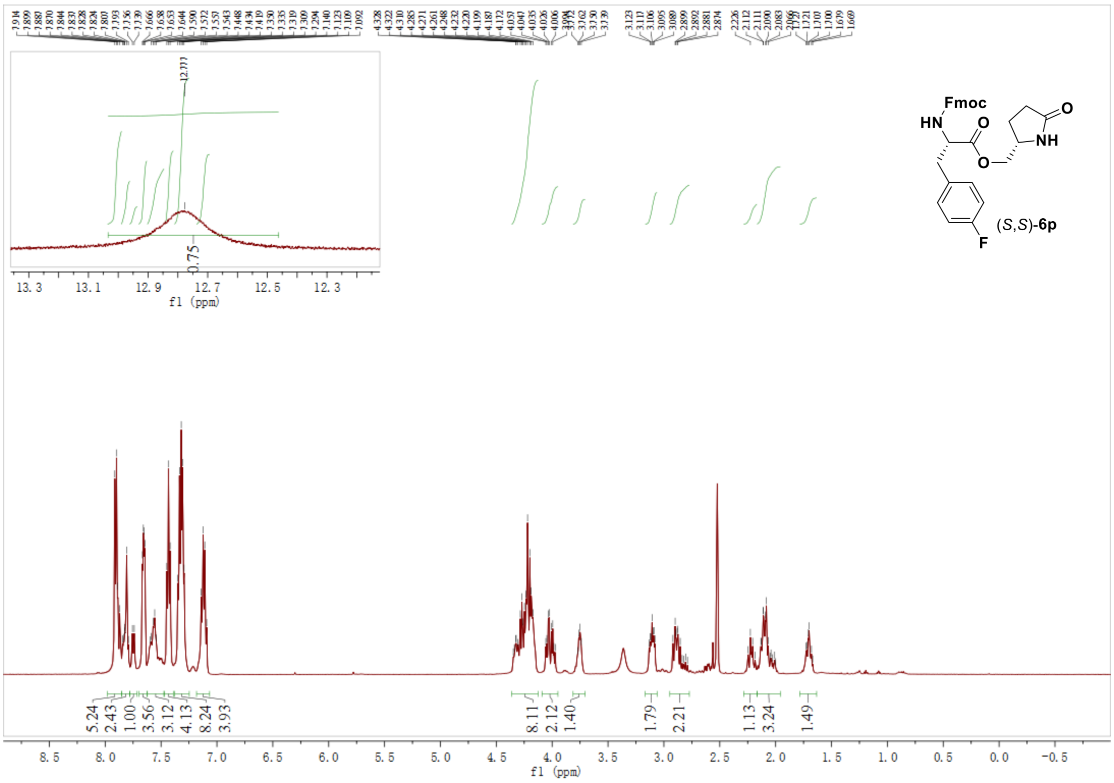

500 MHz, DMSO-d_6, ^{1}H NMR

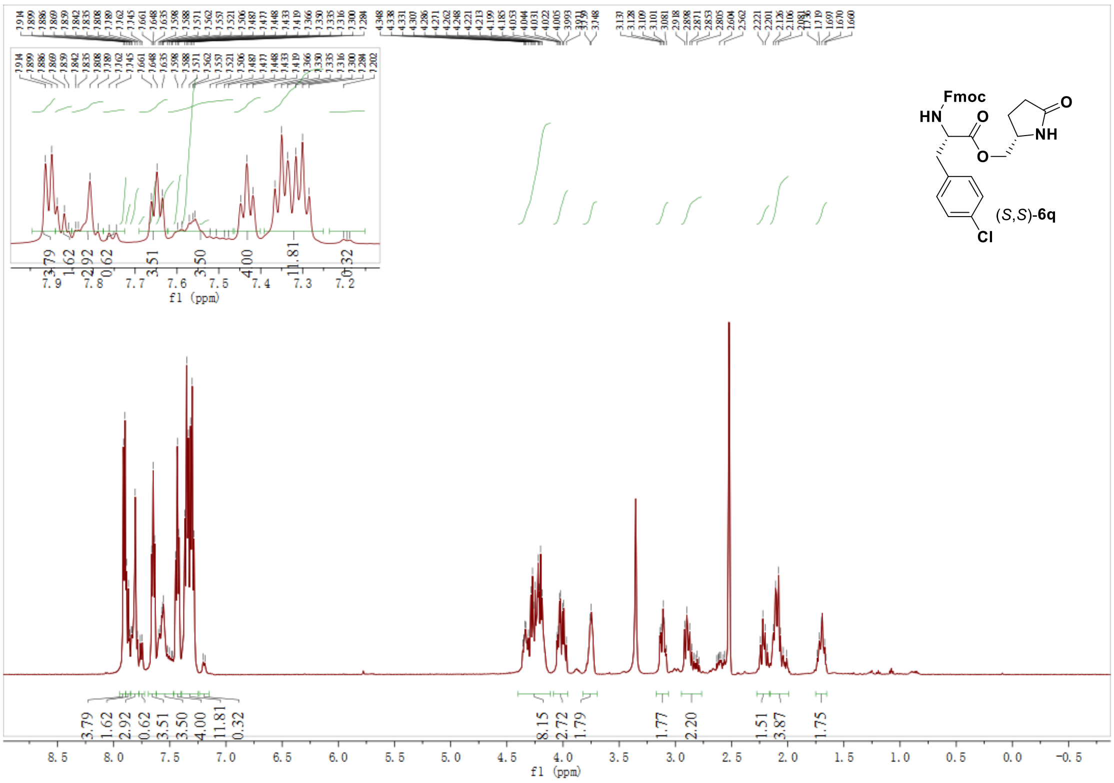

500 MHz, DMSO-d_6, ^{1}H NMR

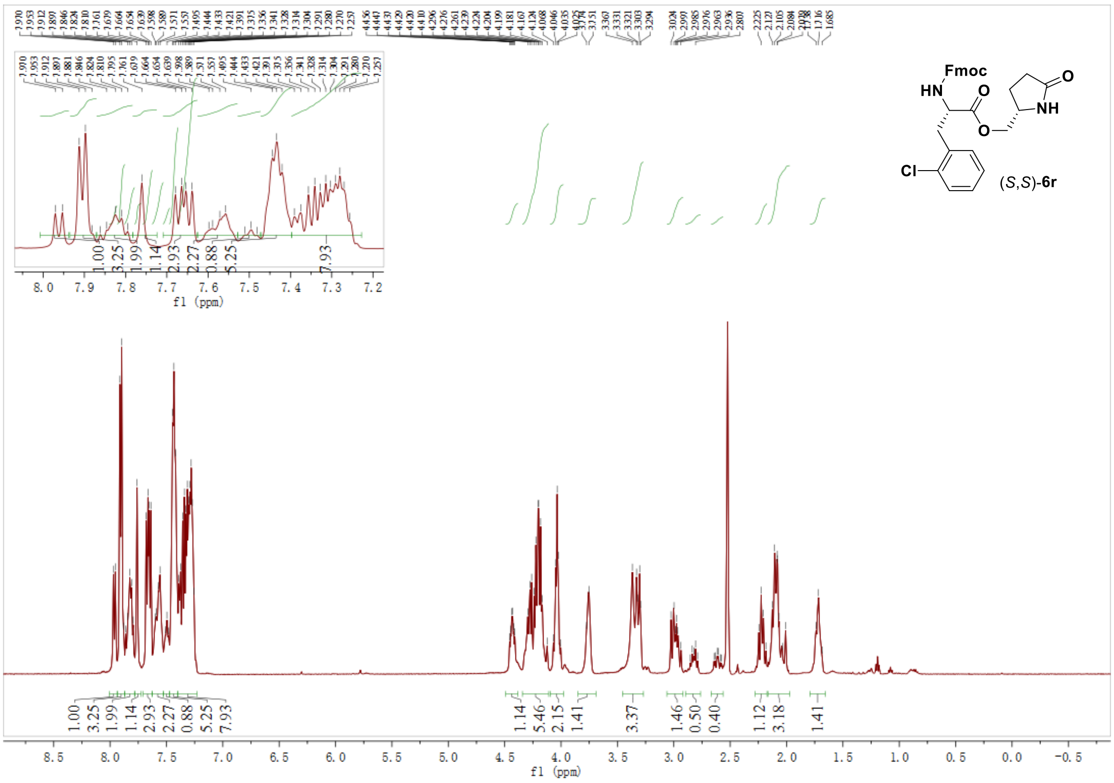

500 MHz, DMSO-d_6, ^1H NMR

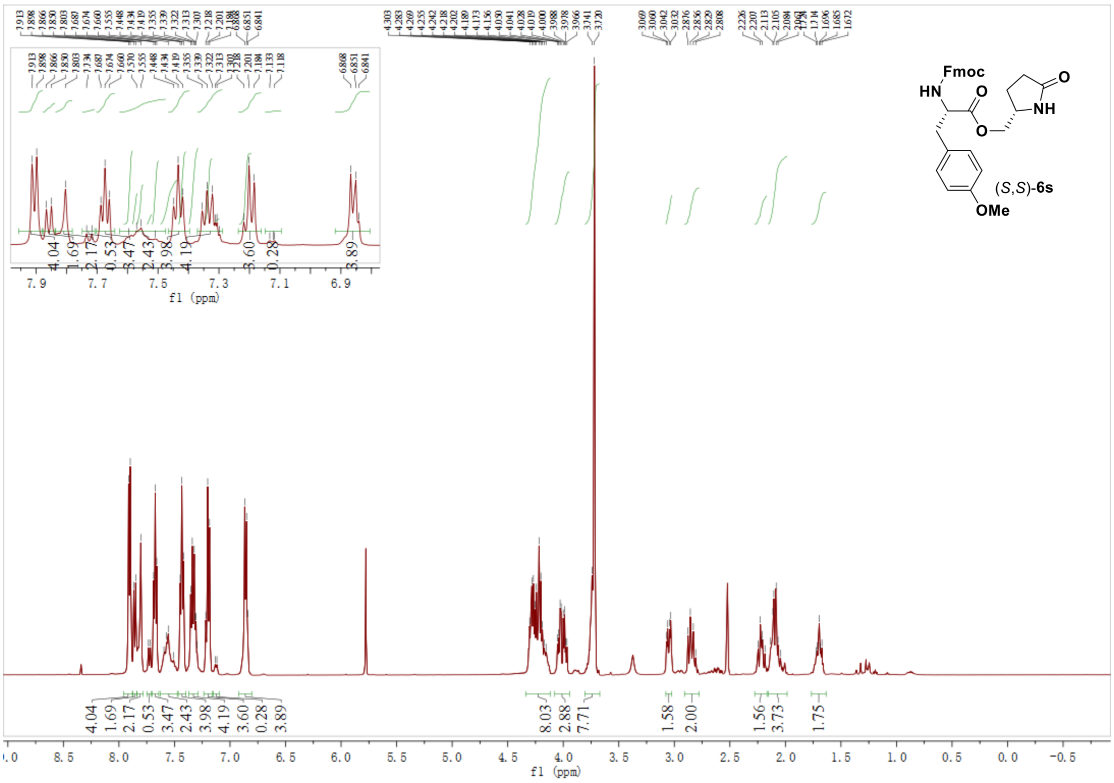

500 MHz, DMSO-d_6, ^1H NMR

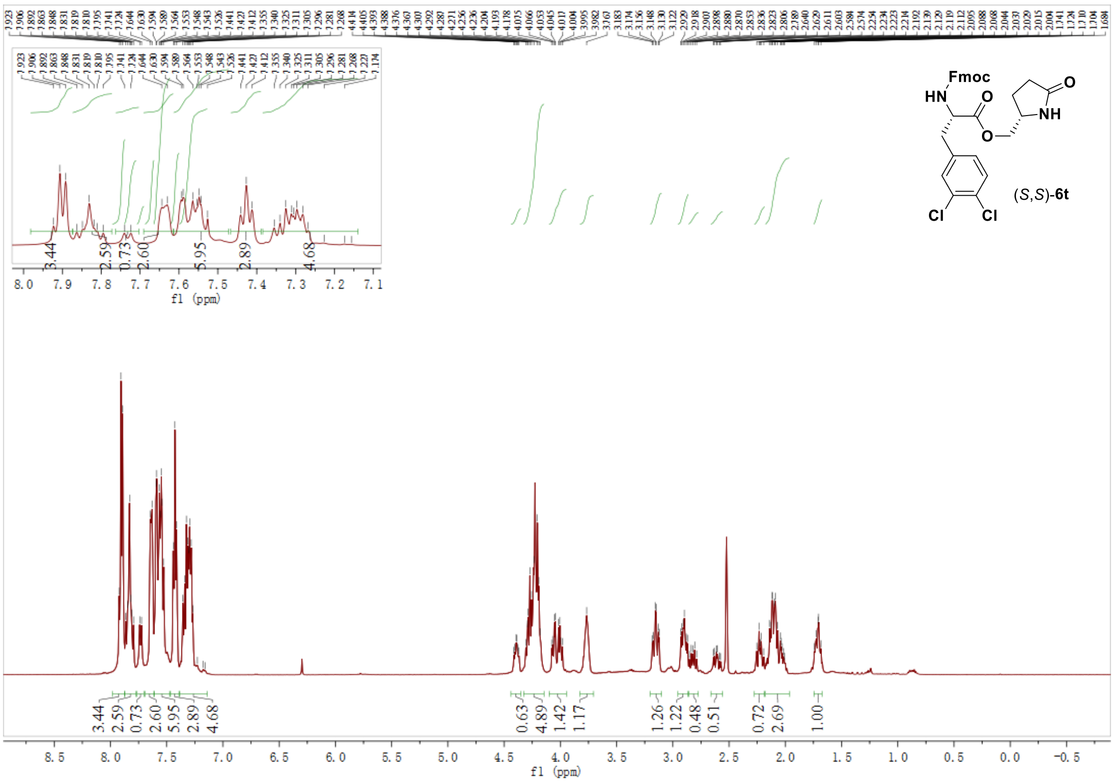

5. Chiral HPLC Spectra

HPLC chromatogram of racemic **4a**

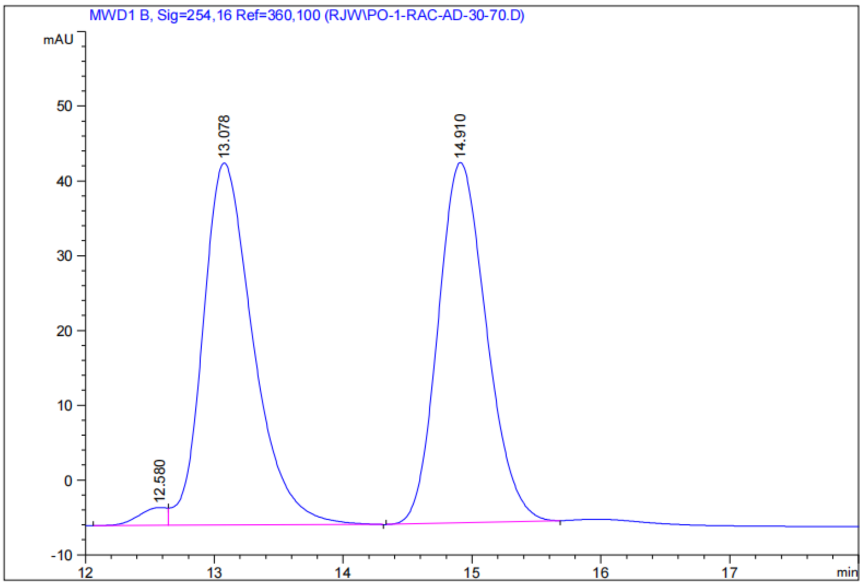

RT [min]	Type	Width [min]	Area	Height	Area%
12.580	BV	0.2325	35.82310	2.39463	1.3774
13.078	VB	0.4119	1311.29846	48.42427	50.4180
14.910	BB	0.4035	1253.73096	48.19933	48.2046

HPLC chromatogram of enantiopure **4a**

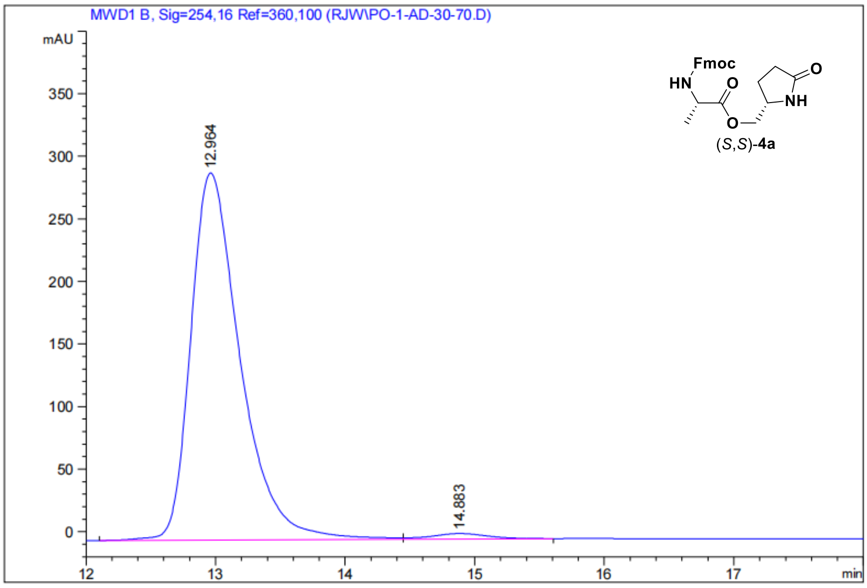

RT [min]	Type	Width [min]	Area	Height	Area%
12.964	BV	0.3993	7780.32520	293.43494	98.3554
14.883	VB	0.4391	130.09506	4.37284	1.6446

HPLC chromatogram of racemic **4b**

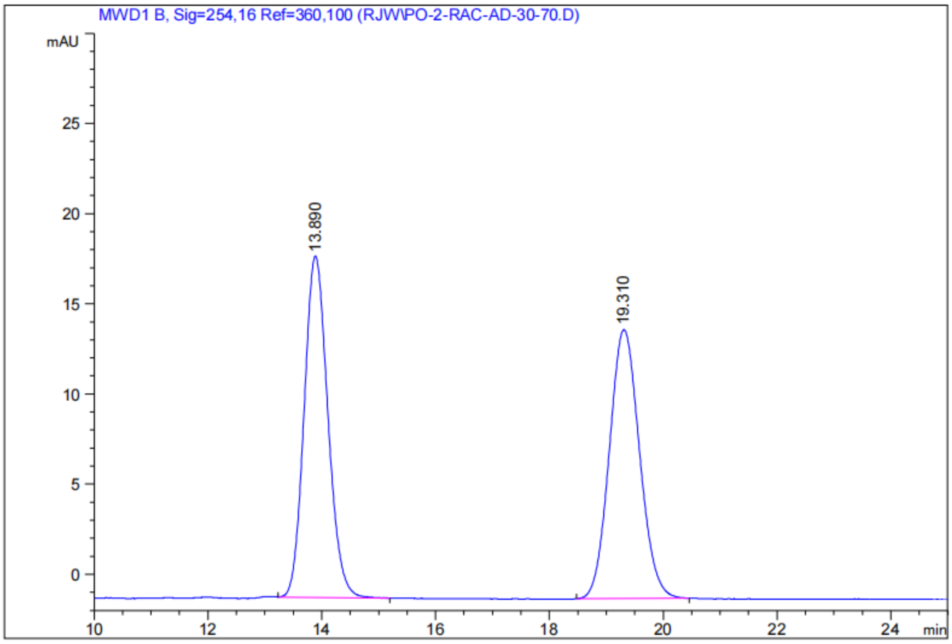

RT [min]	Type	Width [min]	Area	Height	Area%
13.890	BB	0.4453	547.21411	18.92940	49.8915
19.310	BB	0.5779	549.59375	14.91663	50.1085

HPLC chromatogram of enantiopure **4b**

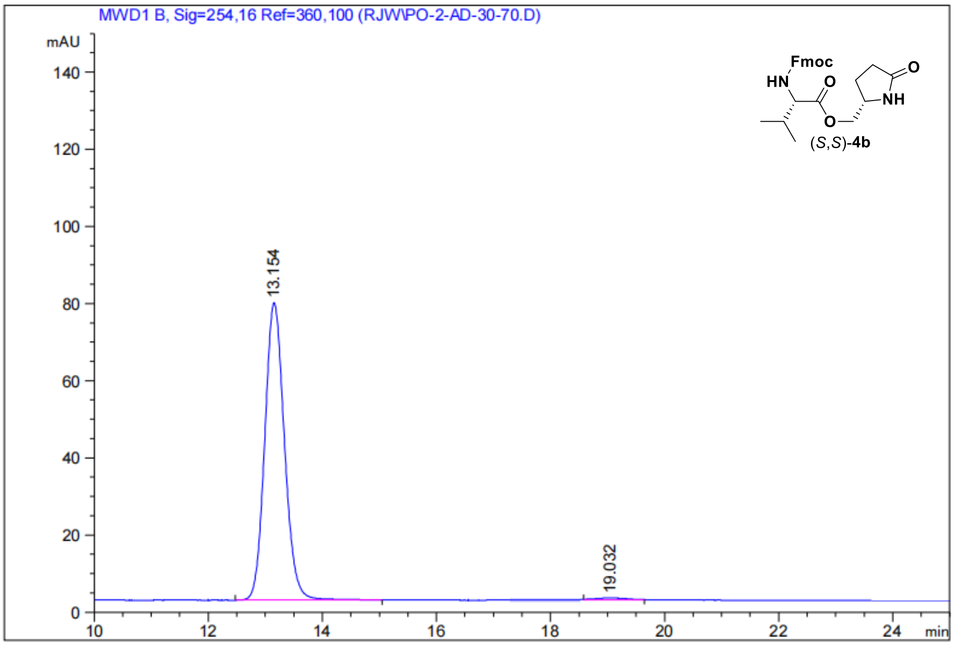

RT [min]	Type	Width [min]	Area	Height	Area%
13.154	BB	0.3693	1845.10779	77.07438	99.0390
19.032	MM R	0.6285	17.90414	4.74799e-1	0.9610

HPLC chromatogram of racemic **4c**

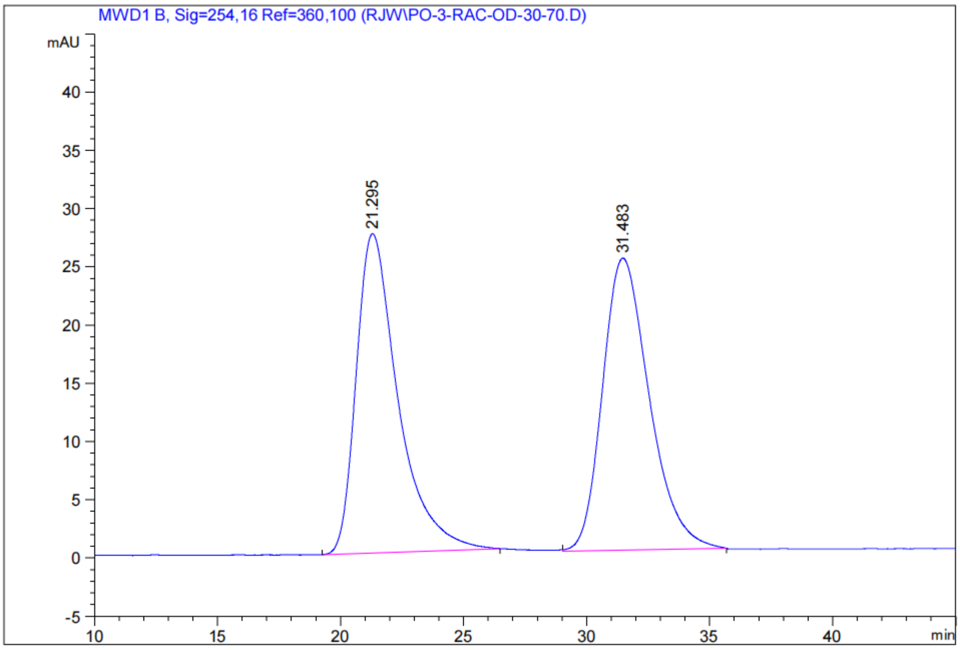

RT [min]	Type	Width [min]	Area	Height	Area%
21.295	BB	1.5305	3240.67773	27.42747	49.4743
31.483	MM R	2.1995	3309.54517	25.07781	50.5257

HPLC chromatogram of enantiopure **4c**

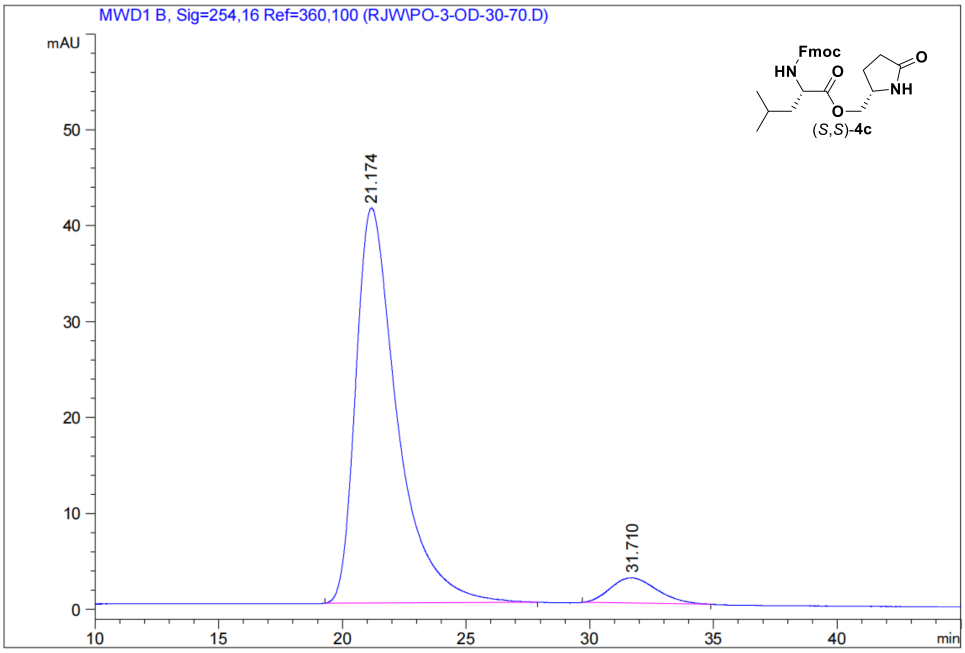

RT [min]	Type	Width [min]	Area	Height	Area%
21.174	BB	1.6363	4808.50928	41.17937	93.3181
31.710	BB	1.5254	344.30771	2.65066	6.6819

HPLC chromatogram of racemic **4d**

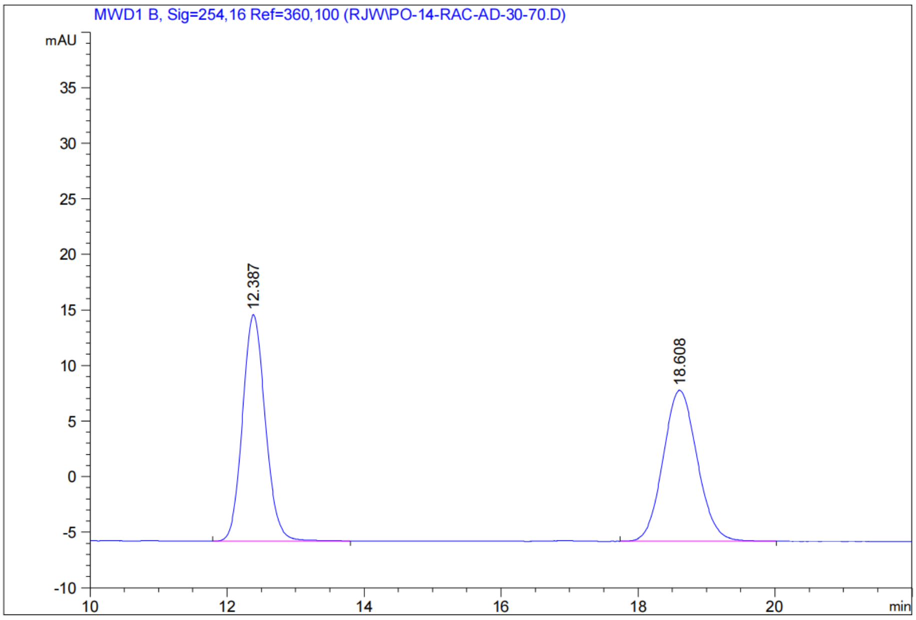

RT [min]	Type	Width [min]	Area	Height	Area%
12.387	BB	0.3601	471.70801	20.37651	50.0824
18.608	BB	0.5335	470.15533	13.59371	49.9176

HPLC chromatogram of enantiopure **4d**

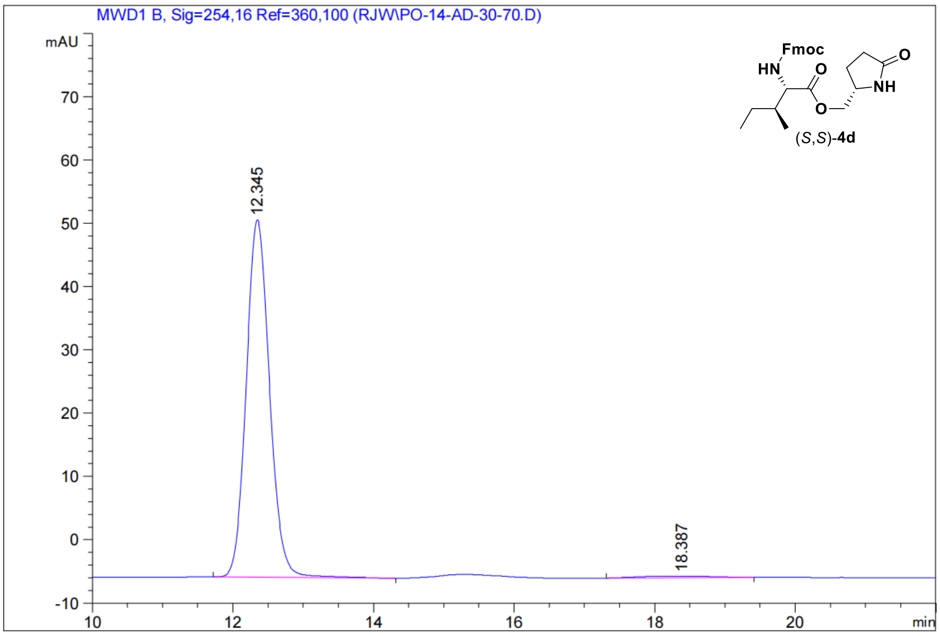

RT [min]	Type	Width [min]	Area	Height	Area%
12.345	BB	0.3567	1300.86536	56.48723	98.5268
18.387	MM R	1.2703	19.45042	2.55188e-1	1.4732

HPLC chromatogram of racemic **4e**

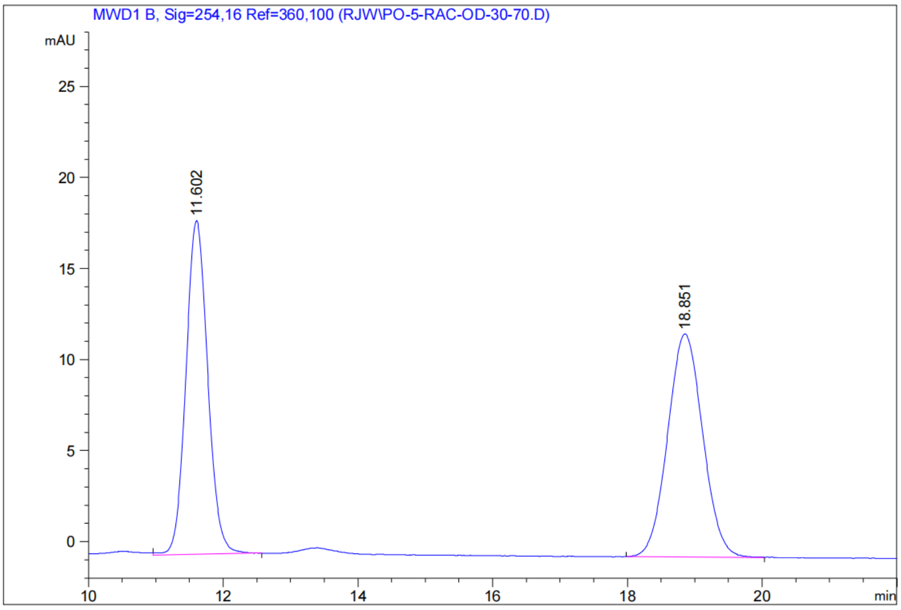

RT [min]	Type	Width [min]	Area	Height	Area%
11.602	MM R	0.3744	411.65594	18.32298	48.1638
18.851	BB	0.5480	443.04443	12.24939	51.8362

HPLC chromatogram of enantiopure **4e**

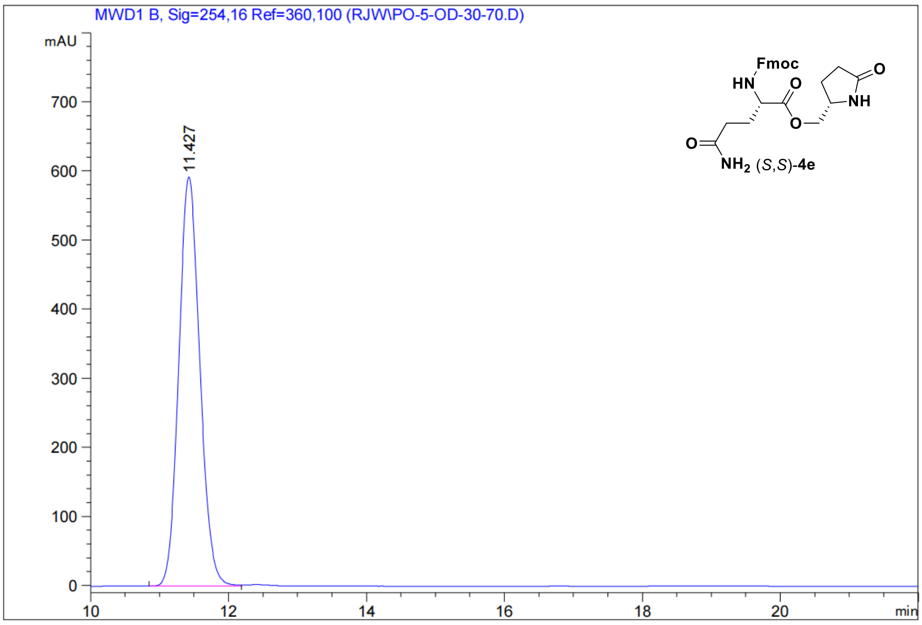

RT [min]	Type	Width [min]	Area	Height	Area%
11.427	BV	0.3293	1.25457e4	591.81616	100.0000

HPLC chromatogram of racemic **4f**

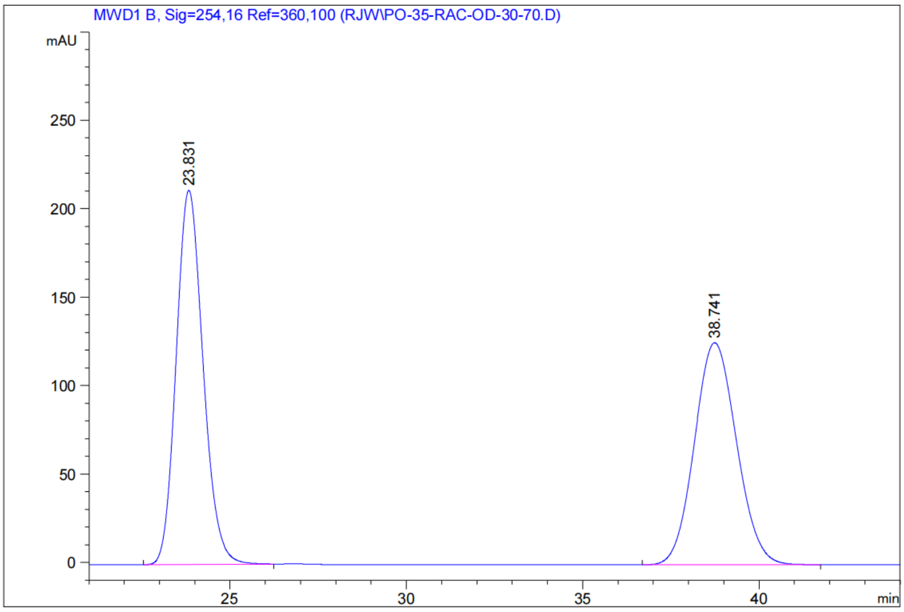

RT [min]	Type	Width [min]	Area	Height	Area%
23.831	BB	0.8185	1.11821e4	211.61855	51.7203
38.741	BB	1.2917	1.04382e4	125.57852	48.2797

HPLC chromatogram of enantiopure **4f**

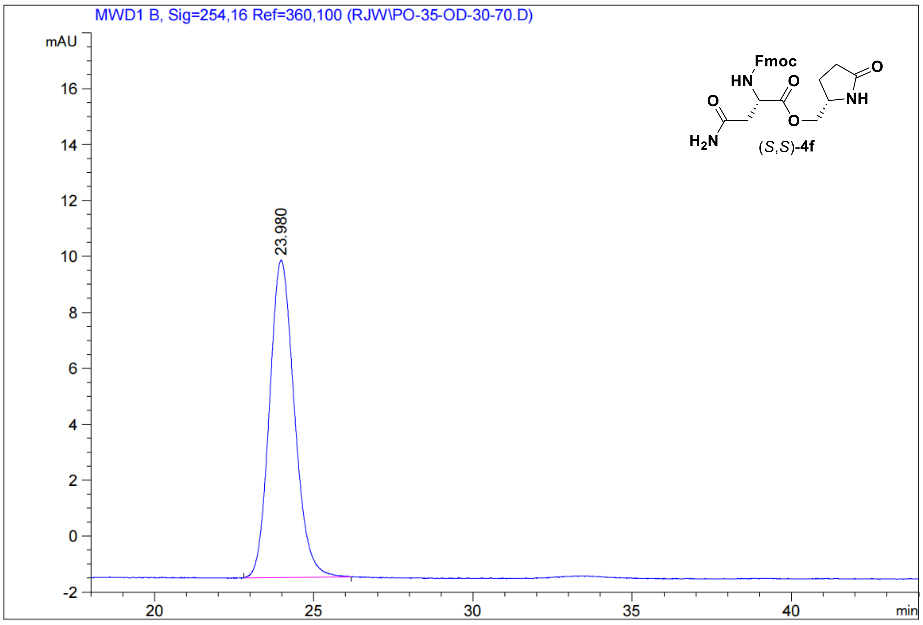

RT [min]	Type	Width [min]	Area	Height	Area%
23.980	BB	0.7378	616.16180	11.34215	100.0000

HPLC chromatogram of racemic **4g**

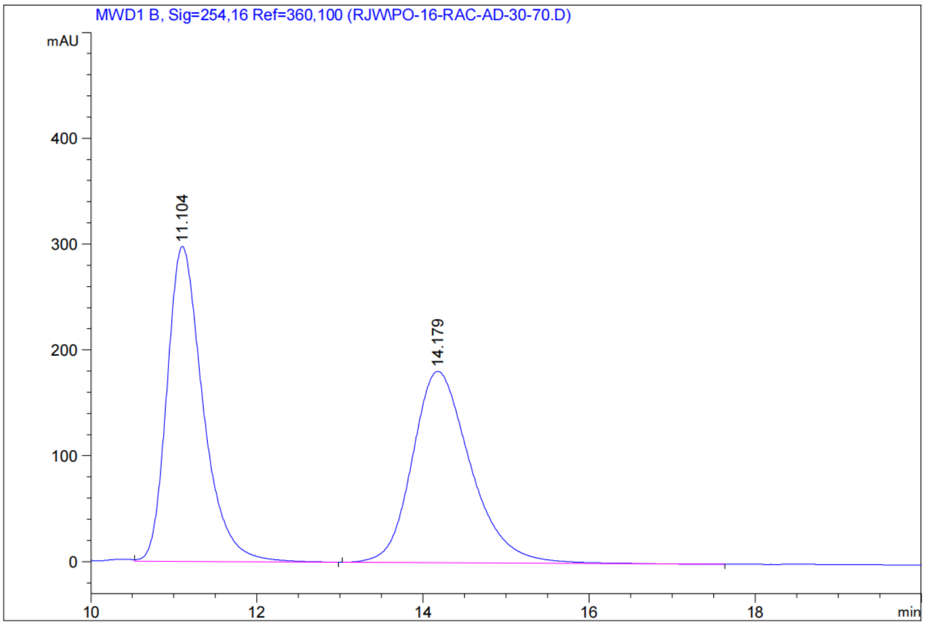

RT [min]	Type	Width [min]	Area	Height	Area%
11.104	VB	0.4577	8964.09375	297.39139	50.4190
14.179	BB	0.7425	8815.11035	180.90050	49.5810

HPLC chromatogram of enantiopure **4g**

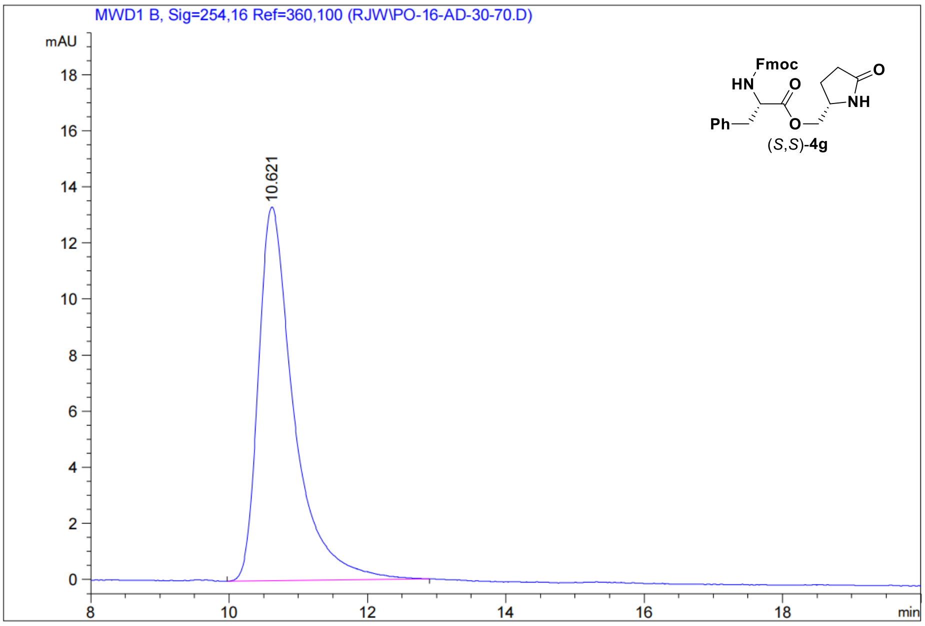

RT [min]	Type	Width [min]	Area	Height	Area%
10.621	BB	0.5296	474.46643	13.32063	100.0000

HPLC chromatogram of racemic **4h**

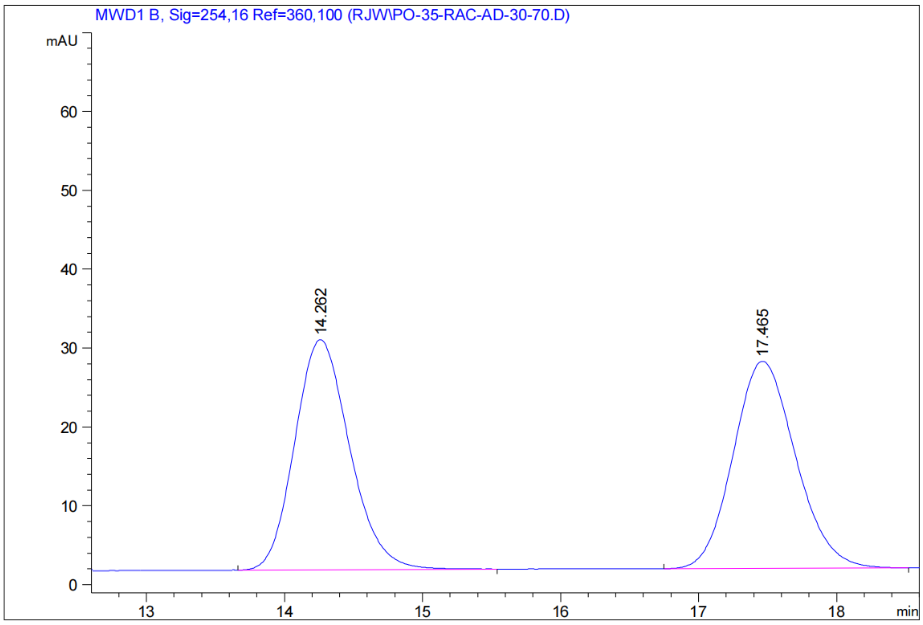

RT [min]	Type	Width [min]	Area	Height	Area%
14.262	BB	0.4286	805.93860	29.14602	49.3630
17.465	BB	0.4891	826.74017	26.27270	50.6370

HPLC chromatogram of enantiopure **4h**

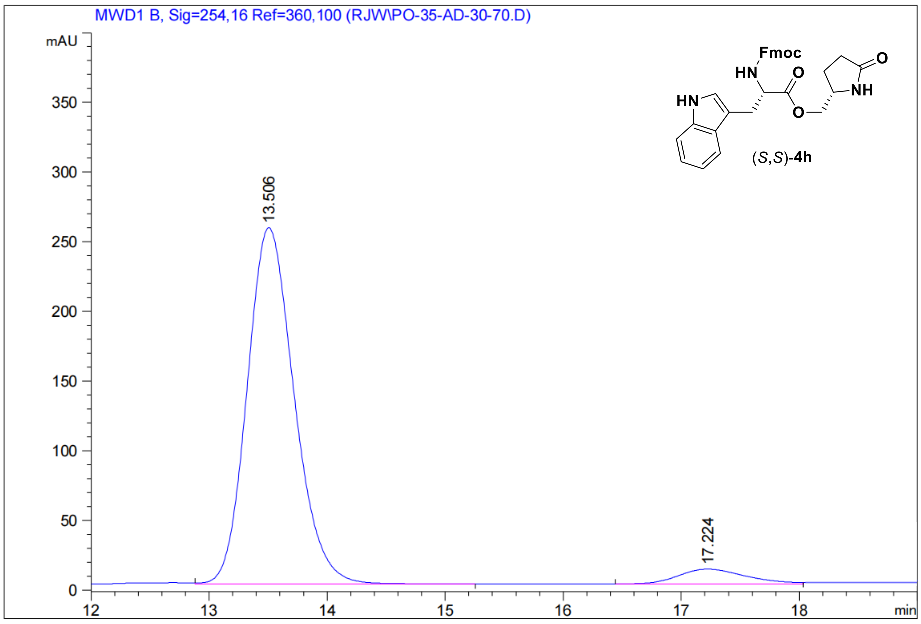

RT [min]	Type	Width [min]	Area	Height	Area%
13.506	VB	0.4262	7066.07861	255.87459	94.3414
17.224	BV	0.5882	423.82571	10.65205	5.6586

HPLC chromatogram of racemic **4i**

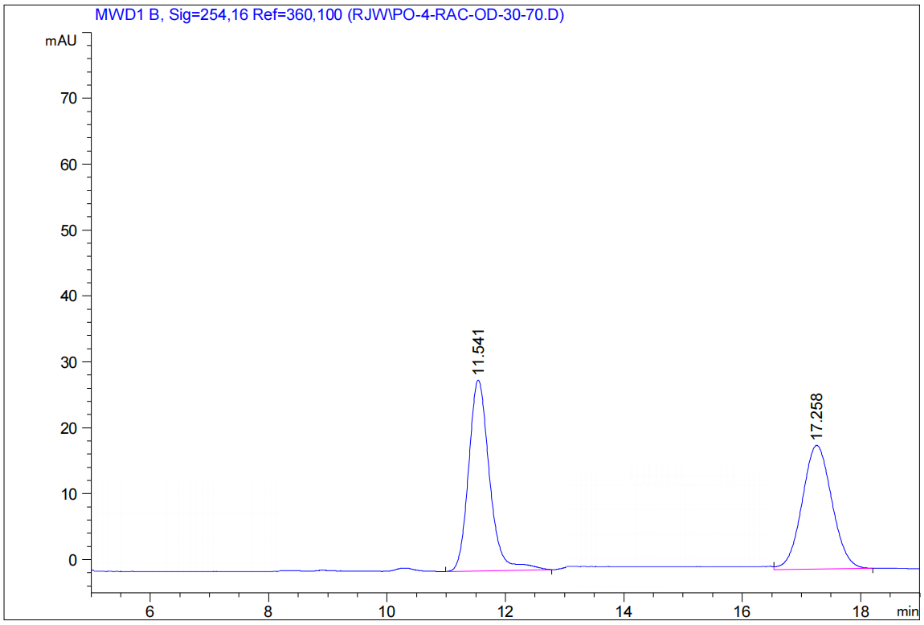

RT [min]	Type	Width [min]	Area	Height	Area%
11.541	BB	0.3739	709.49774	28.95474	51.9369
17.258	VB	0.5398	656.57764	18.78191	48.0631

HPLC chromatogram of enantiopure **4i**

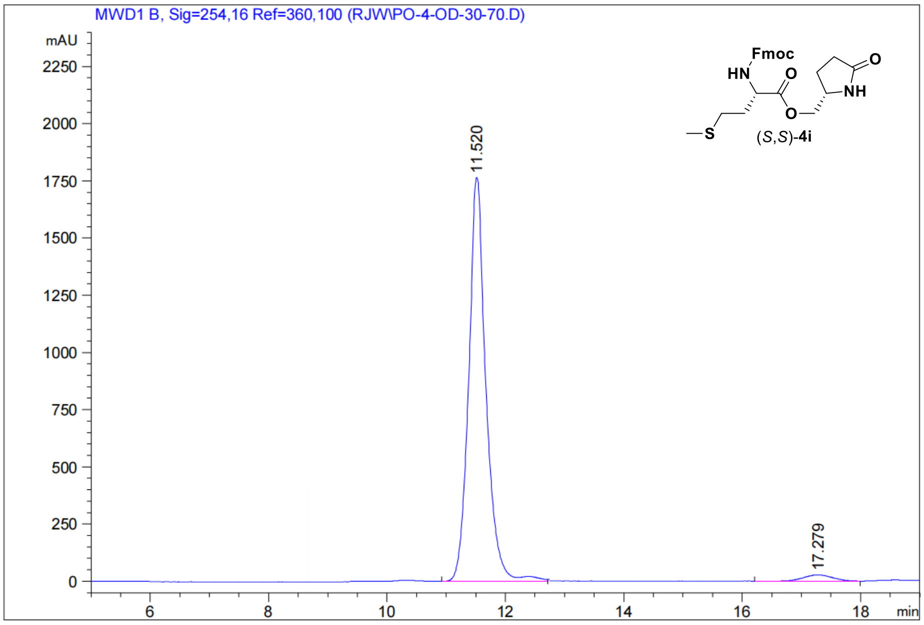

RT [min]	Type	Width [min]	Area	Height	Area%
11.520	MM R	0.3369	3.56853e4	1765.23389	97.0977
17.279	BV	0.5617	1066.66187	28.82915	2.9023

HPLC chromatogram of racemic **4j**

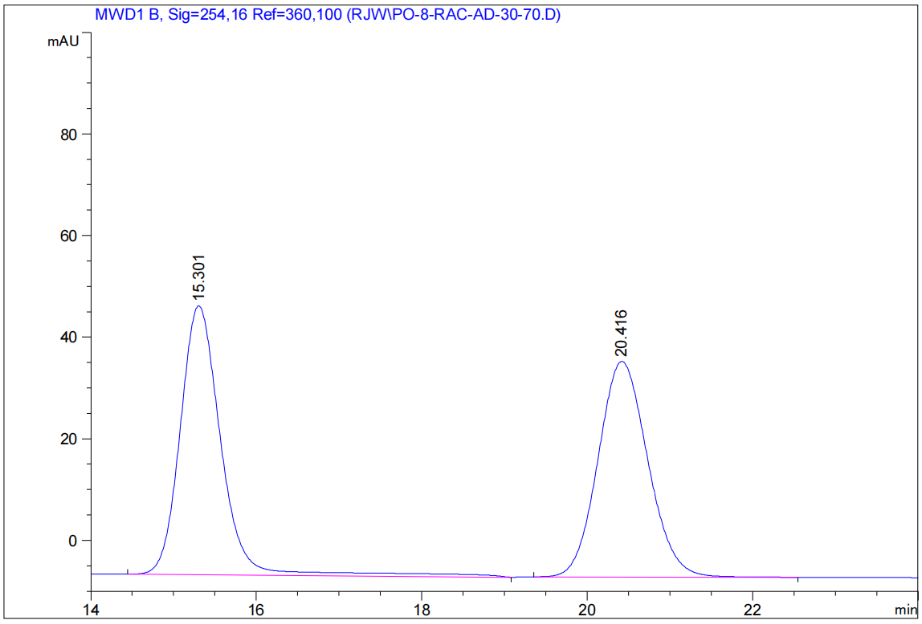

RT [min]	Type	Width [min]	Area	Height	Area%
15.301	BB	0.5296	1840.03198	52.93087	50.9816
20.416	BB	0.6485	1769.17896	42.42698	49.0184

HPLC chromatogram of enantiopure **4j**

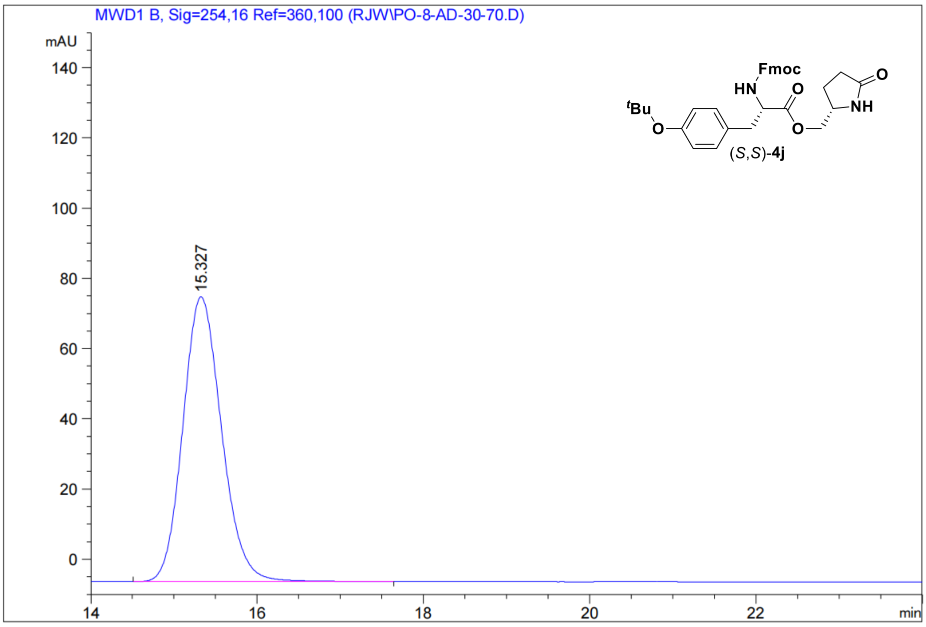

RT [min]	Type	Width [min]	Area	Height	Area%
15.327	BB	0.5014	2623.87988	81.11724	100.0000

HPLC chromatogram of racemic **4k**

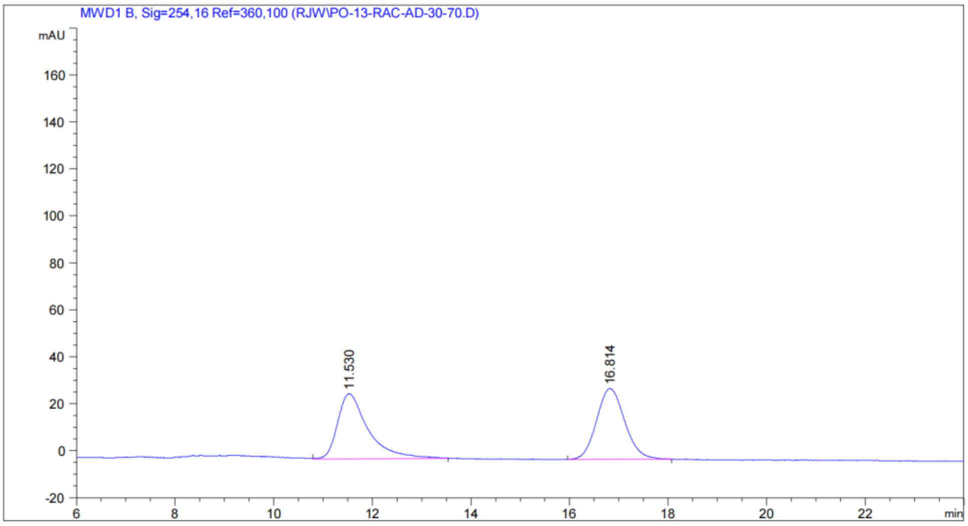

RT [min]	Type	Width [min]	Area	Height	Area%
11.530	MM R	0.7214	1203.14801	27.79632	49.9983
16.814	BB	0.4821	1203.23020	30.24980	50.0017

HPLC chromatogram of enantiopure **4k**

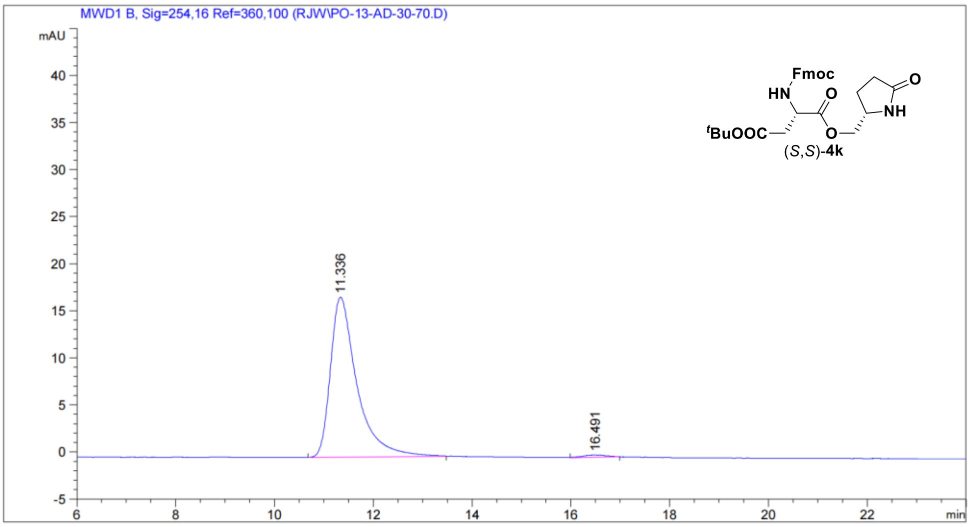

RT [min]	Type	Width [min]	Area	Height	Area%
11.336	BB	0.5490	633.47815	16.99153	98.7552
16.491	MM R	0.5825	7.98466	2.28479e-1	1.2448

HPLC chromatogram of racemic **4l**

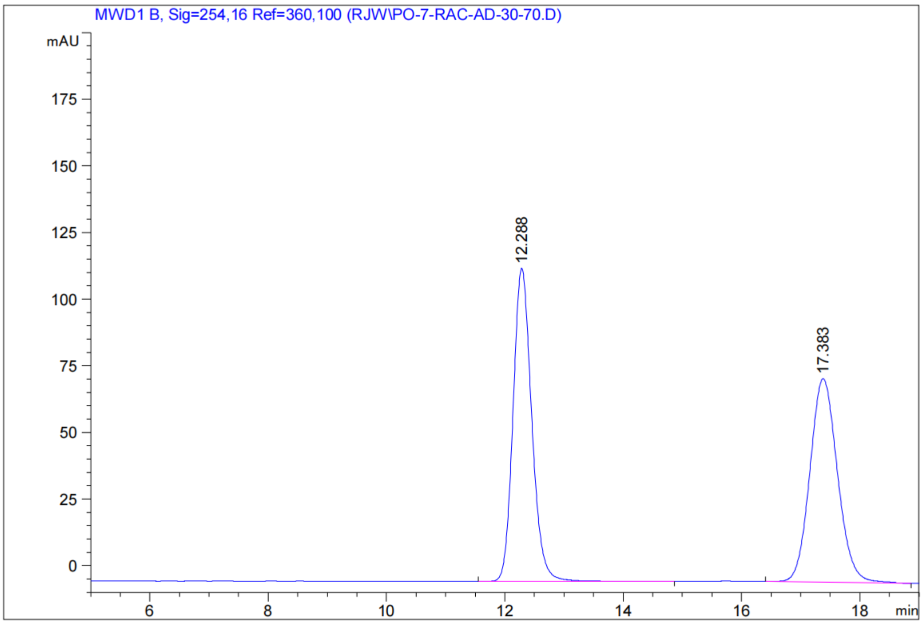

RT [min]	Type	Width [min]	Area	Height	Area%
12.288	BB	0.3378	2576.90015	117.52402	51.0189
17.383	BB	0.5000	2473.97266	76.36546	48.9811

HPLC chromatogram of enantiopure **4l**

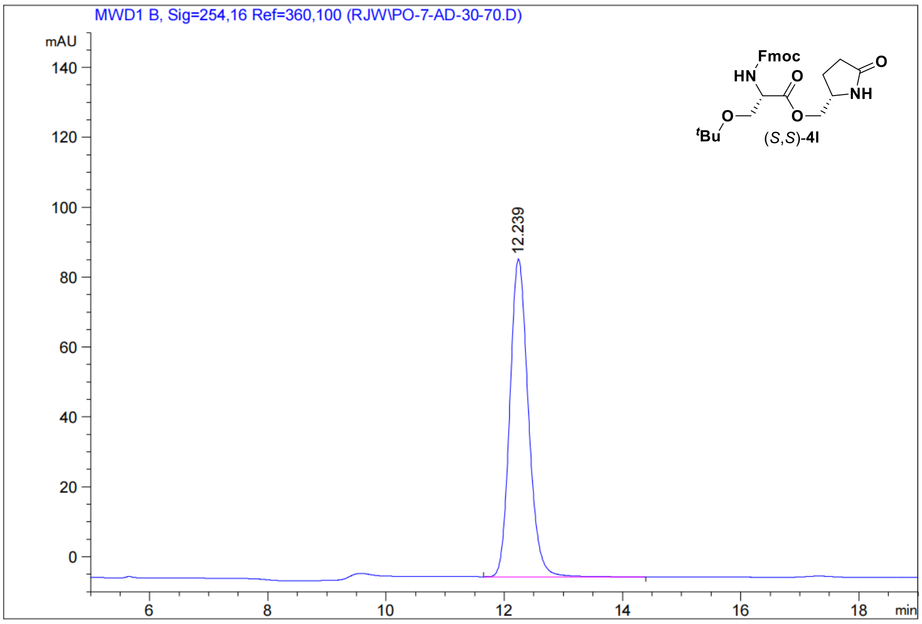

RT [min]	Type	Width [min]	Area	Height	Area%
12.239	BB	0.3353	1973.74536	90.92336	100.0000

HPLC chromatogram of racemic **4m**

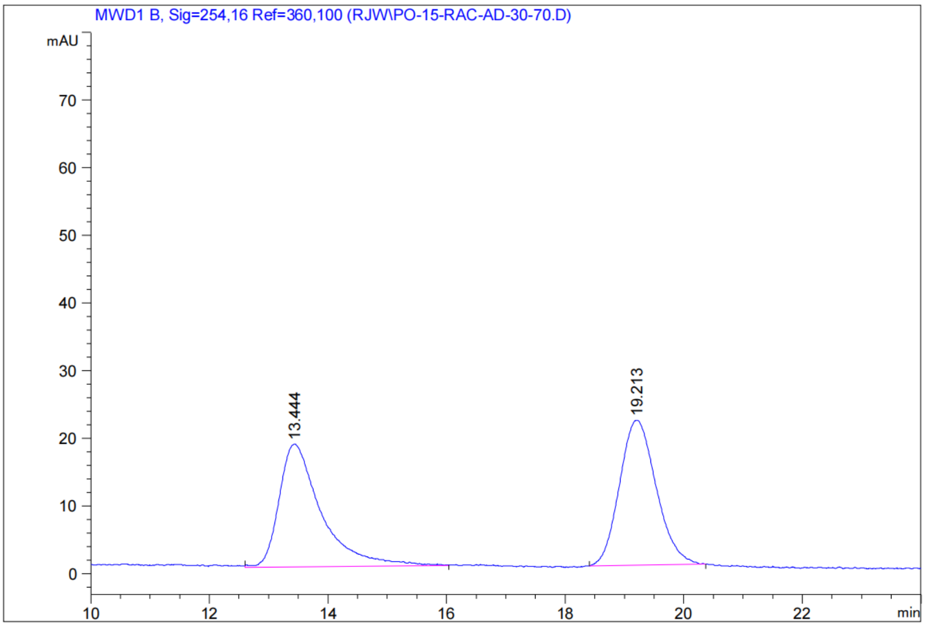

RT [min]	Type	Width [min]	Area	Height	Area%
13.444	MM R	0.8208	894.05960	18.15365	49.2829
19.213	BB	0.5507	920.07871	21.43746	50.7171

HPLC chromatogram of enantiopure **4m**

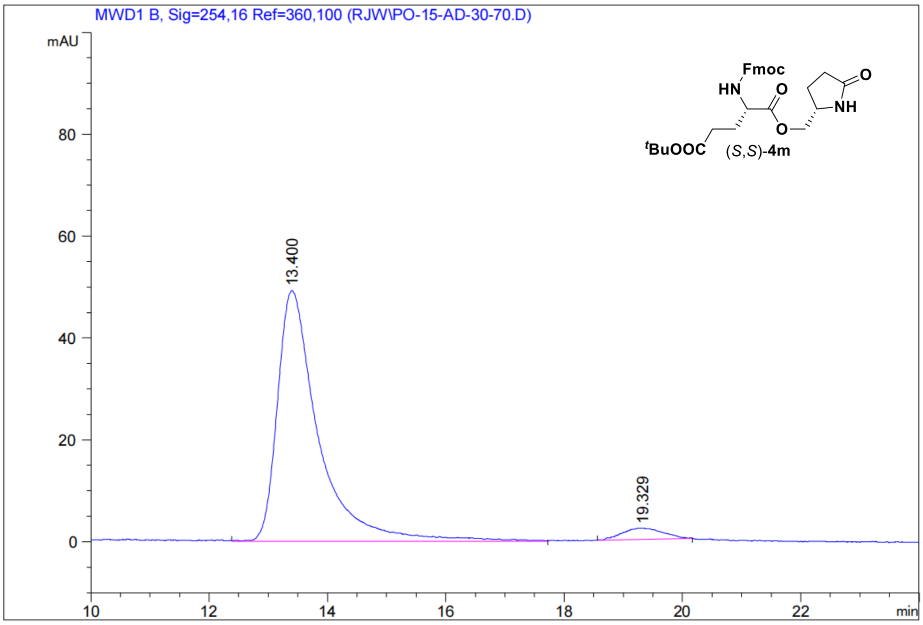

RT [min]	Type	Width [min]	Area	Height	Area%
13.400	MM R	0.8033	2371.93424	49.21433	95.7596
19.329	MM R	0.7897	105.03404	2.21671	4.2404

HPLC chromatogram of racemic **4n**

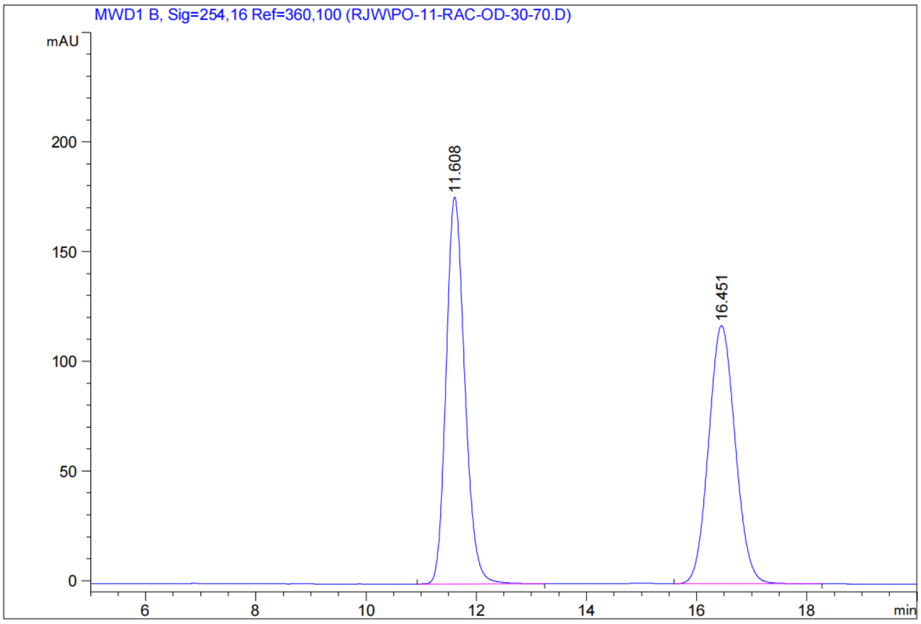

RT [min]	Type	Width [min]	Area	Height	Area%
11.608	BB	0.3622	4145.08398	176.35330	51.2030
16.451	BB	0.5213	3950.30566	117.77408	48.7970

HPLC chromatogram of enantiopure **4n**

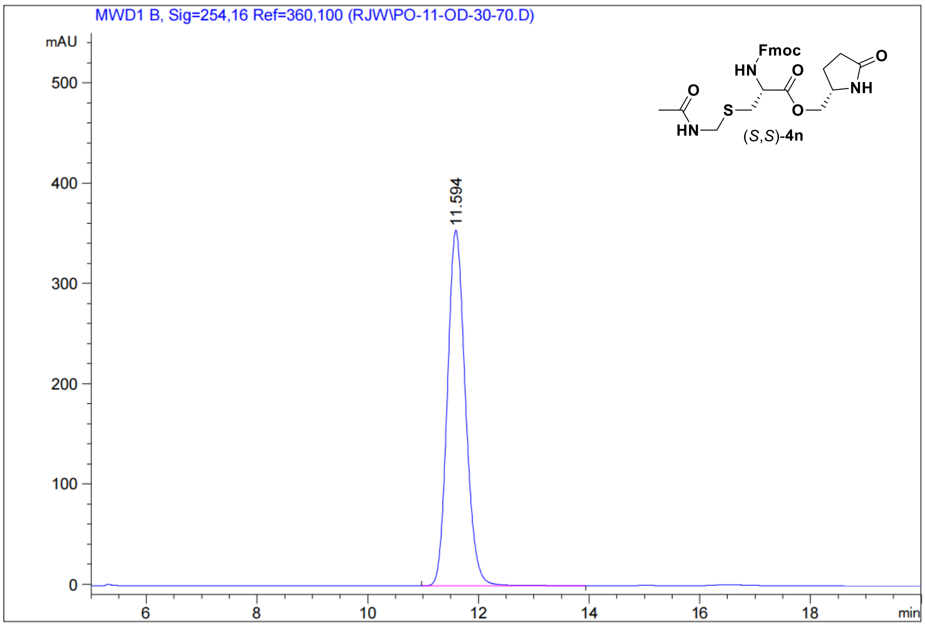

RT [min]	Type	Width [min]	Area	Height	Area%
11.594	BB	0.3493	8000.66846	354.55173	100.0000

HPLC chromatogram of racemic **4o**

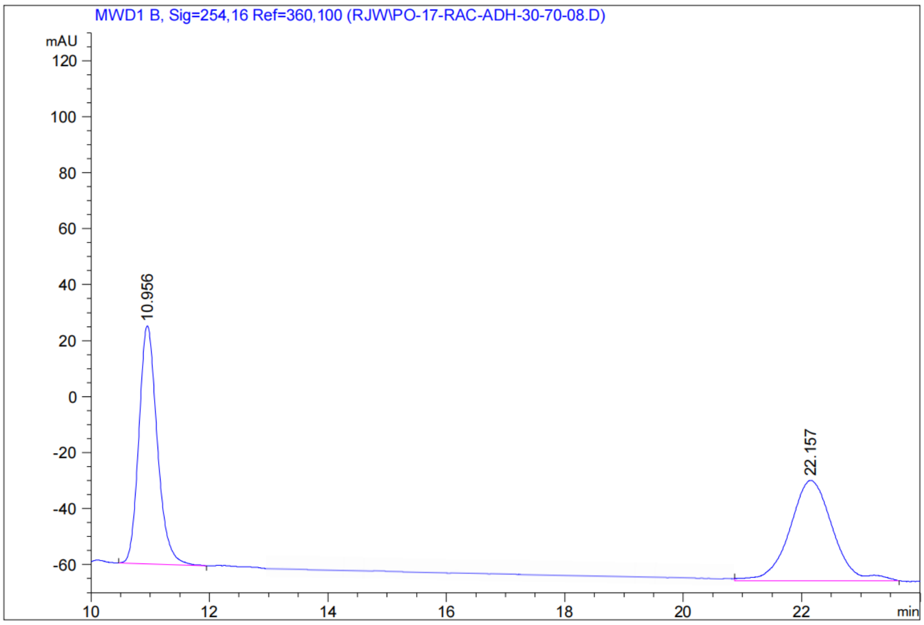

RT [min]	Type	Width [min]	Area	Height	Area%
10.956	BB	0.3333	1832.13526	85.07425	50.3956
22.157	MM R	0.8379	1803.36856	35.86891	49.6044

HPLC chromatogram of enantiopure **4o**

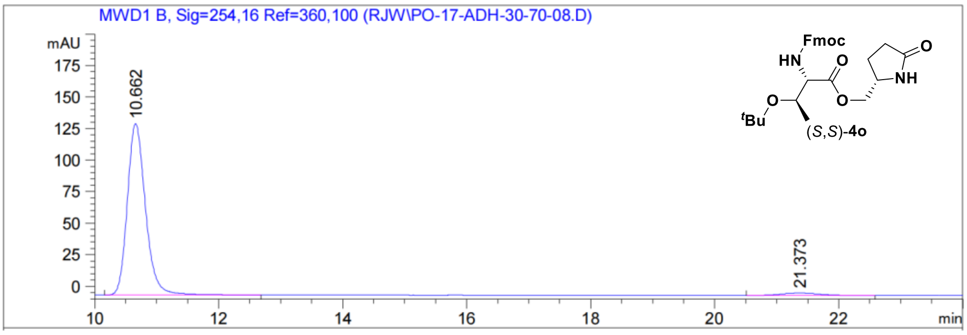

RT [min]	Type	Width [min]	Area	Height	Area%
10.662	BB	0.3112	2762.86499	135.88600	96.8968
21.373	BB	0.5371	88.48228	1.94376	3.1032

HPLC chromatogram of racemic **4p**

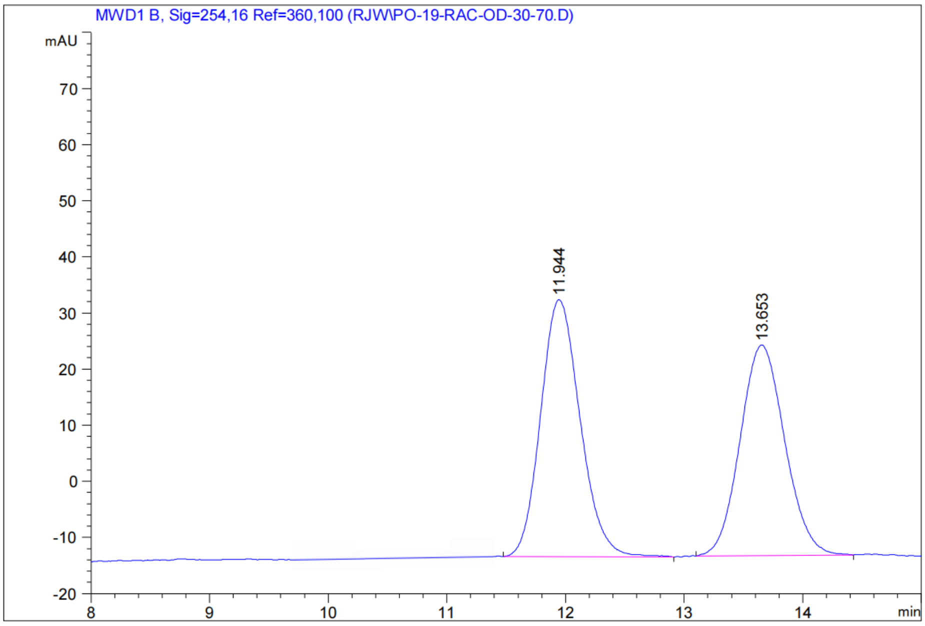

RT [min]	Type	Width [min]	Area	Height	Area%
11.944	BB	0.3602	1060.46427	45.79451	51.9699
13.653	BB	0.4087	980.07204	37.53128	48.0301

HPLC chromatogram of enantiopure **4p**

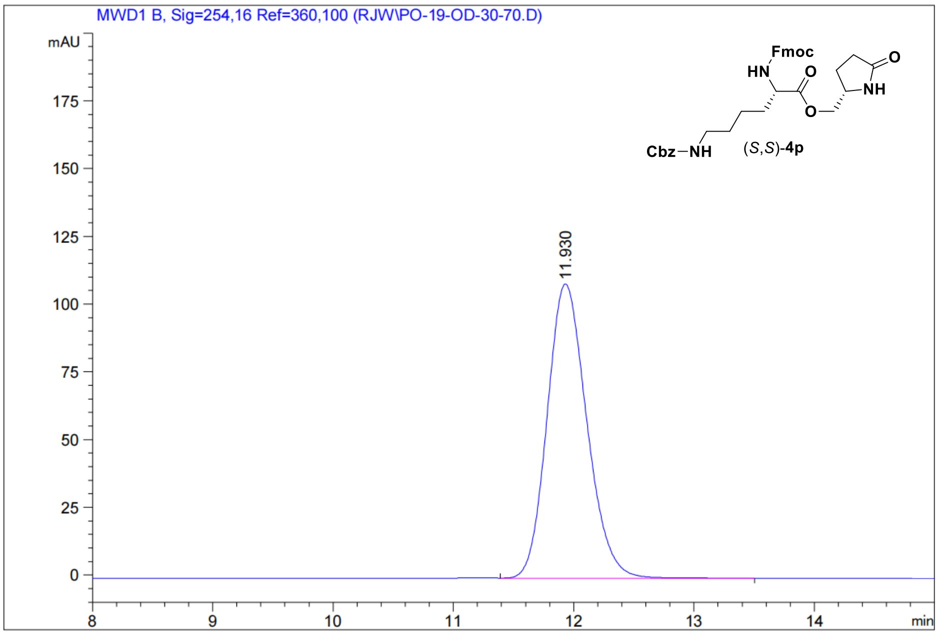

RT [min]	Type	Width [min]	Area	Height	Area%
11.930	VB	0.3553	2490.92261	108.70897	100.0000

HPLC chromatogram of racemic **4q**

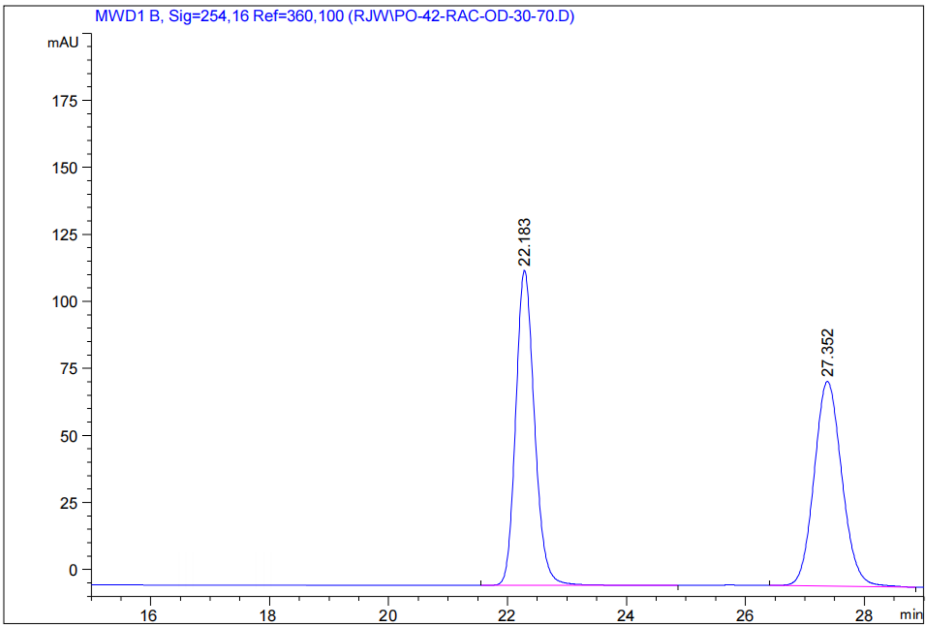

RT [min]	Type	Width [min]	Area	Height	Area%
22.183	BB	0.3359	2572.00029	118.56442	50.9516
27.352	BB	0.5000	2475.93226	77.35526	49.0484

HPLC chromatogram of enantiopure **4q**

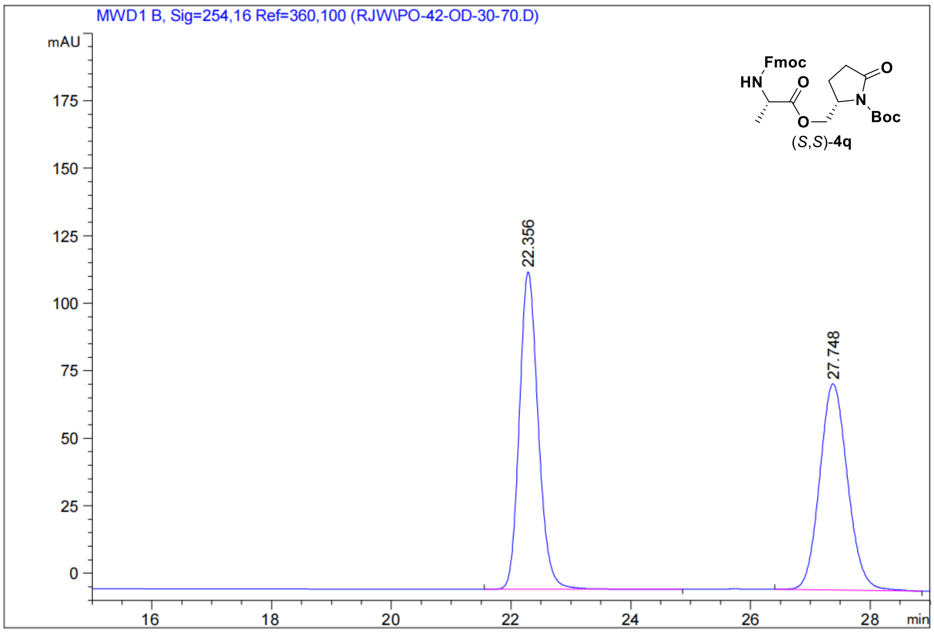

RT [min]	Type	Width [min]	Area	Height	Area%
22.356	BB	0.3361	2582.08020	119.09440	51.0993
27.748	BB	0.5014	2470.98206	77.55521	48.9007

HPLC chromatogram of racemic **4r**

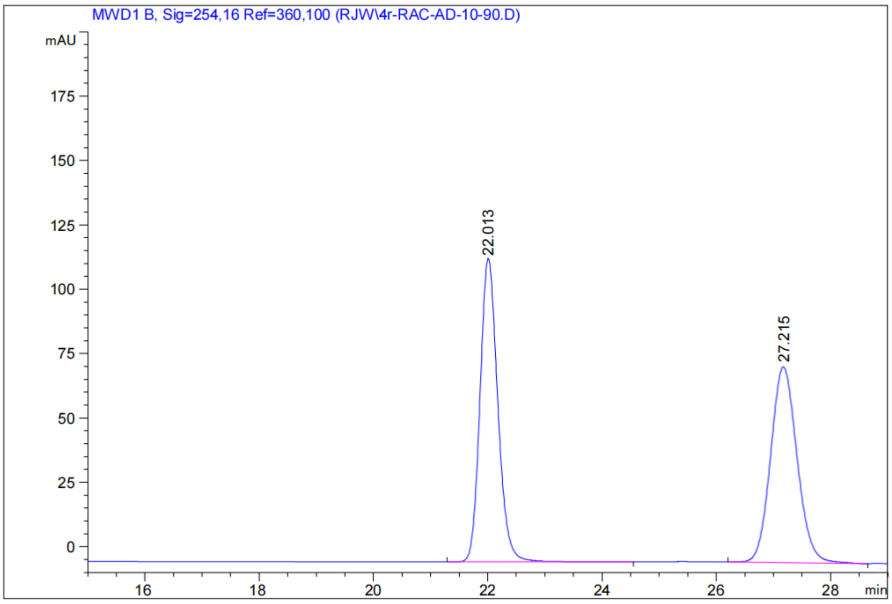

RT [min]	Type	Width [min]	Area	Height	Area%
22.013	BB	0.3359	2572.00109	119.56346	50.2543
27.215	BB	0.5000	2545.97222	76.36522	49.7457

HPLC chromatogram of enantiopure **4r**

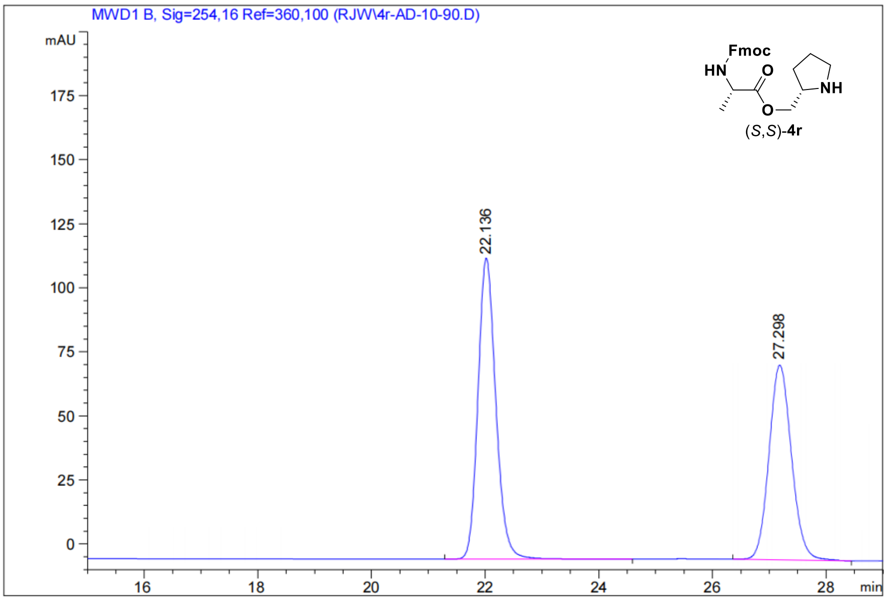

RT [min]	Type	Width [min]	Area	Height	Area%
22.136	BB	0.3361	2592.28017	117.29442	55.0673
27.298	BB	0.4214	2115.19626	78.56541	44.9327

HPLC chromatogram of racemic (*R*,*R*)-**4a**

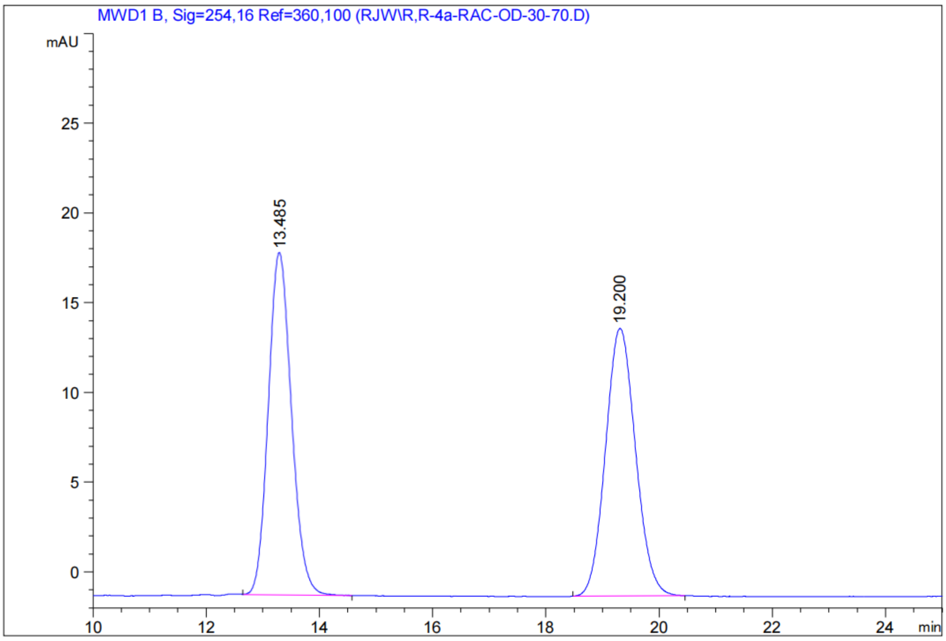

RT [min]	Type	Width [min]	Area	Height	Area%
13.485	BB	0.4139	551.22230	19.12352	50.0556
19.200	BB	0.5698	549.99866	15.01223	49.9444

HPLC chromatogram of enantiopure (*R*,*R*)-**4a**

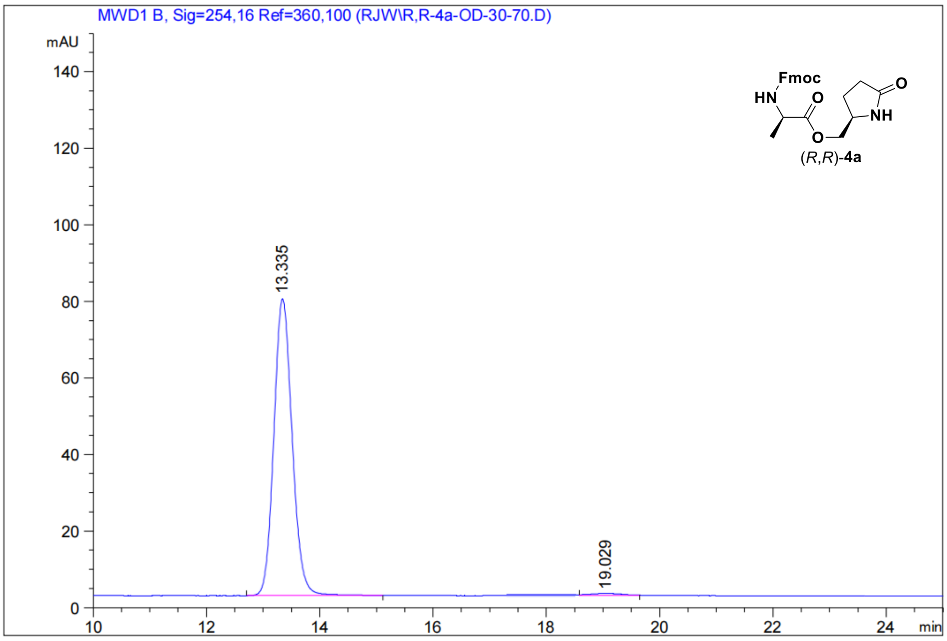

RT [min]	Type	Width [min]	Area	Height	Area%
13.335	BB	0.3687	1846.11453	77.88357	98.9859
19.029	MM R	0.6327	18.91310	4.69787e-1	1.0141

HPLC chromatogram of racemic **6a**

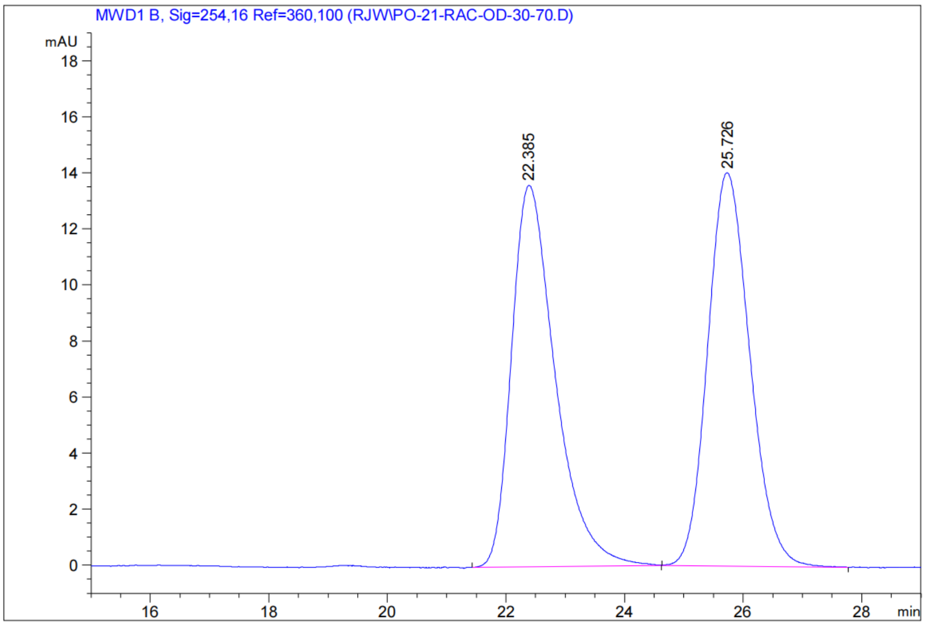

RT [min]	Type	Width [min]	Area	Height	Area%
22.385	BB	0.7214	696.97760	13.61076	50.1845
25.726	BB	0.7453	691.85229	14.03062	49.8155

HPLC chromatogram of enantiopure **6a**

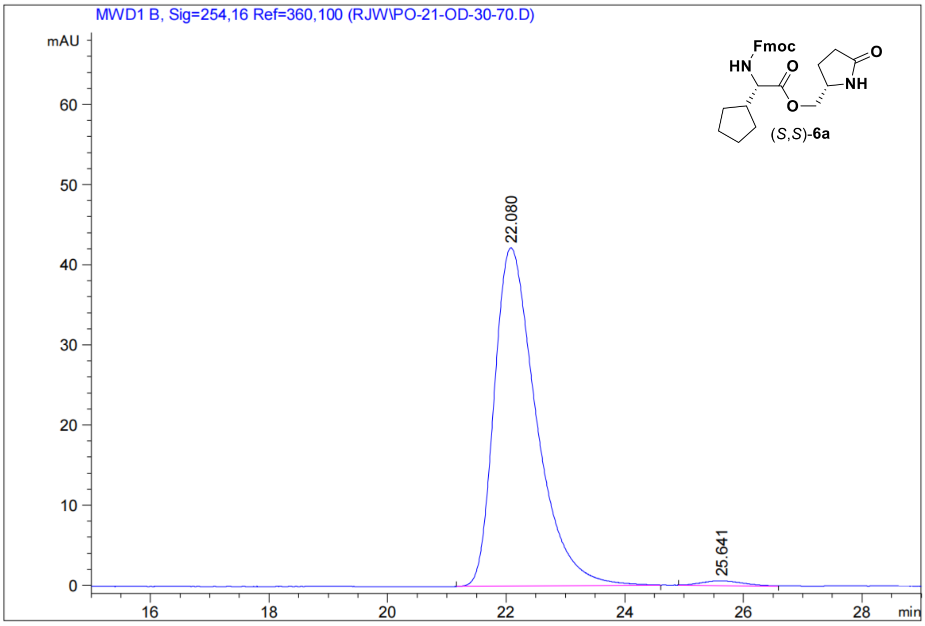

RT [min]	Type	Width [min]	Area	Height	Area%
22.080	BB	0.7402	2077.50952	42.21020	98.5650
25.641	MM R	0.8253	30.24686	6.10843e-1	1.4350

HPLC chromatogram of racemic **6b**

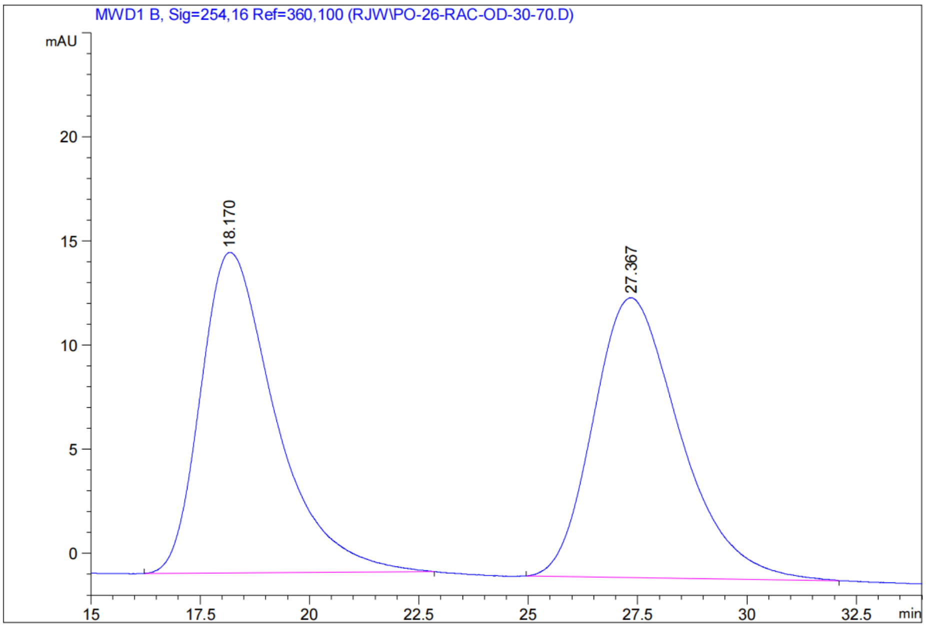

RT [min]	Type	Width [min]	Area	Height	Area%
18.170	BB	1.3999	1827.62756	15.38568	49.6657
27.367	BB	1.6276	1852.23071	13.42492	50.3343

HPLC chromatogram of enantiopure **6b**

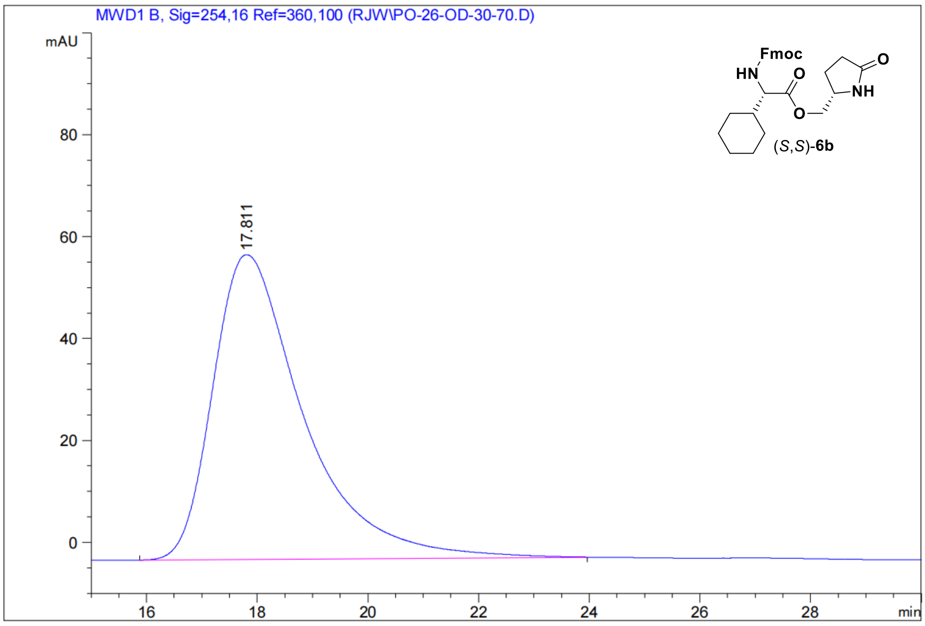

RT [min]	Type	Width [min]	Area	Height	Area%
17.811	BB	1.6300	6817.57422	59.80160	100.0000

HPLC chromatogram of racemic **6c**

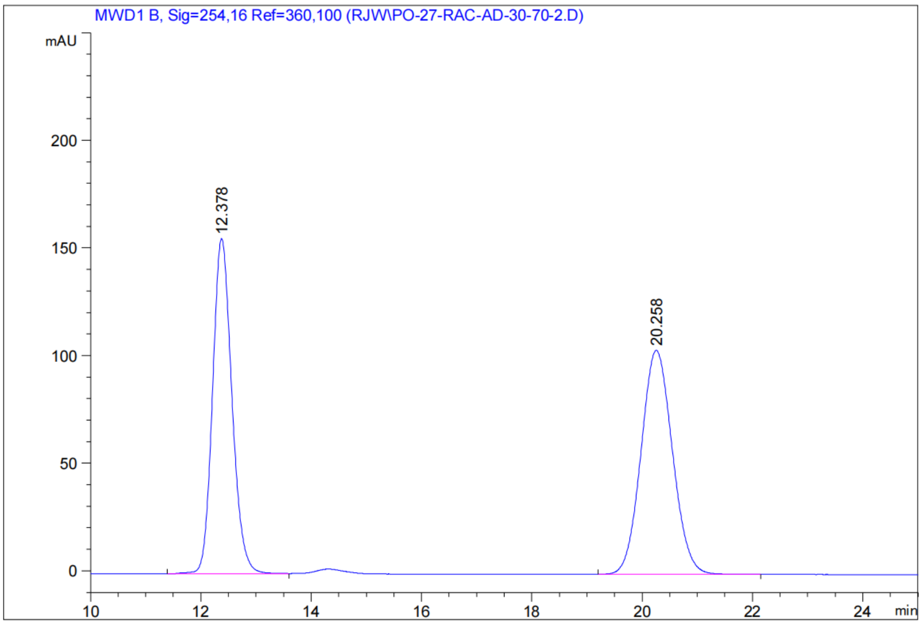

RT [min]	Type	Width [min]	Area	Height	Area%
12.378	BB	0.3760	3789.02148	155.65173	47.9917
20.258	BB	0.6125	4106.14063	104.02364	52.0083

HPLC chromatogram of enantiopure **6c**

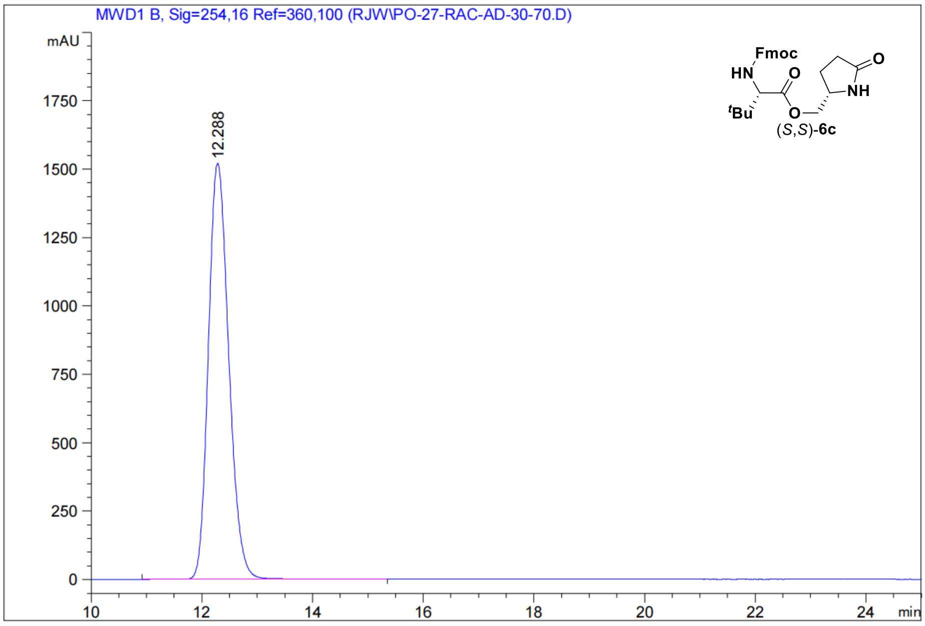

RT [min]	Type	Width [min]	Area	Height	Area%
12.288	BB	0.3960	3.88127e4	1519.51221	100.0000

HPLC chromatogram of racemic **6d**

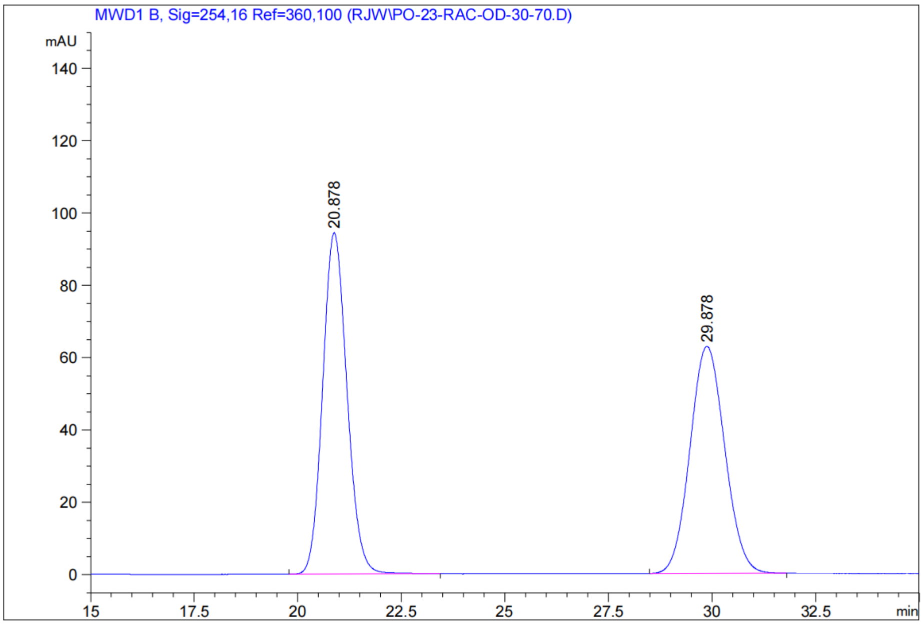

RT [min]	Type	Width [min]	Area	Height	Area%
20.878	BB	0.6361	3851.34155	94.37812	51.2273
29.878	BB	0.8712	3666.79761	62.80106	48.7727

HPLC chromatogram of enantiopure **6d**

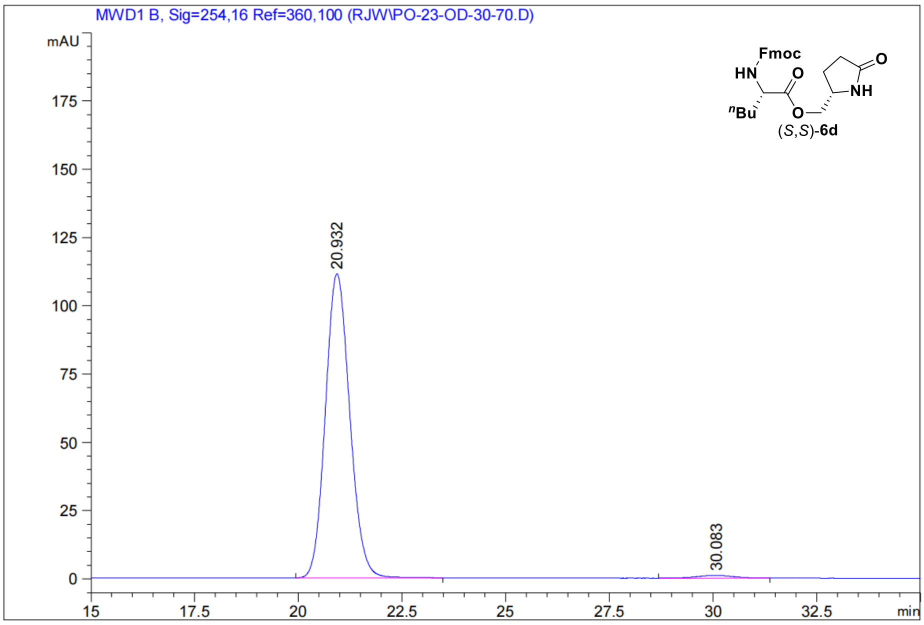

RT [min]	Type	Width [min]	Area	Height	Area%
20.932	BB	0.6327	4547.38184	111.29111	98.5855
30.083	MM R	1.0444	65.24710	1.04119	1.4145

HPLC chromatogram of racemic **6e**

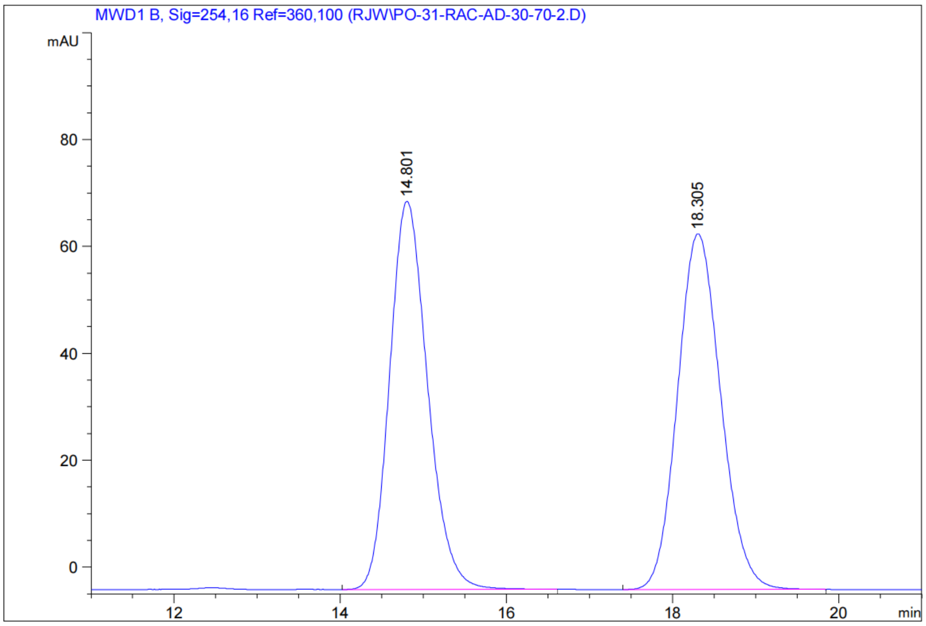

RT [min]	Type	Width [min]	Area	Height	Area%
14.801	BB	0.4883	2305.37891	72.63098	48.9331
18.305	BB	0.5597	2405.90796	66.56560	51.0669

HPLC chromatogram of enantiopure **6e**

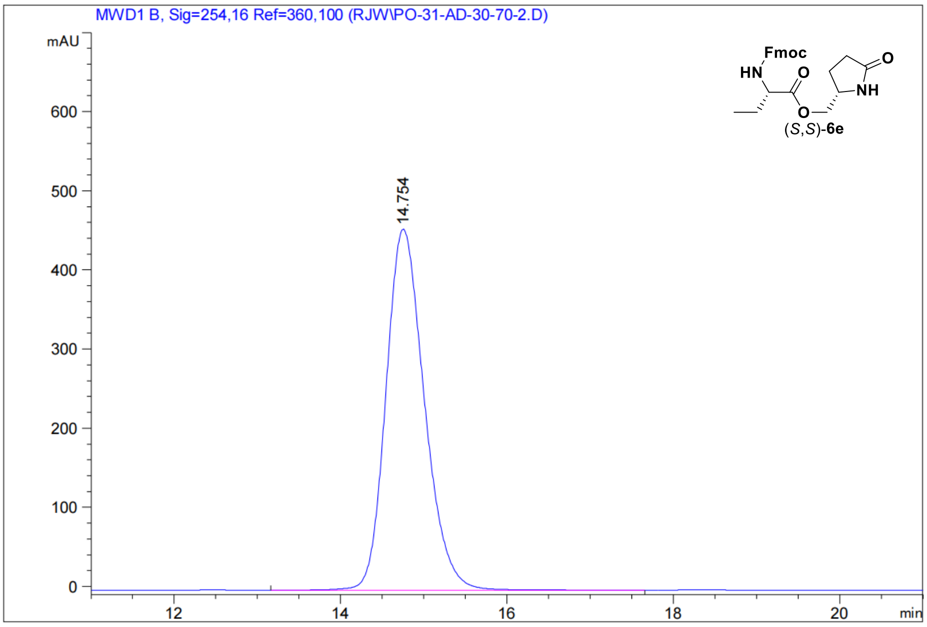

RT [min]	Type	Width [min]	Area	Height	Area%
14.754	BB	0.4919	1.46240e4	456.24854	100.0000

HPLC chromatogram of racemic **6f**

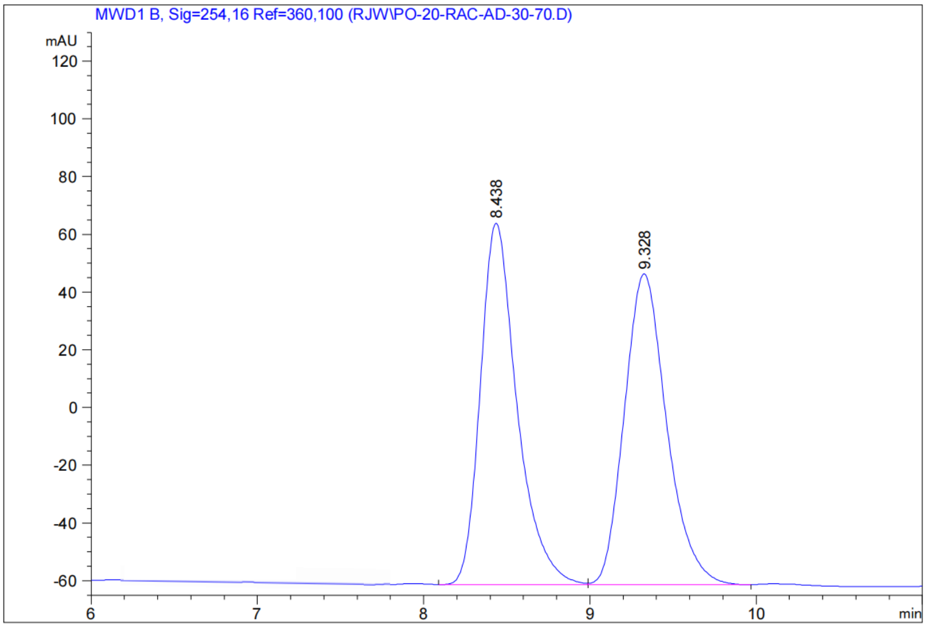

RT [min]	Type	Width [min]	Area	Height	Area%
8.438	BV	0.2309	1920.61974	125.27988	50.1894
9.328	VB	0.2684	1906.12351	107.74803	49.8106

HPLC chromatogram of enantiopure **6f**

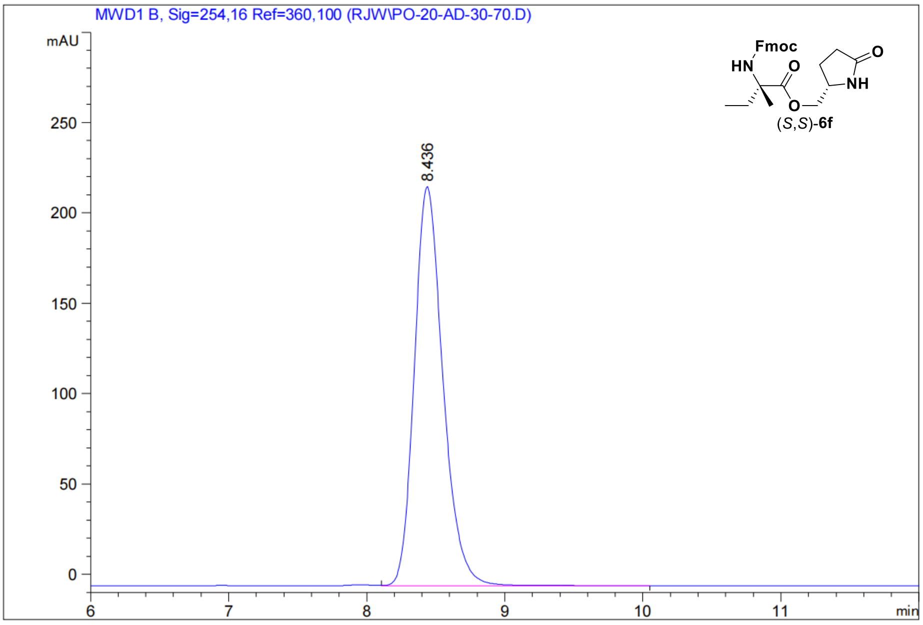

RT [min]	Type	Width [min]	Area	Height	Area%
8.436	VB	0.2204	3191.75269	221.10892	100.0000

HPLC chromatogram of racemic **6g**

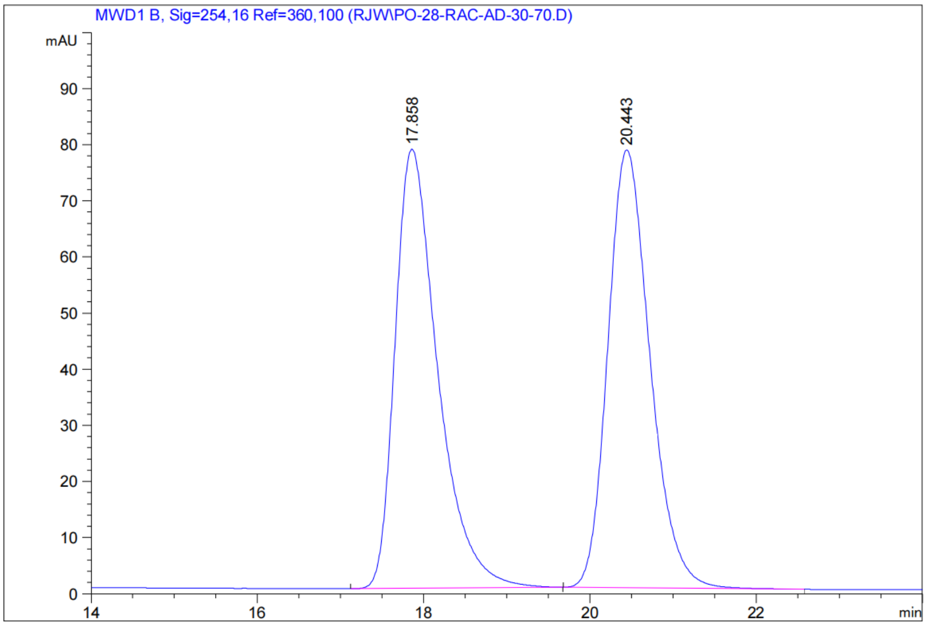

RT [min]	Type	Width [min]	Area	Height	Area%
17.858	BB	0.5421	2790.99170	78.26346	50.3595
20.443	BB	0.5434	2751.14917	78.02217	49.6405

HPLC chromatogram of enantiopure **6g**

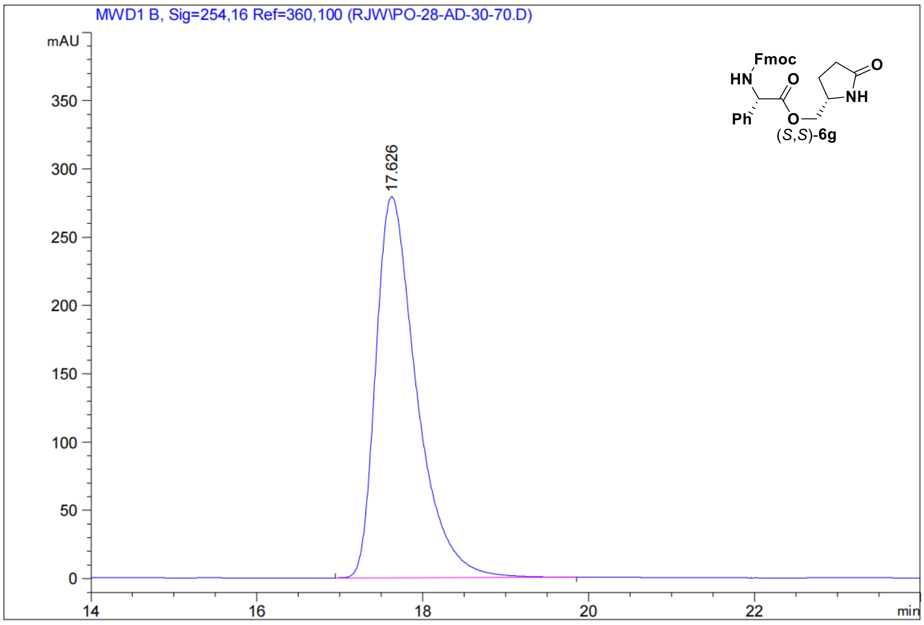

RT [min]	Type	Width [min]	Area	Height	Area%
17.626	BB	0.5144	9531.78613	279.13260	100.0000

HPLC chromatogram of racemic **6h**

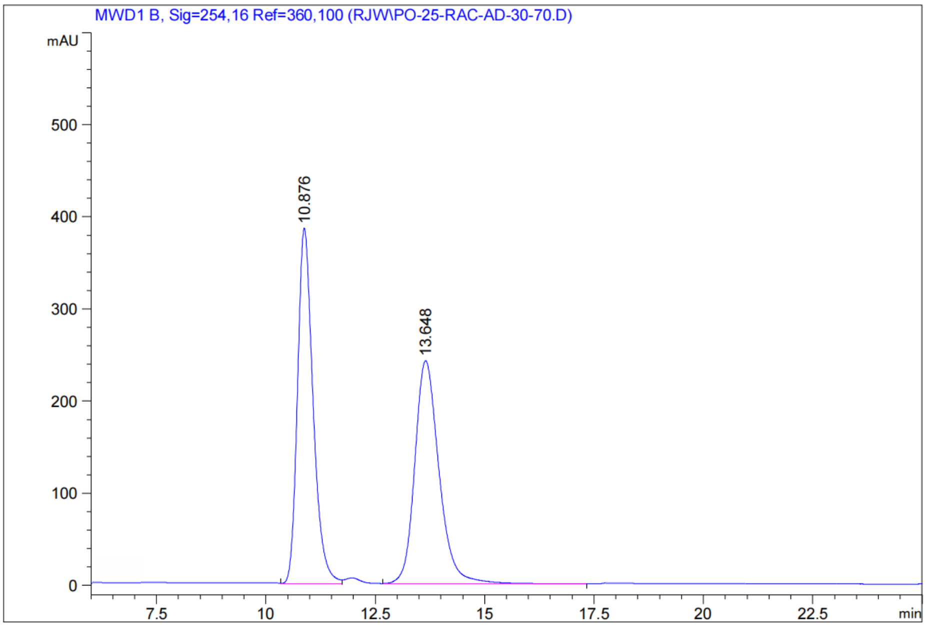

RT [min]	Type	Width [min]	Area	Height	Area%
10.876	VV	0.3768	9553.07520	385.94458	50.4055
13.648	VB	0.5907	9399.38379	242.25034	49.5945

HPLC chromatogram of enantiopure **6h**

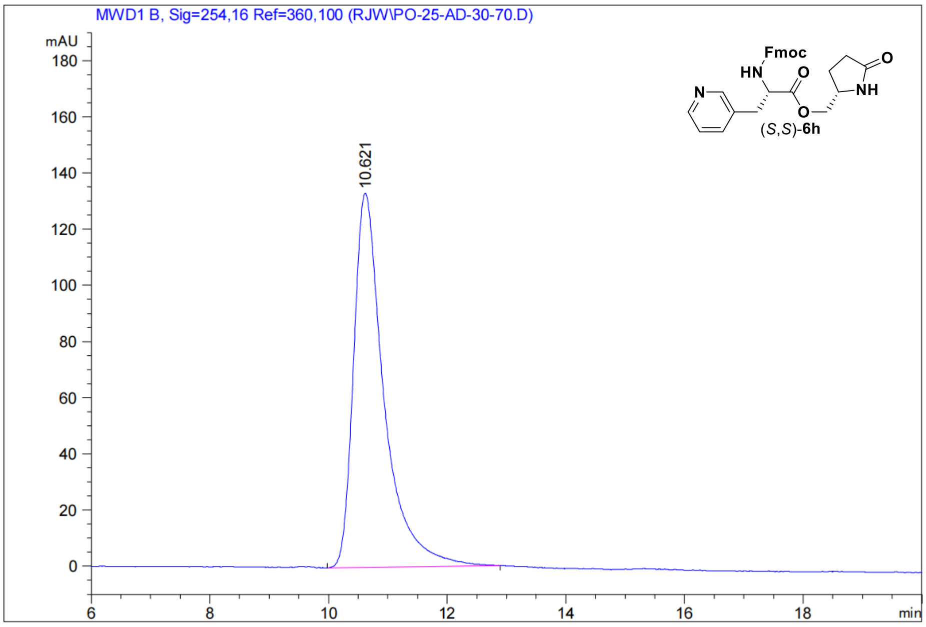

RT [min]	Type	Width [min]	Area	Height	Area%
10.621	BB	0.5296	4744.66437	133.20633	100.0000

HPLC chromatogram of racemic **6i**

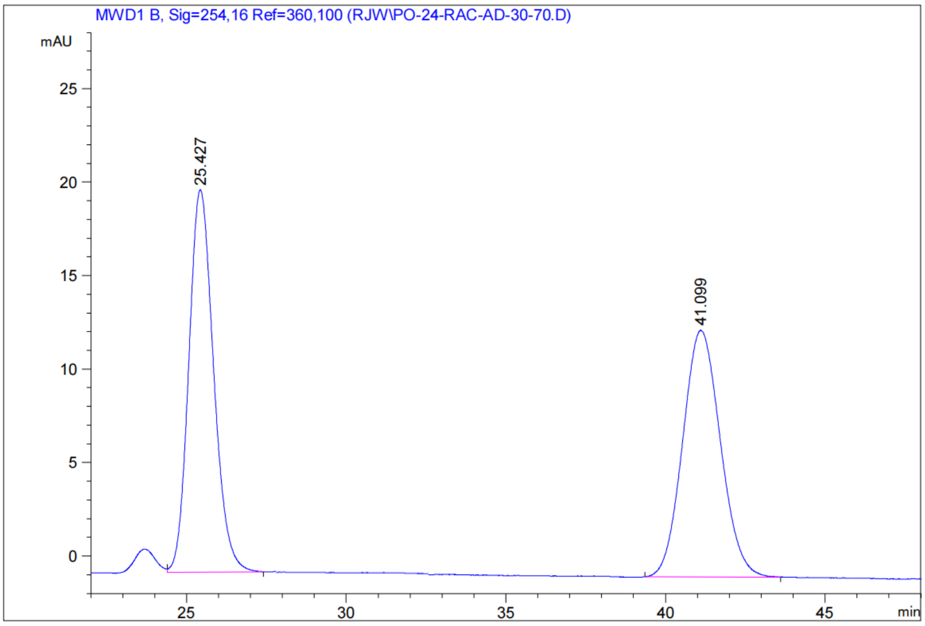

RT [min]	Type	Width [min]	Area	Height	Area%
25.427	VB	0.8288	1103.05164	20.46439	50.4690
41.099	BB	0.9891	1082.55273	13.19529	49.5310

HPLC chromatogram of enantiopure **6i**

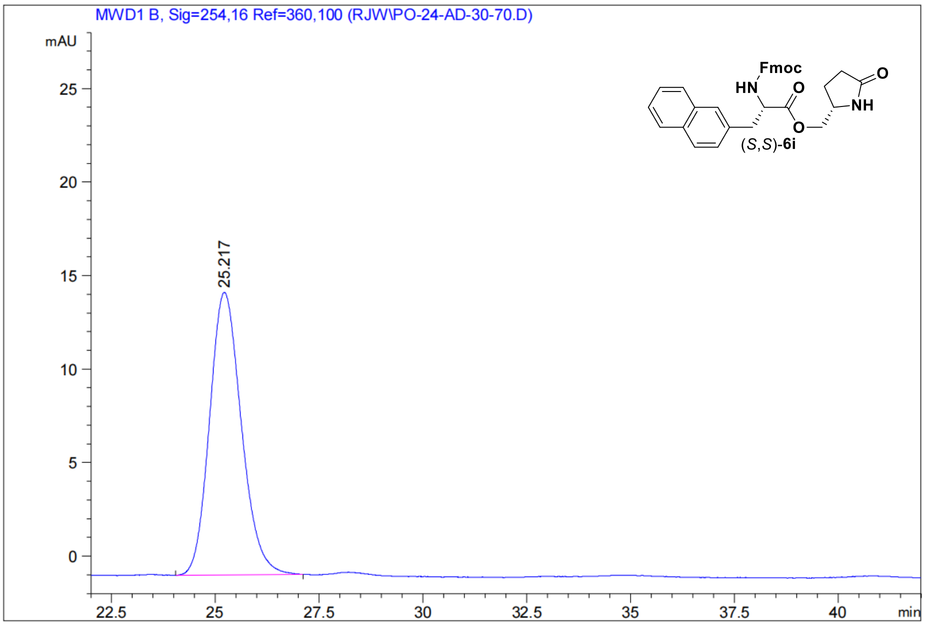

RT [min]	Type	Width [min]	Area	Height	Area%
25.217	BB	0.7815	804.83417	15.10847	100.0000

HPLC chromatogram of racemic **6j**

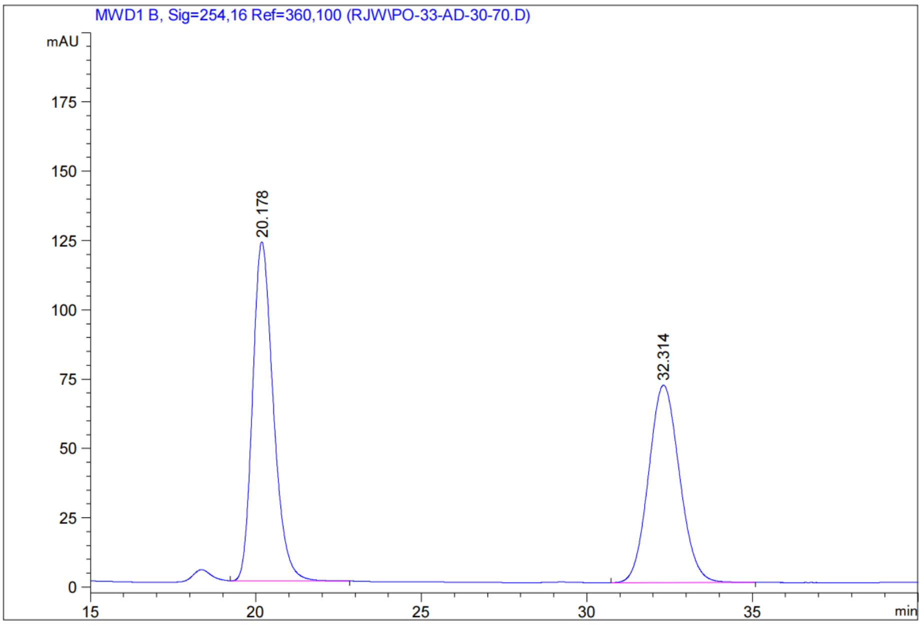

RT [min]	Type	Width [min]	Area	Height	Area%
20.178	BB	0.6649	5313.19238	122.28440	53.4251
32.314	BB	0.9991	4631.92920	71.18931	46.5749

HPLC chromatogram of enantiopure **6j**

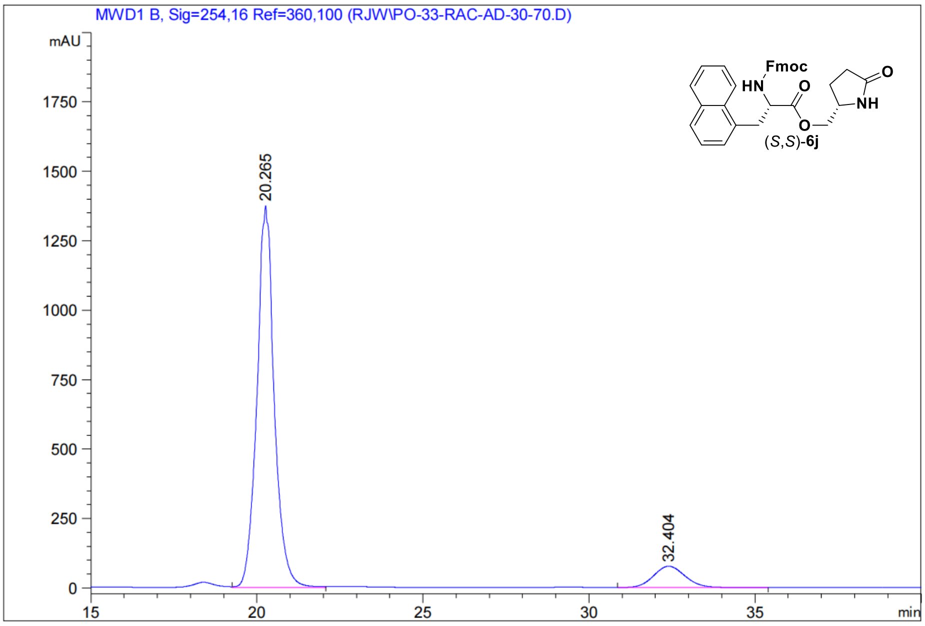

RT [min]	Type	Width [min]	Area	Height	Area%
20.265	VV	0.4386	4.97593e4	1373.80957	90.8721
32.404	BB	1.0015	4998.21094	76.37936	9.1279

HPLC chromatogram of racemic **6k**

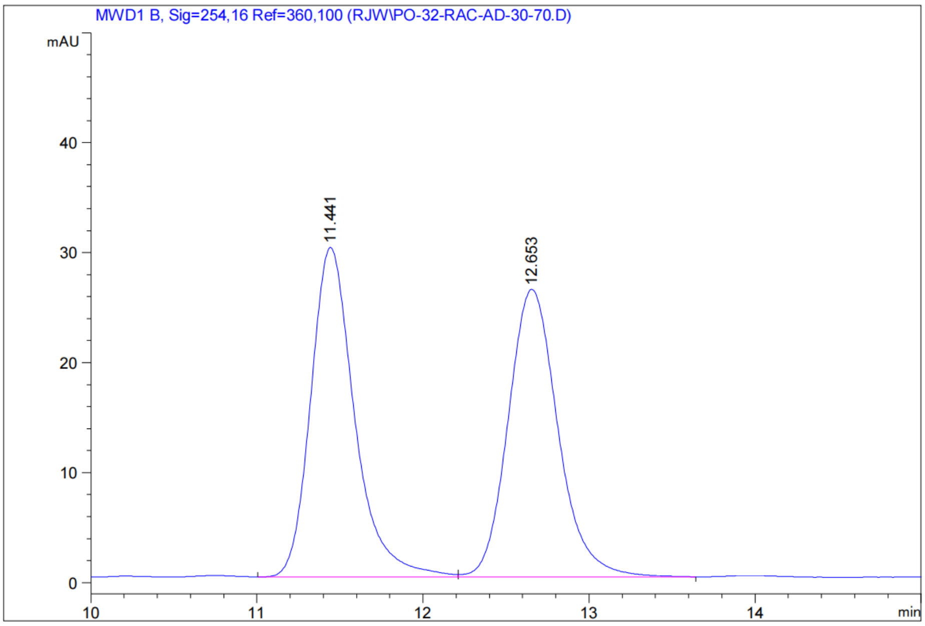

RT [min]	Type	Width [min]	Area	Height	Area%
11.441	BV	0.2798	548.95941	29.96011	50.2029
12.653	VB	0.3230	544.52252	26.14178	49.7971

HPLC chromatogram of enantiopure **6k**

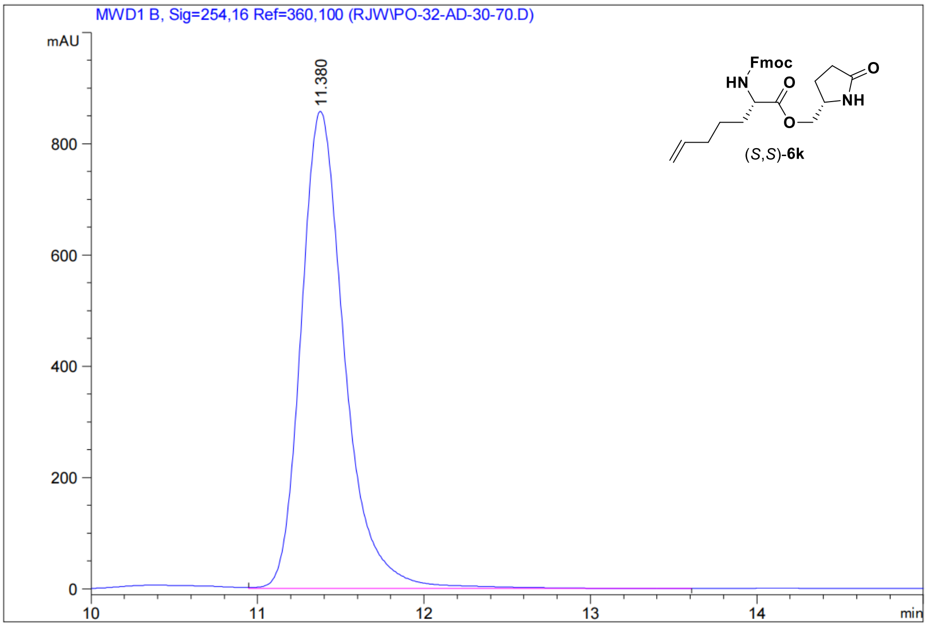

RT [min]	Type	Width [min]	Area	Height	Area%
11.380	VB	0.2762	1.55941e4	857.34216	100.0000

HPLC chromatogram of racemic **6l**

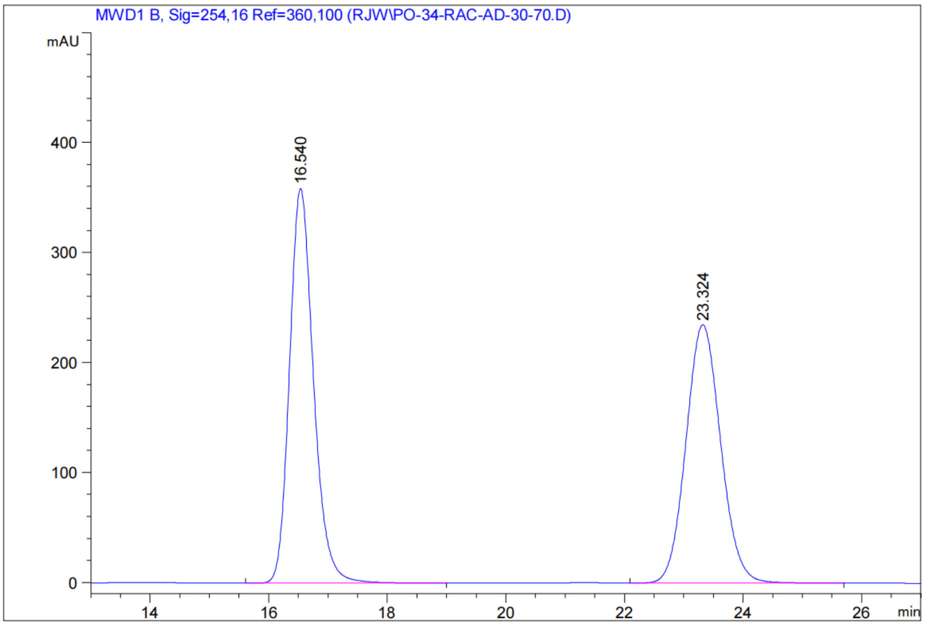

RT [min]	Type	Width [min]	Area	Height	Area%
16.540	BB	0.4321	1.00894e4	358.78632	51.3083
23.324	BB	0.6335	9574.87012	234.90974	48.6917

HPLC chromatogram of enantiopure **6l**

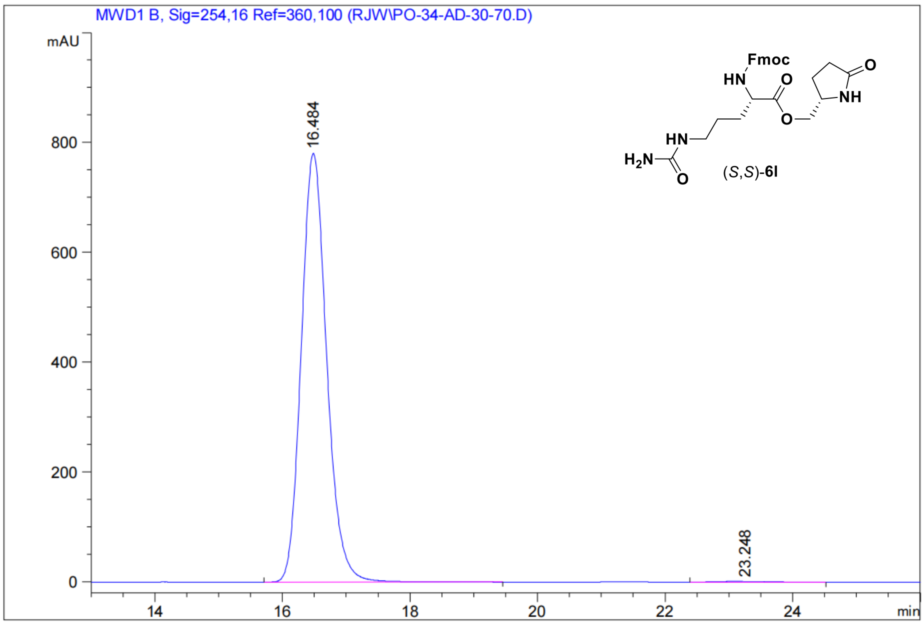

RT [min]	Type	Width [min]	Area	Height	Area%
16.484	BB	0.4285	2.15944e4	781.09296	99.6522
23.248	BB	0.4999	75.37556	1.84013	0.3478

HPLC chromatogram of racemic **6m**

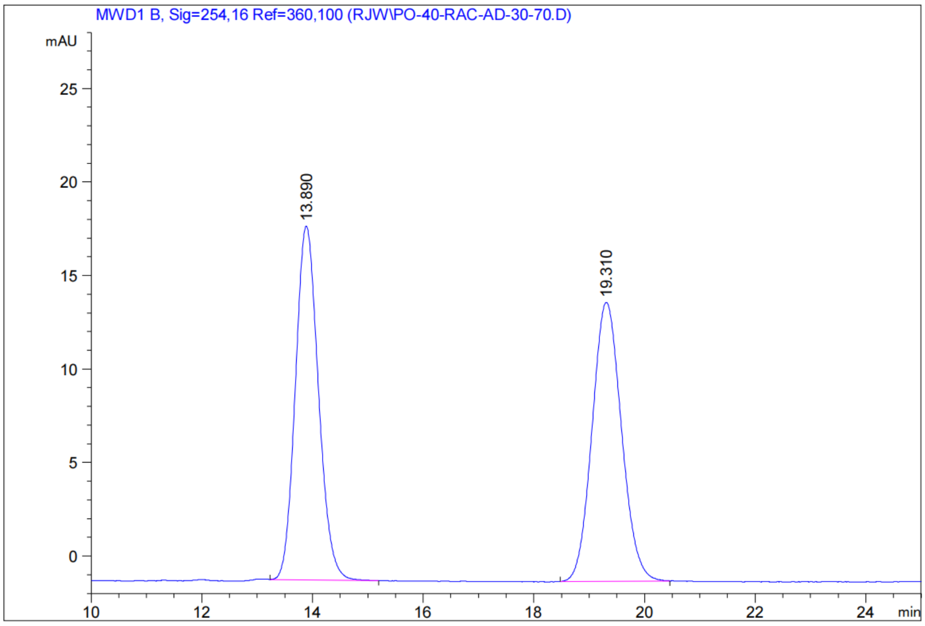

RT [min]	Type	Width [min]	Area	Height	Area%
13.890	BB	0.4453	547.21411	18.92940	49.8915
19.310	BB	0.5779	549.59375	14.91663	50.1085

HPLC chromatogram of enantiopure **6m**

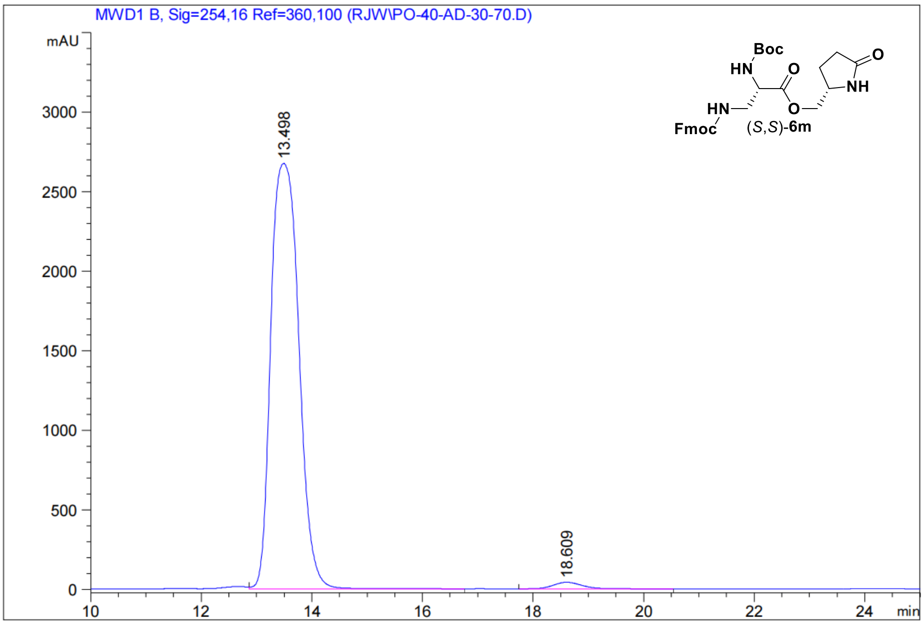

RT [min]	Type	Width [min]	Area	Height	Area%
13.498	VB	0.5654	9.37885e4	2672.81958	98.4532
18.609	BB	0.5566	1473.50427	40.86944	1.5468

HPLC chromatogram of racemic **6n**

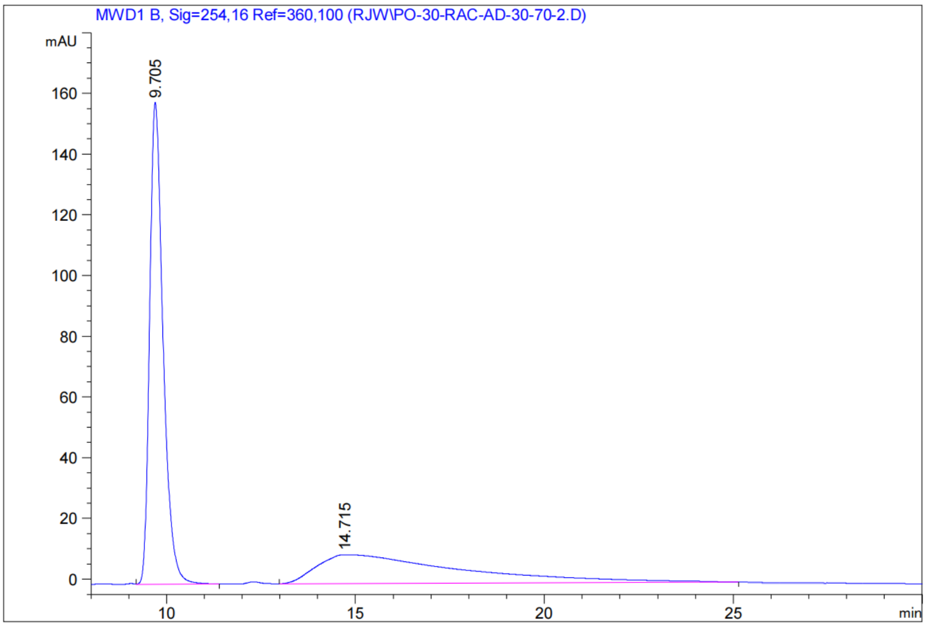

RT [min]	Type	Width [min]	Area	Height	Area%
9.705	VB	0.3763	3922.13770	158.73055	52.1083
14.715	BB	3.0922	3604.75830	9.49953	47.8917

HPLC chromatogram of enantiopure **6n**

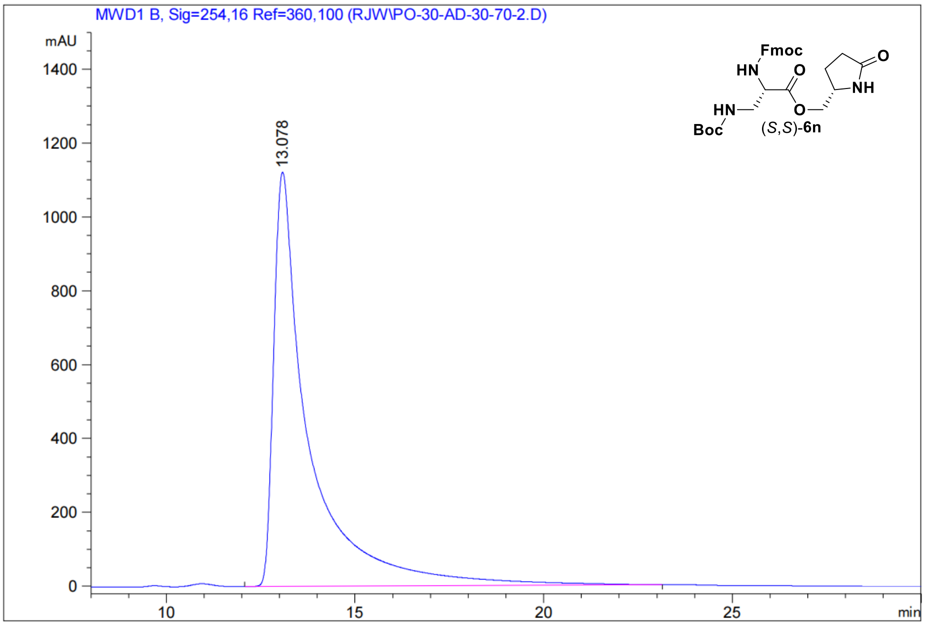

RT [min]	Type	Width [min]	Area	Height	Area%
13.078	BB	0.8999	7.38577e4	1122.10461	100.0000

HPLC chromatogram of racemic **6o**

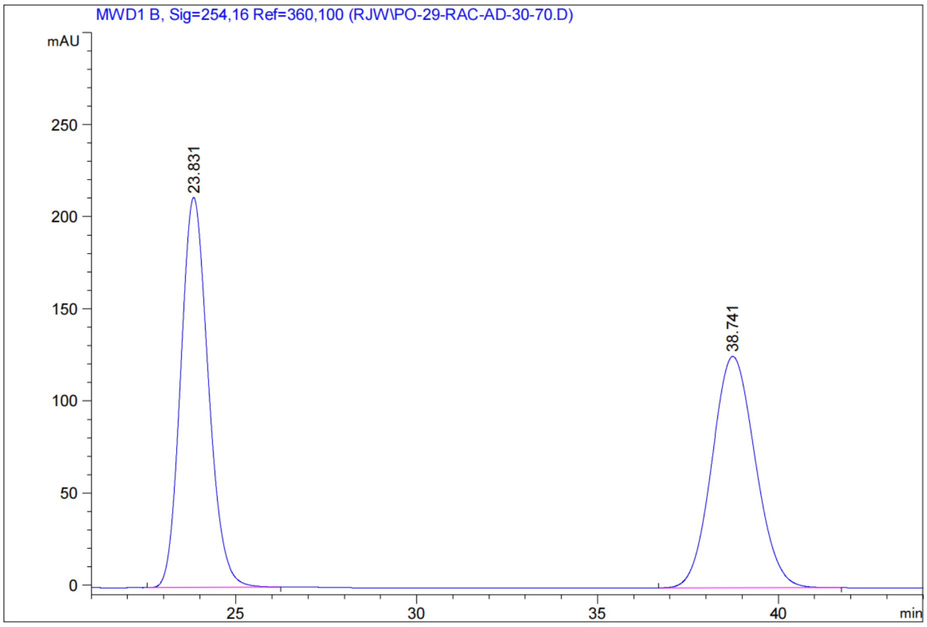

RT [min]	Type	Width [min]	Area	Height	Area%
23.831	BB	0.8185	1.11821e4	211.61855	51.7203
38.741	BB	1.2917	1.04382e4	125.57852	48.2797

HPLC chromatogram of enantiopure **6o**

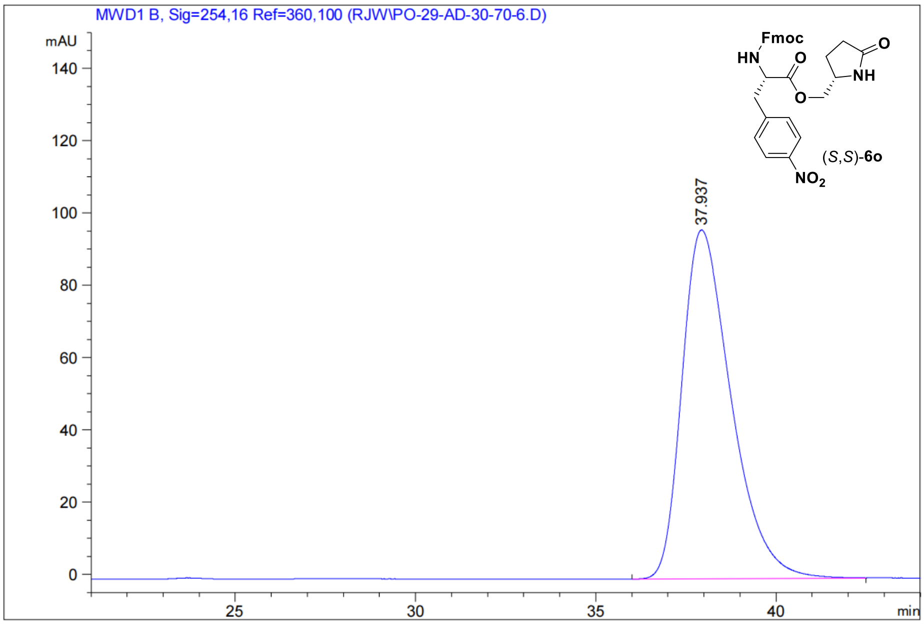

RT [min]	Type	Width [min]	Area	Height	Area%
37.937	BB	1.4112	9163.14746	96.54932	100.0000

HPLC chromatogram of racemic **6p**

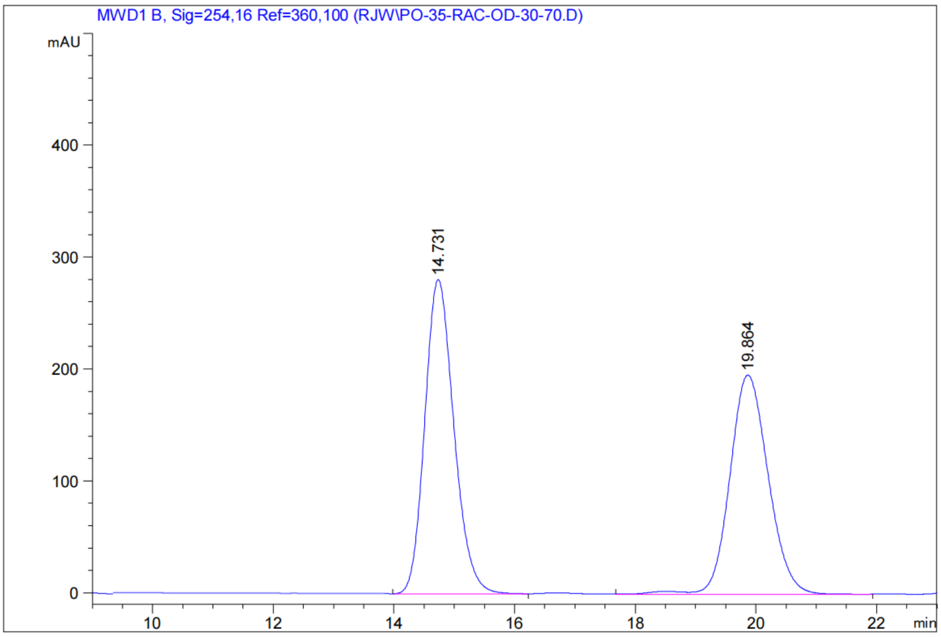

RT [min]	Type	Width [min]	Area	Height	Area%
14.731	BB	0.5171	9408.16699	280.66321	52.0377
19.864	MM R	0.7380	8671.35352	195.81895	47.9623

HPLC chromatogram of enantiopure **6p**

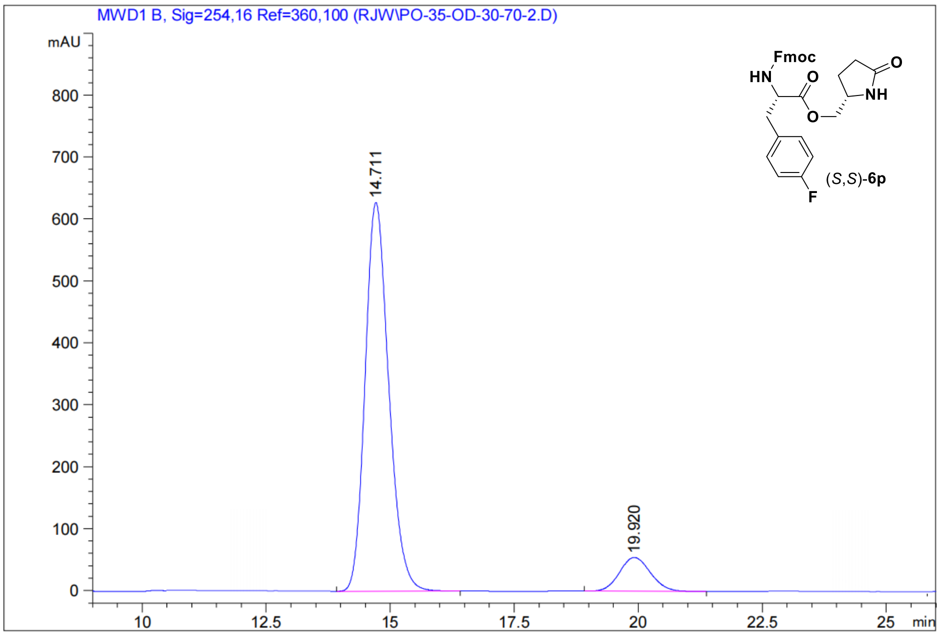

RT [min]	Type	Width [min]	Area	Height	Area%
14.711	BB	0.5080	2.07715e4	627.83606	91.0800
19.920	BB	0.6779	2034.28174	54.56969	8.9200

HPLC chromatogram of racemic **6q**

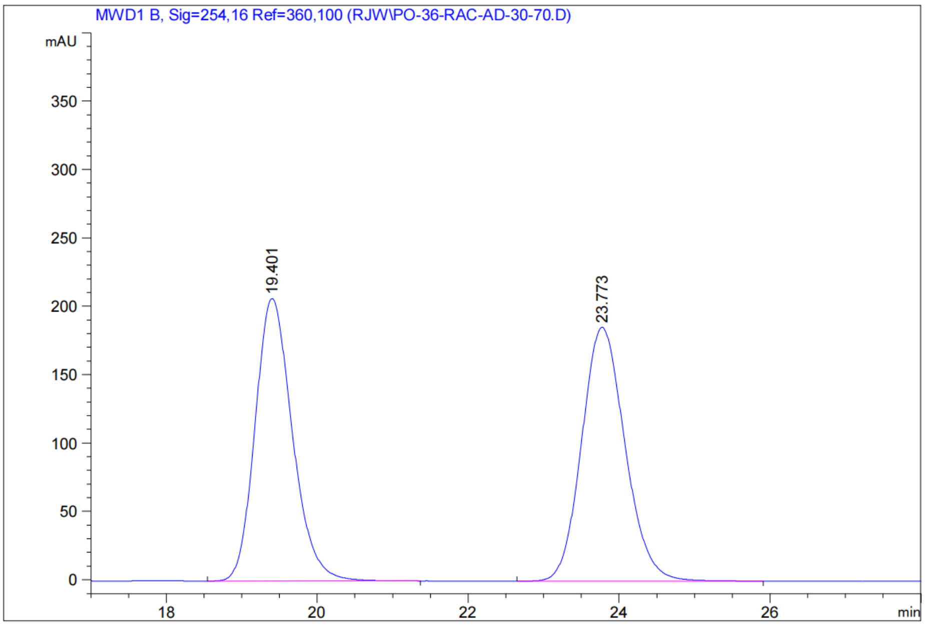

RT [min]	Type	Width [min]	Area	Height	Area%
19.401	BB	0.5287	7087.02979	206.36700	48.9111
23.773	BB	0.6175	7402.57813	185.54364	51.0889

HPLC chromatogram of enantiopure **6q**

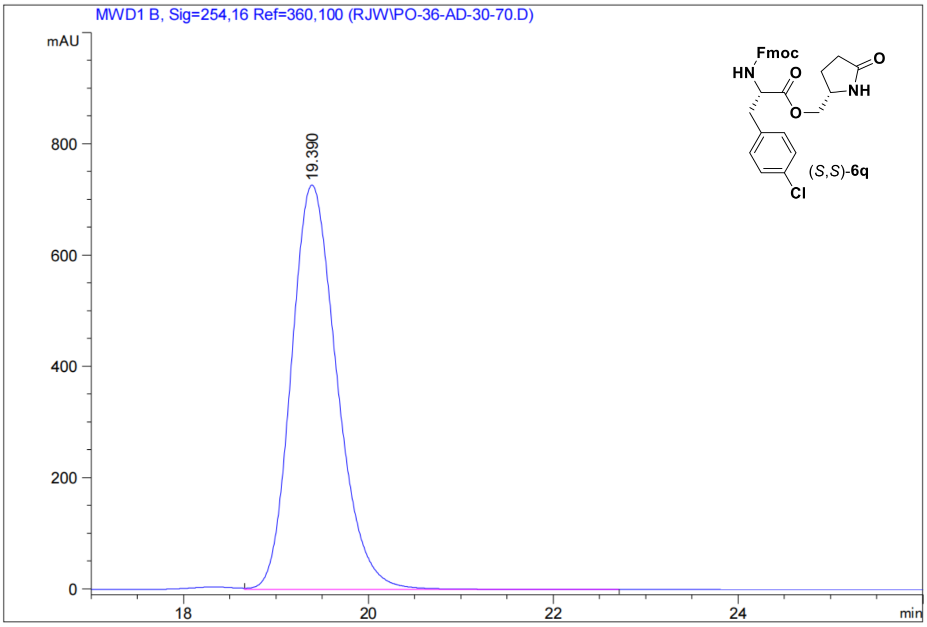

RT [min]	Type	Width [min]	Area	Height	Area%
19.390	VB	0.5301	2.49299e4	726.97772	100.0000

HPLC chromatogram of racemic **6r**

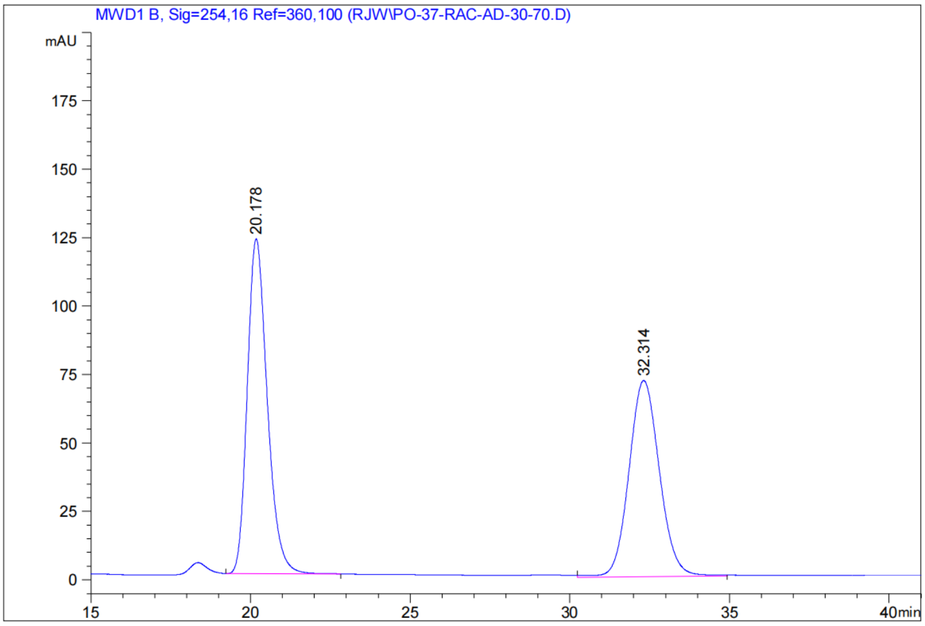

RT [min]	Type	Width [min]	Area	Height	Area%
20.178	BB	0.6649	5313.19238	122.28440	52.6712
32.314	MM R	1.1094	4774.27441	71.72147	47.3288

HPLC chromatogram of enantiopure **6r**

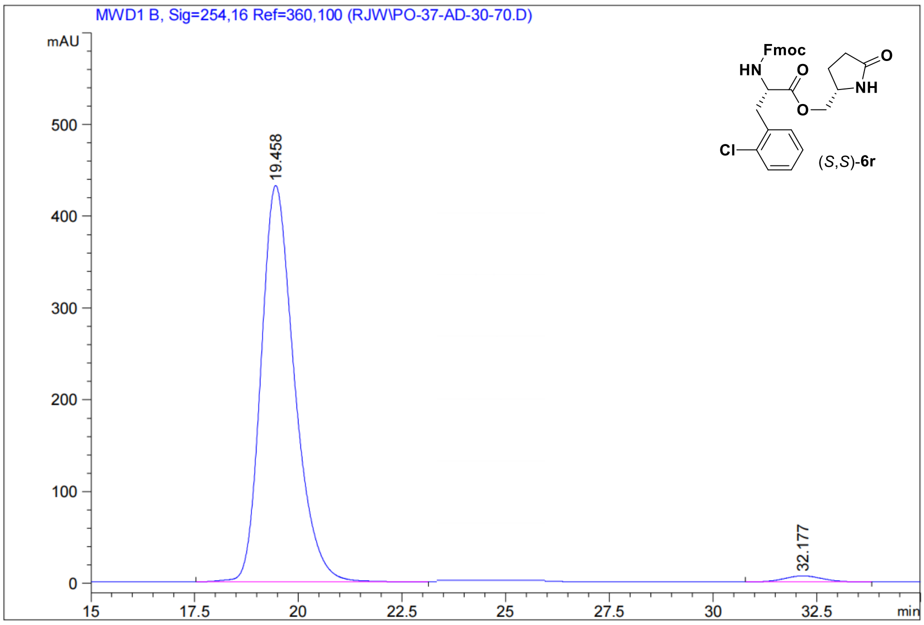

RT [min]	Type	Width [min]	Area	Height	Area%
19.458	BB	0.8497	2.41100e4	431.59012	98.3336
32.177	BB	0.7652	408.57983	6.48404	1.6664

HPLC chromatogram of racemic **6s**

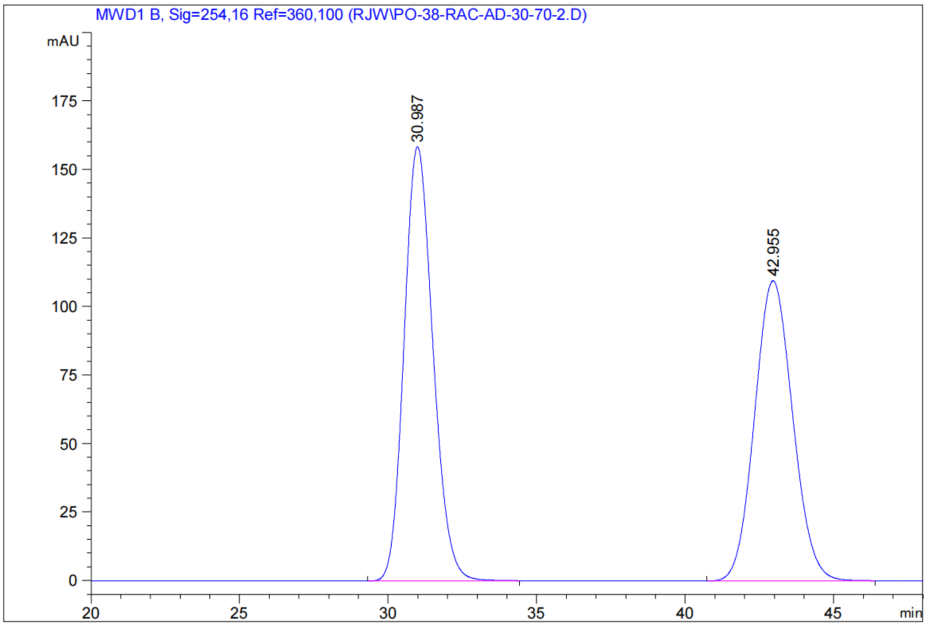

RT [min]	Type	Width [min]	Area	Height	Area%
30.987	BB	1.0514	1.06878e4	158.35825	52.2344
42.955	BB	1.3693	9773.40918	109.50440	47.7656

HPLC chromatogram of enantiopure **6s**

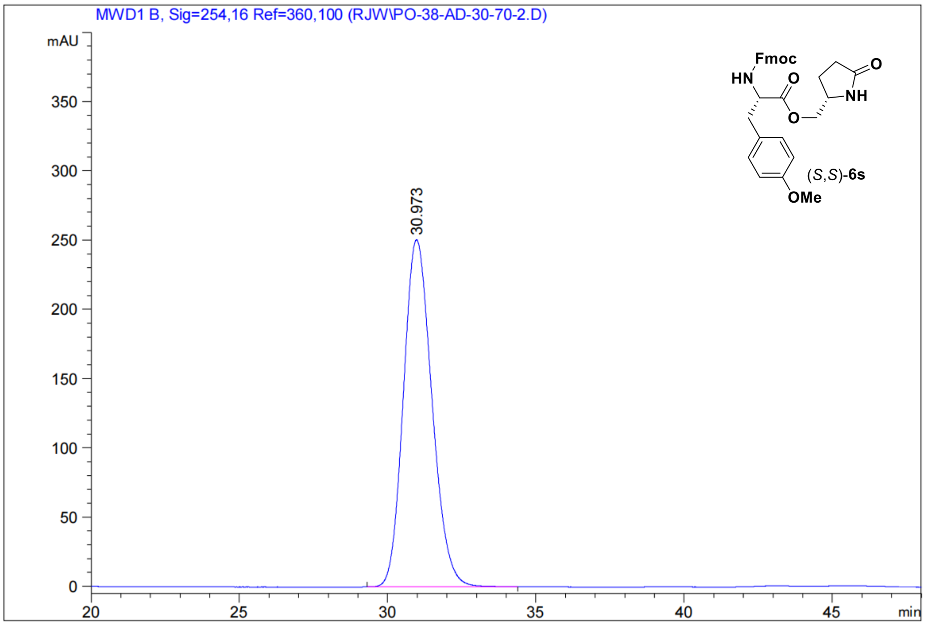

RT [min]	Type	Width [min]	Area	Height	Area%
30.973	BB	1.0299	1.67586e4	250.70291	100.0000

HPLC chromatogram of racemic **6t**

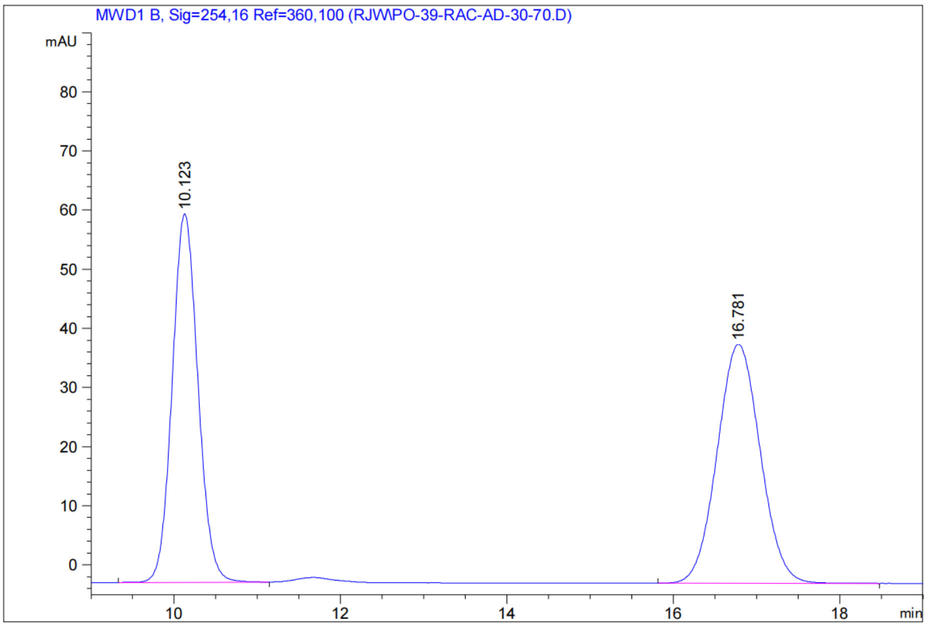

RT [min]	Type	Width [min]	Area	Height	Area%
10.123	BB	0.3349	1339.99304	62.31585	47.9344
16.781	BB	0.5601	1455.47717	40.41769	52.0656

HPLC chromatogram of enantiopure **6t**

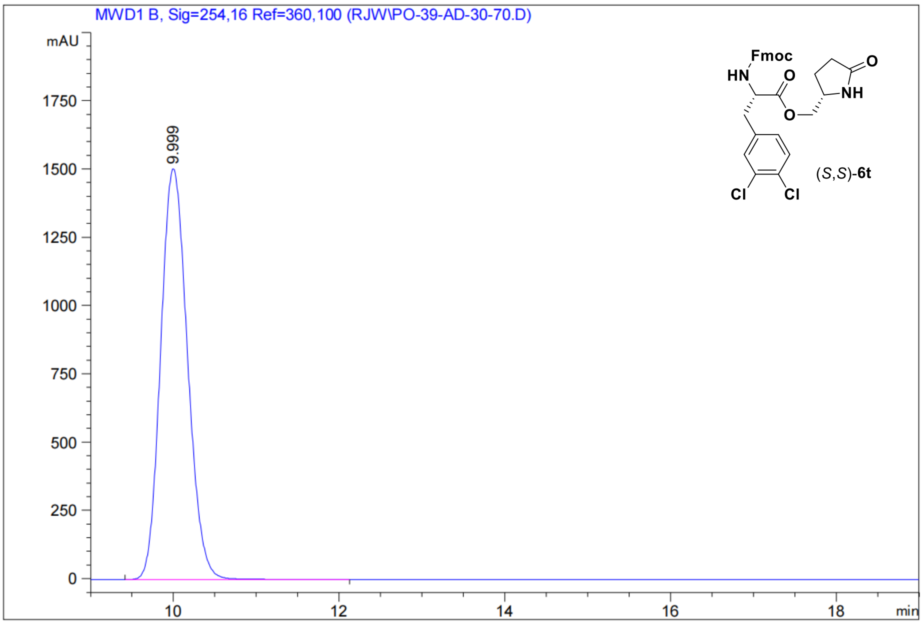

RT [min]	Type	Width [min]	Area	Height	Area%
9.999	VB	0.3524	3.38067e4	1503.33386	100.0000

HPLC chromatogram of racemic **6u**

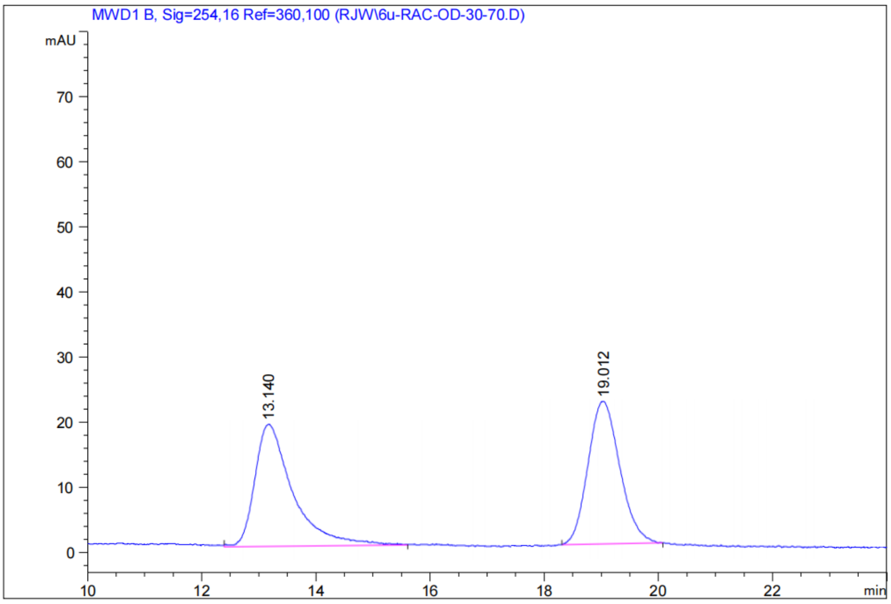

RT [min]	Type	Width [min]	Area	Height	Area%
13.140	MM R	0.8006	899.18360	19.13385	49.4996
19.012	BB	0.5307	917.36473	23.12346	50.5004

HPLC chromatogram of enantiopure **6u**

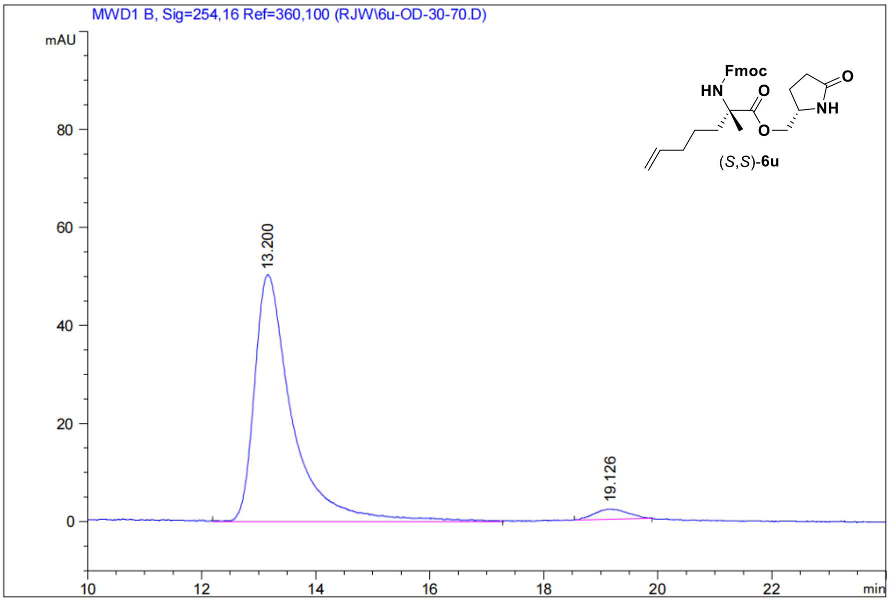

RT [min]	Type	Width [min]	Area	Height	Area%
13.200	MM R	0.7235	2395.94527	50.24635	95.0098
19.126	MM R	0.6997	125.84224	2.00612	4.9902

HPLC chromatogram of racemic **6v**

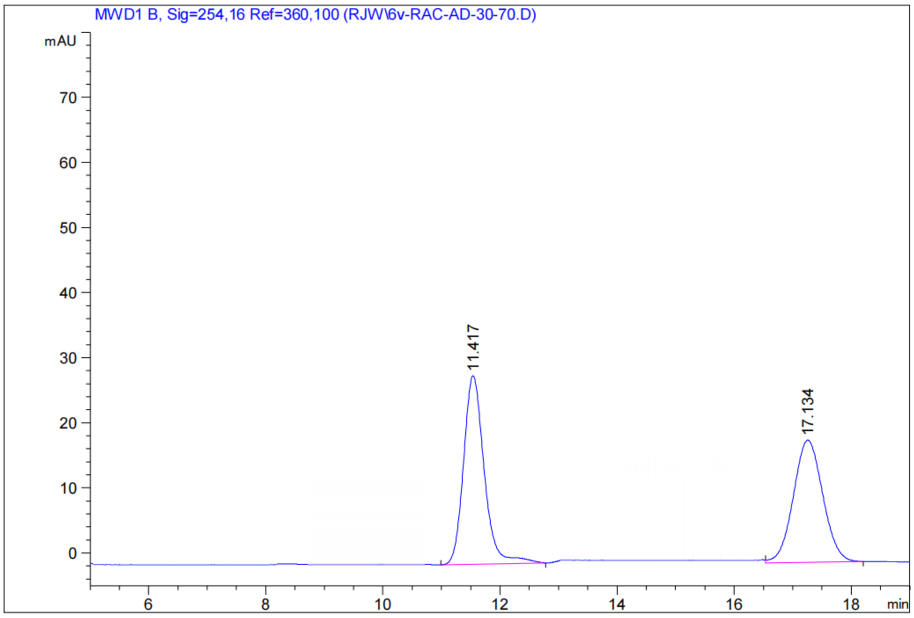

RT [min]	Type	Width [min]	Area	Height	Area%
11.417	BB	0.3687	700.56732	29.05562	50.5006
17.134	VB	0.5288	686.67835	19.09293	49.4994

HPLC chromatogram of enantiopure **6v**

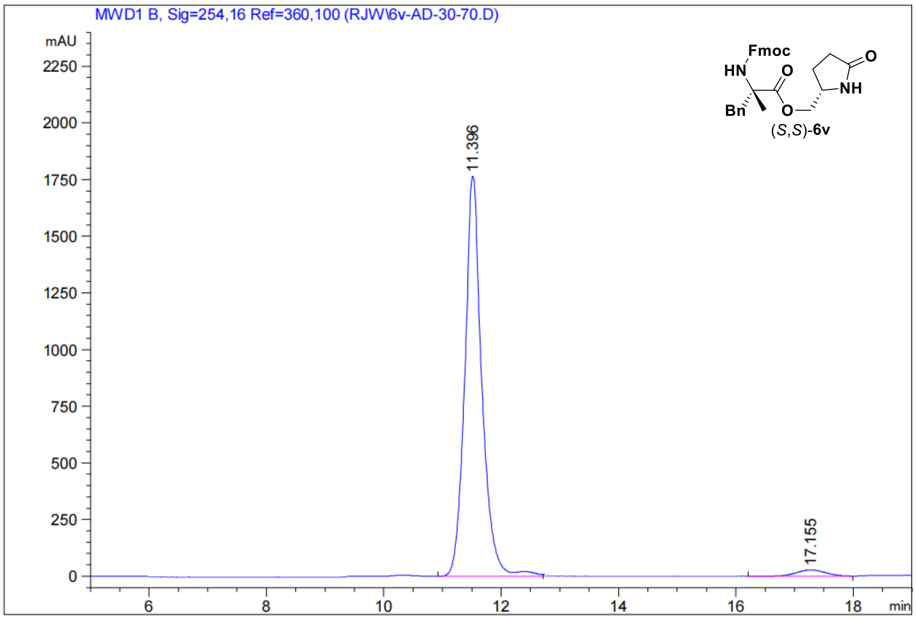

RT [min]	Type	Width [min]	Area	Height	Area%
11.396	MM R	0.3409	3.56781e4	1766.03561	96.0945
17.155	BV	0.5588	1450.03949	27.62743	3.9055

HPLC chromatogram of racemic **1a**

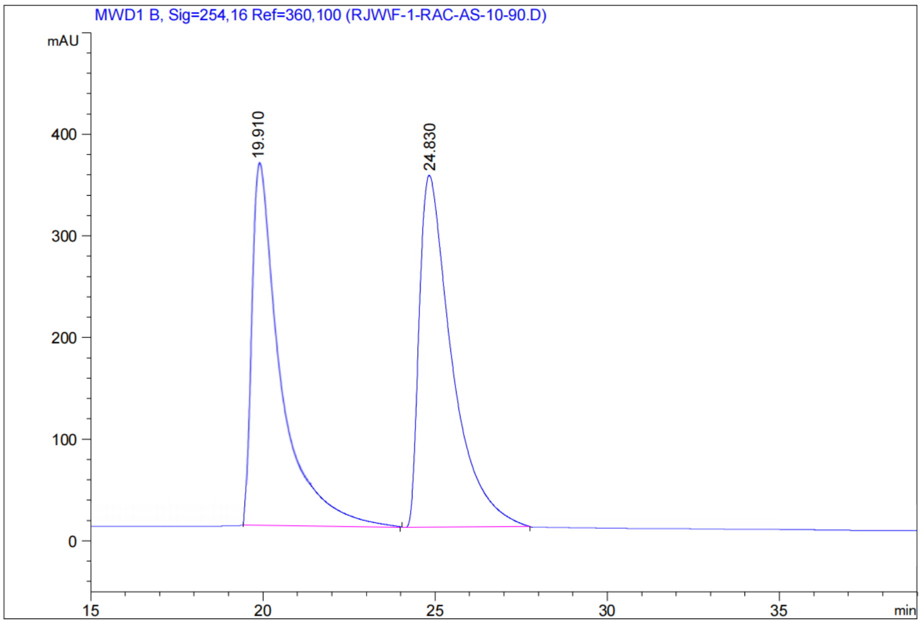

RT [min]	Type	Width [min]	Area	Height	Area%
19.910	MM R	1.3152	2.08885e4	362.29640	47.8879
24.830	MM R	1.0948	2.27311e4	346.04376	52.1121

HPLC chromatogram of enantiopure **1a**

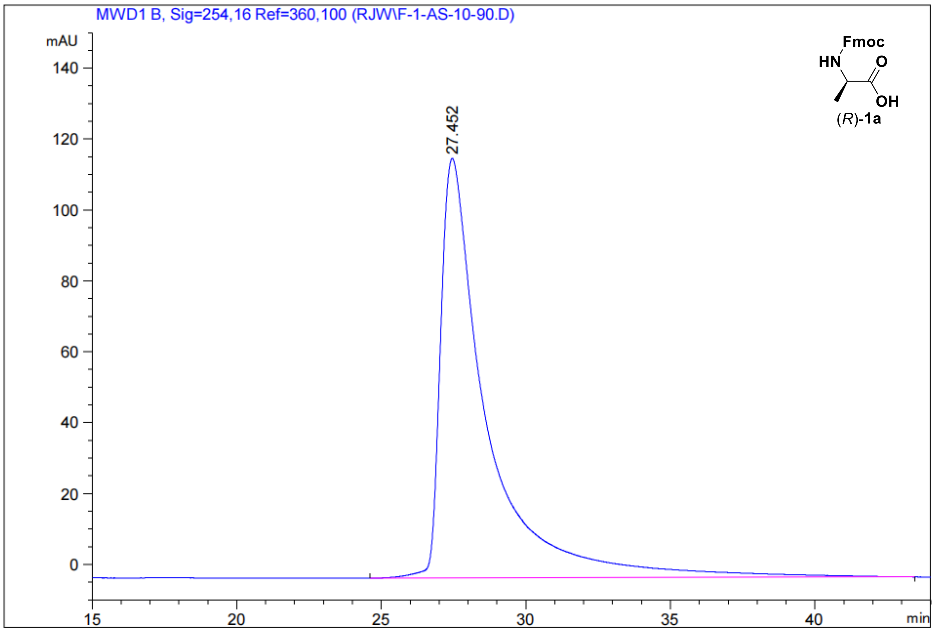

RT [min]	Type	Width [min]	Area	Height	Area%
27.452	BB	1.5822	1.32789e4	118.42163	100.0000

HPLC chromatogram of racemic **1b**

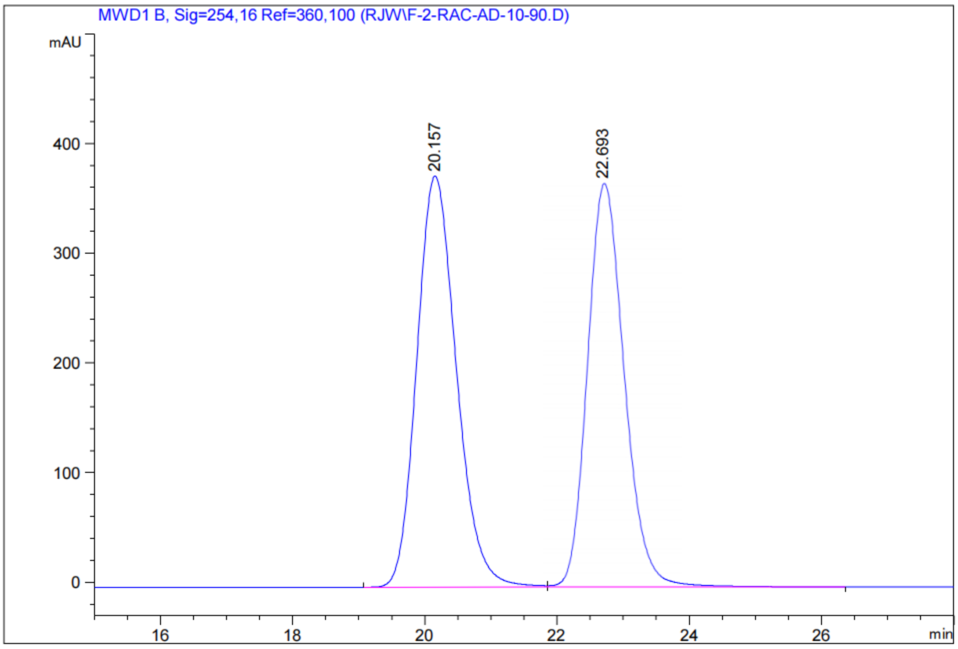

RT [min]	Type	Width [min]	Area	Height	Area%
20.157	BV	0.6437	1.56042e4	374.81952	50.5956
22.693	VB	0.6186	1.52368e4	370.63165	49.4044

HPLC chromatogram of enantiopure **1b**

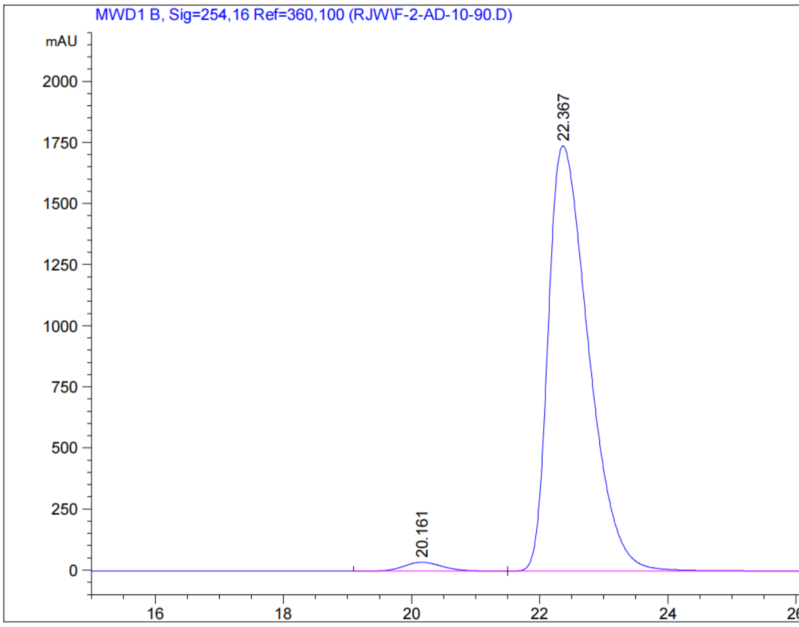

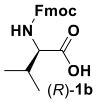

RT [min]	Type	Width [min]	Area	Height	Area%
20.161	BV	0.6509	1538.20996	36.26377	1.9890
22.367	VB	0.6723	7.57973e4	1739.74841	98.0110

HPLC chromatogram of racemic **1c**

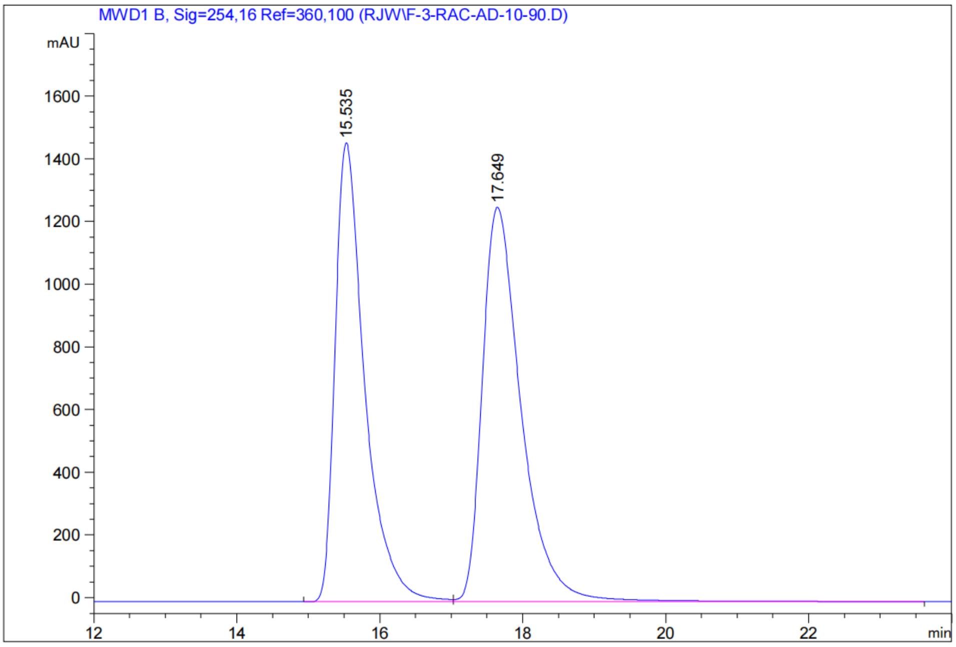

RT [min]	Type	Width [min]	Area	Height	Area%
15.535	BV	0.4320	4.21606e4	1463.73193	46.9364
17.649	VB	0.5739	4.76643e4	1258.46118	53.0636

HPLC chromatogram of enantiopure **1c**

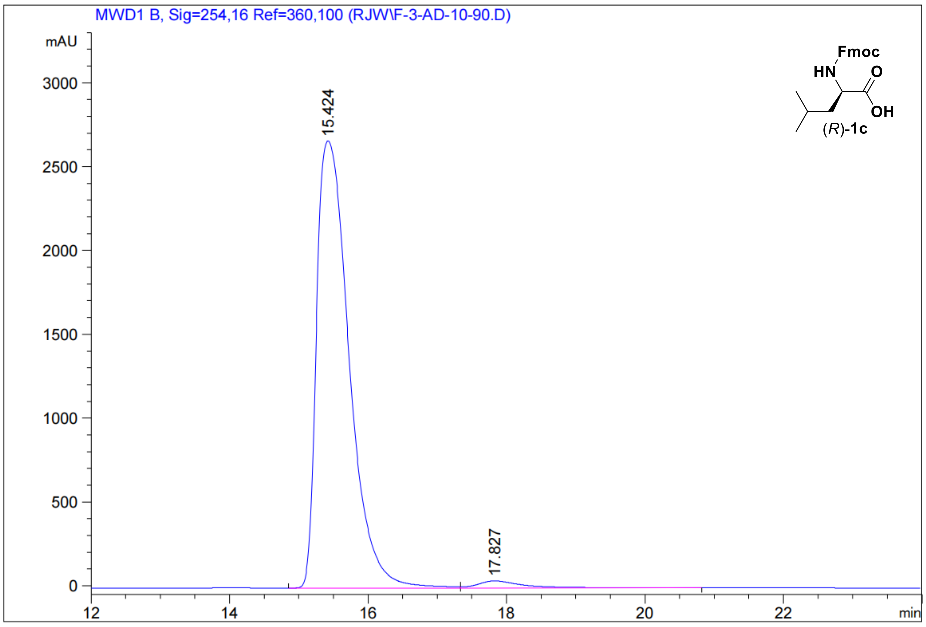

RT [min]	Type	Width [min]	Area	Height	Area%
15.424	BV	0.5074	8.67228e4	2667.01025	97.8412
17.827	VB	0.6569	1913.46777	42.05052	2.1588

HPLC chromatogram of racemic **1d**

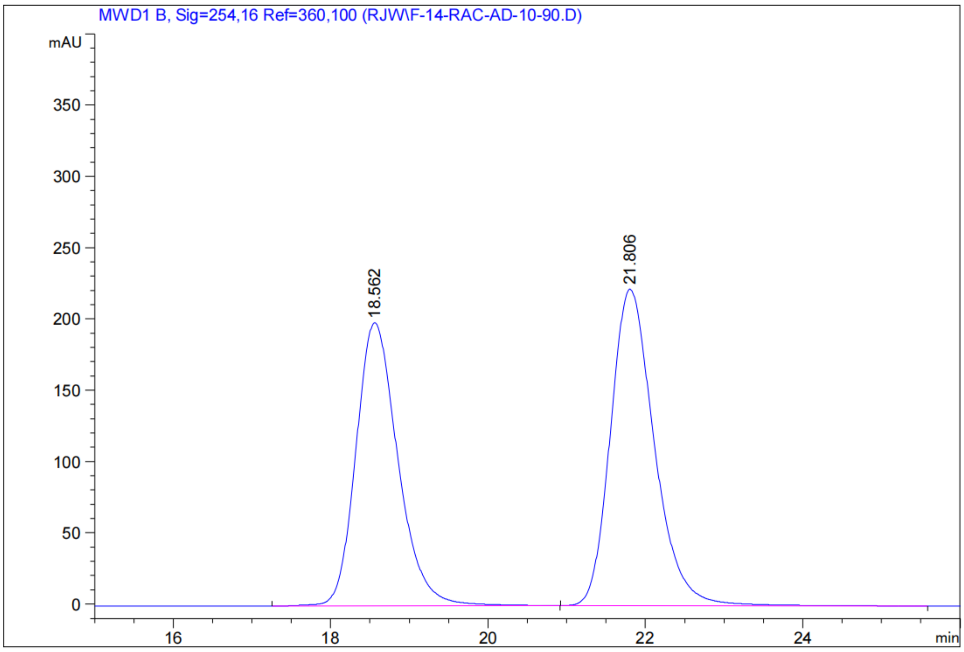

RT [min]	Type	Width [min]	Area	Height	Area%
18.562	BB	0.5707	7666.61384	198.55254	47.1927
21.806	BB	0.5931	8578.72461	221.88132	52.8073

HPLC chromatogram of enantiopure **1d**

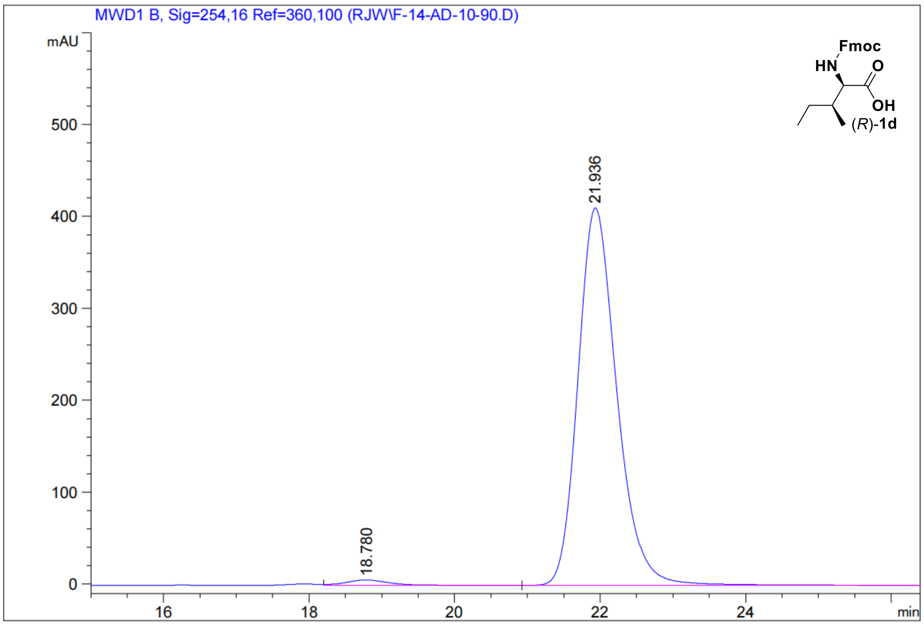

RT [min]	Type	Width [min]	Area	Height	Area%
18.780	VB	0.5956	237.61938	5.78063	1.5325
21.936	BBA	0.5700	1.52680e4	410.40445	98.4675

HPLC chromatogram of racemic **1e**

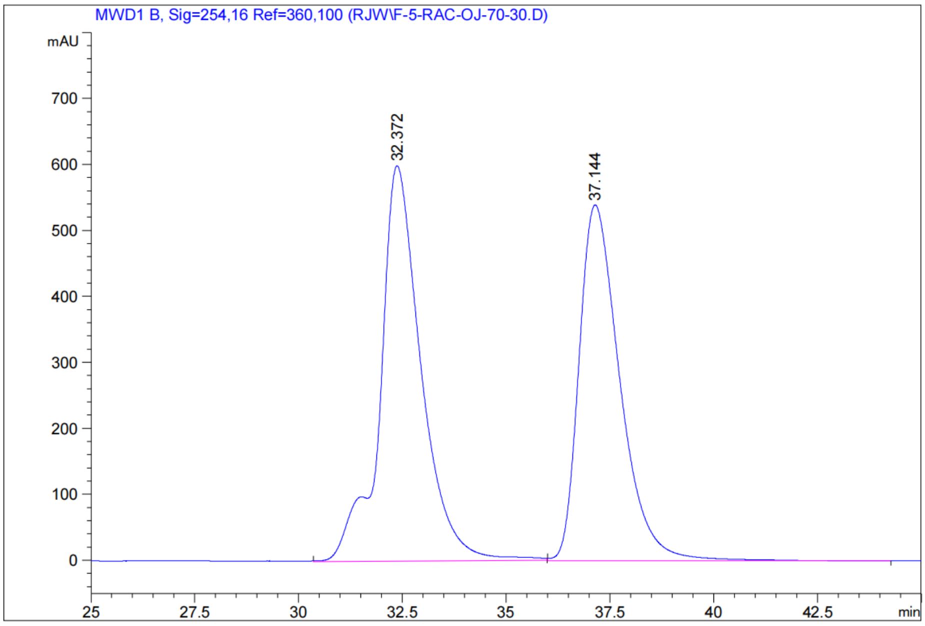

RT [min]	Type	Width [min]	Area	Height	Area%
32.372	MM R	1.1496	4.13234e4	599.12378	52.8051
37.144	VB	1.0508	3.69330e4	539.41455	47.1949

HPLC chromatogram of enantiopure **1e**

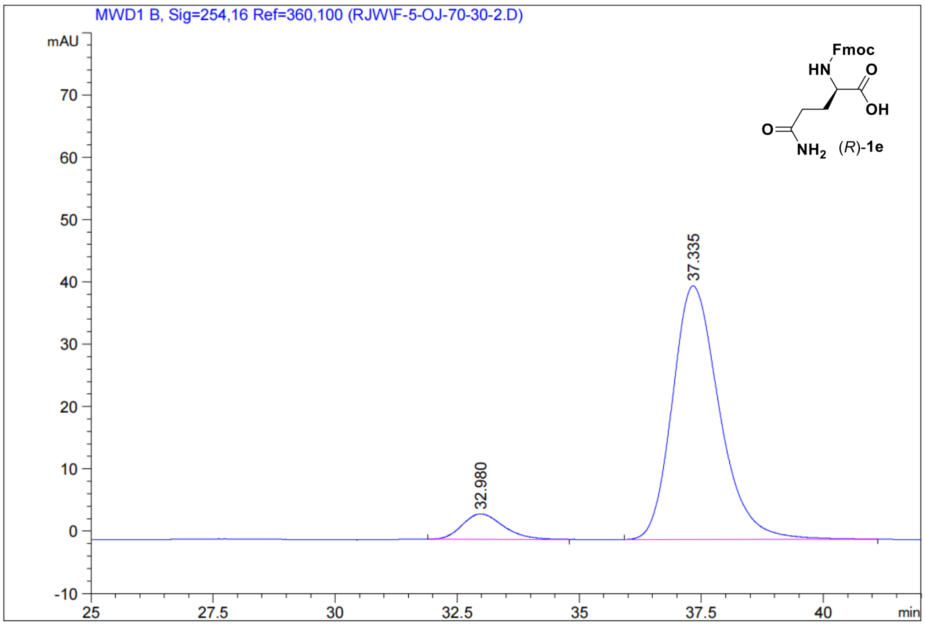

RT [min]	Type	Width [min]	Area	Height	Area%
32.980	BB	0.7077	238.04970	4.08621	8.0383
37.335	BB	1.0049	2723.38379	40.68693	91.9617

HPLC chromatogram of racemic **1f**

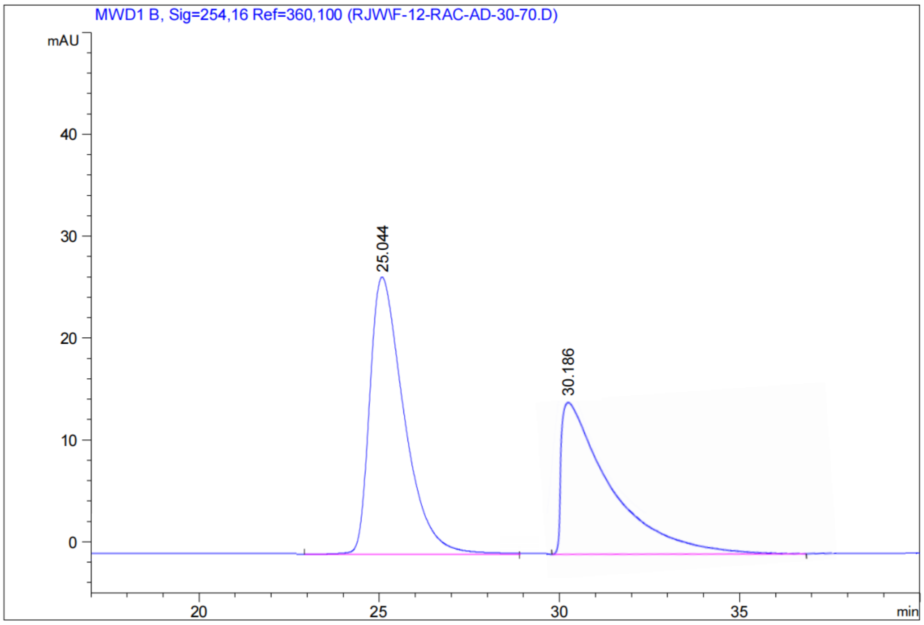

RT [min]	Type	Width [min]	Area	Height	Area%
25.044	MM R	0.8754	1571.63110	29.92359	51.8406
30.186	BB	1.2842	1460.02869	14.93975	48.1594

HPLC chromatogram of enantiopure **1f**

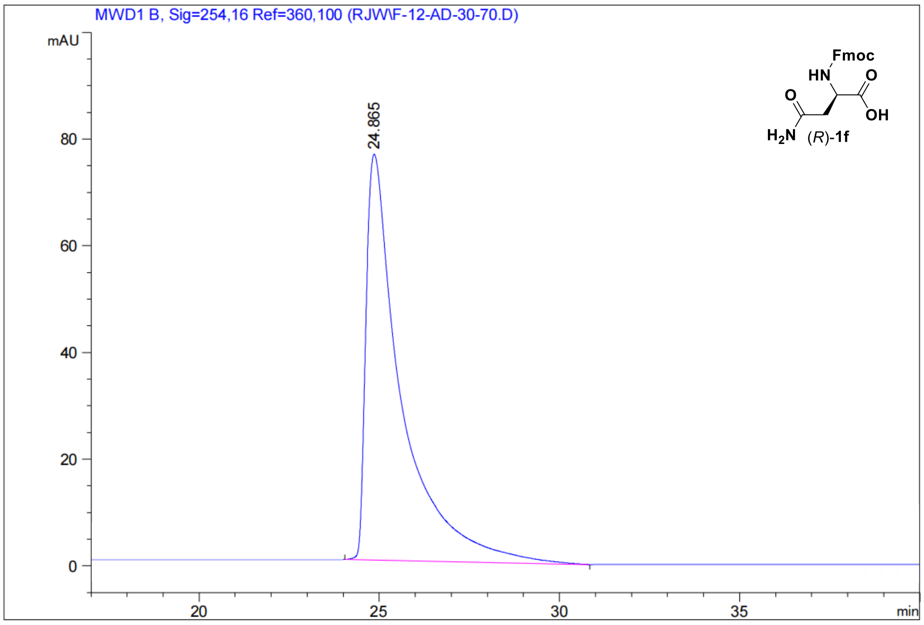

RT [min]	Type	Width [min]	Area	Height	Area%
24.865	BB	0.9579	5203.13574	76.07517	100.0000

HPLC chromatogram of racemic **1g**

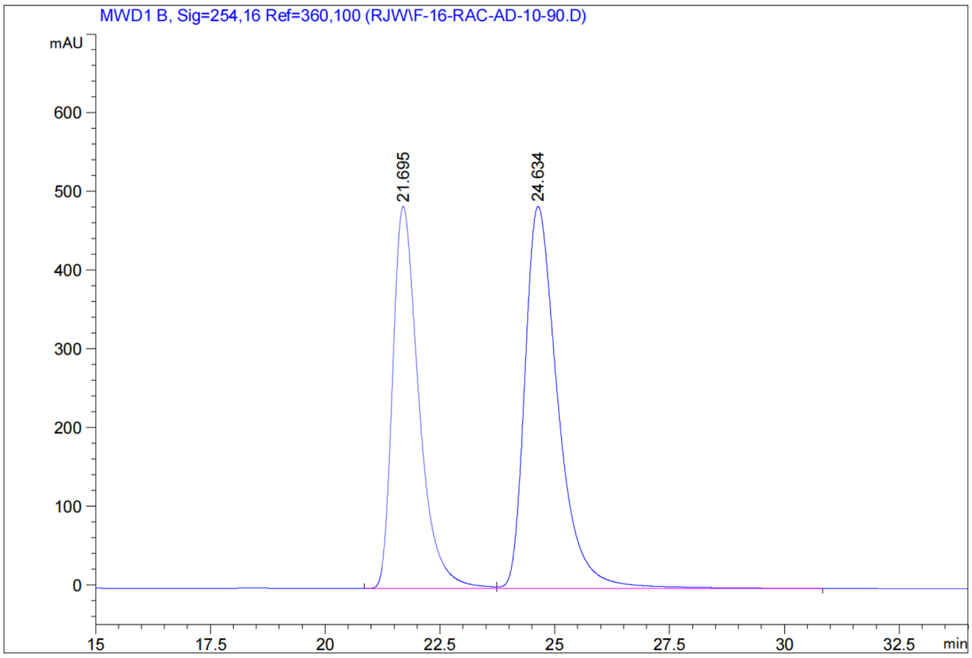

RT [min]	Type	Width [min]	Area	Height	Area%
21.695	BV	0.5943	2.21804e4	485.32355	47.7203
24.634	VB	0.7620	2.42996e4	485.41467	52.2797

HPLC chromatogram of enantiopure **1g**

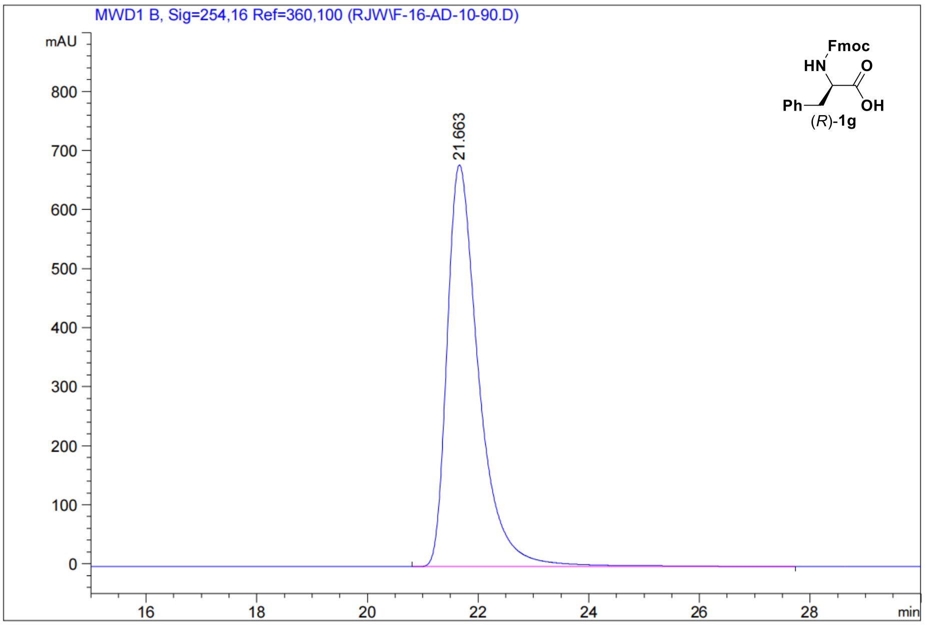

RT [min]	Type	Width [min]	Area	Height	Area%
21.663	BB	0.6013	2.71365e4	680.45734	100.0000

HPLC chromatogram of racemic **1h**

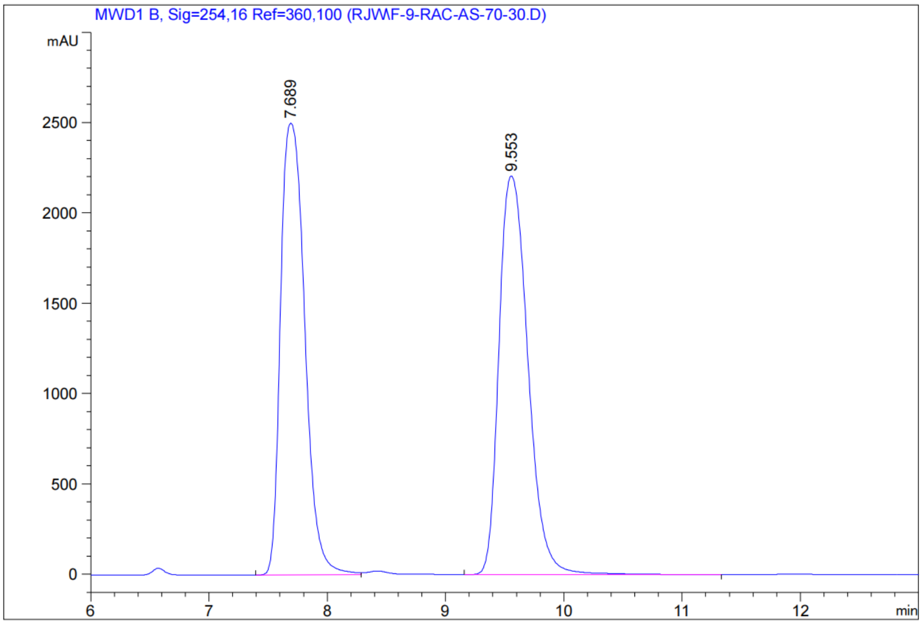

RT [min]	Type	Width [min]	Area	Height	Area%
7.689	BV	0.2258	3.55416e4	2501.17505	48.6204
9.553	BV	0.2685	3.75585e4	2207.12402	51.3796

HPLC chromatogram of enantiopure **1h**

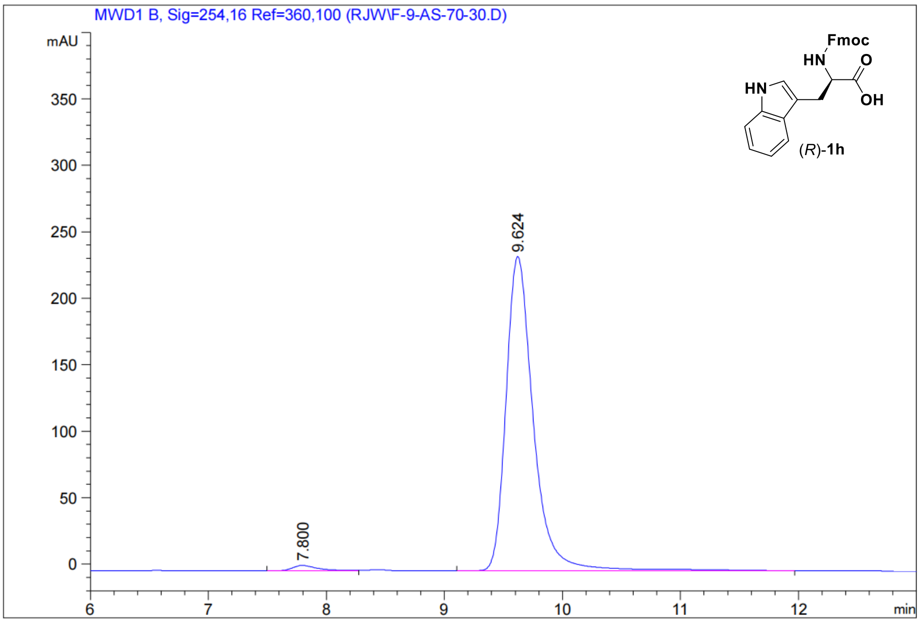

RT [min]	Type	Width [min]	Area	Height	Area%
7.800	BV	0.2247	60.71626	4.00785	1.5671
9.624	BB	0.2462	3813.68042	236.37741	98.4329

HPLC chromatogram of racemic **1i**

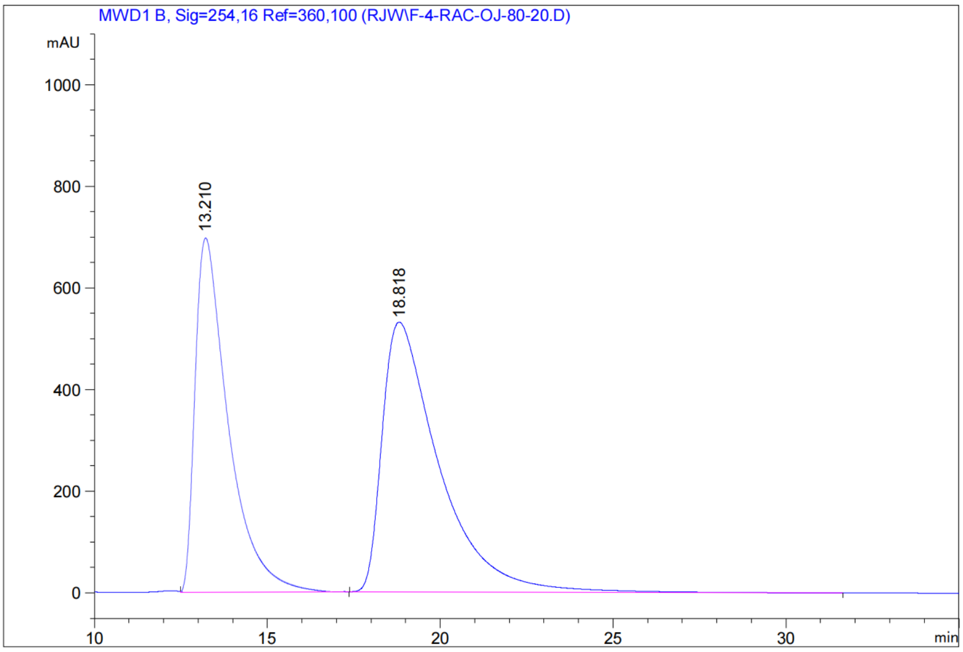

RT [min]	Type	Width [min]	Area	Height	Area%
13.210	VB	0.8163	5.60512e4	703.64523	48.0010
18.818	BB	1.6811	6.07197e4	530.58325	51.9990

HPLC chromatogram of enantiopure **1i**

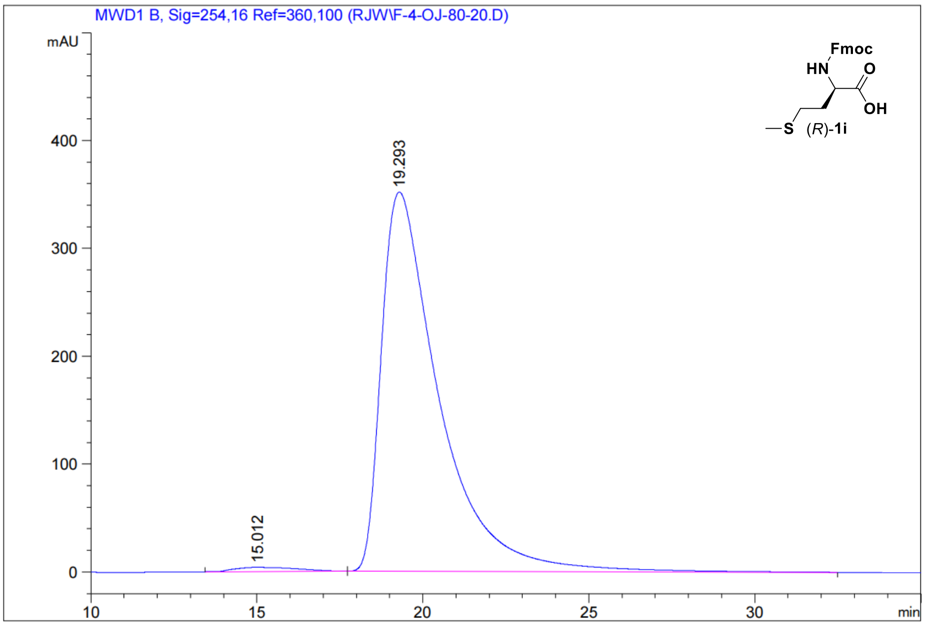

RT [min]	Type	Width [min]	Area	Height	Area%
15.012	BB	1.5460	516.33362	3.91153	1.1988
19.293	BB	1.7177	4.25546e4	351.69125	98.8012

HPLC chromatogram of racemic **1j**

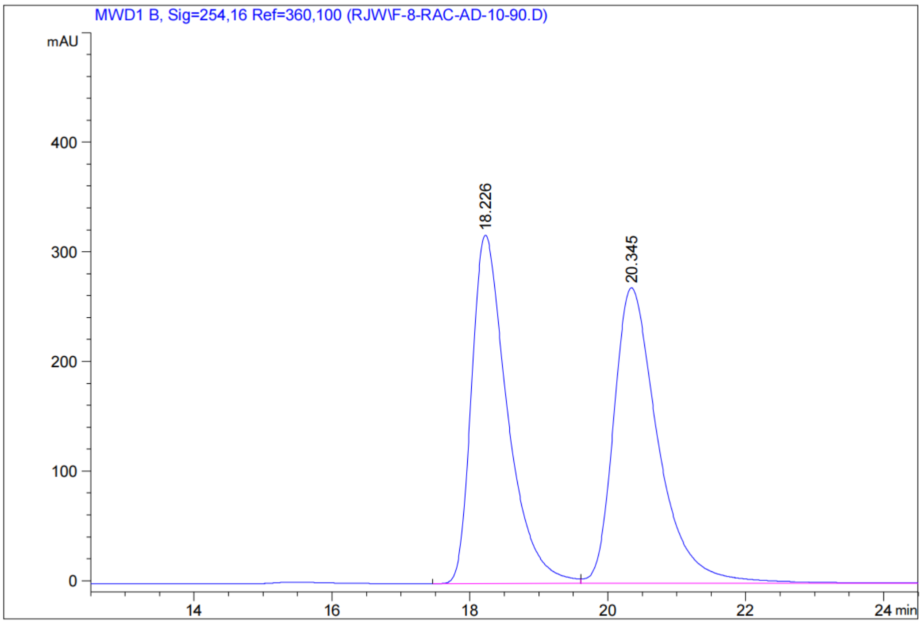

RT [min]	Type	Width [min]	Area	Height	Area%
18.226	BV	0.5437	1.14875e4	317.79190	48.7026
20.345	VBA	0.6753	1.20995e4	269.77411	51.2974

HPLC chromatogram of enantiopure **1j**

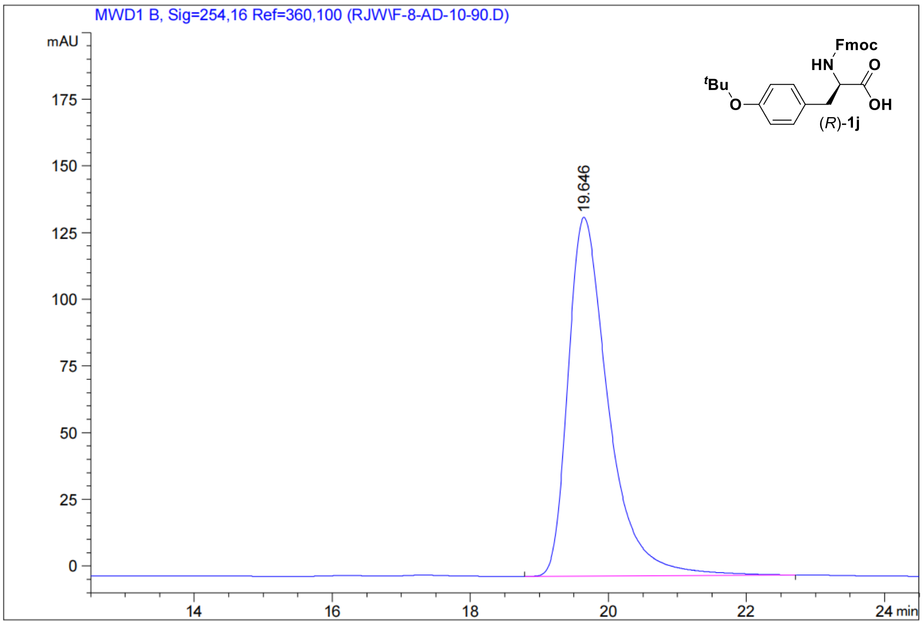

RT [min]	Type	Width [min]	Area	Height	Area%
19.646	BB	0.6120	5464.83984	134.50932	100.0000

HPLC chromatogram of racemic **1k**

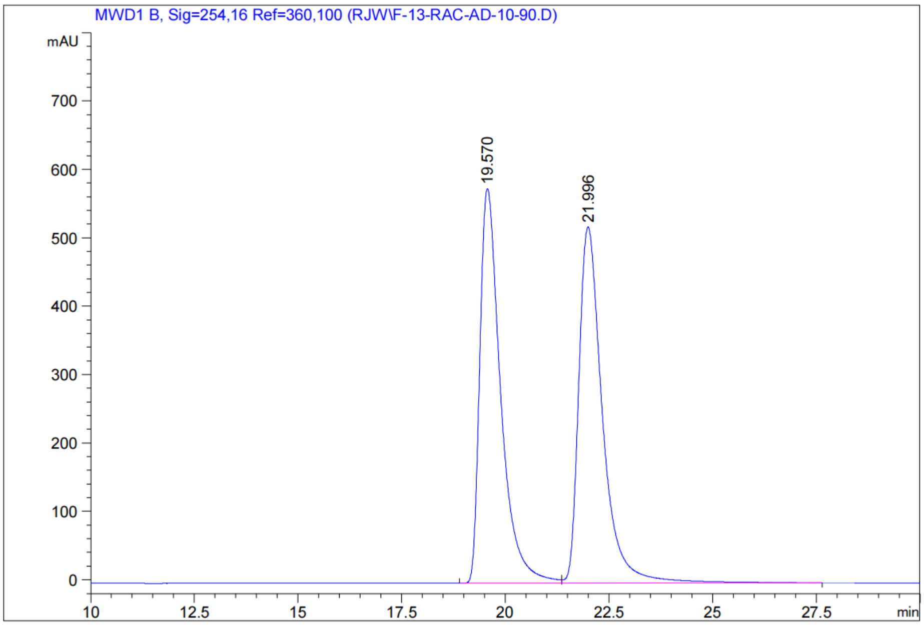

RT [min]	Type	Width [min]	Area	Height	Area%
19.570	BV	0.5258	2.01512e4	576.54028	49.3472
21.996	VB	0.5995	2.06843e4	520.60535	50.6528

HPLC chromatogram of enantiopure **1k**

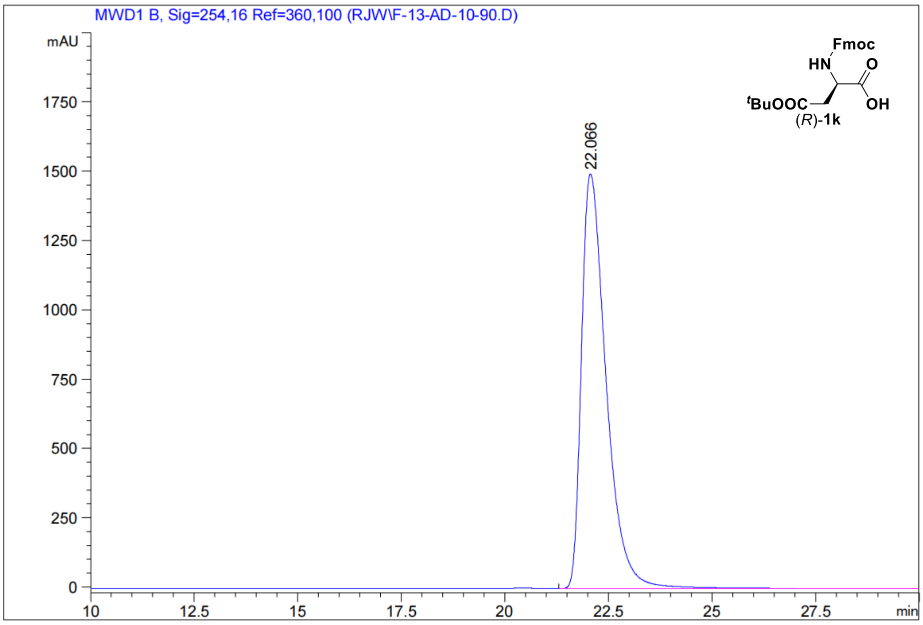

RT [min]	Type	Width [min]	Area	Height	Area%
22.066	VB	0.6300	6.20548e4	1495.76709	100.0000

HPLC chromatogram of racemic **1l**

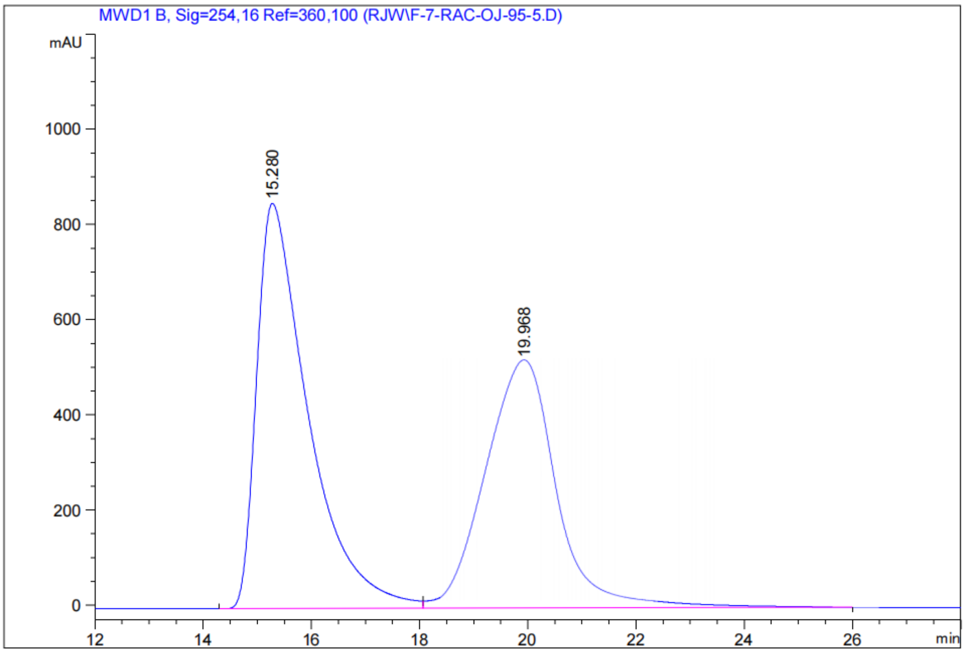

RT [min]	Type	Width [min]	Area	Height	Area%
15.280	BV	0.9838	5.61909e4	851.57410	50.6650
19.968	VB	1.6567	5.47158e4	521.29346	49.3350

HPLC chromatogram of enantiopure **1l**

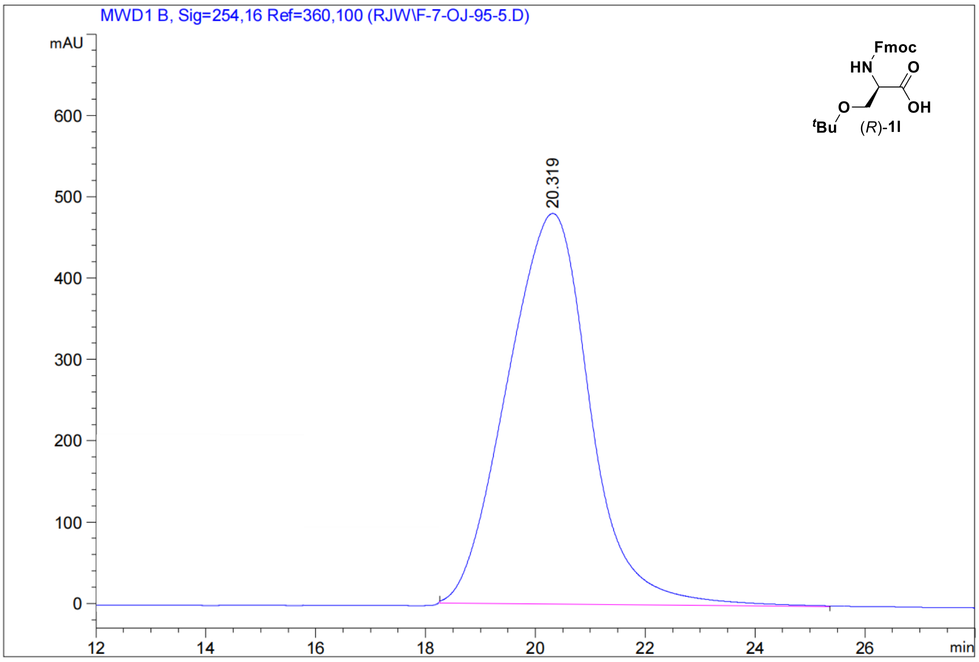

RT [min]	Type	Width [min]	Area	Height	Area%
20.319	MM R	1.7487	5.04013e4	480.37265	100.0000

HPLC chromatogram of racemic **1m**

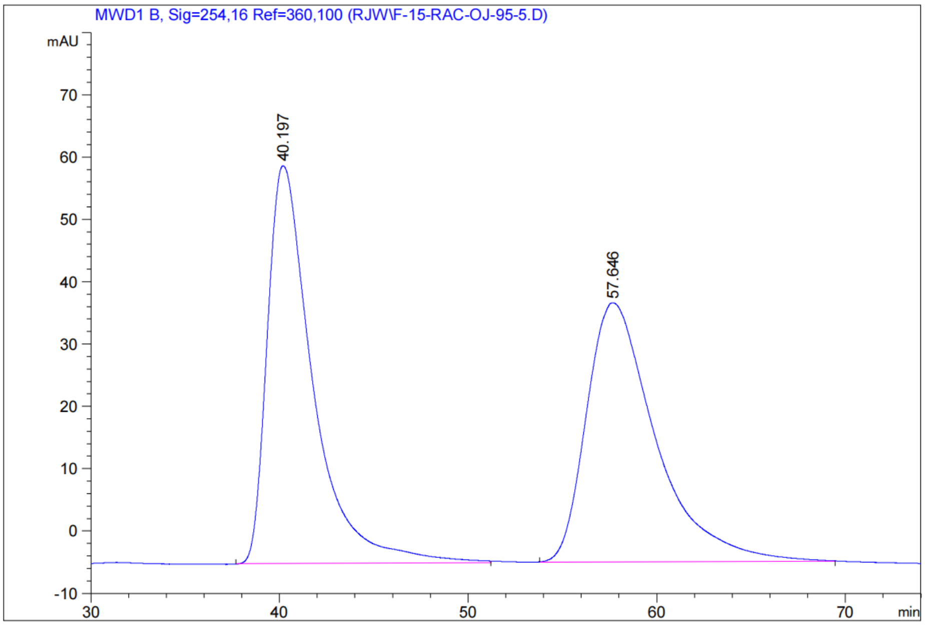

RT [min]	Type	Width [min]	Area	Height	Area%
40.197	MM R	2.8004	1.07237e4	63.82325	50.3639
57.646	BB	2.9748	1.05688e4	41.53203	49.6361

HPLC chromatogram of enantiopure **1m**

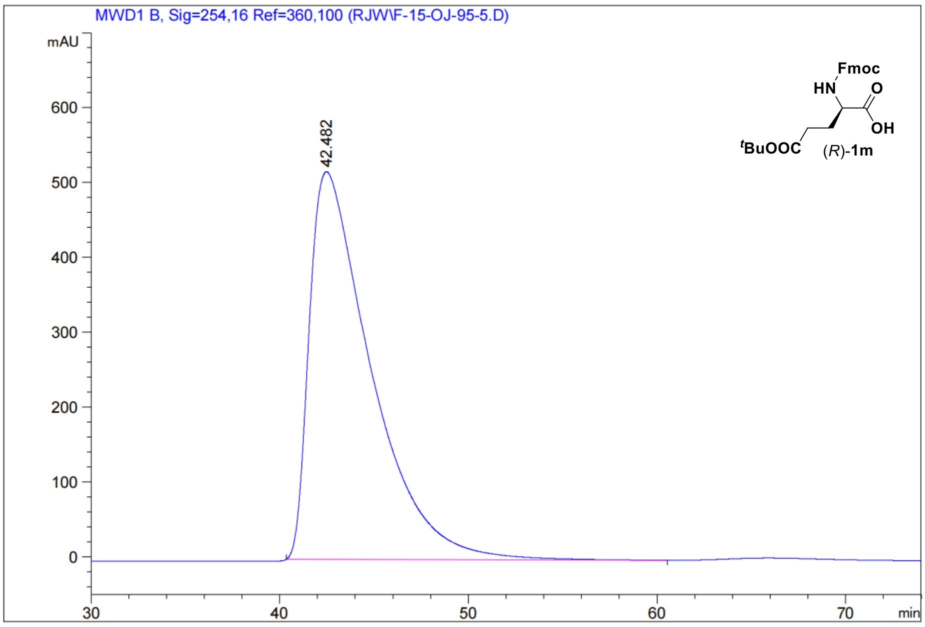

RT [min]	Type	Width [min]	Area	Height	Area%
42.482	BB	2.9376	1.17730e5	517.98792	100.0000

HPLC chromatogram of racemic **1n**

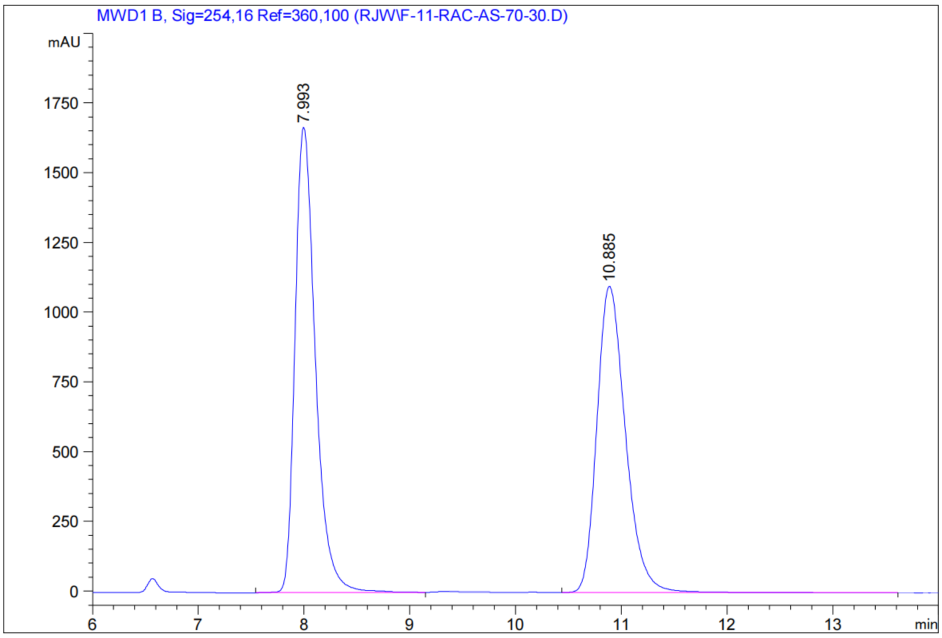

RT [min]	Type	Width [min]	Area	Height	Area%
7.993	BB	0.2062	2.23286e4	1667.79724	52.0779
10.885	BB	0.2903	2.05468e4	1098.01160	47.9221

HPLC chromatogram of enantiopure **1n**

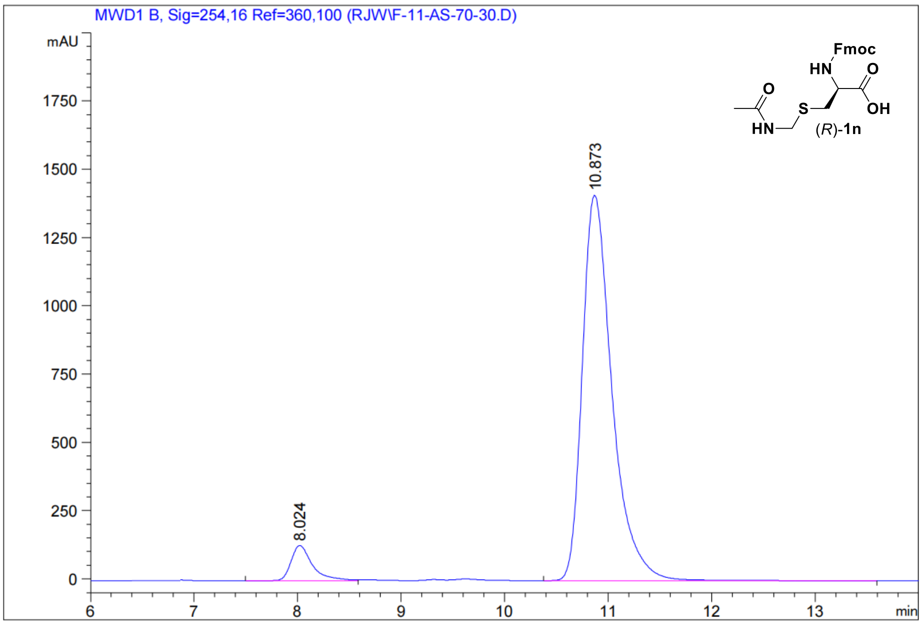

RT [min]	Type	Width [min]	Area	Height	Area%
8.024	BV	0.2130	1845.81384	128.94569	6.1535
10.873	BB	0.3029	2.81505e4	1410.26050	93.8465

HPLC chromatogram of racemic **1o**

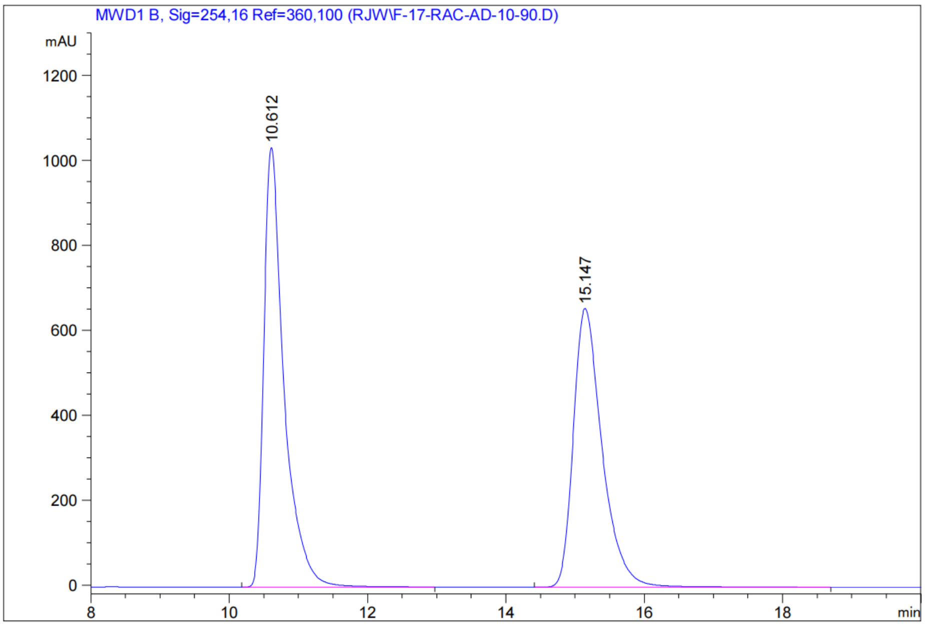

RT [min]	Type	Width [min]	Area	Height	Area%
10.612	BB	0.2984	2.09665e4	1034.59814	52.5684
15.147	BB	0.4365	1.89178e4	655.85992	47.4316

HPLC chromatogram of enantiopure **1o**

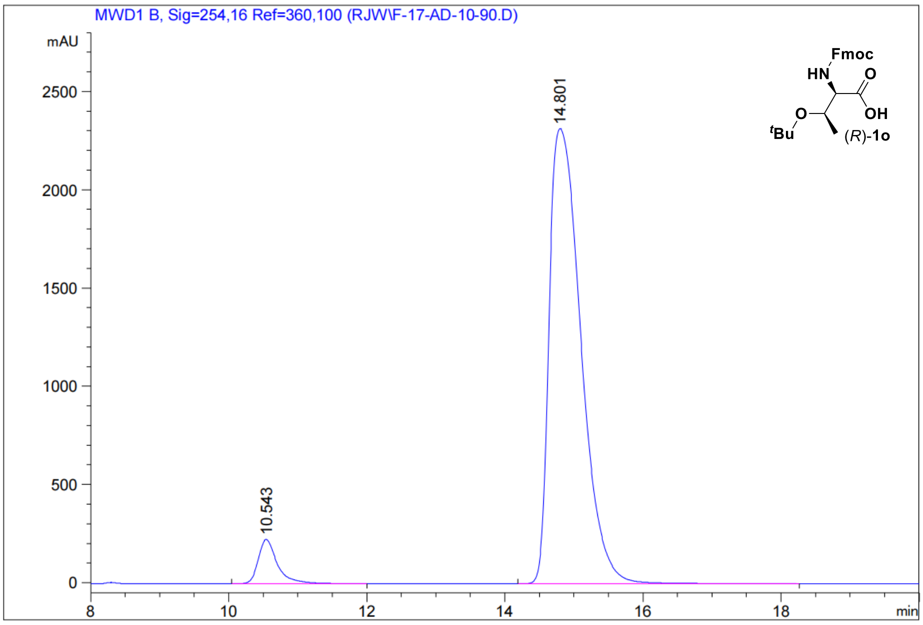

RT [min]	Type	Width [min]	Area	Height	Area%
10.543	BB	0.2873	4343.75195	224.94815	5.5693
14.801	BB	0.4989	7.36515e4	2316.61963	94.4307

HPLC chromatogram of racemic **1p**

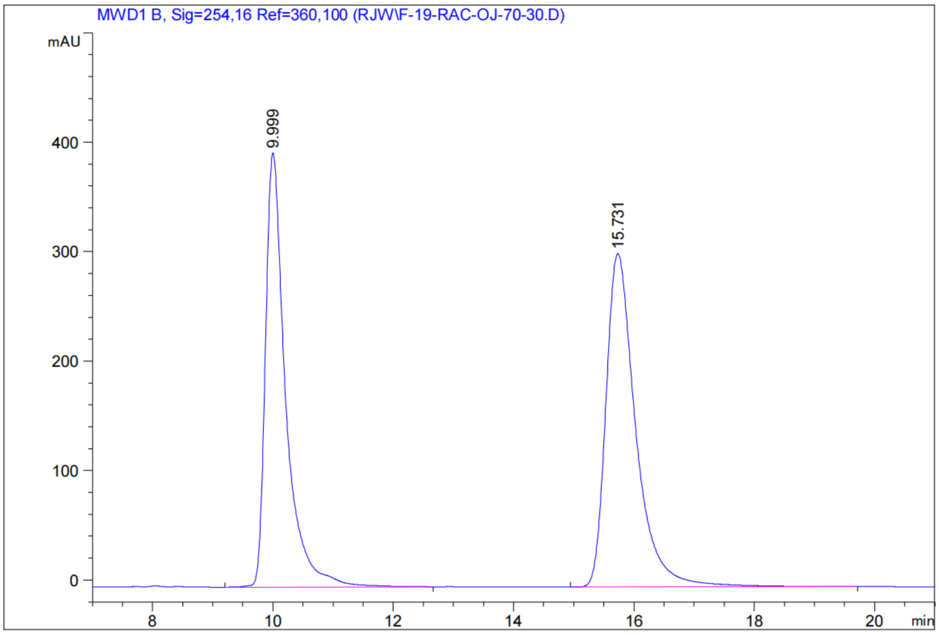

RT [min]	Type	Width [min]	Area	Height	Area%
9.999	BB	0.3434	9230.90430	396.77661	46.7693
15.731	BB	0.5183	1.05062e4	304.67087	53.2307

HPLC chromatogram of enantiopure **1p**

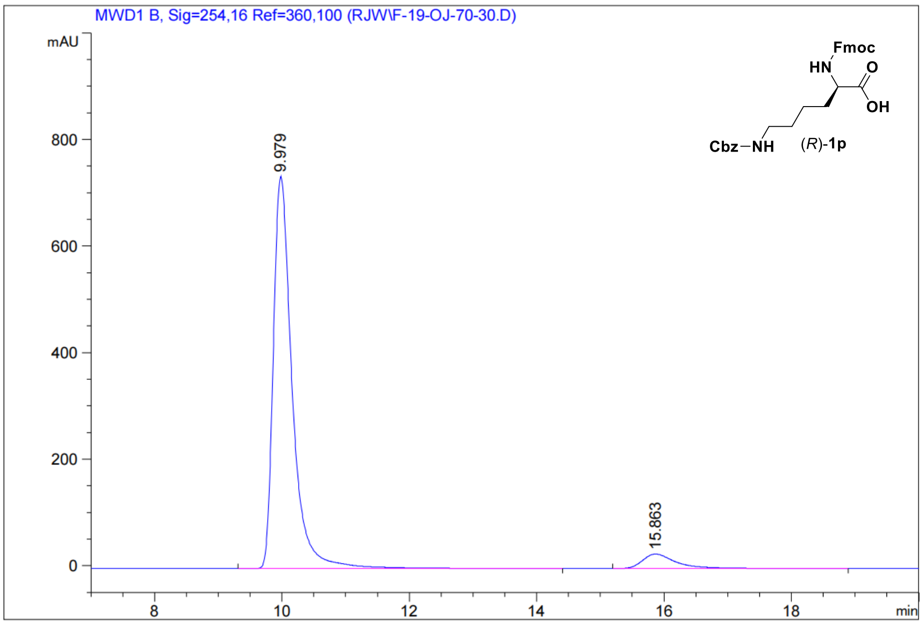

RT [min]	Type	Width [min]	Area	Height	Area%
9.979	BB	0.3104	1.51581e4	735.71289	93.7136
15.863	BB	0.5567	1016.82501	27.16550	6.2864

HPLC chromatogram of racemic (*S*)-**1a**

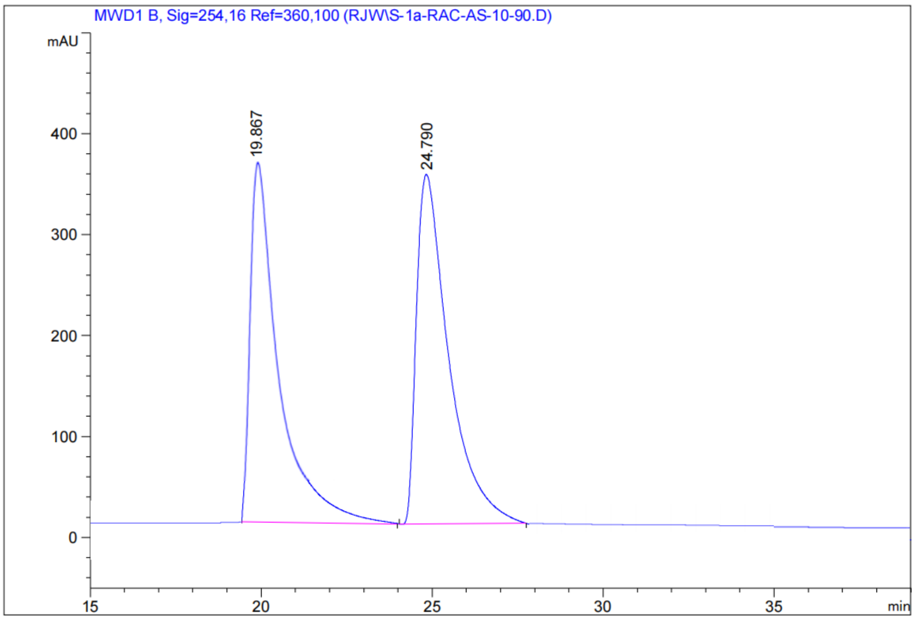

RT [min]	Type	Width [min]	Area	Height	Area%
19.867	MM R	1.3087	2.00097e4	361.79784	47.2572
24.790	MM R	1.1021	2.23324e4	345.94884	52.7428

HPLC chromatogram of enantiopure (*S*)-**1a**

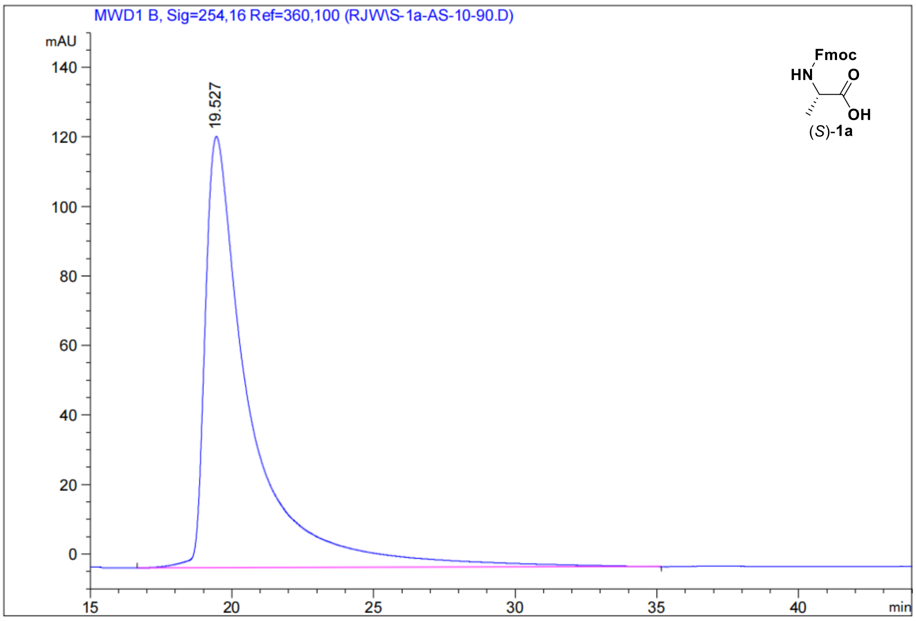

RT [min]	Type	Width [min]	Area	Height	Area%
19.527	BB	1.5798	1.29876e4	123.39154	100.0000

HPLC chromatogram of racemic **5a**

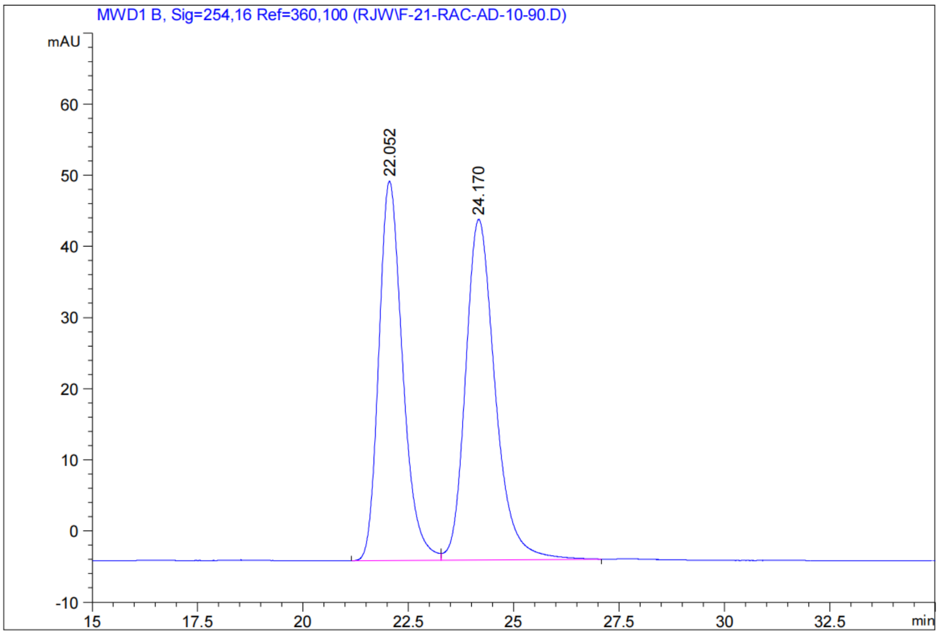

RT [min]	Type	Width [min]	Area	Height	Area%
22.052	BV	0.6025	2095.54590	53.31673	47.2331
24.170	VB	0.7506	2341.05493	47.86197	52.7669

HPLC chromatogram of enantiopure **5a**

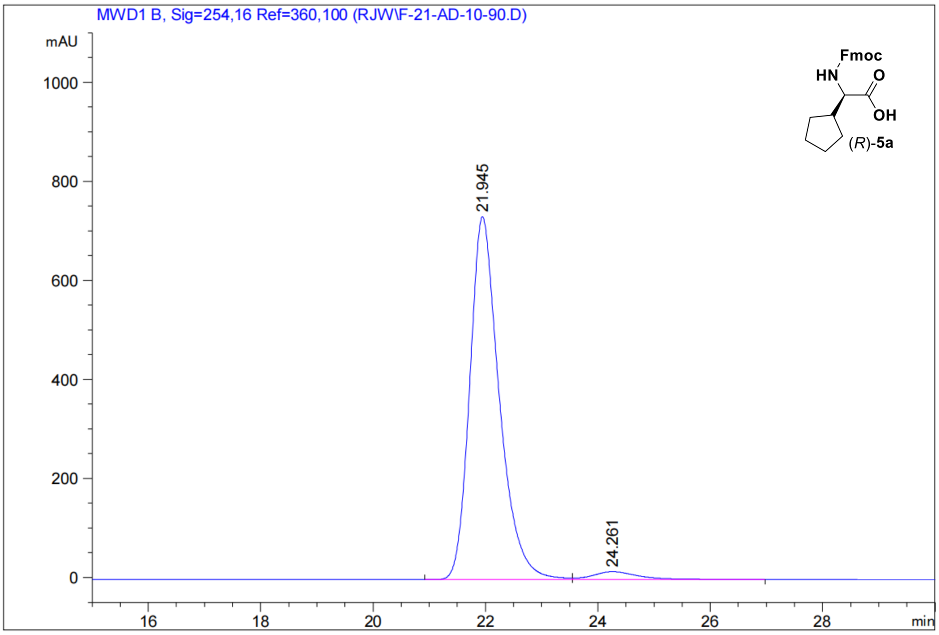

RT [min]	Type	Width [min]	Area	Height	Area%
21.945	BV	0.5459	2.67527e4	732.77948	96.7692
24.261	VB	0.8184	893.18536	15.82619	3.2308

HPLC chromatogram of racemic **5b**

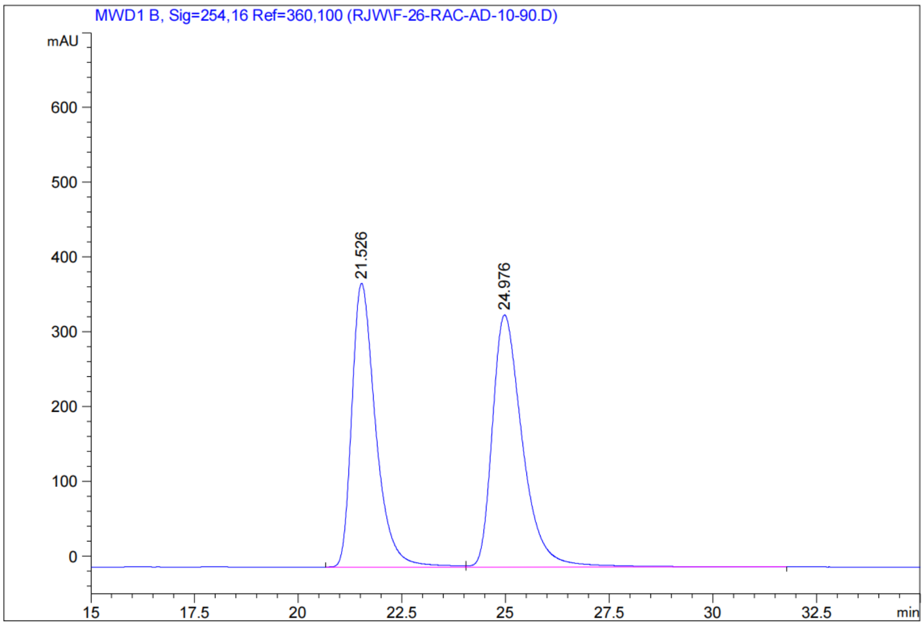

RT [min]	Type	Width [min]	Area	Height	Area%
21.526	BV	0.6061	1.51606e4	379.49991	47.1003
24.976	VB	0.7694	1.70273e4	337.05148	52.8997

HPLC chromatogram of enantiopure **5b**

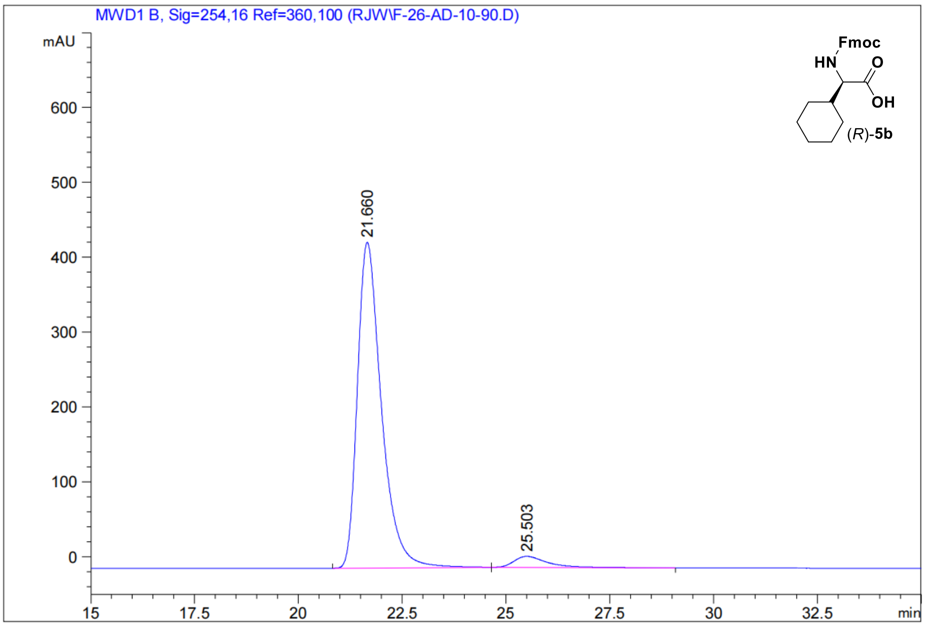

RT [min]	Type	Width [min]	Area	Height	Area%
21.660	BB	0.6070	1.74850e4	434.91583	95.5287
25.503	BB	0.8000	818.41046	14.68991	4.4713

HPLC chromatogram of racemic **5c**

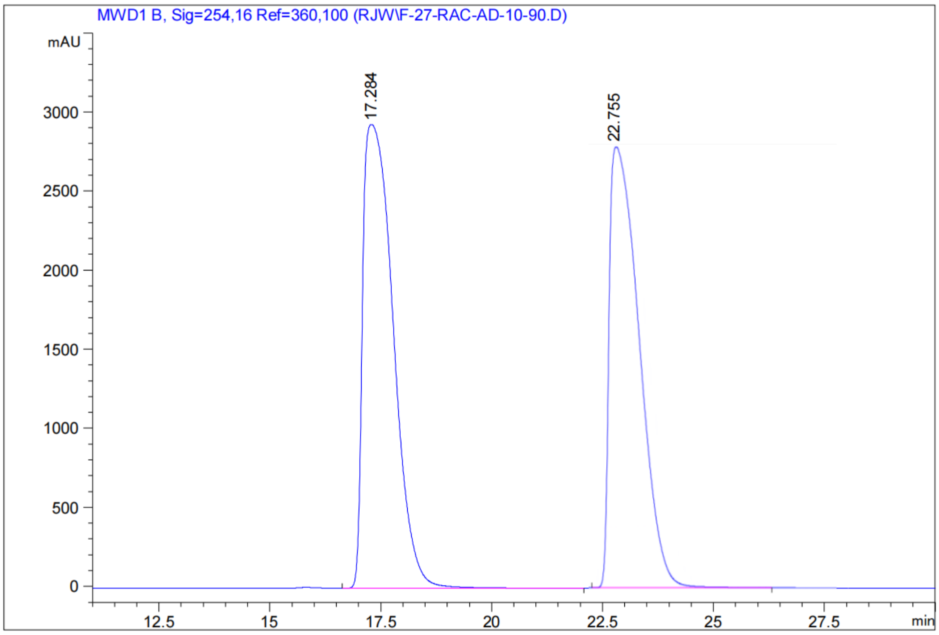

RT [min]	Type	Width [min]	Area	Height	Area%
17.284	BB	0.6432	1.39516e5	2931.97046	50.9361
22.755	MM R	0.9289	1.34388e5	2711.17700	49.0639

HPLC chromatogram of enantiopure **5c**

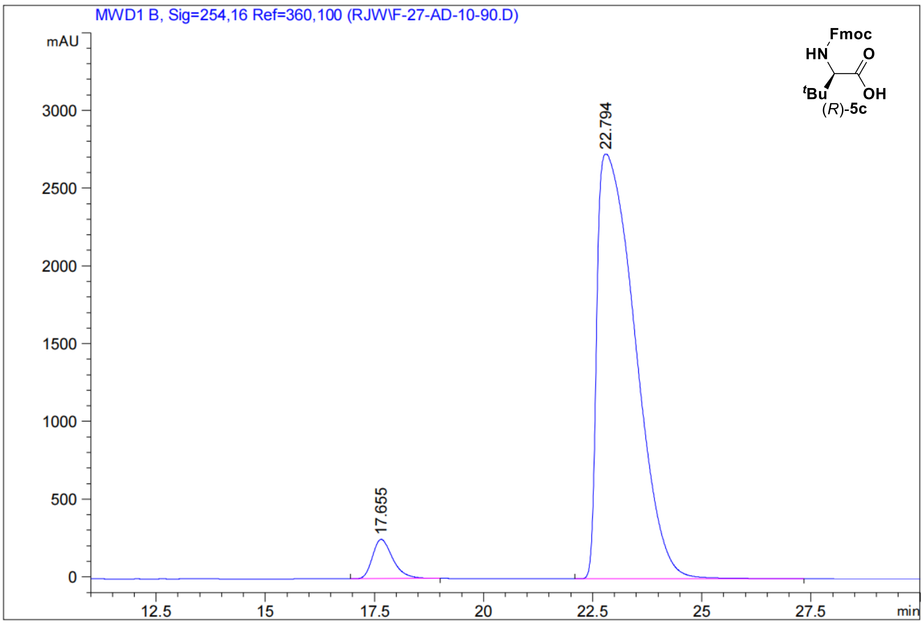

RT [min]	Type	Width [min]	Area	Height	Area%
17.655	BB	0.4950	8227.82422	253.29259	4.7551
22.794	BB	0.7678	1.64805e5	2732.02588	95.2449

HPLC chromatogram of racemic **5d**

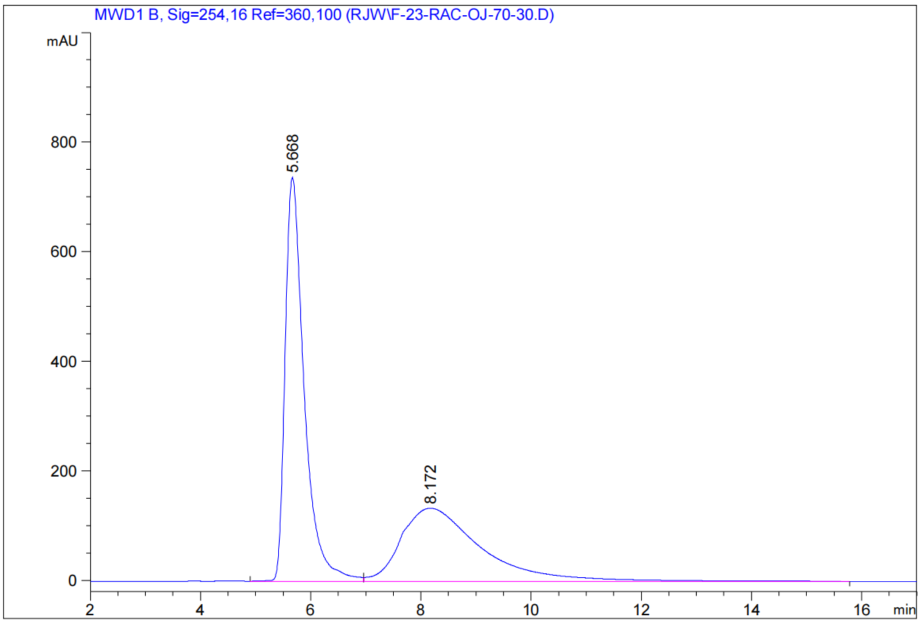

RT [min]	Type	Width [min]	Area	Height	Area%
5.668	VV	0.3631	1.77525e4	736.73999	50.8527
8.172	VB	1.4147	1.71572e4	133.35808	49.1473

HPLC chromatogram of enantiopure **5d**

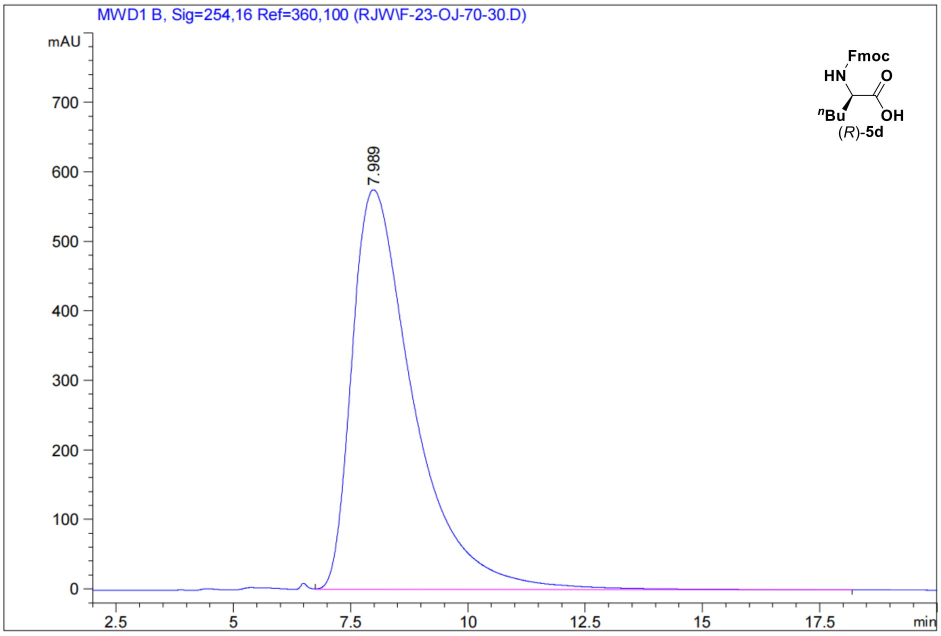

RT [min]	Type	Width [min]	Area	Height	Area%
7.989	VB	1.3458	5.26165e4	574.88782	100.0000

HPLC chromatogram of racemic **5e**

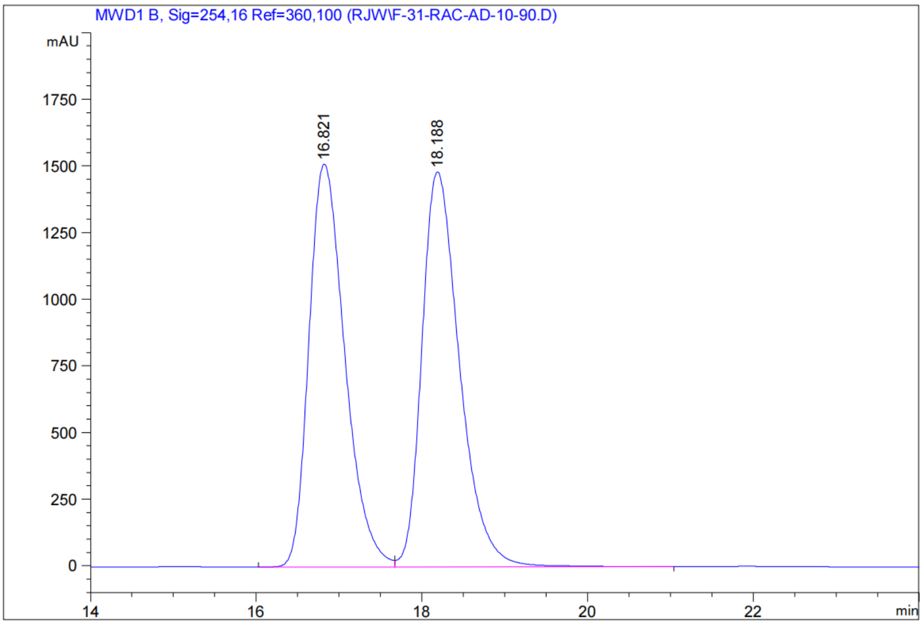

RT [min]	Type	Width [min]	Area	Height	Area%
16.821	BV	0.4649	4.54409e4	1510.95959	49.2692
18.188	VB	0.4846	4.67889e4	1480.92371	50.7308

HPLC chromatogram of enantiopure **5e**

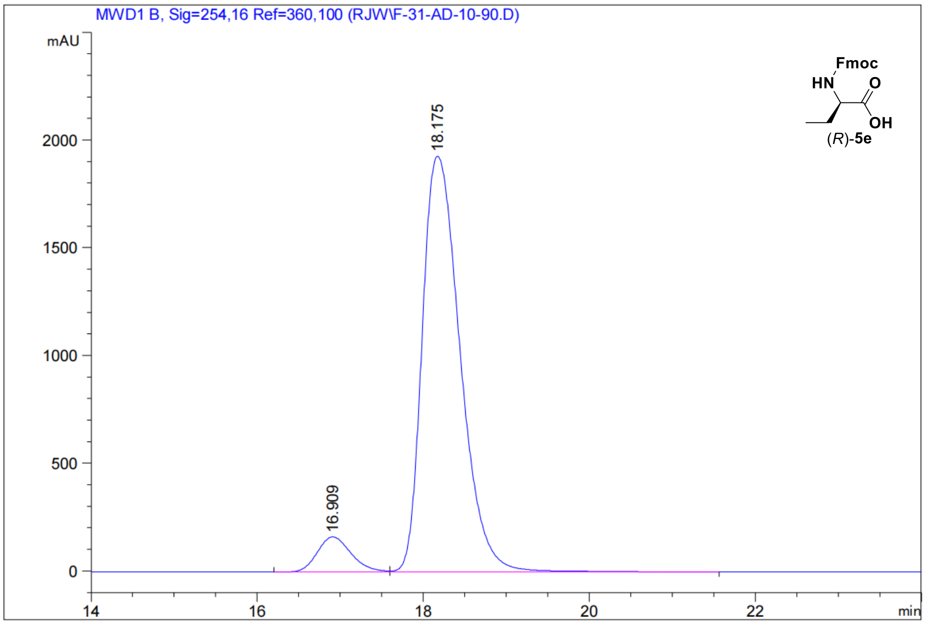

RT [min]	Type	Width [min]	Area	Height	Area%
16.909	BV	0.4394	4625.20068	162.82468	7.0353
18.175	VB	0.4954	6.11174e4	1930.01538	92.9647

HPLC chromatogram of racemic **5f**

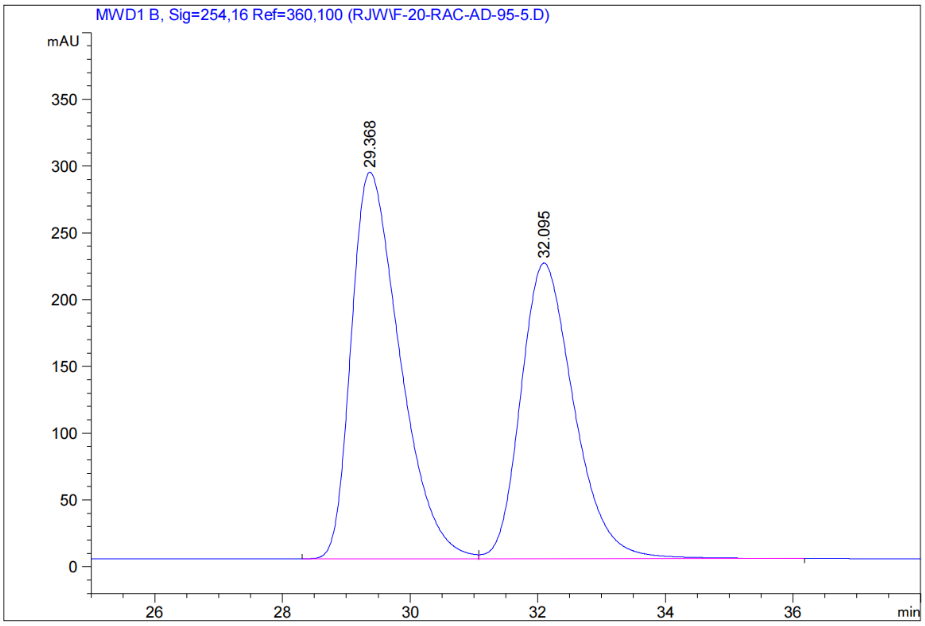

RT [min]	Type	Width [min]	Area	Height	Area%
29.368	BV	0.8087	1.53508e4	289.47513	54.5381
32.095	VB	0.8946	1.27961e4	221.30856	45.4619

HPLC chromatogram of enantiopure **5f**

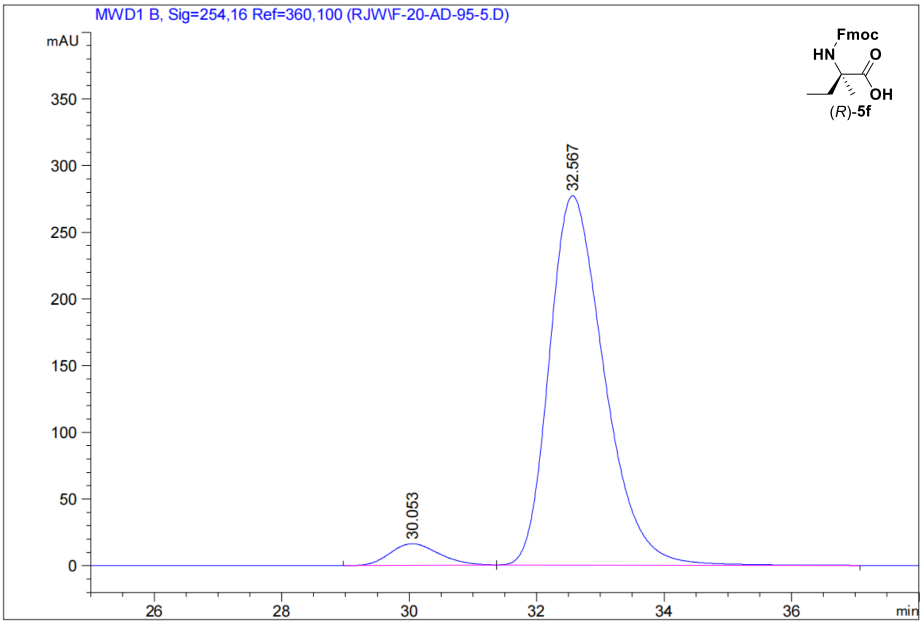

RT [min]	Type	Width [min]	Area	Height	Area%
30.053	BB	0.7918	861.34674	16.21384	4.9452
32.567	BB	0.9255	1.65565e4	276.92175	95.0548

HPLC chromatogram of racemic **5g**

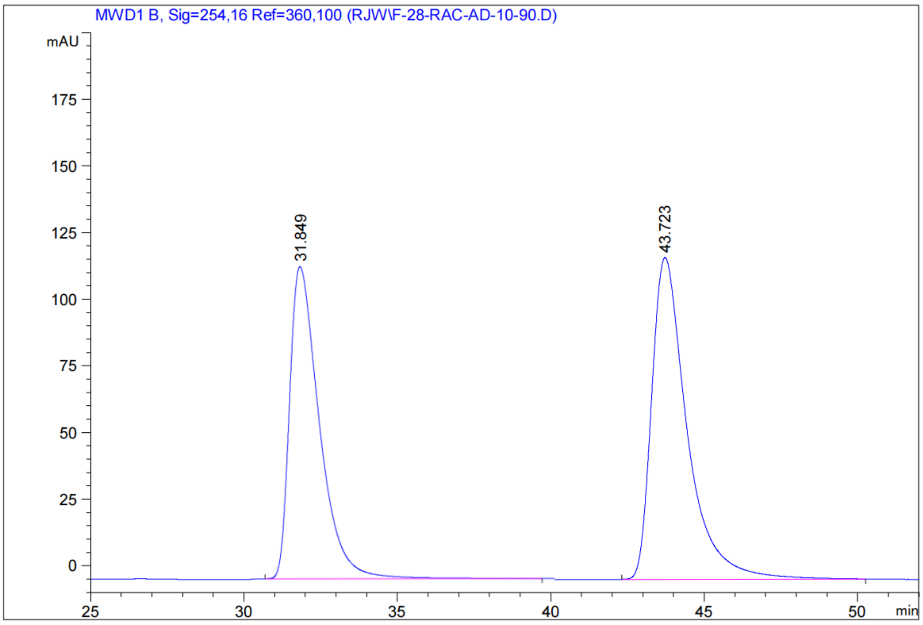

RT [min]	Type	Width [min]	Area	Height	Area%
31.849	BB	0.9287	8866.90373	117.43515	47.7797
43.723	BB	1.1924	9690.98535	120.73658	52.2203

HPLC chromatogram of enantiopure **5g**

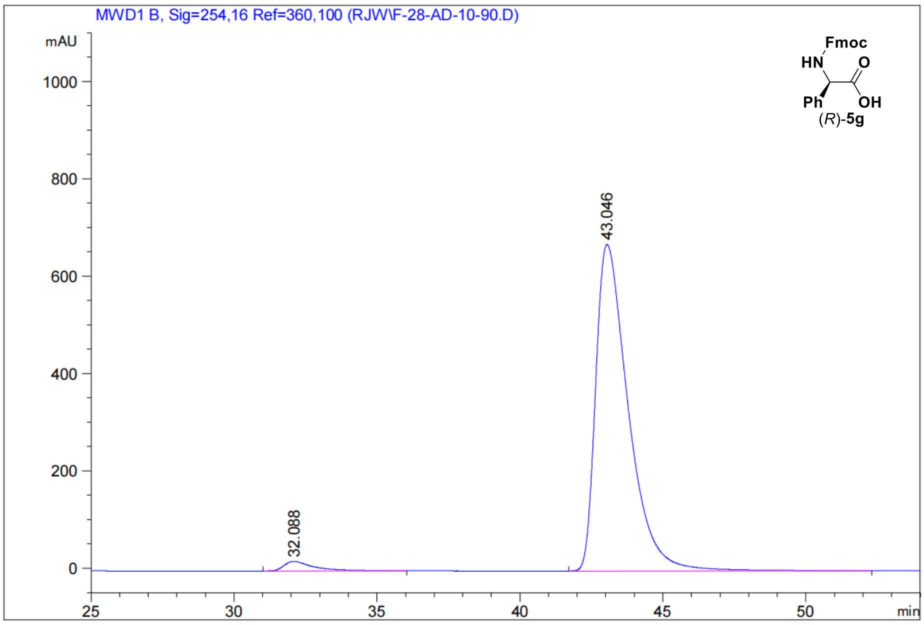

RT [min]	Type	Width [min]	Area	Height	Area%
32.088	BB	1.0157	1430.75244	19.50683	2.6252
43.046	BB	1.1815	5.30708e4	670.47424	97.3748

HPLC chromatogram of racemic **5h**

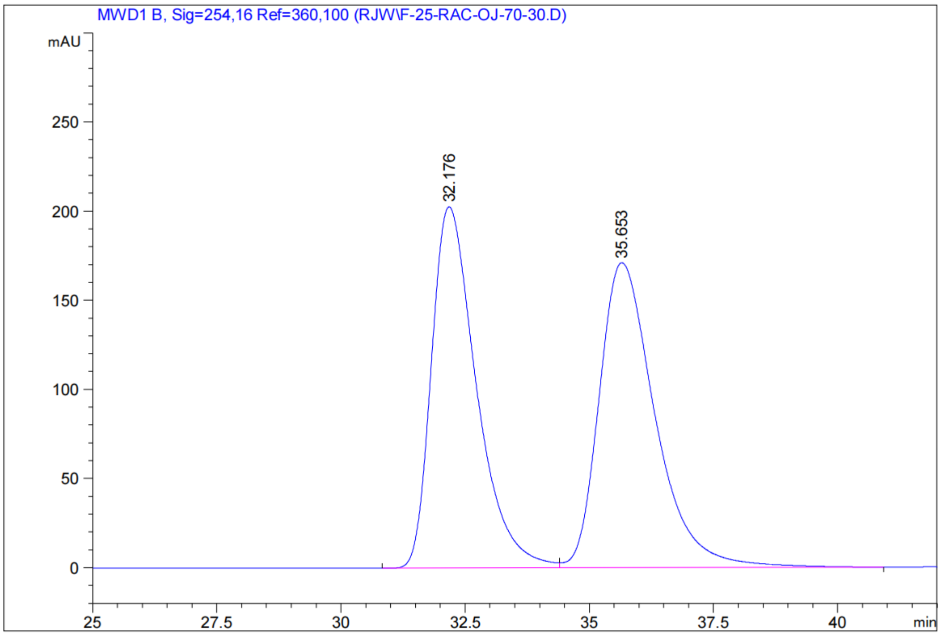

RT [min]	Type	Width [min]	Area	Height	Area%
32.176	BV	0.9449	1.27289e4	202.61510	48.7118
35.653	VB	1.1898	1.34021e4	171.10147	51.2882

HPLC chromatogram of enantiopure **5h**

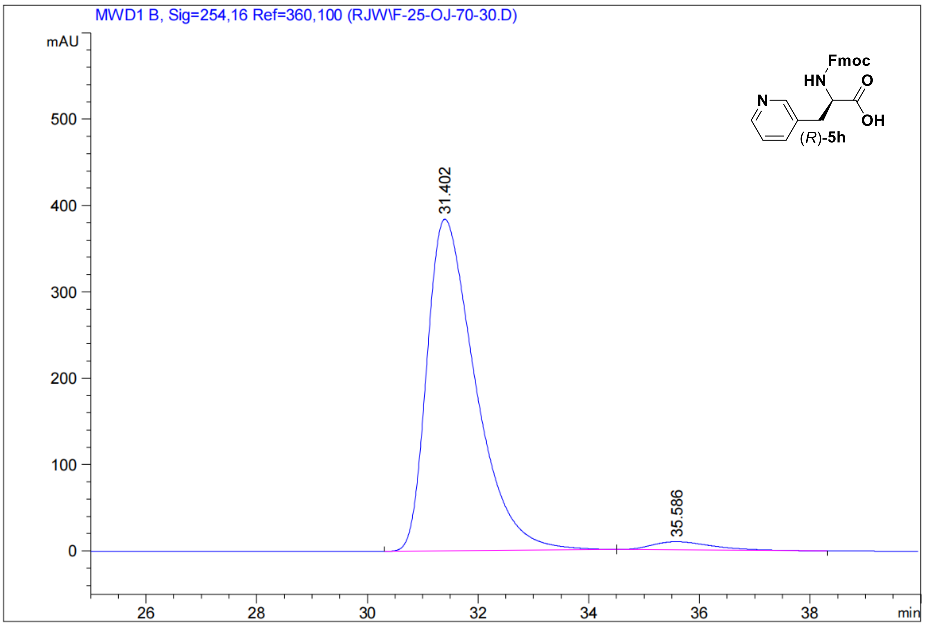

RT [min]	Type	Width [min]	Area	Height	Area%
31.402	BB	0.9301	2.33113e4	384.08240	96.0014
35.586	BB	1.0004	970.95005	9.52778	3.9986

HPLC chromatogram of racemic **5i**

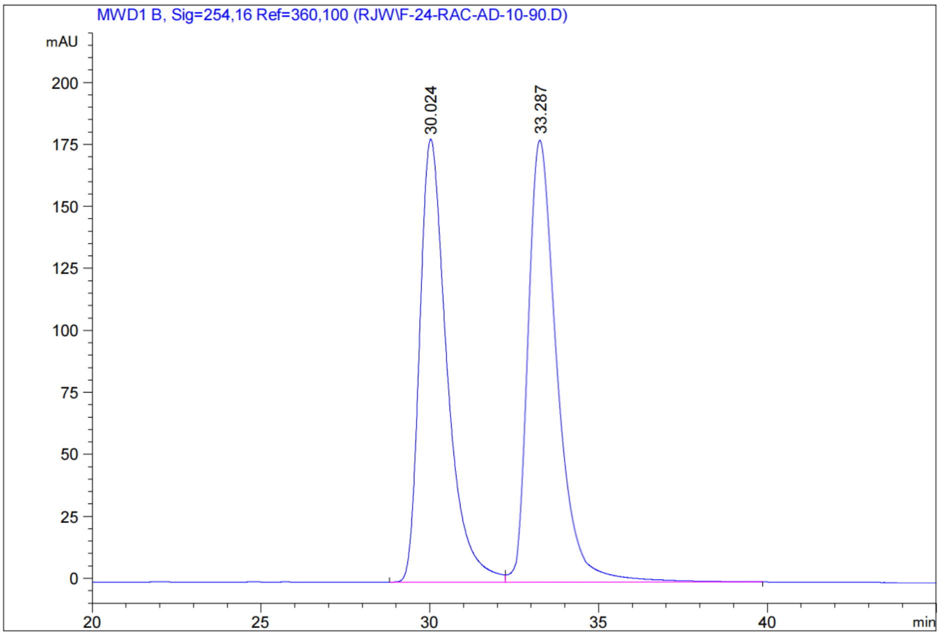

RT [min]	Type	Width [min]	Area	Height	Area%
30.024	BV	0.8472	1.00624e4	178.63223	50.0647
33.287	VB	1.0733	1.00364e4	177.67546	49.9353

HPLC chromatogram of enantiopure **5i**

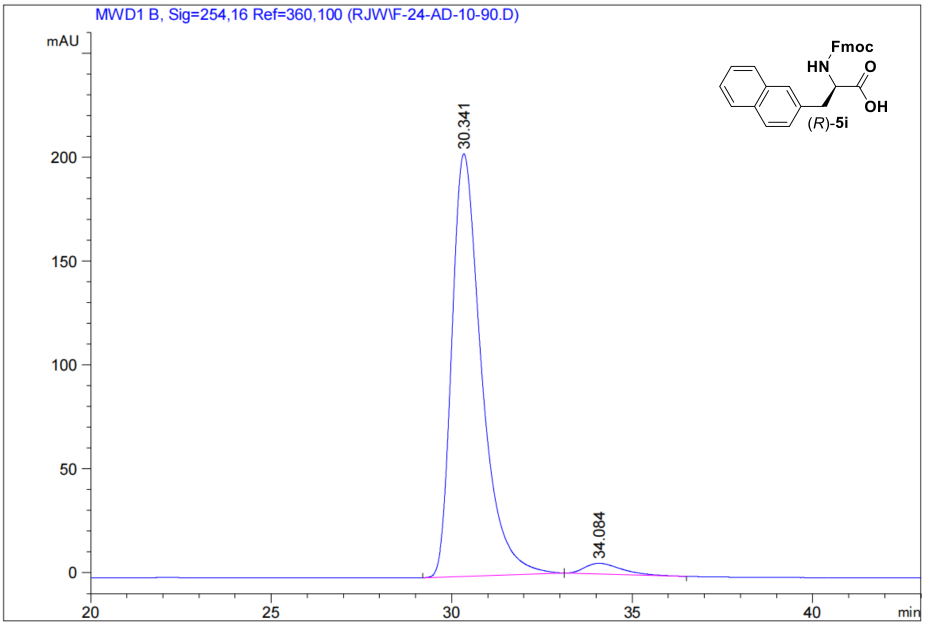

RT [min]	Type	Width [min]	Area	Height	Area%
30.341	BB	0.8567	1.15483e4	203.29462	96.9530
34.084	BB	0.9211	362.93527	5.15706	3.0470

HPLC chromatogram of racemic **5j**

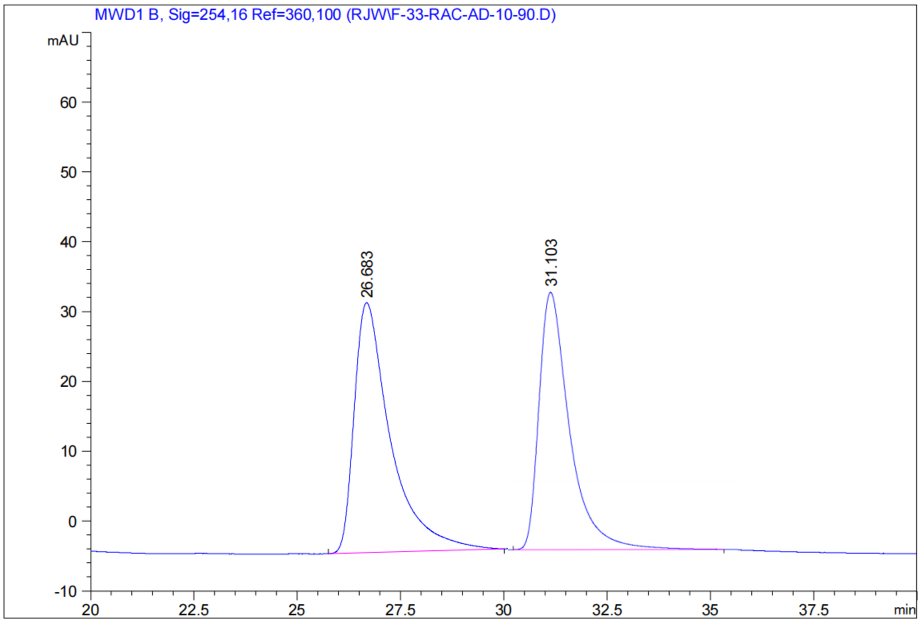

RT [min]	Type	Width [min]	Area	Height	Area%
26.683	BB	0.8956	2196.23975	35.80371	49.3527
31.103	MM R	0.9909	2253.85083	37.91086	50.6473

HPLC chromatogram of enantiopure **5j**

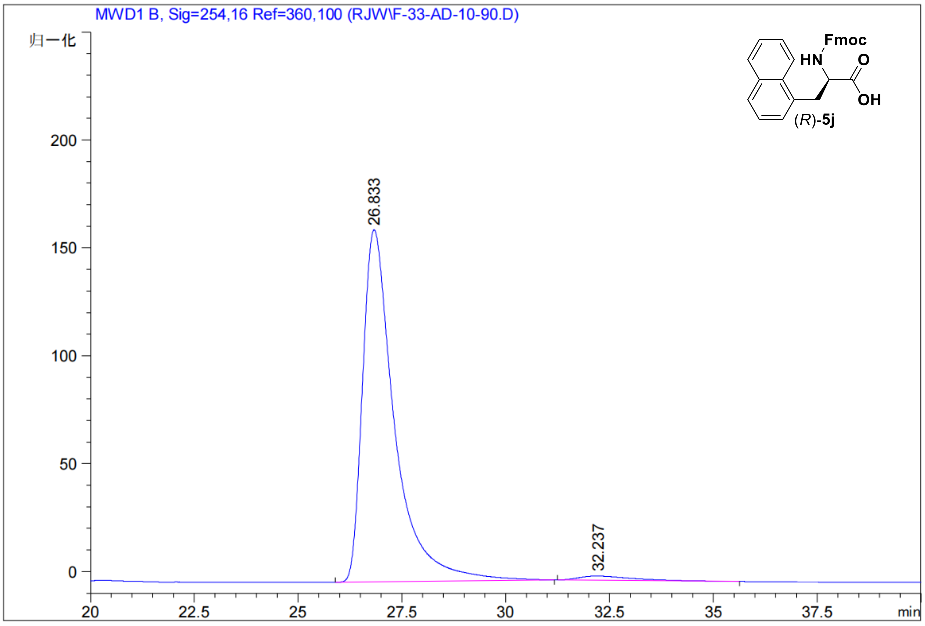

RT [min]	Type	Width [min]	Area	Height	Area%
26.833	BB	0.7939	8778.51172	163.11127	98.0287
32.237	BB	1.0535	176.52769	1.96614	1.9713

HPLC chromatogram of racemic **5k**

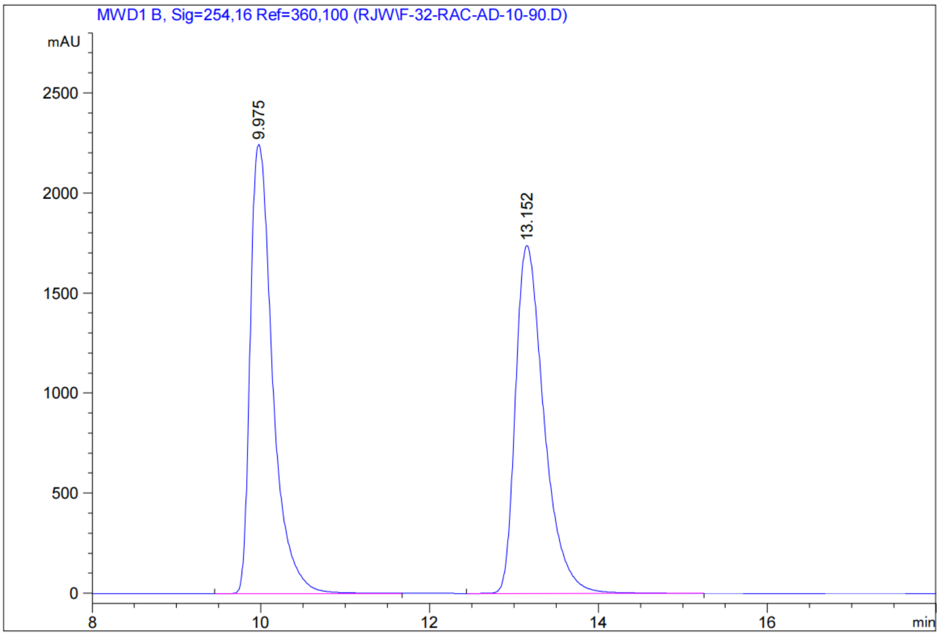

RT [min]	Type	Width [min]	Area	Height	Area%
9.975	BV	0.2829	4.13198e4	2243.33154	50.2782
13.152	BB	0.3584	4.08626e4	1737.35583	49.7218

HPLC chromatogram of enantiopure **5k**

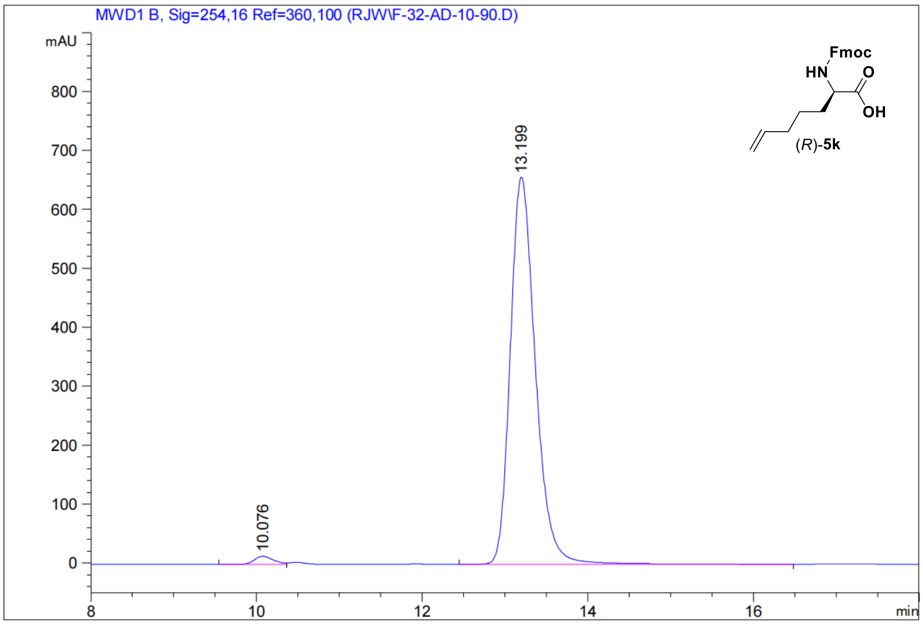

RT [min]	Type	Width [min]	Area	Height	Area%
10.076	BV	0.2455	219.35054	13.64923	1.5595
13.199	BB	0.3240	1.38463e4	656.67273	98.4405

HPLC chromatogram of racemic **5l**

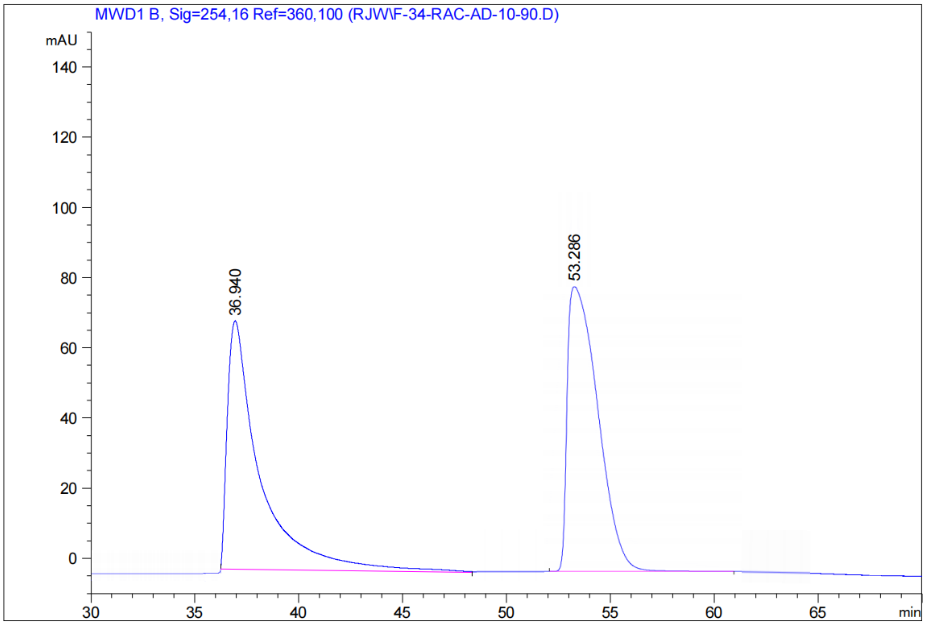

RT [min]	Type	Width [min]	Area	Height	Area%
36.940	MM R	1.8304	7772.31982	70.76884	50.2905
53.286	MM R	1.5139	7682.52979	84.57660	49.7095

HPLC chromatogram of enantiopure **5l**

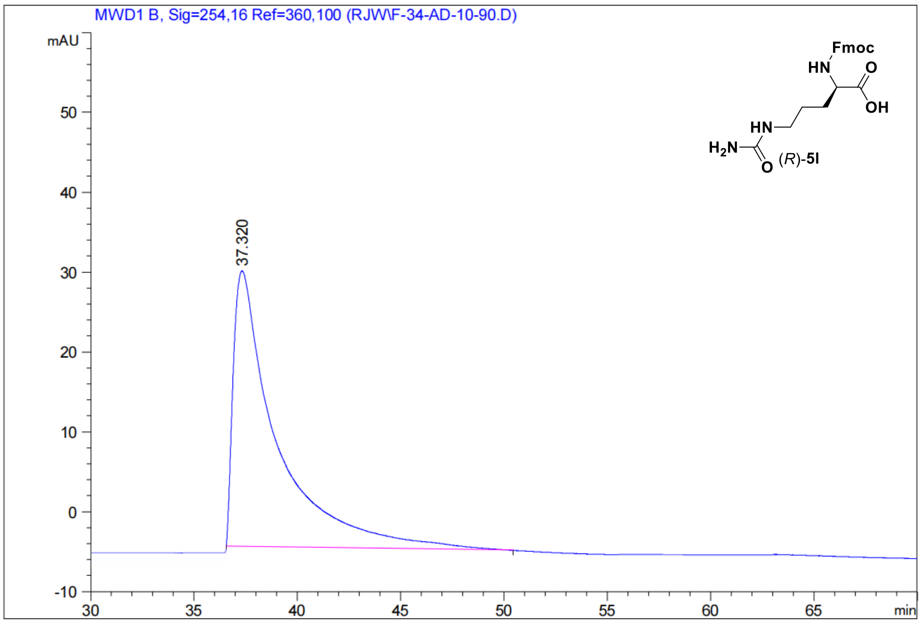

RT [min]	Type	Width [min]	Area	Height	Area%
37.320	MM R	2.4434	5051.74365	34.45790	100.0000

HPLC chromatogram of racemic **5m**

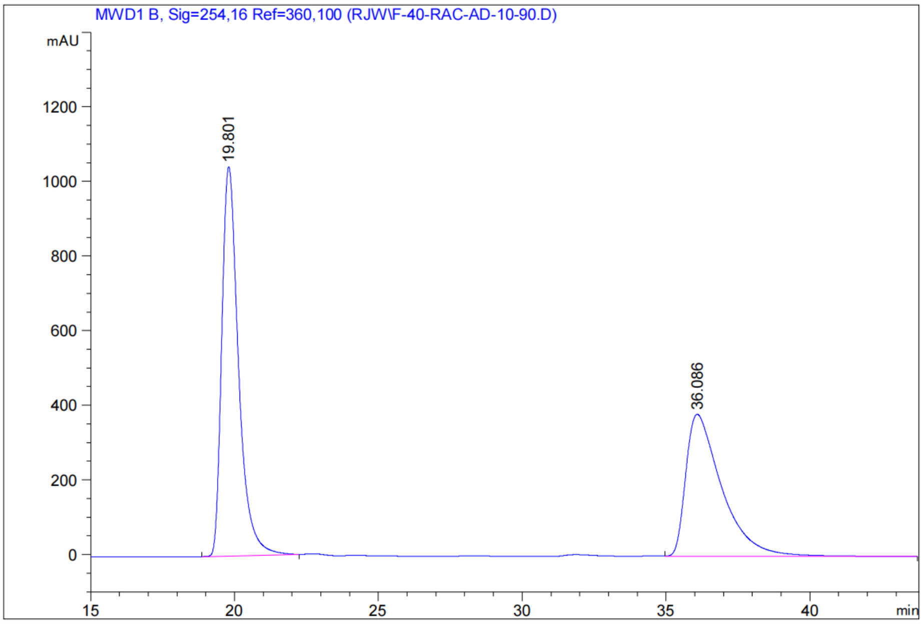

RT [min]	Type	Width [min]	Area	Height	Area%
19.801	BB	0.6185	4.21038e4	1043.96899	52.9722
36.086	VBA	1.3085	3.73790e4	380.84705	47.0278

HPLC chromatogram of enantiopure **5m**

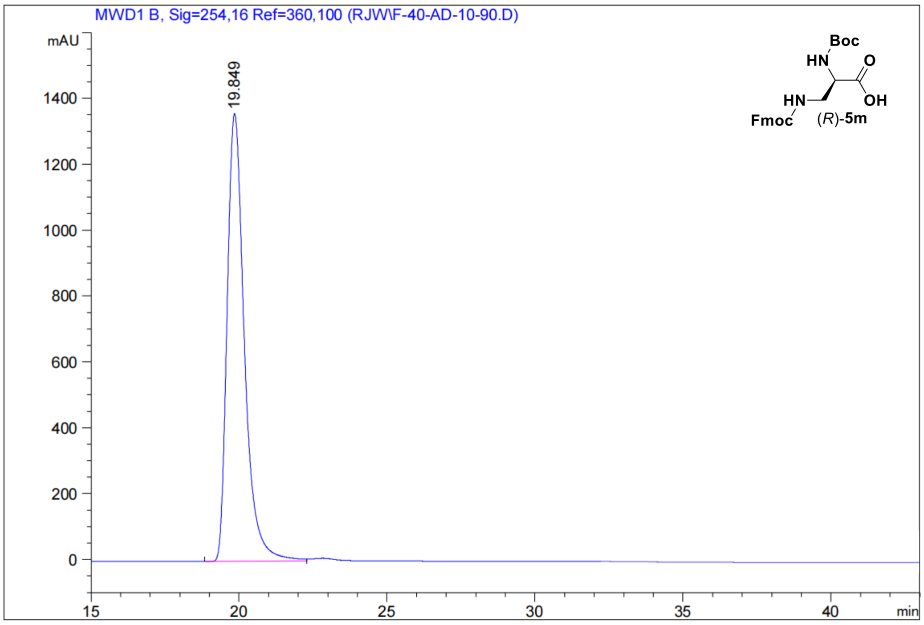

RT [min]	Type	Width [min]	Area	Height	Area%
19.849	BV	0.6211	5.50988e4	1358.70898	100.0000

HPLC chromatogram of racemic **5n**

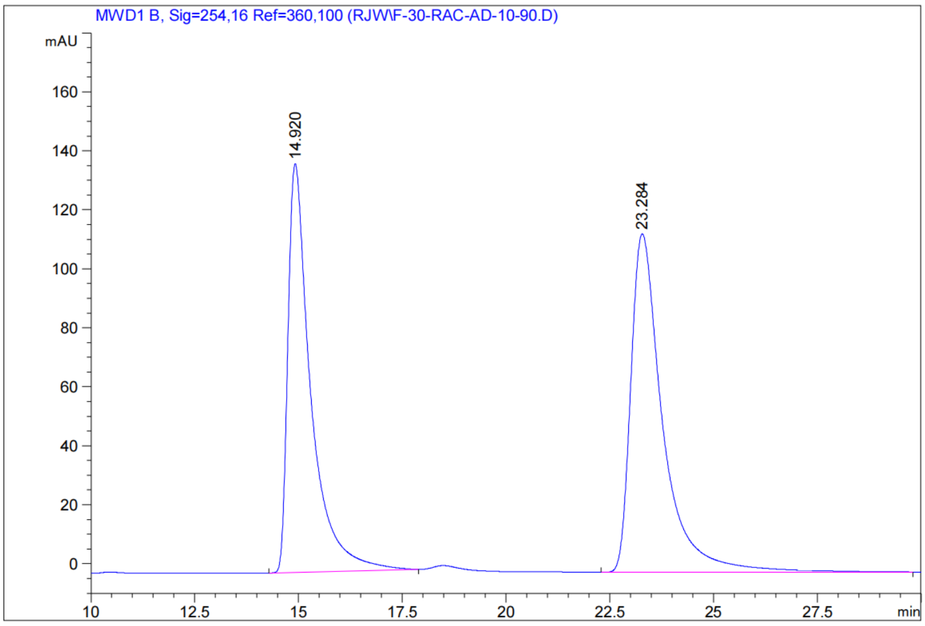

RT [min]	Type	Width [min]	Area	Height	Area%
14.920	BB	0.5552	5312.44434	138.60599	46.3232
23.284	BB	0.7918	6155.77686	114.75441	53.6768

HPLC chromatogram of enantiopure **5n**

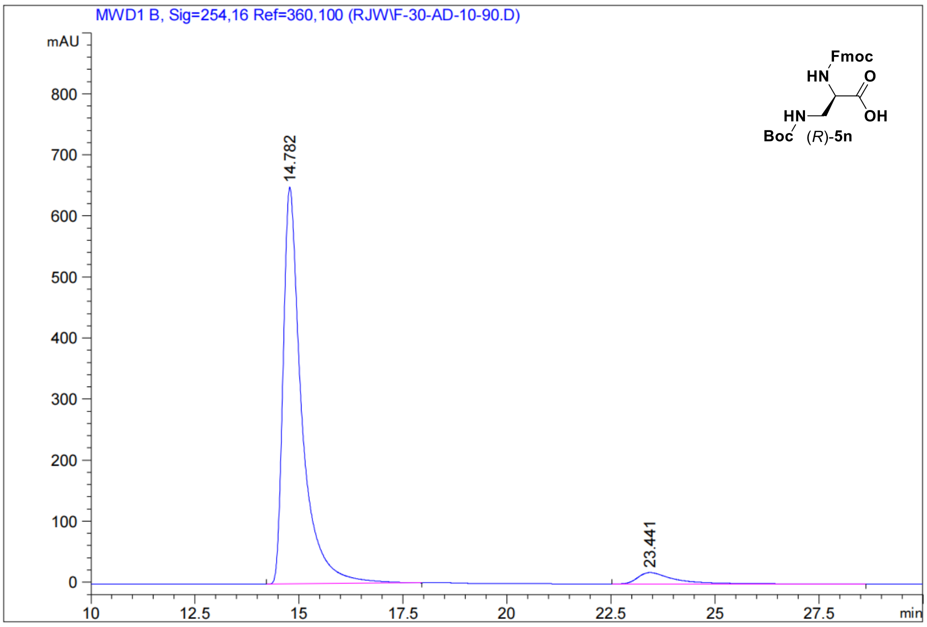

RT [min]	Type	Width [min]	Area	Height	Area%
14.782	BB	0.4527	2.01973e4	649.65570	94.4839
23.441	BB	0.8964	1179.15515	18.68021	5.5161

HPLC chromatogram of racemic **5o**

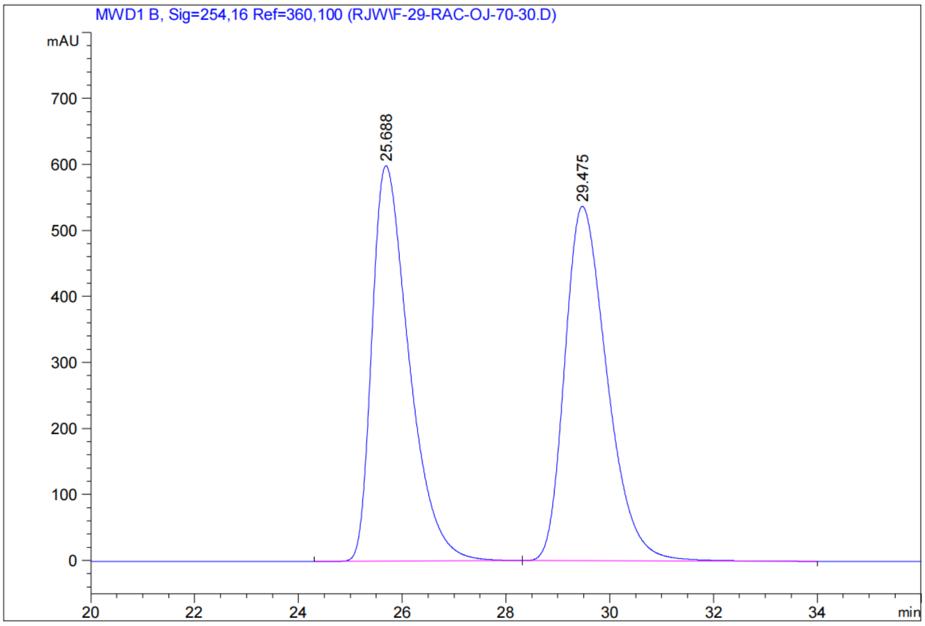

RT [min]	Type	Width [min]	Area	Height	Area%
25.688	BB	0.7494	2.97475e4	598.95367	49.4760
29.475	BB	0.8641	3.03777e4	536.87616	50.5240

HPLC chromatogram of enantiopure **5o**

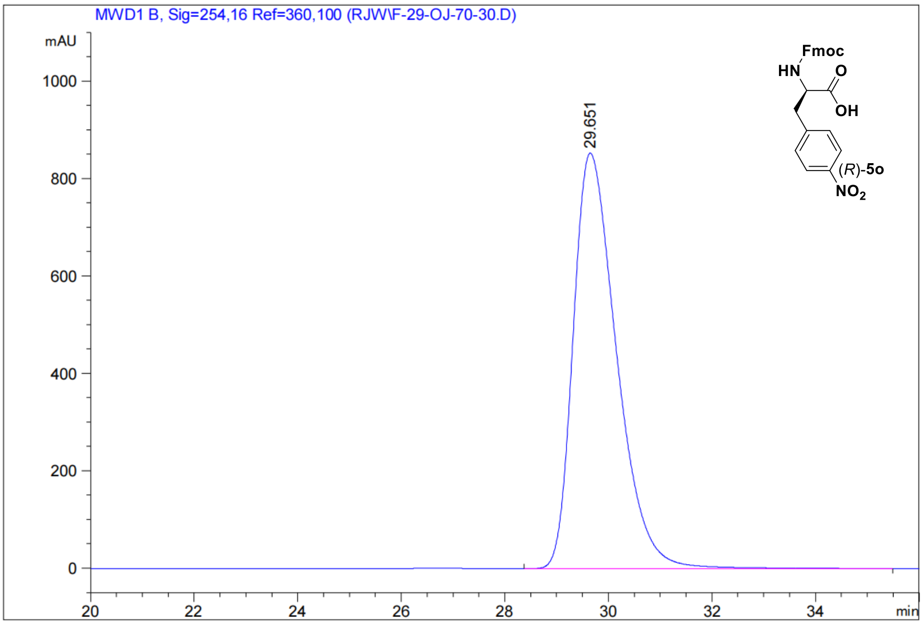

RT [min]	Type	Width [min]	Area	Height	Area%
29.651	BB	0.8916	4.95425e4	853.03564	100.0000

HPLC chromatogram of racemic **5p**

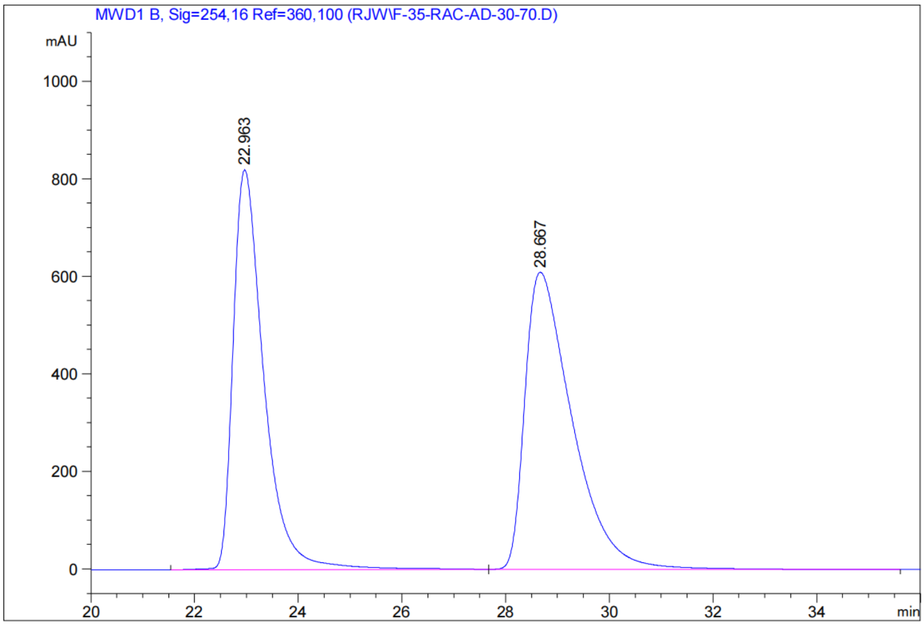

RT [min]	Type	Width [min]	Area	Height	Area%
22.963	BB	0.6221	3.37248e4	819.48053	46.6515
28.667	BB	0.9428	3.85662e4	609.05090	53.3485

HPLC chromatogram of enantiopure **5p**

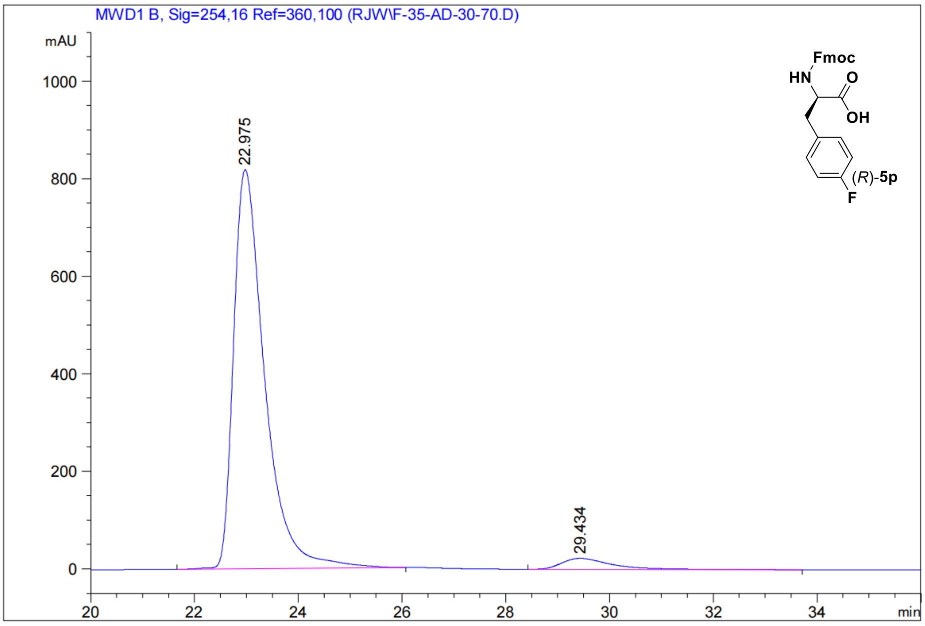

RT [min]	Type	Width [min]	Area	Height	Area%
22.975	BB	0.6269	3.40043e4	818.14954	95.7707
29.434	BB	0.9700	1501.65137	22.51691	4.2293

HPLC chromatogram of racemic **5q**

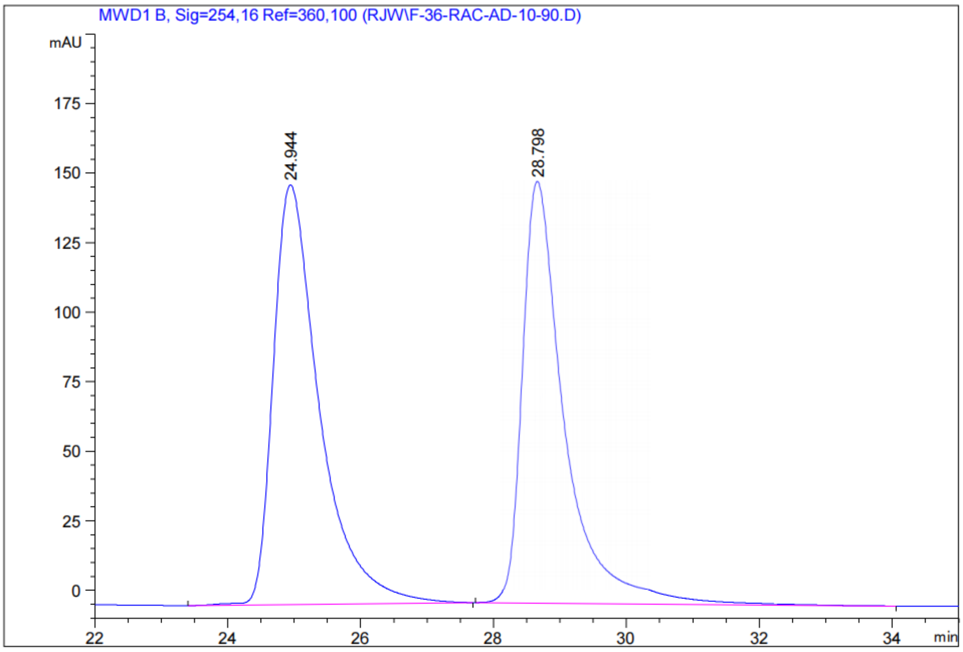

RT [min]	Type	Width [min]	Area	Height	Area%
24.944	BB	0.7201	7267.97803	150.84113	50.0493
28.798	BB	0.9422	7253.65970	156.00260	49.9507

HPLC chromatogram of enantiopure **5q**

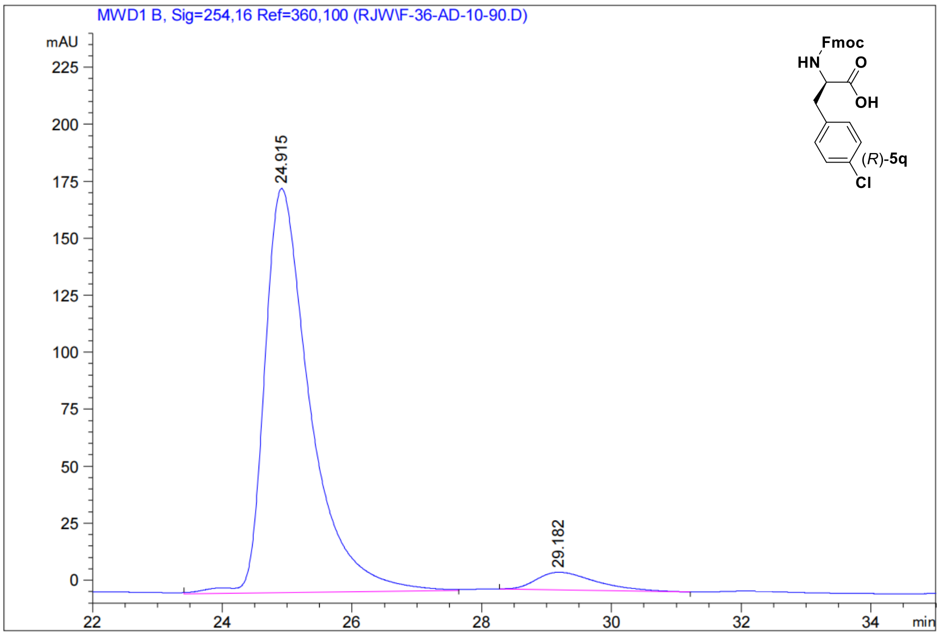

RT [min]	Type	Width [min]	Area	Height	Area%
24.915	MM R	0.8102	8617.54102	177.27788	94.6232
29.182	BB	0.7760	489.68106	7.69984	5.3768

HPLC chromatogram of racemic **5r**

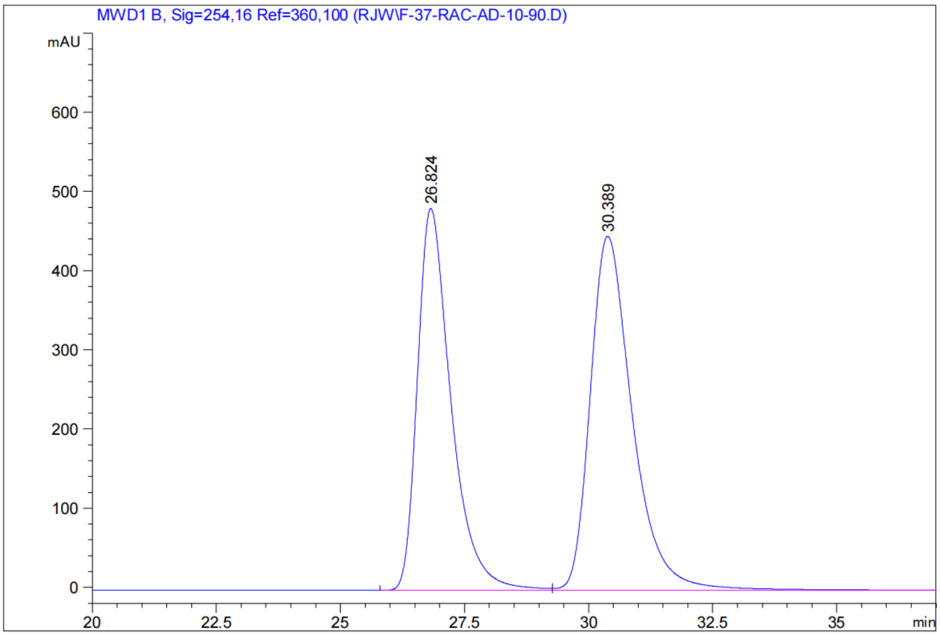

RT [min]	Type	Width [min]	Area	Height	Area%
26.824	BV	0.7075	2.27049e4	482.01990	46.2657
30.389	VBA	0.8903	2.63702e4	446.98337	53.7343

HPLC chromatogram of enantiopure **5r**

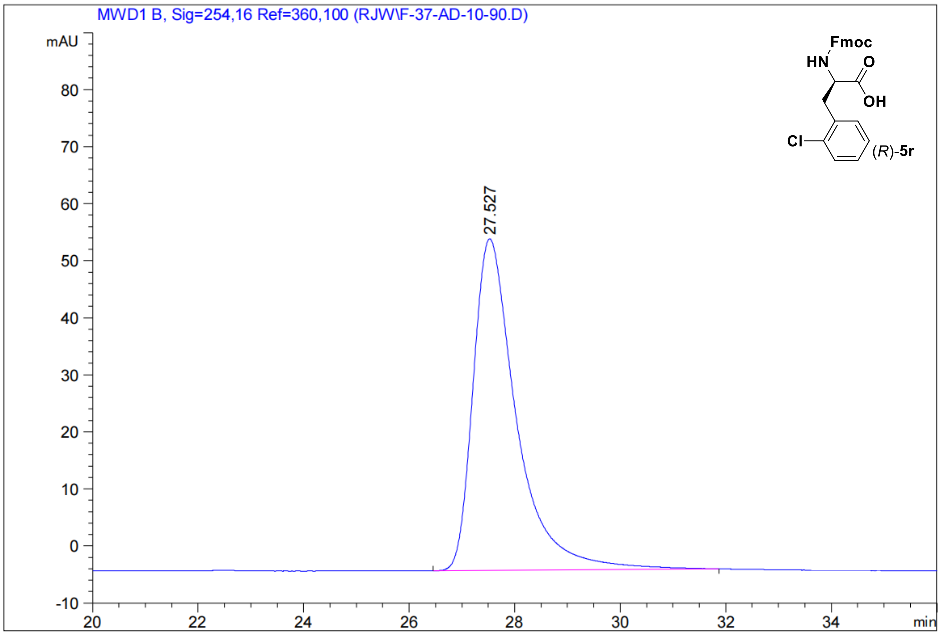

RT [min]	Type	Width [min]	Area	Height	Area%
27.527	BB	0.8543	3330.00317	58.13287	100.0000

HPLC chromatogram of racemic **5s**

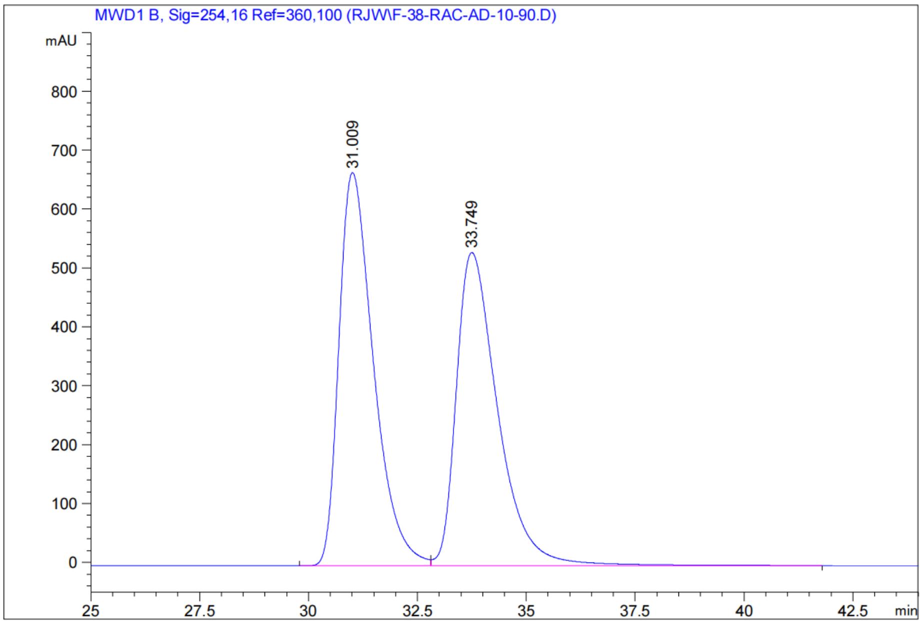

RT [min]	Type	Width [min]	Area	Height	Area%
31.009	BV	0.8393	3.68062e4	667.52710	51.1320
33.749	VB	0.9998	3.51765e4	531.70435	48.8680

HPLC chromatogram of enantiopure **5s**

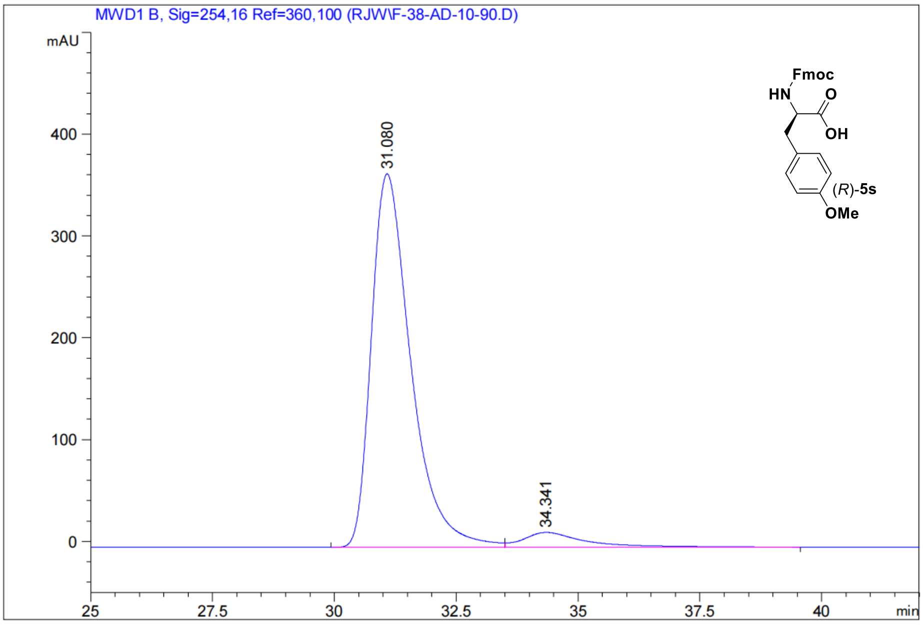

(*R*)-**5s**

RT [min]	Type	Width [min]	Area	Height	Area%
31.080	BV	0.8554	2.06647e4	366.73969	94.0976
34.341	VB	1.1838	1296.22156	14.54433	5.9024

HPLC chromatogram of racemic **5t**

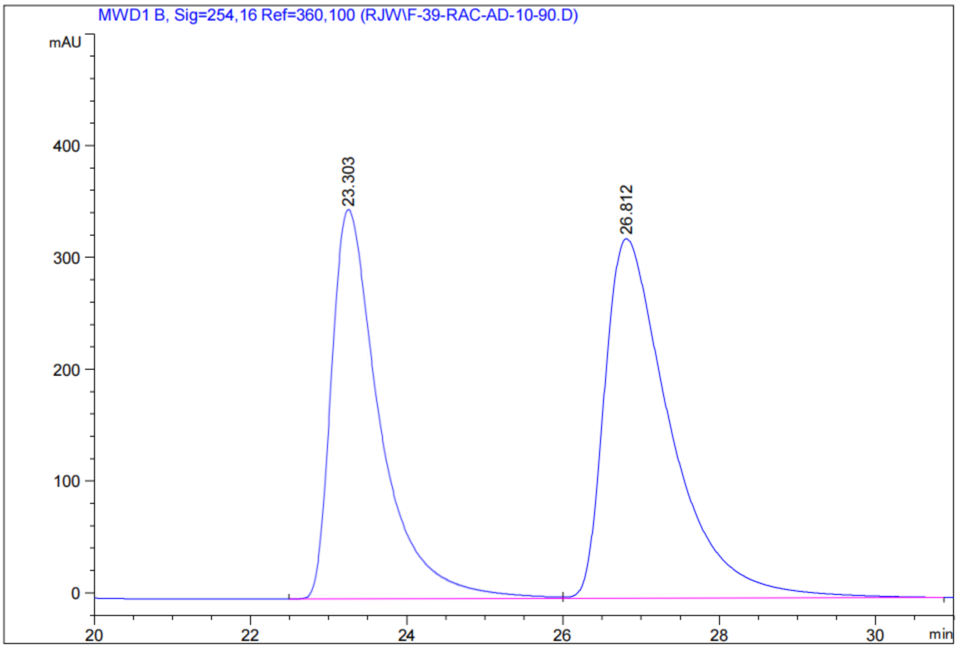

RT [min]	Type	Width [min]	Area	Height	Area%
23.303	BV	0.6697	1.66605e4	346.63705	47.0452
26.812	VB	0.8687	1.87533e4	321.41055	52.9548

HPLC chromatogram of enantiopure **5t**

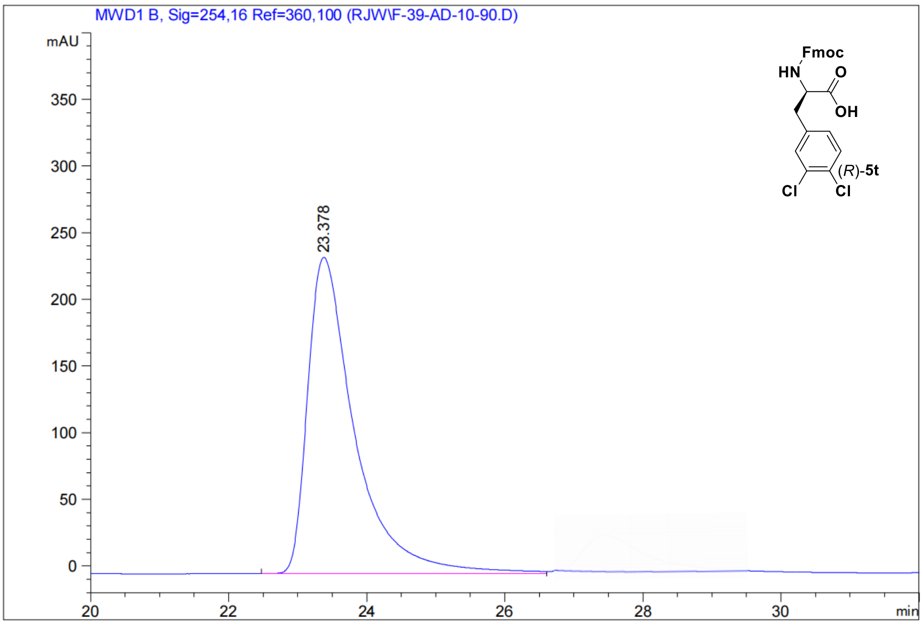

RT [min]	Type	Width [min]	Area	Height	Area%
23.378	BV	0.7009	1.12410e4	237.20477	100.0000

HPLC chromatogram of racemic **5u**

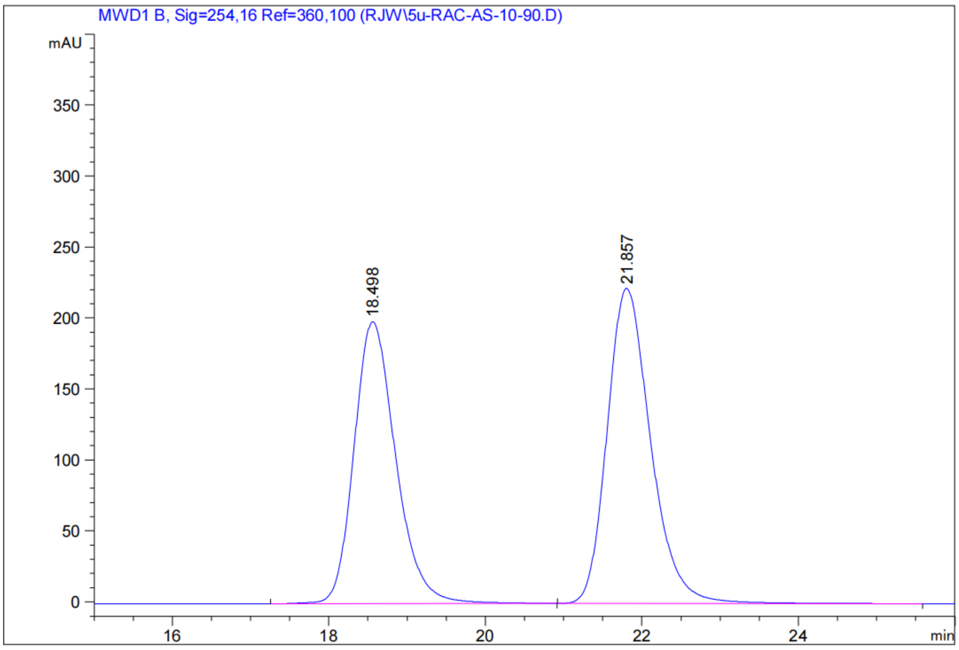

RT [min]	Type	Width [min]	Area	Height	Area%
18.498	BB	0.5696	7671.61495	199.00247	47.2329
21.857	BB	0.6021	8570.48526	222.08895	52.7671

HPLC chromatogram of enantiopure **5u**

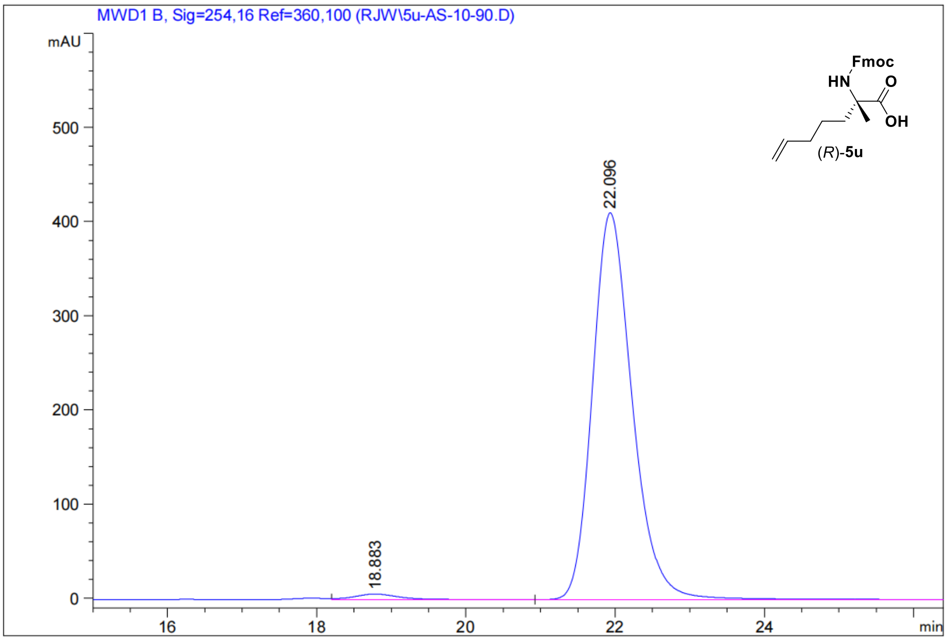

RT [min]	Type	Width [min]	Area	Height	Area%
18.883	VB	0.6011	234.68959	5.49764	1.5337
22.096	BBA	0.5685	1.50675e4	411.10648	98.4663

HPLC chromatogram of racemic **5v**

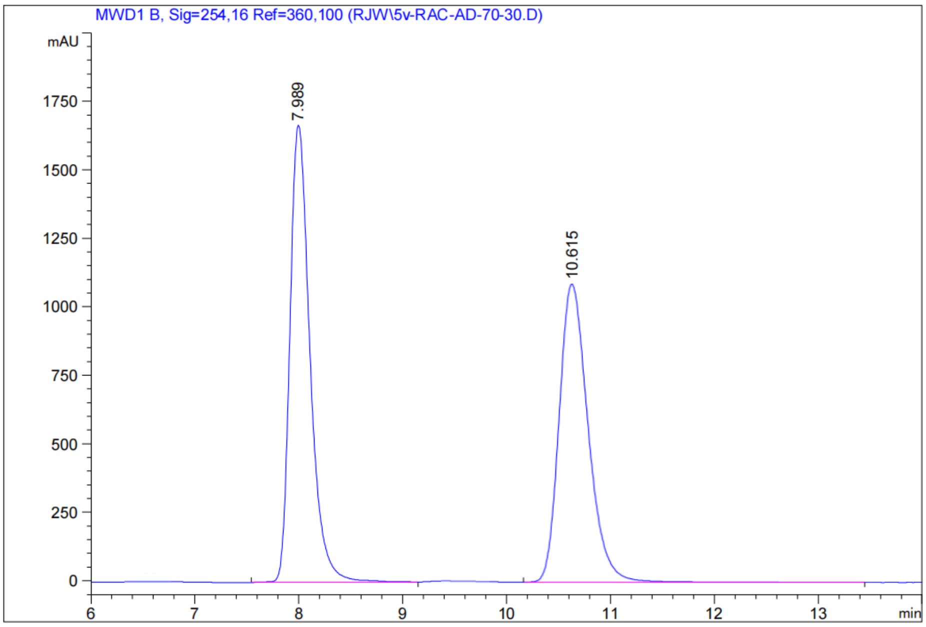

RT [min]	Type	Width [min]	Area	Height	Area%
7.989	BB	0.2059	2.21241e4	1668.00325	51.3189
10.615	BB	0.3041	2.09869e4	1097.89364	48.6811

HPLC chromatogram of enantiopure **5v**

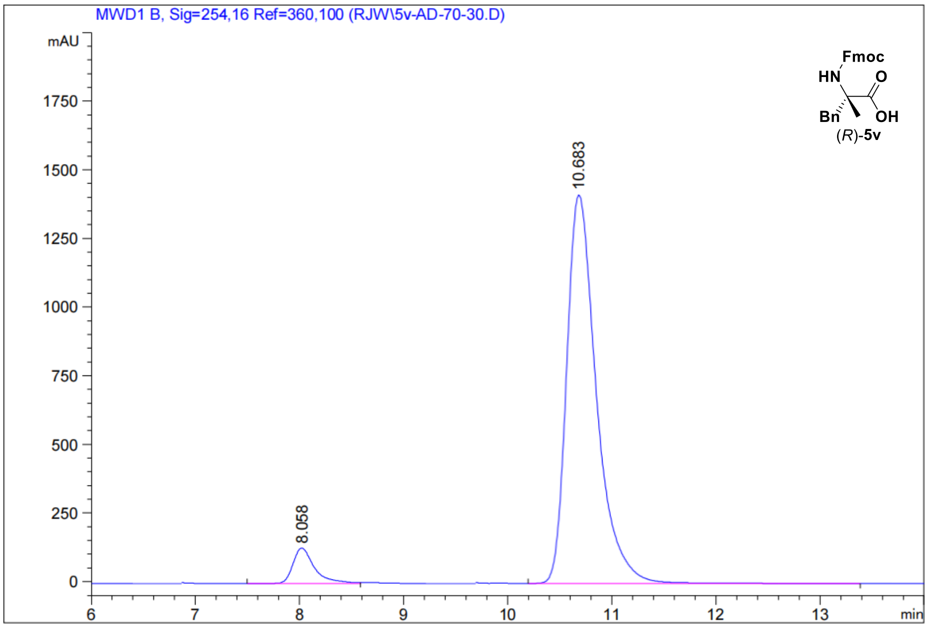

RT [min]	Type	Width [min]	Area	Height	Area%
8.058	BV	0.2097	1786.14856	129.02547	5.9564
10.683	BB	0.3105	2.82009e4	1411.46371	94.0436

HPLC chromatogram of racemic **S4b**

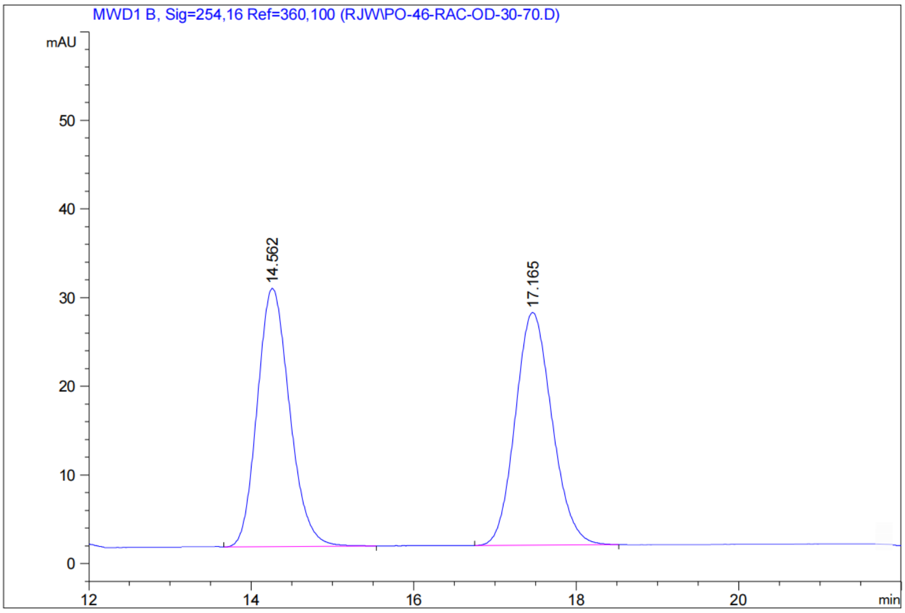

RT [min]	Type	Width [min]	Area	Height	Area%
14.562	BB	0.4286	805.93860	29.14602	49.3630
17.165	BB	0.4891	826.74017	26.27270	50.6370

HPLC chromatogram of enantiopure **S4b**

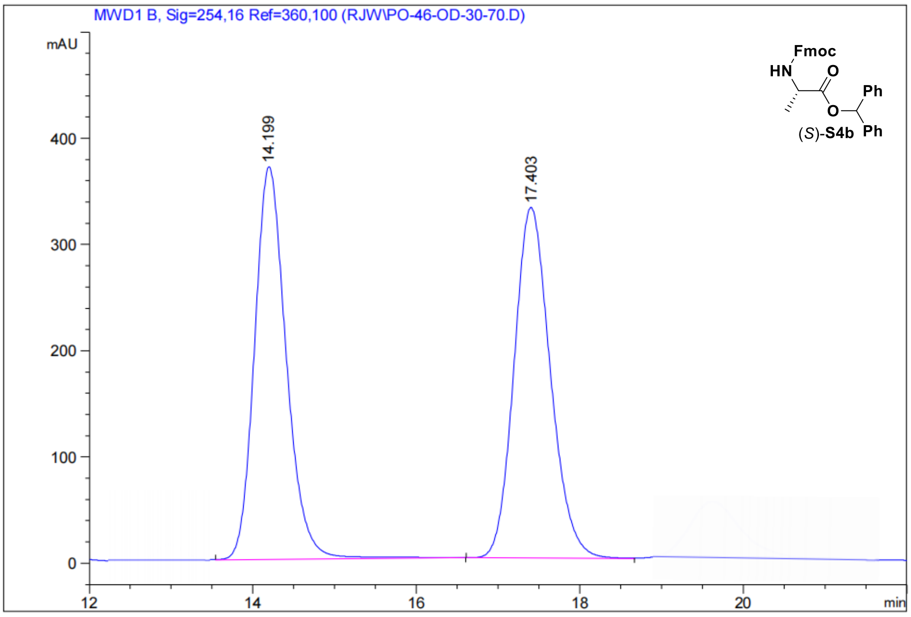

RT [min]	Type	Width [min]	Area	Height	Area%
14.199	VB	0.4153	9992.12988	369.77554	49.4783
17.403	BV	0.4765	1.02028e4	330.16367	50.5217

6. Quantum chemical calculations

All density functional theory (DFT)[1-2] calculations were carried out using the Gaussian 16 program[3]. Geometries of intermediates and transition states were optimized using the dispersion-corrected B3LYP functional[4-5] with basis set of 6-311G(d,p)[6] for all atoms. The Grimme's DFT-D3 method was applied to correct the van der Waals interactions for all molecules. Vibrational frequency calculations were performed for all stationary points to confirm if each optimized structure is a local minimum or a transition state structure. All optimized transition state structures have only one imaginary (negative) frequency, and all minima (reactants, products, and intermediates) have no imaginary frequencies. Solvation energy corrections were calculated using the SMD model with chloroform as the solvent.[7]

Cartesian coordinate for theoretical calculation

(*S*)-1a

C	1.03778	-0.27307	0.10565
C	1.69767	1.09584	0.09337
C	3.10015	0.96299	0.10566
C	3.43516	-0.467	0.14918
C	2.23619	-1.2061	0.16766
C	2.26479	-2.59468	0.22776
C	3.50173	-3.24668	0.26553
C	4.69218	-2.51397	0.24426
C	4.6682	-1.11946	0.18813
C	3.91967	2.0931	0.09017
C	3.32647	3.35598	0.06602
C	1.935	3.48837	0.05894
C	1.11272	2.35709	0.07418
H	1.34346	-3.17014	0.25209
H	3.5372	-4.33067	0.31329
H	5.64504	-3.03451	0.27361
H	5.59821	-0.55837	0.17635
H	5.00232	1.99832	0.10175
H	3.95235	4.24399	0.05515
H	1.48616	4.47745	0.04363
H	0.03281	2.46281	0.0696
C	0.16322	-0.57132	-1.12246

O	-0.99019	0.28251	-1.15671
C	-2.15817	-0.24992	-0.72023
O	-2.30772	-1.38915	-0.32512
N	-3.174	0.63497	-0.92189
C	-4.51951	0.3598	-0.45492
C	-5.20594	-0.77029	-1.22835
C	-4.50206	0.17133	1.07409
O	-3.71897	0.70104	1.81727
O	-5.48072	-0.65489	1.49528
H	0.39793	-0.39729	0.98867
H	-0.17038	-1.60778	-1.10229
H	0.71024	-0.37764	-2.04712
H	-2.92684	1.56827	-1.20894
H	-5.08623	1.28386	-0.61536
H	-6.23146	-0.90647	-0.87942
H	-4.66324	-1.70778	-1.10076
H	-5.22522	-0.51701	-2.29181
H	-5.43499	-0.62005	2.47103

(*R*)-1a

C	0.95857	-0.48302	0.05211
C	1.28738	0.9977	-0.05005
C	2.68342	1.19258	0.01253
C	3.33133	-0.11244	0.20072
C	2.33138	-1.10558	0.25697
C	2.67152	-2.44011	0.45006
C	4.02186	-2.7837	0.58819
C	5.01415	-1.79953	0.53066
C	4.67713	-0.45749	0.33835
C	3.22591	2.47617	-0.07405
C	2.36139	3.56369	-0.21497
C	0.97624	3.37132	-0.25716
C	0.42852	2.08581	-0.17264
H	1.90412	-3.20887	0.50343
H	4.30036	-3.82238	0.74453

H	6.05816	-2.08119	0.64046
H	5.45363	0.30238	0.29987
H	4.30081	2.63171	-0.02505
H	2.76809	4.56954	-0.28116
H	0.31677	4.23032	-0.34975
H	-0.64796	1.94532	-0.18024
C	0.30463	-1.06847	-1.2141
O	-0.95892	-0.45481	-1.53384
C	-2.02853	-0.87793	-0.81339
O	-1.98714	-1.7142	0.07055
N	-3.16259	-0.24322	-1.26394
C	-4.34979	-0.17807	-0.42402
C	-4.03872	0.56311	0.88643
O	-3.04501	1.22175	1.09492
O	-5.04627	0.45305	1.78072
H	0.2889	-0.69064	0.8959
H	0.15592	-2.14459	-1.0947
H	0.92968	-0.8784	-2.089
H	-2.97341	0.56944	-1.83597
H	-4.77778	0.97743	2.5592
C	-5.34562	0.72816	-1.17146
H	-5.41707	0.41415	-2.19185
H	-6.30785	0.65861	-0.70867
H	-5.00384	1.74139	-1.1332
H	-4.7608	-1.1491	-0.24216

(*S*)-2a

C	-2.36733	-0.56455	-0.24795
C	-1.08173	-0.07125	0.41106
C	-0.58536	1.30201	-0.10666
C	0.94443	1.23441	0.02981
C	1.26438	-0.26411	-0.02634
N	0.06774	-0.92354	0.11523
O	2.35845	-0.78463	-0.15366
H	-2.66033	-1.55029	0.13273

H	-1.24145	-0.01757	1.50104
H	-0.86359	1.40479	-1.16239
H	-1.04218	2.13125	0.4411
H	1.29074	1.61451	0.99966
H	1.49352	1.77463	-0.74549
H	0.06568	-1.92337	0.27062
H	-2.23967	-0.63981	-1.33346
O	-3.44162	0.33972	0.02242
H	-4.27747	-0.0846	-0.18472

$(COCl)_2$

C	-3.34651	0.15623	-0.00007
C	-2.83317	0.88218	1.25733
Cl	-5.07688	-0.00455	-0.27859
Cl	-1.1028	1.0404	1.53733
O	-2.52981	-0.32142	-0.82736
O	-3.64987	1.36165	2.08356

3a

C	-2.36711	-0.56458	-0.24882
C	-1.08191	-0.07167	0.41126
C	-0.58557	1.30194	-0.10588
C	0.94429	1.23457	0.02898
C	1.26438	-0.26405	-0.02619
N	0.06791	-0.92362	0.11598
O	2.35853	-0.78443	-0.15357
H	-3.19026	0.1291	-0.04209
H	-1.24219	-0.01832	1.50115
H	-0.86501	1.40573	-1.16119
H	-1.04189	2.13074	0.44298
H	1.29194	1.61561	0.99797
H	1.49241	1.7739	-0.74761
H	0.06622	-1.92335	0.27207
H	-2.23845	-0.63965	-1.33424
P	-2.85463	-2.2005	0.38243

C	-2.39185	-2.33616	2.13738
C	-1.4967	-3.32424	2.54833
C	-2.93197	-1.45228	3.0715
C	-1.14233	-3.42877	3.89321
H	-1.07165	-4.02141	1.81176
C	-2.57684	-1.55609	4.41668
H	-3.63736	-0.67335	2.74775
C	-1.68228	-2.54421	4.82767
H	-0.43726	-4.20796	4.2173
H	-3.00254	-0.85879	5.1529
H	-1.40267	-2.62678	5.888
C	-2.00442	-3.49675	-0.57112
C	-1.648	-3.26484	-1.89991
C	-1.70904	-4.72197	0.02649
C	-0.99696	-4.25823	-2.63103
H	-1.8817	-2.29908	-2.371
C	-1.05692	-5.7154	-0.70442
H	-1.98972	-4.90485	1.07382
C	-0.70099	-5.4838	-2.03303
H	-0.71656	-4.07574	-3.67859
H	-0.82379	-6.68119	-0.23284
H	-0.18761	-6.26687	-2.60969
O	-4.29909	-2.36417	0.24595

CP

C	-2.43863	0.7259	1.66755
C	-2.83715	-0.55648	2.47488
C	-1.81086	-0.71906	3.63036
C	-1.99743	-2.17879	4.06222
C	-2.34107	-2.8471	2.7337
N	-2.85377	-1.90357	1.9085
O	-2.18698	-4.03618	2.43311
P	-2.78297	1.09015	-0.09414
Cl	-5.04779	2.61821	2.12997
H	-3.83012	-0.35459	2.89806

H	-2.95098	1.51661	2.22926
H	-1.3523	0.86249	1.70769
H	-1.95526	0.01665	4.42561
H	-0.7996	-0.59702	3.22452
H	-2.85406	-2.29076	4.73973
H	-1.12163	-2.63369	4.5291
H	-3.21599	-2.16448	0.97204
C	-2.65241	2.8483	-0.5161
C	-2.48761	3.17748	-1.87442
C	-2.80122	3.86321	0.44434
C	-2.45198	4.514	-2.26341
H	-2.39437	2.40037	-2.62746
C	-2.76685	5.19812	0.03828
H	-3.03105	3.59051	1.47134
C	-2.58982	5.52363	-1.30574
H	-2.32586	4.76626	-3.31238
H	-2.89968	5.9801	0.77946
H	-2.57204	6.56531	-1.61467
C	-4.21066	0.29022	-0.83855
C	-5.32952	-0.03508	-0.06191
C	-4.17185	-0.0305	-2.20711
C	-6.40585	-0.68593	-0.66208
H	-5.37265	0.25373	0.98289
C	-5.2504	-0.68829	-2.7884
H	-3.29829	0.19905	-2.80764
C	-6.3678	-1.01593	-2.01534
H	-7.27659	-0.93316	-0.06249
H	-5.22086	-0.94187	-3.84447
H	-7.21086	-1.52592	-2.47346
Cl	-1.27916	0.30152	-1.22471

RC

C	2.46869	-0.05729	-0.12243
C	2.77329	-1.55125	-0.06202
C	3.9791	-1.81082	-0.75128

C	4.5644	-0.53375	-1.18212
C	3.71016	0.51197	-0.77796
C	4.03639	1.83677	-1.04648
C	5.23961	2.1192	-1.70353
C	6.09248	1.08266	-2.09745
C	5.76115	-0.25016	-1.84272
C	4.4576	-3.11504	-0.881
C	3.74636	-4.15558	-0.27896
C	2.604	-3.88642	0.48083
C	2.11248	-2.57981	0.60459
C	2.047	0.54145	1.22062
O	0.74199	-0.01709	1.52371
C	-0.33194	0.72575	1.09652
O	-0.85914	1.58486	1.77447
N	-0.75775	0.37187	-0.15194
C	-1.9564	0.97233	-0.73228
C	-2.61221	-0.14465	-1.57206
O	-1.96524	-0.71695	-2.3807
C	-3.78769	-4.4627	-1.62536
C	-4.76009	-5.70597	-1.43863
C	-5.42757	-6.13077	-2.78652
C	-6.49663	-5.03309	-2.97799
C	-6.85811	-4.69259	-1.52595
N	-5.94215	-5.26177	-0.7195
O	-7.78082	-3.94355	-1.18916
C	-6.68405	-0.62913	1.38174
C	-7.08258	-1.91152	2.18907
C	-6.05628	-2.07409	3.34455
C	-6.24285	-3.53382	3.77641
C	-6.5865	-4.20213	2.44789
N	-7.09919	-3.2586	1.62269
O	-6.43241	-5.39121	2.1473
P	-7.0284	-0.26488	-0.37995
O	-4.00623	-0.29845	-1.59584

H	2.69016	0.21631	2.04077
H	1.97179	1.63363	1.20279
H	3.37672	2.64768	-0.7462
H	5.51263	3.15047	-1.90922
H	7.02212	1.31678	-2.60913
H	6.4274	-1.04977	-2.15589
H	5.38199	-3.31826	-1.41547
H	4.10434	-5.17749	-0.37171
H	2.09395	-4.69875	0.99176
H	1.29887	-2.36217	1.28633
H	-0.25739	-0.27547	-0.75866
H	-2.56488	1.32272	0.10027
C	-1.6064	2.15171	-1.65565
H	-1.23712	2.97153	-1.03403
H	-0.82743	1.84678	-2.36
H	-2.47145	2.507	-2.21715
H	-8.07555	-1.70962	2.61225
H	-7.19641	0.16158	1.94345
H	-5.59773	-0.49254	1.42187
H	-6.20068	-1.33839	4.1398
H	-5.04502	-1.95205	2.93871
H	-7.09949	-3.64579	4.45391
H	-5.36705	-3.98873	4.24329
H	-7.46141	-3.51951	0.68623
H	-6.07839	-4.13925	-3.46799
H	-7.38345	-5.32296	-3.54873
H	-4.73275	-6.21607	-3.62718
H	-5.91741	-7.0989	-2.63938
H	-6.0166	-5.27828	0.30842
H	1.5741	0.10221	-0.74023
H	-2.95763	-4.5555	-0.95657
H	-4.28418	-6.56413	-0.95806
C	-6.89784	1.49327	-0.80191
C	-6.73304	1.82245	-2.16023

C	-7.04665	2.50818	0.15853
C	-6.69741	3.15897	-2.54922
H	-6.63979	1.04533	-2.91327
C	-7.01228	3.84309	-0.24754
H	-7.27647	2.23547	1.18553
C	-6.83525	4.1686	-1.59155
H	-6.57128	3.41123	-3.59819
H	-7.1451	4.62507	0.49364
H	-6.81747	5.21028	-1.90049
C	-8.45609	-1.06481	-1.12437
C	-9.57495	-1.39011	-0.34772
C	-8.41727	-1.38553	-2.49292
C	-10.65127	-2.04096	-0.94789
H	-9.61808	-1.10131	0.69708
C	-9.49583	-2.04333	-3.07422
H	-7.54372	-1.15599	-3.09346
C	-10.61323	-2.37097	-2.30115
H	-11.52202	-2.28819	-0.3483
H	-9.46629	-2.2969	-4.13029
H	-11.45629	-2.88096	-2.75927
H	-4.31903	-3.55899	-1.41112
O	-3.31205	-4.42782	-2.97349
H	-2.44271	-4.0212	-2.99616
Cl	-8.62384	0.50075	0.63492
Cl	-5.44978	-1.11687	-1.35138
H	-4.22384	-1.22248	-1.7387

TS1

C	-0.70857	-0.59599	0.32862
C	-0.63663	-2.12017	0.32401
C	0.49781	-2.53248	-0.41048
C	1.26251	-1.34333	-0.81081
C	0.5897	-0.19682	-0.34233
C	1.10977	1.07239	-0.57092
C	2.32599	1.19428	-1.25295

C	2.99911	0.05662	-1.7108
C	2.47244	-1.21916	-1.49613
C	0.76622	-3.88806	-0.60315
C	-0.08228	-4.83151	-0.01921
C	-1.15087	-4.42238	0.78419
C	-1.43191	-3.06219	0.97138
C	-1.00048	0.00398	1.70519
O	-2.36833	-0.36023	2.02641
C	-3.32483	0.55538	1.65981
O	-3.69679	1.4562	2.38994
N	-3.83001	0.32302	0.41742
C	-4.93533	1.12851	-0.10372
C	-5.77555	0.16096	-0.96945
O	-5.23919	-0.44458	-1.83302
C	-7.25858	-4.07829	-1.32938
C	-8.40628	-5.16967	-1.14496
C	-9.16351	-5.43092	-2.48703
C	-10.0448	-4.1705	-2.61009
C	-10.31443	-3.83924	-1.13611
N	-9.48307	-4.57495	-0.37444
O	-11.10224	-2.96737	-0.74394
C	-9.05042	-0.11068	1.73176
C	-9.6224	-1.34931	2.50231
C	-8.60596	-1.71495	3.61946
C	-9.00496	-3.14555	4.00196
C	-9.47939	-3.69762	2.66039
N	-9.86015	-2.65328	1.8868
O	-9.51777	-4.88276	2.31111
P	-9.37174	0.37482	-0.00392
O	-7.17677	0.21356	-0.95192
H	-0.39562	-0.44957	2.49287
H	-0.90678	1.09459	1.7304
H	0.59044	1.96158	-0.22084
H	2.74974	2.17921	-1.42813

H	3.94115	0.16667	-2.24118
H	2.99969	-2.09766	-1.85887
H	1.63509	-4.20808	-1.17246
H	0.11149	-5.89145	-0.16078
H	-1.76765	-5.16753	1.27958
H	-2.1856	-2.75102	1.68499
H	-3.45019	-0.36256	-0.23285
H	-5.46231	1.52808	0.7593
C	-4.42997	2.27769	-0.98755
H	-3.92384	3.00478	-0.34717
H	-3.7245	1.88659	-1.73096
H	-5.24313	2.78426	-1.50907
H	-10.5619	-1.01549	2.96247
H	-9.41606	0.72515	2.33778
H	-7.95538	-0.14383	1.74421
H	-8.61603	-0.99947	4.4456
H	-7.59804	-1.73246	3.18816
H	-9.85207	-3.15306	4.70033
H	-8.19881	-3.74819	4.42492
H	-10.28068	-2.81654	0.9526
H	-9.50564	-3.33168	-3.07877
H	-10.97917	-4.29221	-3.16425
H	-8.50556	-5.58664	-3.35085
H	-9.79809	-6.31289	-2.36642
H	-9.53444	-4.62253	0.65392
H	-1.58247	-0.27587	-0.25555
H	-6.43691	-4.3048	-0.68984
H	-8.05698	-6.10979	-0.71131
C	-8.98682	2.10823	-0.36371
C	-8.80594	2.4644	-1.71315
C	-8.95421	3.0932	0.63799
C	-8.57401	3.79468	-2.05287
H	-8.85177	1.71411	-2.49713
C	-8.72418	4.4228	0.28118

H	-9.19857	2.81651	1.66055
C	-8.53144	4.77287	-1.05447
H	-8.43575	4.06798	-3.09503
H	-8.71697	5.18444	1.0547
H	-8.36068	5.81146	-1.32471
C	-10.92819	-0.16545	-0.7343
C	-12.06482	-0.34755	0.06303
C	-10.97221	-0.43124	-2.11442
C	-13.24276	-0.80042	-0.5283
H	-12.03777	-0.09917	1.11871
C	-12.15296	-0.89141	-2.68709
H	-10.0884	-0.31343	-2.73203
C	-13.2886	-1.07589	-1.89347
H	-14.12654	-0.93608	0.08762
H	-12.18828	-1.10246	-3.7523
H	-14.21099	-1.43131	-2.34468
H	-7.6489	-3.11857	-1.07563
O	-6.8108	-4.02572	-2.68898
H	-5.74496	-4.26144	-2.68766
Cl	-10.81017	1.33293	1.08645
Cl	-8.47171	-1.51575	-0.57064
H	-7.30629	-0.86497	-1.06867

IM1

C	4.87568	0.80764	0.46616
C	5.20225	-0.67935	0.5688
C	6.41581	-0.93924	-0.10661
C	6.98442	0.33439	-0.56883
C	6.11228	1.37765	-0.19787
C	6.4202	2.69947	-0.50053
C	7.62286	2.98215	-1.1585
C	8.49343	1.94833	-1.51946
C	8.18062	0.61804	-1.2304
C	6.91452	-2.2391	-0.19823
C	6.21545	-3.27359	0.42801

C	5.06478	-3.00125	1.17401
C	4.55305	-1.69934	1.25953
C	4.43726	1.43611	1.79014
O	3.13901	0.86632	2.10122
C	2.05658	1.58098	1.64819
O	1.51265	2.45008	2.29967
N	1.6434	1.18701	0.40752
C	0.4393	1.75332	-0.19541
C	-0.19482	0.6042	-1.00805
O	0.46535	0.02005	-1.79733
C	0.54219	-3.23084	-1.16876
C	-0.48856	-4.34606	-1.02364
C	-1.14175	-4.81728	-2.36308
C	-2.22599	-3.74149	-2.5901
C	-2.60098	-3.36727	-1.14983
N	-1.68131	-3.90047	-0.32325
O	-3.53674	-2.62341	-0.83853
C	-2.5047	0.77566	1.64723
C	-2.88868	-0.49026	2.48695
C	-1.86682	-0.60592	3.65196
C	-2.03402	-2.05607	4.12233
C	-2.35988	-2.76526	2.81062
N	-2.88183	-1.8523	1.95732
O	-2.18626	-3.95958	2.5433
P	-2.8442	1.08679	-0.12555
O	-1.58622	0.42876	-1.03519
Cl	-5.14456	2.64012	2.04391
H	5.08042	1.14308	2.62229
H	4.34581	2.52615	1.74221
H	5.74673	3.50815	-0.22598
H	7.8816	4.01147	-1.39066
H	9.42243	2.18253	-2.03228
H	8.86057	-0.17959	-1.51811
H	7.84495	-2.44274	-0.72197

H	6.58922	-4.29211	0.36501
H	4.56398	-3.80706	1.70405
H	3.73231	-1.47564	1.93069
H	2.15692	0.5311	-0.17866
H	-0.17921	2.1169	0.62401
C	0.77697	2.91241	-1.14859
H	1.13031	3.75426	-0.54749
H	1.5645	2.60032	-1.84016
H	-0.09002	3.23923	-1.72422
H	-3.88701	-0.29201	2.8991
H	-3.0321	1.57346	2.18446
H	-1.4208	0.9297	1.68952
H	-2.02684	0.14879	4.42612
H	-0.85515	-0.47964	3.24844
H	-2.89282	-2.16258	4.79797
H	-1.15424	-2.48486	4.60613
H	-3.23465	-2.14393	1.02634
H	-1.81833	-2.85503	-3.10195
H	-3.10503	-4.06009	-3.15754
H	-0.44085	-4.91481	-3.19734
H	-1.61789	-5.78836	-2.19233
H	-1.76148	-3.89024	0.70431
H	3.98241	0.9368	-0.16059
H	0.78531	-2.85208	-0.16429
H	-0.00266	-5.18359	-0.51735
C	-2.73752	2.83463	-0.59443
C	-2.56976	3.12934	-1.96028
C	-2.9071	3.87283	0.33724
C	-2.55188	4.45521	-2.38526
H	-2.46051	2.3336	-2.6914
C	-2.89035	5.19661	-0.10476
H	-3.13879	3.62461	1.37002
C	-2.71039	5.48822	-1.45615
H	-2.42344	4.68084	-3.44

H	-3.03918	5.99629	0.61416
H	-2.70641	6.5213	-1.79319
C	-4.2554	0.24548	-0.85567
C	-5.37376	-0.07554	-0.07654
C	-4.20381	-0.11159	-2.21477
C	-6.43671	-0.75861	-0.66462
H	-5.42729	0.24079	0.95978
C	-5.26899	-0.80113	-2.7838
H	-3.33031	0.11479	-2.81658
C	-6.38585	-1.12456	-2.00818
H	-7.30714	-1.00265	-0.06325
H	-5.22952	-1.08275	-3.83241
H	-7.2185	-1.65946	-2.45683
H	0.12867	-2.39772	-1.74871
O	1.68206	-3.79961	-1.80375
H	2.36454	-3.12533	-1.86362

(*Si*,*S*)-TS2

C	-4.90002	-0.6465	0.29158
C	-5.58452	0.51329	1.05435
C	-6.66397	1.01104	0.28901
C	-6.83831	0.15864	-0.9022
C	-5.85544	-0.85544	-0.87605
C	-5.82396	-1.83812	-1.85746
C	-6.78738	-1.8131	-2.87479
C	-7.76052	-0.80747	-2.90503
C	-7.79278	0.18419	-1.91992
C	-7.39778	2.11535	0.72267
C	-7.08837	2.68531	1.96037
C	-6.11198	2.10646	2.77898
C	-5.35805	1.00765	2.34467
C	-4.65846	-1.67303	1.40463
O	-3.48091	-0.62513	2.03256
C	-2.27743	-1.16456	1.36647
O	-1.75467	-2.17671	1.84149

N	-1.83577	-0.61882	0.19347
C	-0.64138	-1.21962	-0.43751
C	0.12509	-0.06812	-1.06088
O	-0.39116	0.74784	-1.79993
O	0.57298	1.86903	-1.1555
C	0.08472	3.02719	-1.84764
C	0.98776	4.30091	-1.51099
C	1.95421	4.49768	-2.73648
C	3.00219	3.36923	-2.54074
C	3.08017	3.28916	-1.00066
N	1.97549	3.95761	-0.48352
O	3.88739	2.61413	-0.31217
C	2.27344	-0.43987	1.79991
C	2.8674	0.83584	2.43131
C	1.90844	1.36016	3.54961
C	2.31547	2.84145	3.71405
C	2.71706	3.22204	2.2806
N	3.02142	2.05894	1.60321
O	2.74869	4.37119	1.78046
P	2.63883	-0.90037	0.02498
O	1.56423	-0.03134	-0.96715
Cl	4.60963	-2.61333	2.31777
H	-5.53841	-1.703	2.04668
H	-4.31025	-2.68622	1.19995
H	-5.06714	-2.61541	-1.84122
H	-6.77988	-2.57452	-3.64587
H	-8.49747	-0.79878	-3.69948
H	-8.55153	0.9583	-1.94423
H	-8.21695	2.50565	0.12936
H	-7.65369	3.53612	2.32212
H	-5.95438	2.48969	3.78037
H	-4.78217	0.43522	3.06383
H	-2.24717	0.14586	-0.31838
H	-0.08873	-1.73296	0.34342

C	-1.08138	-2.21393	-1.53691
H	-1.53044	-3.08854	-1.05818
H	-1.81667	-1.71755	-2.17661
H	-0.22886	-2.53769	-2.14327
H	3.83839	0.56881	2.86779
H	2.68095	-1.25774	2.40846
H	1.17696	-0.40864	1.82046
H	1.99395	0.77967	4.4703
H	0.87679	1.30028	3.18839
H	3.19602	2.92564	4.36165
H	1.51149	3.48208	4.07633
H	3.29987	1.92137	0.64284
H	2.66148	2.40743	-2.94126
H	3.97844	3.61264	-2.96359
H	1.43443	4.43722	-3.69632
H	2.44511	5.47223	-2.66079
H	1.96575	4.07878	0.56438
H	1.50607	1.67527	-1.41289
H	-3.93602	-0.3941	-0.14321
H	-0.938	3.15671	-1.48761
H	0.07301	2.87865	-2.93602
H	0.42727	5.21827	-1.33593
C	2.33559	-2.70118	-0.26181
C	2.21093	-3.18487	-1.57093
C	2.30016	-3.57406	0.82589
C	2.03771	-4.551	-1.78925
H	2.25404	-2.51256	-2.41556
C	2.13225	-4.94105	0.59764
H	2.45509	-3.19223	1.82782
C	1.99982	-5.42897	-0.70265
H	1.94305	-4.92664	-2.80113
H	2.11805	-5.62056	1.4408
H	1.87592	-6.49234	-0.87174
C	4.1953	-0.35444	-0.76912

C	5.3359	-0.0491	-0.02917
C	4.19012	-0.23456	-2.16642
C	6.48582	0.37023	-0.69889
H	5.33589	-0.16686	1.04484
C	5.34489	0.18372	-2.8244
H	3.29517	-0.45594	-2.73514
C	6.49391	0.48566	-2.08934
H	7.37598	0.601	-0.1272
H	5.3464	0.27477	-3.90418
H	7.39253	0.81079	-2.60044

(*Si*,*R*)-TS2

C	4.93489	0.67949	0.44482
C	5.23949	-0.81447	0.50523
C	6.4453	-1.07404	-0.18403
C	7.0306	0.20303	-0.61488
C	6.17636	1.24875	-0.21071
C	6.50259	2.57355	-0.47923
C	7.70581	2.85598	-1.13629
C	8.55868	1.81944	-1.5302
C	8.22735	0.48662	-1.27547
C	6.9238	-2.37826	-0.31375
C	6.21256	-3.4188	0.28829
C	5.0702	-3.14964	1.04808
C	4.57868	-1.84303	1.17184
C	4.5132	1.27823	1.78787
O	3.20819	0.71969	2.09096
C	2.13426	1.46253	1.66377
O	1.60706	2.32164	2.34172
N	1.70845	1.10865	0.41531
C	0.5098	1.70911	-0.16504
C	-0.14602	0.59213	-1.00481
O	0.50096	0.01983	-1.81345
O	-0.22443	-1.89591	-1.08752
C	0.45773	-3.10766	-1.33955

C	-0.51466	-4.35094	-1.15282
C	-1.18215	-4.77573	-2.50071
C	-2.2512	-3.67806	-2.69218
C	-2.61269	-3.33756	-1.24014
N	-1.69673	-3.90674	-0.43368
O	-3.5354	-2.58851	-0.90335
C	-2.43863	0.7259	1.66755
C	-2.83715	-0.55648	2.47488
C	-1.81086	-0.71906	3.63036
C	-1.99743	-2.17879	4.06222
C	-2.34107	-2.8471	2.7337
N	-2.85377	-1.90357	1.9085
O	-2.18698	-4.03618	2.43311
P	-2.78297	1.09015	-0.09414
O	-1.54003	0.43833	-1.02859
Cl	-5.04779	2.61821	2.12997
H	5.15636	0.95309	2.60802
H	4.43799	2.37041	1.77004
H	5.84292	3.38446	-0.17895
H	7.97883	3.88725	-1.34197
H	9.48832	2.05356	-2.04188
H	8.8936	-0.31299	-1.58864
H	7.84819	-2.58148	-0.84822
H	6.57054	-4.44071	0.19554
H	4.56015	-3.96197	1.55901
H	3.76507	-1.62539	1.85357
H	2.20881	0.46131	-0.19142
H	-3.83012	-0.35459	2.89806
H	-2.95098	1.51661	2.22926
H	-1.3523	0.86249	1.70769
H	-1.95526	0.01665	4.42561
H	-0.7996	-0.59702	3.22452
H	-2.85406	-2.29076	4.73973
H	-1.12163	-2.63369	4.5291

H	-3.21599	-2.16448	0.97204
H	-1.83297	-2.78422	-3.18218
H	-3.13802	-3.96793	-3.26292
H	-0.48733	-4.86104	-3.34137
H	-1.67198	-5.74386	-2.35356
H	-1.77117	-3.92325	0.59423
H	-1.03566	-1.86011	-1.60103
H	4.0403	0.83899	-0.17298
H	1.33245	-3.13161	-0.68454
H	0.80722	-3.14858	-2.38493
H	-0.03875	-5.2091	-0.67225
C	-2.65241	2.8483	-0.5161
C	-2.48761	3.17748	-1.87442
C	-2.80122	3.86321	0.44434
C	-2.45198	4.514	-2.26341
H	-2.39437	2.40037	-2.62746
C	-2.76685	5.19812	0.03828
H	-3.03105	3.59051	1.47134
C	-2.58982	5.52363	-1.30574
H	-2.32586	4.76626	-3.31238
H	-2.89968	5.9801	0.77946
H	-2.57204	6.56531	-1.61467
C	-4.21066	0.29022	-0.83855
C	-5.32952	-0.03508	-0.06191
C	-4.17185	-0.0305	-2.20711
C	-6.40585	-0.68593	-0.66208
H	-5.37265	0.25373	0.98289
C	-5.2504	-0.68829	-2.7884
H	-3.29829	0.19905	-2.80764
C	-6.3678	-1.01593	-2.01534
H	-7.27659	-0.93316	-0.06249
H	-5.22086	-0.94187	-3.84447
H	-7.21086	-1.52592	-2.47346
C	-0.35059	2.20456	1.01219

H	-1.35956	2.34191	0.68354
H	-0.3275	1.48103	1.80015
H	0.03749	3.13497	1.37083
H	0.77994	2.53126	-0.79431

(*Re*,*S*)-TS2

C	5.53135	-0.34906	-0.10987
C	6.02016	-1.76629	-0.39631
C	7.43135	-1.81977	-0.3378
C	7.94002	-0.47481	-0.00855
C	6.83994	0.40245	0.12966
C	7.03944	1.74234	0.43636
C	8.34876	2.20997	0.61541
C	9.43764	1.34189	0.48236
C	9.24224	-0.00669	0.16708
C	8.10248	-3.02274	-0.55834
C	7.35294	-4.17174	-0.83033
C	5.95497	-4.12008	-0.86969
C	5.27814	-2.91335	-0.64881
C	4.62685	-0.29079	1.13045
O	3.34732	-0.99309	0.85947
C	2.39801	-0.24286	0.19111
O	2.64896	0.91497	-0.23082
N	1.24379	-0.91825	0.0865
C	0.04786	-0.39583	-0.5914
C	-1.12527	-1.20048	-0.05476
O	-1.0166	-2.16405	0.71085
O	0.88444	2.48011	-1.52335
C	1.11197	3.88938	-1.77992
C	0.73755	4.7353	-0.53787
C	1.61334	4.37029	0.69842
C	0.84975	3.19654	1.36495
C	-0.54365	3.24712	0.72346
N	-0.60801	4.30739	-0.10712
O	-1.47818	2.42981	0.95296

C	-4.5169	0.43428	-1.19638
C	-4.85723	1.72085	-0.39181
C	-5.95413	2.57307	-1.09481
C	-5.15994	3.46031	-2.07415
C	-3.82407	3.65337	-1.32888
N	-3.7287	2.67086	-0.39251
O	-2.95676	4.52573	-1.56414
P	-4.01726	-1.0009	-0.06534
O	-2.27693	-0.69449	-0.55313
Cl	-6.6173	-0.96668	0.39141
H	5.06683	-0.83396	1.96525
H	4.4095	0.74562	1.39451
H	6.19985	2.42248	0.53168
H	8.51903	3.25288	0.8559
H	10.44377	1.7198	0.62265
H	10.09041	-0.67357	0.06171
H	9.18474	-3.07094	-0.51641
H	7.85969	-5.11364	-1.00613
H	5.39027	-5.02347	-1.069
H	4.19705	-2.87315	-0.64283
H	1.11847	-1.81035	0.56574
H	-0.11436	0.64995	-0.32308
C	0.12595	-0.51077	-2.1496
H	0.05919	0.49879	-2.55581
H	1.08683	-0.95236	-2.4276
H	-0.6824	-1.13491	-2.53708
H	-5.16334	1.45834	0.6214
H	-5.38323	0.07504	-1.74675
H	-3.65828	0.60445	-1.84568
H	-6.44154	3.2029	-0.34399
H	-6.71297	1.94672	-1.56393
H	-5.62089	4.42475	-2.29233
H	-4.96258	2.94803	-3.02425
H	-2.86361	2.5597	0.17499

H	1.32152	2.23943	1.14223
H	0.75386	3.30734	2.44884
H	2.62428	4.08243	0.39697
H	1.67503	5.22698	1.37429
H	-1.45804	4.45964	-0.69318
H	1.66503	2.02061	-1.08039
H	4.9794	0.07965	-0.9546
H	0.43893	4.16142	-2.59904
H	2.14348	4.11656	-2.08726
H	0.76705	5.8017	-0.77424
C	-4.22223	-2.71902	-0.7289
C	-3.32715	-3.72226	-0.33754
C	-5.27235	-3.00571	-1.60654
C	-3.4882	-5.01546	-0.84208
H	-2.49383	-3.48539	0.30906
C	-5.41069	-4.29416	-2.11872
H	-6.00614	-2.24692	-1.8385
C	-4.52181	-5.30184	-1.7341
H	-2.79962	-5.79408	-0.53593
H	-6.22387	-4.51359	-2.79988
H	-4.64008	-6.30598	-2.12441
C	-3.77314	-0.84515	1.76967
C	-4.50654	-1.67447	2.62386
C	-2.84689	0.07186	2.27583
C	-4.29689	-1.59569	3.9992
H	-5.25566	-2.33889	2.21857
C	-2.66834	0.15838	3.65944
H	-2.26236	0.72529	1.63826
C	-3.38315	-0.67484	4.51946
H	-4.85955	-2.24183	4.66201
H	-1.96413	0.88167	4.0529
H	-3.23519	-0.60531	5.59096

(*Re*,*R*)-TS2

C	5.71218	0.12582	0.54478

C	5.88222	0.49437	-0.92896
C	7.25364	0.54331	-1.26475
C	8.04532	0.2412	-0.0625
C	7.16199	0.00995	1.01235
C	7.65451	-0.32759	2.26815
C	9.04014	-0.41851	2.45266
C	9.91569	-0.18164	1.38826
C	9.42556	0.14811	0.12176
C	7.66035	0.87966	-2.55667
C	6.68766	1.18069	-3.51249
C	5.33078	1.16136	-3.17577
C	4.92048	0.82179	-1.8804
C	5.03038	-1.23899	0.7723
O	3.64349	-1.36991	0.33888
C	2.79511	-0.29934	0.47281
O	2.88143	0.55577	1.33996
N	1.81374	-0.41387	-0.47994
C	0.72512	0.53306	-0.73743
C	-0.40616	-0.31209	-1.35658
O	-0.25538	-0.97967	-2.3475
O	-1.47346	2.56846	-1.37586
C	-1.70854	3.95878	-1.67624
C	-2.66986	4.66761	-0.63875
C	-1.87731	5.35386	0.51527
C	-1.56369	4.18018	1.46366
C	-2.79817	3.26644	1.25696
N	-3.44388	3.70085	0.13604
O	-3.08674	2.26438	1.92017
C	-4.19586	-1.55902	-0.94409
C	-5.46189	-0.74145	-0.53301
C	-6.1692	-0.24717	-1.80163
C	-6.82827	1.1019	-1.45163
C	-5.99994	1.60853	-0.28601
N	-5.17774	0.61172	0.10457

O	-5.99623	2.73716	0.23613
P	-2.5794	-1.50479	-0.08474
O	-1.61143	-0.27628	-0.66126
Cl	-6.89105	-2.08077	-1.45117
H	5.55913	-2.00591	0.20372
H	5.07399	-1.49249	1.83672
H	6.98207	-0.51054	3.10168
H	9.43646	-0.6741	3.43047
H	10.98716	-0.2552	1.54768
H	10.11088	0.32837	-0.70103
H	8.71447	0.91438	-2.81545
H	6.98864	1.44927	-4.52053
H	4.58848	1.42245	-3.92388
H	3.86612	0.85055	-1.62562
H	1.87022	-1.22146	-1.09005
H	-6.04178	-1.31656	0.16758
H	-4.46453	-2.61921	-0.85359
H	-3.94461	-1.31447	-1.97653
H	-6.77196	-1.06408	-2.15477
H	-5.39641	-0.00879	-2.5575
H	-7.85794	0.95698	-1.08493
H	-6.84393	1.8248	-2.26973
H	-4.52951	0.73722	0.88653
H	-0.64813	3.652	1.18124
H	-1.46327	4.46883	2.51261
H	-0.99687	5.91778	0.19297
H	-2.56685	6.02983	1.03173
H	-4.46303	3.46826	0.03343
H	-1.27099	2.47228	-0.42076
H	5.17143	0.89986	1.09682
H	-2.12057	3.96619	-2.69004
H	-0.76358	4.52429	-1.66931
H	-3.29227	5.37121	-1.19705
C	-1.69726	-3.0747	-0.38561

C	-0.35228	-3.17943	0.02451
C	-2.3536	-4.20139	-0.91149
C	0.32696	-4.38491	-0.11736
H	0.17397	-2.32445	0.42998
C	-1.66121	-5.40446	-1.04125
H	-3.40419	-4.17345	-1.17221
C	-0.32413	-5.50047	-0.65167
H	1.36482	-4.45239	0.19221
H	-2.18186	-6.27149	-1.43347
H	0.20729	-6.44073	-0.75602
C	-2.65144	-1.16561	1.69224
C	-3.82595	-1.4191	2.41377
C	-1.50933	-0.6656	2.33428
C	-3.84958	-1.16244	3.78263
H	-4.69078	-1.86005	1.92817
C	-1.54916	-0.40672	3.70137
H	-0.60264	-0.47982	1.76817
C	-2.71917	-0.64863	4.42307
H	-4.7521	-1.36918	4.34736
H	-0.66886	-0.01819	4.20265
H	-2.74828	-0.44344	5.48814
C	0.33624	1.48272	0.41616
H	-0.71637	1.75523	0.30761
H	0.44301	1.05534	1.40434
H	0.94529	2.38576	0.34614
H	1.00463	1.19934	-1.56941

PC

C	-4.514	0.0054	0.0312
C	-4.51876	-0.43276	-1.46162
C	-3.42406	0.15913	-2.13074
C	-2.6103	0.89398	-1.15253
C	-3.21349	0.77374	0.11566
C	-2.63591	1.36561	1.23357
C	-1.43151	2.06327	1.08559

C	-0.82732	2.17592	-0.1711
C	-1.41214	1.59541	-1.29895
C	-3.22864	-0.04852	-3.49656
C	-4.10942	-0.88827	-4.18217
C	-5.13691	-1.5465	-3.49972
C	-5.34484	-1.33236	-2.13039
C	-4.74291	-1.13953	0.98834
O	-6.11937	-1.56365	0.80852
C	-7.03709	-0.95431	1.62957
O	-7.34618	-1.38914	2.7209
N	-7.58254	0.16758	1.07365
C	-8.65826	0.89012	1.74807
C	-9.56602	1.42564	0.62017
O	-9.08712	2.05843	-0.25746
O	-10.96165	1.4207	0.76049
H	-4.14318	-2.02021	0.75014
H	-4.59403	-0.84582	2.03257
H	-3.10157	1.28505	2.21316
H	-0.96311	2.52062	1.95264
H	0.10631	2.72286	-0.27139
H	-0.93834	1.69125	-2.27243
H	-2.39057	0.41005	-4.01504
H	-3.97247	-1.05945	-5.24659
H	-5.77846	-2.24203	-4.03425
H	-6.06495	-1.93065	-1.58485
H	-7.25316	0.58719	0.20591
H	-9.1438	0.17784	2.41373
C	-8.11695	2.07867	2.5607
H	-7.56002	1.6814	3.41329
H	-7.44959	2.67705	1.93423
H	-8.91572	2.71845	2.93814
H	-5.38424	0.65044	0.18446
C	-11.56962	1.28148	-0.52632
H	-11.38916	2.16425	-1.10344

H	-11.15069	0.43489	-1.02901
C	-13.08701	1.07948	-0.35802
C	-13.46686	1.09054	1.11593
H	-13.59215	1.92005	-0.89717
C	-14.24931	-1.03158	0.14334
C	-14.15941	-0.22735	1.43236
H	-14.14534	1.95235	1.33763
H	-12.55637	1.21639	1.75405
H	-15.18026	-0.04374	1.85178
H	-13.58274	-0.79413	2.20605
N	-13.51476	-0.25889	-0.94268
H	-13.34341	-0.57486	-1.87585
O	-14.82474	-2.13218	0.00174
H	-14.97205	-2.29844	-0.93221
N	-15.1255	-2.47161	-1.90508
C	-15.79486	-3.57164	-2.43329
C	-14.70697	-1.65603	-2.95235
C	-15.82424	-3.48917	-3.86834
C	-15.1272	-2.26195	-4.20086
H	-15.31967	-4.32937	-4.29774
H	-14.2722	-2.48077	-4.80582
O	-14.07835	-0.59607	-2.74301
H	-16.79696	-3.59031	-2.05867
H	-16.83529	-3.44638	-4.21596
H	-15.78465	-1.59966	-4.72435
C	-15.06816	-4.86211	-2.01114
H	-15.18178	-5.00692	-0.95709
H	-14.02835	-4.7803	-2.24988
P	-15.78474	-6.28006	-2.89903
C	-15.55745	-7.79191	-1.91159
C	-16.44283	-8.08745	-0.87466
C	-14.49823	-8.65518	-2.19152
C	-16.26855	-9.24567	-0.11744
H	-17.27735	-7.40627	-0.65372

C	-14.32433	-9.8143	-1.43481
H	-13.80042	-8.42249	-3.00887
C	-15.20918	-10.10959	-0.3978
H	-16.96601	-9.47836	0.7003
H	-13.48933	-10.49497	-1.65602
H	-15.072	-11.02277	0.19933
C	-14.95168	-6.47377	-4.50555
C	-15.69854	-6.46635	-5.68394
C	-13.5666	-6.62953	-4.55873
C	-15.06046	-6.61534	-6.91515
H	-16.79059	-6.3443	-5.64163
C	-12.92805	-6.77759	-5.79029
H	-12.97778	-6.63522	-3.63008
C	-13.67473	-6.77066	-6.96841
H	-15.64915	-6.61014	-7.84397
H	-11.83592	-6.89997	-5.83194
H	-13.17183	-6.88806	-7.93929
O	-17.25206	-6.04255	-3.12101

(*S*,*S*)-4a

C	2.30912	0.1634	0.06085
C	3.36247	-0.85481	-0.35973
C	4.62888	-0.23165	-0.45445
C	4.47597	1.19643	-0.15158
C	3.11891	1.45383	0.14035
C	2.69272	2.73694	0.46774
C	3.63184	3.778	0.48734
C	4.97718	3.52544	0.19236
C	5.4106	2.235	-0.12784
C	5.76191	-0.97346	-0.79494
C	5.62052	-2.34166	-1.0457
C	4.36439	-2.95591	-0.96394
C	3.22598	-2.21626	-0.61543
H	1.64634	2.93909	0.69197
H	3.31126	4.79016	0.726

H	5.69232	4.34527	0.20319
H	6.45689	2.04391	-0.35936
H	6.73638	-0.49581	-0.86524
H	6.49168	-2.93306	-1.31788
H	4.27476	-4.01817	-1.18023
H	2.25473	-2.69666	-0.54251
C	1.67257	-0.14347	1.42669
O	0.87775	-1.36848	1.37022
C	-0.4756	-1.2008	1.21675
O	-1.04023	-0.11847	1.07875
N	-1.10391	-2.40819	1.25888
C	-2.54459	-2.51404	1.03171
C	-3.36206	-1.90791	2.18297
C	-2.8798	-1.94141	-0.35984
O	-2.13675	-1.99103	-1.32057
O	-4.1506	-1.44582	-0.39506
C	-4.60595	-0.87347	-1.66077
C	-4.63444	0.6528	-1.61611
C	-3.27972	1.34059	-1.296
C	-3.67668	2.64358	-0.57458
C	-5.05313	2.34432	0.03039
N	-5.50998	1.19736	-0.57943
O	-5.66134	2.99527	0.87298
H	1.49822	0.24214	-0.67943
H	1.01938	0.67538	1.73948
H	2.44381	-0.32922	2.17999
H	-0.52776	-3.2386	1.27301
H	-2.76188	-3.59047	0.96435
H	-4.43323	-2.04136	2.0052
H	-3.15303	-0.8376	2.26324
H	-3.08923	-2.39991	3.12331
H	-5.61096	-1.28234	-1.80837
H	-3.94318	-1.21351	-2.46182
H	-4.98843	0.96851	-2.61414

H	-2.69289	0.71765	-0.61386
H	-2.69044	1.49636	-2.20452
H	-3.78941	3.49053	-1.26658
H	-2.97499	2.94702	0.20696
H	-6.4303	0.83185	-0.36702

(*R*,*S*)-4a

C	2.72633	0.08246	-0.54131
C	3.80556	-0.92191	-0.15765
C	5.02659	-0.2533	0.09076
C	4.8244	1.18788	-0.10478
C	3.47779	1.41017	-0.47073
C	3.00537	2.70133	-0.68167
C	3.88704	3.78095	-0.52969
C	5.22578	3.56129	-0.18176
C	5.70377	2.26511	0.03224
C	6.16462	-0.97318	0.46304
C	6.0742	-2.36142	0.59393
C	4.8648	-3.02381	0.34487
C	3.72268	-2.30439	-0.03209
H	1.96772	2.87996	-0.95804
H	3.52758	4.79595	-0.68556
H	5.89933	4.40889	-0.07426
H	6.74527	2.09813	0.2977
H	7.10843	-0.46661	0.65186
H	6.95034	-2.93395	0.89117
H	4.8083	-4.10391	0.4575
H	2.78314	-2.8139	-0.22413
C	1.50144	0.10308	0.37524
O	0.7564	-1.14744	0.17998
C	-0.59397	-1.06948	0.40983
O	-1.20024	-0.03825	0.685
N	-1.1733	-2.29929	0.31655
C	-2.61734	-2.42831	0.44428
C	-3.02035	-3.90785	0.50379

C	-3.32032	-1.71831	-0.72329
O	-2.85561	-1.55225	-1.83325
O	-4.56033	-1.2974	-0.32993
C	-5.30893	-0.4659	-1.27439
C	-5.2592	1.00706	-0.8779
C	-3.85114	1.65058	-0.79292
C	-4.05895	2.81232	0.19741
C	-5.14696	2.28439	1.14554
N	-5.78549	1.26859	0.46732
O	-5.4251	2.67838	2.27169
H	2.3719	-0.10531	-1.56674
H	0.83458	0.93533	0.1349
H	1.78886	0.16784	1.43087
H	-0.62069	-3.07641	-0.02097
H	-2.93398	-1.91327	1.35805
H	-2.72537	-4.43302	-0.4139
H	-4.10471	-3.99844	0.61819
H	-2.53923	-4.3919	1.36142
H	-6.33441	-0.84907	-1.23477
H	-4.89282	-0.60883	-2.27674
H	-5.87295	1.54107	-1.62818
H	-3.15161	0.9302	-0.35373
H	-3.47027	1.95099	-1.77395
H	-4.45755	3.70408	-0.31028
H	-3.16294	3.10719	0.74826
H	-6.52768	0.73295	0.89984

HCl

Cl	-1	-9.29322	1.26317	1.84416
H	0	-8.00322	1.26317	1.84416

7. References

1. Hohenberg, P., Kohn, W. Inhomogeneous Electron Gas. *Phys. Rev.* **136**, B864-B871 (1964).

2. Kohn, W., Sham, L. J. Self-consistent Equations Including Exchange and Correlation Effects. *Phys. Rev.* **140**, A1133-A1138 (1965).

3. Frisch, M. J., Trucks, G. W., Schlegel, H. B., Scuseria, G. E., Robb, M. A., Cheeseman, J. R., Scalmani, G., Barone, V., Petersson, G. A., Nakatsuji, H., Li, X., Caricato, M., Marenich, A. V., Bloino, J., Janesko, B. G., Gomperts, R., Mennucci, B., Hratchian, H. P., Ortiz, J. V., Izmaylov, A. F., Sonnenberg, J. L., Williams-Young, D., Ding, F., Lipparini, F., Egidi, F., Goings, J., Peng, B., Petrone, A., Henderson, T., Ranasinghe, D., Zakrzewski, V. G., Gao, J., Rega, N., Zheng, G., Liang, W., Hada, M., Ehara, M., Toyota, K., Fukuda, R., Hasegawa, J., Ishida, M., Nakajima, T., Honda, Y., Kitao, O., Nakai, H., Vreven, T., Throssell, K., Montgomery, J. A. Jr., Peralta, J. E., Ogliaro, F., Bearpark, M. J., Heyd, J. J., Brothers, E. N., Kudin, K. N., Staroverov, V. N., Keith, T. A., Kobayashi, R., Normand, J., Raghavachari, K., Rendell, A. P., Burant, J. C., Iyengar, S. S., Tomasi, J., Cossi, M., Millam, J. M., Klene, M., Adamo, C., Cammi, R., Ochterski, J. W., Martin, R. L., Morokuma, K., Farkas, O., J. B. Foresman J. B., Fox, D. J. Gaussian16 Revision B.01, Gaussian Inc. Wallingford CT (2016).

4. Lee, C. T., Yang, W. T., Parr, R. G. Development of the Colle-Salvetti Correlation-energy Formula into a Functional of the Electron Density. *Phys. Rev. B* **37**, 785-789 (1988).

5. Becke, A. D. Density-functional Thermochemistry. III. The Role of Exact Exchange. *J. Chem. Phys.* **98**, 5648-5652 (1993).

6. Woon, D. E., Dunning, T. H. Jr. Gaussian Basis Sets for Use in Correlated Molecular Calculations. V. Core-valence Basis Sets for Boron Through Neon. *J. Chem. Phys.* **103**, 4572-4585 (1995).

7. Marenich, A. V., Cramer, C. J., Truhlar, D. G. Universal Solvation Model Based on Solute Electron Density and a Continuum Model of the Solvent Defined by the Bulk Dielectric Constant and Atomic Surface Tensions. *J. Phys. Chem. B* **113**, 6378-6396 (2009).
