# Supplementary material for: Kinetic resolution of amino acids by phosphine oxide catalyzed enantioselective esterification
Source: Nat Commun. 2026 Apr 13;17:5157. doi: 10.1038/s41467-026-71469-x (PMC13250068; doi:10.1038/s41467-026-71469-x)
Supplement: Supplementary file 2 — Description of Additional Supplementary Files [file 41467_2026_71469_MOESM2_ESM.pdf]

File Name: Supplementary Data 1

Description: The change in the enantiopurity of the recovered amino acid (*R*)-**1a** was obtained by varying the enantiomeric purity of catalyst **3a** (0, 20, 39, 61, 82, 99% ee) or *L*-pyroglutaminol **2a** (0, 20, 40, 60, 80, 99% ee).

File Name: Supplementary Data 2

Description: The nonlinear effect experiments of **3a** and **2a**.
