# Supplementary material for: Kinetic resolution of amino acids by phosphine oxide catalyzed enantioselective esterification
Source: Nat Commun. 2026 Apr 13;17:5157. doi: 10.1038/s41467-026-71469-x (PMC13250068; doi:10.1038/s41467-026-71469-x)
Supplement: Supplementary file 4 — Supplementary Data 2 [file 41467_2026_71469_MOESM4_ESM.pdf]

Nonlinear effect experiments.

Figure D1. Nonlinear effect experiment of **3a**

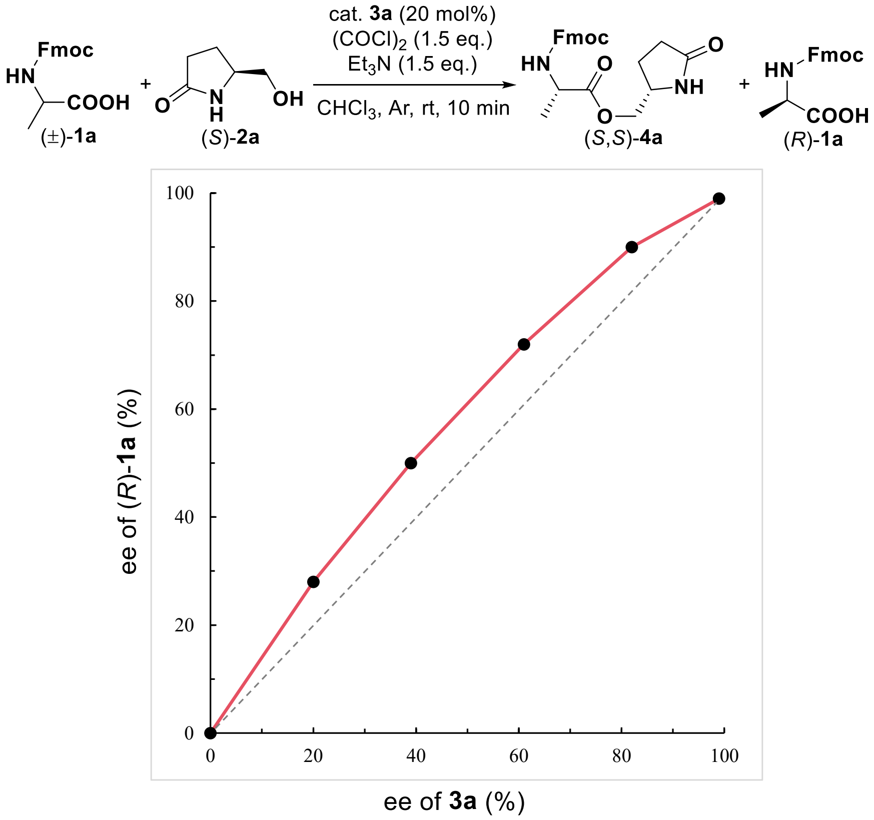

Racemic amino acid **1a** (0.5 mmol, 1.0 equiv.), *L*-pyroglutaminol **2a** (99% ee, 0.3 mmol, 0.6 equiv.) and catalyst **3a** (0, 20, 39, 61, 82, 99% ee, 0.1 mmol, 20 mol %) were well mixed in chloroform (1.0 mL). Then oxalyl chloride (0.75 mmol, 1.5 equiv.) and triethylamine (0.75 mmol, 1.5 equiv.) were added in sequence at ambient temperature under argon atmosphere. The resulting mixture was stirred at room temperature for 10 min. Subseqently, the mixture was partitioned between EtOAc (70 mL) and H_2O (30 mL) at room temperature. The organic layer was washed with saturated brine (30 mL×2), dried over Na_2SO_4, and concentrated *in vacuo*. The resulting residue was dissolved in dichloromethane (10 mL) and 1M aqueous solution NaOH (3 mL). The organic layer and the aqueous layer were separated. The aqueous layer was washed with dichloromethane (10 mL×2). The organic layer was discarded. The aqueous layer was made acidic with excess 1M aqueous solution HCl (to pH ~5) and was extracted with dichloromethane (10 mL×2). The combined organic layer was washed with brine (20 mL×2), dried over Na_2SO_4, then filtered and evaporated to afford the recovered amino acid (*R*)-**1a**. Enantiomeric excess was established by HPLC analysis using a CHIRALCEL AS-H column (*i*-propanol/*n*-hexane = 10/90, flow rate = 0.8 mL/min, wave length = 254 nm).

Figure D2. Nonlinear effect experiment of (*S*)-**2a**

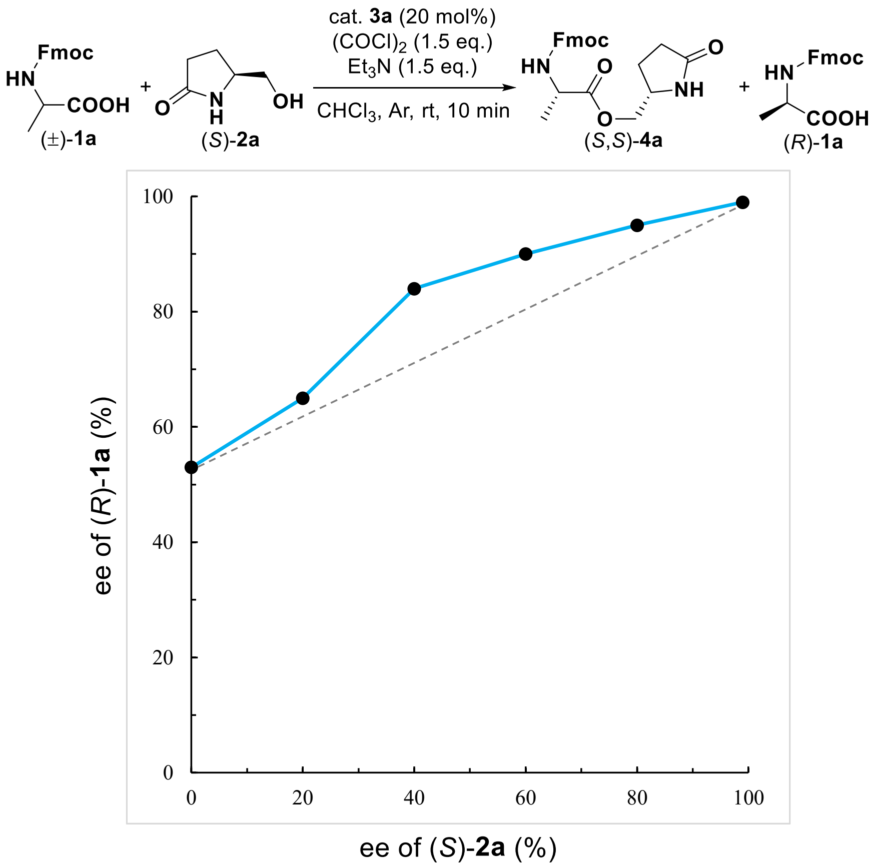

Racemic amino acid **1a** (0.5 mmol, 1.0 equiv.), *L*-pyroglutaminol **2a** (0, 20, 40, 60, 80, 99% ee, 0.3 mmol, 0.6 equiv.) and catalyst **3a** (99% ee, 0.1 mmol, 20 mol %) were well mixed in chloroform (1.0 mL). Then oxalyl chloride (0.75 mmol, 1.5 equiv.) and triethylamine (0.75 mmol, 1.5 equiv.) were added in sequence at ambient temperature under argon atmosphere. The resulting mixture was stirred at room temperature for 10 min. Subseqently, the mixture was partitioned between EtOAc (70 mL) and H_2O (30 mL) at room temperature. The organic layer was washed with saturated brine (30 mL×2), dried over Na_2SO_4, and concentrated *in vacuo*. The resulting residue was dissolved in dichloromethane (10 mL) and 1M aqueous solution NaOH (3 mL). The organic layer and the aqueous layer were separated. The aqueous layer was washed with dichloromethane (10 mL×2). The organic layer was discarded. The aqueous layer was made acidic with excess 1M aqueous solution HCl (to pH ~5) and was extracted with dichloromethane (10 mL×2). The combined organic layer was washed with brine (20 mL×2), dried over Na_2SO_4, then filtered and evaporated to afford the recovered amino acid (*R*)-**1a**. Enantiomeric excess was established by HPLC analysis using a CHIRALCEL AS-H column (*i*-propanol/*n*-hexane = 10/90, flow rate = 0.8 mL/min, wave length = 254 nm).
