# Supplementary material for: Kinetic resolution of amino acids by phosphine oxide catalyzed enantioselective esterification
Source: Nat Commun. 2026 Apr 13;17:5157. doi: 10.1038/s41467-026-71469-x (PMC13250068; doi:10.1038/s41467-026-71469-x)
Supplement: Supplementary file 5 — Transparent Peer Review file [file 41467_2026_71469_MOESM5_ESM.pdf]

nature portfolio

Peer Review File

Kinetic Resolution of Amino Acids by Phosphine Oxide Catalyzed Enantioselective Esterification

Corresponding Author: Dr Ji-Wei Ren

This file contains all reviewer reports in order by version, followed by all author rebuttals in order by version.

Version 0:

Reviewer comments:

Reviewer #1

(Remarks to the Author)
Ren and co-workers report a highly efficient kinetic resolution (KR) of racemic amino acids with L-pyroglutaminol as an esterification reagent. The process is catalyzed by a phosphine oxide organocatalyst under mild conditions and provides a wide range of chiral esters and recovered amino acids with excellent stereoselectivities. The intimate double H-bonding interaction between the pyroglutaminol core and the catalyst is proposed based on control experiments and DFT calculations.
Amino acids are undoubtedly an important class of compounds in chemistry and biology. Although many methods have been developed to make enantioenriched amino acids, kinetic resolution methods have been less developed. This work provides a practical and general solution to the problem. The newly designed phosphine oxide catalyst is a highlight of the work. The efficient resolution of a series of unnatural amino acids shows the importance of the work.
I suggest accepting the paper after the following issues being addressed:
1. In Fig 2, actually there are only examples of amino acids with trisubstituted stereocenters. So, the representative reaction equation should be revised.
2. This reviewer is interested in access to amino acids bearing tetrasubstituted stereocenters. But there is only one example. It will be very nice to see more examples.
3. A control experiment using a compound removing the carbonyl oxygen of (S)-2a should be conducted to the importance of the carbonyl group.

Reviewer #2

(Remarks to the Author)
The manuscript by Ren and co-workers describes a phosphine oxide organocatalyst derived from L-pyroglutaminol for the kinetic resolution (KR) of racemic amino acids via enantioselective esterification. Using N-protected racemic amino acids and L-pyroglutaminol as substrates, the authors achieved highly enantioselective synthesis of over 30 natural and non-natural amino acids, demonstrated scalability to 5 mmol, and provided supporting evidence for a double hydrogen-bonding interaction in the catalytic mechanism through control experiments and DFT calculations. However, in terms of conceptual novelty and originality, I do not believe this work meets the standards of Nature Communications for the following reasons:

1) Numerous strategies for stereoselective synthesis of chiral amino acids have been reported, including catalytic asymmetric hydrolysis of amino acid esters that not mentioned in the manuscript. These approaches enable stereoconvergent synthesis of chiral amino acids. In comparison, the KR strategy described here does not demonstrate clear superiority in terms of concept, mechanism, or application, particularly given the inherent limitation of maximum 50% theoretical yield for KR processes.
2) The authors employ optically pure L-pyroglutaminol as a template substrate but fail to address how its chiral center influences stereoselectivity. For instance, in Table 1, the use of the enantiomeric catalyst 3f resulted in reduced stereoselectivity (53:47 dr) rather than producing the opposite enantiomer of the product. This raises critical questions: Does the chirality of L-pyroglutaminol play a decisive role in stereocontrol? If so, does this diminish the significance of the catalyst itself in governing enantioselectivity? Further mechanistic studies (e.g., using racemic or D-pyroglutaminol) are necessary to disentangle the contributions of the substrate and catalyst to stereoselectivity.
3) The assertion that this work represents the "first KR of amino acids" overlooks prior enzymatic KR strategies for amino

acids. Such omissions weaken the manuscript's scholarly context. Moreover, the claim that "only CO, CO_2, and HCl are generated as waste" is misleading, as it neglects the stoichiometric use of triethylamine, which generates ammonium salts as byproducts.

Overall, though the work is technically sound and represents a valid contribution to the field of organocatalytic KR, the elements of innovation and broad appeal required for Nature Communications are insufficiently demonstrated. The study would be more appropriately suited for a specialized journal focusing on asymmetric catalysis or synthetic methodology.

Reviewer #3

(Remarks to the Author)
Ren and co-workers' manuscript reports a kinetic resolution of racemic amino acids using a newly developed L-pyroglutaminol-derived phosphine oxide organocatalyst. The methodology is broadly applicable and delivers good stereoselectivities. To investigate the operative mechanism and stereocontrol, the authors used control experiments and DFT computations. While this work presents an interesting new synthetic methodology, especially for non-natural amino acids, the reviewer questions whether it currently meets the broad appeal required for publication in Nature Communications. I recommend publication only after the authors fully address the major issues outlined below.

1. The role of oxalyl chloride and its reaction/interaction with the phosphine oxide catalyst is crucial for the overall mechanism and selectivity. A more detailed mechanistic proposal, supported, for example, by spectroscopic evidence (low-T NMR?) for intermediates A and B (Figure 6), would significantly strengthen the manuscript.

2. The authors should compute the full reaction profile, rather than limiting their analysis to only the selectivity-determining transition states.

3. The authors state in the Supporting Information that they used "dispersion-corrected" DFT. However, B3LYP is not dispersion-corrected by default. The authors should clarify whether a dispersion correction was applied and, if so, specify which one.

4. The manuscript could benefit from a more explicit discussion of the methodology's limitations. For example, how do electron-rich vs. electron-poor substrates or sterically hindered systems perform? Are there known classes of amino acids where the method fails?

5. The authors should include a short comparison with existing KR methods for amino acids (e.g., enzymatic or transition-metal-catalyzed routes), which would help contextualize this organocatalytic method's practical advantages.

6. While the manuscript is generally well-written, some grammatical errors and awkward phrasing should be corrected (e.g., "reaction completed in less than 10 min" → "the reaction was completed within 10 minutes").

Version 1:

Reviewer comments:

Reviewer #1

(Remarks to the Author)
The authors have made nice revision and conducted necessary addtiional experiments to address all the concerns from the reviewers, and I suggest accepting the paper.

Reviewer #2

(Remarks to the Author)
After thorough evaluation of the authors' point-by-point response and the revised manuscript with supporting materials, I appreciate their substantial efforts in addressing reviewers' concerns. While I still maintain that the core concept of this work (i.e. phosphine oxide catalysis for stereoselective amino acid synthesis) lacks transformative novelty given the extensive prior literature in both organocatalytic KR process and amino acid derivatization, the authors' rigorous engagement with all specific technical queries raised by myself and other 2 reviewers is recognized. Nevertheless, several critical issues still require resolution:
Although the authors supplemented experiments using (R)-2a (Fig. 4e) demonstrate stereochemical inversion, they remain inadequate to conclusively elucidate the origin of stereocontrol. More critically, the proposed double hydrogen-bonding interaction—central to the catalytic mechanism—cannot be definitively proven through substrate/catalyst analogue studies alone. To resolve these mechanistic ambiguities, nonlinear effect studies are strongly recommended to unravel in-depth chiral-induction model, complemented by NMR titration to characterize hydrogen-bonding thermodynamics under reaction-like conditions. Moreover, regarding methodological transparency, section 2.2 of the Supporting Information ("Optimization studies") requires significant expansion to enhance methodological transparency. Specifically: Rationale for initial catalyst design choices must be clarified. Was the racemic phosphine oxide catalyst evaluated during preliminary screening? If not, justification for its exclusion should be provided. The solvent screening table referenced in the main text must be reproduced in full within the SI, including all tested solvents and their corresponding selectivity factors (s). Finally, the current transition-

state models (Figures 5–7) remain suboptimally presented, impeding mechanistic comprehension and necessitating additional optimization for clarity.

Reviewer #3

(Remarks to the Author)
The authors have addressed all of my concerns. I have only one very minor correction. As stated in their rebuttal, the authors used Grimme's DFT-D3 method, but the manuscript still reports: “B3LYP/6-311G(d,p)/SMD(CHCl3)”. This should be corrected to: “B3LYP-D3/6-311G(d,p)/SMD(CHCl3)”.

Version 2:

Reviewer comments:

Reviewer #2

(Remarks to the Author)
The reviewer appreciates the authors' efforts to improve the manuscript, and the revised version is certainly an improvement over the previous one. However, my key concern regarding the interaction mode between the catalyst and the substrate has not been fully addressed.
Although the authors have supplemented the manuscript with a 1H NMR titration experiment at a 1:1 molar ratio of catalyst to substrate, the previously suggested nonlinear effect study has not been implemented. While the added NMR data do indicate an interaction between the catalyst and the substrate, they do not provide further insight into the specific binding mode. In this context, a nonlinear effect experiment remains highly important and informative for elucidating the nature of the interaction. Therefore, I strongly recommend that this experiment be carried out before the manuscript can be considered for acceptance.

Version 3:

Reviewer comments:

Reviewer #2

(Remarks to the Author)
As noted previously, the detailed reaction mechanism and interaction model require further experimental validation. Through supplementary nonlinear effect experiments, a positive nonlinear effect was observed, which may suggest the existence of a non-1:1 catalyst-substrate interaction mode. However, the authors did not provide further discussion or explanation for this experimental phenomenon, especially since it was inconsistent with the transition-state model proposed in Figs. 5–7. Therefore, the current version of the manuscript, in my assessment, falls short of the completeness and rigor expected for a publishable contribution.

Version 4:

Reviewer comments:

Reviewer #2

(Remarks to the Author)
Thanks for the authors' diligent work in addressing the points raised in my previous review. The additional control experiments and the nuanced interpretation offered have satisfactorily resolved my concerns about the reaction mechanism. The authors' explanation for the origin of the nonlinear effect is compelling and significantly strengthens the manuscript.
I believe the revised manuscript, with these supplements, now meets the high standards required for publication. I support its acceptance in its present form.

Open Access This Peer Review File is licensed under a Creative Commons Attribution 4.0 International License, which permits use, sharing, adaptation, distribution and reproduction in any medium or format, as long as you give appropriate credit to the original author(s) and the source, provide a link to the Creative Commons license, and indicate if changes were made.
In cases where reviewers are anonymous, credit should be given to 'Anonymous Referee' and the source.
The images or other third party material in this Peer Review File are included in the article’s Creative Commons license, unless indicated otherwise in a credit line to the material. If material is not included in the article’s Creative Commons license and your intended use is not permitted by statutory regulation or exceeds the permitted use, you will need to obtain permission directly from the copyright holder.
To view a copy of this license, visit https://creativecommons.org/licenses/by/4.0/

August 18, 2025

Dear Reviewers,

Thank you very much for your insightful comments of our manuscript entitled "*Kinetic Resolution of Amino Acids by Phosphine Oxide Catalyzed Enantioselective Esterification*". We have revised this manuscript according to your kind advices and useful suggestions. Revisions in the manuscript have been highlighted in yellow. We have copied and pasted all reviewers' comments below, and addressed each one individually. As you will see, we have made every attempt to incorporate all suggestions as thoroughly as possible. We sincerely hope this manuscript will be finally acceptable to be published on *Nature Communications*.

Again, we would like to express our sincere thanks to all the reviewers for their constructive comments. The following changes have been made to address all the comments (*reviewers' comments in blue and italic*).

Reviewer 1:

Comments:

Ren and co-workers report a highly efficient kinetic resolution (KR) of racemic amino acids with L-pyroglutaminol as an esterification reagent. The process is catalyzed by a phosphine oxide organocatalyst under mild conditions and provides a wide range of chiral esters and recovered amino acids with excellent stereoselectivities. The intimate double H-bonding interaction between the pyroglutaminol core and the catalyst is proposed based on control experiments and DFT calculations. Amino acids are undoubtedly an important class of compounds in chemistry and biology. Although many methods have been developed to make enantioenriched amino acids, kinetic resolution methods have been less developed. This work provides a practical and general solution to the problem. The newly designed phosphine oxide catalyst is a highlight of the work. The efficient resolution of a series of unnatural amino acids

shows the importance of the work. I suggest accepting the paper after the following issues being addressed.

Response: We really appreciate the reviewer's positive comments on our work.

1) In Fig 2, actually there are only examples of amino acids with trisubstituted stereocenters. So, the representative reaction equation should be revised.

Response: Per the reviewer's suggestion, the representative reaction equation has been revised in the revised manuscript.

2) This reviewer is interested in access to amino acids bearing tetrasubstituted stereocenters. But there is only one example. It will be very nice to see more examples.

Response: Per the reviewer's suggestion, more amino acids bearing tetrasubstituted stereocenters were tested and these results have been included in the revised manuscript and supporting information.

3) A control experiment using a compound removing the carbonyl oxygen of (S)-2a should be conducted to the importance of the carbonyl group.

Response: Per the reviewer's suggestion, *L*-prolinol **2c** was tested. Not surprisingly, the corresponding ester **4r** was obtained in markedly reduced diastereoselectivity. These results have been included in the revised manuscript and supporting information.

Reviewer 2:

Comments:

The manuscript by Ren and co-workers describes a phosphine oxide organocatalyst derived from L-pyroglutaminol for the kinetic resolution (KR) of racemic amino acids via enantioselective esterification. Using N-protected racemic amino acids and L-pyroglutaminol as substrates, the authors achieved highly enantioselective synthesis of over 30 natural and non-natural amino acids, demonstrated scalability to 5 mmol, and provided supporting evidence for a double hydrogen-bonding interaction in the

catalytic mechanism through control experiments and DFT calculations. However, in terms of conceptual novelty and originality, I do not believe this work meets the standards of Nature Communications for the following reasons.

Response: We really appreciate the reviewer's positive comments on our work.

1) Numerous strategies for stereoselective synthesis of chiral amino acids have been reported, including catalytic asymmetric hydrolysis of amino acid esters that not mentioned in the manuscript. These approaches enable stereoconvergent synthesis of chiral amino acids. In comparison, the KR strategy described here does not demonstrate clear superiority in terms of concept, mechanism, or application, particularly given the inherent limitation of maximum 50% theoretical yield for KR processes.

Response: Per the reviewer's suggestion, the asymmetric hydrolysis of amino acid esters and the corresponding reference have been added in the revised manuscript. While numerous strategies for stereoselective synthesis of chiral amino acids achieve theoretical 100% yield, they often exhibit limited scope for sterically hindered or non-natural amino acids. In contrast, our KR strategy leverages a novel double H-bonding mechanism between the phosphine oxide catalyst and pyroglutaminol, enabling precise stereocontrol (s > 1057) unmatched by enzymatic KR. In addition, our KR strategy accommodates challenging substrates, including α,α-dialkyl substituted amino acids and side-chain-functionalized variants, which are often problematic for enzymatic systems due to steric constraints or sensitivity.

The inherent 50% yield limitation of KR is mitigated by three key advantages. (1) Simultaneous access to both enantiopure products: The recovered amino acids (up to >99% ee) and the chiral esters (up to >99:1 dr) are both high-value building blocks (for peptidomimetics or asymmetric catalysis). (2) Unprecedented efficiency: Reactions were completed within 10 minutes (vs. hours/days for enzymatic systems), with exceptional selectivity (s > 1057). (3) Scalability and robustness: Demonstrated at gram-scale without sensitivity to ambient conditions (unlike biocatalysts, which require strict pH/temperature control).

In summary, our KR strategy offers a complementary approach to stereoselective methods by combining rapidity, excellent selectivities of amino acids with operational simplicity and scalability—addressing gaps in efficiency and scope for structurally diverse substrates. Therefore, this work should be of wide interest to the community of organic chemistry. We have revised the manuscript to more explicitly position our work within the broader landscape of amino acid synthesis strategies.

2) The authors employ optically pure L-pyroglutaminol as a template substrate but fail to address how its chiral center influences stereoselectivity. For instance, in Table 1, the use of the enantiomeric catalyst 3f resulted in reduced stereoselectivity (53:47 dr) rather than producing the opposite enantiomer of the product. This raises critical questions: Does the chirality of L-pyroglutaminol play a decisive role in stereocontrol? If so, does this diminish the significance of the catalyst itself in governing enantioselectivity? Further mechanistic studies (e.g., using racemic or D-pyroglutaminol) are necessary to disentangle the contributions of the substrate and catalyst to stereoselectivity.

Response: Per the reviewer's suggestion, the KR of **1a** was carried out using catalyst **3f** and *D*-pyroglutaminol (*R*)-**2a** provided the enantiomer (*R*,*R*)-**4a** with 99:1 dr and the recovered (*S*)-**1a** with >99% ee, with excellent selectivity factor (s > 529) (Fig. 4e). These results demonstrated that the chiral environments of both the catalyst and pyroglutaminol synergistically govern the stereoselectivity. The corresponding results and details have been included in the revised manuscript and supporting information.

3) The assertion that this work represents the "first KR of amino acids" overlooks prior enzymatic KR strategies for amino acids. Such omissions weaken the manuscript's scholarly context. Moreover, the claim that "only CO, CO_2, and HCl are generated as waste" is misleading, as it neglects the stoichiometric use of triethylamine, which generates ammonium salts as byproducts.

Response: Per the reviewer's suggestion, the corresponding changes have been made in the revised manuscript.

4) Overall, though the work is technically sound and represents a valid contribution to the field of organocatalytic KR, the elements of innovation and broad appeal required for Nature Communications are insufficiently demonstrated. The study would be more appropriately suited for a specialized journal focusing on asymmetric catalysis or synthetic methodology.

Response: We really appreciate the reviewer's positive comments on our work. We respectfully contend that our work demonstrates conceptual innovation, mechanistic novelty, and broad interdisciplinary appeal that align with *Nature Communications*' scope. Below, we highlight three key aspects underscoring its significance.

(1) Our study introduces the first *L*-pyroglutaminol-derived phosphine oxide organocatalyst, which leverages a unique double H-bonding interaction to achieve unprecedented stereocontrol (s > 1057). The catalyst's design-integrating a chiral lactam H-bonding motif with a phosphine oxide activator-enables simultaneous substrate recognition and activation, a strategy unreported in amino acid KR.

(2) Our method achieves excellent selectivity (s > 1057) and ultrafast resolution (10 min) for amino acid KR. In addition, our method accommodates broad substrate diversity-including natural/non-natural amino acids, sterically demanding α,α-dialkyl substituted amino acids.

(3) The work bridges organic synthesis, catalyst design, and chemical biology. Gram-scale synthesis delivers both enantiopure amino acids (e.g., drug intermediates) and chiral pyroglutaminol esters (e.g., peptidomimetic precursors). DFT studies reveal H-bonding dominated enantiocontrol, offering a blueprint for designing lactam-based organocatalysts—a nascent area with untapped potential. Enantioenriched non-natural amino acids are pivotal in peptide therapeutics and biocatalysis, and our method provides rapid, general access to these motifs.

While specialized methodology journals focus on catalytic efficiency, our work advances fundamental questions in stereoselective catalysis (H-bonding networks in enantioselection) and addresses practical limitations in biocatalysis (enzyme instability and narrow scope). This dual impact aligns with *Nature Communications*' emphasis on "scientific advances that represent a significant breakthrough in their

field".

Reviewer 3:

Comments:

Ren and co-workers' manuscript reports a kinetic resolution of racemic amino acids using a newly developed L-pyroglutaminol-derived phosphine oxide organocatalyst. The methodology is broadly applicable and delivers good stereoselectivities. To investigate the operative mechanism and stereocontrol, the authors used control experiments and DFT computations. While this work presents an interesting new synthetic methodology, especially for non-natural amino acids, the reviewer questions whether it currently meets the broad appeal required for publication in Nature Communications. I recommend publication only after the authors fully address the major issues outlined below.

Response: We really appreciate the reviewer's positive comments on our work.

1) The role of oxalyl chloride and its reaction/interaction with the phosphine oxide catalyst is crucial for the overall mechanism and selectivity. A more detailed mechanistic proposal, supported, for example, by spectroscopic evidence (low-T NMR?) for intermediates A and B (Figure 6), would significantly strengthen the manuscript.

Response: Per the reviewer's suggestion, the intermediates **A** and **B** in Figure 6 were successfully characterized by high-resolution mass spectrometry, which tentatively supports the proposed reaction mechanism. The corresponding results and details have been included in the revised manuscript and supporting information.

2) The authors should compute the full reaction profile, rather than limiting their analysis to only the selectivity-determining transition states.

Response: Per the reviewer's suggestion, relative energy profiles for the reaction pathway have been added. A detailed discussion has been included in the revised manuscript and supporting information.

3) The authors state in the Supporting Information that they used "dispersion-corrected" DFT. However, B3LYP is not dispersion-corrected by default. The authors should clarify whether a dispersion correction was applied and, if so, specify which one.

Response: Actually, the dispersion correction was applied. The Grimme's DFT-D3 method was applied to correct the van der Waals interactions for all molecules. The corresponding changes have been made in the revised supporting information.

4) The manuscript could benefit from a more explicit discussion of the methodology's limitations. For example, how do electron-rich vs. electron-poor substrates or sterically hindered systems perform? Are there known classes of amino acids where the method fails?

Response: Per the reviewer's suggestion, a more explicit discussion and unsuccessful cases have been added in the revised manuscript.

5) The authors should include a short comparison with existing KR methods for amino acids (e.g., enzymatic or transition-metal-catalyzed routes), which would help contextualize this organocatalytic method's practical advantages.

Response: Per the reviewer's suggestion, a short comparison with existing KR methods has been included in the revised manuscript. Compared to existing methods, this organocatalytic approach offers distinct advantages in efficiency, scope, and practicality. Enzymatic KR (e.g., using hydrolases/deaminases) typically achieves moderate selectivity (s ≈ 20-200) but suffers from narrow substrate scope (limited to natural amino acids), sensitivity to pH/temperature, and high enzyme costs.[53-57] In contrast, our method achieves unprecedented selectivity (s up to 1057) within 10 minutes under mild conditions, accommodates broad substrate diversity-including natural/non-natural amino acids, sterically demanding α,α-dialkyl substituted amino acids. While enzymatic KR remains viable for aqueous biotransformations, this organocatalytic system bridges critical gaps in efficiency, versatility, and scalability, providing a practical, sustainable alternative for synthesizing enantiopure amino acid

building blocks.

6) While the manuscript is generally well-written, some grammatical errors and awkward phrasing should be corrected (e.g., "reaction completed in less than 10 min" → "the reaction was completed within 10 minutes").

Response: Per the reviewer's suggestion, the error has been thoroughly checked and corrected in the revised manuscript.

If you have any further questions about this manuscript, please do not hesitate to contact me. Thank you very much for all your help and looking forward to hearing from you soon.

Sincerely,

Ji-Wei Ren, Ph. D.
College of Chemistry and Chemical Engineering
Taishan University
Tai'an, Shandong, 271000, P. R. China.
E-mail: renjiwei@tsu.edu.cn

November 14, 2025

Dear Reviewers,

Thank you very much for your insightful comments of our manuscript entitled "*Kinetic Resolution of Amino Acids by Phosphine Oxide Catalyzed Enantioselective Esterification*". We have revised this manuscript according to your kind advices and useful suggestions. Revisions in the manuscript have been highlighted in yellow. We have copied and pasted all reviewers' comments below, and addressed each one individually. As you will see, we have made every attempt to incorporate all suggestions as thoroughly as possible. We sincerely hope this manuscript will be finally acceptable to be published on *Nature Communications*.

Again, we would like to express our sincere thanks to all the reviewers for their constructive comments. The following changes have been made to address all the comments (*reviewers' comments in blue and italic*).

Reviewer 1:

Comments:

The authors have made nice revision and conducted necessary addtiional experiments to address all the concerns from the reviewers, and I suggest accepting the paper.

Response: We really appreciate the reviewer's positive comments on our work.

Reviewer 2:

Comments:

After thorough evaluation of the authors' point-by-point response and the revised manuscript with supporting materials, I appreciate their substantial efforts in addressing reviewers' concerns. While I still maintain that the core concept of this work (i.e. phosphine oxide catalysis for stereoselective amino acid synthesis) lacks transformative novelty given the extensive prior literature in both organocatalytic KR

process and amino acid derivatization, the authors' rigorous engagement with all specific technical queries raised by myself and other 2 reviewers is recognized. Nevertheless, several critical issues still require resolution.

Response: We really appreciate the reviewer’s positive comments on our work.

1) Although the authors supplemented experiments using (R)-2a (Fig. 4e) demonstrate stereochemical inversion, they remain inadequate to conclusively elucidate the origin of stereocontrol. More critically, the proposed double hydrogen-bonding interaction—central to the catalytic mechanism—cannot be definitively proven through substrate/catalyst analogue studies alone. To resolve these mechanistic ambiguities, nonlinear effect studies are strongly recommended to unravel in-depth chiral-induction model, complemented by NMR titration to characterize hydrogen-bonding thermodynamics under reaction-like conditions.

Response: Per the reviewer’s suggestion, ^{1}H NMR spectroscopic studies on the mixture of (*S*)-**2a** with **3a** with a molar ratio of 1:1 were tested and these results have been included in the revised manuscript and supporting information.

2) Moreover, regarding methodological transparency, section 2.2 of the Supporting Information ("Optimization studies") requires significant expansion to enhance methodological transparency. Specifically: Rationale for initial catalyst design choices must be clarified. Was the racemic phosphine oxide catalyst evaluated during preliminary screening? If not, justification for its exclusion should be provided. The solvent screening table referenced in the main text must be reproduced in full within the SI, including all tested solvents and their corresponding selectivity factors (s).

Response: Per the reviewer’s suggestion, the corresponding changes have been made in the revised supporting information.

3) Finally, the current transition-state models (Figures 5–7) remain suboptimally presented, impeding mechanistic comprehension and necessitating additional optimization for clarity.

Response: Thank you for your valuable comment on the presentation of the transition-state (TS) models in Figures 5-7; we fully agree that a clear and optimized TS model is crucial for facilitating mechanistic comprehension and appreciate your insight that helps improve this work. As detailed in the manuscript, the current TS models are rigorously derived from a combination of experimental results and density functional theory (DFT) calculations: control experiments (Figure 4b-4e) confirmed that the double H-bonding interaction between the lactam moieties of *L*-pyroglutaminol and the phosphine oxide catalyst is essential for stereocontrol (masking H-bonding sites significantly reduced diastereoselectivity), while DFT calculations at the B3LYP-D3/6-311G(d,p)/SMD($CHCl_3$) level yielded relative free energies of key TSs (e.g., (*Si*,*S*)-TS2 with ΔG = 6.0 kcal/mol) that align with experimental selectivity (s > 1057) and product configuration ((*S*,*S*)-**4a** as the dominant isomer), verifying the models' rationality. Therefore, the transition-state models in Figures 5-7 are still being kept and remains unchanged in the revised manuscript.

Reviewer 3:

Comments:

The authors have addressed all of my concerns.

Response: We really appreciate the reviewer's positive comments on our work.

1) I have only one very minor correction. As stated in their rebuttal, the authors used Grimme's DFT-D3 method, but the manuscript still reports: "B3LYP/6-311G(d,p)/SMD(CHCl3)". This should be corrected to: "B3LYP-D3/6-311G(d,p)/SMD(CHCl3)".

Response: Per the reviewer's suggestion, the corresponding change has been made in the revised manuscript.

If you have any further questions about this manuscript, please do not hesitate to contact me. Thank you very much for all your help and looking forward to hearing

from you soon.

Sincerely,

Ji-Wei Ren, Ph. D.
College of Chemistry and Chemical Engineering
Taishan University
Tai'an, Shandong, 271000, P. R. China.
E-mail: renjiwei@tsu.edu.cn

December 20, 2025

Dear Reviewer,

Thank you very much for your insightful comments of our manuscript entitled "*Kinetic Resolution of Amino Acids by Phosphine Oxide Catalyzed Enantioselective Esterification*". We have revised this manuscript according to your kind advices and useful suggestions. Revisions in the manuscript have been highlighted in yellow. We have copied and pasted all reviewers' comments below, and addressed each one individually. As you will see, we have made every attempt to incorporate all suggestions as thoroughly as possible. We sincerely hope this manuscript will be finally acceptable to be published on *Nature Communications*.

Again, we would like to express our sincere thanks to all the reviewers for their constructive comments. The following changes have been made to address all the comments (*reviewers' comments in blue and italic*).

Reviewer 2:

Comments:

The reviewer appreciates the authors' efforts to improve the manuscript, and the revised version is certainly an improvement over the previous one. However, my key concern regarding the interaction mode between the catalyst and the substrate has not been fully addressed.

Response: We really appreciate the reviewer's positive comments on our work.

1) Although the authors have supplemented the manuscript with a ^{1}H NMR titration experiment at a 1:1 molar ratio of catalyst to substrate, the previously suggested nonlinear effect study has not been implemented. While the added NMR data do indicate an interaction between the catalyst and the substrate, they do not provide further insight into the specific binding mode. In this context, a nonlinear effect

experiment remains highly important and informative for elucidating the nature of the interaction. Therefore, I strongly recommend that this experiment be carried out before the manuscript can be considered for acceptance.

Response: Per the reviewer's suggestion, we carried out a nonlinear effect experiment for elucidating the nature of the interaction. We observed a small but positive nonlinear effect, which indicates that more than one molecule of **3a** was likely to be involved in the transition state of the enantiodifferentiating step. These results have been included in the revised manuscript and supporting information.

If you have any further questions about this manuscript, please do not hesitate to contact me. Thank you very much for all your help and looking forward to hearing from you soon.

Sincerely,

Ji-Wei Ren, Ph. D.
College of Chemistry and Chemical Engineering
Taishan University
Tai'an, Shandong, 271000, P. R. China.
E-mail: renjiwei@tsu.edu.cn

January 27, 2026

Dear Reviewer,

Thank you very much for your insightful comments of our manuscript entitled "*Kinetic Resolution of Amino Acids by Phosphine Oxide Catalyzed Enantioselective Esterification*". We have revised this manuscript according to your kind advices and useful suggestions. Revisions in the manuscript have been highlighted in yellow. We have copied and pasted all reviewers' comments below, and addressed each one individually. As you will see, we have made every attempt to incorporate all suggestions as thoroughly as possible. We sincerely hope this manuscript will be finally acceptable to be published on *Nature Communications*.

Again, we would like to express our sincere thanks to all the reviewers for their constructive comments. The following changes have been made to address all the comments (*reviewers' comments in blue and italic*).

Reviewer 2:

1) As noted previously, the detailed reaction mechanism and interaction model require further experimental validation. Through supplementary nonlinear effect experiments, a positive nonlinear effect was observed, which may suggest the existence of a non-1:1 catalyst-substrate interaction mode. However, the authors did not provide further discussion or explanation for this experimental phenomenon, especially since it was inconsistent with the transition-state model proposed in Figs. 5–7. Therefore, the current version of the manuscript, in my assessment, falls short of the completeness and rigor expected for a publishable contribution.

Response: We sincerely thank the reviewer for this profound and insightful comment, which prompted us to perform additional experiments that have led to a more nuanced and robust understanding of our system. To directly address the concern regarding the positive nonlinear effect and its implication for the reaction mechanism, we conducted

a decisive control experiment: we examined the kinetic resolution using enantiopure catalyst **3a** while systematically varying the enantiomeric purity of the chiral reagent (*S*)-**2a**. The results are unequivocal. The enantiomeric excess of the recovered amino acid (*R*)-**1a** increased nonlinearly from 53% ee (when **2a** was racemic) to 99% ee (when **2a** was enantiopure). This nonlinear relationship is critical for three reasons. First, the baseline selectivity of 53% ee with racemic **2a** proves that the catalyst **3a** itself possesses significant intrinsic enantioselectivity, affirming its role as the primary chiral director. Second, the nonlinear enhancement demonstrates that the chiral **2a** acts as a co-factor, providing a complementary chiral environment that nonlinearly boosts the catalyst's inherent selectivity to exceptional levels. Third, and most importantly, the stark contrast between this nonlinear "substrate nonlinear effect" and the positive nonlinear "catalyst nonlinear effect" (Fig. 4g) allows us to pinpoint the origin of the nonlinearity. This analysis is fully consistent with, and reinforced by, key data already present in the manuscript: (i) the drastic loss of selectivity when the mismatched catalyst **3f** was used with (*S*)-**2a** (Table 1, entry 6), and (ii) the restoration of high selectivity (s > 529) when the chiralities of both components were switched to the matched **3f**/(*R*)-**2a** pair (Fig. 4e). These results had already established the foundational principle of chiral matching for optimal performance. Building on this, we now posit that the observed nonlinear relationship in the original catalyst experiment arises because, when the catalyst's ee is lowered, the introduced mismatched catalyst enantiomer **3f** interacts detrimentally—and in a nonlinear, inhibitory fashion—with the ever-present enantiopure (*S*)-**2a**. This effect disproportionately suppresses the selectivity achieved by the optimal matched pair **3a**/(*S*)-**2a**. Therefore, the nonlinear effect reports on the system-level behavior when non-productive interactions are introduced, while the DFT model (Figs. 5-7) correctly depicts the structure and energetics of the productive pathway involving the matched 1:1 complex. The new data and the unified interpretation do not contradict but rather enrich the mechanistic picture, solidifying the conclusion that the extreme selectivity arises from a synergistic dual-chiral induction. We have incorporated this comprehensive discussion and the new data into the revised manuscript and

supporting information, which we believe now fully addresses the reviewer's concern and meets the expected standards of completeness and rigor.

If you have any further questions about this manuscript, please do not hesitate to contact me. Thank you very much for all your help and looking forward to hearing from you soon.

Sincerely,

Ji-Wei Ren, Ph. D.
College of Chemistry and Chemical Engineering
Taishan University
Tai'an, Shandong, 271000, P. R. China.
E-mail: renjiwei@tsu.edu.cn
